# Profiling the reactivity of cyclic C-nucleophiles towards electrophilic sulfur in cysteine sulfenic acid†
†Electronic supplementary information (ESI) available. See DOI: 10.1039/c5sc02569a



**DOI:** 10.1039/c5sc02569a

**Published:** 2015-10-07

**Authors:** Vinayak Gupta, Kate S. Carroll

**Affiliations:** a Department of Chemistry , The Scripps Research Institute , Jupiter , Florida 33458 , USA . Email: kcarroll@scripps.edu

## Abstract

Oxidation of a protein cysteine thiol to sulfenic acid, termed *S*-sulfenylation, is a reversible post-translational modification that plays a crucial role in regulating protein function and is correlated with disease states. The majority of reaction-based small molecule and immunochemical probes used for detecting sulfenic acids are based on the 5,5-dimethyl-1,3-cyclohexanedione (dimedone) scaffold, which is selective, but suffers from low reactivity. In addition, mechanistic details and features that diminish or enhance nucleophile reactivity remain largely unknown. A significant hurdle to resolving the aforementioned issues has been the chemically unstable nature of small-molecule sulfenic acid models. Herein, we report a facile mass spectrometry-based assay and repurposed dipeptide-based model to screen a library of cyclic C-nucleophiles for reactivity with sulfenic acid under aqueous conditions. Observed rate constants for ∼100 cyclic C-nucleophiles were obtained and, from this collection, we have identified novel compounds with more than 200-fold enhanced reactivity, as compared to dimedone. The increase in reactivity and retention of selectivity of these C-nucleophiles were validated in secondary assays, including a protein model for sulfenic acid. Together, this work represents a significant step toward developing new chemical reporters for detecting protein *S*-sulfenylation with superior kinetic resolution. The enhanced rates and varied composition of the C-nucleophiles should enable more comprehensive analyses of the sulfenome and serve as the foundation for reversible or irreversible nucleophilic covalent inhibitors that target oxidized cysteine residues in therapeutically important proteins.

## Abstract 2

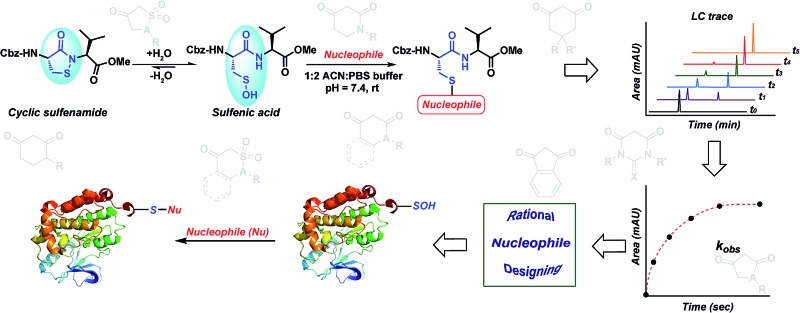
Oxidation of a protein cysteine thiol to sulfenic acid, termed *S*-sulfenylation, is a reversible post-translational modification that plays a crucial role in regulating protein function and is correlated with disease states.

## Introduction

Reactive oxygen species (ROS) are continuously generated, transformed and consumed in living organisms as a consequence of aerobic life. Due to their role in both physiology and pathology, ROS are considered scientific equivalents of “antiheroes”.^1^ Once generated, ROS mediates diverse arrays of reversible and irreversible modifications on biomolecules such as proteins, lipids DNA and RNA.^2,3^ Due to their strong nucleophilic character and low redox potential in proteins (*E* ^o^, –0.27 to –0.125 V) side chain thiol(ate) of cysteines (Cys-SH) are one of the more common targets of ROS.^4^ Indeed, thiolate oxidation by hydrogen peroxide (H_2_O_2_) represents a widely studied area of redox-based post-translational protein modification. Nucleophilic attack of a protein thiolate on electrophilic H_2_O_2_ releases water and results in the formation of cysteine sulfenic acid (Cys-SOH) also known as *S*-sulfenylation. Depending upon the protein microenvironment where the thiolate is located, the rate of oxidation by H_2_O_2_ can vary substantially (1–10^8^ M^–1^ s^–1^). This stark difference in oxidation rates is highlighted by the reaction rates of two major targets of H_2_O_2_ signaling in cells, peroxiredoxin 2 (Prx2; 10^8^ M^–1^ s^–1^) and protein tyrosine phosphatase type 1B (PTP1B; 9 M^–1^ s^–1^).^4,5^ Reversible Cys-SOH formation plays a regulatory role among transcription factors, kinases (EGFR, JAK2, Akt2, IKK-β, RegB, PGKase, L-PYK), phosphatases (PTP1B, YopH, PTEN, Cdc25a, SHP-1 and SHP-2), ion channels, peroxidases and cysteine proteases, human serum albumin (HSA) and many other proteins.^6–20^ Moreover, aberrant *S*-sulfenylation correlates with tumor progression and can lead to noncanonical scurvy in mice.^10,21^ The aforesaid examples and many other reports demonstrate that protein *S*-sulfenylation constitutes a global signal mechanism, not unlike phosphorylation.

**Fig. 1:**
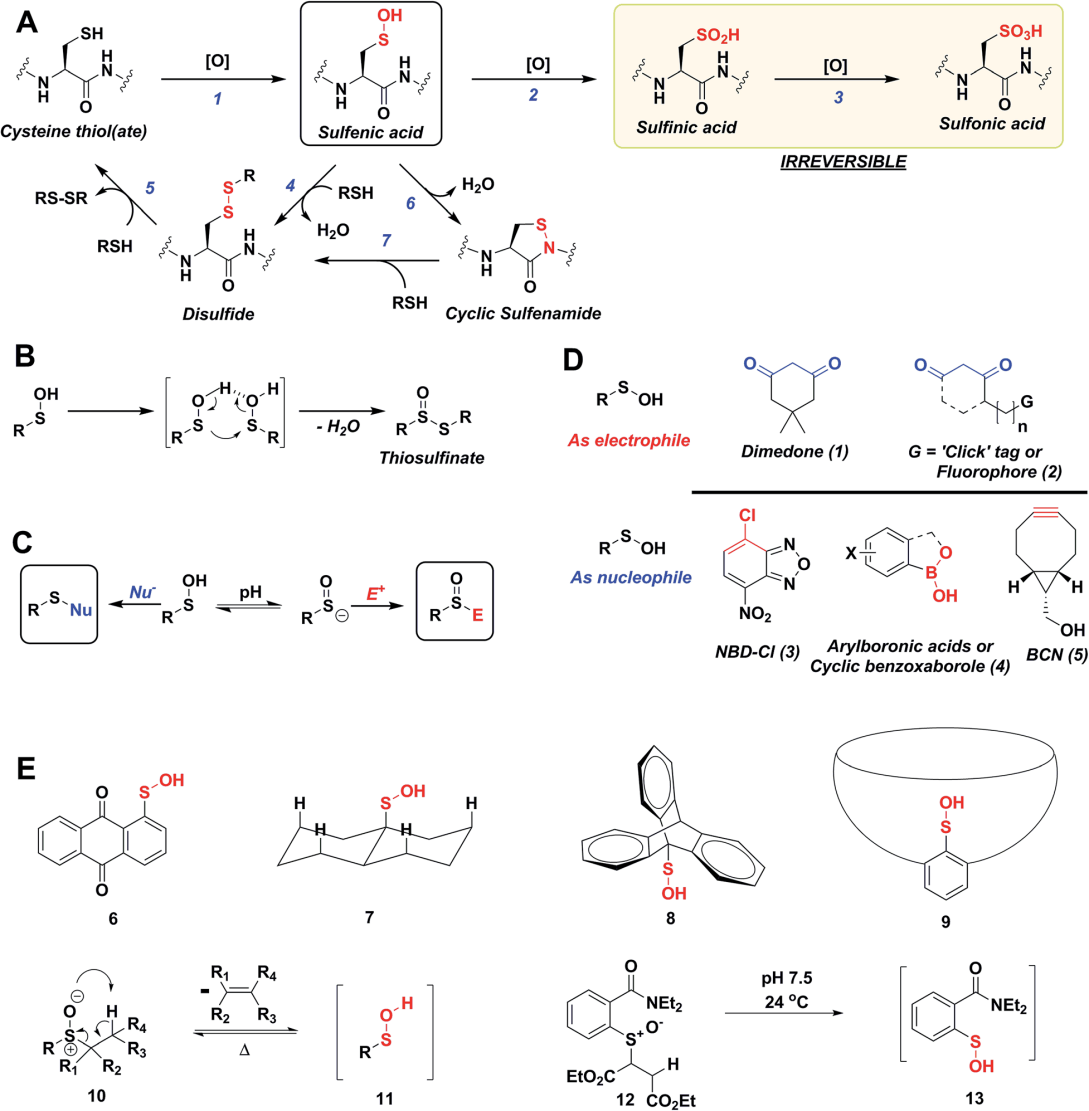
(A) Biological cysteine oxoforms. (B) Sulfenic acid acts as a nucleophile and an electrophile. (C) Nucleophilic probes (Nu^–^) result in the formation of a thioether-type linkage and electrophilic probes (E^+^) result in the formation of a sulfoxide. (D) General structures of nucleophilic and electrophilic sulfenic acid probes. (E) Examples of currently known stable and transient small-molecule sulfenic acids.

The cellular lifetime of Cys-SOH depends on numerous factors, including the level of ROS and/or duration of ROS signaling as well as the local protein environment. Essentially, the absence of proximal thiols capable of generating an intramolecular disulfide is considered to be a primary stabilizing factor; limited solvent access and proximal hydrogen bond acceptors also contribute toward Cys-SOH stabilization. Cys-SOH is the first oxidation product that results from the reaction between a cysteine thiolate and H_2_O_2_ (Fig. 1A, Reaction 1). High ROS, chronic oxidative stress, and/or the lack of adjacent thiols may cause –SOH to undergo further oxidization to sulfinic (–SO_2_H) or sulfonic acid (–SO_3_H) (Fig. 1A, Reactions 2 and 3). In contrast to biologically reversible Cys-SOH, these higher oxoforms are essentially irreversible (the only exception to this statement has been found to date is with Prx-SO_2_H, which can be reduced to Prx-SH by the ATP-dependent enzyme, sulfiredoxin^22^). An important biological reaction of Cys-SOH is disulfide bond formation. Mechanistically, the electrophilic sulfur atom of Cys-SOH reacts with the thiolate nucleophile to give the disulfide with concomitant loss of water (Fig. 1A, Reaction 4). Due to the abundance of biological thiols (mM levels) including protein and low-molecular weight molecule thiols, such as glutathione (GSH), this reaction can be facile and constitutes a major pathway for disulfide formation. The nascent disulfide may undergo thiol–disulfide exchange to give the initial thiol (Fig. 1A, Reactions 4 and 5). Cys-SOH may also undergo intramolecular reaction with adjacent amide nitrogen, which results in the formation of isothiazolidinone, also known as cyclic sulfenamide (Fig. 1A, Reaction 6).^23,24^ The cyclic sulfenamide species may be reduced back to thiol *via* disulfide formation (Fig. 1A, Reactions 7 and 5). On the basis of the reversible/irreversible reactions that Cys-SOH can undergo, this post-translational modification serves as an important hub within the redox milieu. Accordingly, an important goal to dissect regulatory redox pathways has been to develop robust, sensitive and rapid detection techniques to identify sites, conditions and the cellular lifetime of protein *S*-sulfenyl modifications.^4,6,25–29^

The sulfur atom in sulfenic acid is distinguished from other cysteine redox modifications by its weak nucleophilic and moderate electrophilic reactivity (due to the higher p*K*_a_ leading to lower tendency to form sulfenate anion, they are better electrophiles than nucleophiles). This behavior is epitomized by the tendency of –SOH to self-condense resulting in the formation of a thiosulfinate (Fig. 1B). Detection methods exploiting the electrophilic or the nucleophilic character of Cys-SOH have been reported (Fig. 1C).^4,28,30^ However, the vast majority of probes capitalize on the unique electrophilic character of sulfur atom in Cys-SOH and are based on 5,5-dimethyl-1,3-cyclohexanedione (**1**) or dimedone scaffold.^31^ Dimedone (**1**) and probes based on the cyclic 1,3-dicarbonyl scaffold (**2**) are extensively employed for qualitative and quantitative study of protein *S*-sulfenylation.^12–15,20^ Though they are selective under aqueous physiological conditions, the above probes suffer from poor reaction kinetics when compared with other common biological reactions of Cys-SOH.^4,32^ Conventional electrophilic probes are either slow and cross-react with other biological functionalities (*e.g.*, NBD-Cl (**3**), Fig. 1D)^4,28^ or are reversible (*e.g.*, arylboronic acids (**4**), Fig. 1D).^33^ Recently, however, an electrophilic ring strained alkyne, bicyclo[6.1.0]nonyne (BCN (**5**), Fig. 1D) was shown to react with sulfenic acid at 100-fold higher reaction rate compared to dimedone.^32^ Since protein thiols and persulfides are well documented to readily react with activated alkynes such as **5**, this probe has major chemoselectivity issues.^34–38^ Thus, there is still significant room for exploration and further improvement of chemical probes for qualitatively/quantitatively profiling of cellular protein *S*-sulfenylation.

A significant hurdle to study –SOH reactivity and probe development is the unstable nature of small-molecule sulfenic acid models. In principle, protein sulfenic acid model could be used, however, rates of probe reaction could be biased by the microenvironment surrounding Cys-SOH. For example, a sterically bulky probe may be very reactive, but unable to access Cys-SOH buried in an active-site pocket. Such a case also underscores the importance of developing a suite of probes to profile Cys-SOH, to maximize comprehensive detection of this modification. Existing small molecule sulfenic acid models may be divided into two categories: (i) stable sulfenic acid systems that can be synthesized and stored, and (ii) small-molecule sulfenic acids generated *in situ*. The first category are stabilized through hydrogen bonding (*e.g.* Fig. 1E, **6**, **7**) and/or steric factors (*e.g.* Fig. 1E, **8**, **9**). Like proteins, these structures protect and stabilize the sulfenic acid through the surrounding microenvironment.^4^ Ideally, however, the model should not be unduly influenced by such factors. For this reason, we were more interested in a model wherein the sulfenic acid is generated *in situ*. Although such currently known reactions are highly efficient in generating small molecule sulfenic acids, these reactions either require heat and organic conditions (Fig. 1E, **11**) or are kinetically slow (Fig. 1E, **13**).^4,39^ In the ideal case, we envisaged a cysteine-based small-molecule model that is: (i) straightforward to prepare/store, and (ii) sterically and chemically accessible (*i.e.*, not physically hindered or excessively stabilized by electrostatic interactions). Consequently, the aim of our study was two-fold. First, we wanted to develop a facile small-molecule sulfenic acid model. Second, we wanted to use this model to screen, identify, and kinetically characterize small-molecule C-nucleophiles that react with cysteine sulfenic acid under aqueous conditions.

## Results

### Synthesis and validation of a dipeptide-based sulfenic acid model

**Scheme 1:**
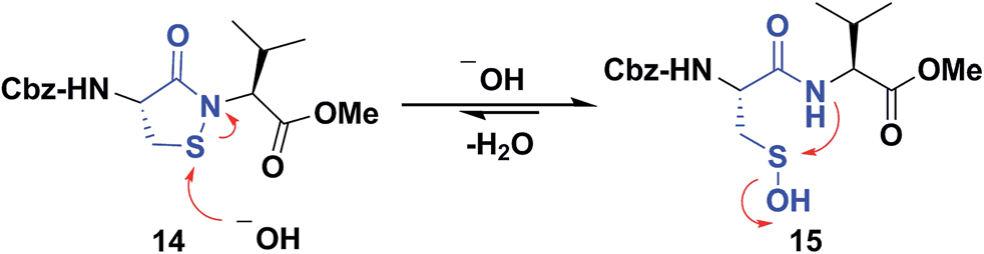
Dipeptide based cyclic sulfenamide model is hypothesized to exist in equilibrium with corresponding sulfenic acid under aqueous conditions.

**Scheme 2:**
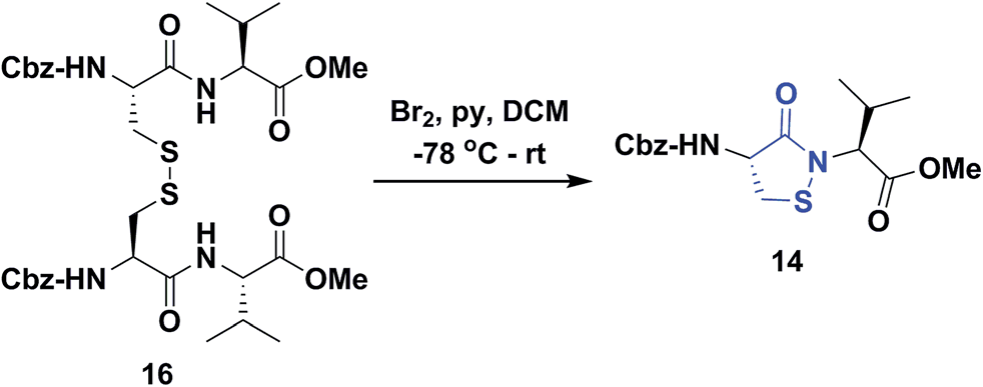
Synthesis of dipeptide based cyclic sulfenamide **14**.

Several literature-reported persistent and transient sulfenic acid models were surveyed, but the example that caught our attention was a dipeptide-based model for its isostere, cyclic sulfenamide (Scheme 1, **14**). Dipeptide **14** was originally reported by Shiau *et al.* at Sunesis pharmaceuticals and employed as a model of cysteine oxidation to cyclic sulfenamide in PTP1B.^40^ Owing to the combination of ring strain and electronic factors, we reasoned that the sulfur of cyclic sulfenamide might also be moderately electrophilic (Scheme 1, **15**). Furthermore, we were curious about the stability of the sulfenamide under aqueous conditions and wondered whether the cyclic structure could be a synthon of sorts, existing in equilibrium with the corresponding sulfenic acid (Scheme 1). The reported synthesis is low in yield but a straight-forward sequence with well-established synthetic precedent for the key oxidative cyclization step.^41^ Even so, following the reported procedure, we obtained the target cyclic sulfenamide (**14**) in poorer and variable yield. Closer analysis of reaction products revealed the presence of precursor disulfide (Cbz-Cys-Val-OMe)_2_ (**16**) and a new compound, identified as cyclic sulfinamide (**17**) (Scheme S1A†). To address the issue of yield and variability, we varied the ratio of bromine to pyridine and avoided the aqueous workup. With these modifications in place, the cyclization step was successfully standardized at gram scale to give the dipeptide based cyclic sulfenamide product in >85% yield after silica gel based column purification (Scheme 2).

With the dipeptide cyclic sulfenamide (**14**) in hand, we next evaluated its stability under aqueous conditions. In these experiments, we observed that dipeptide cyclic sulfenamide (**14**) reacted over time to form cyclic sulfinamide (**17**) and (Cbz-Cys-Val-OMe)_2_ (**16**) (Scheme S1A†). The mechanism shown in Scheme S1B† accounts for the formation of **16** and **17** and is consistent with our proposal that cyclic sulfenamide (**14**) exist in equilibrium with sulfenic acid (**15**) under aqueous conditions. In the absence of other reactive groups, cyclic sulfenamide **14** can be reformed from **15** through attack by nitrogen. In addition, **15** can condense with itself (or cyclic sulfenamide **14**) to give thiosulfinate (**18**) as an intermediate, the eventual rearrangement of which was observed over the time (Scheme S2B†). In subsequent steps, the amide nitrogen nucleophile attacks the electrophilic sulfinyl sulfur, producing cyclic sulfinamide (**17**) and dipeptide thiolate (**19**). Thiolate **19** subsequently reacts with sulfenic acid **15** (or with cyclic sulfenamide **14**) resulting in the formation of dipeptide disulfide **16**. Importantly, the dipeptide cyclic sulfenamide **14** was stable in acetonitrile over the same period of time (and longer) demonstrating that H_2_O is required for decomposition (Scheme S3†). Further chemical evidence for the formation of sulfenic acid (**15**) was obtained through the addition of methyl iodide and NBD-Cl to the reaction, giving corresponding methyl and aryl sulfone respectively (Schemes S4 and S5†).

Since formation of sulfinamide **17** and disulfide **16** has the potential to interfere with downstream kinetic analysis, we determined the second-order rate constant for this reaction (Scheme S2D†). In this analysis, a value of 1.2 M^–1^ s^–1^ was obtained and deemed acceptable given the anticipated rate constants for our assay (see below). Since the rate-limiting step in this rearrangement is formation of **18** and the sulfenate anion is required for facile self-condensation of sulfenic acid, we were presented with the opportunity to determine the p*K*_a_ of sulfenic acid **15**. Pseudo first-order rate constants (*k*_obs_) were obtained for the rearrangement from pH 3–9 (Scheme S6B†). The plot of *k*_obs_ *versus* pH gave a p*K*_a_ value of 7.1 for sulfenic acid **15** (Scheme S6C†). This value agrees well with small-molecule sulfenic acid p*K*_a_s, which generally range between 4 and 8 depending upon their stability.^4^ The measured p*K*_a_ value of 7.1 is significant as it indicates that under our aqueous experimental conditions, sulfenic acid **15** and the corresponding sulfenate anion are present in roughly equal amounts. The existence of both species is required for facile formation of thiosulfinate (**18**), which is clearly observed in our assay. Collectively, the aforementioned data provide strong support for the formation of sulfenic acid **15** under aqueous conditions.

### Validation of the LC-MS assay for screening cyclic C-nucleophiles

**Scheme 3:**
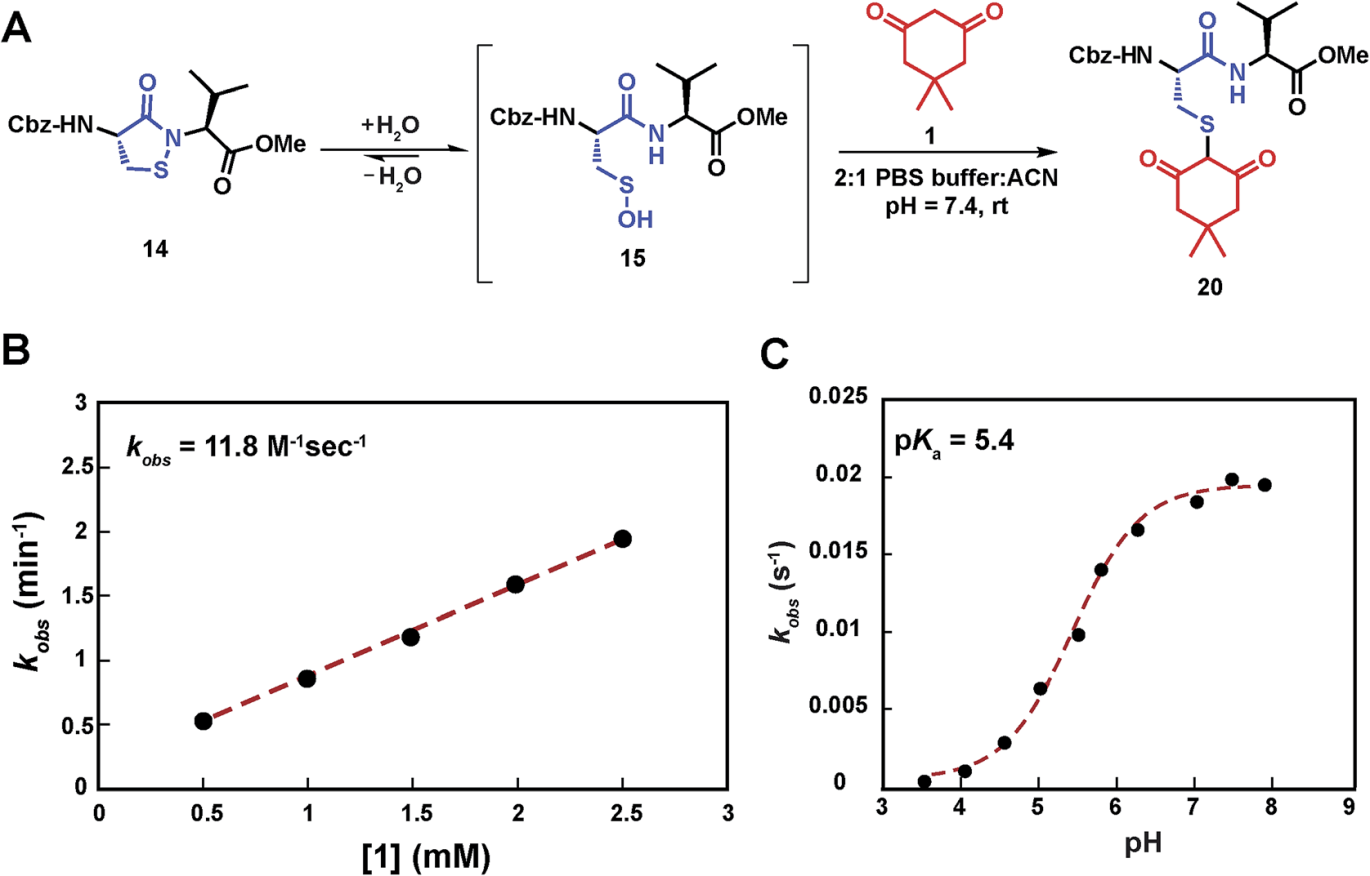
Study of the kinetics of the reaction between sulfenic acid **15** and dimedone **1**. (A) Reaction pathway showing the adduct formation as a result of the reaction of sulfenic acid **15** and dimedone **1**. (B) Pseudo 1^st^ order rate constants at varying concentration of **1** (0.5 –2.5 mM), while keeping concentration of **14** fixed (100 μM) were obtained. *k*_obs_ at different dimedone concentrations were plotted to give the 2^nd^ order rate constant value of 11.8 M^–1^ s^–1^. (C) pH dependence of the reaction of dimedone **1** with sulfenic acid **15** was studied. Pseudo 1^st^ order *k*_obs_ thus obtained were plotted against pH to obtain a sigmoid plot.

Having shown that sulfenic acid **15** forms under aqueous conditions, we next evaluated its ability to react with dimedone **1** to give the expected thioether adduct **20** (Scheme 3A) under pseudo first-order conditions (*i.e.*, ≥10-fold excess of C-nucleophile, Scheme S7C†). The resulting plot of *k*_obs_ *versus* cyclic sulfenamide **14** gave a straight line, the slope of which yielded a second-order rate constant value of 11.8 M^–1^ s^–1^, consistent with the rate constants reported for reaction between dimedone **1** and protein sulfenic acids (Scheme 3B).^4,42^ Additional experiments confirmed that self-condensation of **15** (*i.e.*, the competing background reaction shown in Scheme S1†) was negligible under the conditions of our kinetic assay (≥1 mM dimedone and ≤100 μM cyclic sulfenamide **14**, see also Scheme S7B†).

Compared to cyclic sulfenamide **14**, the sulfur of sulfenic acid **15** is considerably more electrophilic. Even so, it is formally possible that dimedone (**1**) could react with either sulfur center. To identify the reactive specie(s) under our aqueous assay conditions, we investigated the reaction between dimedone (**1**) and two additional sulfenamide models in which sulfenic acid formation was either minimized (*i.e.*, electron-rich cyclic sulfenamide) or absent (*i.e.*, linear sulfenamide). Cyclic sulfenamide ethyl 4-(3-oxobenzo[*d*]isothiazol-2(3*H*)-yl)benzoate (**41**) reacted with dimedone (**1**) to form an adduct (*k*_obs_ = 0.03 min^–1^, Scheme S8†); however, the observed rate was ∼30-fold less than the equivalent reaction with cyclic sulfenamide **14** (*k*_obs_ = 0.8 min^–1^). Linear sulfenamide, methyl 2-(acetamidothio)benzoate (**43**) failed to react with dimedone (**1**), as expected (Scheme S10†). Though sulfenamides **41** and **43** are not perfect experimental models for **14** (*e.g.*, **41** and **43** are more sterically hindered around the sulfur atom) these data are consistent with the hypothesis that sulfenic acid **15** is the reactive species. Furthermore, both **41** and **43** failed to give the diagnostic sulfoxide under aqueous conditions, which is exhibited by **14** and a hallmark of sulfenic acid formation (Schemes S9 and S11†). In addition to the above data, we note the excellent correspondence in second order rate constants for the reaction between dimedone (**1**) and protein sulfenic acids or dipeptide **14**. Lastly, it has been well established that dimedone does not react with the stable cyclic sulfenamide formed in the tyrosine phosphatase, PTP1B.^12,13,15,43^ When taken together, these data support our proposal that sulfenic acid **15** is the major reactive species in our aqueous kinetic assay.

In subsequent studies, we characterized the pH dependence for the reaction of dimedone (**1**) and sulfenic acid **15**. The resulting plot of pH *versus k*_obs_ for formation of thioether **20** was best fit to an equation with a single ionization with a p*K*_a_ value of 5.4 (Scheme 3C). This value matches closely with the p*K*_a_ of dimedone (**1**) obtained in water (p*K*_a_ = 5.2).^44,45^ An analogous experiment was performed with a closely related C-nucleophile, 1,3-cyclopentanedione (**21a**).^46^ In this case, the resulting p*K*_a_ for **21a** gave a value of 4.2, which matches closely with the reported p*K*_a_ in water (p*K*_a_ = 4.3)^47^ (Scheme S12†). Taken together, the data from these experiments suggests that the reaction rate of sulfenic acid **15** and the aforementioned cyclic 1,3-dicarbonyl nucleophiles is influenced by the position of the C-2 acid/base equilibrium. These findings thus substantiate the importance of C-nucleophile p*K*_a_ as an important determinant in the dimedone (**1**) reaction and highlight the utility of our assay to evaluate the reactivity of C-nucleophiles with sulfenic acid.

### Ring size and C-nucleophile reactivity

**Chart 1:**
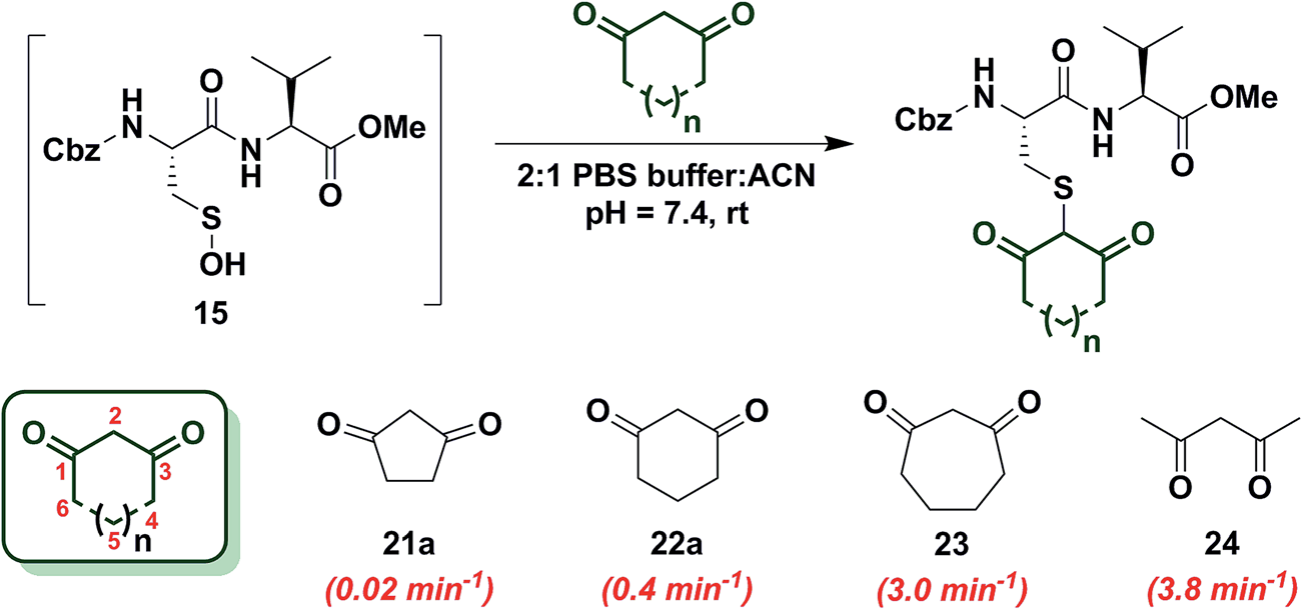
Reaction of sulfenic acid **15** with nucleophiles – effect of ring size.

**Scheme 4:**
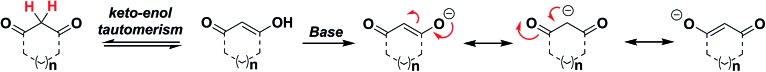
1,3-Dicarbonyls have lower p*K*_a_ (<14) as a result of the resonance stabilization of resulting enolate.^48^

In subsequent studies, we examined the effect of C-nucleophile ring size on reaction rate constants with sulfenic acid. To this end, we selected four commercially available nucleophiles: 1,3-cyclopentanedione (**21a**), 1,3-cyclohexanedione (**22a**), 1,3-cycloheptanedione (**23**) and 2,4-pentanedione (**24**) (Chart 1). The resulting pseudo first-order rate constants show an increase in reactivity with increasing ring size. Due to resonance stabilization of the enolate, the p*K*_a_ of the α-carbon nucleophile in 1,3-dicarbonyls is relatively low (<14) (Scheme 4) and, consequently, these compounds will have varied anionic character at physiological pH. For example, the enol tautomer of **21a** (p*K*_a_ ∼ 4.3) is the dominant form under aqueous conditions at pH 7.4 and its low p*K*_a_ leads to a highly stabilized enolate. Consequently, **21a** has a lower tendency to react with sulfenic acid **15** (*k*_obs_ = 0.02 min^–1^) compared to **22a** (*k*_obs_ = 0.4 min^–1^).

As ring size increases, the p*K*_a_ of the α-carbon rises and the tautomeric equilibrium shifts toward the keto form. Consistent with these properties, **22a** (p*K*_a_ = 5.23)^47^ and **23** showed a respective 20-fold and 150-fold (*k*_obs_ = 3 min^–1^) enhancement in reaction rate constants relative to **21a**. Linear 1,3-dicarbonyl **24** (p*K*_a_ = 8.99),^47^ which favors the keto tautomer by 4 : 1,^48^ displayed a 190-fold (*k*_obs_ = 3.8 min^–1^) rate enhancement compared to **21a**. Together, the observed trend in C-nucleophile reactivity can be rationalized by two principle factors: electronics or α-carbon p*K*_a_ and keto–enol tautomerism.

### C-4 or C-5 alkylation of 1,3-cyclohexanedione (6-membered ring system)

**Chart 2:**
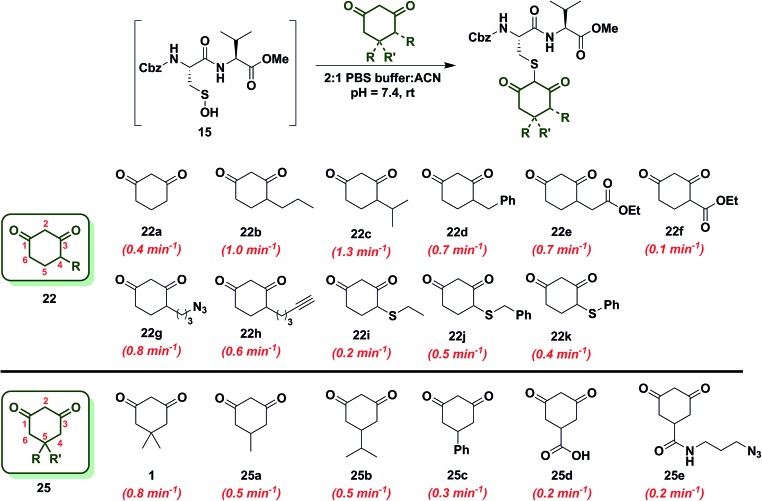
Reaction of sulfenic acid **15** with nucleophiles – effect of C-4 or C-5 alkylation.

Since the change in p*K*_a_ of C-4 or C-5-substituted analogs is minimal (predicted from SciFinder using ACD/Labs software V11.02), changes in *k*_obs_ can be most simply attributed to the electronic effect of substitution by electron donating groups (EDG) or electron withdrawing groups (EWG). With this aspect in mind, we next examined the effect of C-4 or C-5 alkylation on the reactivity of 1,3-cyclohexanedione (**22a**) with sulfenic acid **15**. C-4 analogs **22b–k** were prepared according to the literature^7,12,49–51^ and C-5 derivatives were either commercially procured (**1**, **25a–c**, **e**) or synthesized using a previously reported method^9^ (**25d**) (Scheme S14†). At the C-4 position, straight- and branched-chain alkylation slightly increased reactivity (up to 3-fold faster relative to **22a**, Chart 2). For example, reaction of 4-propylcyclohexane-1,3-dione (**22b**) and 4-isopropylcyclohexane-1,3-dione (**22c**) gave *k*_obs_ equal to 1.0 min^–1^ and 1.3 min^–1^, respectively. 4-Benzylcyclohexane-1,3-dione (**22d**) and ethyl 2-(2,4-dioxocyclohexyl)acetate (**22e**) produced identical *k*_obs_ (0.7 min^–1^). Similarly, azide- and alkyne-functionalized probes for sulfenic acid DAz-2 ^12^ (**22g**) and DYn-2 ^7^ (**22h**) exhibited *k*_obs_ corresponding to 0.8 min^–1^ and 0.6 min^–1^. On the other hand, C-4 substitution with EWGs slightly decreased reactivity (up to 4-fold slower relative to **22a**). For instance, the electron-withdrawing carboxylate ester at C-4 in ethyl 2,4-dioxocyclohexane-1-carboxylate (**22f**) led to a modest decrease in *k*_obs_ (0.1 min^–1^) compared to **22a**. In the case of C-4 alkylthio substitutions, empty sulfur d-orbitals appeared to impart a net electron-withdrawing effect on the 1,3-cyclohexanedione ring.^52^ Consistent with this proposal, 4-(ethylthio)cyclohexane-1,3-dione (**22i**), 4-(benzylthio)cyclohexane-1,3-dione (**22j**) and 4-(phenylthio)cyclohexane-1,3-dione (**22k**) produced *k*_obs_ of 0.2 min^–1^, 0.5 min^–1^ and 0.4 min^–1^ respectively (Chart 2). At the C-5 position, the effect of EDG or EWG substitution was also quite mild (up to a 2-fold increase or decrease in *k*_obs_ compared to **22a**). For example, dimethyl C-5 substitution of **22a** as in dimedone (**1**) led to a two-fold increase in reactivity (0.8 ± 0.03 min^–1^). Both 5-methylcyclohexane-1,3-dione (**25a**) and 5-isopropylcyclohexane-1,3-dione (**25b**) yielded a *k*_obs_ of 0.5 min^–1^; the change in *k*_obs_ for 5-phenylcyclohexane-1,3-dione (**25c**) was also slight (0.3 min^–1^). Lastly, both 3,5-dioxocyclohexane-1-carboxylic acid (**25d**) and DAz-1 ^8,9^ (**25e**) gave *k*_obs_ equal to 0.2 min^–1^ (Chart 2). To summarize, the observed increase or decrease in *k*_obs_ for C-4 or C-5 substituted derivatives was small and can be attributed to the decrease (*e.g.*, substitution with EDG) or increase (*e.g.*, substitution with EWG) in C-2 anion stability.

### Cyclic C-nucleophile heteroatom incorporation

**Chart 3:**
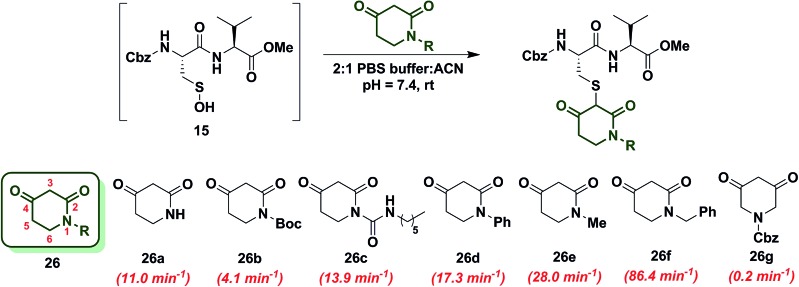
Reaction of sulfenic acid **15** with 2,4-piperidinedione based nucleophiles.

To increase the reactivity of cyclic C-nucleophiles, we next sought to destabilize the C-2 anion and shift the keto–enol equilibrium towards the keto tautomer. To this end, we pursued the substitution of one or more ring C-atoms with heteroatoms, such as nitrogen or oxygen. The most straightforward, commercially available compound was 2,4-piperidinedione (**26a**) and the *k*_obs_ for this reaction was 11 min^–1^ or ∼15-fold faster than dimedone (**1**) (Chart 3). In subsequent studies, novel derivatization methods (*e.g.*, base mediated alkylation, Ullmann-type arylation and alkyl isocyanate-based urea derivatization) were developed in order to functionalize **26a** (Scheme S15†). Urea-, arylated- and alkylated derivatives of **26a** were thus prepared and evaluated for their reactivity with dipeptide sulfenic acid **15**. The *k*_obs_ for reaction of **26b** was almost 3-fold less than **26a**, suggesting that the electron-withdrawing Boc-group stabilizes the C-3 anion. On the other hand, electron-donating urea **26c** (*k*_obs_ = 13.9 min^–1^) and *N*-aryl **26d** (*k*_obs_ = 17.3 min^–1^) derivatives gave ∼20-fold enhancement in reactivity compared to dimedone (**1**). Interestingly, the *k*_obs_ for **26e** was 35-fold faster than dimedone (**1**) implying that simple alkylation is sufficient to destabilize the C-3 anion and enhance its reactivity towards sulfenic acid. *N*-Benzylation (**26f**, *k*_obs_ = 86.4 ± 2.2 min^–1^) augmented reactivity 100-fold compared to dimedone (**1**), underscoring our observation that *N*-alkylation with EDG leads to an increase in reaction rate constants (Chart 3). Identification of piperidine-2,4-dione **26a** as a cyclic C-nucleophile with enhanced reactivity for sulfenic acid represents an important advance, since it is structurally similar to **22a**, but its derivatives exhibit rate enhancements of almost two orders of magnitude relative to dimedone (**1**). Moreover, unlike C-4 alkylation of **22a**, *N*-alkylation (or *N*-arylation) of **26a** is more straightforward from a synthetic point of view. Our findings at C-4, however, did not extend to C-5, as replacement with an *N*-heteroatom (**26g**) afforded a compound with only moderate activity (*k*_obs_ = 0.2 min^–1^) (Chart 3). The reduced reactivity of **26g** stems from its 1,3-dicabonyl functionality (*versus* keto-lactams (**26a–f**) and thus shows similar reactivity to compounds listed in Chart 2).

**Chart 4:**
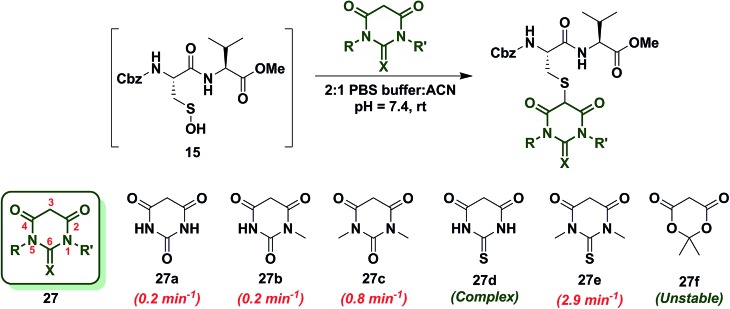
Reaction of sulfenic acid **15** with barbituric acid based nucleophiles.

Next, we studied the reactivity of cyclic C-nucleophiles containing two heteroatoms in the ring. The first such nucleophile screened was commercially available barbituric acid (**27a**). Barbituric acid (**27a**) is based on pyrimidine heterocycle skeleton and its p*K*_a_ is more than one unit lower than dimedone (**1**) (p*K*_a,barbituric acid_ = 4.01 *versus* p*K*_a,dimedone_ = 5.23).^53^ Although (**27a**) can exist as several different tautomers, the triketo form is generally considered to be most stable.^53^ Given its low p*K*_a_ and greater stabilization of the anion, we anticipated that (**27a**) would be less reactive than dimedone (**1**) or 1,3-cyclohexanedione (**22a**). Consistent with this hypothesis, the *k*_obs_ for the reaction of (**27a**) and sulfenic acid **15** was 0.2 min^–1^ (Chart 4). For subsequent studies, we prepared mono (**27b**) and dimethylated (**27c**) derivatives of **27a** according to literature procedures.^54^ 1-Methylbarbituric acid (**27b**) had similar reactivity to **27a** whereas 1,3-dimethylbarbituric acid (**27c**) displayed a slight rate enhancement (∼4-fold increase over **27a**). On the other hand, reaction with 2-thiobarbituric acid (**27d**) resulted in the formation of the expected adduct as well as side products, possibly due to the aromatization of **27d** and resulting reactive thiol nucleophile. Among all barbituric acid derivatives examined in our studies, 1,3-dimethyl-2-thiobarbituric acid (**27e**) gave the highest *k*_obs_ (2.9 min^–1^) (Chart 4). Meldrum's acid (**27f**), an oxygen-based heterocycle, was also evaluated for its reactivity. The expected adduct was observed, however, it rapidly decomposed owing to the aqueous instability of lactone **27f**. Due to the inherent instability of such lactones, analogous nucleophiles were not pursued further. In short, due to their electron-deficient heterocyclic ring, barbituric acid-based nucleophiles exhibit poor reactivity relative to dimedone (**1**). The slight increase in the reactivity of **27e** can be attributed to resonance destabilization of the C-3 carbanion.

### The effect of keto–enol tautomerism on cyclic C-nucleophile reactivity

To gain more insight into the effect of enolization on reactivity of cyclic C-nucleophiles, we selected cyclic 1,3-dicarbonyls with at least one carbonyl in conjugation with a phenyl ring, thus shifting the keto–enol tautomerism primarily towards enol form (**28a–e**, Chart S1†). Owing to the added stability imparted by aromatization or extended conjugation, compounds **28a–e** largely exist as **28a′–e′**. Minor adduct formation was observed for each compound; however, reactions were quite slow and did not proceed to completion. To further evaluate the effect of enolization on nucleophile reactivity, several enamines and hydrazide derivatives of dimedone (**1**) were prepared. With enamine derivatives (**29a–f**) either no reaction took place or *k*_obs_ was too slow to measure (Chart S2†). Likewise, hydrazide derivatives (**30a–d**) showed poor reactivity and rate constants were again too slow to measure accurately (Chart S2†). Together, these data underscore the detrimental effect of aromatic stabilization on cyclic C-nucleophile reactivity with sulfenic acid.

**Chart 5:**
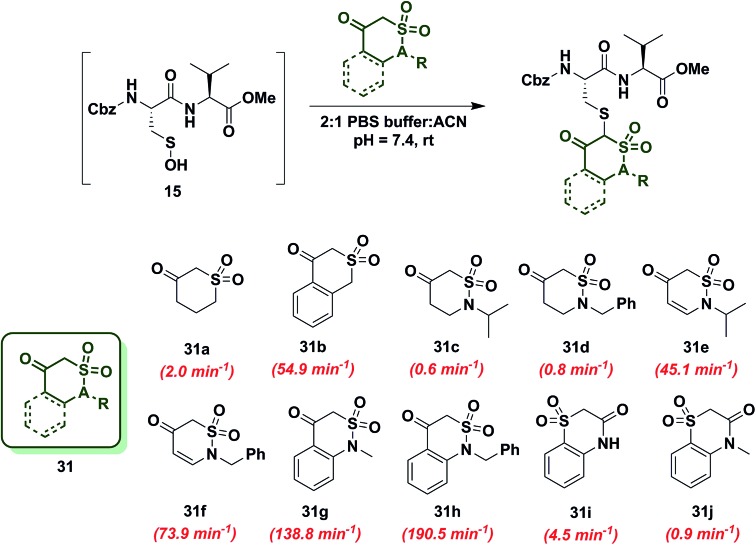
Reaction of sulfenic acid **15** with thiazine and benzo[*c*][1,2]thiazine-based nucleophiles.

Next, we explored the reactivity of cyclic C-nucleophiles with tautomeric equilibria shifted toward the keto form. To this end, we used the commercially available compound, dihydro-2*H*-thiopyran-3(4*H*)-one 1,1-dioxide (**31a**) in which one carbonyl is replaced with a sulfone. ^1^H-NMR of **31a** in DMSO-*d*_6_ clearly demonstrates that the remaining carbonyl exists predominantly as the keto form (Table S1,† Entry J). *k*_obs_ for reaction of **31a** and sulfenic acid **15** was 2.0 min^–1^, which represents a 2.5-fold increase relative to dimedone. This increase in reaction rate of **31a** is attributed to the enhanced reactivity of C-2 anion owing to the loss of resonance stability compared to 1,3-dicarbonyl compounds. Following up on this result, the phenyl-conjugated derivative of **31a**, isothiochroman-4-one 2,2-dioxide (**31b**) was prepared using a three-step literature reported procedure.^55^ Like **31a**, the keto form of **31b** predominates (Table S1,† Entry F) and showed a rate enhancement of almost 70-fold (*k*_obs_ = 54.9 min^–1^), when compared to dimedone (**1**). Another direct follow-up to **31a** is the class of compounds in which the sulfone is replaced with a sulfonamide moiety, as in 2-alkyl-1,2-thiazinan-5-one 1,1-dioxide (**31c**, **d**)^56^ and 2-alkyl-2*H*-1,2-thiazin-5(6*H*)-one 1,1-dioxide (**31e**, **f**)^57^ (prepared as described in Scheme S17†). Both 2-isopropyl-(**31c**) and 2-benzyl-(**31d**) 1,2-thiazinan-5-one 1,1-dioxides formed the expected adduct with sulfenic acid **15** with rate constants comparable to dimedone (**1**) (*k*_obs_ = 0.6 min^–1^ for **31c** and 0.8 min^–1^ for **31d**). However, **31e** and **31f** (*k*_obs_ = 45 min^–1^ and 73.9 min^–1^, respectively) were 50- and 90-fold more reactive than dimedone respectively (**1**) (Chart 5). It is worth noting that the only structural difference between **31c**, **d** and **31e**, **f** is the presence of a double bond, which is conjugated to the carbonyl. This difference leads to a substantial change in their reactivity towards sulfenic acid. Along these lines, we prepared benzo[*c*][1,2]thiazine-based analogs (**31g**, **h**) to evaluate the influence of benzene ring conjugation on sulfenic acid reactivity.^58^ Both **31g** and **31h** readily reacted with sulfenic acid **15** to form stable thioether adducts with relatively fast rate constants (*k*_obs_ = 138.8 min^–1^ for **31g** and 190.5 ± 12.7 min^–1^ for **31h**) or 200-fold greater, compared to dimedone (**1**) (Chart 5). The keto forms of **31g** and **31h** are greatly favored and very small signals from enol tautomers were observed by ^1^H-NMR (Table S1,† Entry D). In this regard, the crystal structure of **31g** indicates that the heterocyclic ring adopts a half-boat conformation with the sulfone S out of the plane, thus distorting the tetrahedral geometry around the S atom. Since formation of the enol tautomer of **31g** would require the ring to be planar, the non-planar heterocyclic ring forces the carbonyl to adopt the keto form.^59^ Consequently, the carbanion that forms under aqueous conditions is stabilized by resonance to lesser extent and is extremely reactive. Interestingly, replacement of the sulfonamide with an amide and the carbonyl with a sulfone (*i.e.*, converting benzo[*c*][1,2]thiazine analogs to benzo[*b*][1,4]thiazines) led to a significant reduction in reactivity (*k*_obs_ = 4.5 min^–1^ for **31i** and 0.9 min^–1^ for **31j**) (Chart 5). To summarize, thiazine analogs have shown generally enhanced reactivity compared to dimedone (**1**) which can be attributed to the effect of two factors – destabilization of carbanion due to reduced resonance (achieved by replacing one carbonyl with a sulfonamide) and further destabilization of carbanion due to sterics introduced by non-planar heterocyclic ring structure. The maximal cumulative effect of these factors is observed in benzo[*c*][1,2]thiazine analogs **31g**, **h**, which exhibited an increase of two orders of magnitude in reactivity compared to dimedone (**1**).

### Reactivity of 1,3-cyclopentanedione derivatives (5-membered ring system)

**Chart 6:**
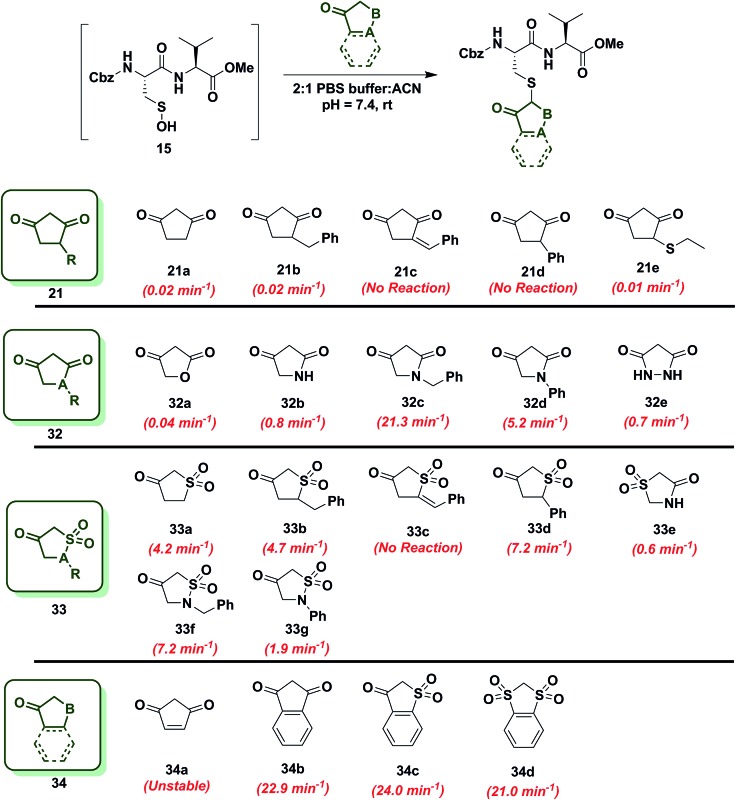
Reaction of sulfenic acid **15** with 5-membered cyclic nucleophiles.

To further increase the diversity of C-nucleophiles and verify the trends observed with 6-membered ring systems, we evaluated several 5-membered ring systems. As reported above, the lower p*K*_a_ and complete enolization of 1,3-cyclopentanedione (**21a**, *k*_obs_ = 0.02 min^–1^) manifested as a 40-fold decrease in the observed rate constant, compared to dimedone. Our observation is in line with protein-labeling data reported by Furdui and coworkers,^46^ however the effect is more pronounced in our model dipeptide sulfenic acid **15**. Next, we investigated the effect of C-4 alkylation on the reactivity of **21a**. 4-Benzylcyclopentane-1,3-dione (**21b**) did not show any rate enhancement and 4-benzylidenecyclopentane-1,3-dione (**21c**) exhibited a total loss of reactivity. Likewise, the C-4 aryl derivative, 4-phenylcyclopentane-1,3-dione (**21d**) proved unreactive. 4-(Ethylthio)cyclopentane-1,3-dione (**21e**) successfully reacted with sulfenic acid **15** (*k*_obs_ = 0.01 min^–1^), although with 2-fold decrease in reactivity relative to **21a**. This observation is in contrast to protein-labeling experiments reported by Furdui *et al.* ^46^ that show a two-fold rate enhancement with 4-ethylthio substitution, compared to **21a**. However, this apparent discordance with our data is readily explained by the presence of empty d-orbitals on the S atom that exerts a net electron-withdrawing effect on the 1,3-cyclopentanedione ring with concomitant stabilization of the C-2 carbanion and reduced reactivity with sulfenic acid. These contrasting data highlight the impact that protein microenvironment can have on probe reactivity and suggest that intrinsic nucleophile reactivity is best studied in small-molecule sulfenic acid model systems (Chart 6).

Since replacement of C-4 with an N-heteroatom yielded substantial rate enhancements in 6-membered C-nucleophile ring systems, we investigated similar heteroatom substitutions in 1,3-cyclopentanedione (**21a**). Replacing C-4 with an O-heteroatom gave the commercially available lactone, tetronic acid (**32a**), which formed the expected adduct with sulfenic acid **15** (*k*_obs_ = 0.04 min^–1^), albeit with only a two-fold increase in reactivity compared to **21a**. Next, we replaced C-4 with an N-heteroatom in the reaction of 2,4-pyrrolidinedione (**32b**) with sulfenic acid **15**. The *k*_obs_ value for this derivative was 0.8 min^–1^, which represents a 40-fold increase over **21a** and is also equivalent to dimedone (**1**). The *N*-alkylated nucleophile, 1-benzylpyrrolidine-2,4-dione (**32c**) exhibited a rate acceleration of more than 1000-fold (*k*_obs_ = 21.3 min^–1^) relative to **21a**, representing more than a 25-fold rate enhancement compared to dimedone (**1**). Similarly, the *N*-arylated C-nucleophile, 1-phenylpyrrolidine-2,4-dione (**32d**) was 250-fold more reactive (*k*_obs_ = 5.2 min^–1^) than **21a** and 5-fold more reactive than dimedone (**1**) (Chart 6). In this regard, we note that although ^1^H-NMR analysis of (**21a**) in DMSO-*d*_6_ indicates that this compound exists exclusively in the enol form, analogous spectra of **32c** and **32d** show a respective 10 : 3 and 1 : 1 ratio of keto to enol tautomeric forms, respectively (Table S1,† Entries K and L). These observations again suggest that shifting the tautomeric equilibrium to favor the keto form is a general mechanism to increase the reactivity of these C-nucleophiles toward sulfenic acid. Two substituting N-heteroatoms, as in 3,5-pyrazolidinedione (**32e**), accelerated reactivity 35-fold (*k*_obs_ = 0.7 min^–1^) when compared to **21a**, but remained similar in reaction rate constant to dimedone (**1**) (Chart 6).

In subsequent experiments, we tested the effect of replacing a carbonyl group with a sulfone moiety on 5-membered ring system C-nucleophiles. Dihydrothiophen-3(2*H*)-one 1,1-dioxide (**33a**) and 5-benzyldihydrothiophen-3(2*H*)-one 1,1-dioxide (**33b**) generated the expected thioether adduct with sulfenic acid **15** and both compounds exhibited more than a 200-fold enhancement in reactivity (*k*_obs_ = 4.2 min^–1^ and 4.7 min^–1^, respectively) relative to **21a**. However, the structurally related nucleophile, 5-benzylidenedihydrothiophen-3(2*H*)-one 1,1-dioxide (**33c**) failed to react with **15**. 5-Phenyldihydrothiophen-3(2*H*)-one 1,1-dioxide (**33d**) showed robust reactivity (*k*_obs_ = 7.2 min^–1^) translating into a rate enhancement of 350-fold in comparison to **21a** and a 10-fold increase relative to dimedone (**1**) (Chart 6). As a follow up to the above studies, we examined the reactivity of sulfonamide derivatives of **21a** toward sulfenic acid. Thiazolidin-4-one 1,1-dioxide (**33e**) reacted with **15** with *k*_obs_ = 0.6 min^–1^. Alkylated or arylated 5-membered ring systems, as in isothiazolidin-4-one 1,1-dioxide, 2-benzylisothiazolidin-4-one 1,1-dioxide (**33f**) or 2-phenylisothiazolidin-4-one 1,1-dioxide (**33g**) exhibited *k*_obs_ of 7.2 min^–1^ and 1.9 min^–1^, respectively (Chart 6).

Finally, we tested the reactivity of 5-membered C-nucleophile ring systems containing an internal double bond. When the commercially available 4-cyclopentene-1,3-dione (**34a**) was reacted with sulfenic acid **15**, minor adduct formation was observed. However, due to **34a** being a diene as well as a dienophile (substituted alkene) it readily undergoes [4 + 2] cycloaddition, the major species was identified as the self-condensation product. 1,3-Indandione (**34b**) and related sulfone derivatives, benzo[*b*]thiophen-3(2*H*)-one 1,1-dioxide (**34c**) and 2*H*-benzo[*d*][1,3]dithiole 1,1,3,3-tetraoxide (**34d**) showed an approximate increase in rate constant of 30-fold in comparison to dimedone (**1**) (*k*_obs_ = 22.9 ± 0.8 min^–1^, 24.0 min^–1^ and 21.0 min^–1^, respectively, Chart 6). In general, 5-membered C-nucleophiles display reactivity trends similar to those observed for 6-membered ring systems. However, the comparative reaction rates are generally lower, owing to the enhanced carbanion stability of the planar heterocycle structure. A notable exception is 1,3-indanedione **34b**, which was substantially more reactive, compared to its 6-membered counterpart **28a** (which was stabilized due to resonance). ^1^H-NMR in DMSO showed that, 1,3-indandione **34b** existed exclusively in keto form, unlike naphthalene-1,3-diol **28a** (Table S1,† Entry E). These data, along with a predicted p*K*_a_ of 8.9, resulting in the formation of a sufficiently reactive carbanion at physiological pH, can account for the elevated reactivity of **34b**.

### Evaluating C-nucleophile selectivity and thioether bond stability

Increased C-nucleophile reactivity may lead to decreased selectivity for the sulfenic acid target. Consequently, we thought it prudent to screen representative C-nucleophiles (**1**, **26a**, **31f**, **31h**, **34b**) for cross-reactivity with other biological functional groups (Scheme S19†). For these studies, we utilized Fmoc (or Cbz) – protected amino acids cysteine (thiol), serine (alcohol), lysine (amine), cystine (disulfide) as well as sulfinic acid (BnSO_2_Na) in aqueous buffer at pH 7.4. The resulting data demonstrate that the majority of nucleophiles retained their selectivity for sulfenic acid. One exception to these findings was 2-benzyl-1,2-thiazinan-5-one 1,1-dioxide (**bTD**, **31f**), which gave the expected Michael adduct with Fmoc-Lys-OH (Scheme S19, Fig. S13†). Next, we evaluated the stability of the thioether bond formed between C-nucleophiles **1**, **26a** and **31h** and **15** under reducing conditions, such as that encountered within the cytosol. For these studies, the dipeptide–nucleophile product from each reaction was purified and analyzed by NMR to establish that the correct thioether bond was formed (Scheme S20†). Incubation of each product with millimolar concentration of dithiothreitol (DTT), glutathione (GSH) or tris(2-carboxyethyl) phosphine (TCEP) indicated that each adduct was stable for more than 12 h (Scheme S21†). These cross-reactivity and stability studies affirm the selectivity of the C-nucleophiles for sulfenic acid and the irreversible nature of thioether adduct thus formed.

### Screening C-nucleophiles in a protein sulfenic acid model

**Fig. 2:**
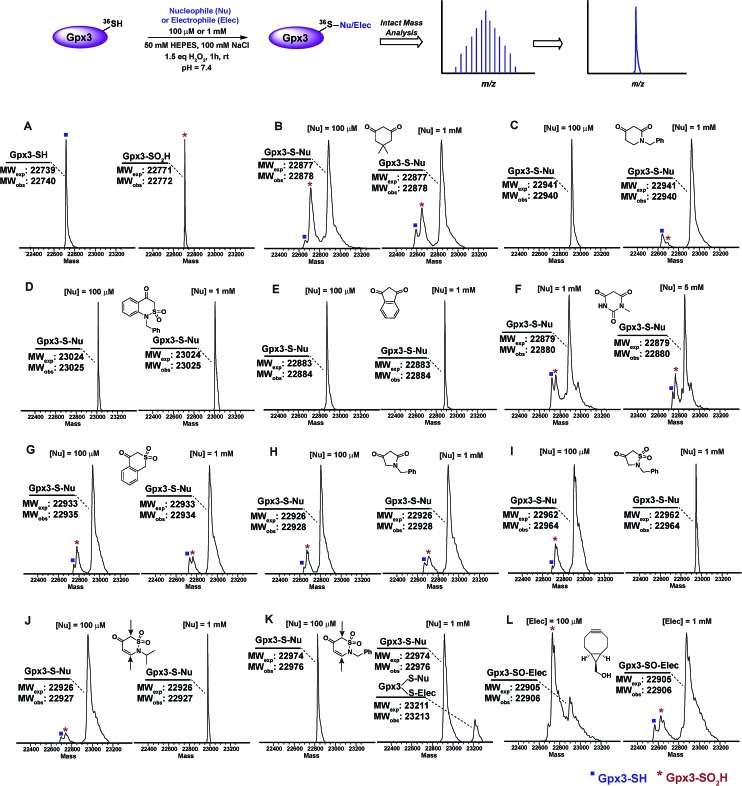
Labeling of Gpx3-SOH with various nucleophiles under oxidizing conditions. Gpx3-SH (10 μM) was incubated with various nucleophiles at 100 μM or 1 mM concentration under oxidizing (1.5 eq. H_2_O_2_) conditions for 1 h and analyzed by LTQ-MS. (A) Reduced and oxidized Gpx3; (B) dimedone (**1**); (C) 1-benzylpiperidine-2,4-dione **26f**; (D) 1-benzyl-1*H*-benzo[*c*][1,2]thiazin-4(3*H*)-one 2,2-dioxide **31h**; (E) 1,3-indandione **34b**; (F) *N*-methylbarbituric acid **27b**; (G) isothiochroman-4-one 2,2-dioxide **31b**; (H) 1-benzylpyrrolidine-2,4-dione **32c**; (I) 2-benzylisothiazolidin-4-one 1,1-dioxide **33f**; (J) 2-benzyl-2*H*-1,2-thiazin-5(6*H*)-one 1,1-dioxide **31f**; (K) 2-isopropyl-2*H*-1,2-thiazin-5(6*H*)-one 1,1-dioxide **31e**; (L) ((1*R*,8*S*,9*s*)-bicyclo[6.1.0]non-4-yn-9-yl)methanol **5**.

Next, we examined the reactivity/selectivity of C-nucleophiles that exhibited enhanced *k*_obs_ (relative to dimedone) with dipeptide sulfenic acid **15**. For these studies, we utilized a Cys64Ser Cys82Ser variant of the thiol peroxidase, Gpx3 which we have previously established as a facile model for a protein sulfenic acid.^7,11,13,60–62^ Control experiments demonstrated that incubation of Gpx3 with dimedone (**1**) under reducing conditions did not result in protein-adduct formation, as expected since C-nucleophiles do not react with the thiol functional group (Fig. S34A†). Nearly quantitative oxidation of catalytic Gpx3 Cys36-SH was achieved using 1.5 equivalents of H_2_O_2_ (Fig. 2A, also see Fig. S33B†). Incubation of Gpx3 Cys36-SOH with dimedone (1 mM) afforded the expected thioether adduct (22 878 Da), as verified by intact ESI-LC/MS analysis (Fig. 2B, Panel 2 and Fig. S34C†). Of note, labeling with dimedone (**1**) was not quantitative, as we also observed unreacted Gpx3-SH (22 740 Da) and Gpx3-SO_2_H (22 772 Da). Next, we selected one C-nucleophile from each structural class and evaluated their reactivity towards Gpx3 under oxidizing or reducing states. With Gpx3 Cys36-SOH, 1-benzylpiperidine-2,4-dione (**26f**), 1-benzyl-1*H*-benzo[*c*][1,2]thiazin-4(3*H*)-one 2,2-dioxide (**31h**), 1,3-indandione (**34b**), *N*-methylbarbituric acid (**27b**), isothiochroman-4-one 2,2-dioxide (**31b**), 1-benzylpyrrolidine-2,4-dione (**32c**) and 2-benzylisothiazolidin-4-one 1,1-dioxide (**33f**) all showed nearly quantitative adduct formation (Fig. 2C–I). With Cys36 Cys-SH, no reaction occurred between the aforementioned C-nucleophiles and Gpx3 (Fig. S35A–S41A†), further validating their selectivity for sulfenic acid.

2-Isopropyl-2*H*-1,2-thiazin-5(6*H*)-one 1,1-dioxide (**31e**) and 2-benzyl-2*H*-1,2-thiazin-5(6*H*)-one 1,1-dioxide (**31f**) each contain an α,β-unsaturated carbonyl system with the potential to react with thiols or amines *via* a Michael-type addition. Incubation of Gpx3 Cys36-SOH with **31f** indicated the formation of two adducts (Fig. 2K, Panel 2). Of these modifications, one corresponded to the expected Cys36-thioether adduct, while the second was ostensibly formed *via* Michael addition with a Lys residue. When **31f** was used at 10-fold lower concentration (100 μM), the side-reaction with Lys was mitigated (Fig. 2K, Panel 1). By contrast, incubation of **31e** with Gpx3 Cys36-SOH gave only the expected thioether adduct, suggesting that the isopropyl group may sterically hinder Michael addition (Fig. 2J). Irrespective, the use of **31e**, **f** chemotypes as probes for protein sulfenic acid detection is not recommended owing to their potential cross-reactivity with Cys and Lys residues.

For the sake of inclusivity, we examined the reactivity of a recently reported electrophilic probe,^32^ BCN (**5**) for reactivity with oxidized and reduced Gpx3. Interestingly, when present at 1 mM, **5** formed a covalent adduct with Gpx3 Cys36-SH (Fig. S44A†), which indicates cross-reactivity with protein thiols. Under oxidizing conditions, **5** reacted with Cys36-SOH to give the expected sulfoxide adduct (Fig. 2L and Fig. S44C†). Of note, adduct formation was sub-stoichiometric at 100 μM of **5**, (Fig. 2L, Panel 1 and Fig. S44B†) but was the major product when the concentration of **5** was increased 10-fold (1 mM) (Fig. 2L, Panel 2 and Fig. S44C†). These data, particularly the cross reactivity with reduced Gpx3 Cys36-SH suggest limited applicability of BCN (**5**)^32^ as a *selective* probe for detecting protein sulfenic acids.^34–36^

**Fig. 3:**
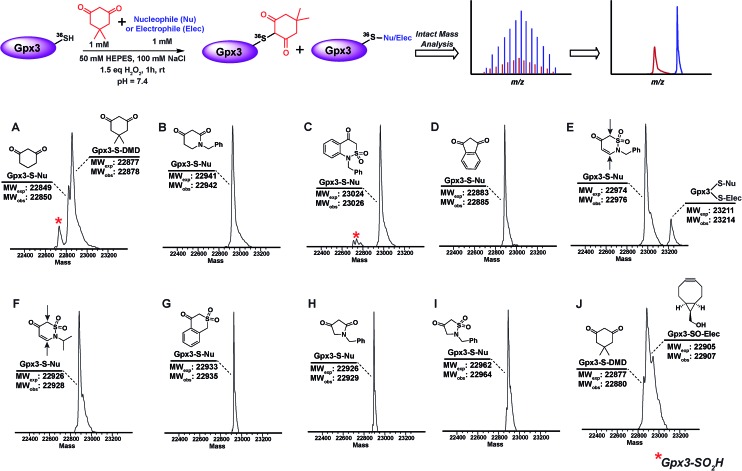
Competitive labeling of Gpx3-SOH with various nucleophiles in presence of 1 mM dimedone–Gpx3-SH (10 μM) was incubated with various nucleophiles (1 mM concentration) in presence of dimedone (**1**) (1 mM) under oxidizing (1.5 eq. H_2_O_2_) conditions. Each sample was analyzed by LTQ-MS for competitive labeling. (A) 1,3-Cyclohexanedione **22a**; (B) 1-benzylpiperidine-2,4-dione **26f**; (C) 1-benzyl-1*H*-benzo[*c*][1,2]thiazin-4(3*H*)-one 2,2-dioxide **31h**; (D) 1,3-indandione **34b**; (E) 2-benzyl-2*H*-1,2-thiazin-5(6*H*)-one 1,1-dioxide **31f**; (F) 2-isopropyl-2*H*-1,2-thiazin-5(6*H*)-one 1,1-dioxide **31e**; (G) isothiochroman-4-one 2,2-dioxide **31b**; (H) 1-benzylpyrrolidine-2,4-dione **32c**; (I) 2-benzylisothiazolidin-4-one 1,1-dioxide **33f**; (J) ((1*R*,8*S*,9*s*)-bicyclo[6.1.0]non-4-yn-9-yl)methanol **5**.

The abovementioned panel of cyclic C-nucleophiles (**22a**, **26f**, **31b**, **31e**, **f**, **31h**, **32c**, **33f**, and **34b**) was also tested for their ability to covalently label Gpx3 Cys36-SOH in the presence of an equal concentration of dimedone (**1**). All nucleophiles, except **22a** entirely outcompeted Gpx3 labeling by dimedone (**1**) (Fig. 3A–I and Scheme S23, Fig. S48–S56†), a gratifying conclusion that is fully consistent with the kinetic rate studies detailed above. In contrast, adducts corresponding to Gpx3-*S*-dimedone and Gpx3-*S*-BCN were observed with the electrophilic probe, BCN (**5**) (Fig. 3J, Fig. S57†), which is also consistent with the kinetic data obtained in the dipeptide sulfenic acid **15** model system.

## Discussion

Although numerous studies profiling electrophiles as reactivity probes for thiols have been reported,^27,63–66^ to our knowledge, this study represents the first of its kind to comprehensively profile nucleophiles as reactivity probes for the related sulfur oxoform, sulfenic acid. Herein, we have conceived, synthesized and screened several classes of cyclic C-nucleophiles for their reactivity with a novel model dipeptide sulfenic acid using a newly developed, facile LC-MS assay. The observed rate constants obtained from the fits to the ensuing data enables the stratification of C-nucleophiles based on their reaction kinetics. Our approach is user-friendly and utilizes a simply prepared dipeptide that can be stored in stable form until it is needed for conversion to sulfenic acid under aqueous conditions. Thus, this work addresses a fundamental, previously unmet need for a workflow that expedites the identification of compounds, which react with cysteine sulfenic acid over a broad range of time scales (10 to 2×10^5^ M^–1^ min^–1^).

A major goal of this study was to identify new classes of cyclic C-nucleophiles with robust reaction kinetics for future development as cellular probes of protein sulfenic acid. To this end, in the present work, we have identified several classes of cyclic C-nucleophiles with 100- to 200-fold enhanced rate of reaction compared to dimedone (**1**). Screening nucleophiles based on ring size showed that reactivity increases with the shift from the enol to keto forms, indicating that factors resulting in the destabilization of carbanion at C-2 positively influence reactivity. The destabilization and reactivity of the C-2 carbanion was found to depend upon three primary factors: (i) electronic effects, as EDG substitution of the ring system enhances C-2 reactivity and *vice versa*; (ii) loss of resonance stability, and (iii) steric factors, which influence the ring to achieve non-planar forms. In Chart 2, we observe the effect of EDG or EWG substitution, which cause a respective increase or decrease in reactivity of cyclic C-nucleophiles towards sulfenic acid. Nucleophiles based on the 2,4-piperidinedione (**26a**) scaffold had one of the carbonyls replaced with a lactam, resulting in loss of resonance stabilization and a substantially more reactive carbanion (Chart 3). In general, barbituric acid derivatives were electron-deficient heterocycles and resonance stabilized, which lead to reduced reactivity towards sulfenic acid (Chart 4). The greater reactivity of thiazine nucleophiles stems from the decrease in resonance stabilization and steric factors introduced by the sulfonamide substitution (Chart 5). The maximal additive effect of these two factors was observed for benzo[*c*][1,2]thiazine analogs **31g**, **h**, which were ∼200-fold more reactive towards sulfenic acid compared to dimedone (**1**). Lastly, 5-membered cyclic nucleophiles followed same reactivity trends, but showed reduced reactivity relative to 6-membered counterparts (Chart 6). The observed enhancement in reactivity was further verified by obtaining 2^nd^ order rate constants for representative reactive cyclic C-nucleophiles (Chart S3†). For example, the 2^nd^ order rate constant for benzyl-PRD (**26f**, *k*_obs_ = 1192 M^–1^ s^–1^) showed a 100-fold increase compared to dimedone (**1**, *k*_obs_ = 11.8 M^–1^ s^–1^). Likewise, the rate enhancement calculated from the 2^nd^ order rate constants of benzyl-BTD (**31h**, *k*_obs_ = 1725 M^–1^ s^–1^) and 1,3-indandione (**34b**, *k*_obs_ = 251 M^–1^ s^–1^) agreed well with the reactivity increase obtained from earlier pseudo 1^st^ order rate constant values (Chart S3†).

**Fig. 4:**
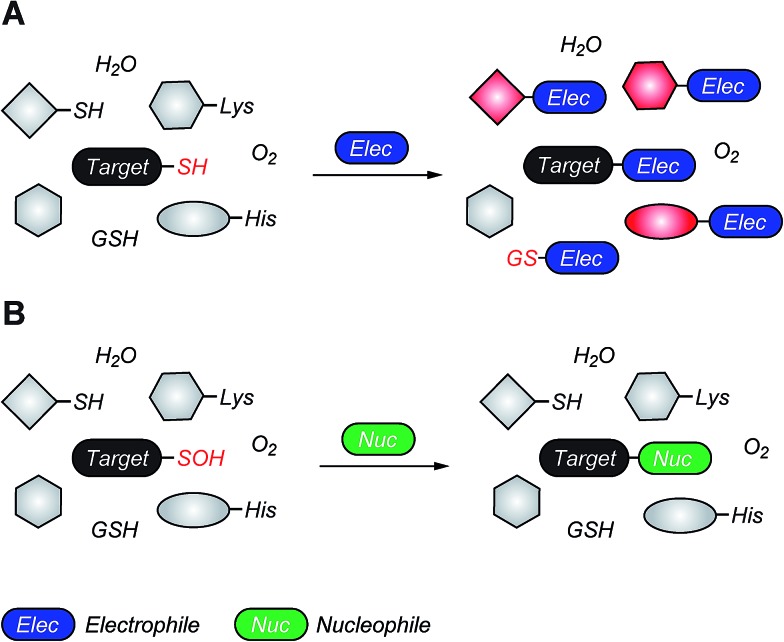
Covalent cysteine-based inhibition strategies. (A) Electrophilic covalent inhibitors inactivate their target through covalent attachment to the cysteine thiol functional group. However, the electrophilic center (*e.g.*, acrylamide, haloacetamide, and vinyl sulfonamide) can also react with other cellular nucleophiles such as glutathione as well as the amino and imidazole groups of amino acids. (B) Nucleophilic covalent strategy as an alternative or complementary inhibition mechanism. According to this approach, active site-directed small-molecule inhibitors containing a reactive nucleophilic center form a covalent bond with a cysteine side chain that has oxidized to sulfenic acid. Such modifications form transiently in specific proteins during H_2_O_2_-mediated signal transduction in normal cells, but form constitutively in diseases associated with chronically elevated levels of H_2_O_2_, including cancer. In the sulfenic acid oxidation state, the electron deficient sulfur exhibits enhanced electrophilic character that can be selectively targeted by certain nucleophilic compounds. Because sulfenic acid is a unique chemical moiety in biochemistry, this strategy could decrease the potential for off-target activity while retaining the advantages gained by covalent targeting.

**Fig. 5:**
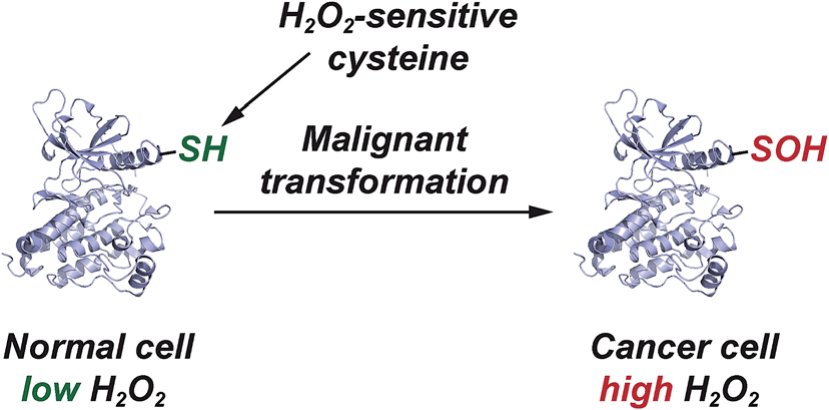
Elevated EGFR and HER2 levels in cancer cells correlate with a significant increase in protein sulfenylation.

Finally, with the re-emergence of covalent inhibition strategies,^67–71^ one possible use of our cyclic C-nucleophile library is toward the development of inhibitors that target oxidized cysteine residues in therapeutically important proteins, such as kinases. With the FDA approval of afatinib^72^ and ibrutinib,^73^ Cys-targeting covalent inhibitors of the ErbB family of tyrosine kinases and Bruton tyrosine kinase (BTK) respectively, inhibition of receptor tyrosine kinase signaling has emerged as one of the more effective anticancer treatment strategy. Recent findings indicate that elevated EGFR and HER2 (ErbB family) levels in cancer cells correlate with an increase in H_2_O_2_ levels and global protein sulfenylation.^74,75^ Moreover, we have previously reported that Cys_797_ of EGFR undergoes sulfenic acid modification.^7^ Because of its electrophilic nature, EGFR-Cys_797_-SOH precludes the covalent bond formation with electrophilic inhibitors like afatinib, resulting in significant loss of overall effectiveness. However, it also presents a unique opportunity to utilize the nucleophiles library as warheads to target electrophilic EGFR-Cys_797_-SOH (Fig. 4). With nine other protein tyrosine kinases including BTK^76^ (Cys_481_) harboring a Cys residue that is structurally homologous to EGFR-Cys_797_,^68^ this group of kinases may be regulated by oxidation of this key residue and susceptible to irreversible inhibition by nucleophilic redox-based inhibitors (Fig. 5). These studies are currently underway in our laboratory and will be reported in due course.

## Conclusions

We have reported a facile mass spectrometry-based assay and repurposed dipeptide-based model to screen a library of cyclic C-nucleophiles for reactivity with sulfenic acid under aqueous conditions. Observed rate constants for ∼100 cyclic C-nucleophiles were obtained and, from this collection, we have identified novel compounds with more than 200-fold enhanced reactivity, as compared to dimedone (**1**). The increase in reactivity and retention of selectivity of these C-nucleophiles were validated in secondary assays, including a protein model for sulfenic acid. Together, this work represents a significant step toward developing new chemical reporters for detecting protein *S*-sulfenylation with superior kinetic resolution. The enhanced rates and varied composition of the C-nucleophiles should enable more comprehensive analyses of the sulfenome and serve as the foundation for reversible or irreversible nucleophilic covalent inhibitors that target oxidized cysteine residues in therapeutically important proteins.
