# Supplementary material for: Profiling the reactivity of cyclic C-nucleophiles towards electrophilic sulfur in cysteine sulfenic acid
Source: Chem Sci. 2015 Oct 7;7(1):400–15. doi: 10.1039/c5sc02569a (PMC4724439; doi:10.1039/c5sc02569a)
Supplement: Supplementary file 1 [file SC-007-C5SC02569A-s001.pdf]

Electronic Supplementary Material (ESI) for Chemical Science.
This journal is © The Royal Society of Chemistry 2015

Profiling the Reactivity of Cyclic C-Nucleophiles towards Electrophilic Sulfur in Cysteine Sulfenic Acid

Vinayak Gupta and Kate S. Carroll*

Department of Chemistry, The Scripps Research Institute, Jupiter, Florida, 33458

^{1}H-NMR and ^{13}C-NMR Data

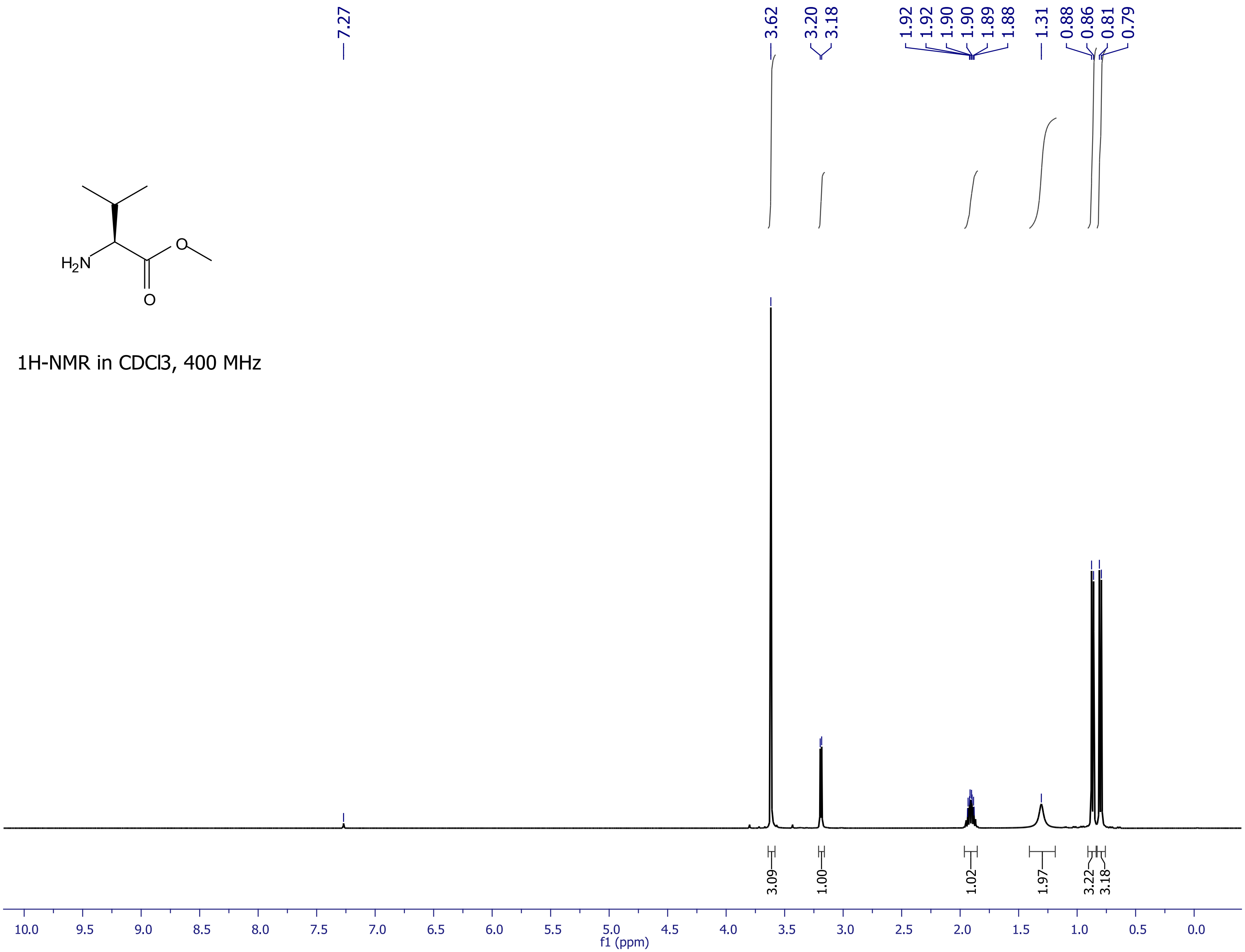

1H-NMR in CDCl3, 400 MHz

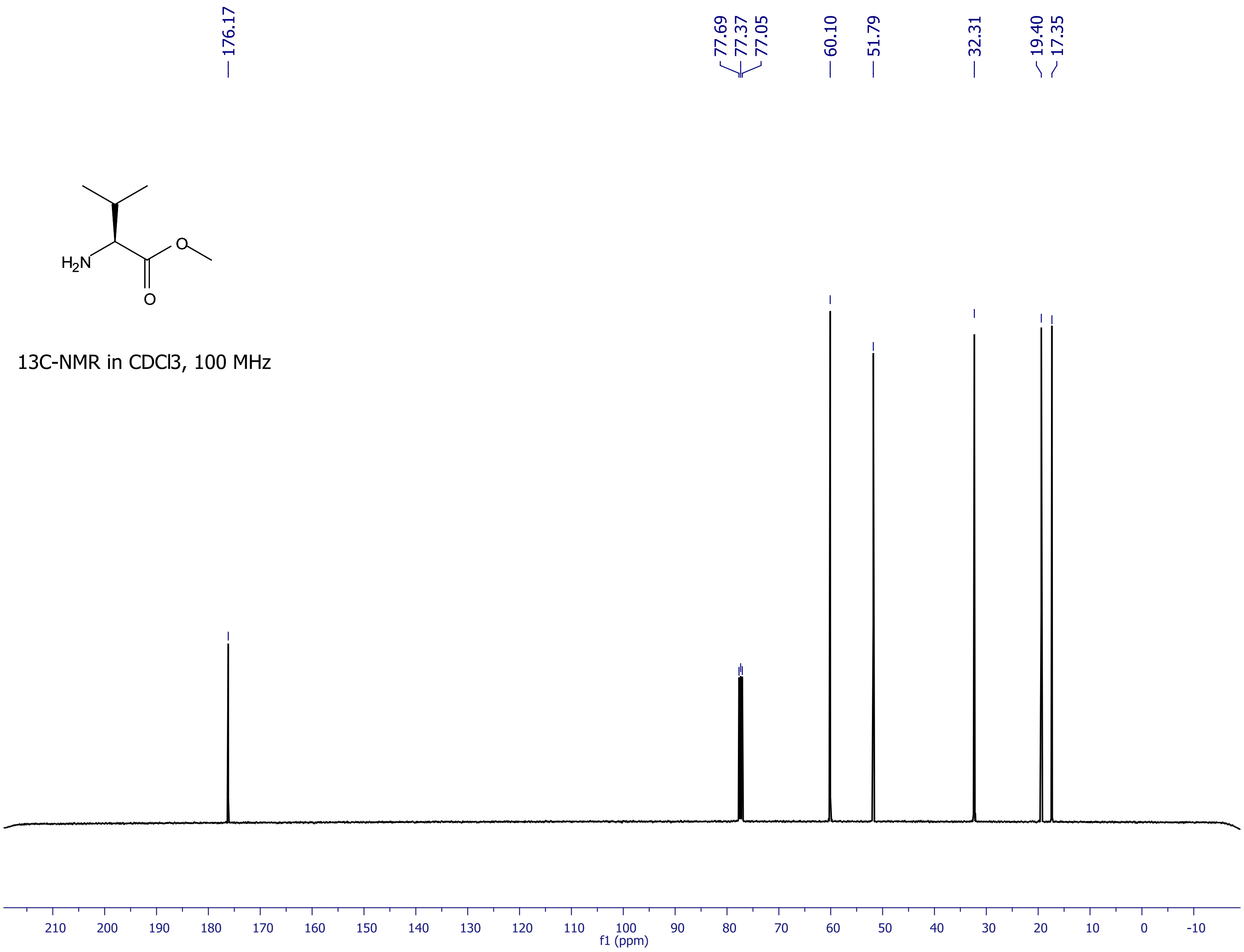
176.17
77.69
77.37
77.05
60.10
51.79
32.31
19.40
17.35
H2N
O
O
13C-NMR in CDCl3, 100 MHz
210 200 190 180 170 160 150 140 130 120 110 100 90 80 70 60 50 40 30 20 10 0 -10
f1 (ppm)

(Cbz-Cys-OH)2
1H-NMR in DMSO-d6, 400 MHz

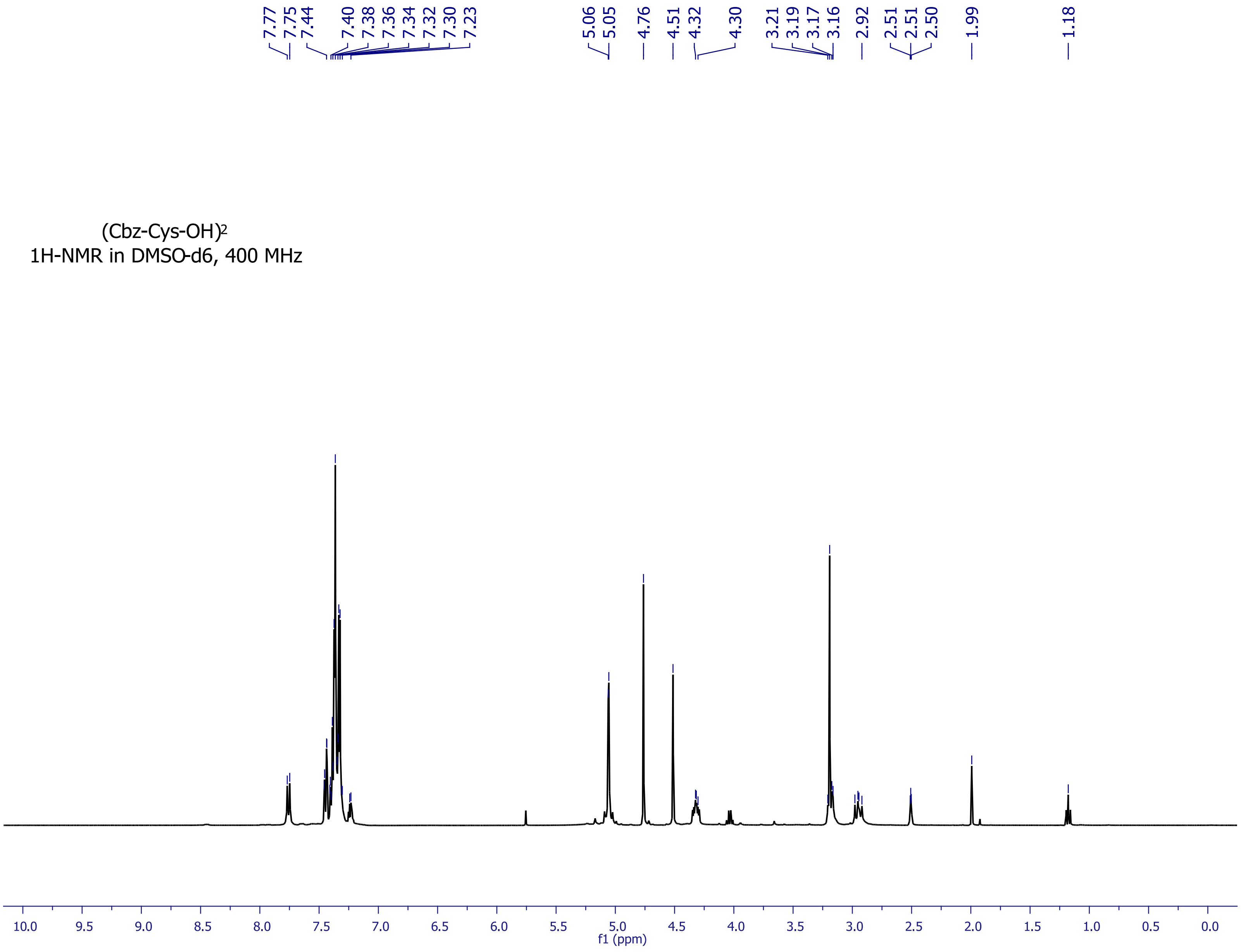

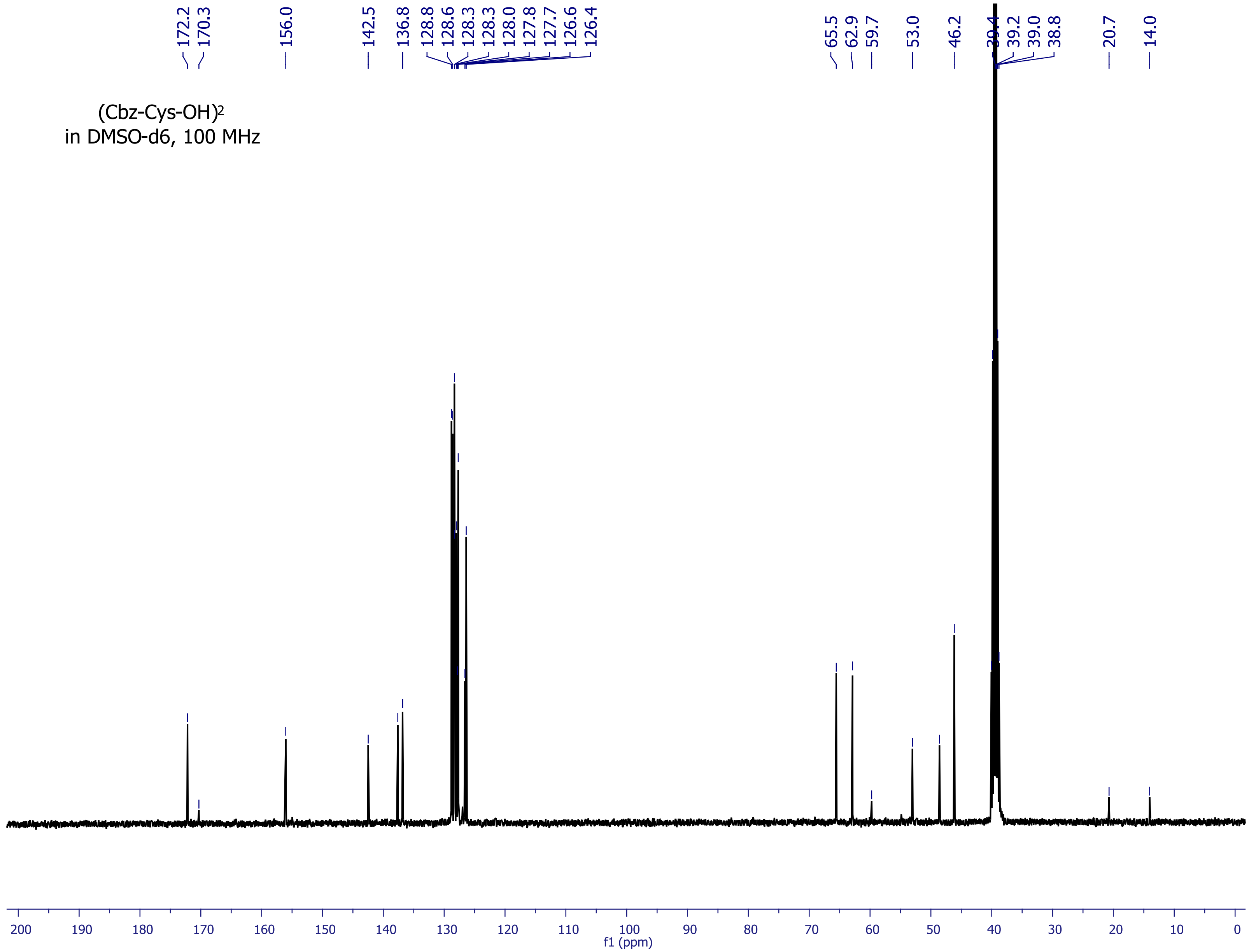
(Cbz-Cys-OH)2
in DMSO-d6, 100 MHz
172.2
170.3
156.0
142.5
136.8
128.8
128.6
128.3
128.3
128.0
127.8
127.7
126.6
126.4
65.5
62.9
59.7
53.0
46.2
39.2
39.0
38.8
20.7
14.0
200 190 180 170 160 150 140 130 120 110 100 90 80 70 60 50 40 30 20 10 0
f1 (ppm)

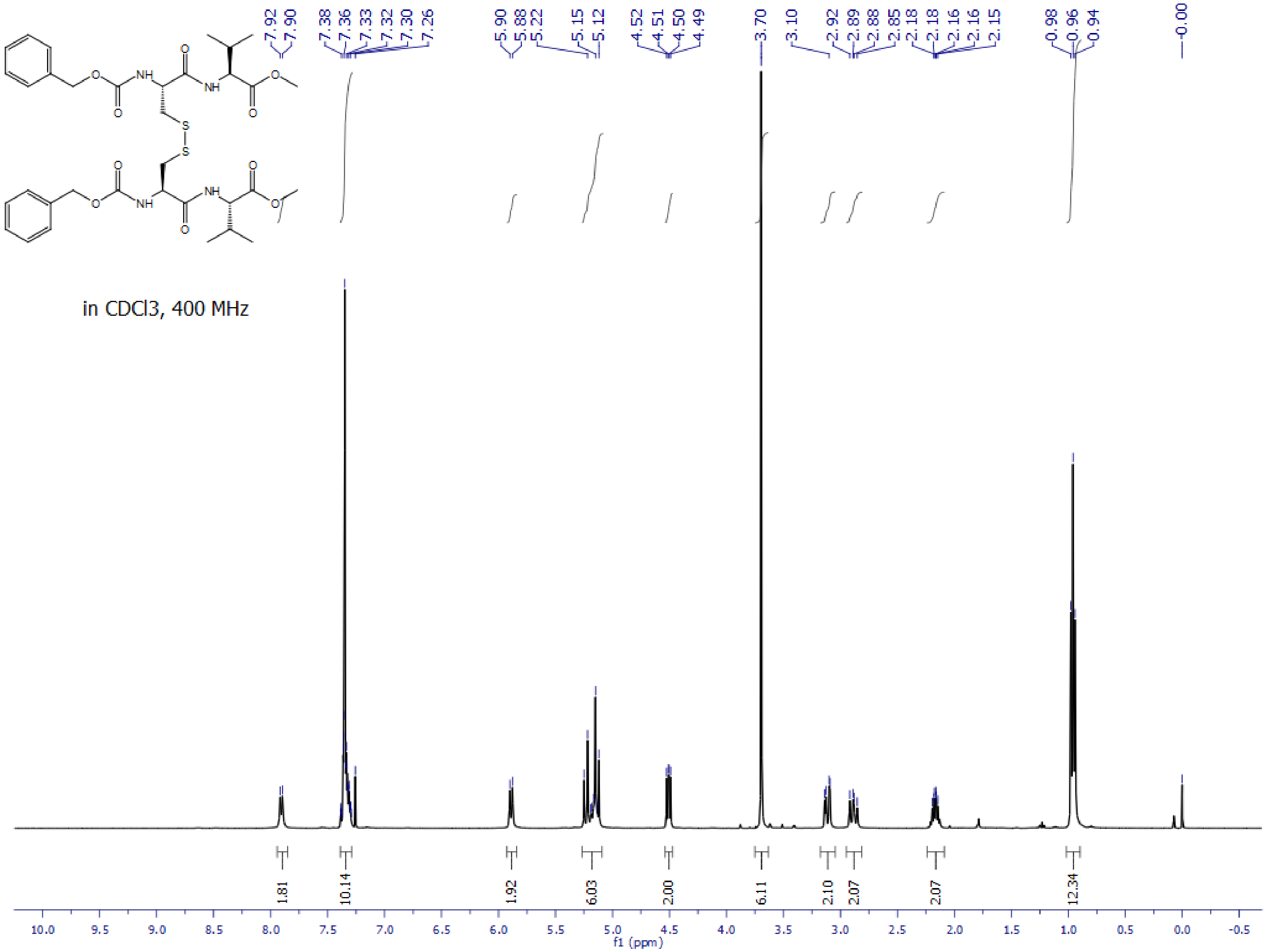
in CDCl3, 400 MHz
f1 (ppm)

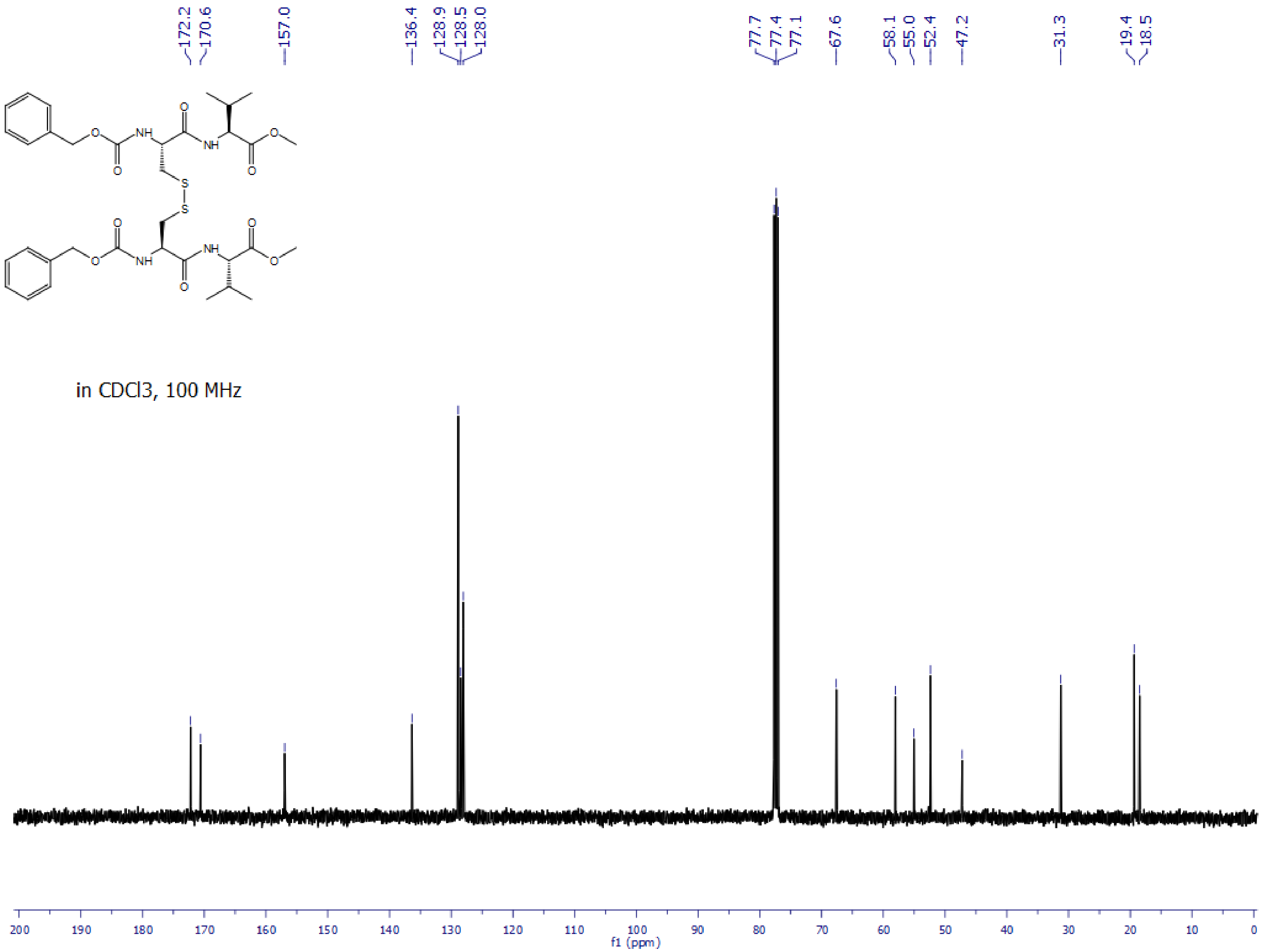

in CDCl3, 100 MHz

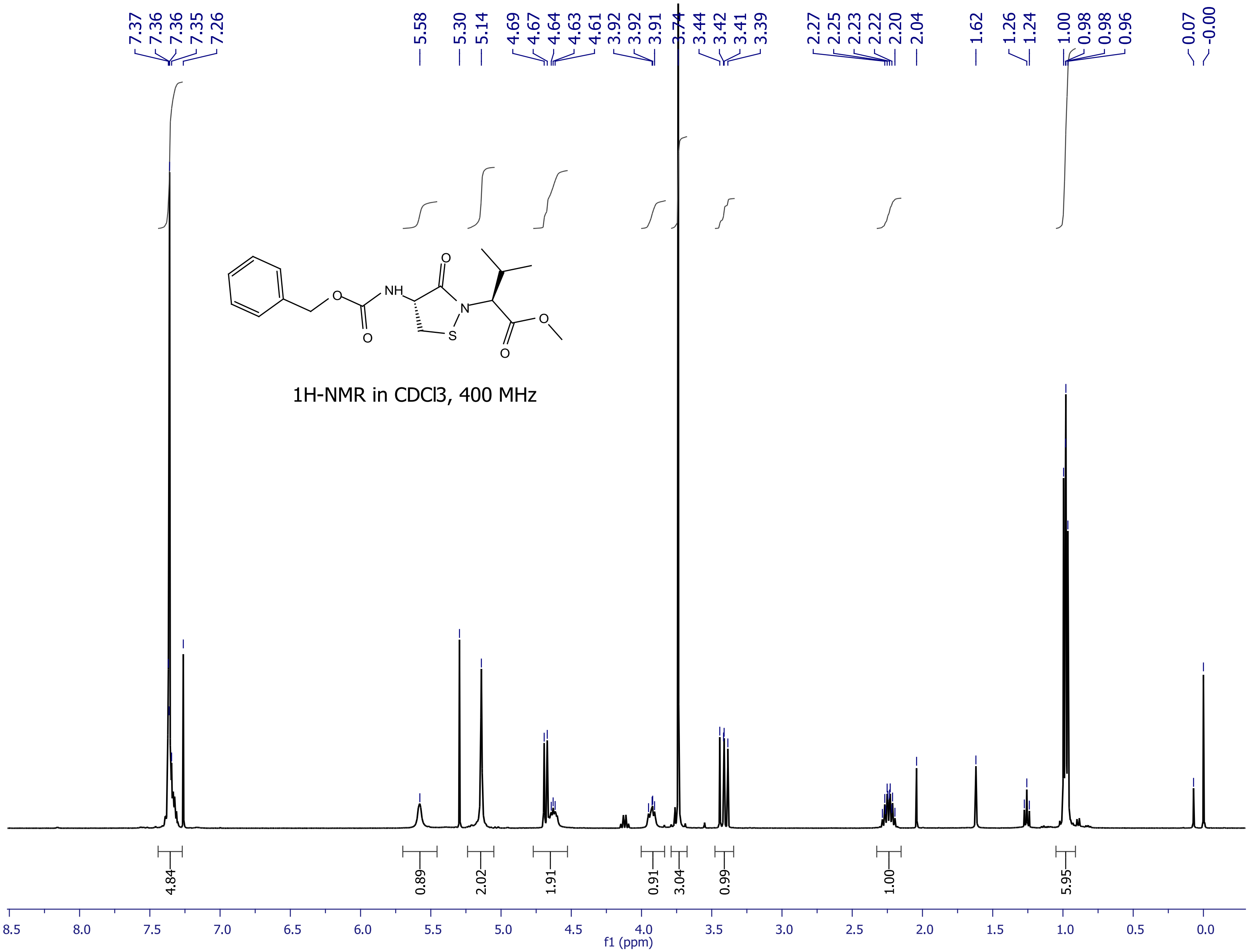
1H-NMR in CDCl3, 400 MHz
7.37 7.36 7.36 7.35 7.26 5.58 5.30 5.14 4.69 4.67 4.64 4.63 4.61 3.92 3.92 3.91 3.74 3.44 3.42 3.41 3.39 2.27 2.25 2.23 2.22 2.20 2.04 1.62 1.26 1.24 1.00 0.98 0.98 0.96 0.07 -0.00
4.84 0.89 2.02 1.91 0.91 3.04 0.99 1.00 5.95
f1 (ppm)

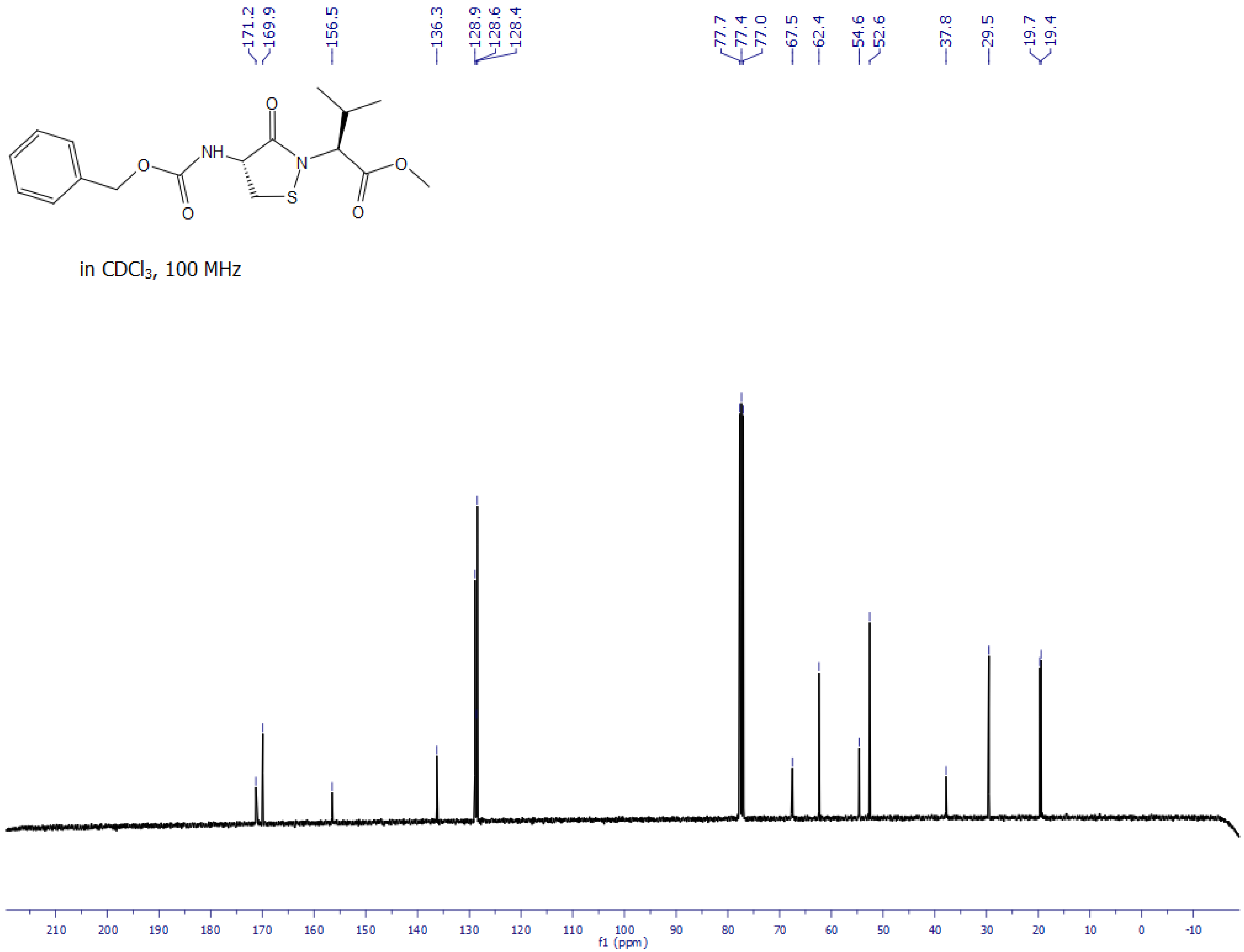

in $CDCl_3$, 100 MHz

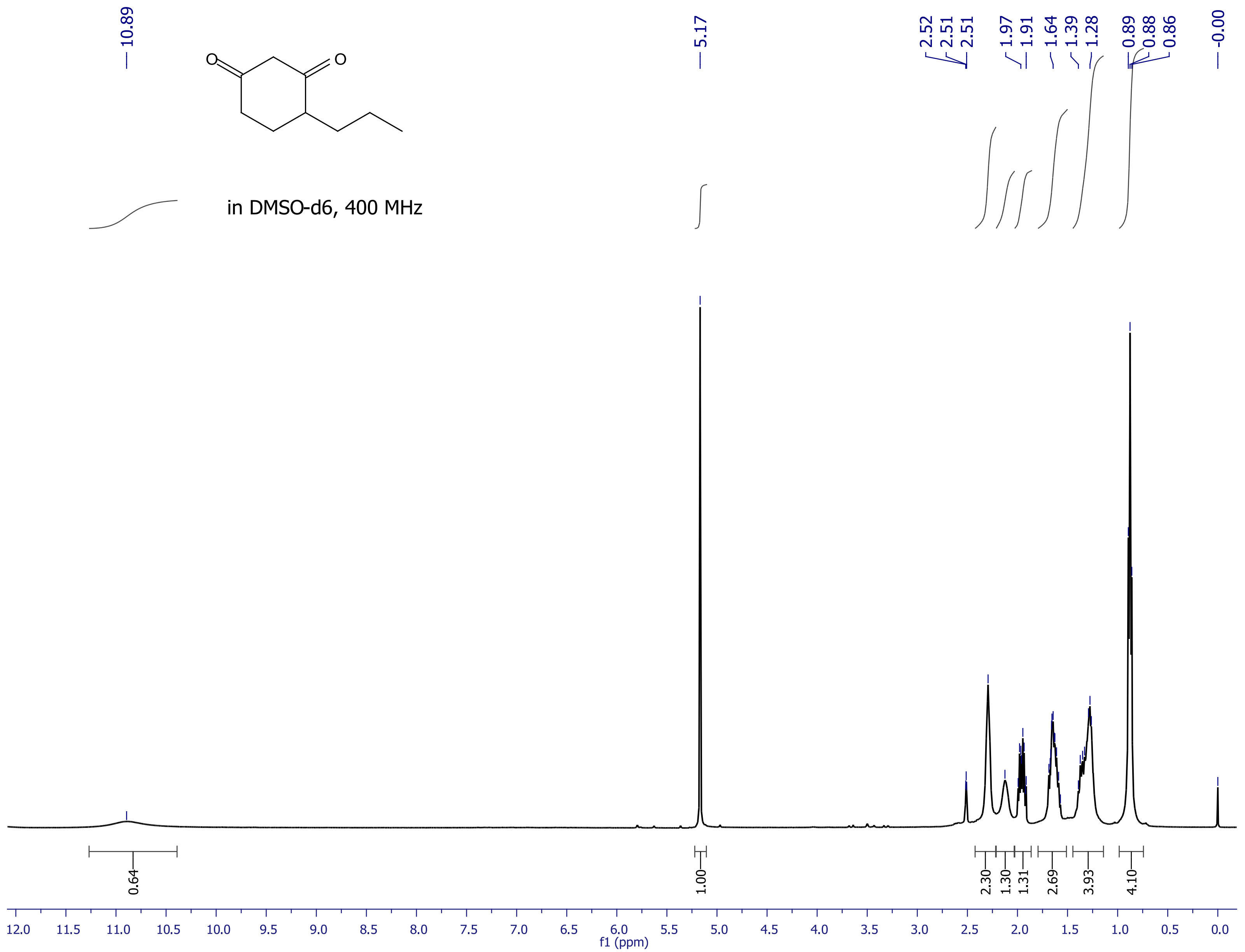
in DMSO-d6, 400 MHz
10.89
5.17
2.52
2.51
2.51
1.97
1.91
1.64
1.39
1.28
0.89
0.88
0.86
-0.00
0.64
1.00
2.30
1.30
1.31
2.69
3.93
4.10
f1 (ppm)
12.0 11.5 11.0 10.5 10.0 9.5 9.0 8.5 8.0 7.5 7.0 6.5 6.0 5.5 5.0 4.5 4.0 3.5 3.0 2.5 2.0 1.5 1.0 0.5 0.0

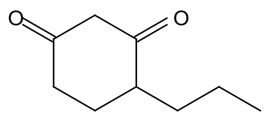

in DMSO-d6, 100 MHz

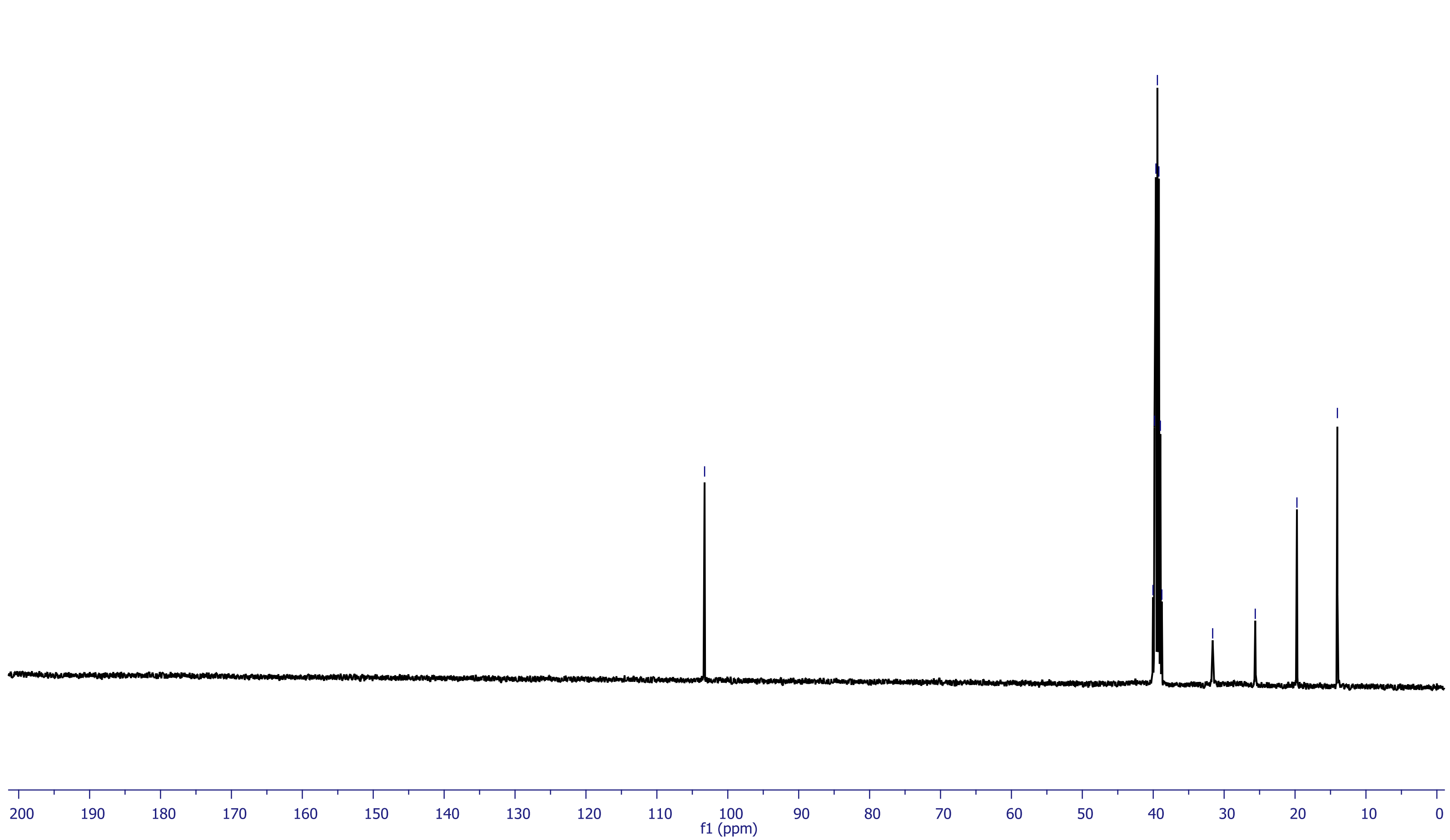

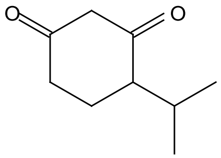

in DMSO-d6, 400 MHz

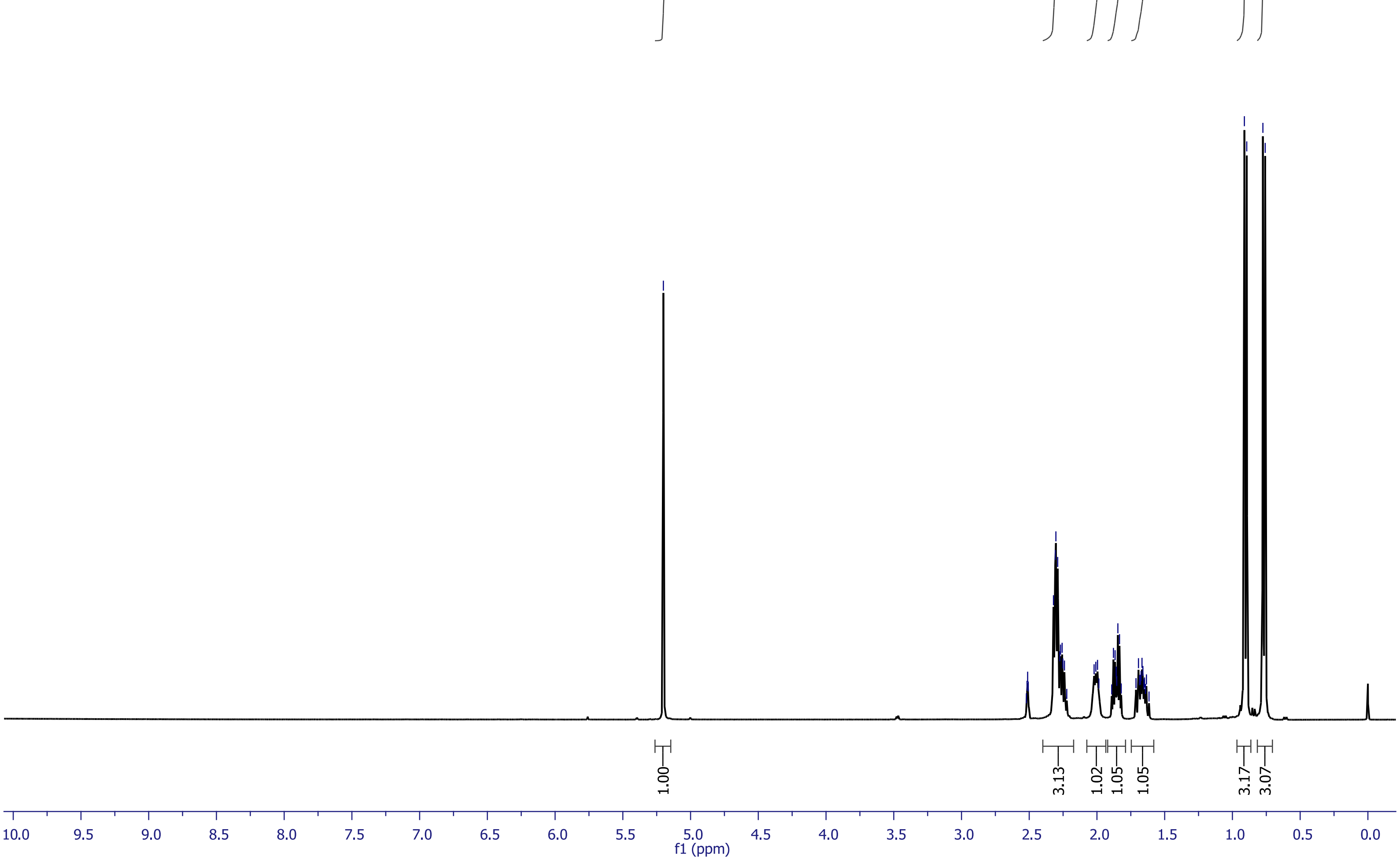

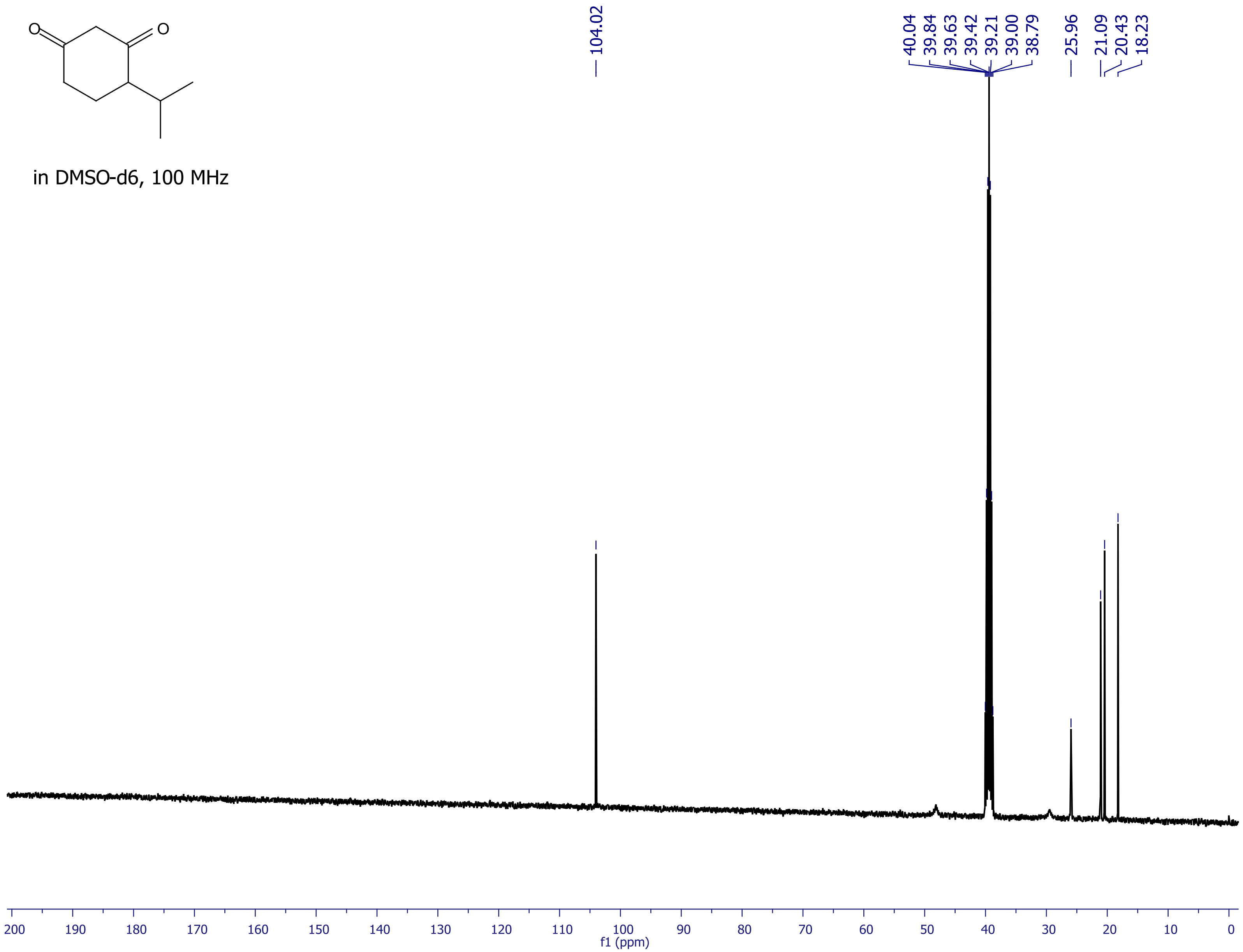
in DMSO-d6, 100 MHz
104.02
40.04
39.84
39.63
39.42
39.21
39.00
38.79
25.96
21.09
20.43
18.23
200 190 180 170 160 150 140 130 120 110 100 90 80 70 60 50 40 30 20 10 0
f1 (ppm)

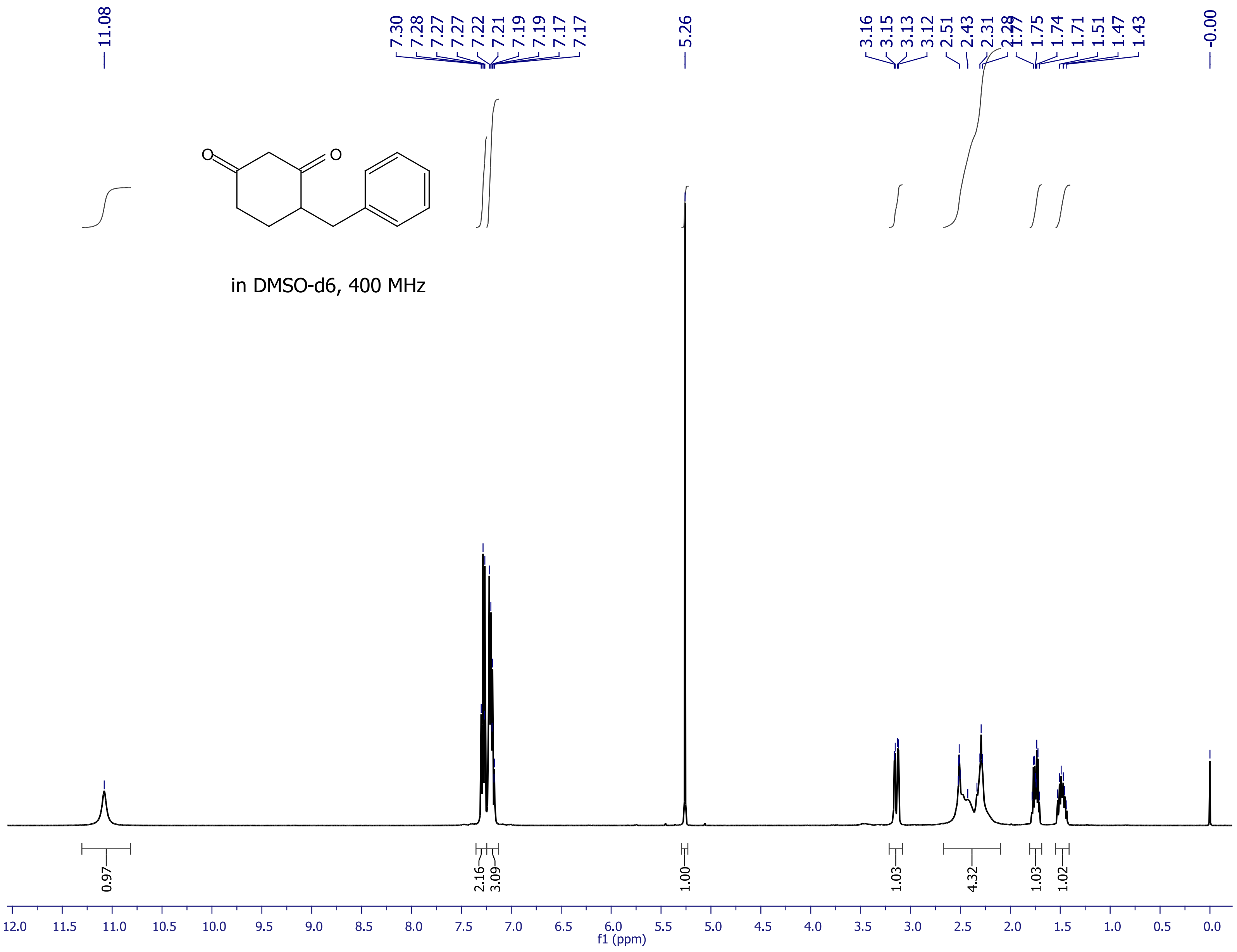

in DMSO-d6, 400 MHz
11.08
7.30
7.28
7.27
7.27
7.22
7.21
7.19
7.19
7.17
7.17
5.26
3.16
3.15
3.13
3.12
2.51
2.43
2.31
2.28
1.77
1.75
1.74
1.71
1.51
1.47
1.43
-0.00
0.97
2.16
3.09
1.00
1.03
4.32
1.03
1.02
f1 (ppm)

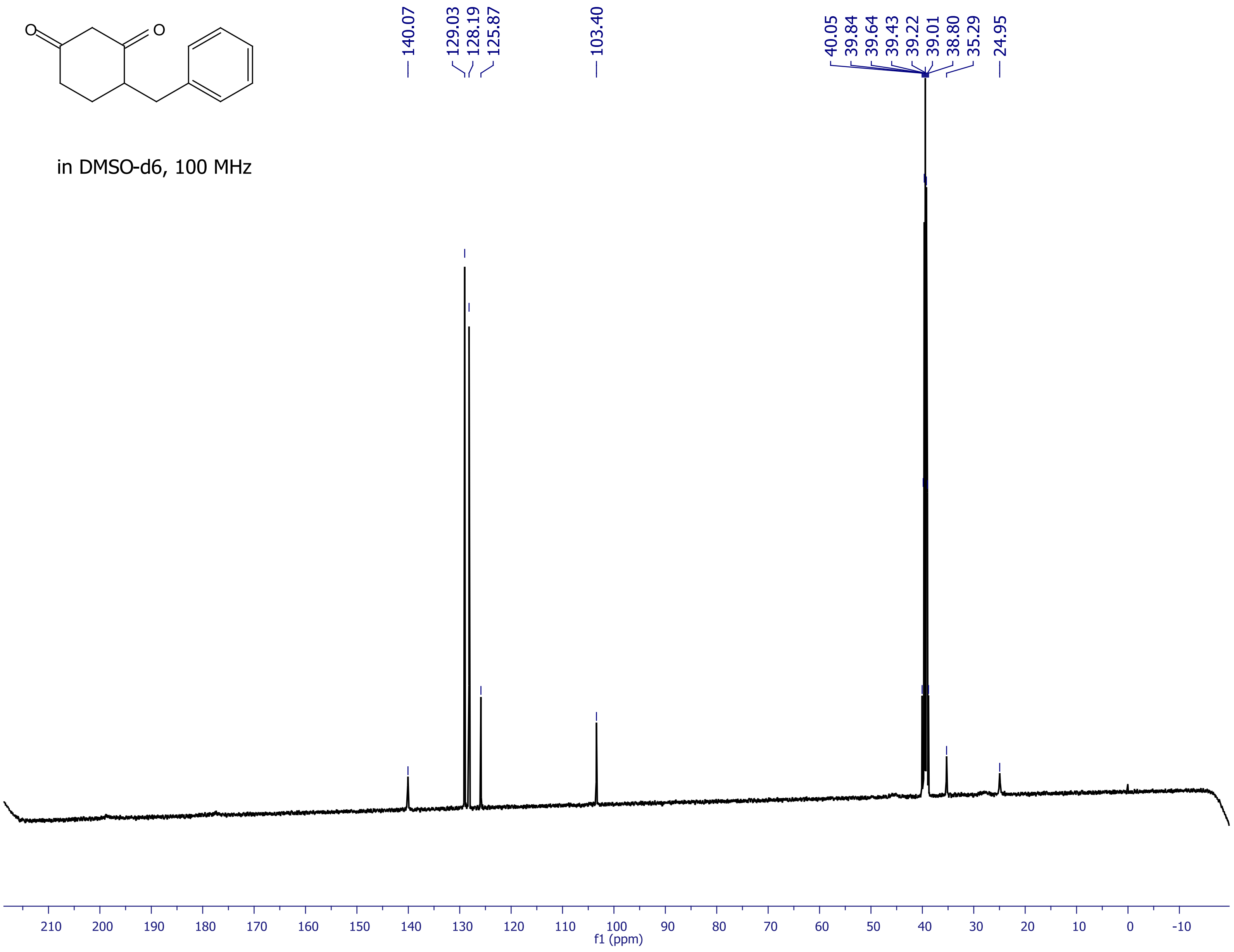
O
O
in DMSO-d6, 100 MHz
140.07
129.03
128.19
125.87
103.40
40.05
39.84
39.64
39.43
39.22
39.01
38.80
35.29
24.95
210 200 190 180 170 160 150 140 130 120 110 100 90 80 70 60 50 40 30 20 10 0 -10
f1 (ppm)

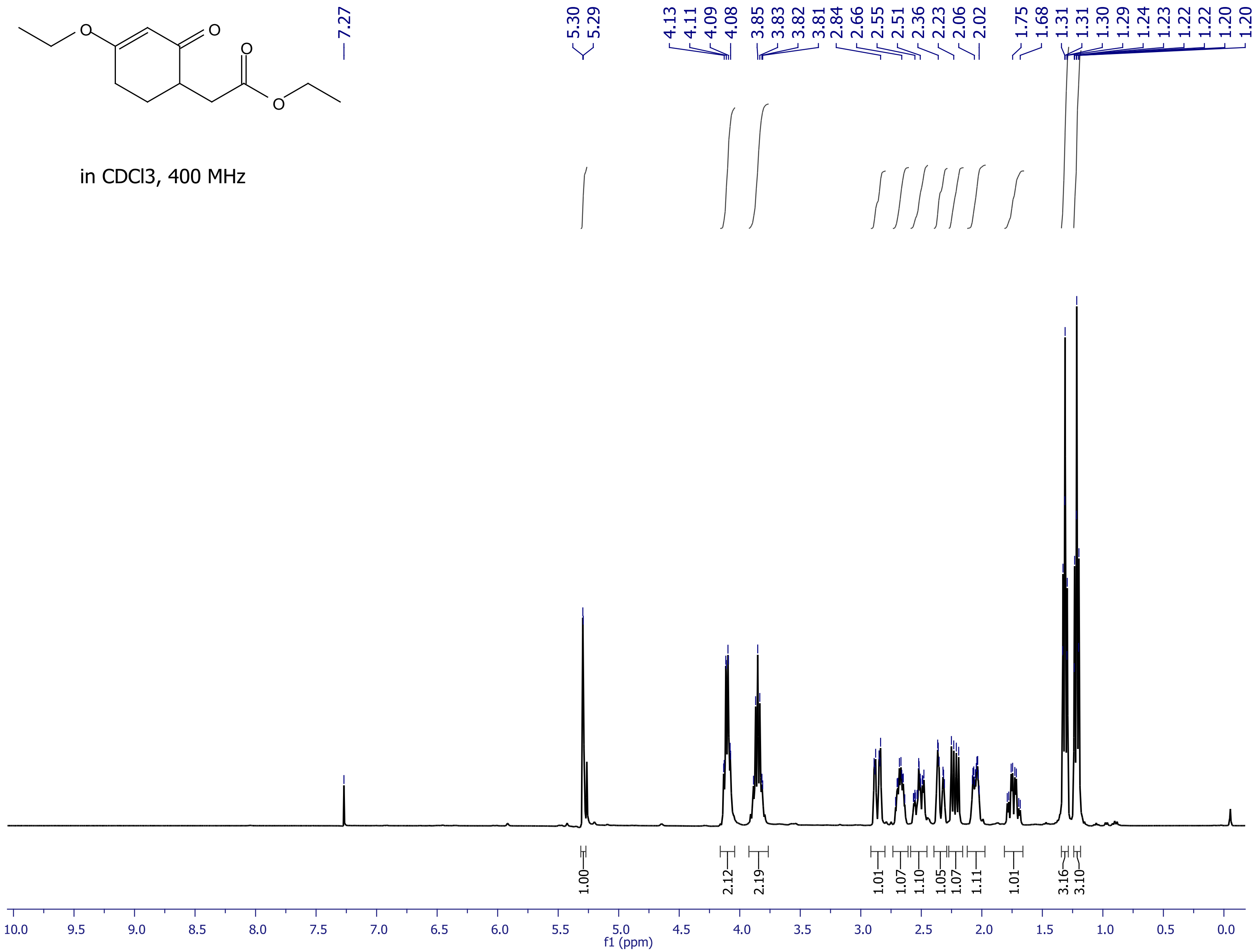
in CDCl3, 400 MHz
7.27
5.30
5.29
4.13
4.11
4.09
4.08
3.85
3.83
3.82
3.81
2.84
2.66
2.55
2.51
2.36
2.23
2.06
2.02
1.75
1.68
1.31
1.31
1.30
1.29
1.24
1.23
1.22
1.22
1.20
1.20
1.00
2.12
2.19
1.01
1.07
1.10
1.05
1.07
1.11
1.01
3.16
3.10
10.0 9.5 9.0 8.5 8.0 7.5 7.0 6.5 6.0 5.5 5.0 4.5 4.0 3.5 3.0 2.5 2.0 1.5 1.0 0.5 0.0
f1 (ppm)

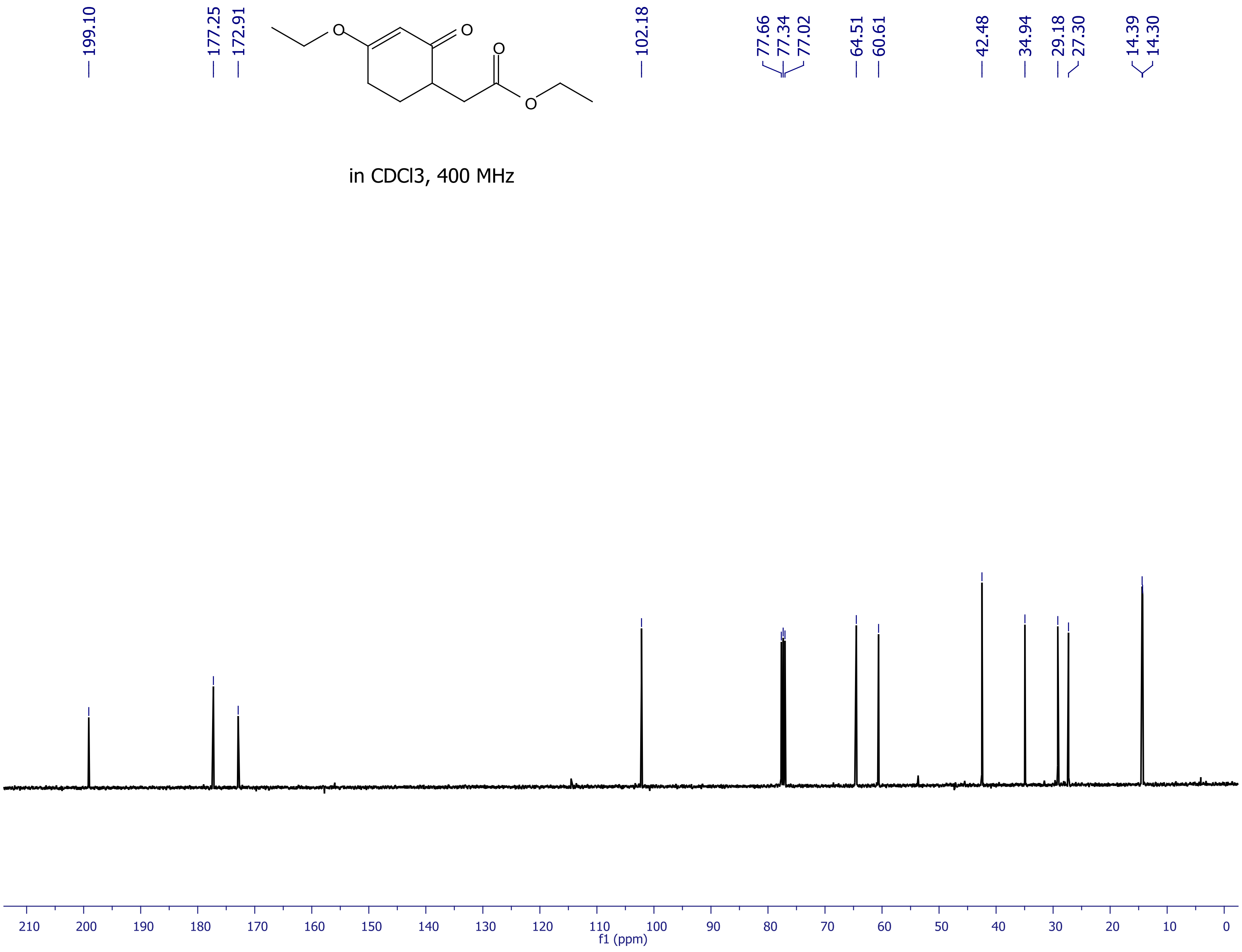
199.10
177.25
172.91
102.18
77.66
77.34
77.02
64.51
60.61
42.48
34.94
29.18
27.30
14.39
14.30
in CDCl3, 400 MHz
210
200
190
180
170
160
150
140
130
120
110
100
90
80
70
60
50
40
30
20
10
0
f1 (ppm)

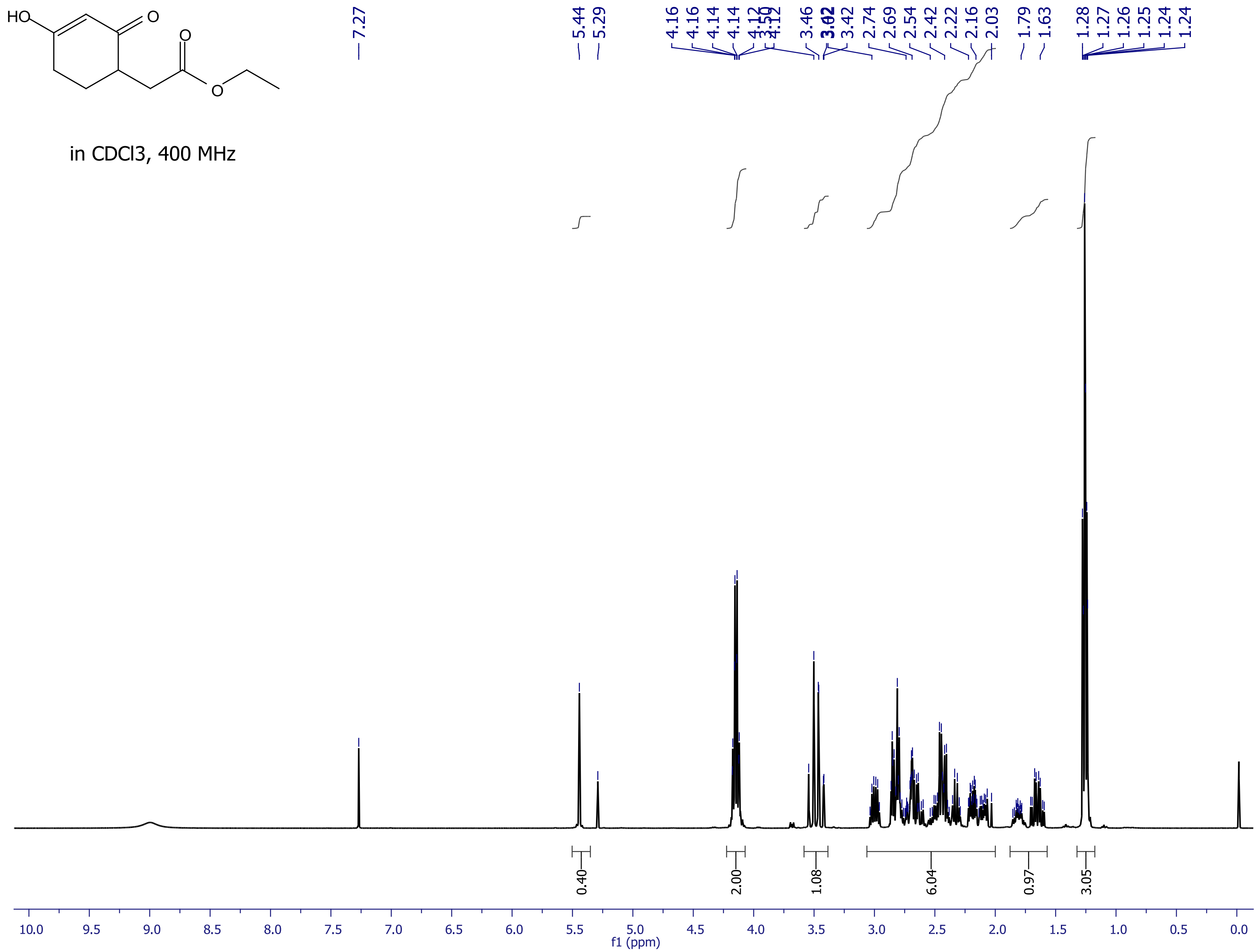
in CDCl3, 400 MHz
f1 (ppm)

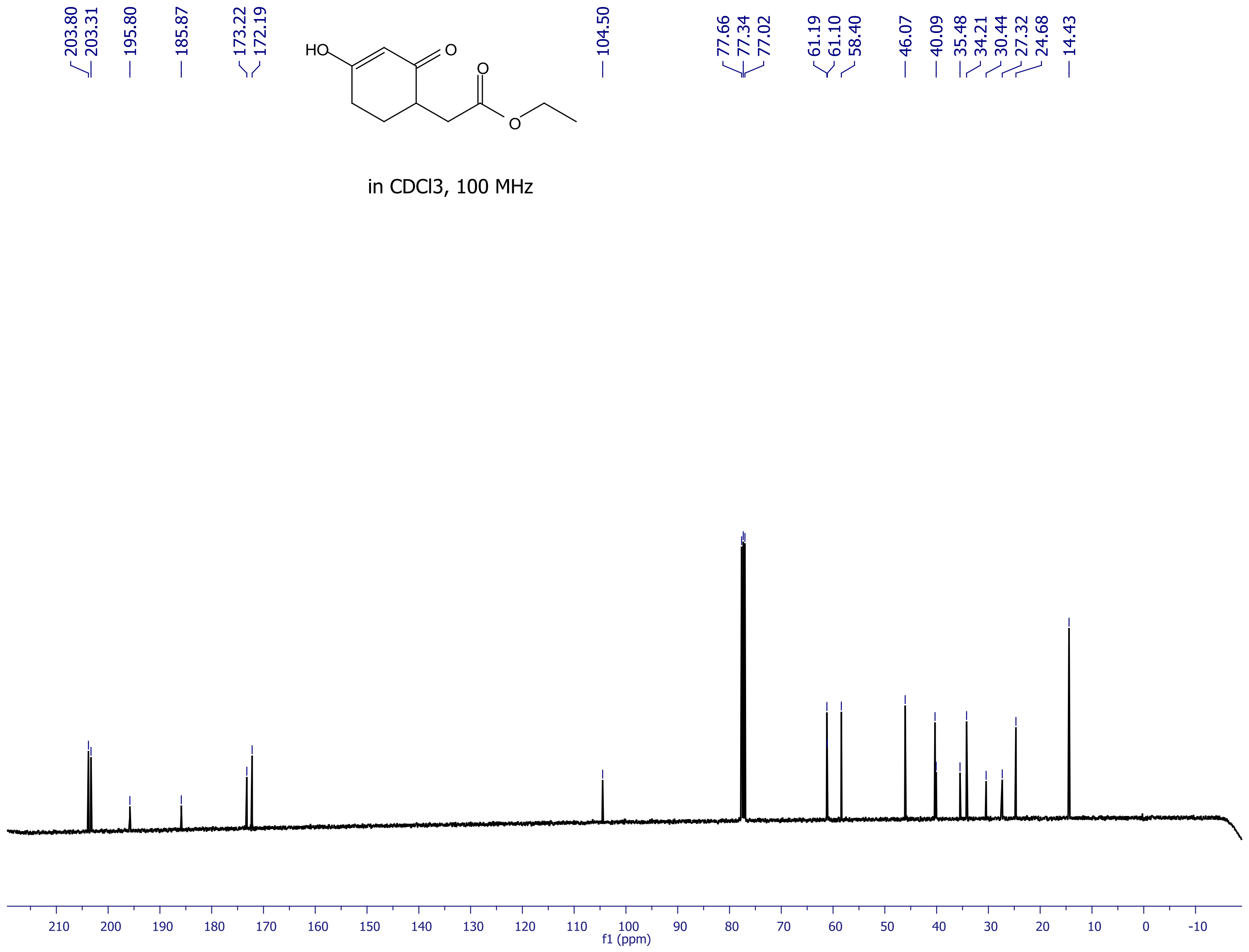

203.80
203.31
195.80
185.87
173.22
172.19
HO
O
O
O
104.50
77.66
77.34
77.02
61.19
61.10
58.40
46.07
40.09
35.48
34.21
30.44
27.32
24.68
14.43
in CDCl3, 100 MHz
210 200 190 180 170 160 150 140 130 120 110 100 90 80 70 60 50 40 30 20 10 0 -10
f1 (ppm)

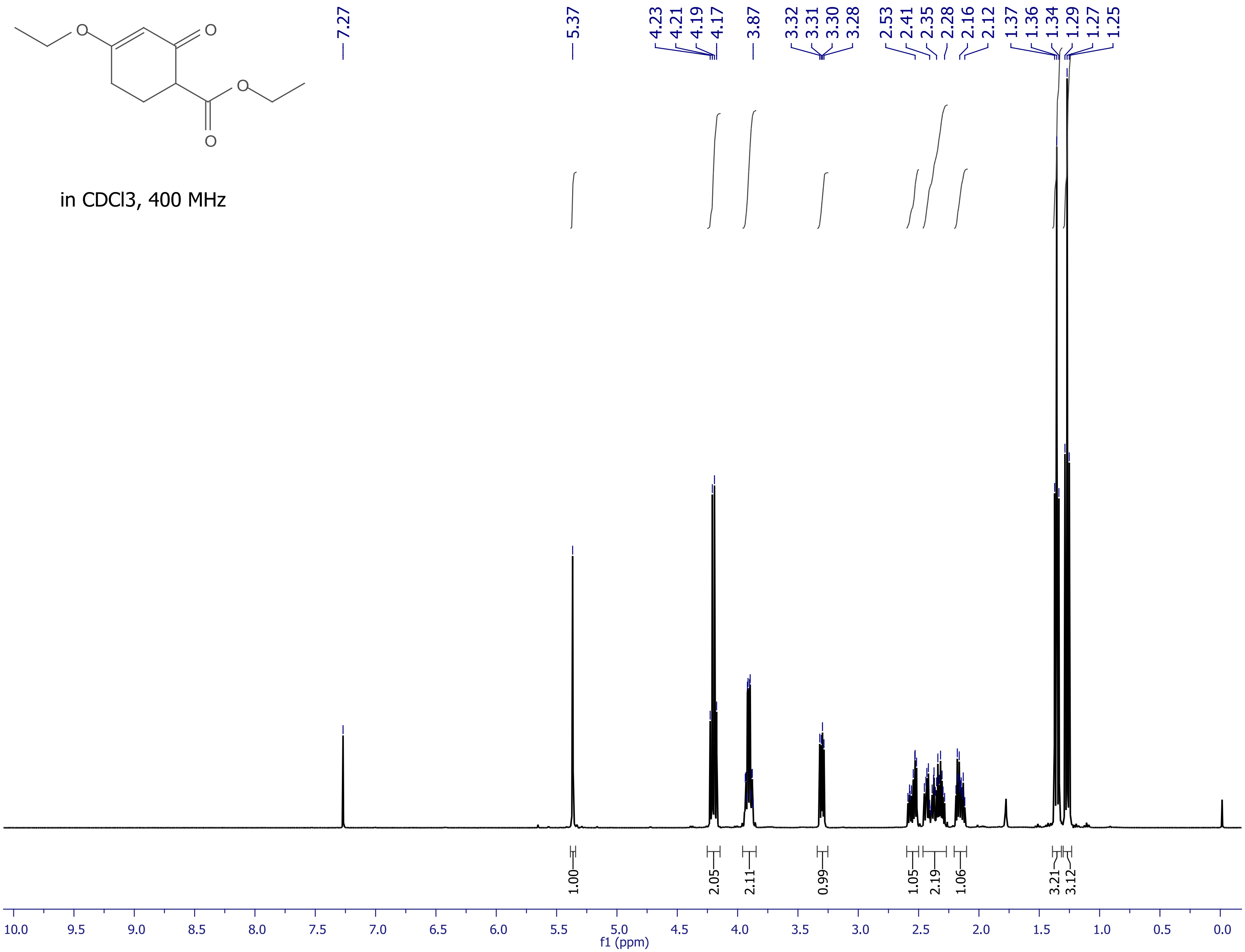
in CDCl3, 400 MHz
7.27
5.37
4.23
4.21
4.19
4.17
3.87
3.32
3.31
3.30
3.28
2.53
2.41
2.35
2.28
2.16
2.12
1.37
1.36
1.34
1.29
1.27
1.25
1.00
2.05
2.11
0.99
1.05
2.19
1.06
3.21
3.12
f1 (ppm)

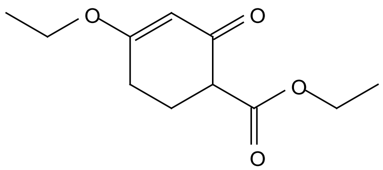

in CDCl3, 100 MHz

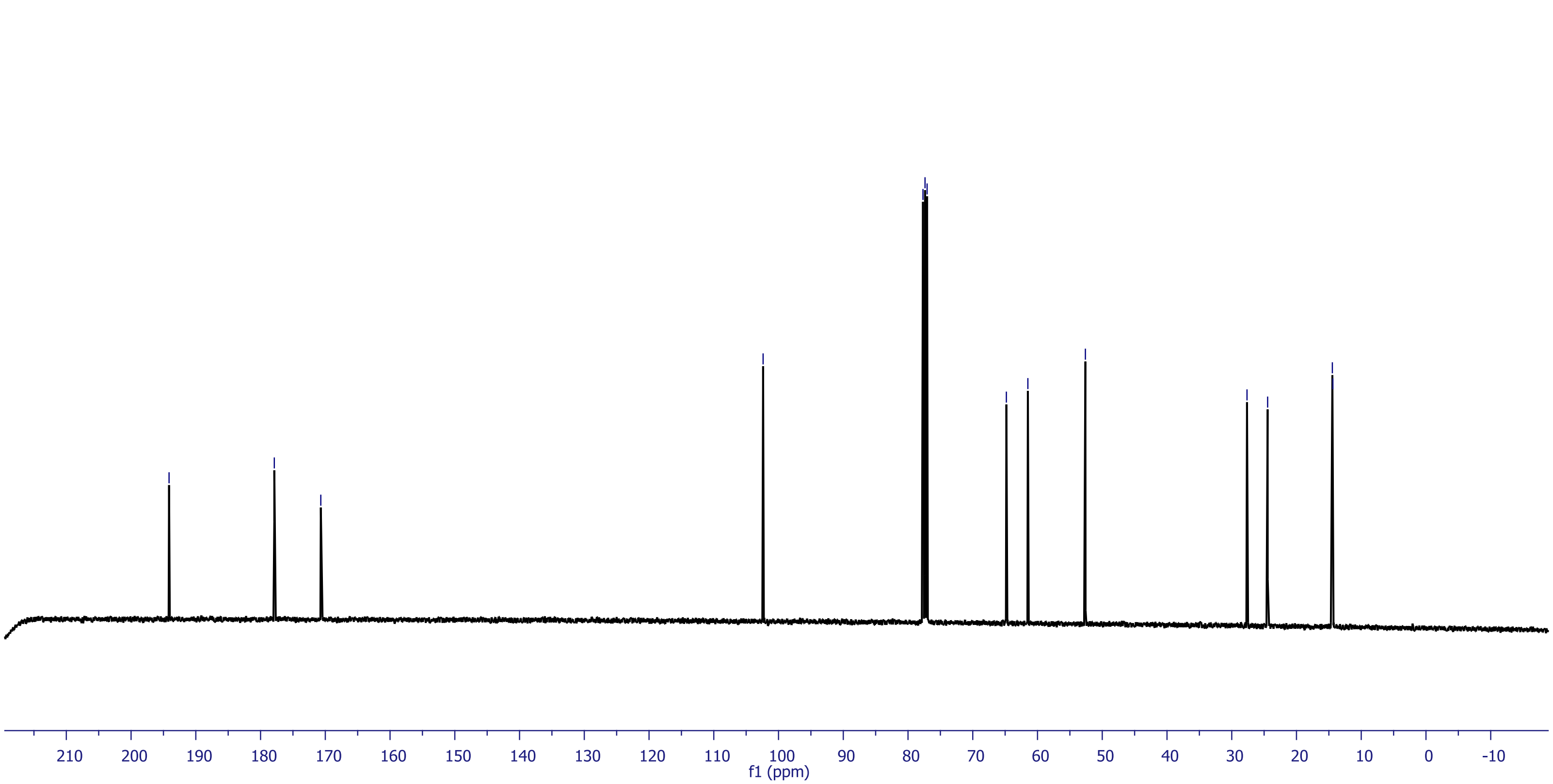

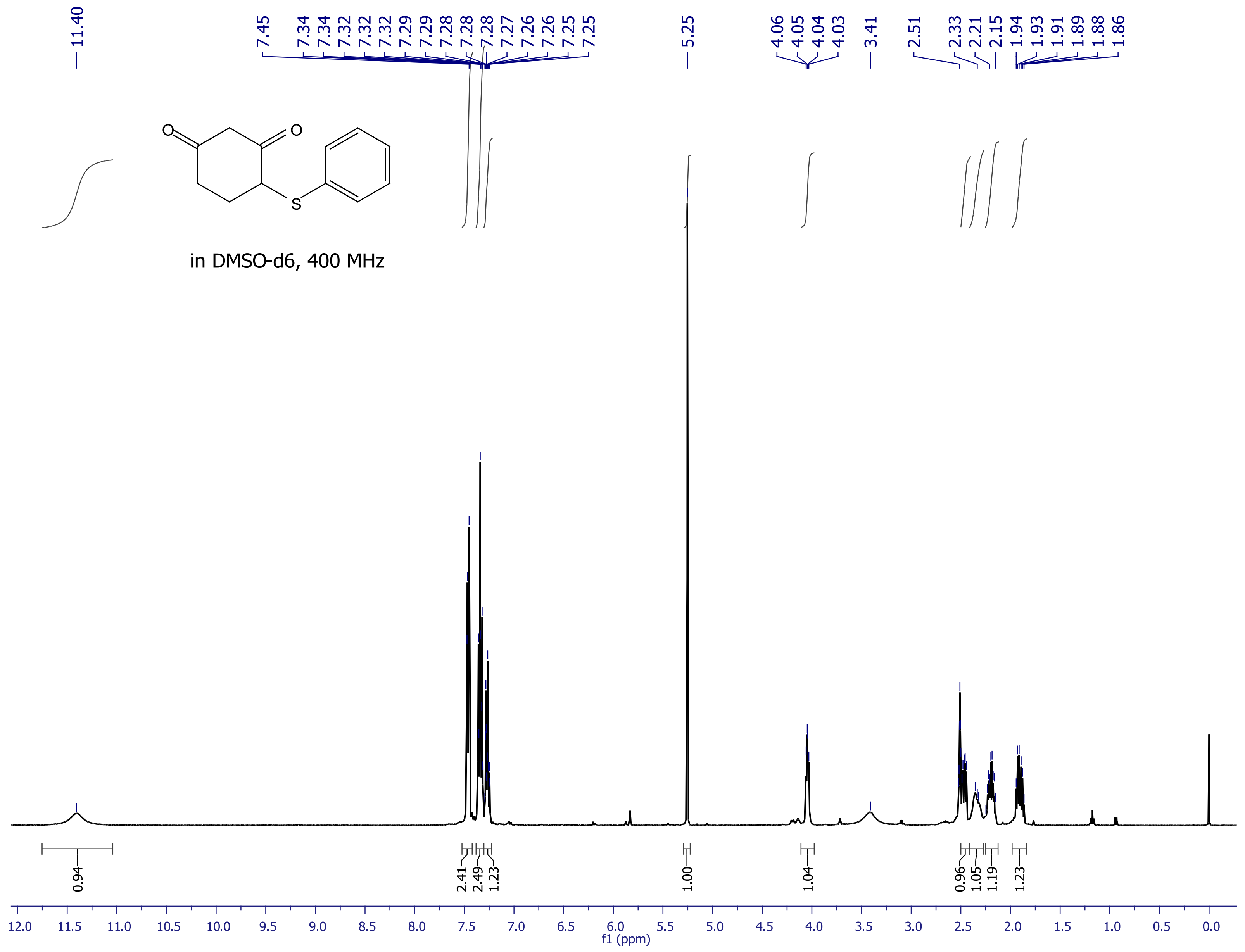
11.40
7.45 7.34 7.34 7.32 7.32 7.32 7.29 7.29 7.28 7.28 7.28 7.27 7.26 7.26 7.25 7.25
5.25
4.06 4.05 4.04 4.03
3.41
2.51
2.33 2.21 2.15
1.94 1.93 1.91 1.89 1.88 1.86
O
O
S
in DMSO-d6, 400 MHz
0.94
2.41
2.49
1.23
1.00
1.04
0.96
1.05
1.19
1.23
12.0 11.5 11.0 10.5 10.0 9.5 9.0 8.5 8.0 7.5 7.0 6.5 6.0 5.5 5.0 4.5 4.0 3.5 3.0 2.5 2.0 1.5 1.0 0.5 0.0
f1 (ppm)

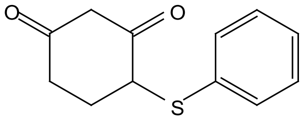

in DMSO-d6, 100 MHz

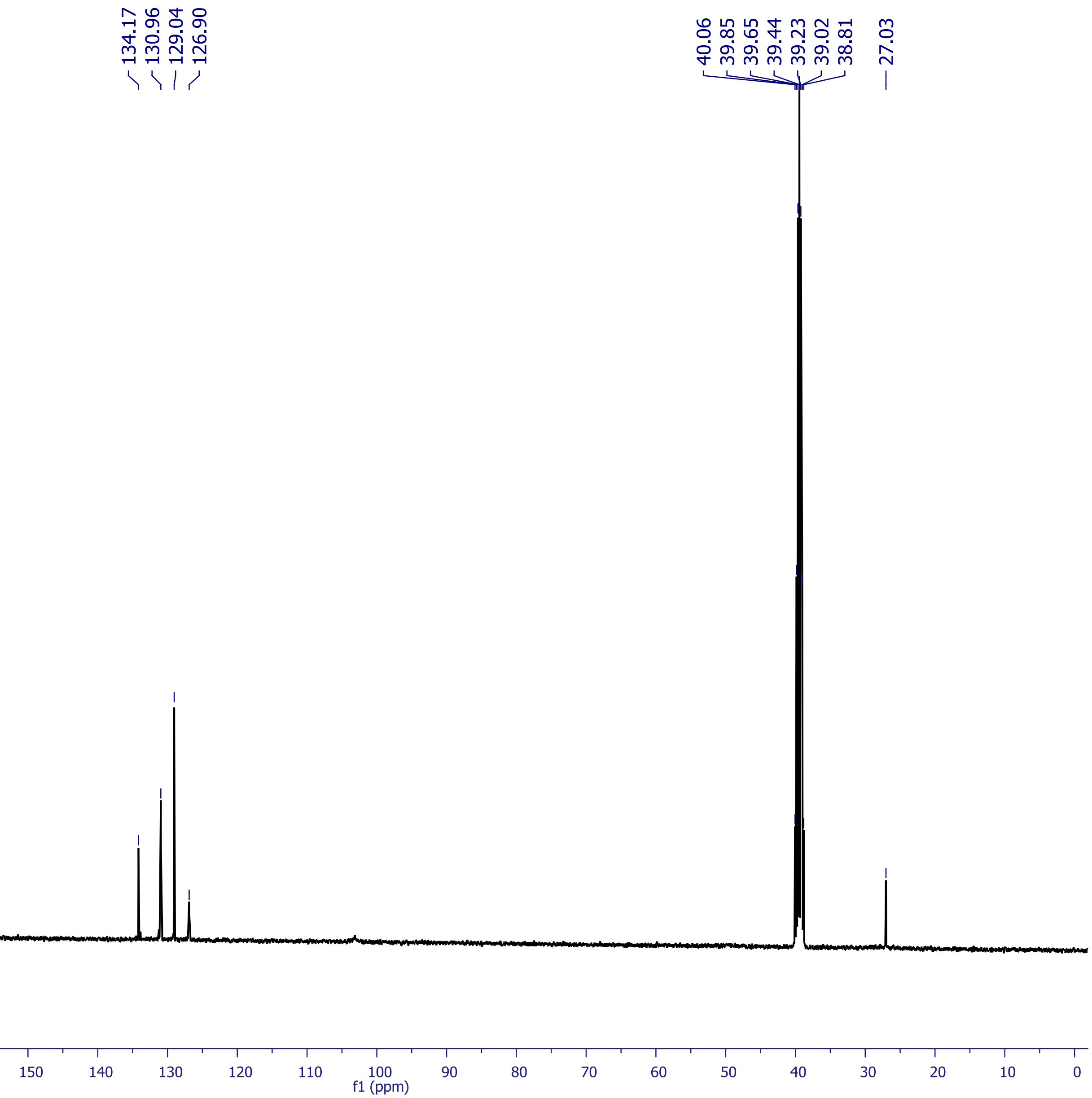

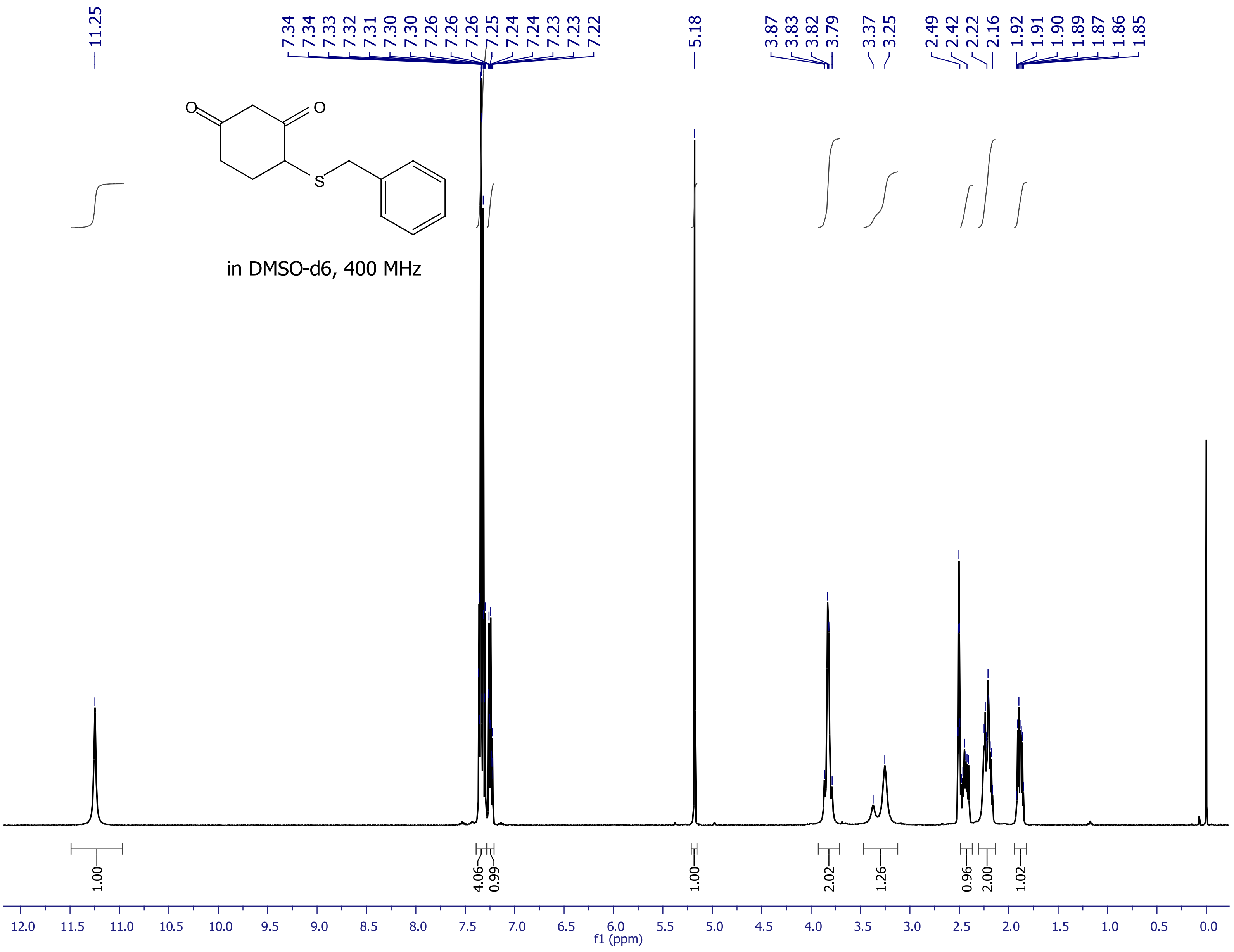

11.25
7.34 7.34 7.33 7.32 7.31 7.30 7.30 7.26 7.26 7.26 7.25 7.24 7.24 7.23 7.23 7.22
5.18
3.87 3.83 3.82 3.79
3.37 3.25
2.49 2.42 2.22 2.16
1.92 1.91 1.90 1.89 1.87 1.86 1.85
O
O
S
in DMSO-d6, 400 MHz
1.00
4.06
0.99
1.00
2.02
1.26
0.96
2.00
1.02
12.0 11.5 11.0 10.5 10.0 9.5 9.0 8.5 8.0 7.5 7.0 6.5 6.0 5.5 5.0 4.5 4.0 3.5 3.0 2.5 2.0 1.5 1.0 0.5 0.0
f1 (ppm)

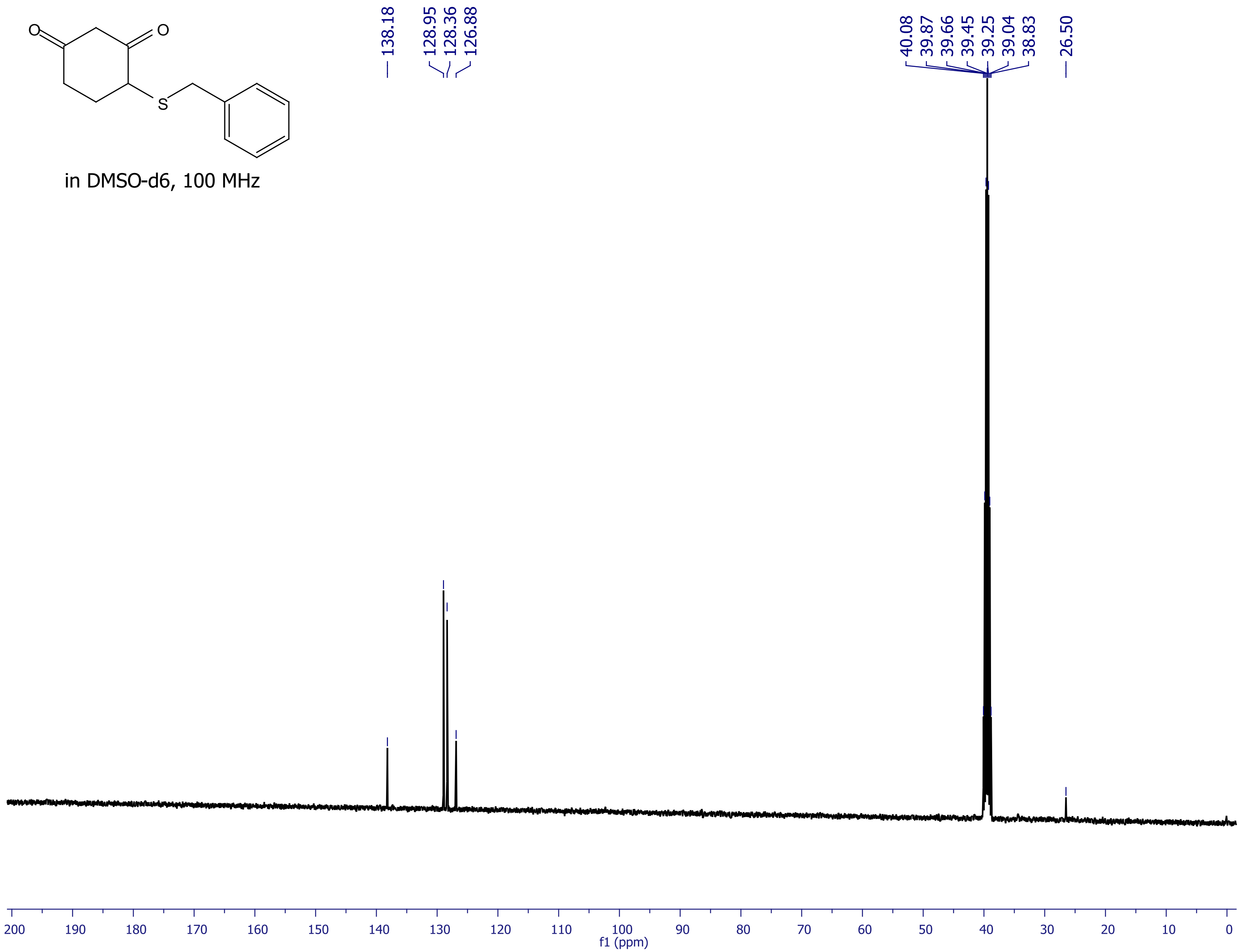

O
O
S
in DMSO-d6, 100 MHz
138.18
128.95
128.36
126.88
40.08
39.87
39.66
39.45
39.25
39.04
38.83
26.50
200
190
180
170
160
150
140
130
120
110
100
90
80
70
60
50
40
30
20
10
0
f1 (ppm)

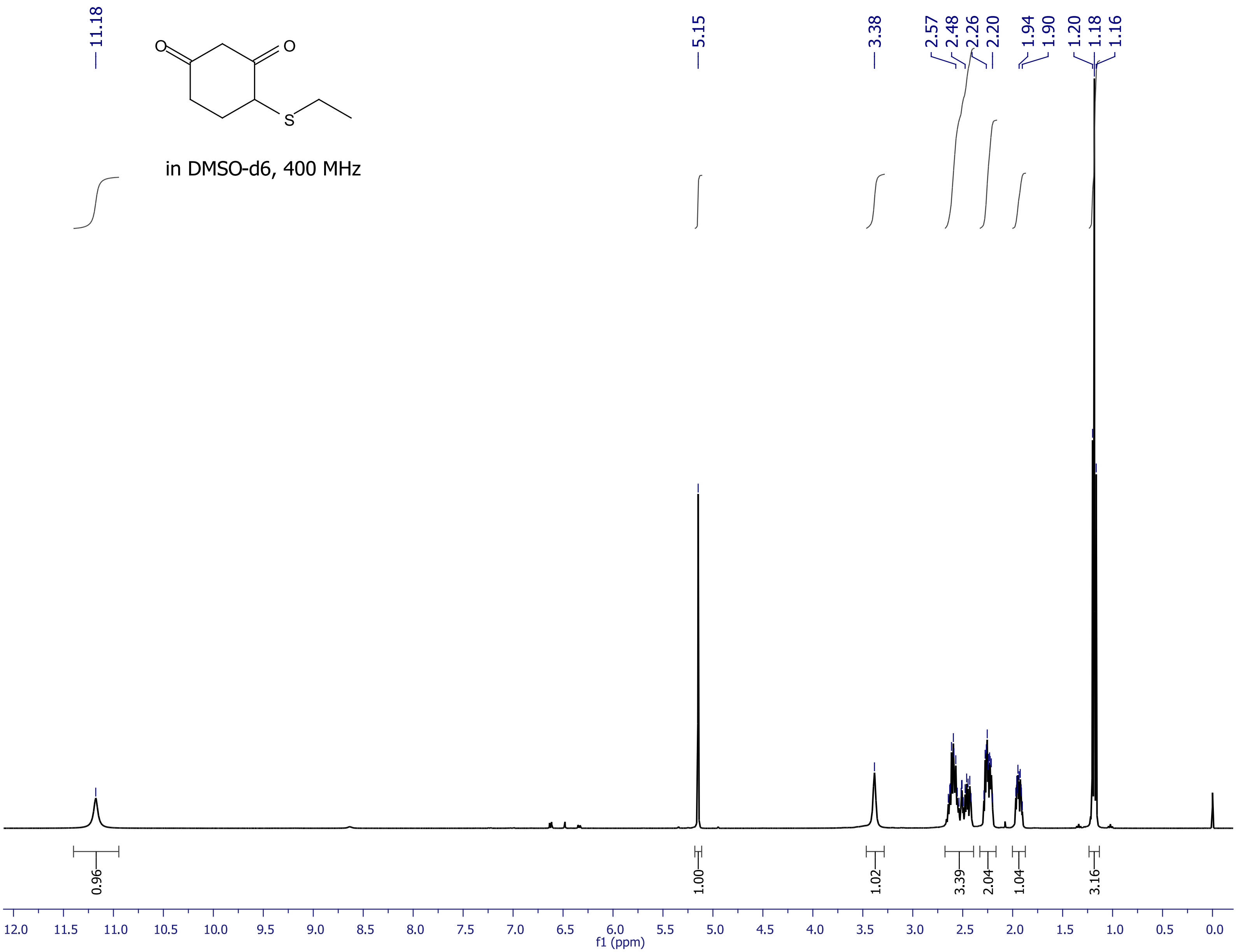

in DMSO-d6, 400 MHz
11.18
5.15
3.38
2.57
2.48
2.26
2.20
1.94
1.90
1.20
1.18
1.16
0.96
1.00
1.02
3.39
2.04
1.04
3.16
12.0
11.5
11.0
10.5
10.0
9.5
9.0
8.5
8.0
7.5
7.0
6.5
6.0
5.5
5.0
4.5
4.0
3.5
3.0
2.5
2.0
1.5
1.0
0.5
0.0
f1 (ppm)

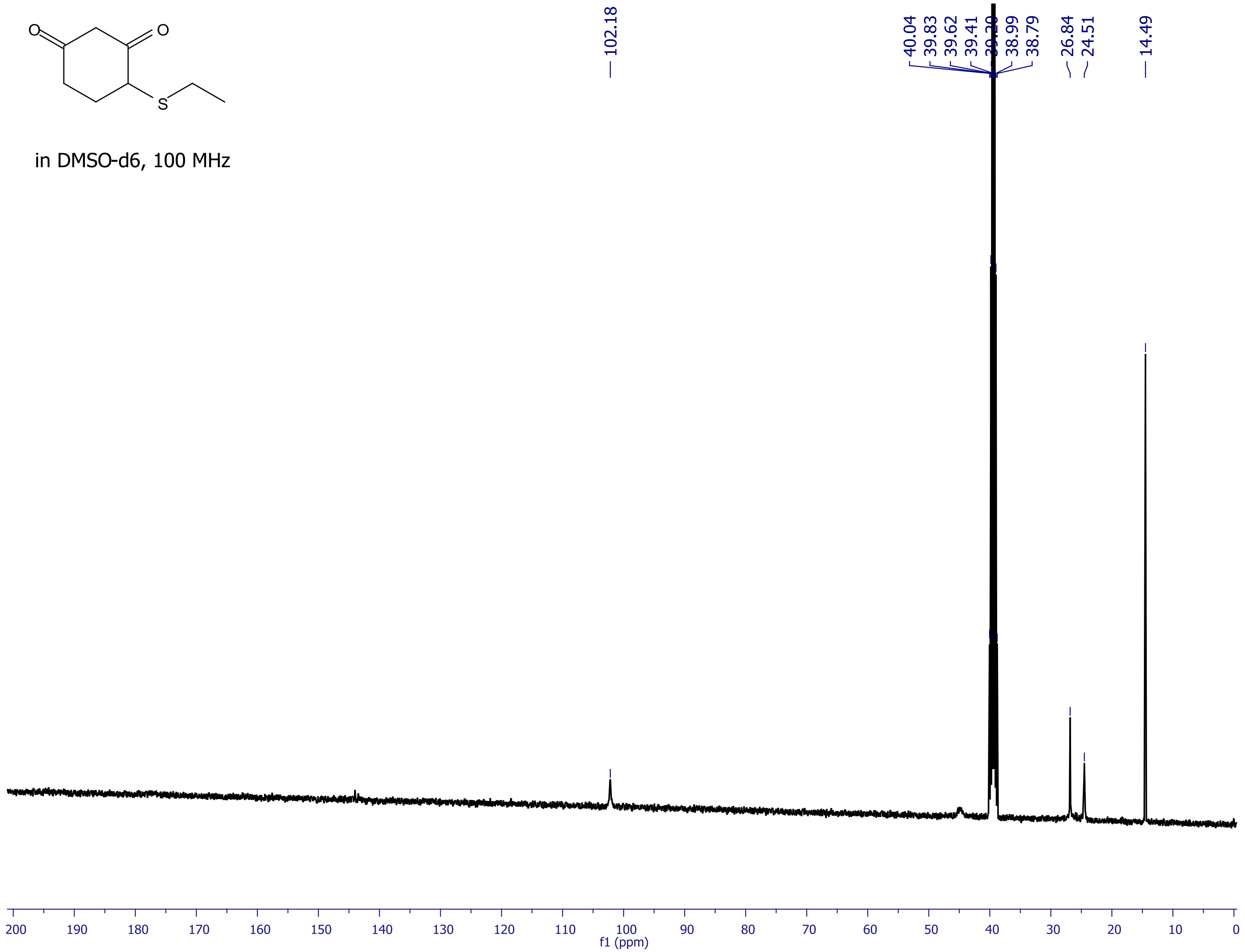
O
O
S
in DMSO-d6, 100 MHz
102.18
40.04
39.83
39.62
39.41
38.99
38.79
26.84
24.51
14.49
200 190 180 170 160 150 140 130 120 110 100 90 80 70 60 50 40 30 20 10 0
f1 (ppm)

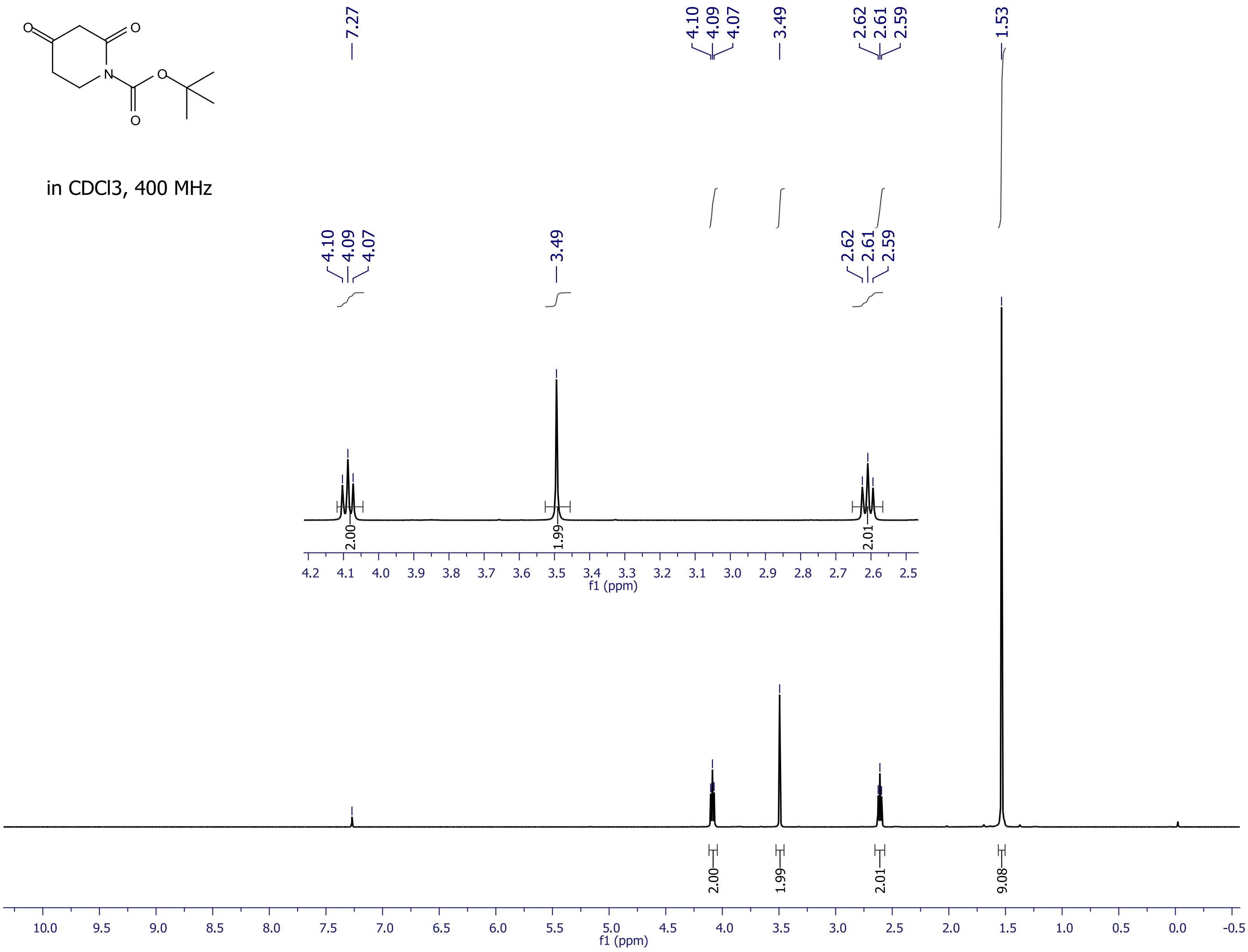

in CDCl3, 400 MHz
7.27
4.10
4.09
4.07
3.49
2.62
2.61
2.59
1.53
2.00
1.99
2.01
9.08
f1 (ppm)

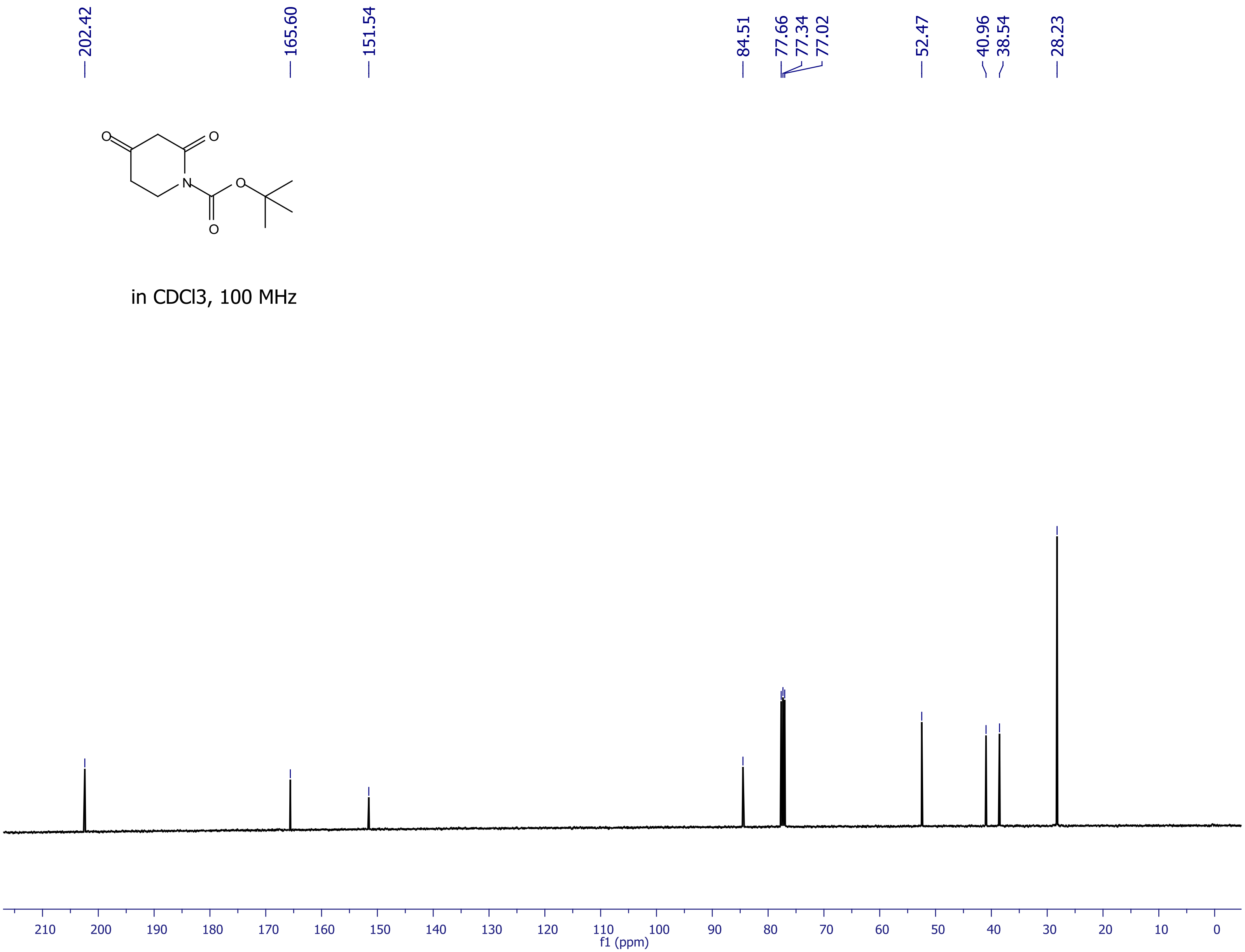
202.42
165.60
151.54
84.51
77.66
77.34
77.02
52.47
40.96
38.54
28.23
O
O
N
O
O
O
in CDCl3, 100 MHz
210 200 190 180 170 160 150 140 130 120 110 100 90 80 70 60 50 40 30 20 10 0
f1 (ppm)

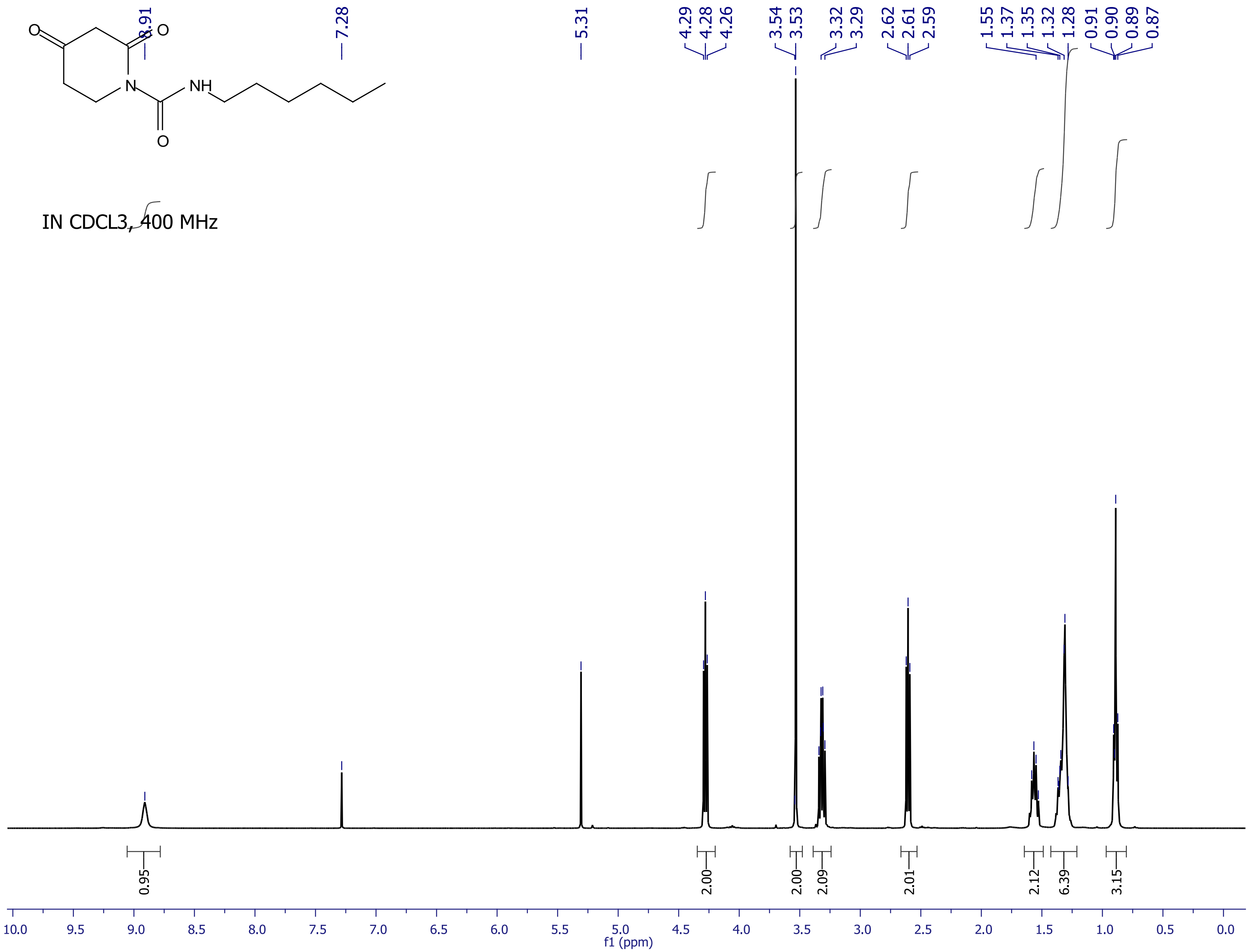

IN CDCL3, 400 MHz
8.91
7.28
5.31
4.29
4.28
4.26
3.54
3.53
3.32
3.29
2.62
2.61
2.59
1.55
1.37
1.35
1.32
1.28
0.91
0.90
0.89
0.87
0.95
2.00
2.00
2.09
2.01
2.12
6.39
3.15
10.0
9.5
9.0
8.5
8.0
7.5
7.0
6.5
6.0
5.5
5.0
4.5
4.0
3.5
3.0
2.5
2.0
1.5
1.0
0.5
0.0
f1 (ppm)

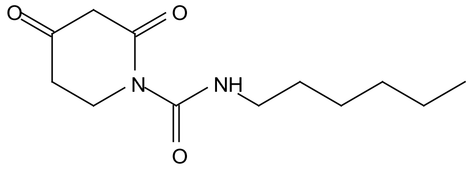

IN CDCl3, 100 MHz

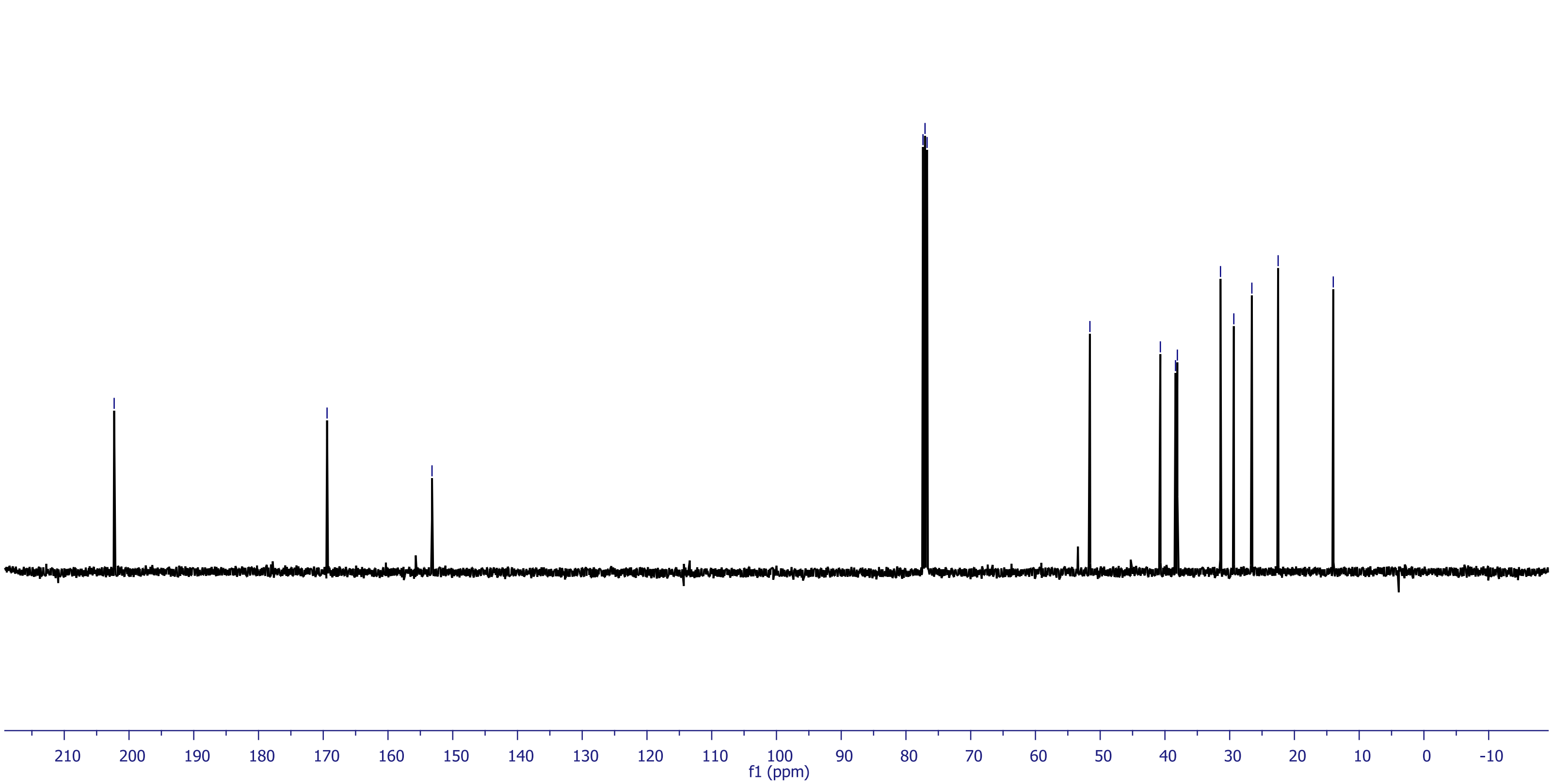

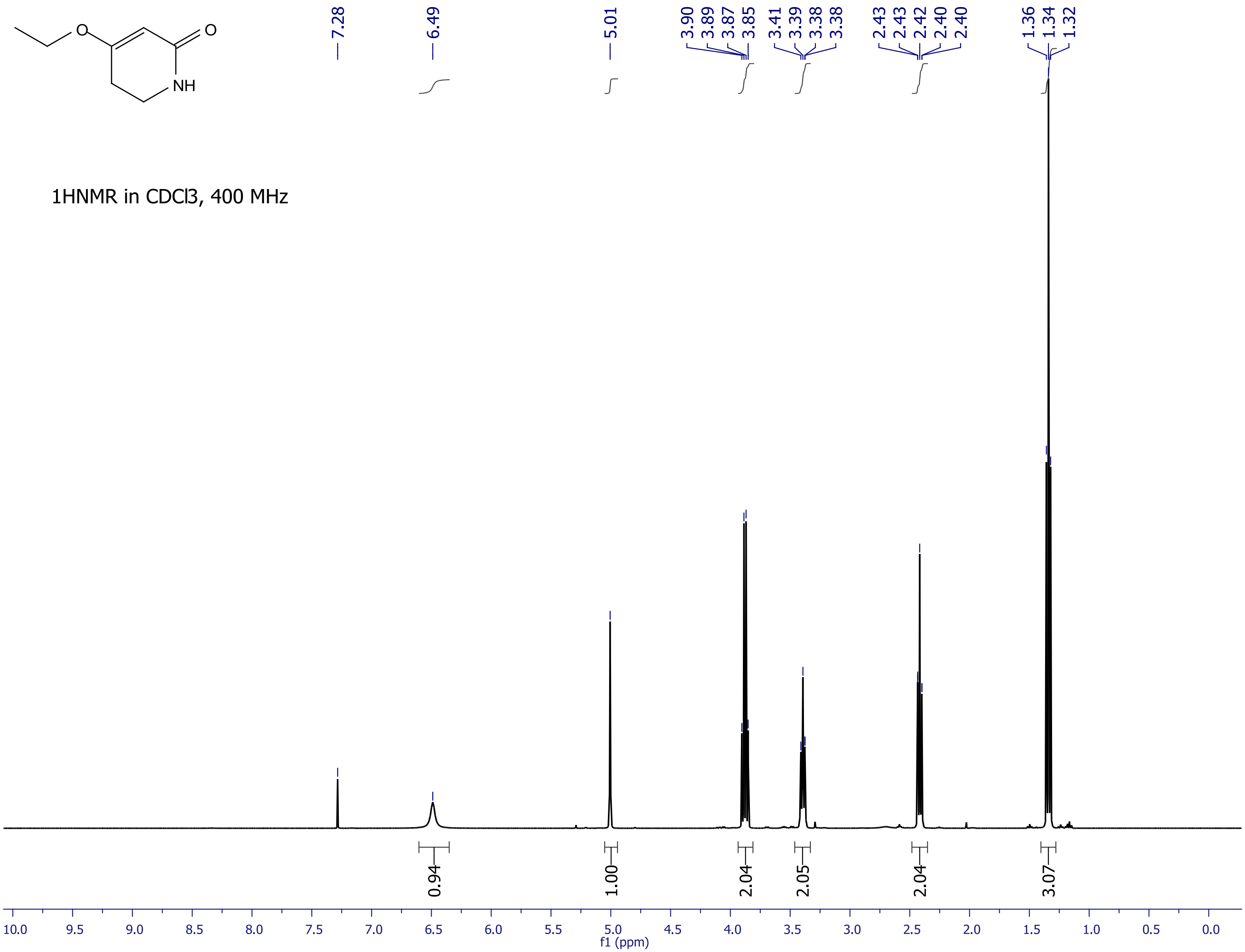

O
O
NH
1HNMR in CDCl3, 400 MHz
7.28
6.49
5.01
3.90 3.89 3.87 3.85
3.41 3.39 3.38 3.38
2.43 2.43 2.42 2.40 2.40
1.36 1.34 1.32
0.94
1.00
2.04
2.05
2.04
3.07
10.0 9.5 9.0 8.5 8.0 7.5 7.0 6.5 6.0 5.5 5.0 4.5 4.0 3.5 3.0 2.5 2.0 1.5 1.0 0.5 0.0
f1 (ppm)

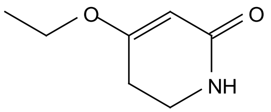

13-CNMR in CDCl3, 100 MHz

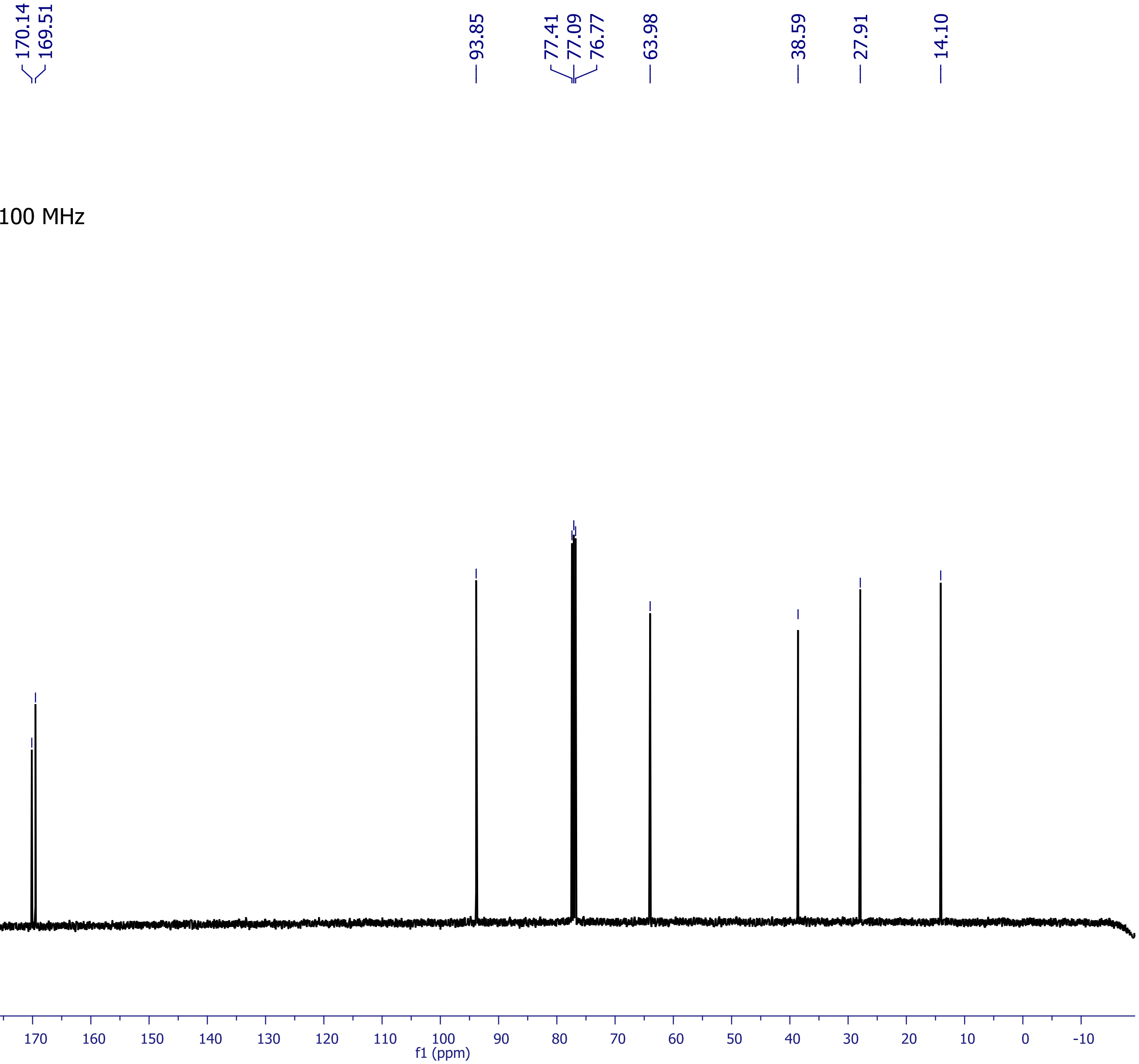

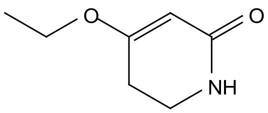

1H-NMR in DMSO-d6, 400 MHz

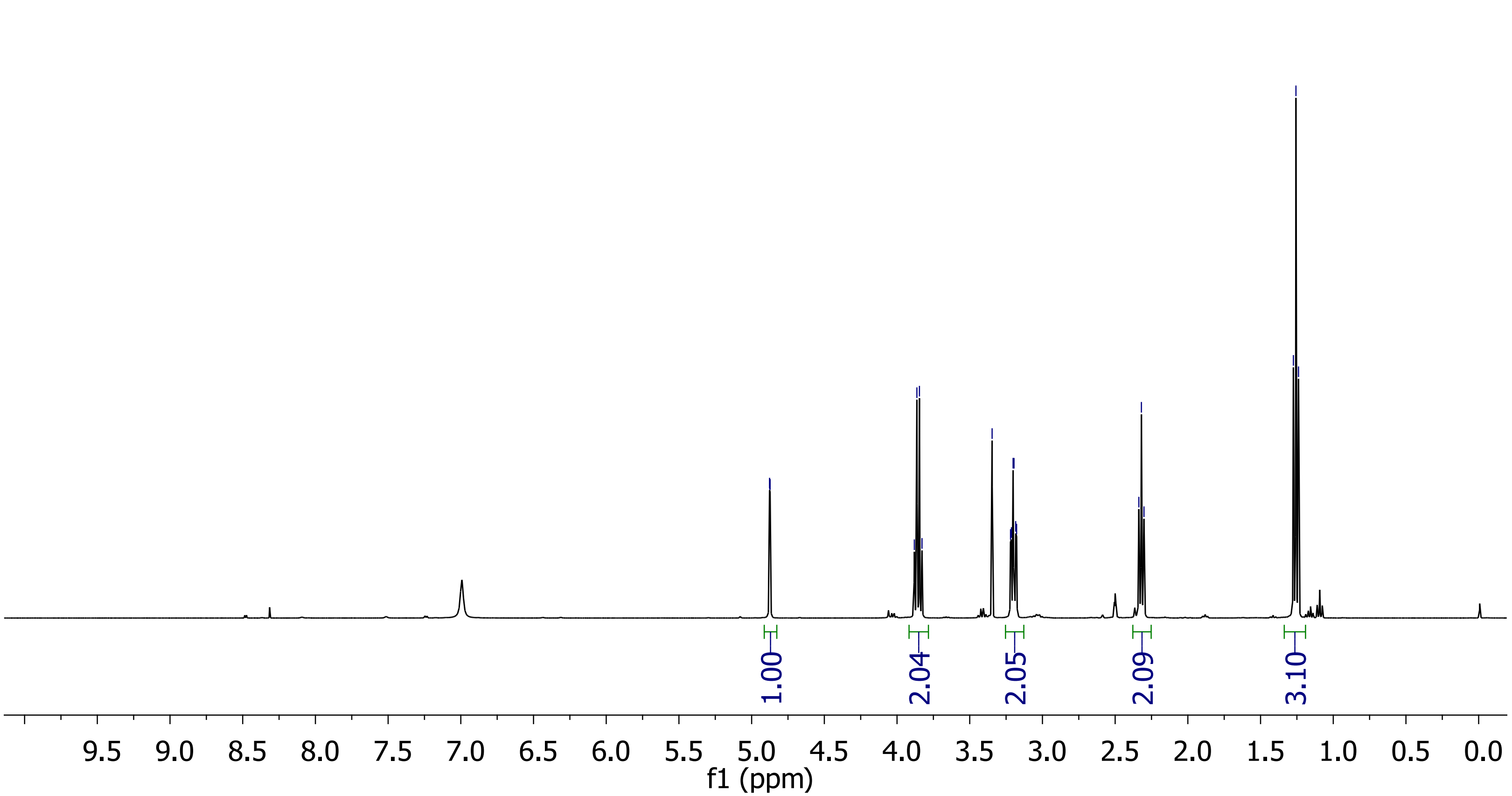

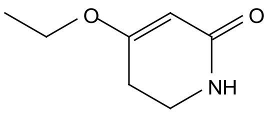

13C-NMR in DMSO-d6, 100 MHz

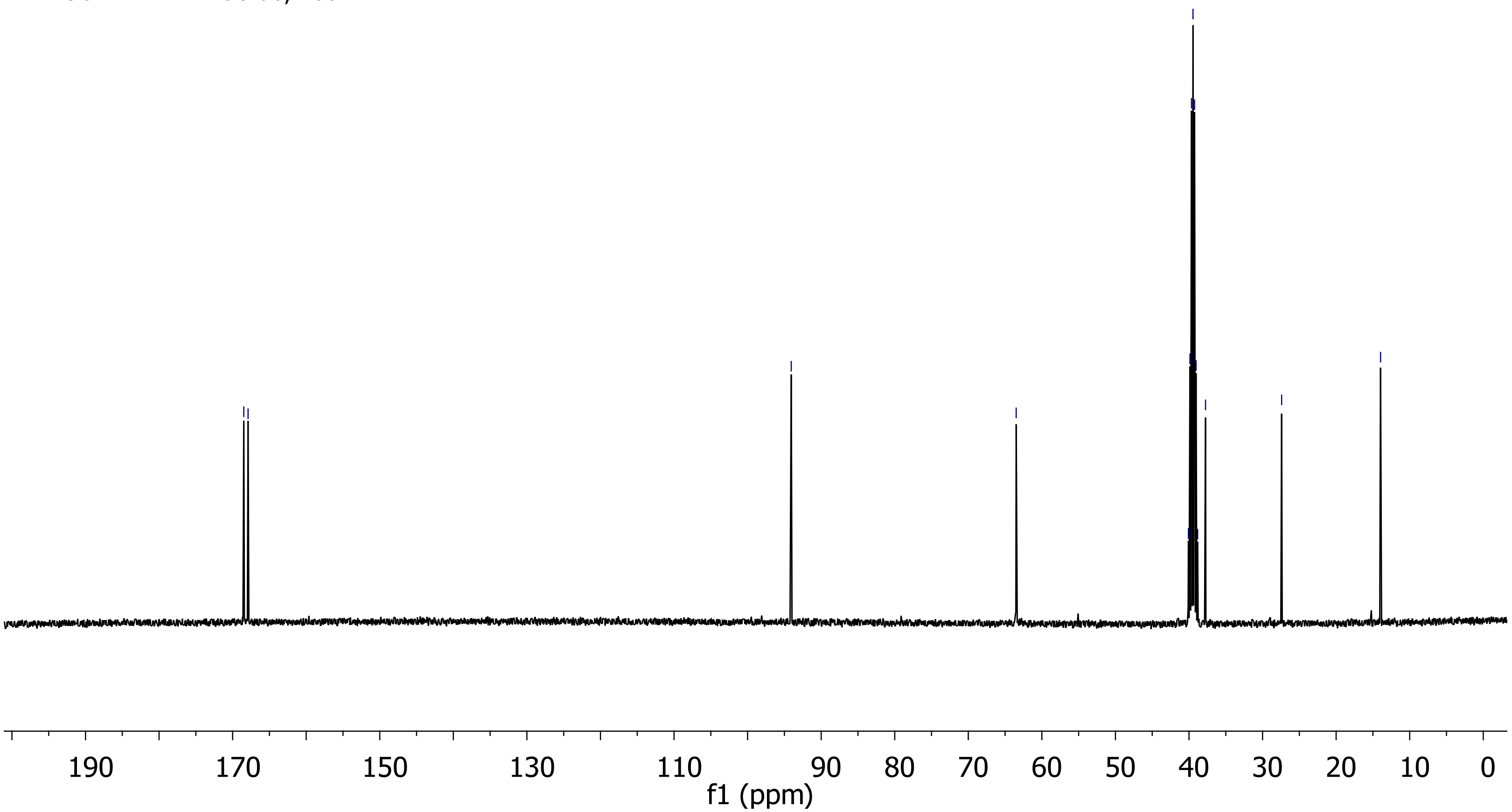

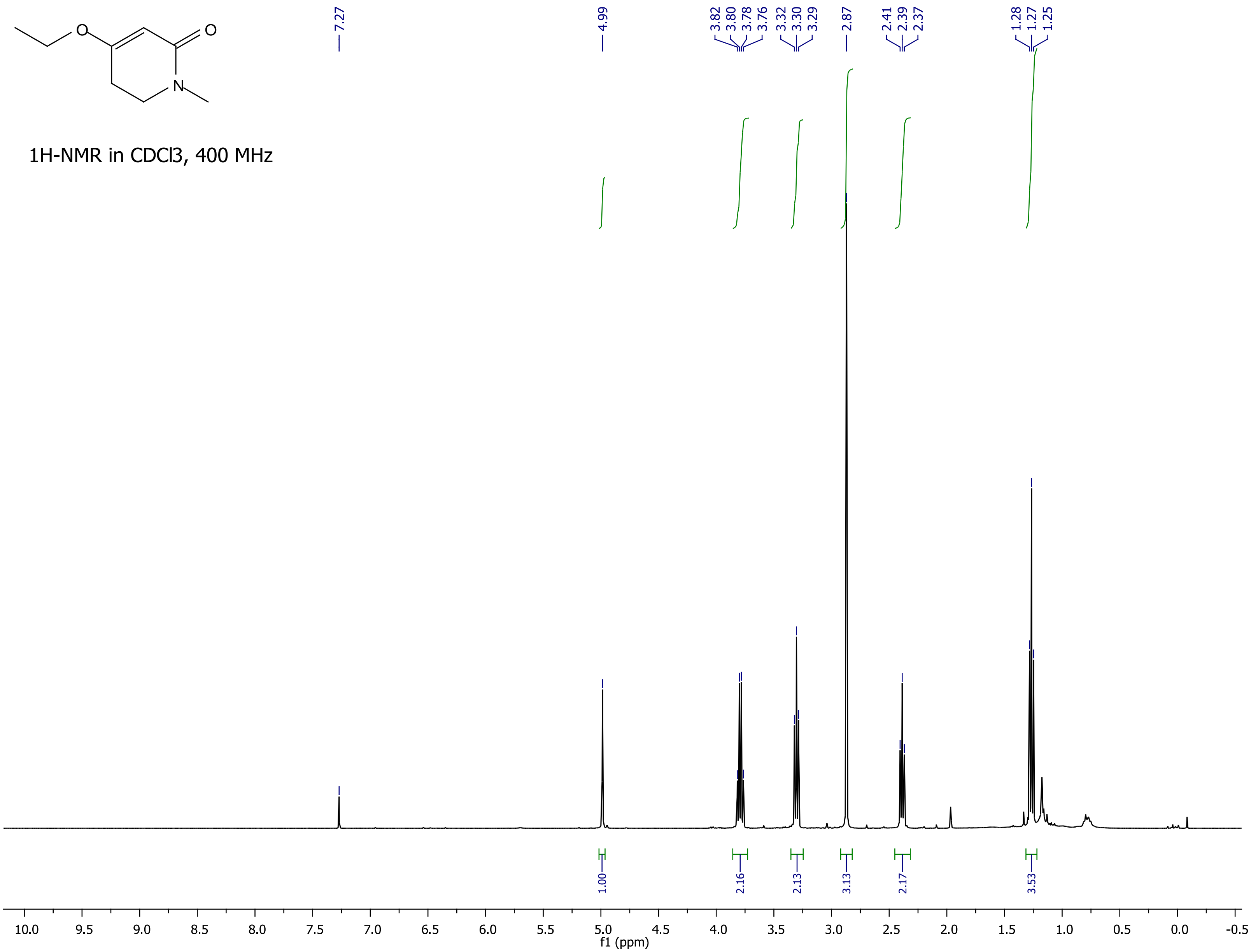

1H-NMR in CDCl3, 400 MHz
7.27
4.99
3.82
3.80
3.78
3.76
3.32
3.30
3.29
2.87
2.41
2.39
2.37
1.28
1.27
1.25
1.00
2.16
2.13
3.13
2.17
3.53
f1 (ppm)

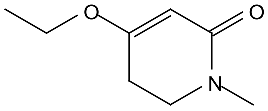

13-CNMR in CDCl3, 100 MHz

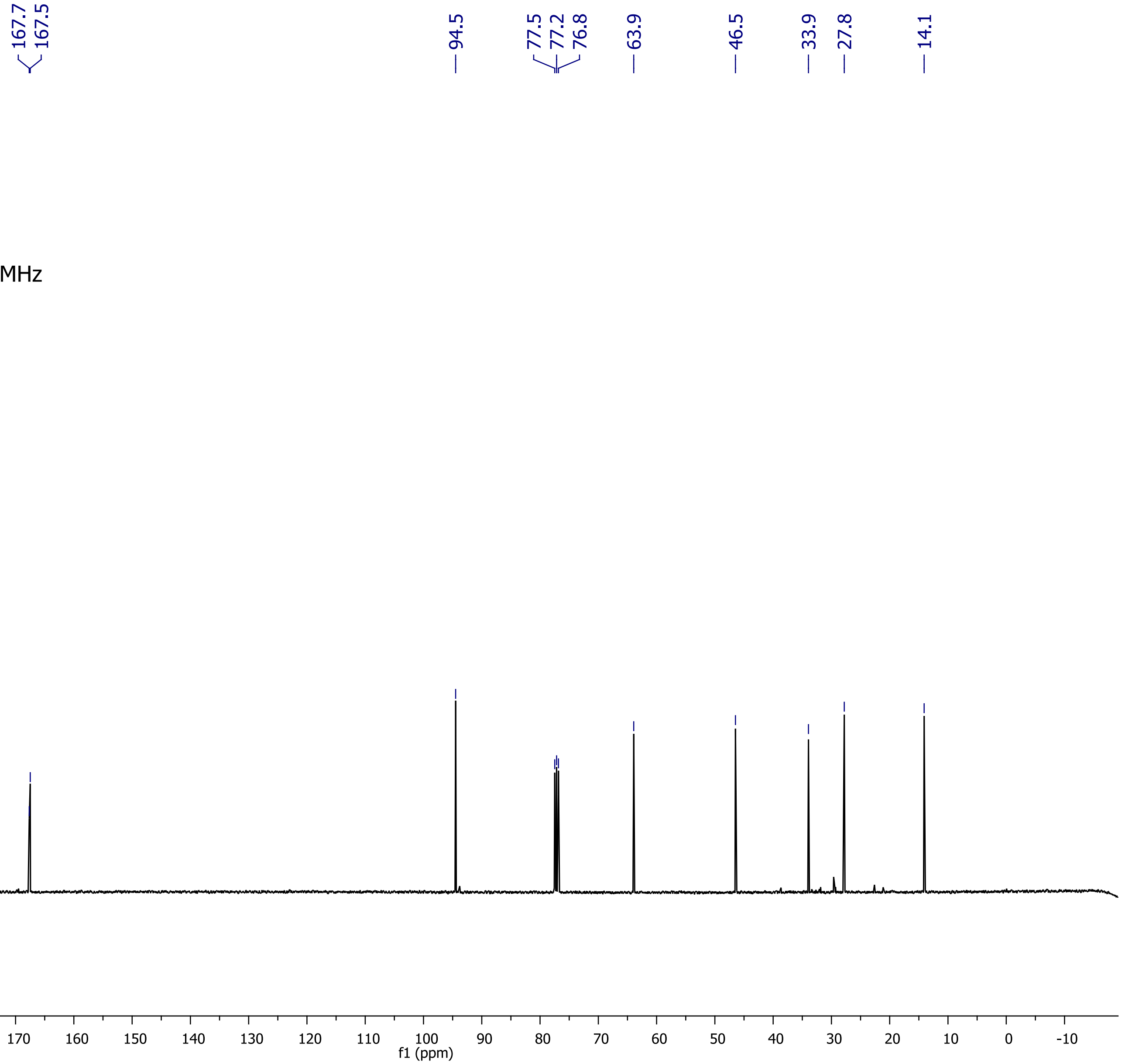

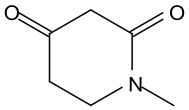

1H-NMR, in CDCl3, 400 MHz

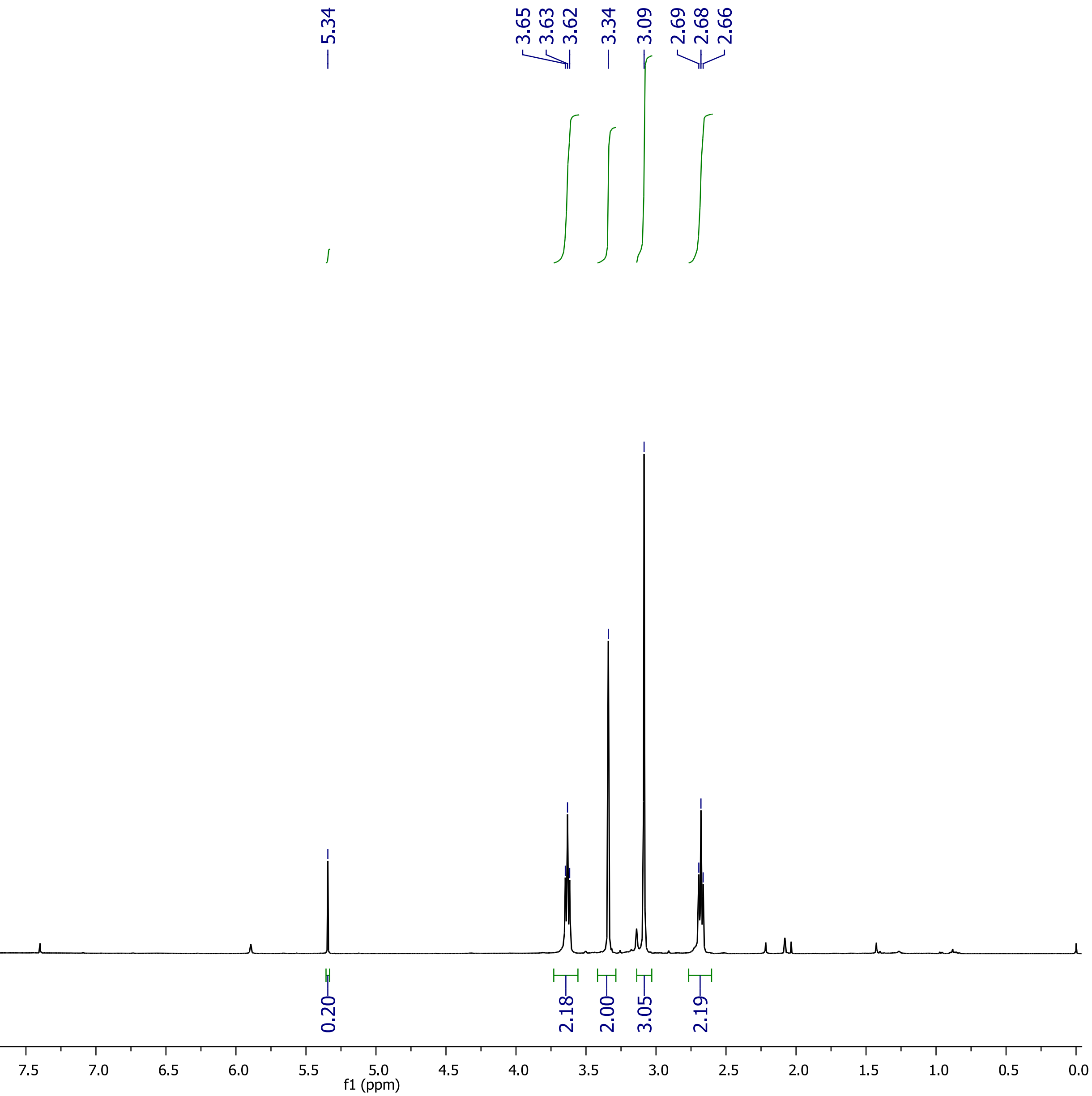

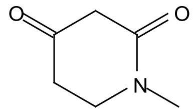

13C-NMR, in CDCl3, 100 MHz

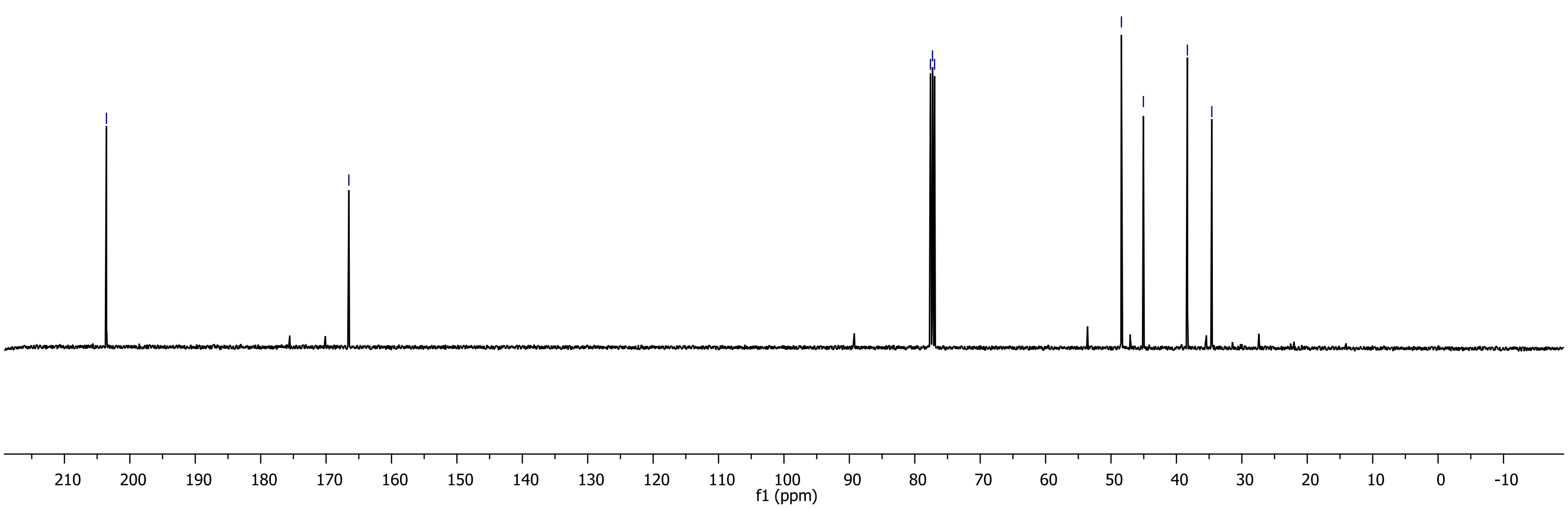

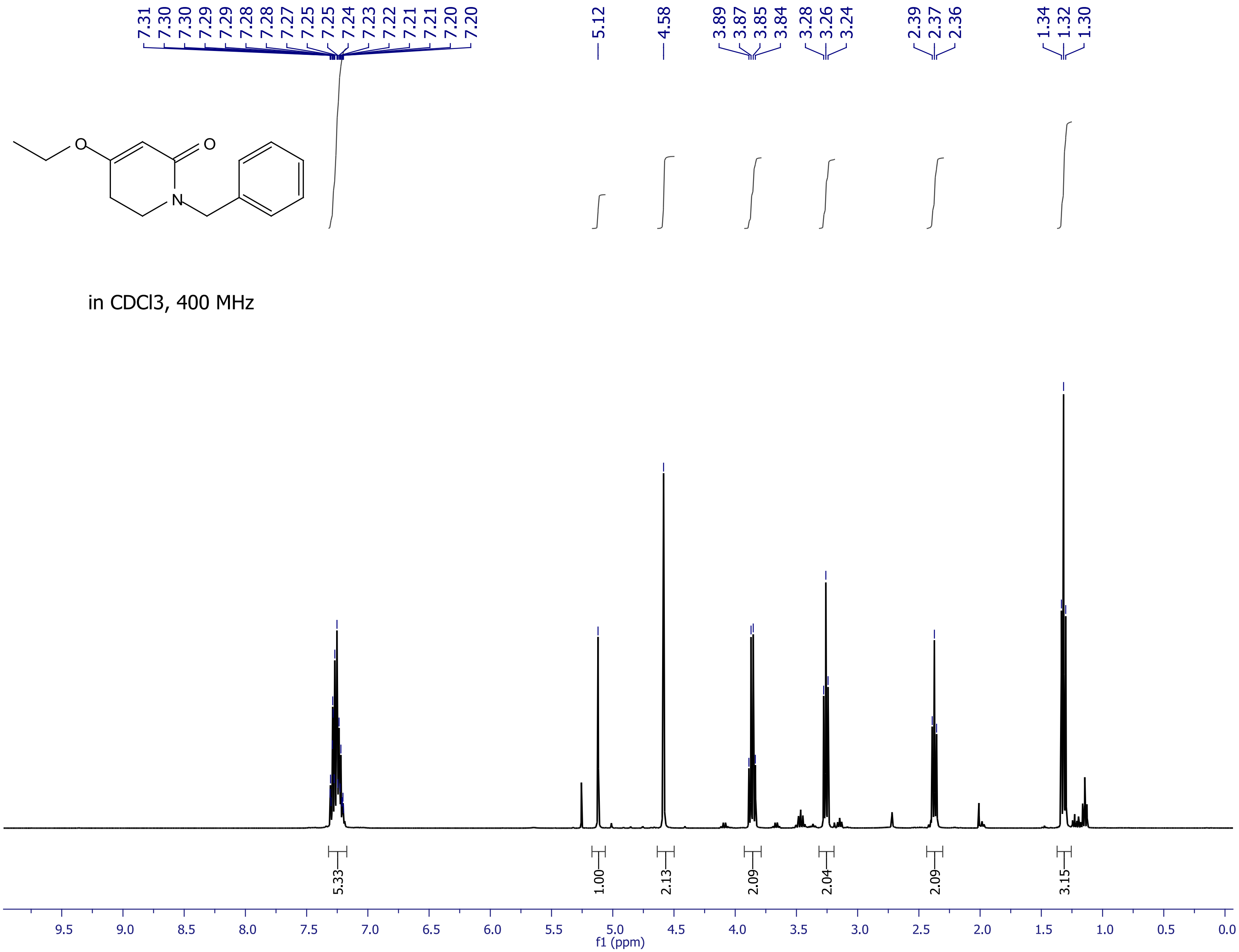
in CDCl3, 400 MHz
f1 (ppm)

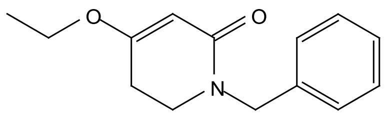

in CDCl3, 100 MHz

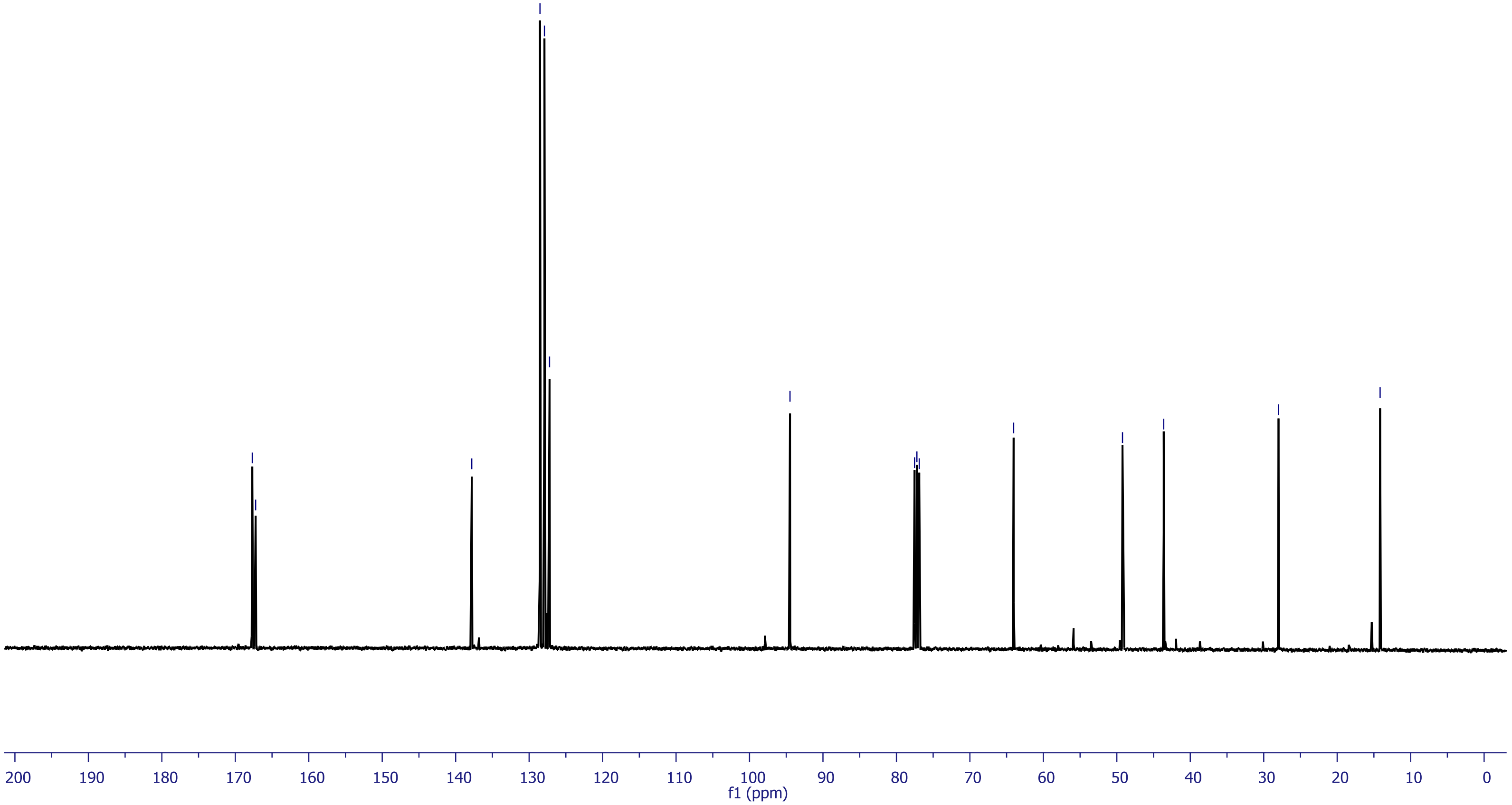

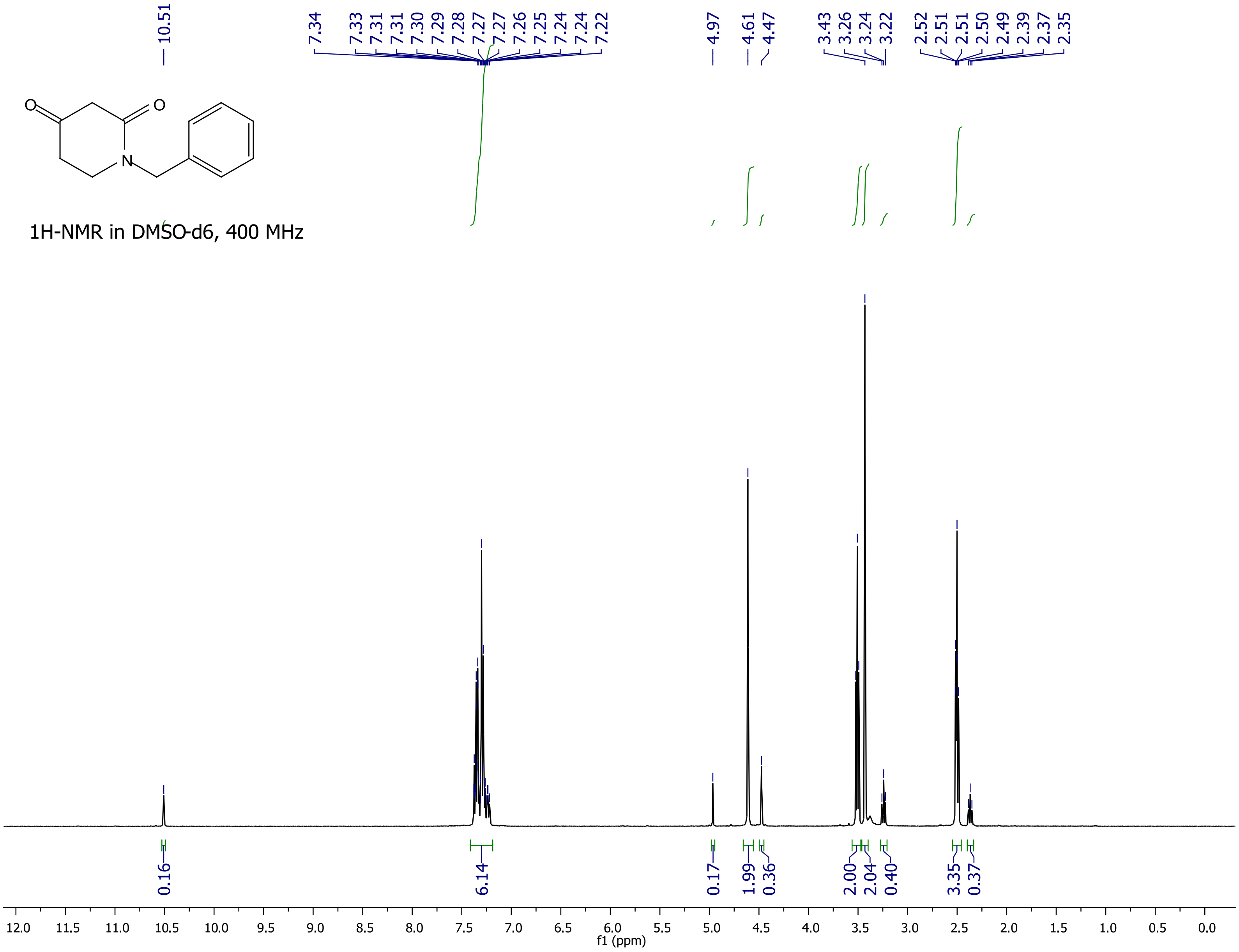

1H-NMR in DMSO-d6, 400 MHz
10.51
7.34 7.33 7.31 7.31 7.30 7.29 7.28 7.27 7.27 7.26 7.25 7.24 7.24 7.22
4.97
4.61
4.47
3.43 3.26 3.24 3.22
2.52 2.51 2.51 2.50 2.49 2.39 2.37 2.35
0.16
6.14
0.17
1.99
0.36
2.00
2.04
0.40
3.35
0.37
f1 (ppm)

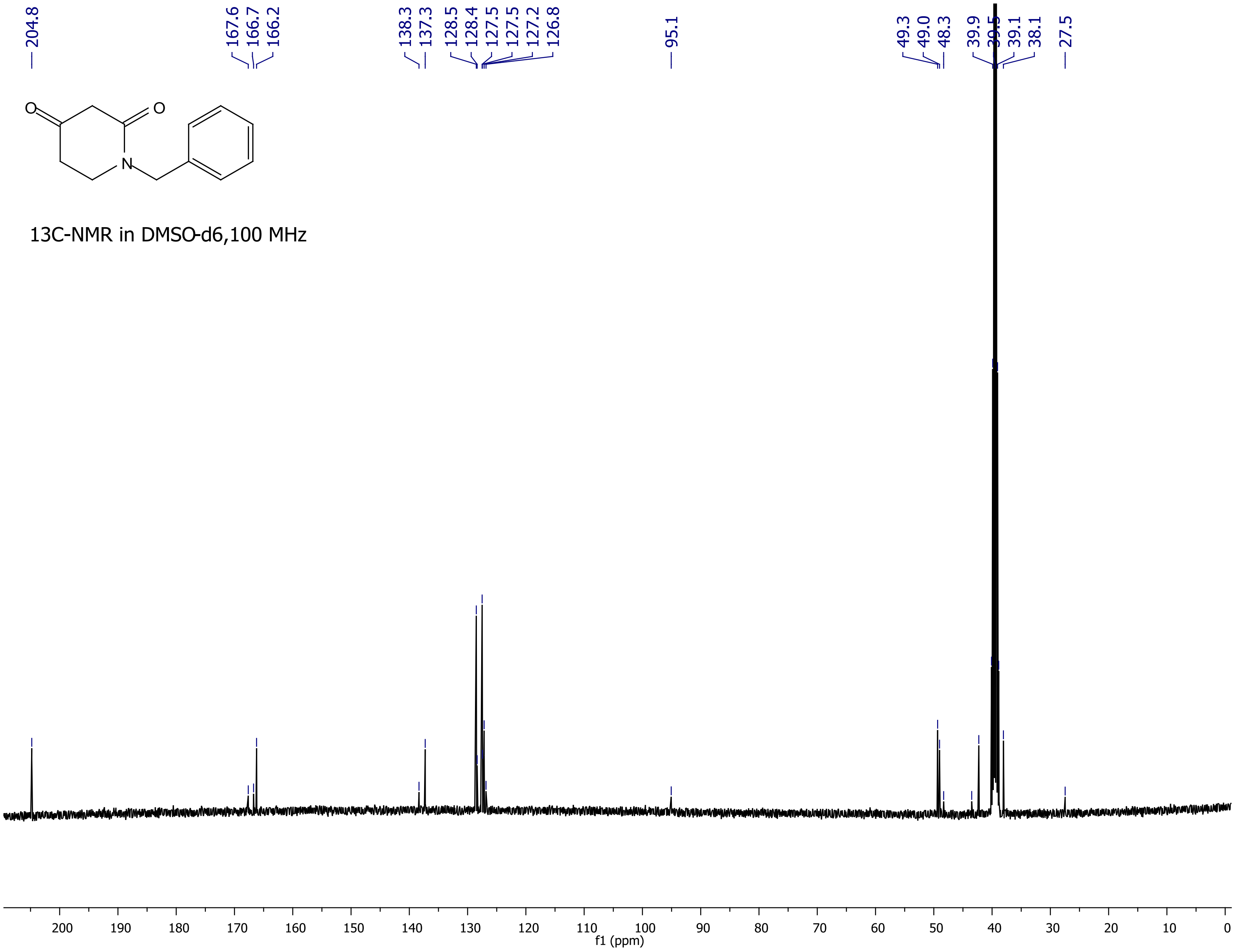
204.8
167.6
166.7
166.2
138.3
137.3
128.5
128.4
127.5
127.5
127.2
126.8
95.1
49.3
49.0
48.3
39.9
39.5
39.1
38.1
27.5
O
O
N
13C-NMR in DMSO-d6,100 MHz
200
190
180
170
160
150
140
130
120
110
100
90
80
70
60
50
40
30
20
10
0
f1 (ppm)

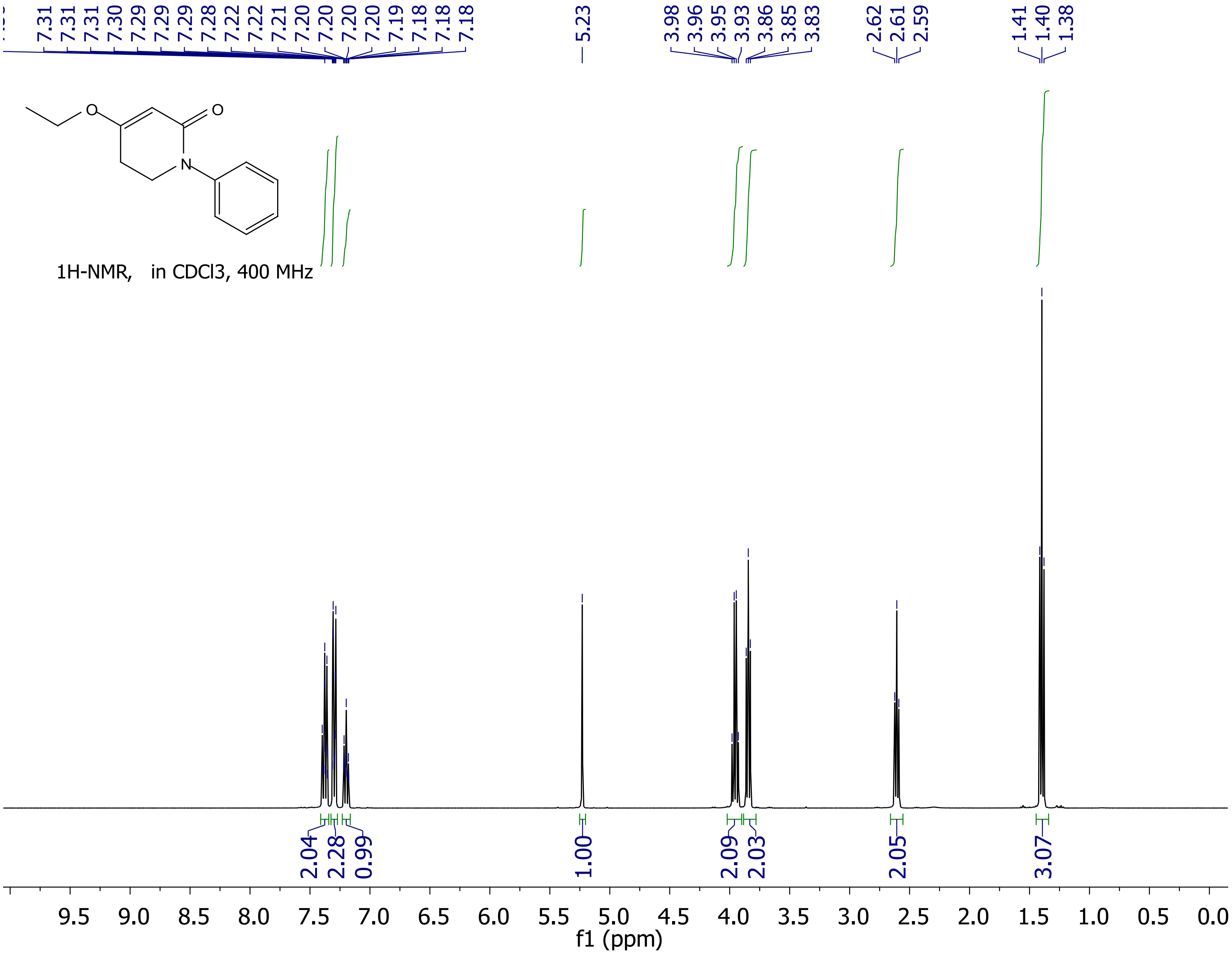
7.31 7.31 7.31 7.30 7.29 7.29 7.29 7.28 7.22 7.22 7.21 7.20 7.20 7.20 7.20 7.19 7.18 7.18 7.18
5.23
3.98 3.96 3.95 3.93 3.86 3.85 3.83
2.62 2.61 2.59
1.41 1.40 1.38
O
O
N
1H-NMR, in CDCl3, 400 MHz
2.04 2.28 0.99
1.00
2.09 2.03
2.05
3.07
9.5 9.0 8.5 8.0 7.5 7.0 6.5 6.0 5.5 5.0 4.5 4.0 3.5 3.0 2.5 2.0 1.5 1.0 0.5 0.0
f1 (ppm)

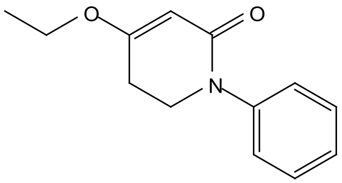

13C-NMR, in CDCl3, 100 MHz

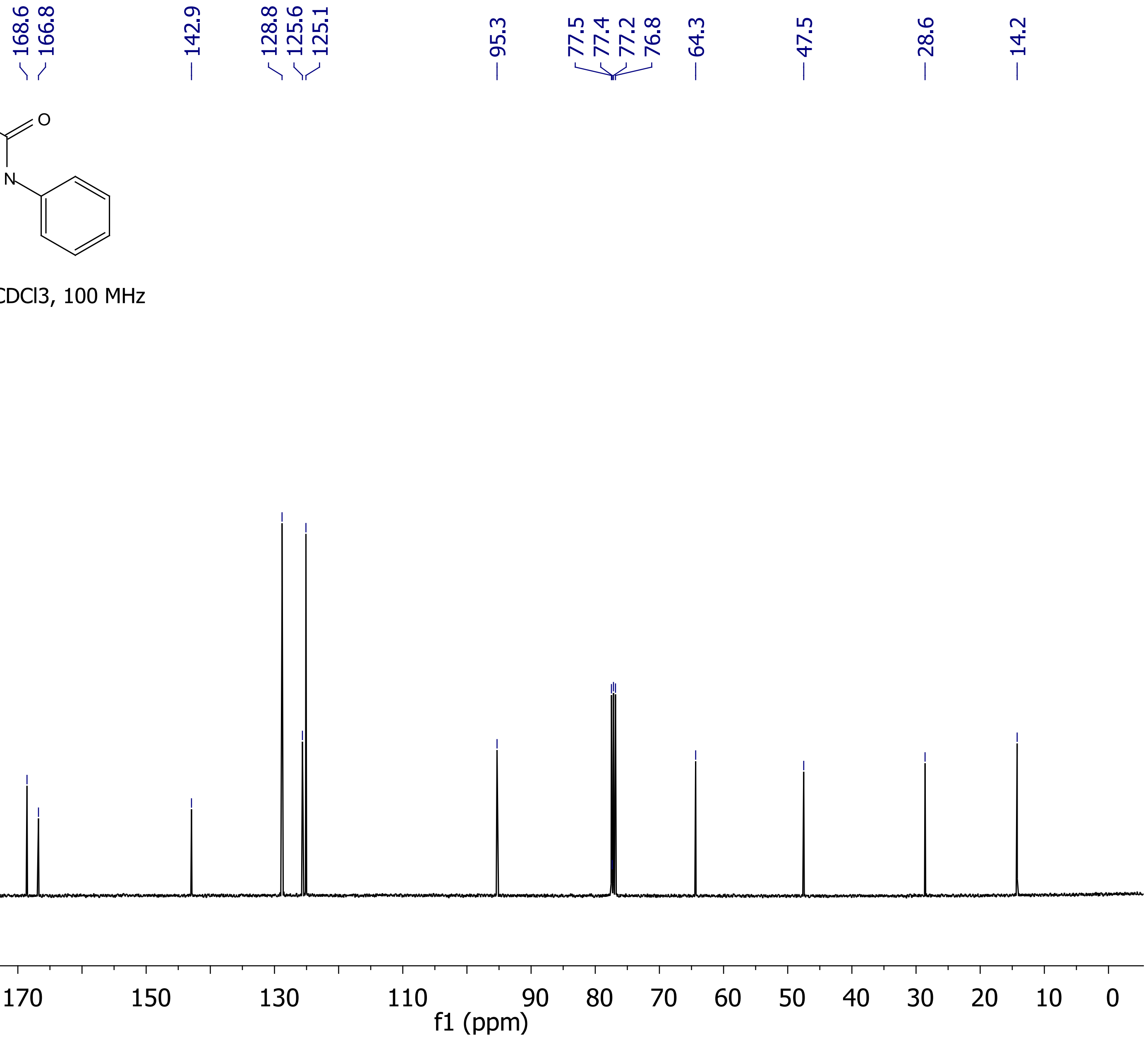

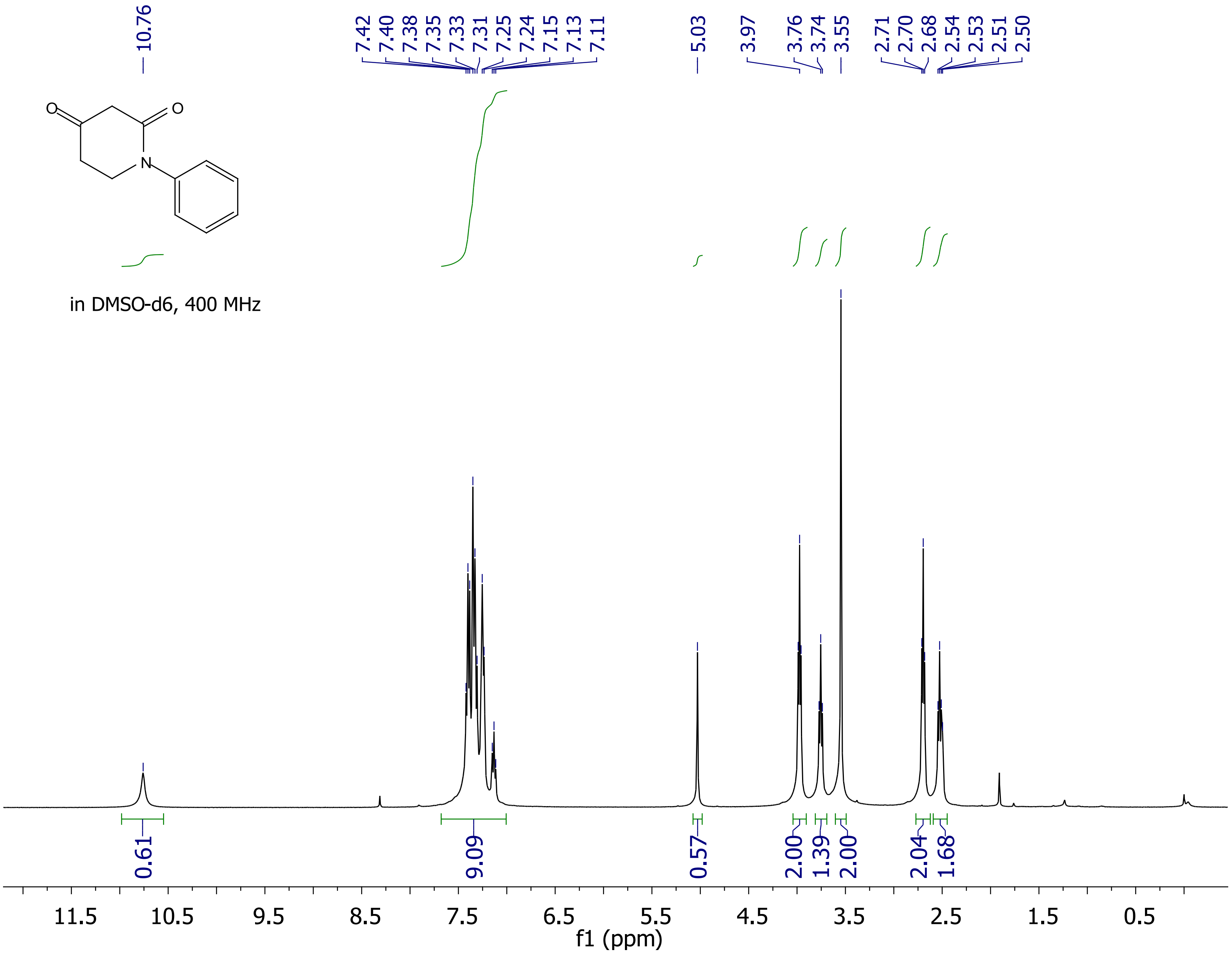
10.76
7.42 7.40 7.38 7.35 7.33 7.31 7.25 7.24 7.15 7.13 7.11
5.03
3.97 3.76 3.74 3.55
2.71 2.70 2.68 2.54 2.53 2.51 2.50
O
O
N
in DMSO-d6, 400 MHz
0.61
9.09
0.57
2.00
1.39
2.00
2.04
1.68
11.5 10.5 9.5 8.5 7.5 6.5 5.5 4.5 3.5 2.5 1.5 0.5
f1 (ppm)

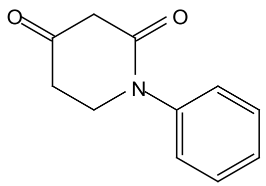

in DMSO-d6, 100 MHz

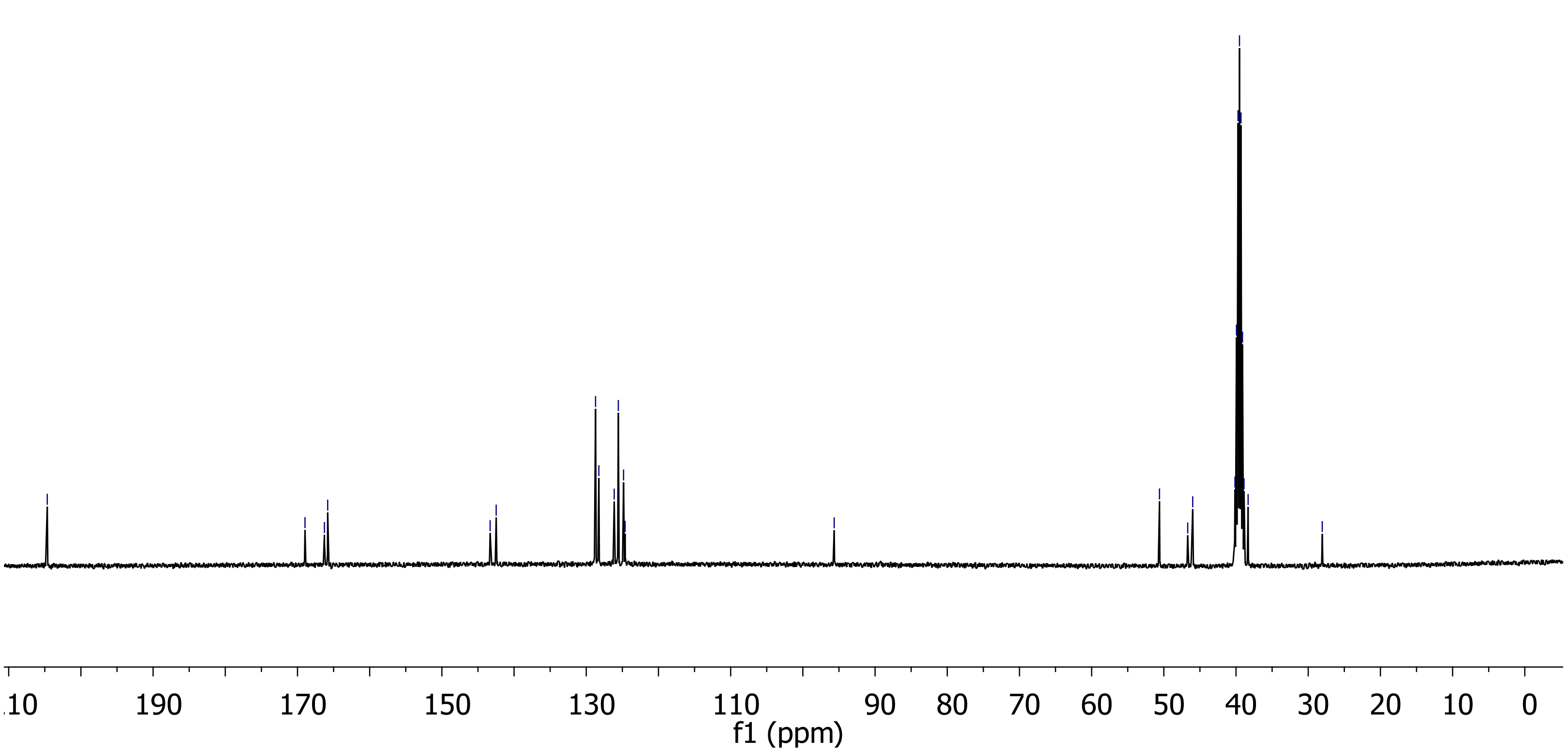

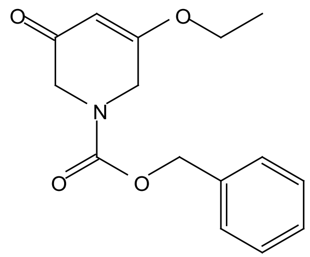

1H-NMR in CDCl3, 400 MHz

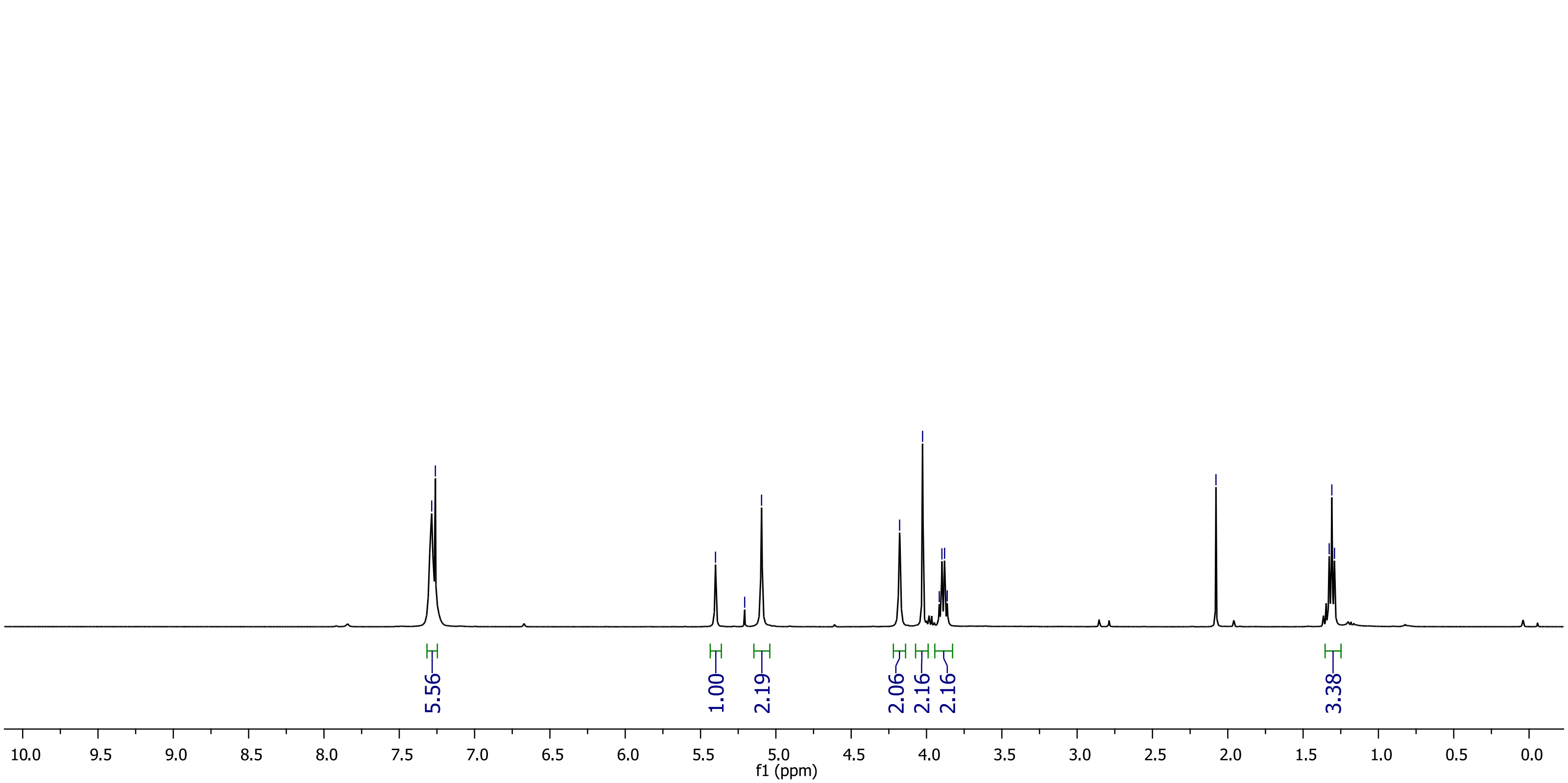

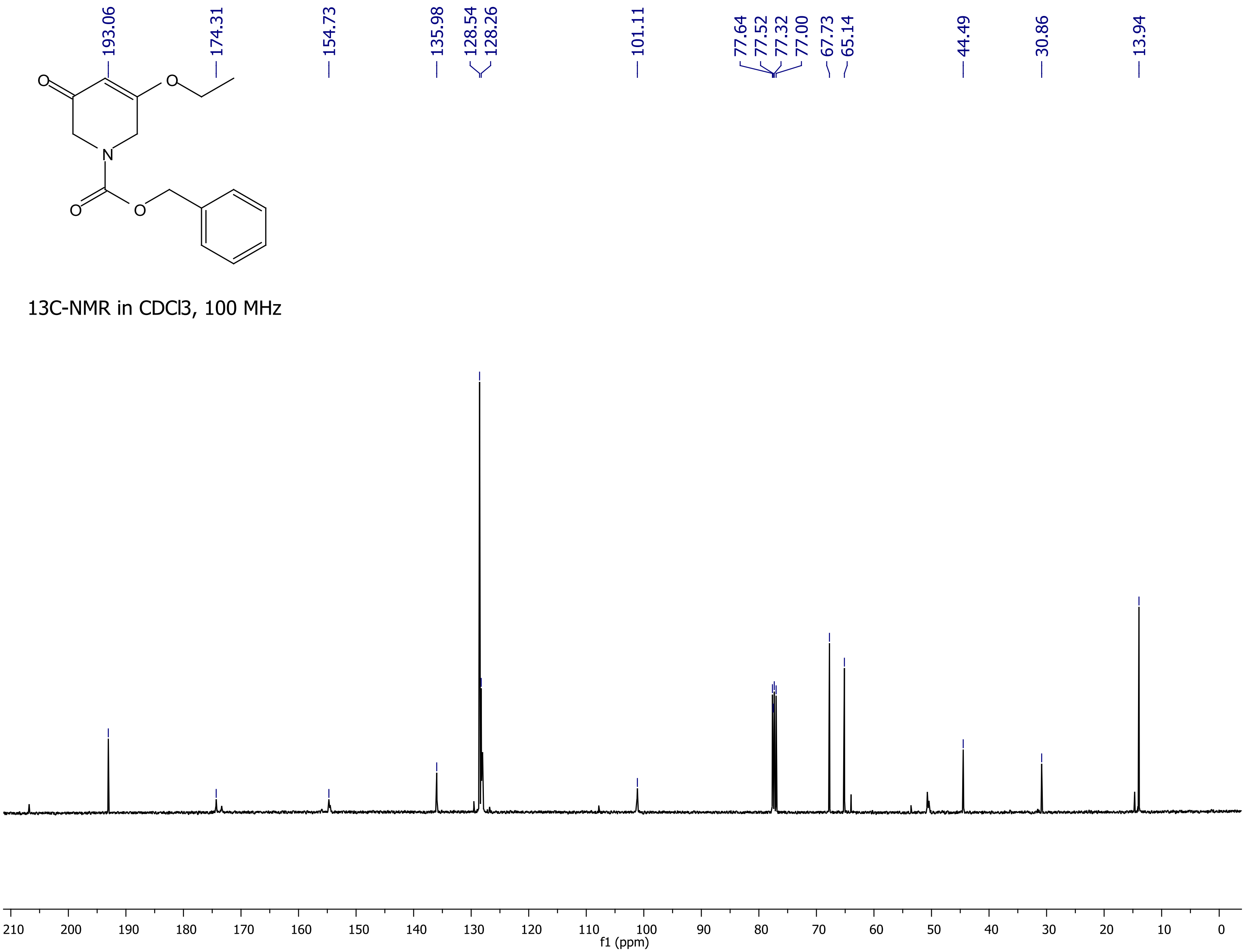

13C-NMR in CDCl3, 100 MHz

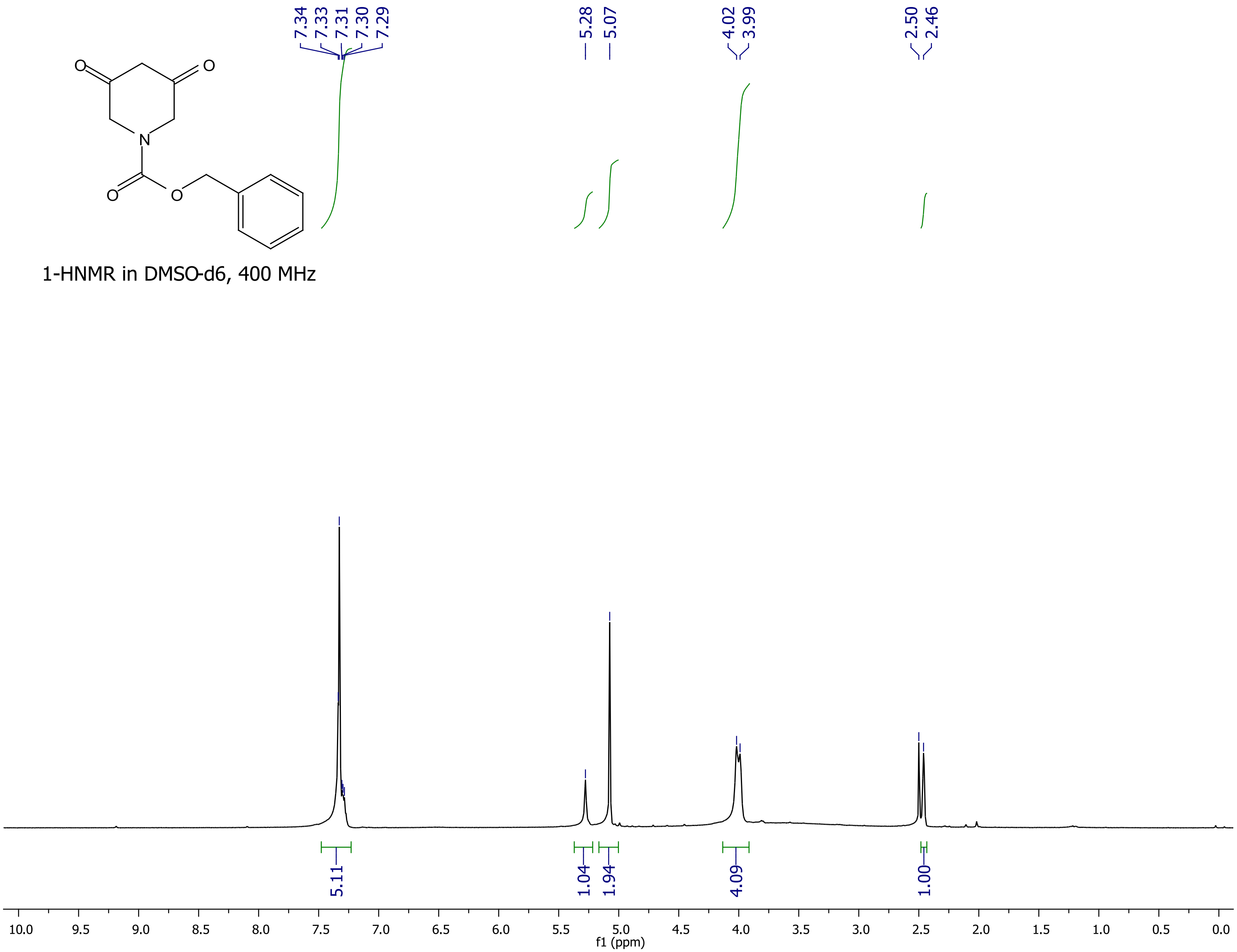

1-HNMR in DMSO-d6, 400 MHz

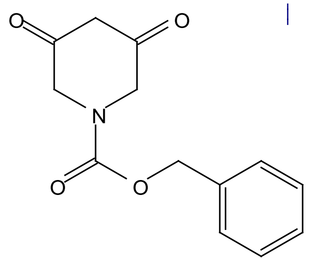

13-CNMR in DMSO-d6, 100 MHz

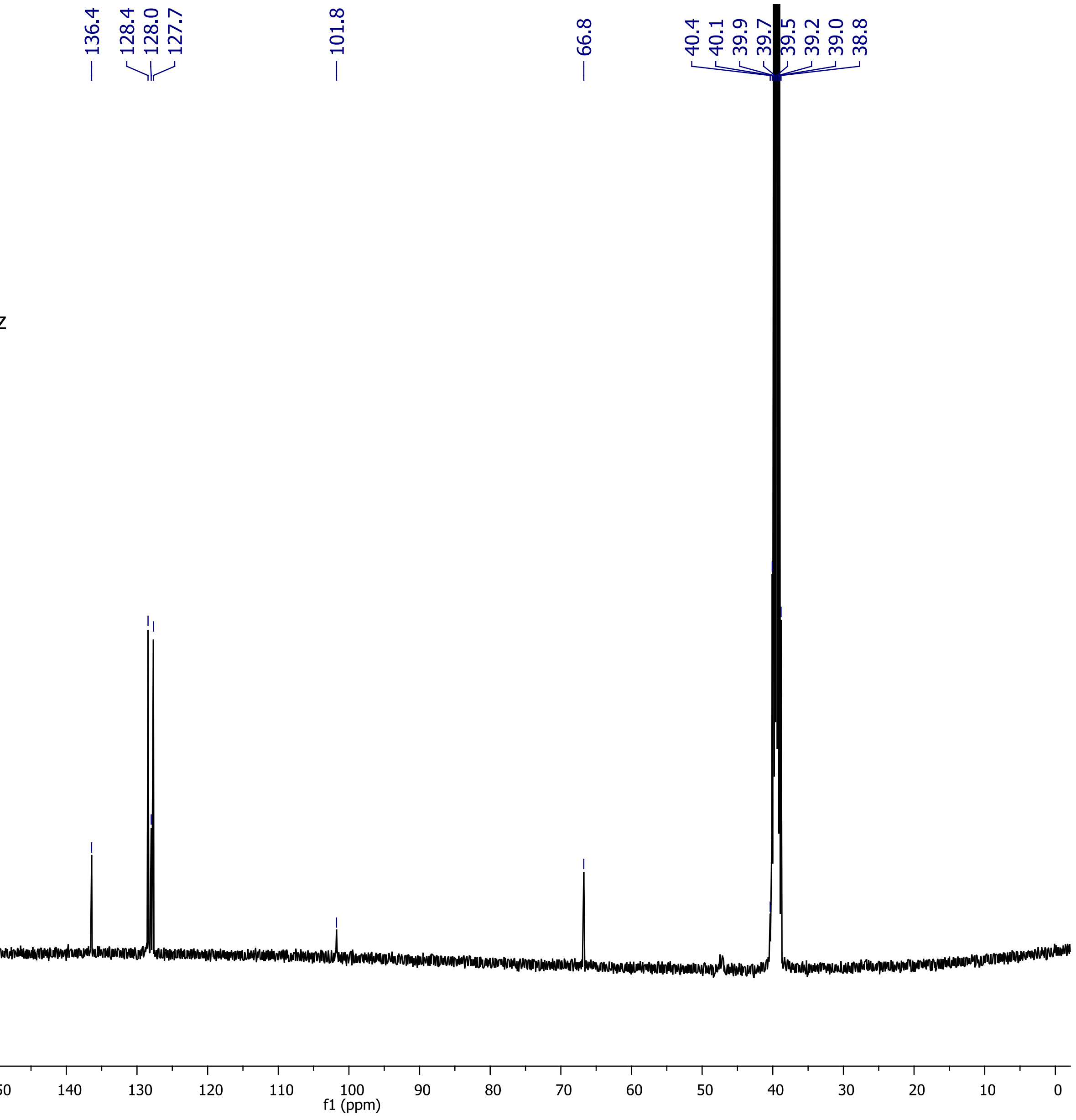

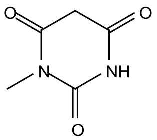

1H-NMR in DMSO-d6, 400 MHz

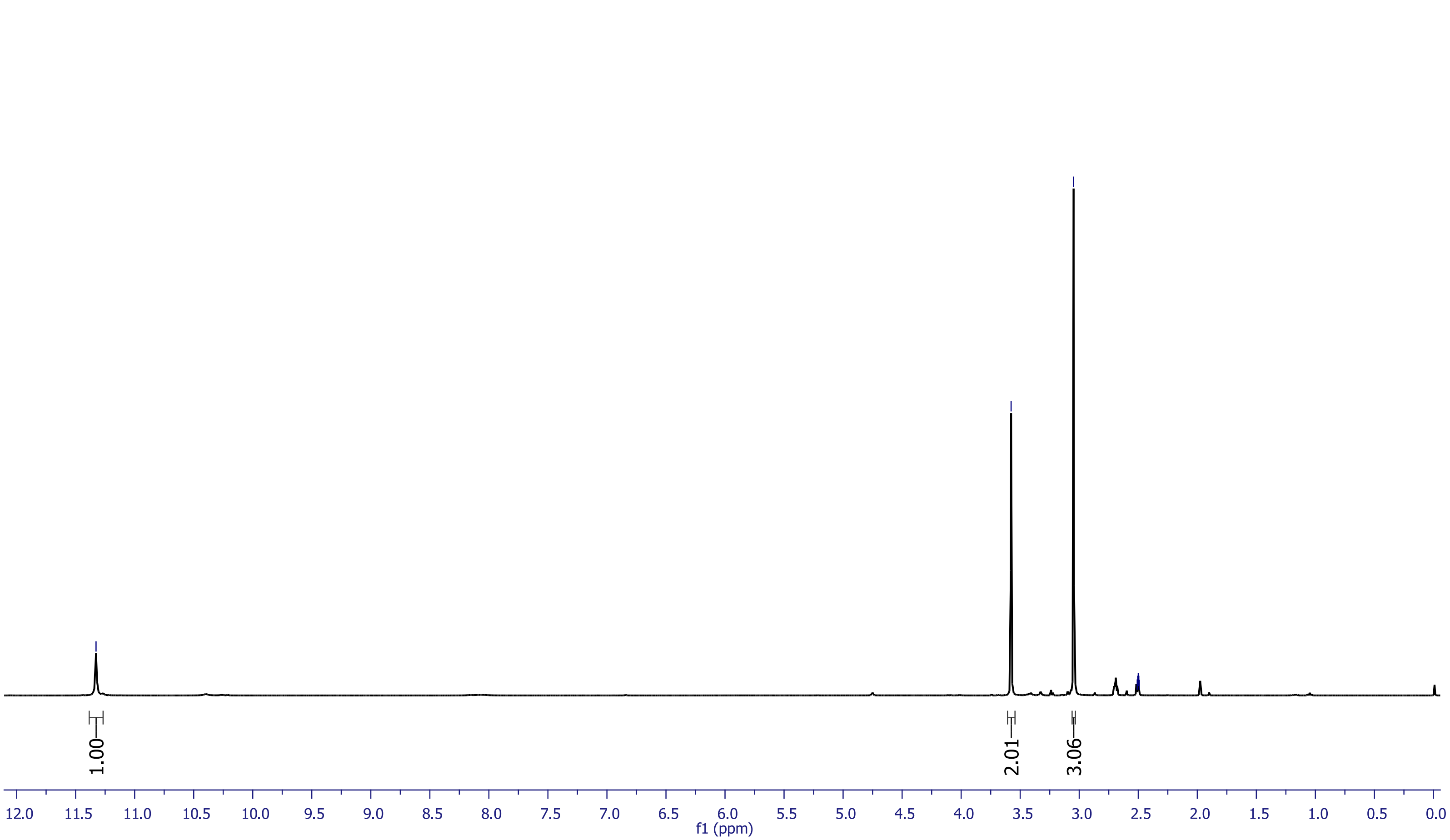

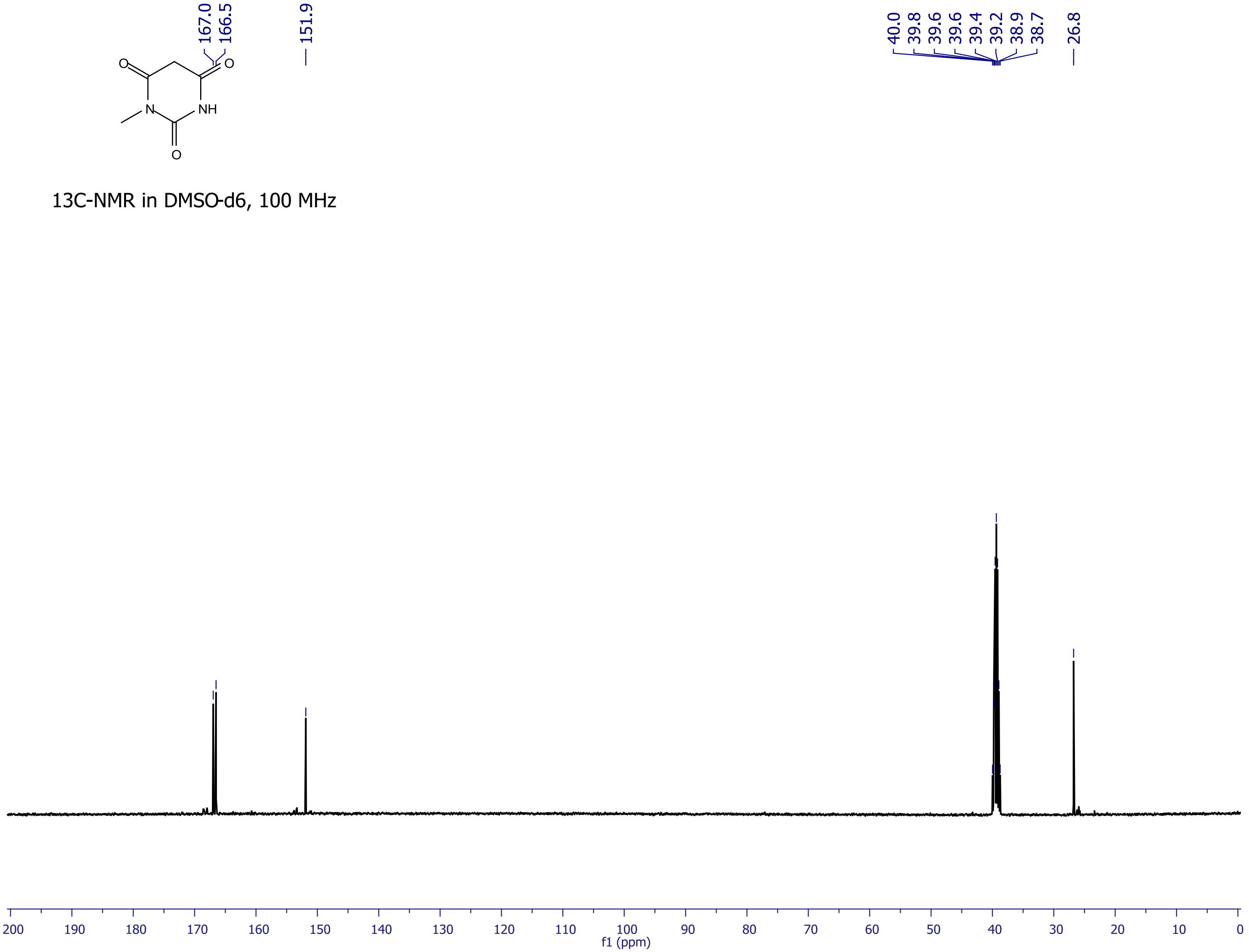
13C-NMR in DMSO-d6, 100 MHz
167.0
166.5
151.9
40.0
39.8
39.6
39.6
39.4
39.2
38.9
38.7
26.8
200
190
180
170
160
150
140
130
120
110
100
90
80
70
60
50
40
30
20
10
0
f1 (ppm)

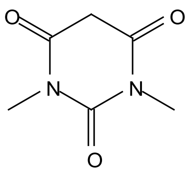

1H-NMR in DMSO-d6, 400 MHz

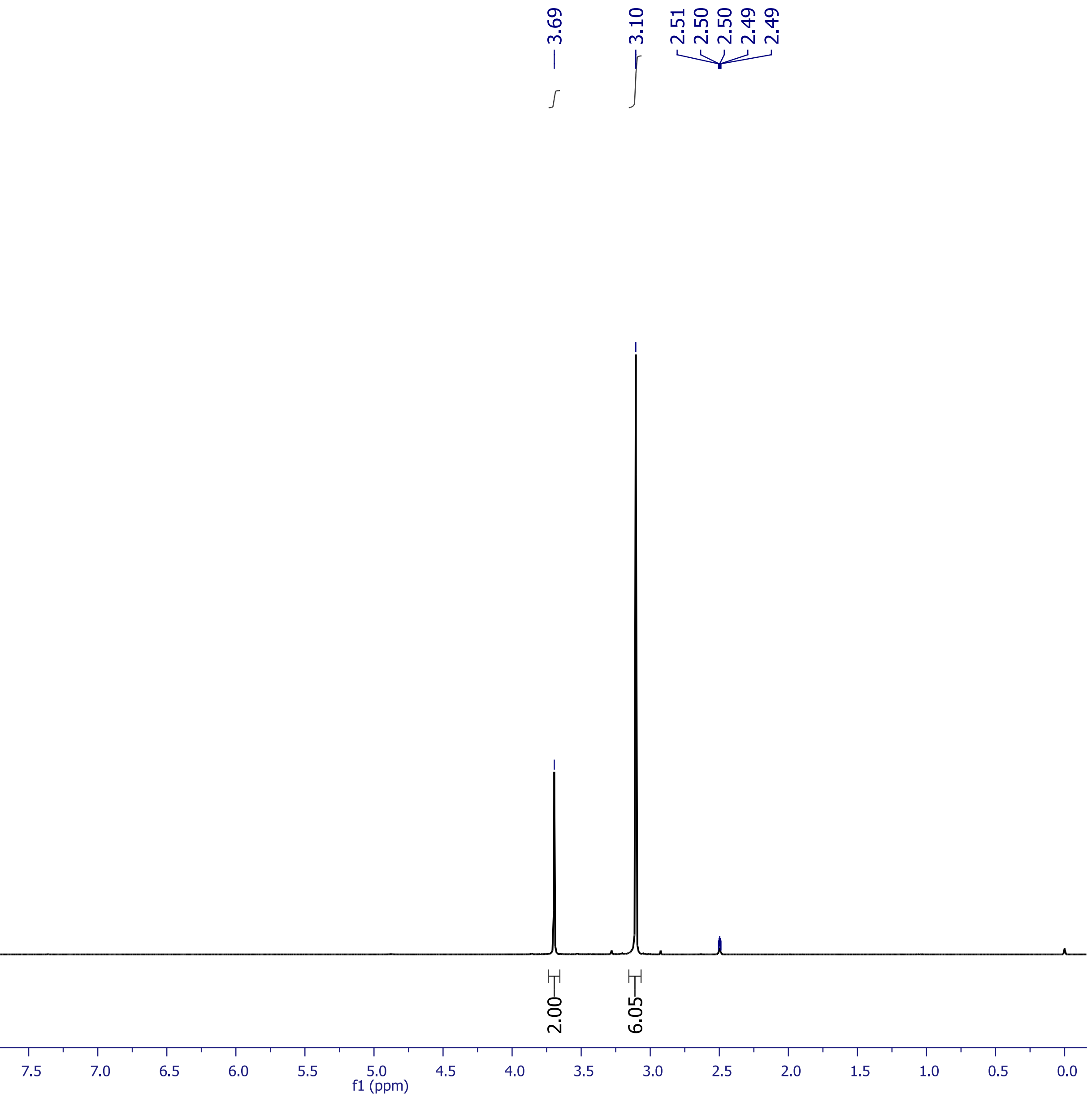

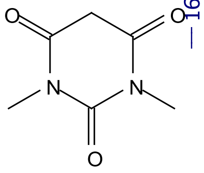

13C-NMR in DMSO-d6, 100 MHz

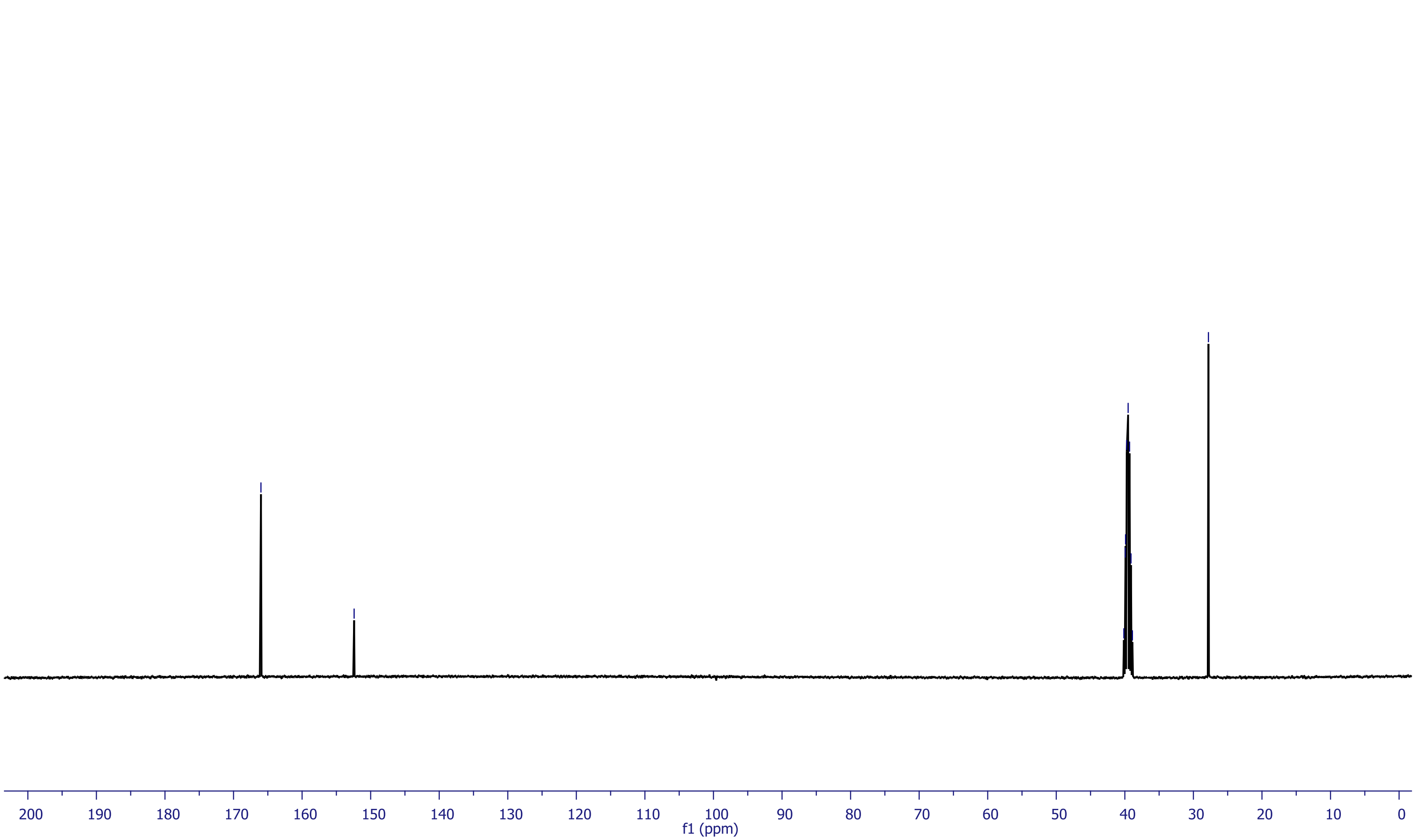

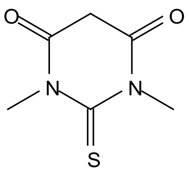

1H-NMR, in DMSO-d6, 400 MHz

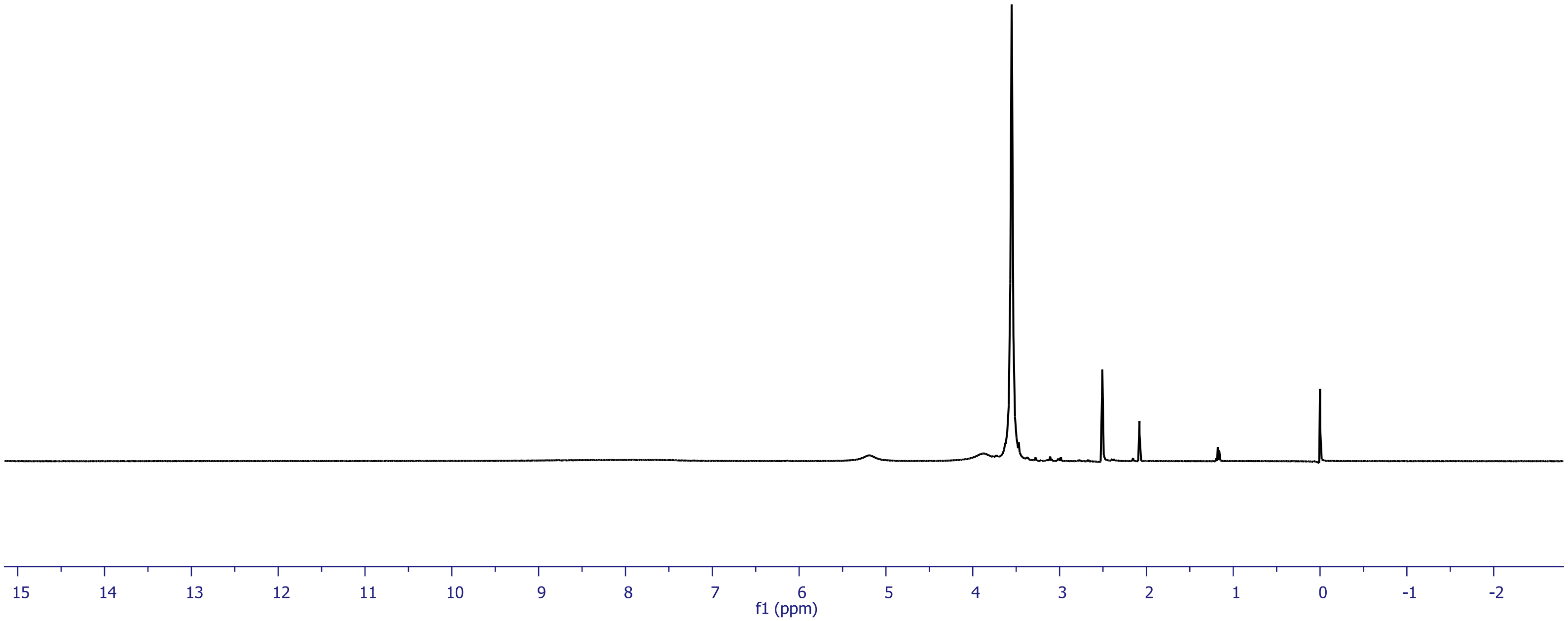

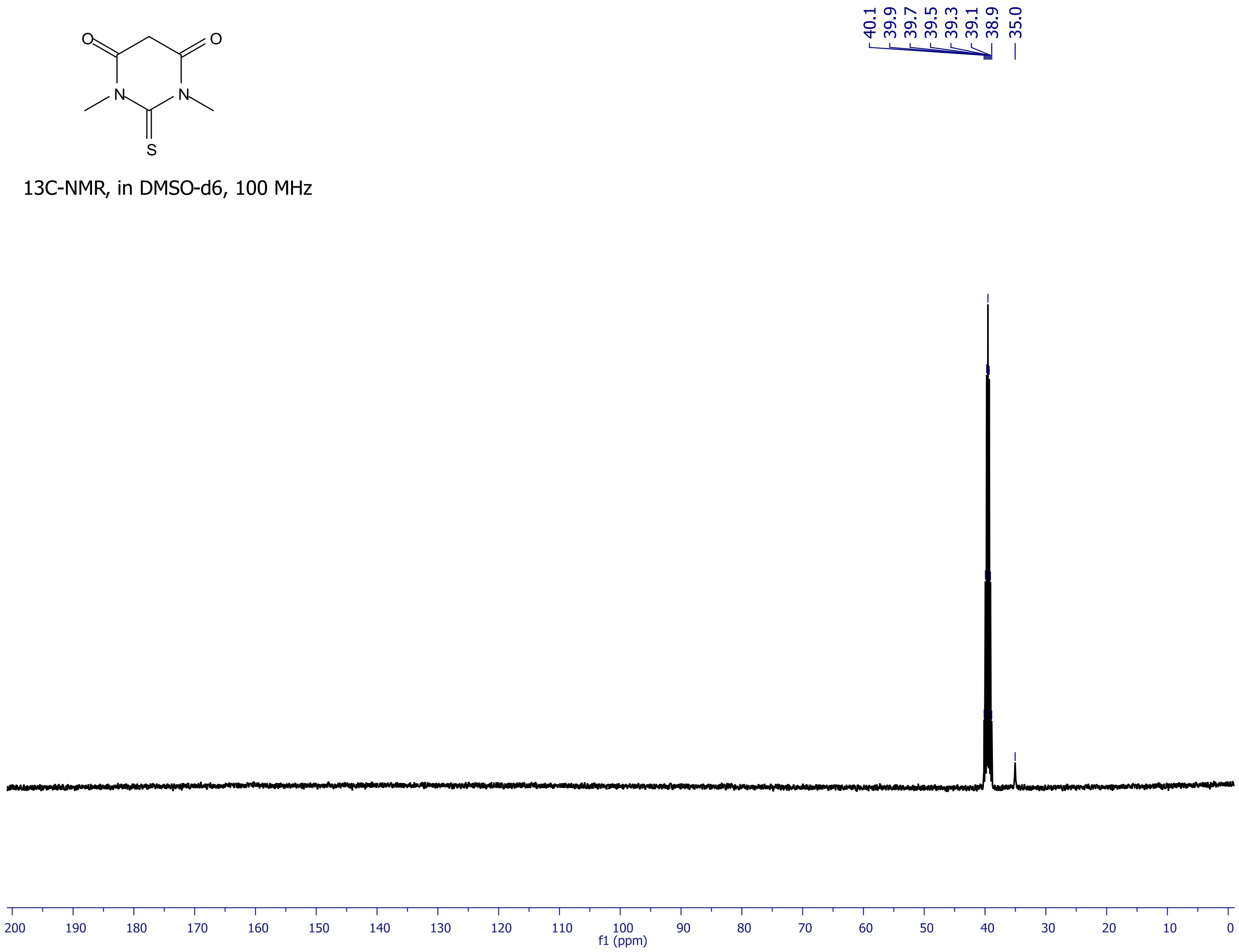

13C-NMR, in DMSO-d6, 100 MHz

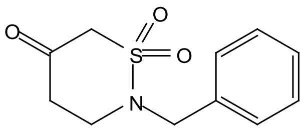

1H-NMR, in CDCl3, 400 MHz

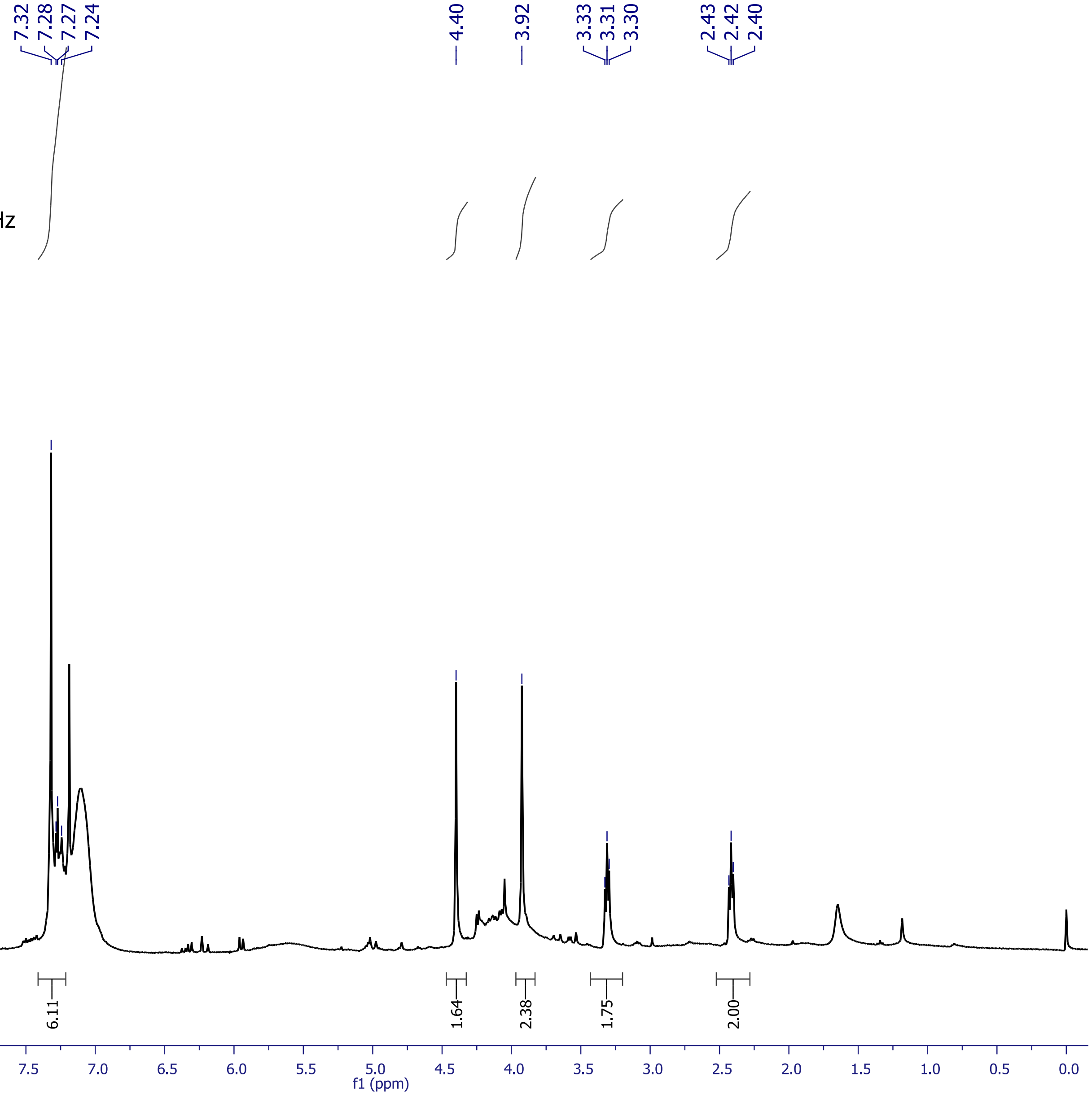

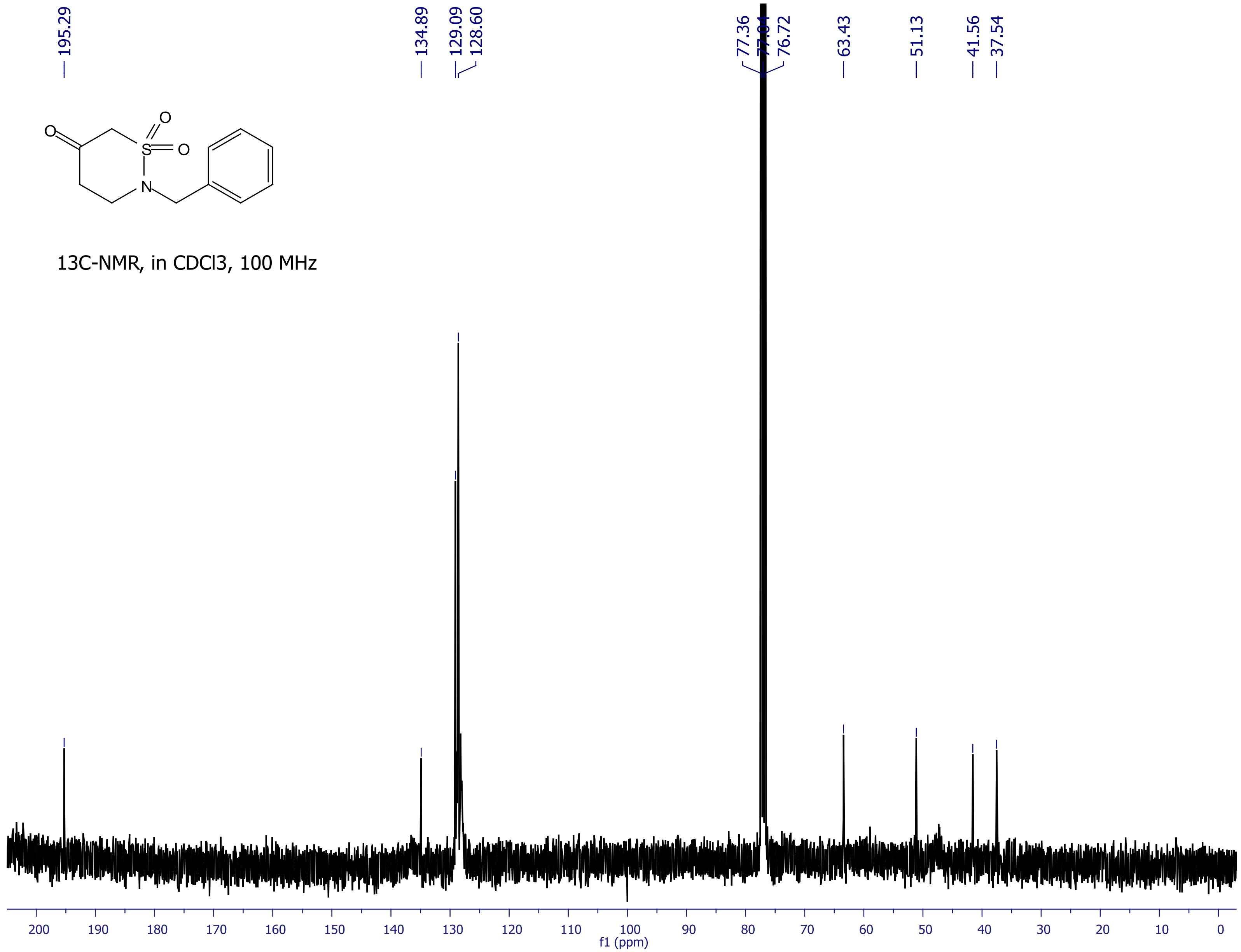
195.29
134.89
129.09
128.60
77.36
76.72
63.43
51.13
41.56
37.54
13C-NMR, in CDCl3, 100 MHz
200 190 180 170 160 150 140 130 120 110 100 90 80 70 60 50 40 30 20 10 0
f1 (ppm)

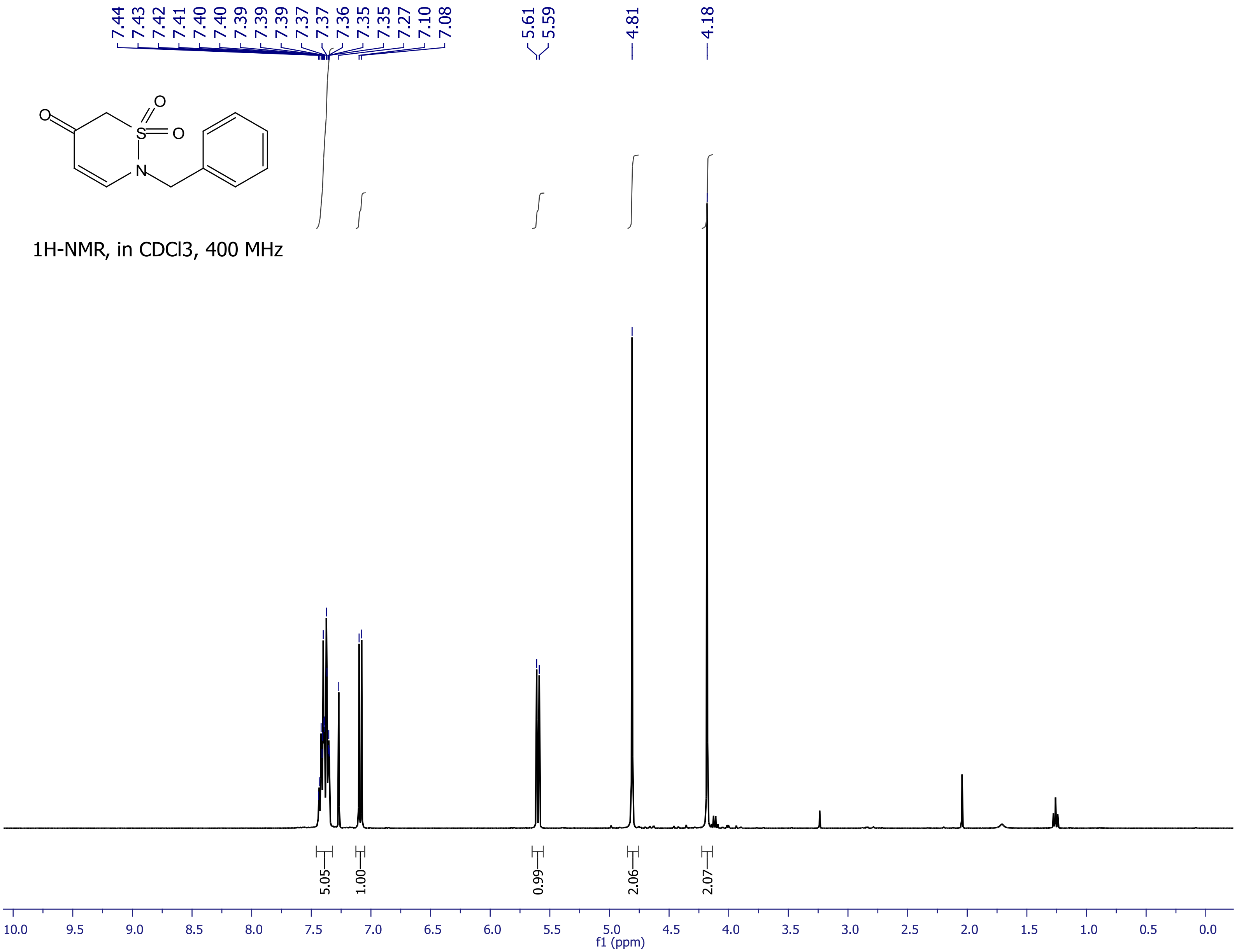

1H-NMR, in CDCl3, 400 MHz

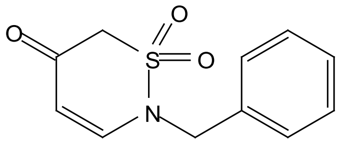

13C-NMR, in CDCl3, 100 MHz

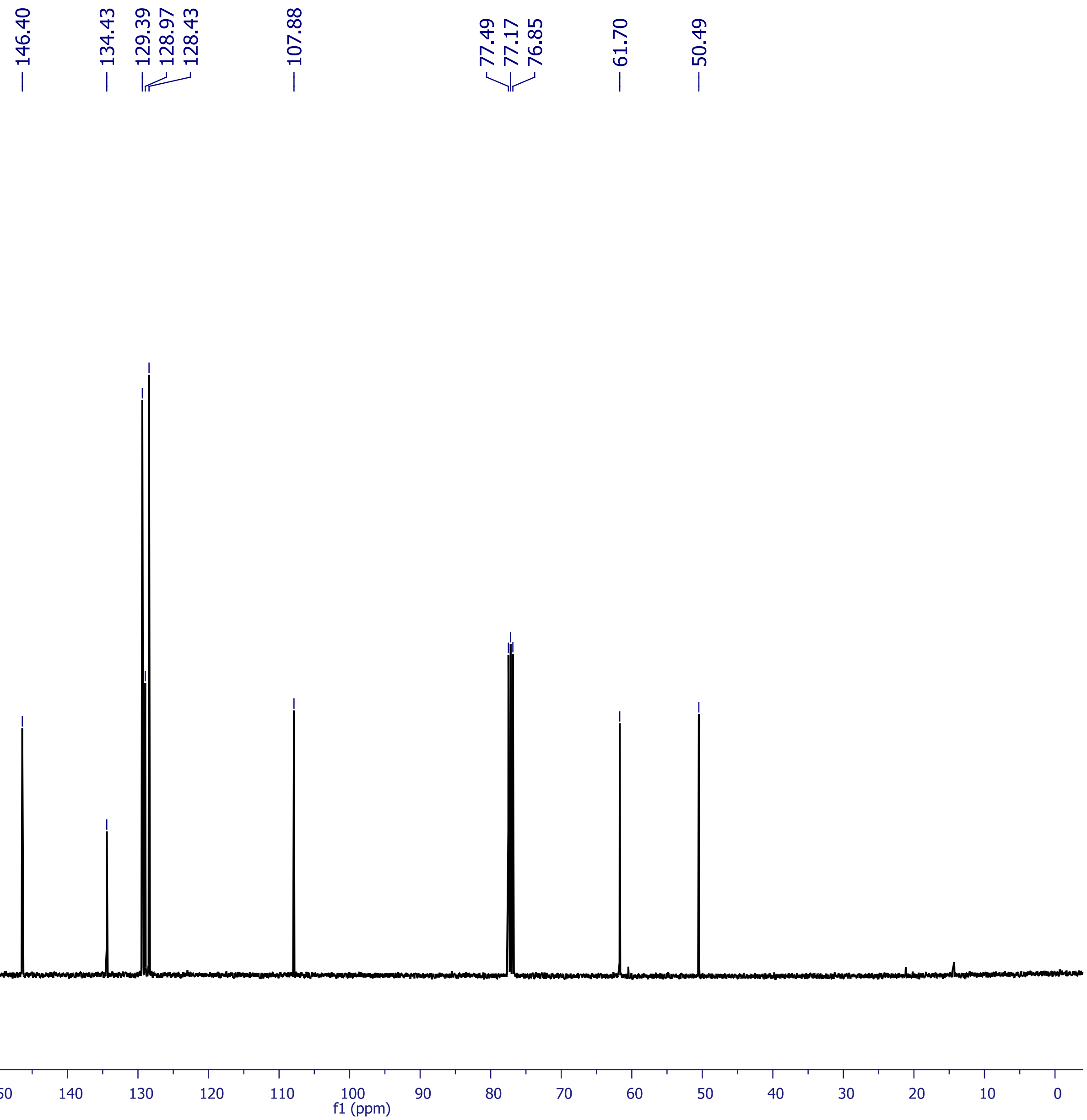

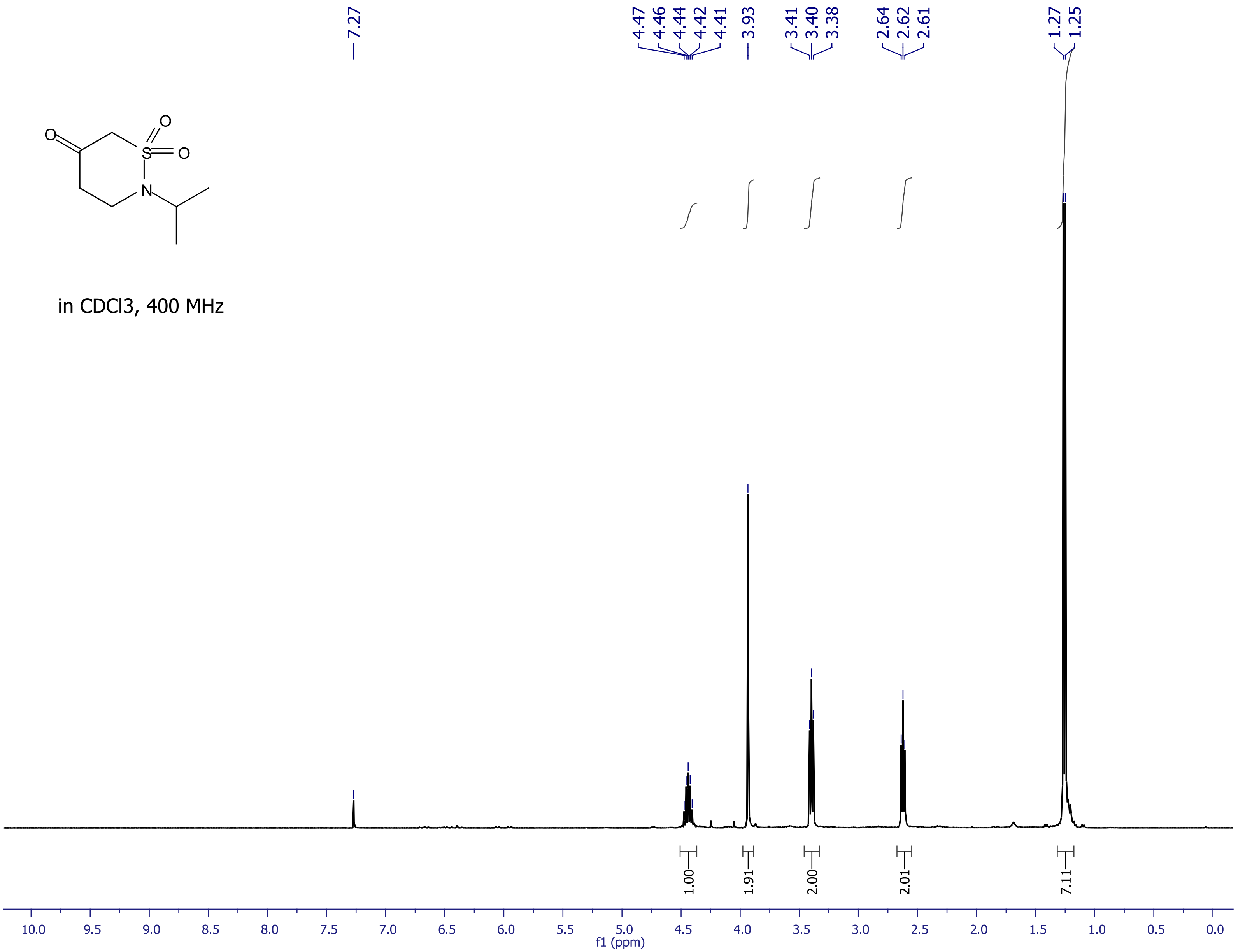
in CDCl3, 400 MHz
7.27
4.47
4.46
4.44
4.42
4.41
3.93
3.41
3.40
3.38
2.64
2.62
2.61
1.27
1.25
1.00
1.91
2.00
2.01
7.11
10.0 9.5 9.0 8.5 8.0 7.5 7.0 6.5 6.0 5.5 5.0 4.5 4.0 3.5 3.0 2.5 2.0 1.5 1.0 0.5 0.0
f1 (ppm)

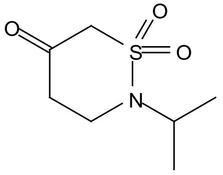

in CDCl3, 100 MHz

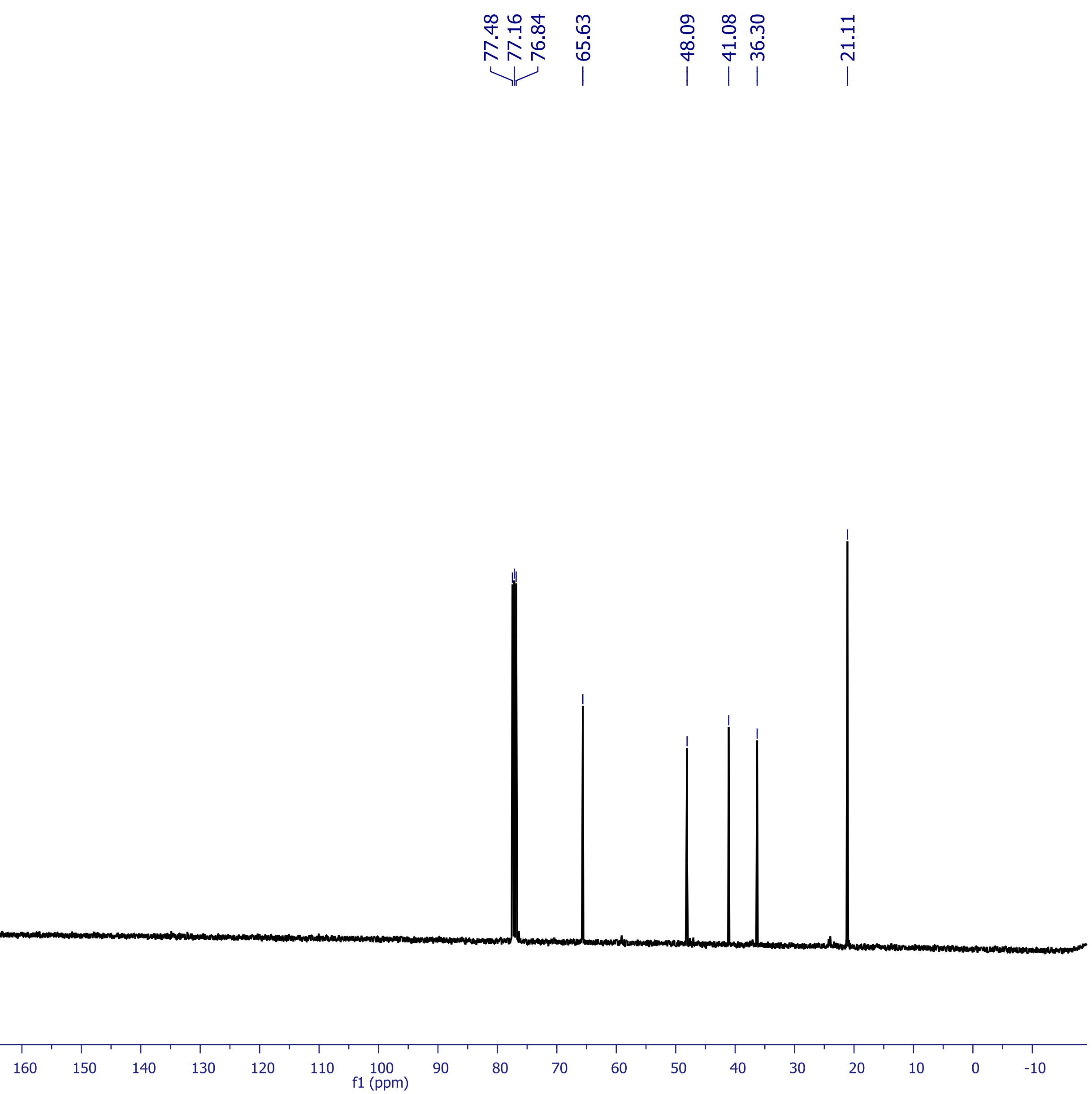

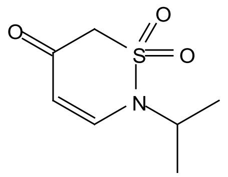

1H-NMR, in CDCl3, 400 MHz

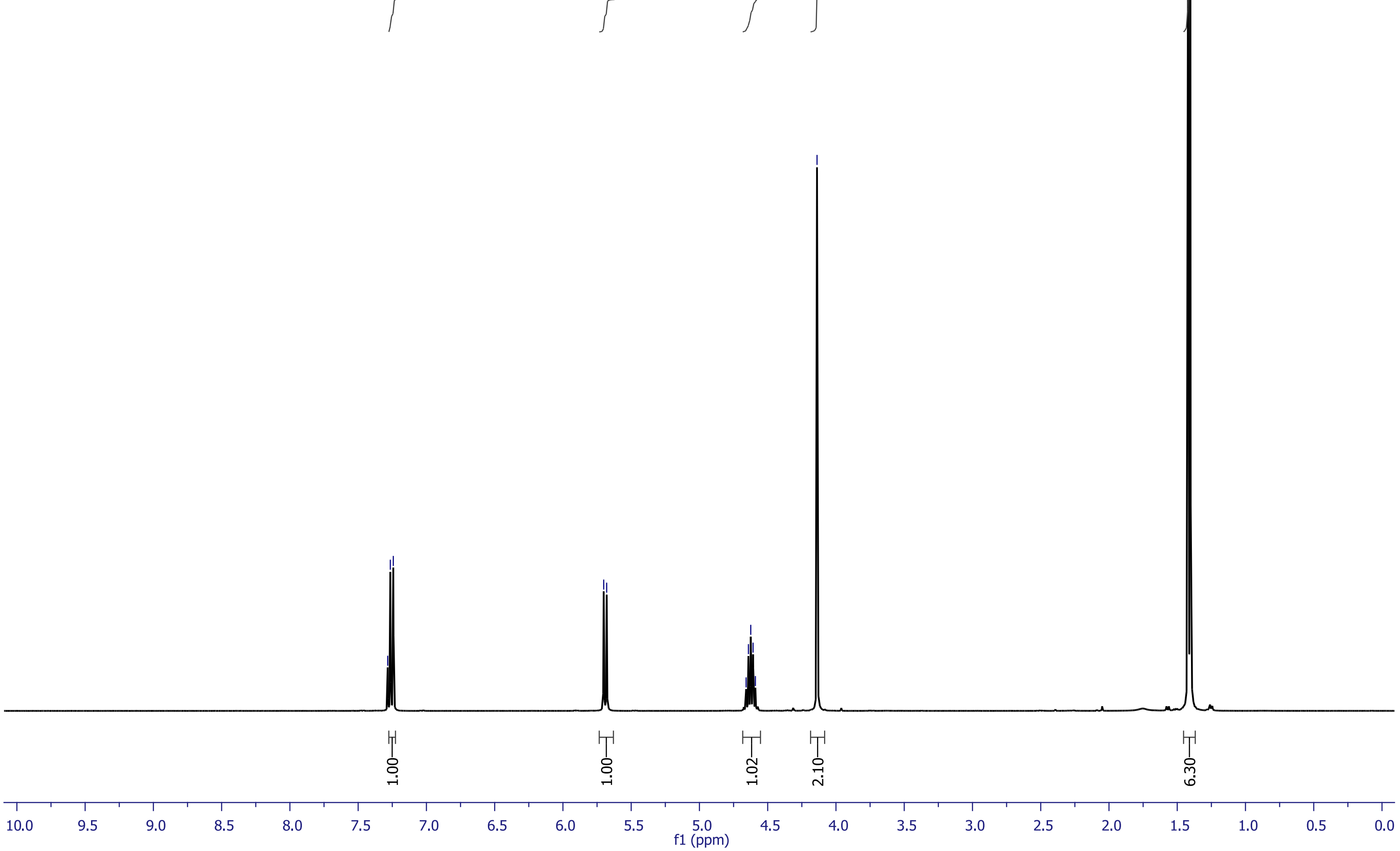

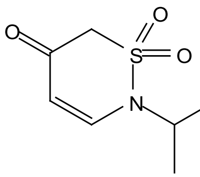

13C-NMR, in CDCl3, 100 MHz

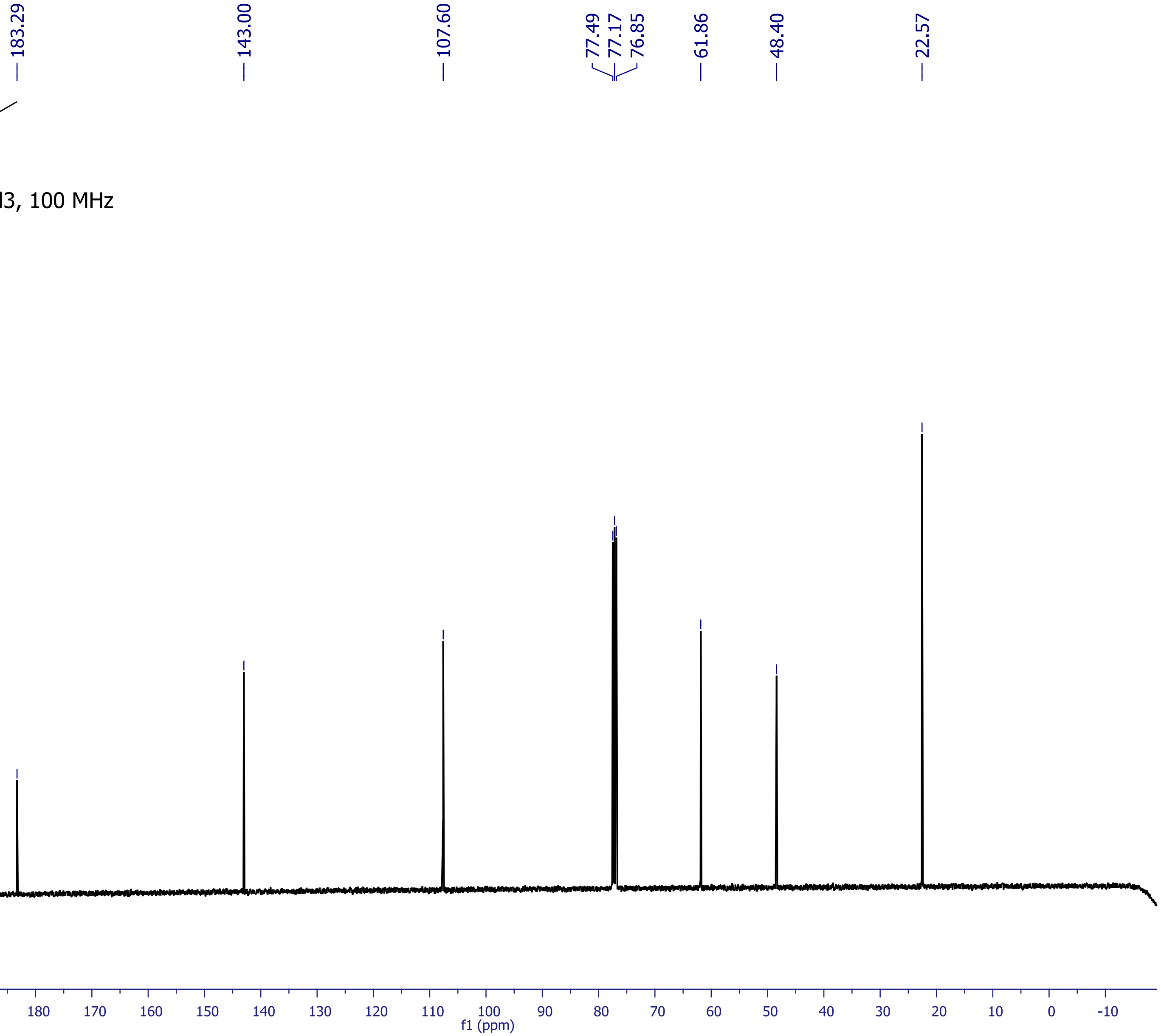

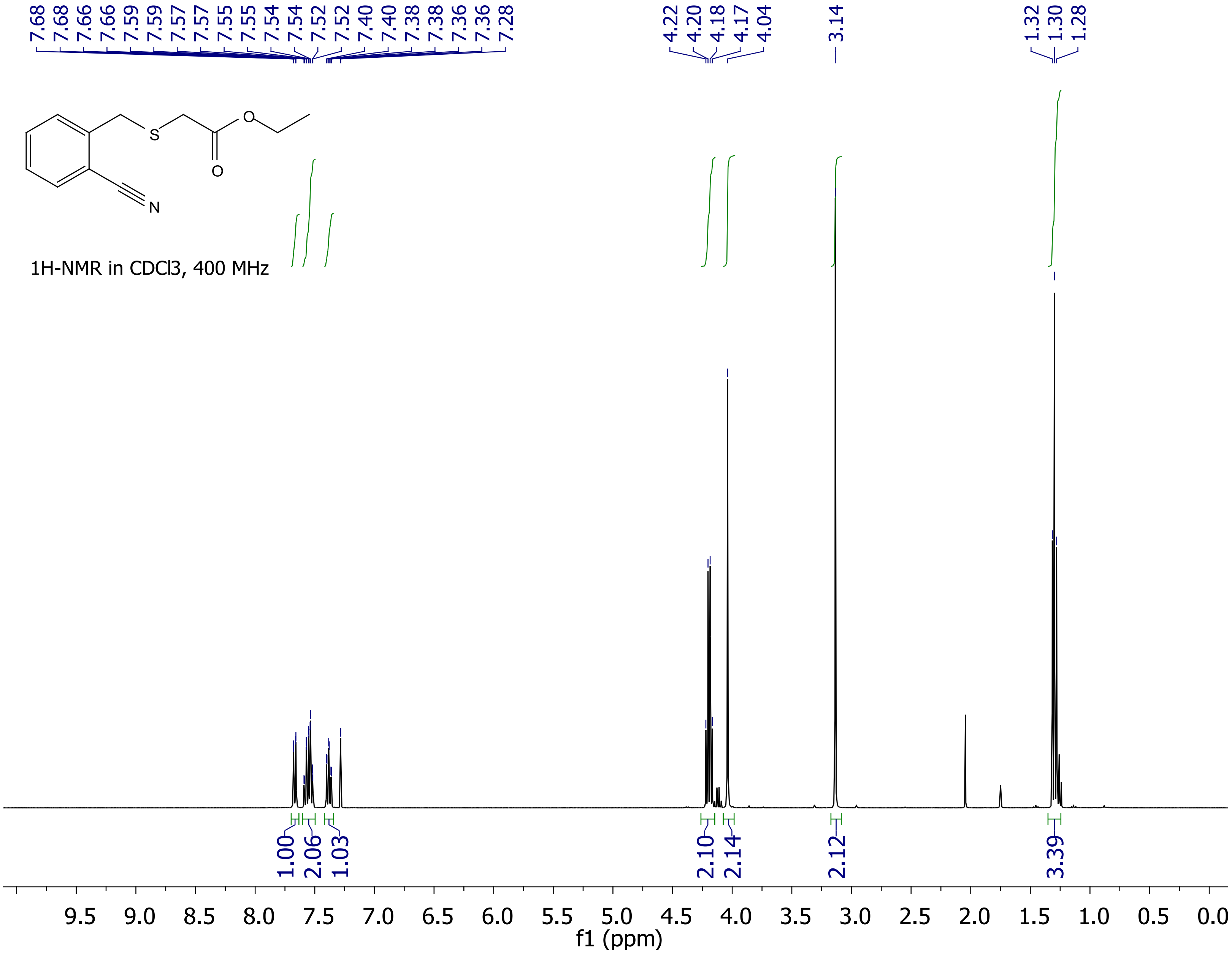

7.68
7.68
7.66
7.66
7.59
7.59
7.57
7.57
7.55
7.55
7.54
7.54
7.52
7.52
7.40
7.40
7.38
7.38
7.36
7.36
7.28
4.22
4.20
4.18
4.17
4.04
3.14
1.32
1.30
1.28
S
O
O
N
1H-NMR in CDCl3, 400 MHz
1.00
2.06
1.03
2.10
2.14
2.12
3.39
9.5
9.0
8.5
8.0
7.5
7.0
6.5
6.0
5.5
5.0
4.5
4.0
3.5
3.0
2.5
2.0
1.5
1.0
0.5
0.0
f1 (ppm)

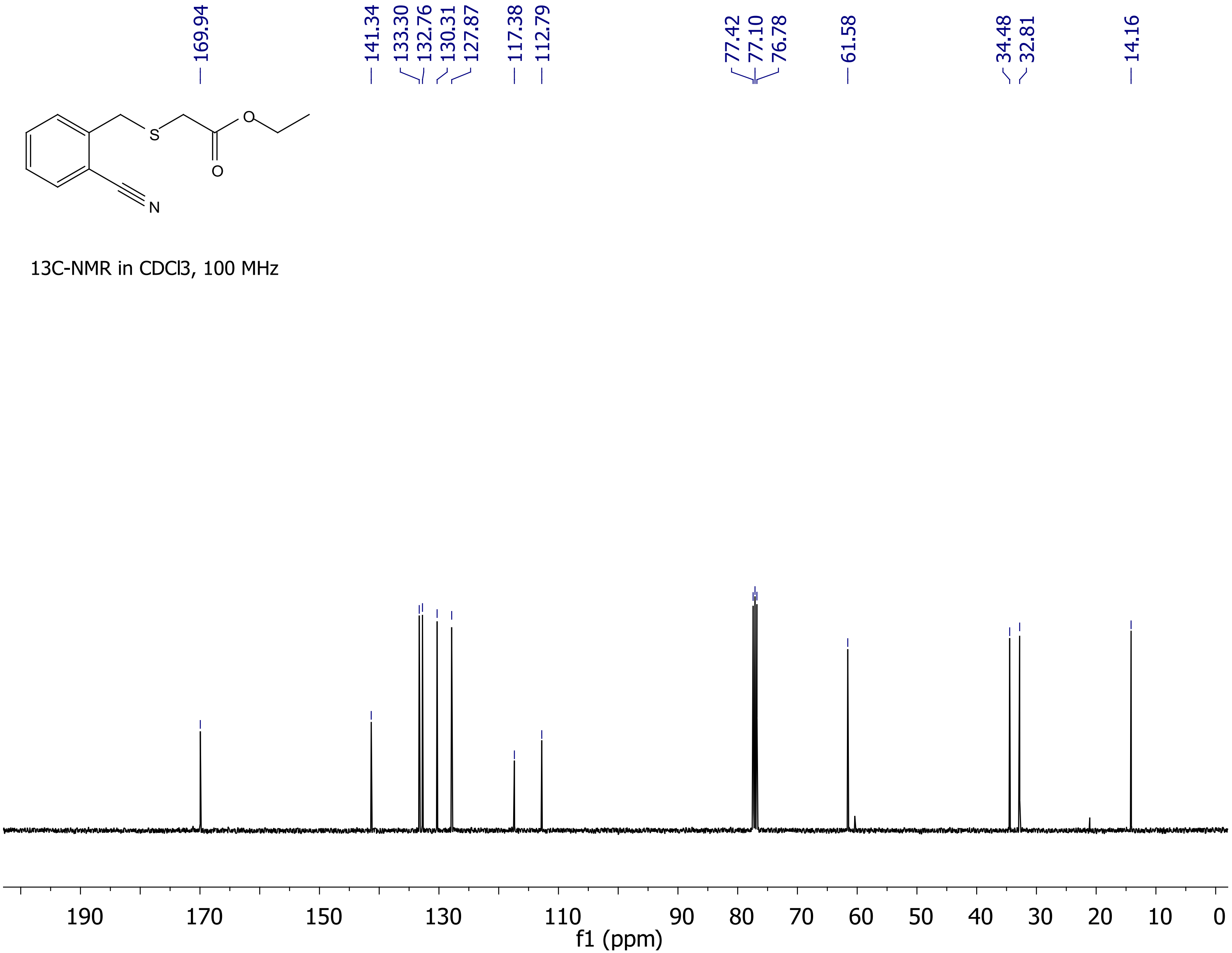
169.94
141.34
133.30
132.76
130.31
127.87
117.38
112.79
77.42
77.10
76.78
61.58
34.48
32.81
14.16
S
O
O
N
13C-NMR in CDCl3, 100 MHz
190
170
150
130
110
90
80
70
60
50
40
30
20
10
0
f1 (ppm)

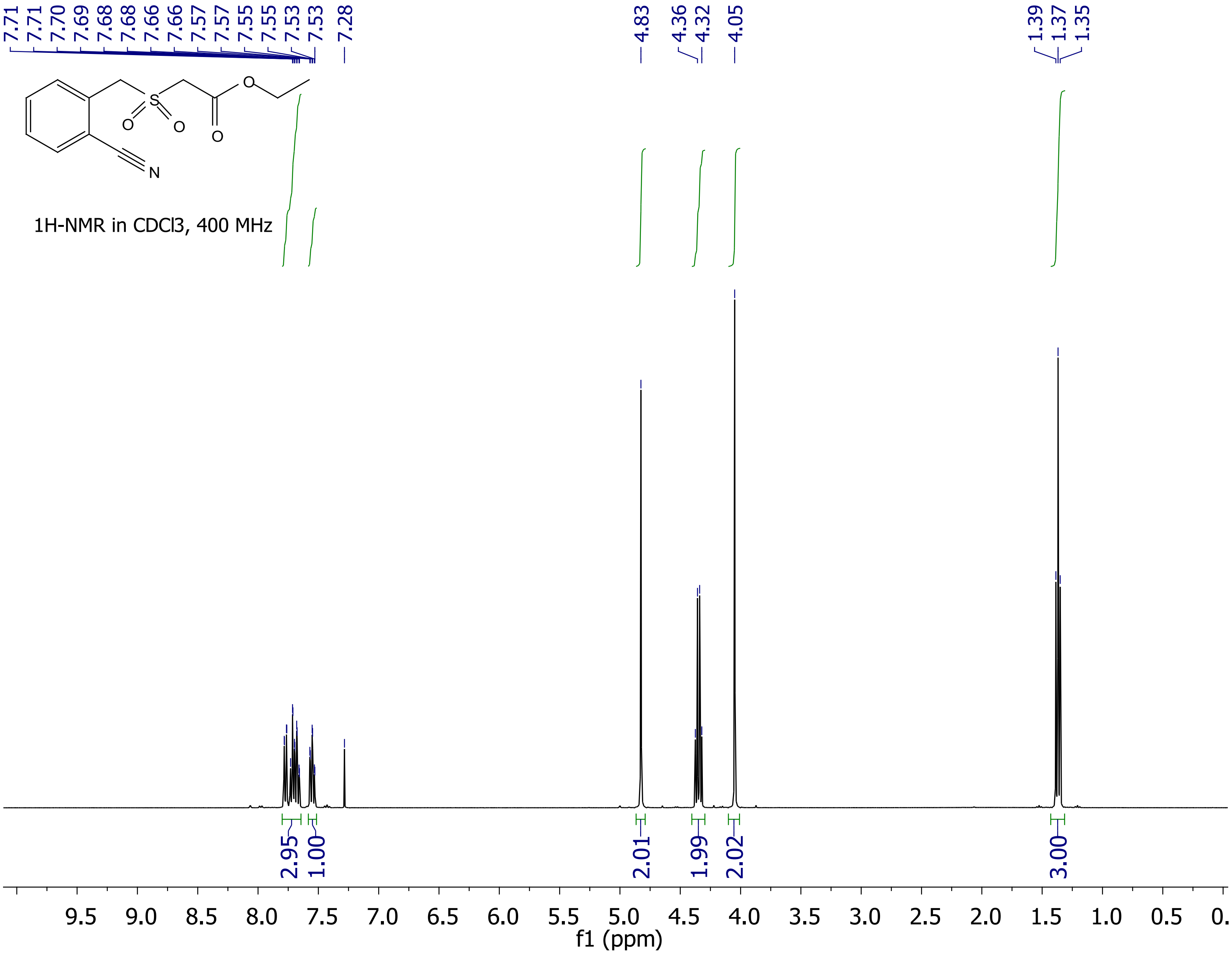
7.71
7.71
7.70
7.69
7.68
7.68
7.66
7.66
7.57
7.57
7.55
7.55
7.53
7.53
7.28
4.83
4.36
4.32
4.05
1.39
1.37
1.35
O
S
O
O
O
N
1H-NMR in CDCl3, 400 MHz
2.95
1.00
2.01
1.99
2.02
3.00
9.5
9.0
8.5
8.0
7.5
7.0
6.5
6.0
5.5
5.0
4.5
4.0
3.5
3.0
2.5
2.0
1.5
1.0
0.5
0.
f1 (ppm)

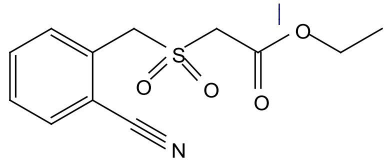

13C-NMR in CDCl3, 100 MHz

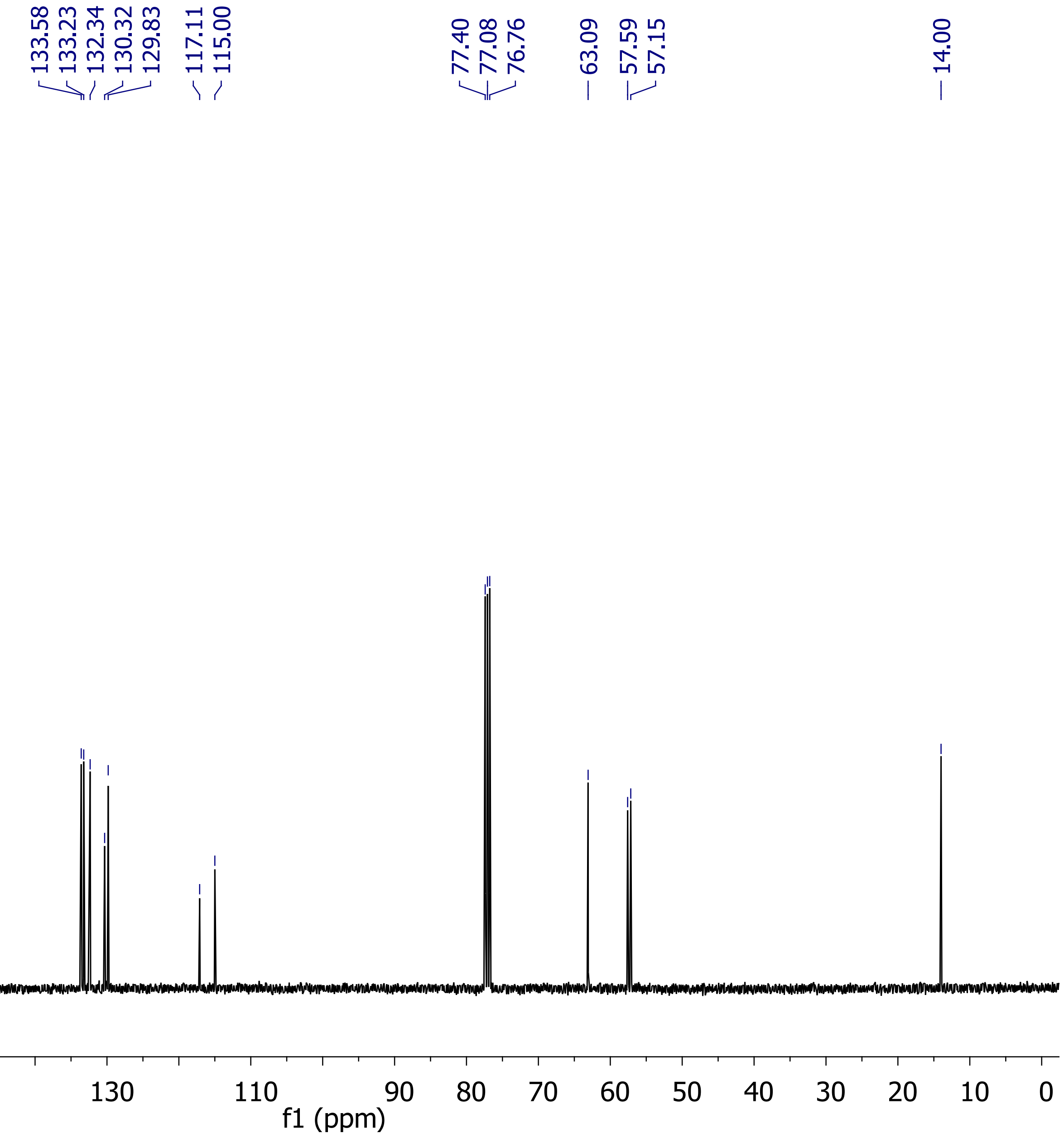

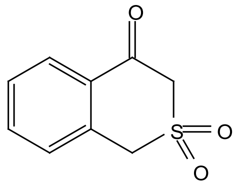

1H-NMR in DMSO-d6, 400 MHz

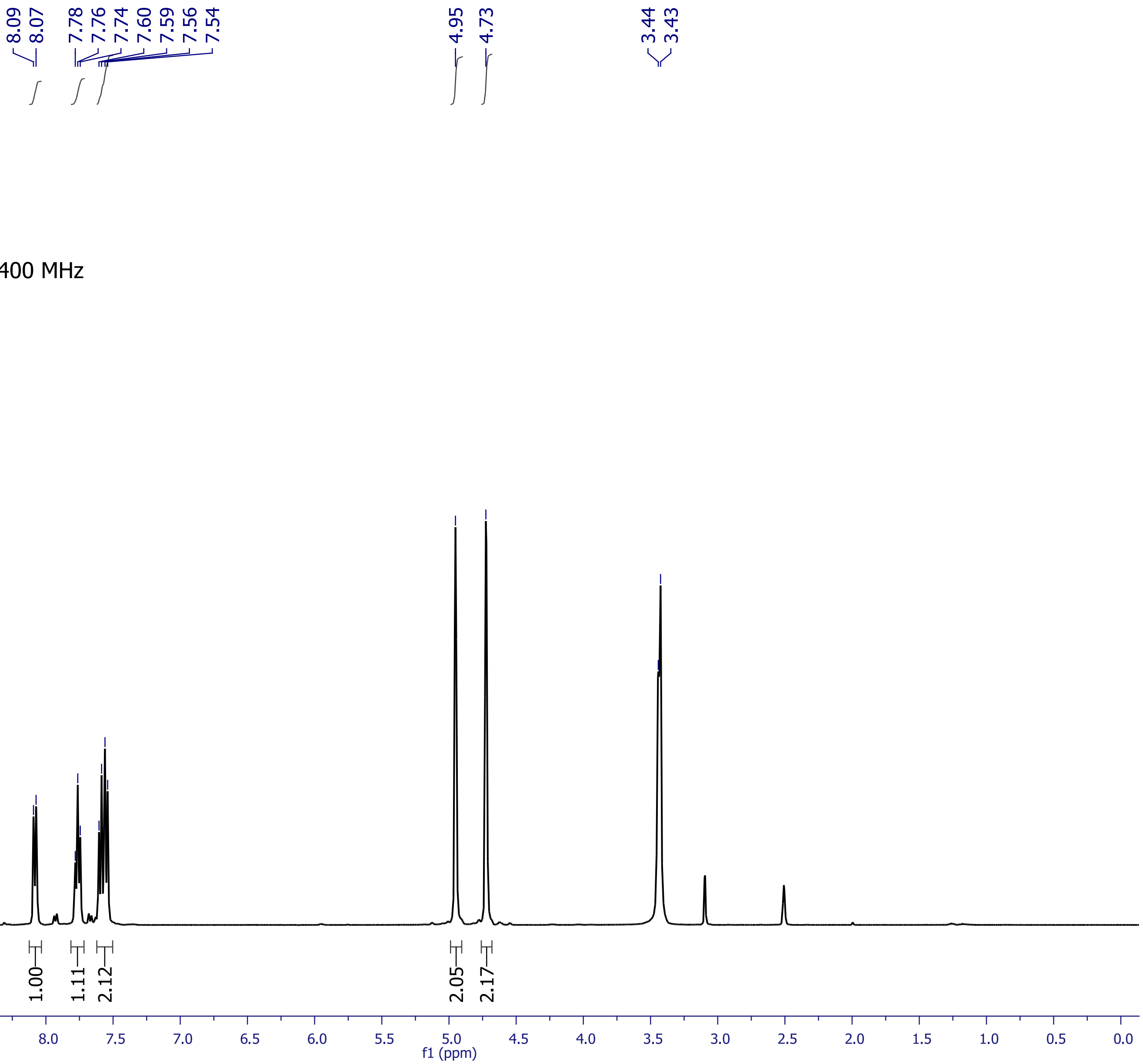

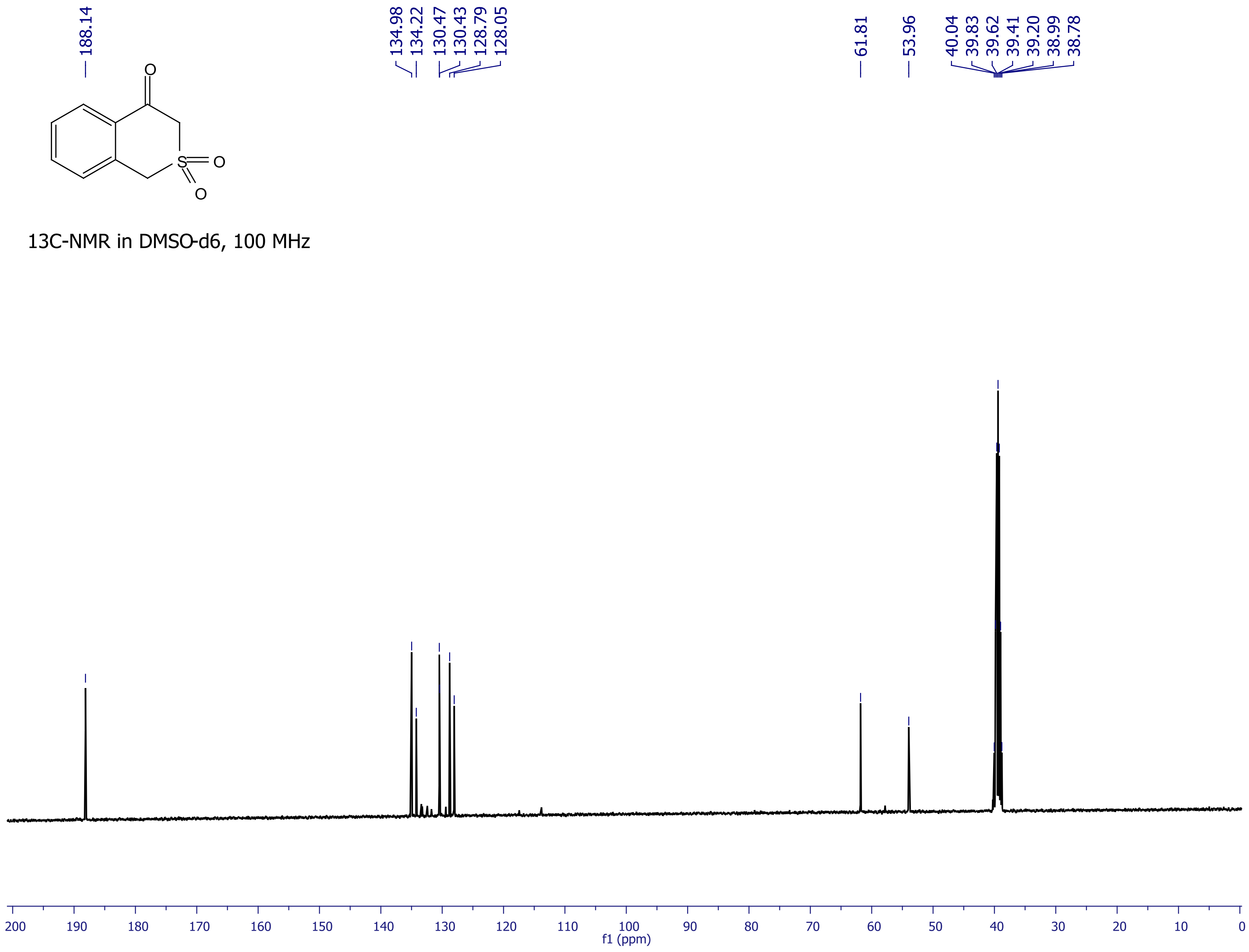

188.14
134.98
134.22
130.47
130.43
128.79
128.05
61.81
53.96
40.04
39.83
39.62
39.41
39.20
38.99
38.78
13C-NMR in DMSO-d6, 100 MHz
f1 (ppm)

1HNMR, in CDCl3, 400 MHz

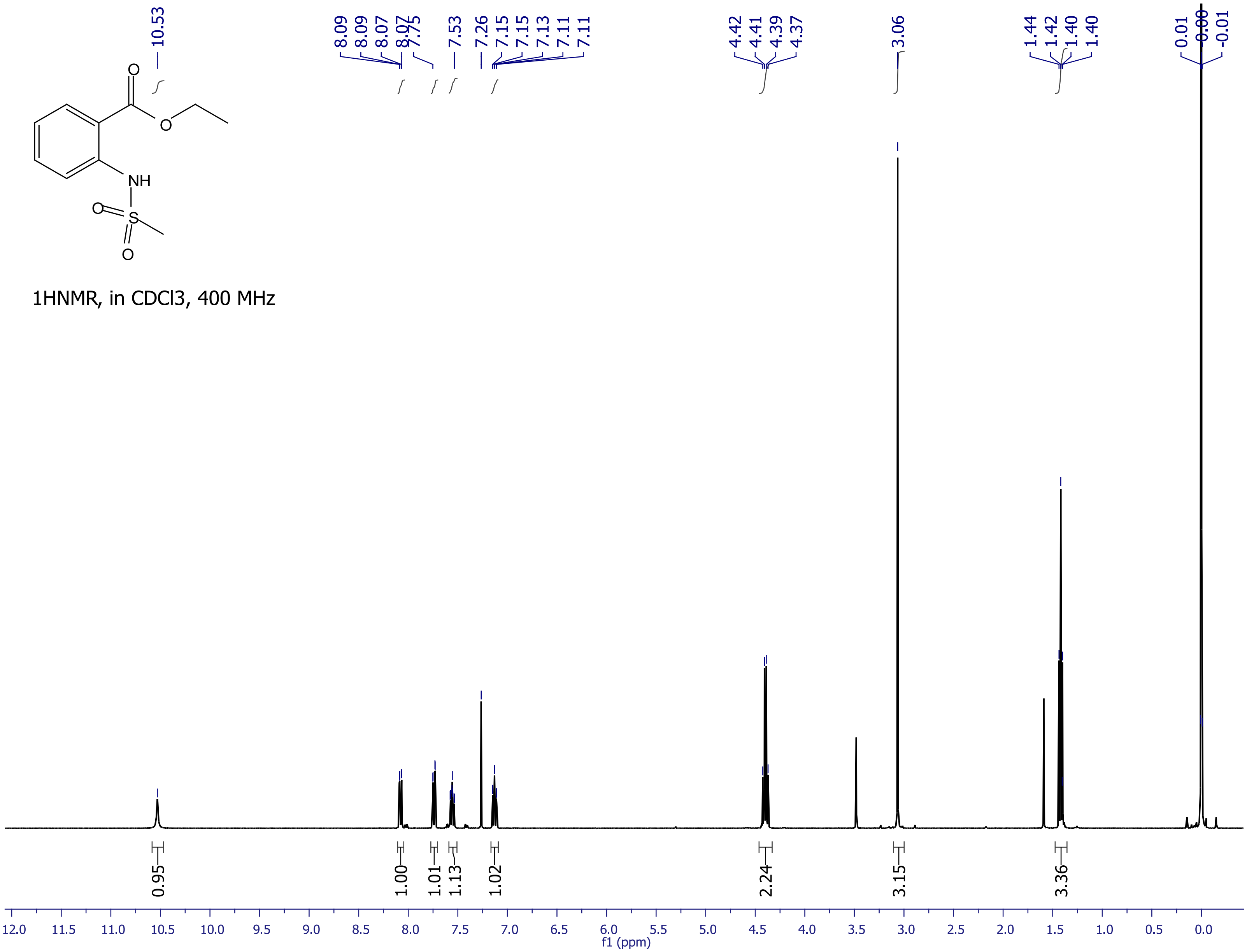

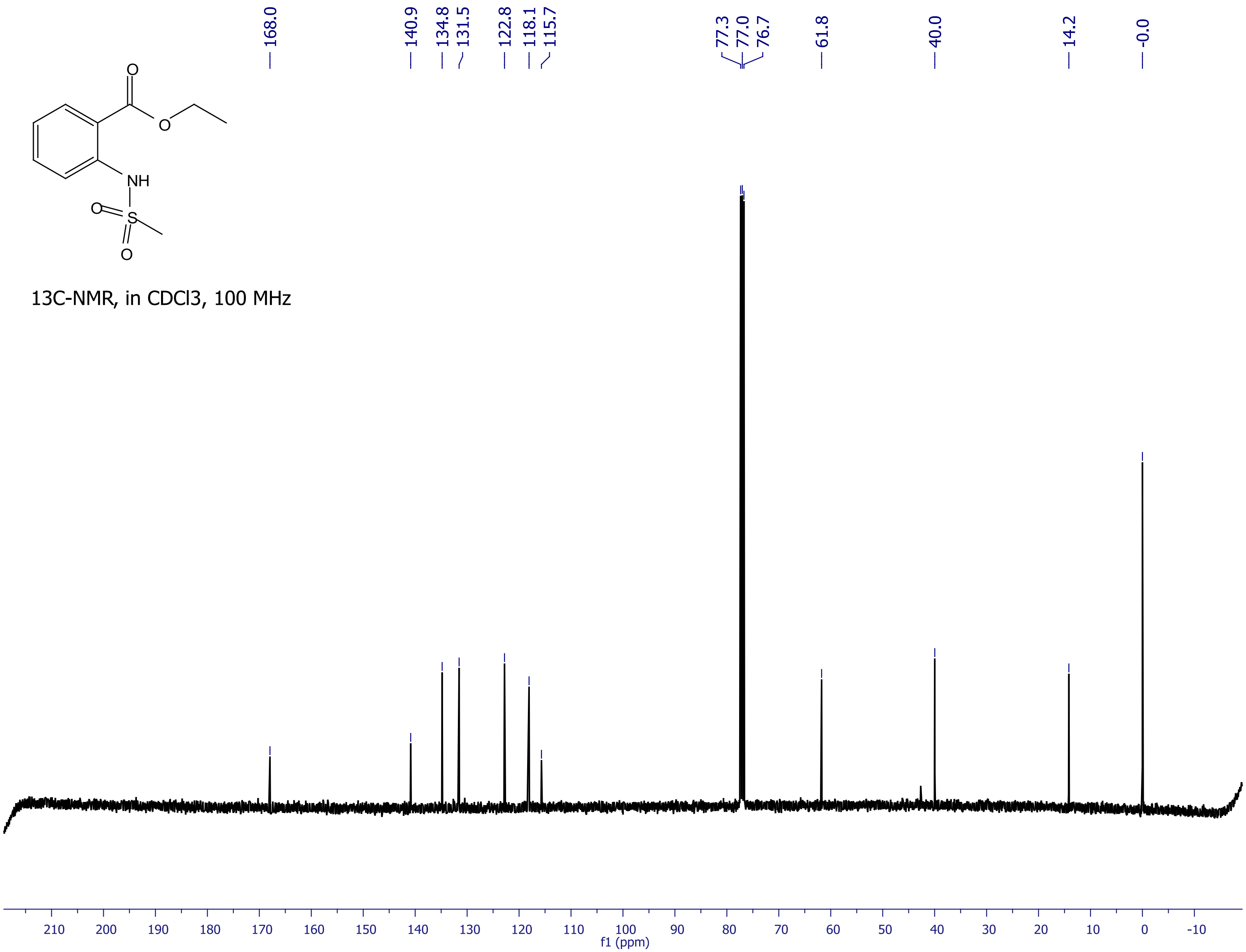
13C-NMR, in CDCl3, 100 MHz
168.0
140.9
134.8
131.5
122.8
118.1
115.7
77.3
77.0
76.7
61.8
40.0
14.2
-0.0
f1 (ppm)

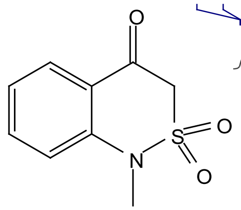

1H-NMR, in CDCl3, 400 MHz

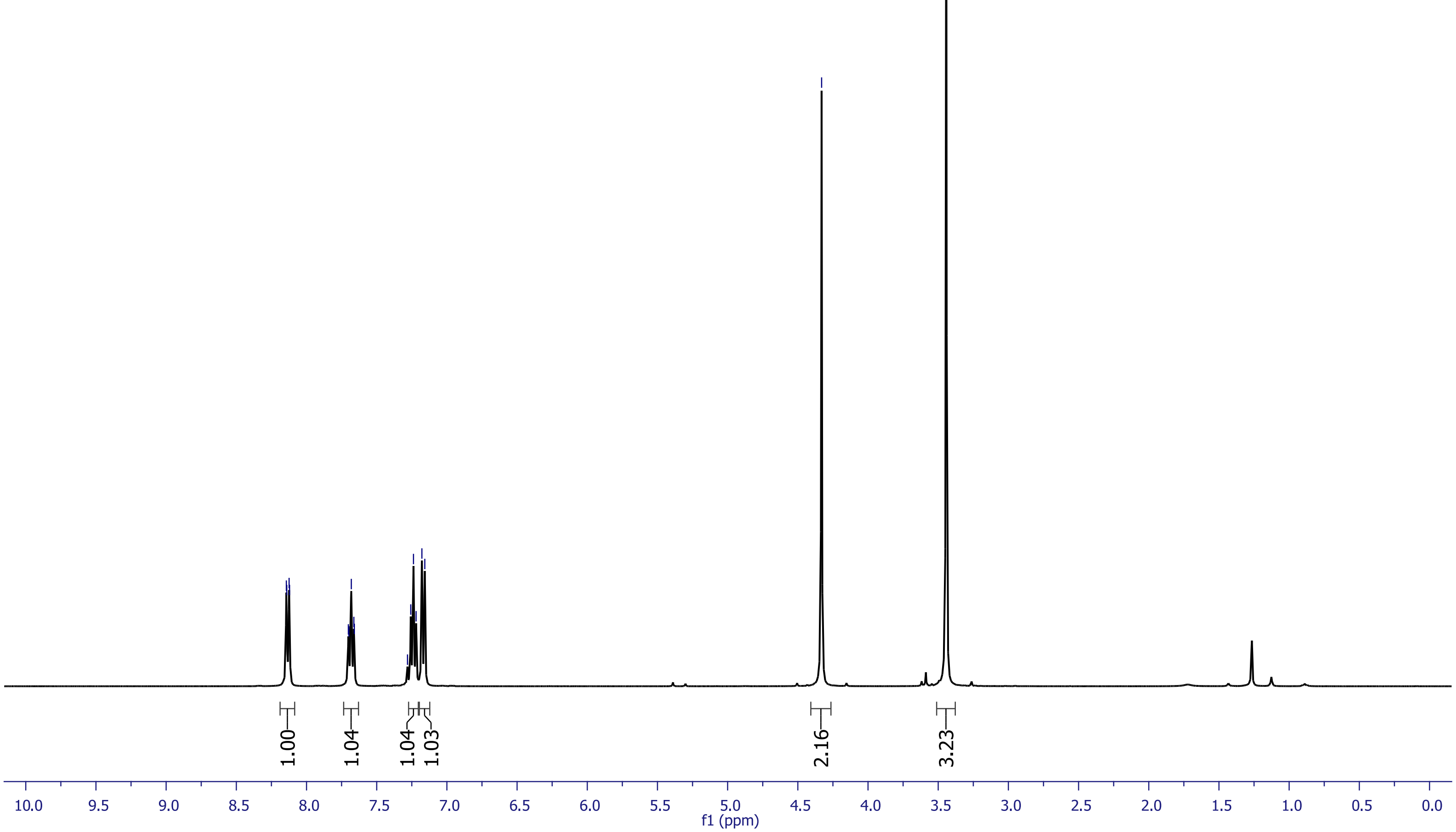

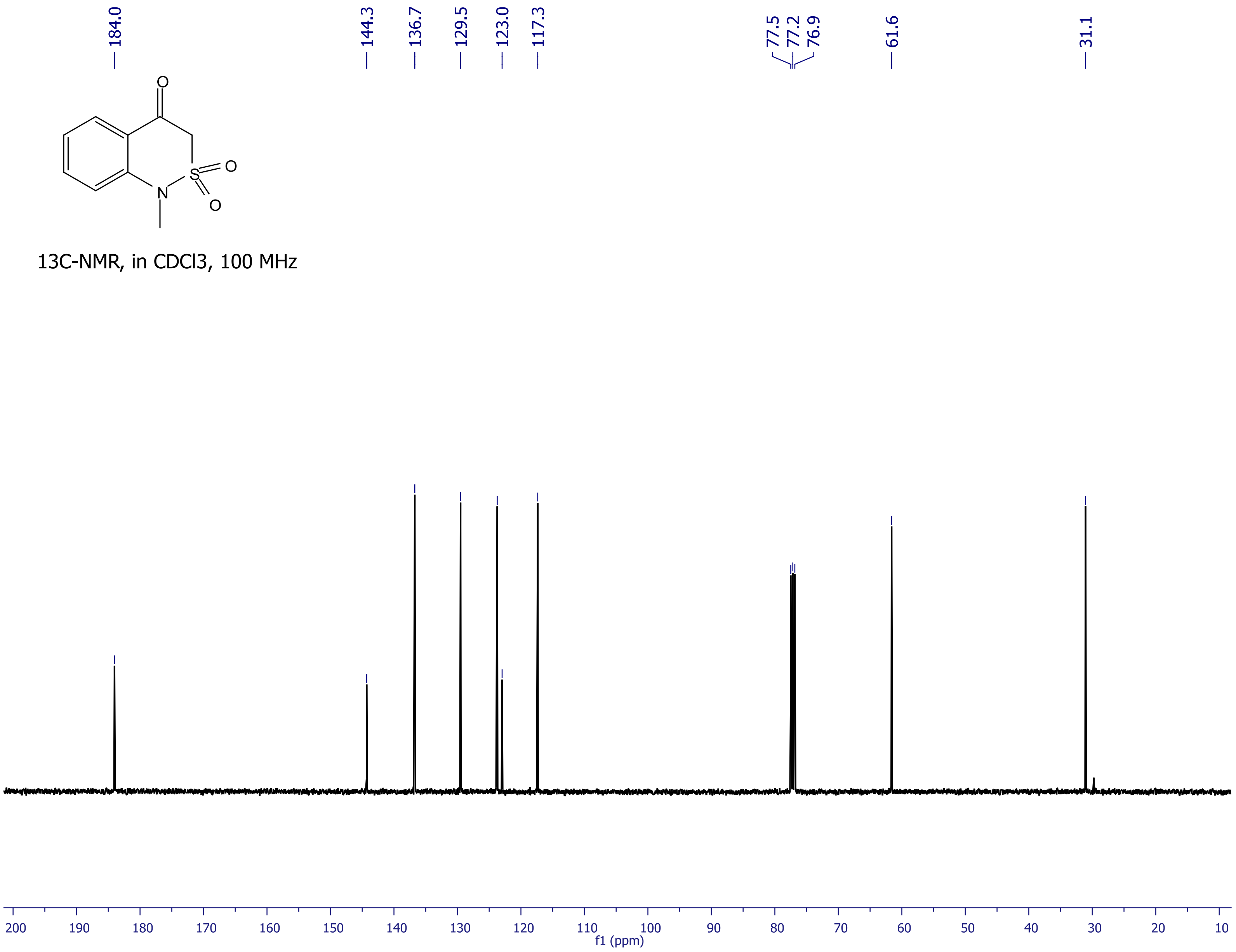
184.0
144.3
136.7
129.5
123.0
117.3
77.5
77.2
76.9
61.6
31.1
O
S
O
O
N
13C-NMR, in CDCl3, 100 MHz
200
190
180
170
160
150
140
130
120
110
100
90
80
70
60
50
40
30
20
10
f1 (ppm)

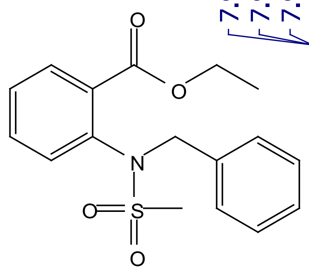

1H-NMR, in CDCl3, 400 MHz

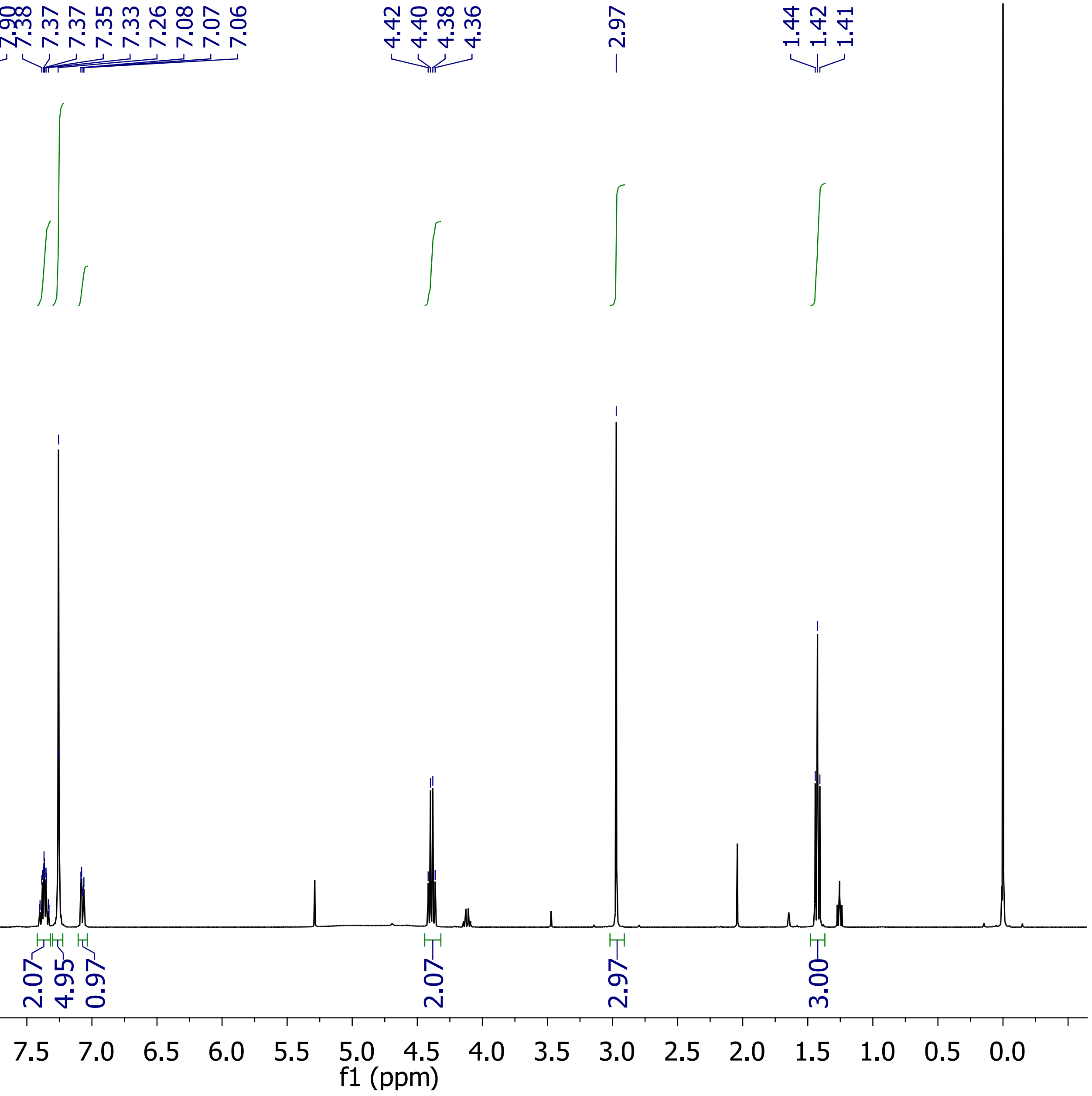

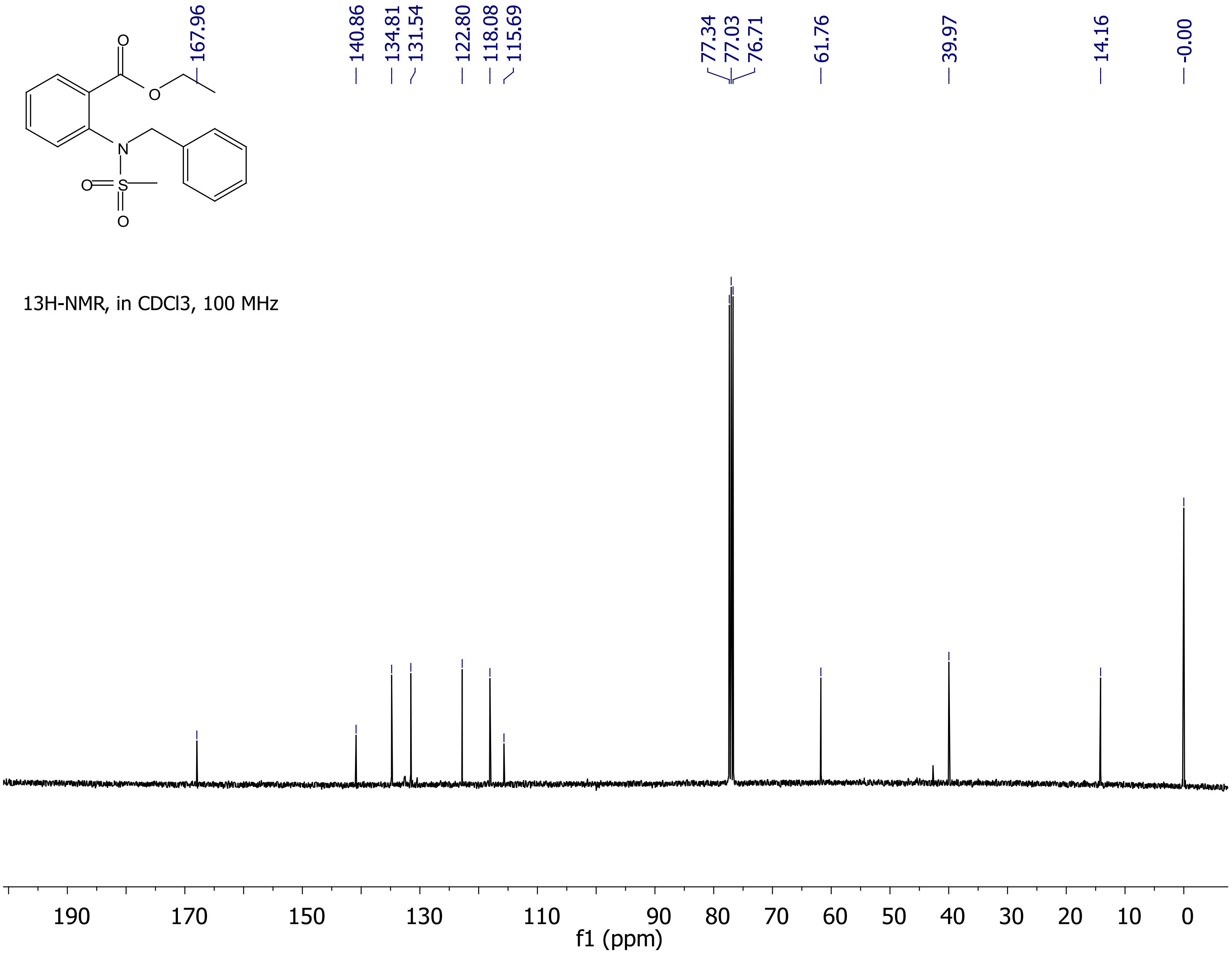
167.96
140.86
134.81
131.54
122.80
118.08
115.69
77.34
77.03
76.71
61.76
39.97
14.16
-0.00
13H-NMR, in CDCl3, 100 MHz
190 170 150 130 110 90 80 70 60 50 40 30 20 10 0
f1 (ppm)

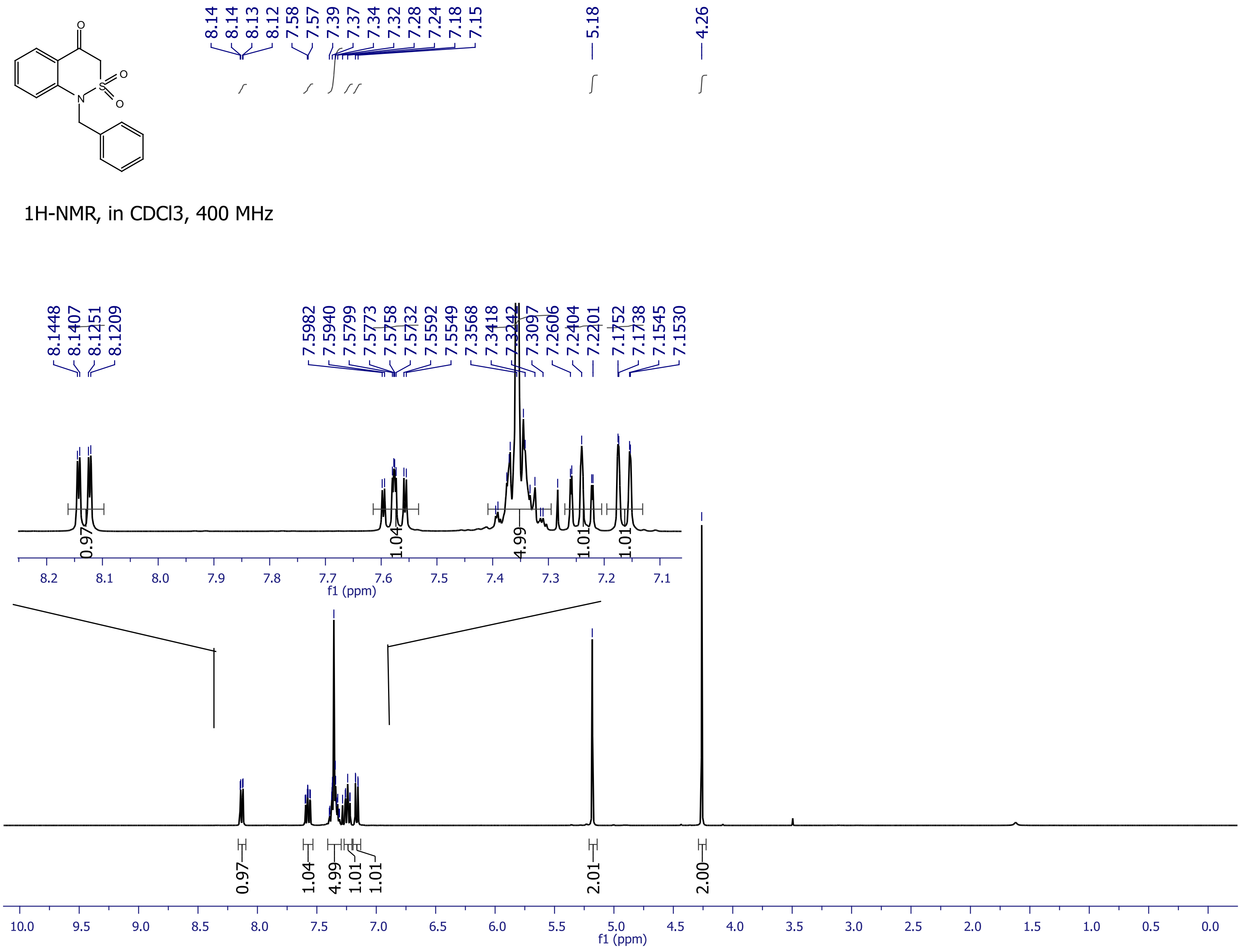

1H-NMR, in CDCl3, 400 MHz
8.14 8.14 8.13 8.12 7.58 7.57 7.39 7.37 7.34 7.32 7.28 7.24 7.18 7.15
5.18
4.26
8.1448 8.1407 8.1251 8.1209
7.5982 7.5940 7.5799 7.5773 7.5758 7.5732 7.5592 7.5549 7.3568 7.3418 7.3242 7.3097 7.2606 7.2404 7.2201 7.1752 7.1738 7.1545 7.1530
0.97 1.04 4.99 1.01 1.01 2.01 2.00
f1 (ppm)

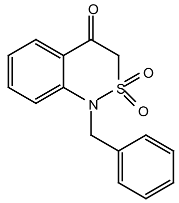

13C-NMR, in CDCl3, 100 MHz

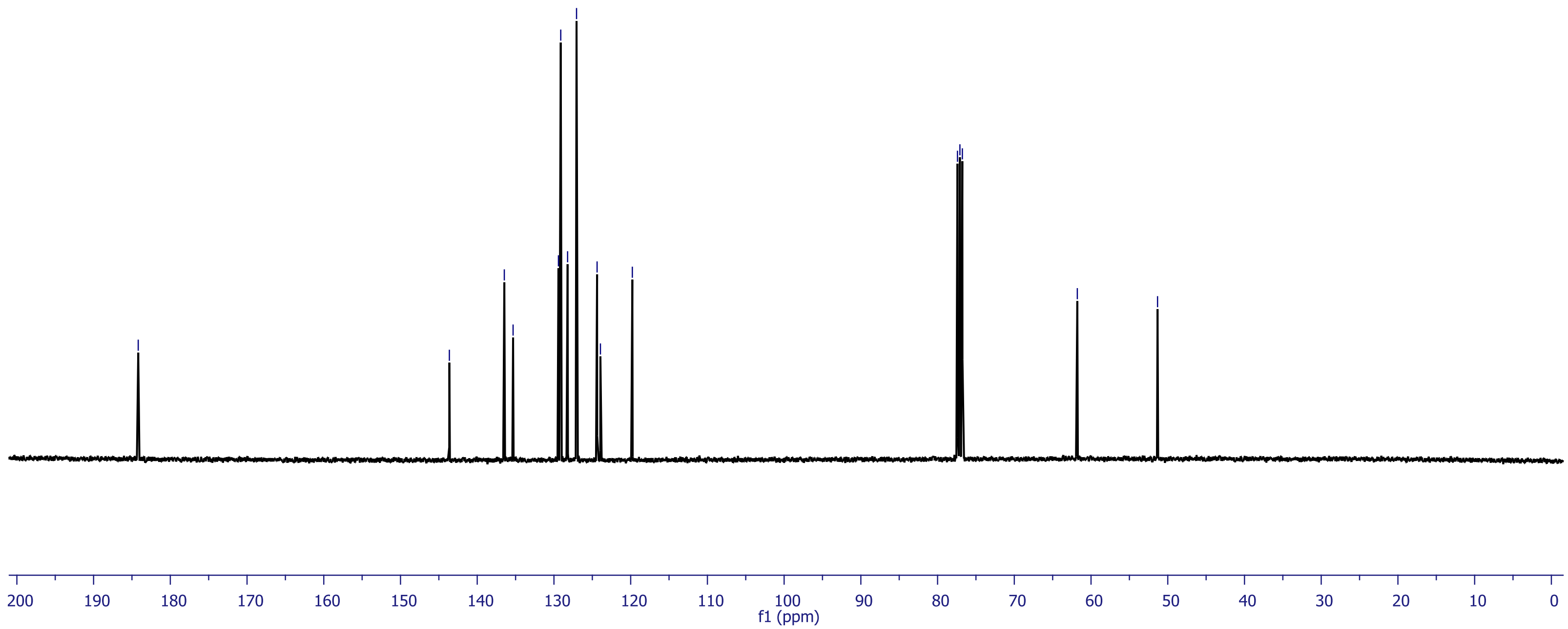

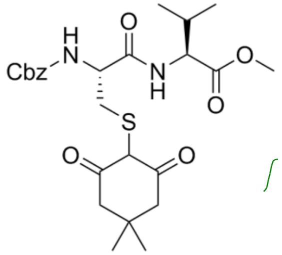

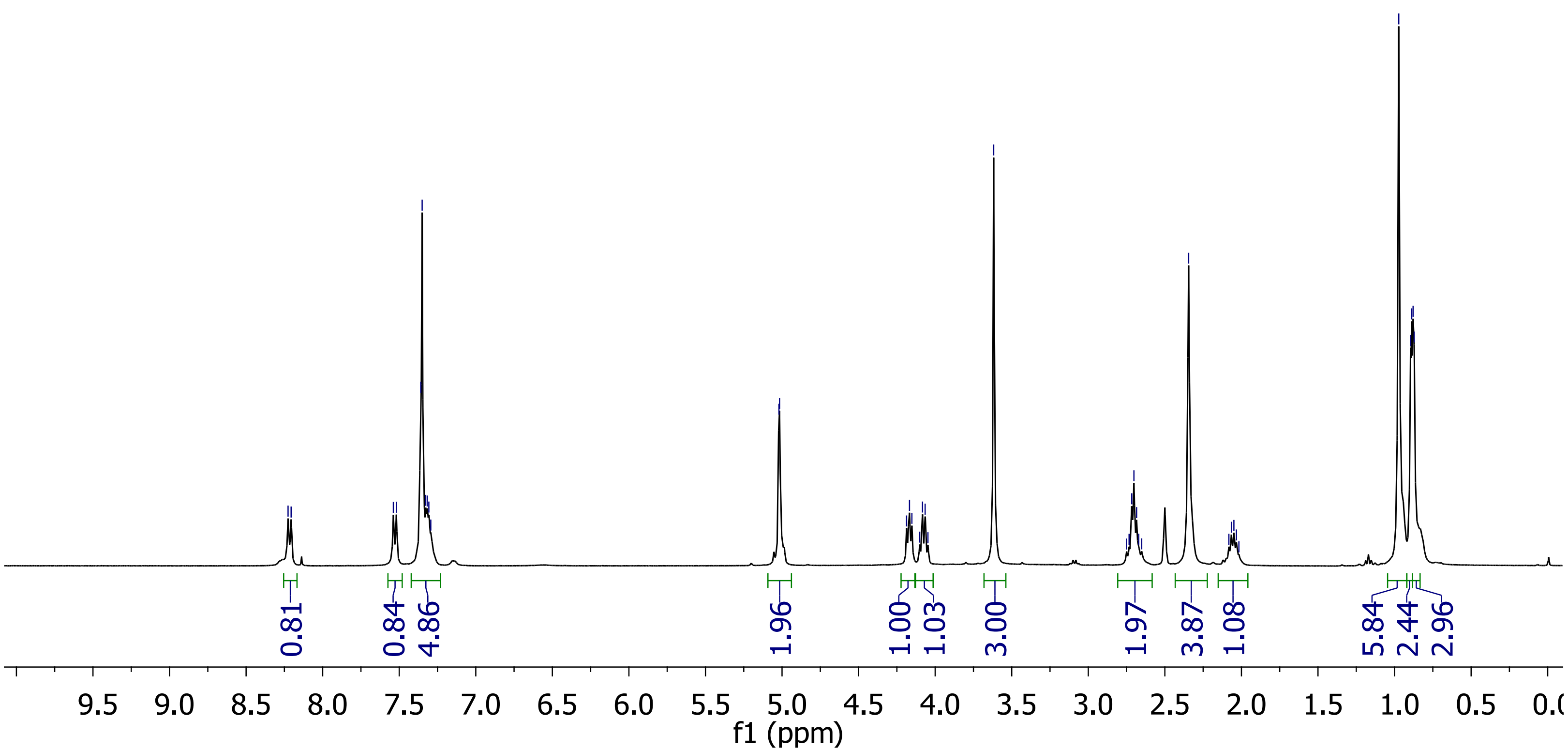

1H-NMR in DMSO-d6, 400 MHz

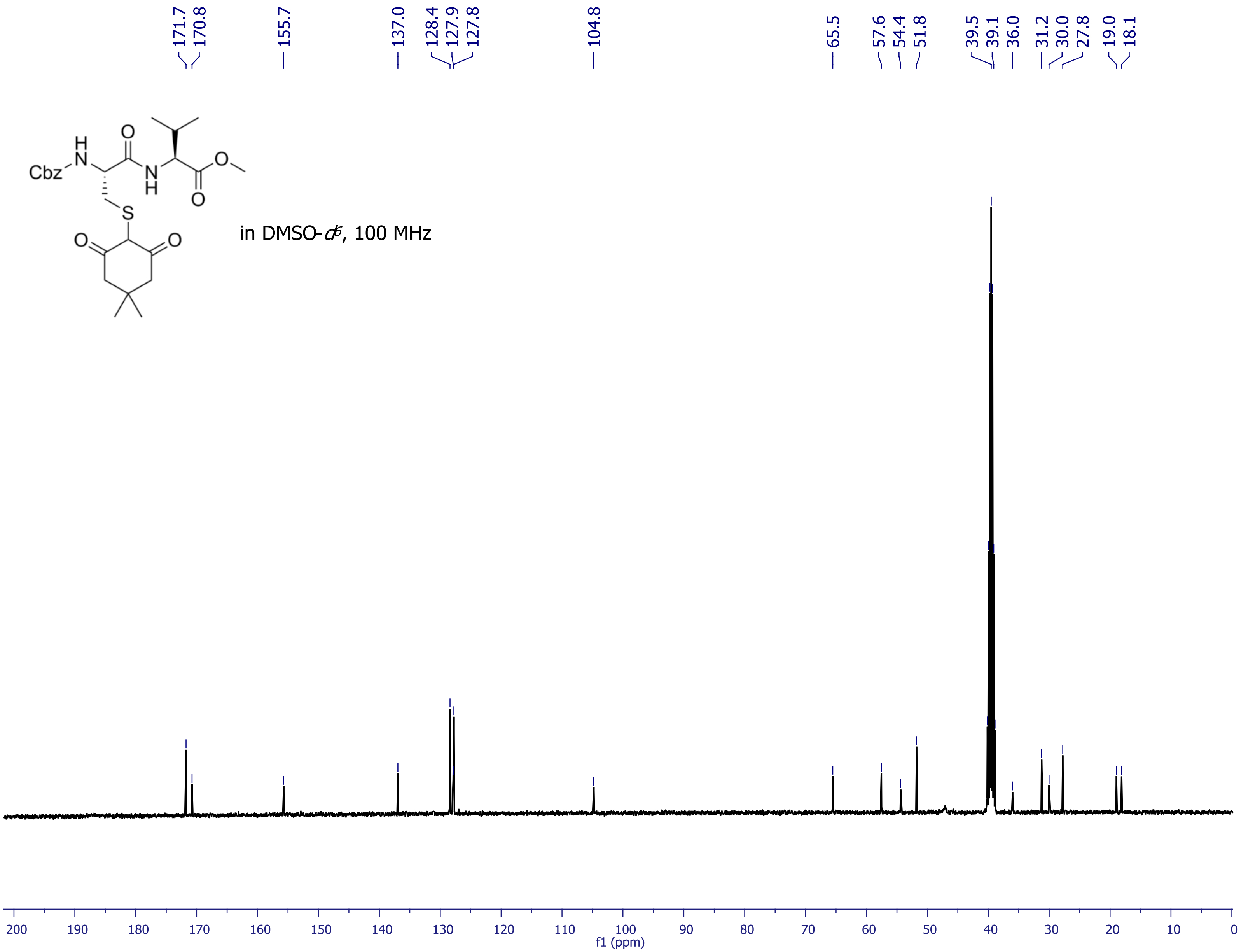
171.7
170.8
155.7
137.0
128.4
127.9
127.8
104.8
65.5
57.6
54.4
51.8
39.5
39.1
36.0
31.2
30.0
27.8
19.0
18.1
Cbz
in DMSO-d_6, 100 MHz
f1 (ppm)

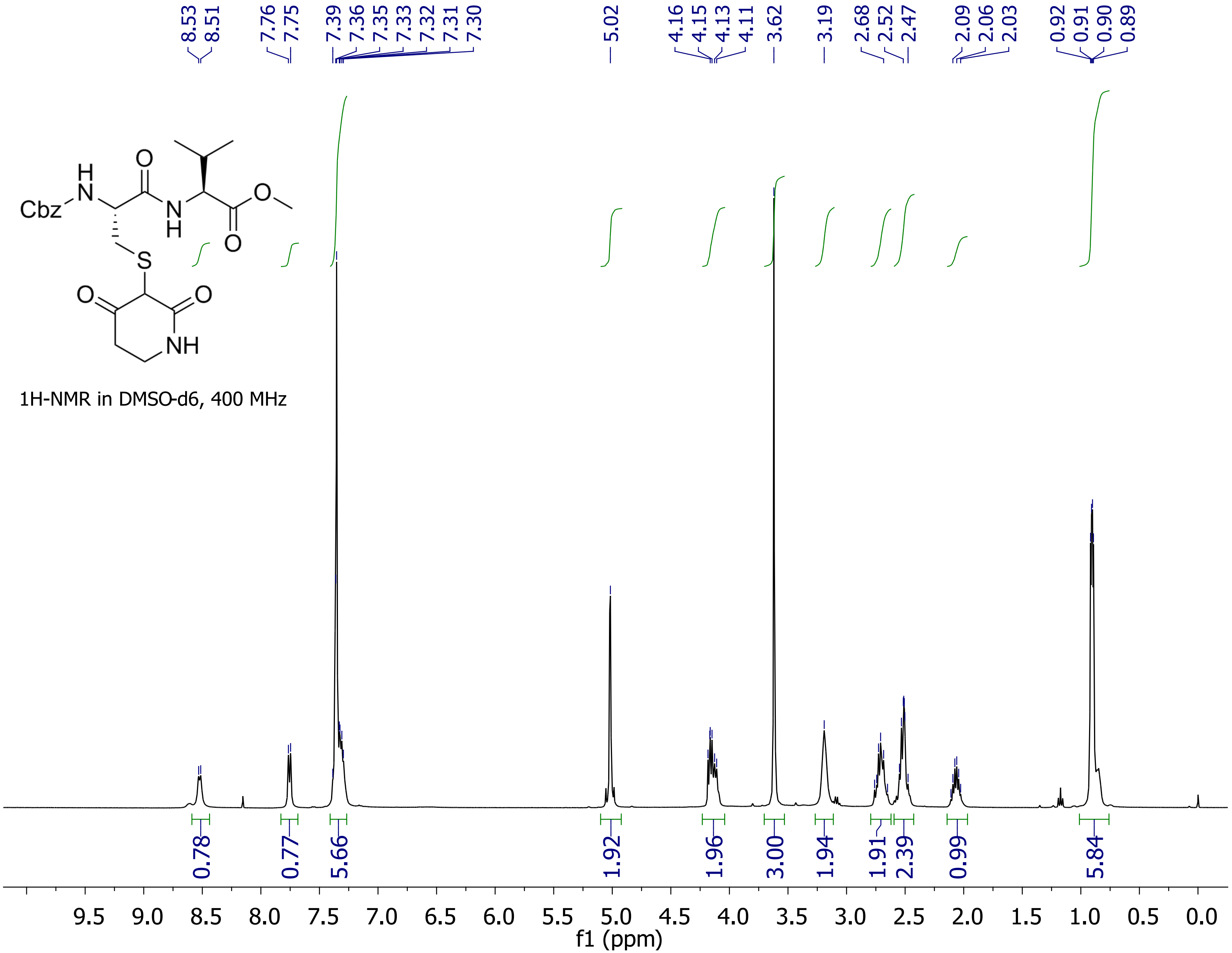
8.53
8.51
7.76
7.75
7.39
7.36
7.35
7.33
7.32
7.31
7.30
5.02
4.16
4.15
4.13
4.11
3.62
3.19
2.68
2.52
2.47
2.09
2.06
2.03
0.92
0.91
0.90
0.89
Cbz
1H-NMR in DMSO-d6, 400 MHz
0.78
0.77
5.66
1.92
1.96
3.00
1.94
1.91
2.39
0.99
5.84
f1 (ppm)

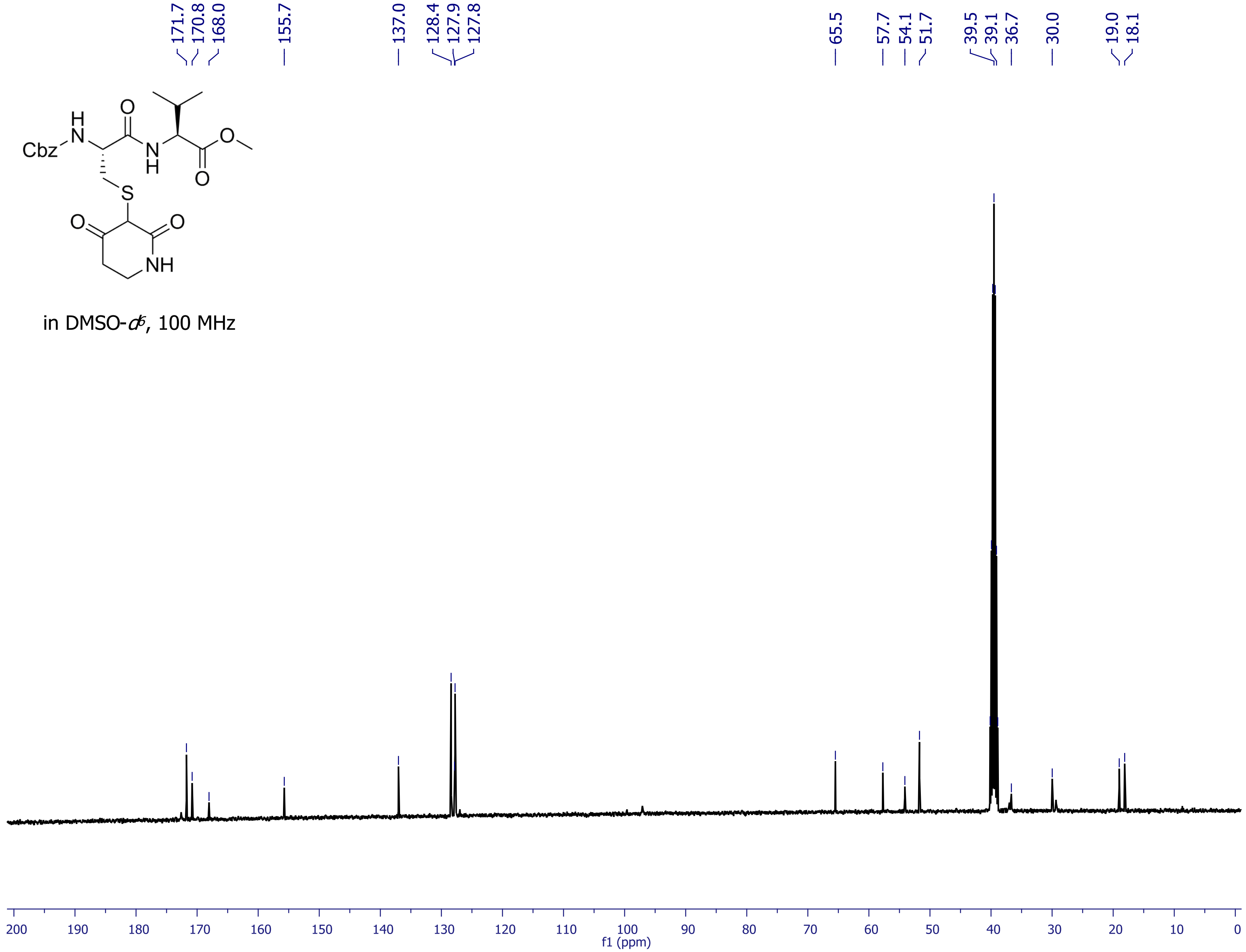
171.7
170.8
168.0
155.7
137.0
128.4
127.9
127.8
65.5
57.7
54.1
51.7
39.5
39.1
36.7
30.0
19.0
18.1
Cbz
H
N
O
N
H
O
O
S
O
O
NH
in DMSO-d_6, 100 MHz
200 190 180 170 160 150 140 130 120 110 100 90 80 70 60 50 40 30 20 10 0
f1 (ppm)

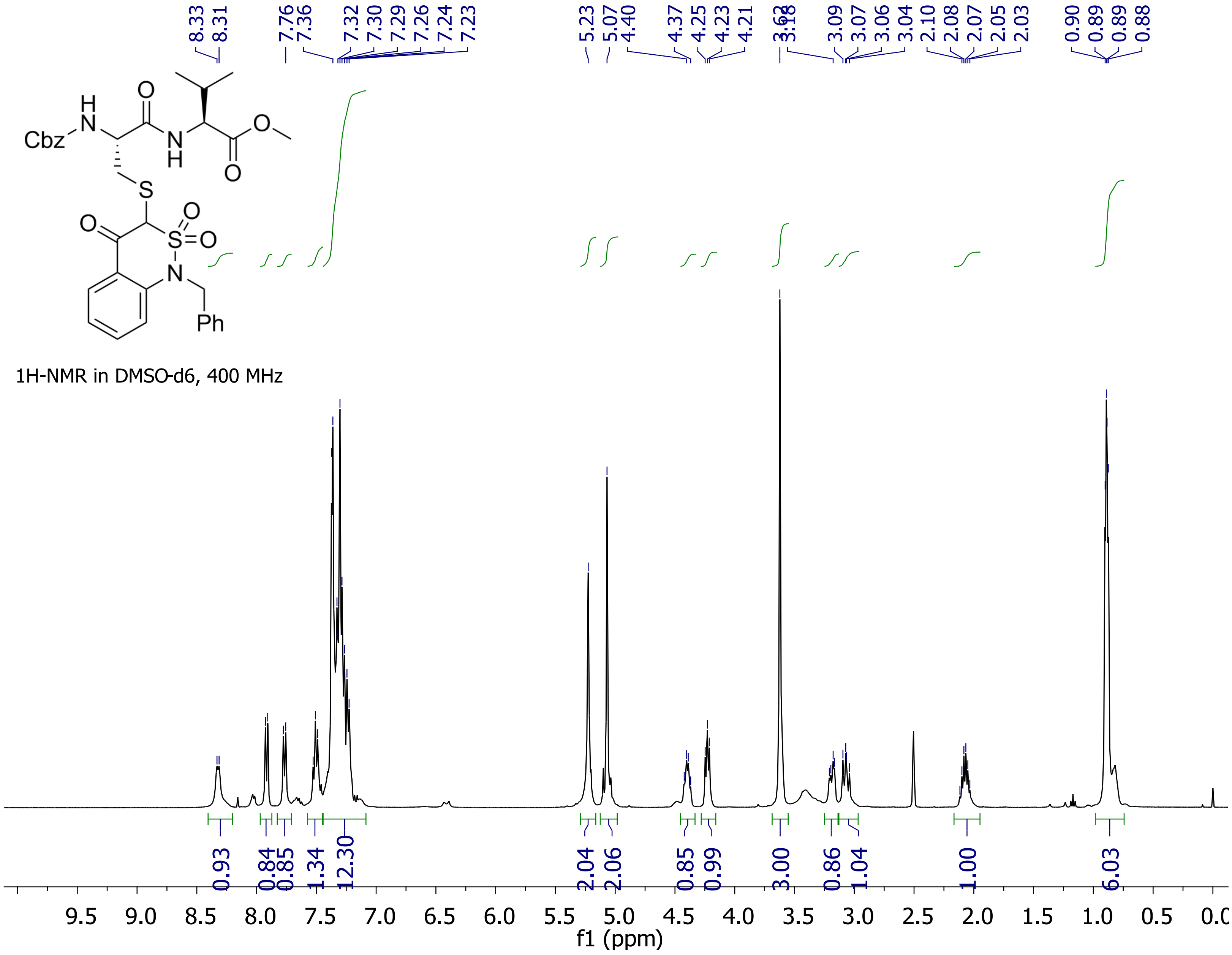

Cbz
1H-NMR in DMSO-d6, 400 MHz
f1 (ppm)

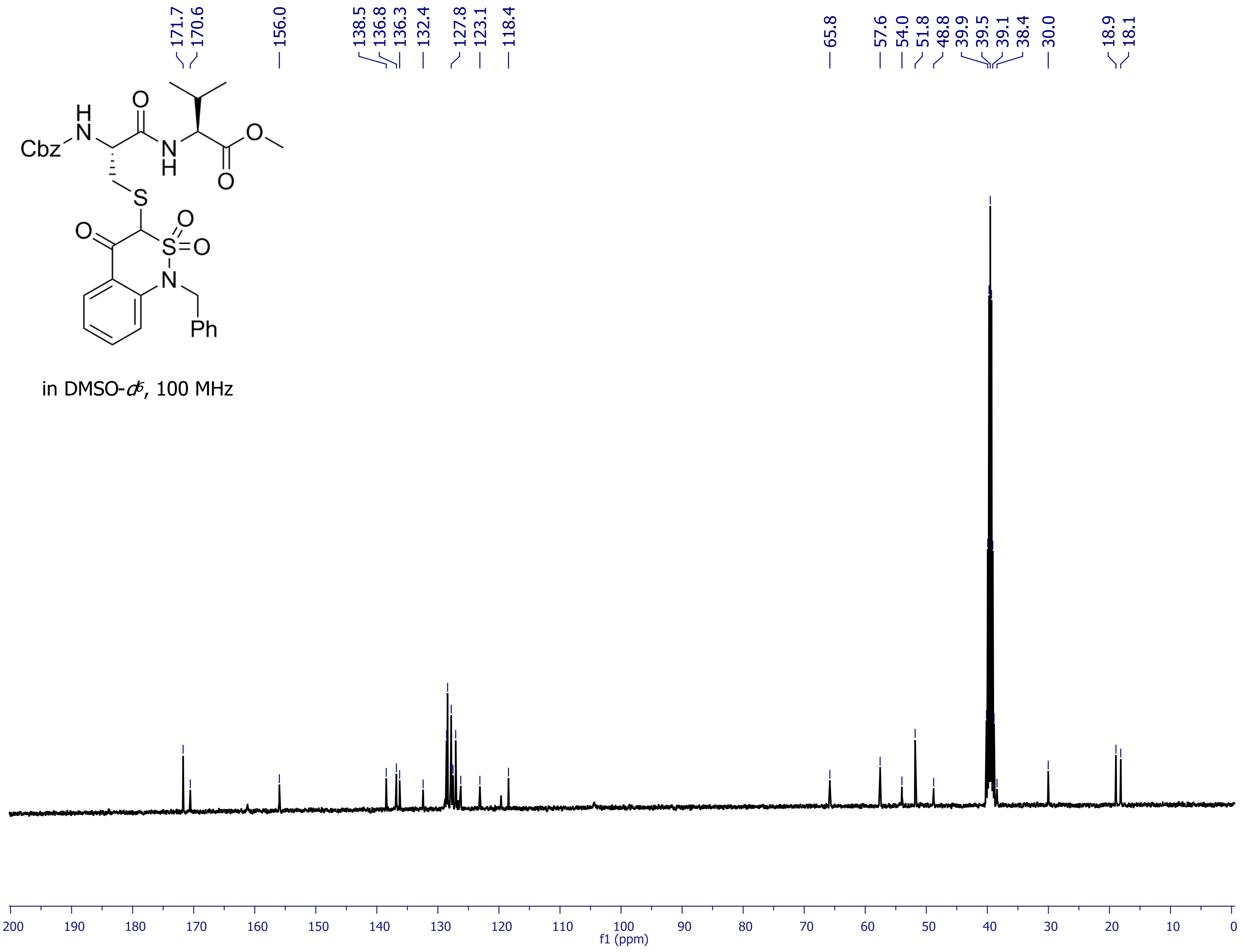
171.7
170.6
156.0
138.5
136.8
136.3
132.4
127.8
123.1
118.4
65.8
57.6
54.0
51.8
48.8
39.9
39.5
39.1
38.4
30.0
18.9
18.1
Cbz
in DMSO-d6, 100 MHz
f1 (ppm)
