# Supplementary material for: Profiling the reactivity of cyclic C-nucleophiles towards electrophilic sulfur in cysteine sulfenic acid
Source: Chem Sci. 2015 Oct 7;7(1):400–15. doi: 10.1039/c5sc02569a (PMC4724439; doi:10.1039/c5sc02569a)
Supplement: Supplementary file 2 [file SC-007-C5SC02569A-s002.pdf]

Electronic Supplementary Material (ESI) for Chemical Science.
This journal is © The Royal Society of Chemistry 2015

Profiling the Reactivity of Cyclic C-Nucleophiles towards Electrophilic Sulfur in Cysteine Sulfenic Acid

Supporting information

Vinayak Gupta and Kate S. Carroll*
Department of Chemistry, The Scripps Research Institute, Jupiter, Florida, 33458

Contents:

1. General information & instrumentation……SI-2
2. Sample preparation for LC-MS assay……SI-3
3. Aqueous instability of cyclic sulfenamide **(14)** ……SI-4
4. 2nd order rate studies of the self-reaction of cyclic sulfenamide **(14)**……SI-6
5. Stability of cyclic sulfenamide in acetonitrile ……SI-7
6. Nucleophilic reactions of dipeptide sulfenic acid ……SI-8
7. pH dependence of the self-reaction of cyclic sulfenamide **(14)** ……SI-12
8. Kinetics of the reaction of dimedone with dipeptide sulfenic acid **(15)** ……SI-13
9. Comparative reactivity of cyclic sulfenamide models with dimedone ……SI-14
10. pH dependence of the reaction of dipeptide sulfenic acid with **21a** ……SI-18
11. Structures of aromatic nucleophiles, enamines and hydrazides ……SI-19
12. Pseudo 1st order rate constant plots ……SI-20
13. 2nd order rate constant plots ……SI-32
14. Experimental procedures and NMRs……SI-35
15. Cross-reactivity profiles of selected nucleophiles ……SI-59
16. Synthesis of dipeptide sulfenic acid adduct with various nucleophiles ……SI-75
17. Stability of dipeptide-S-nucleophile adducts under reducing conditions ……SI-77
18. Labeling of protein-SOH with various nucleophiles ……SI-84
19. Competitive labeling of protein-SOH with various nucleophiles ……SI-98
20. Effect of keto-enol tautomerism on the reactivity of C-nucleophiles ……SI-105

Synthetic Materials and Methods.

All reactions were conducted in flame-dried glassware under nitrogen pressure with dry solvents, unless otherwise noted. Tetrahydrofuran (THF) was freshly distilled from sodium/benzophenone. All chemicals were purchased from Sigma-Aldrich (St. Louis, MO) and used as received. Silica gel P60 (Sorbent Technologies) was used for column chromatography. Reactions were monitored by thin layer chromatography (TLC) carried out using Analtech 60 F254 silica gel (precoated sheets, 0.25 mm thick). ^{1}H-NMR and ^{13}C-NMR spectra were collected in DMSO-d_6 or $CDCl_3$ (Cambridge Isotope Laboratories, Cambridge, MA) at 400 and 100 MHz respectively, using a Bruker AM-400 instrument with chemical shifts relative to residual $CHCl_3$ (7.27 and 77.37 ppm). Low resolution mass spectral analyses were carried out on an Agilent LC/MS system. All spectra are available upon request.

Instrumentation for kinetics assay (Rate Studies)

The LC-MS used was Agilent technologies 1220 Infinity LC and Agilent Technologies 6120 quadrupole MS. The column used was Agilent Poroshell 120 SB C-18, 2.7 μM particle size and dimensions were 3.0 x 50 mm. LC-MS grade solvents were used which were buffered with 0.1% Formic acid (LC-MS grade). Data was analyzed by LC/MSD ChemStation (Rev. B.04.03-SP1).

KaleidaGraph (version 4.1.1) was used for graphing and further data analysis to obtain pseudo unimolecular 1st order rate constants, 2nd order rate constants and pH plots.

Following equations were used for the purpose of graphing:

Pseudo unimolecular 1st order rate constant (k_{obs}) -

$$m1 * (1 - \exp(-m2 * m0)); m1 = xx; m2 = yy;$$

2nd order rate constant –

$$m1 + m2 * M0;\ m1 = 1;\ m2 = 1$$

pH plots -

$$(m1/(1+(10^\wedge(-m0))/(10^\wedge(-m2))))+m3; m1=xx; m2=yy; m3=zz$$

Sample Preparation and General LC-MS Assay

Sample preparation - Stock solutions of nucleophiles were prepared in DMSO (100 mM concentration). Stock solutions were then diluted to appropriate concentration in PBS (10 mM, pH = 7.4). The pH was checked for each stock solution and was found to be in range of 7.40 – 7.45. Similarly, a 100 mM stock solution of dipeptide sulfenamide was prepared in acetonitrile and diluted to appropriate concentration in acetonitrile.

Assay - For each rate study, to a 2 mL solution of the nucleophile, was added 1 mL solution of cyclic sulfenamide. Effective concentrations were: Cyclic sulfenamide – 10, 25 or 50 or 100 μM; Nucleophile – 50, 125 or 250 μM, 1.0 mM or higher. Resulting reaction mixture was quenched after regular intervals by taking out a measured aliquot (300 μL) and adding it to a LC-MS vial containing formic acid (100 μL) and analyzing it by LC-MS. Area under the curve for the product formation at each time point was obtained from LC and plotted against time to get k_{obs}.

LC-MS method – The gradient was started at 95% H_2O – 5% acetonitrile (0 minutes) with a flow rate of 1 ml/min. The gradient was changed to 0% H_2O – 100% acetonitrile over 5 min. with same flow rate. This gradient was maintained for 2 min. Total run time was 7 minutes followed by a 1.4 min. of post-time. The LC trace was obtained by monitoring 190 nm wavelength.

Assay approximation - In the cases of kinetically faster nucleophiles (where < 1 mM nucleophile concentrations were used), the k_{obs} was adjusted to [Nu] = 1 mM by multiplying the observed rate values with appropriate factor for comparison purposes. The correction presupposes that the same rate law applies throughout the entire concentration range, which may or may not hold true.

Standard deviation for k_{obs} reported are $\leq$ 7.5% and representative plot for each nucleophile is shown here.

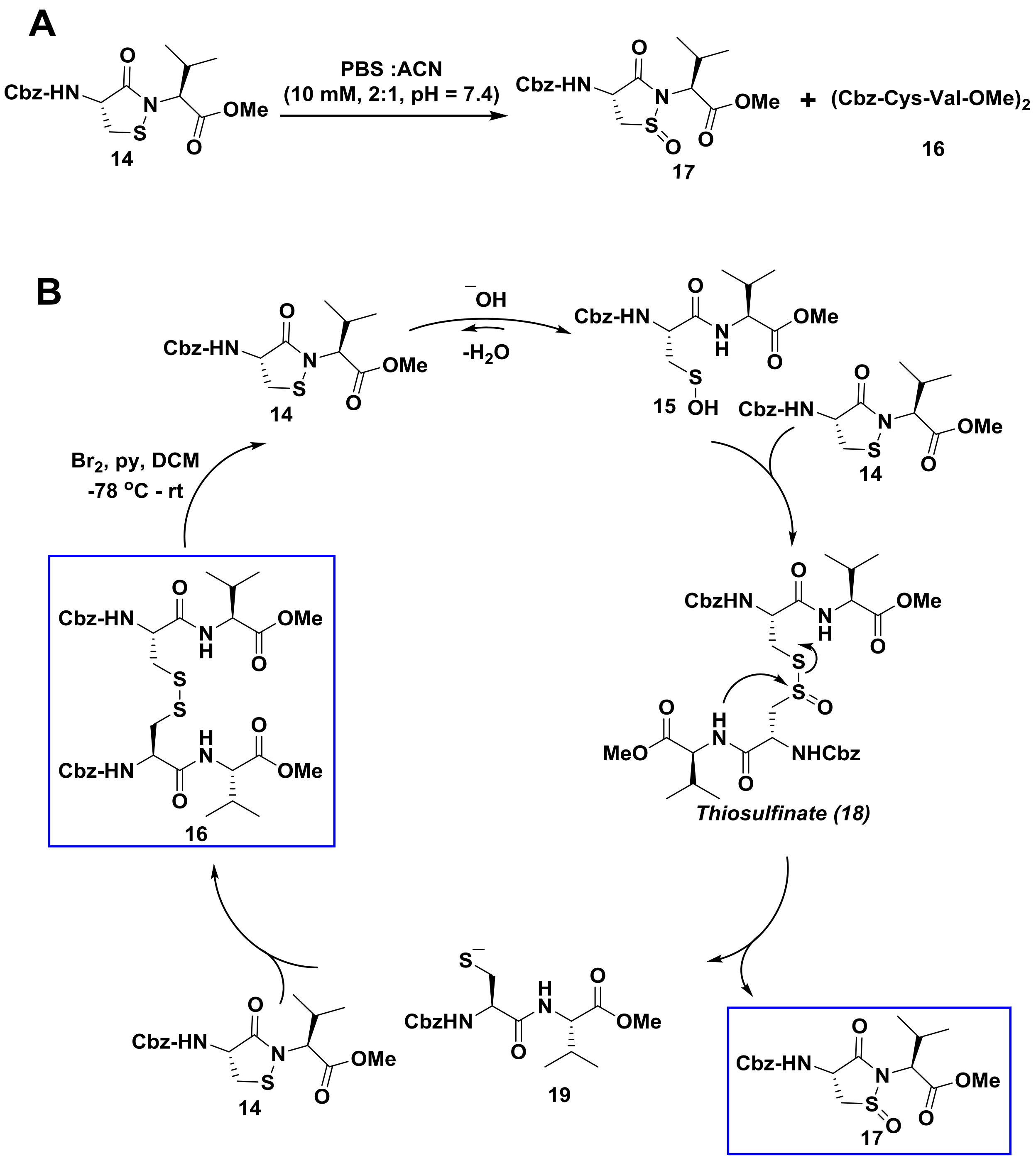

Scheme S1. Under aqueous conditions, cyclic sulfenamide **(14)** exists in equilibrium with sulfenic acid **(15)**. **(A)** Aqueous instability of cyclic sulfenamide **14** results in the formation of cyclic sulfinamide **(17)** and disulfide **(16)**; **(B)** Proposed mechanism for the formation of cyclic sulfinamide and disulfide as a result of self reaction of cyclic sulfenamide. Under aqueous conditions, cyclic sulfenamide **(14)** exist in equilibrium with

sulfenic acid **(15)**. In the absence of a nucleophile, **14** and sulfenic acid **15** react with each other to generate thiosulfinate ester **(18)**. Nitrogen of the backbone amide of **18** forms a covalent bond with sulfoxide forming cyclic sulfinamide **(17)** and releasing thiolate **(19)** in the process. Thiolate **(19)** then reacts with **14** (or **15**) and form the disulfide **(16)**.

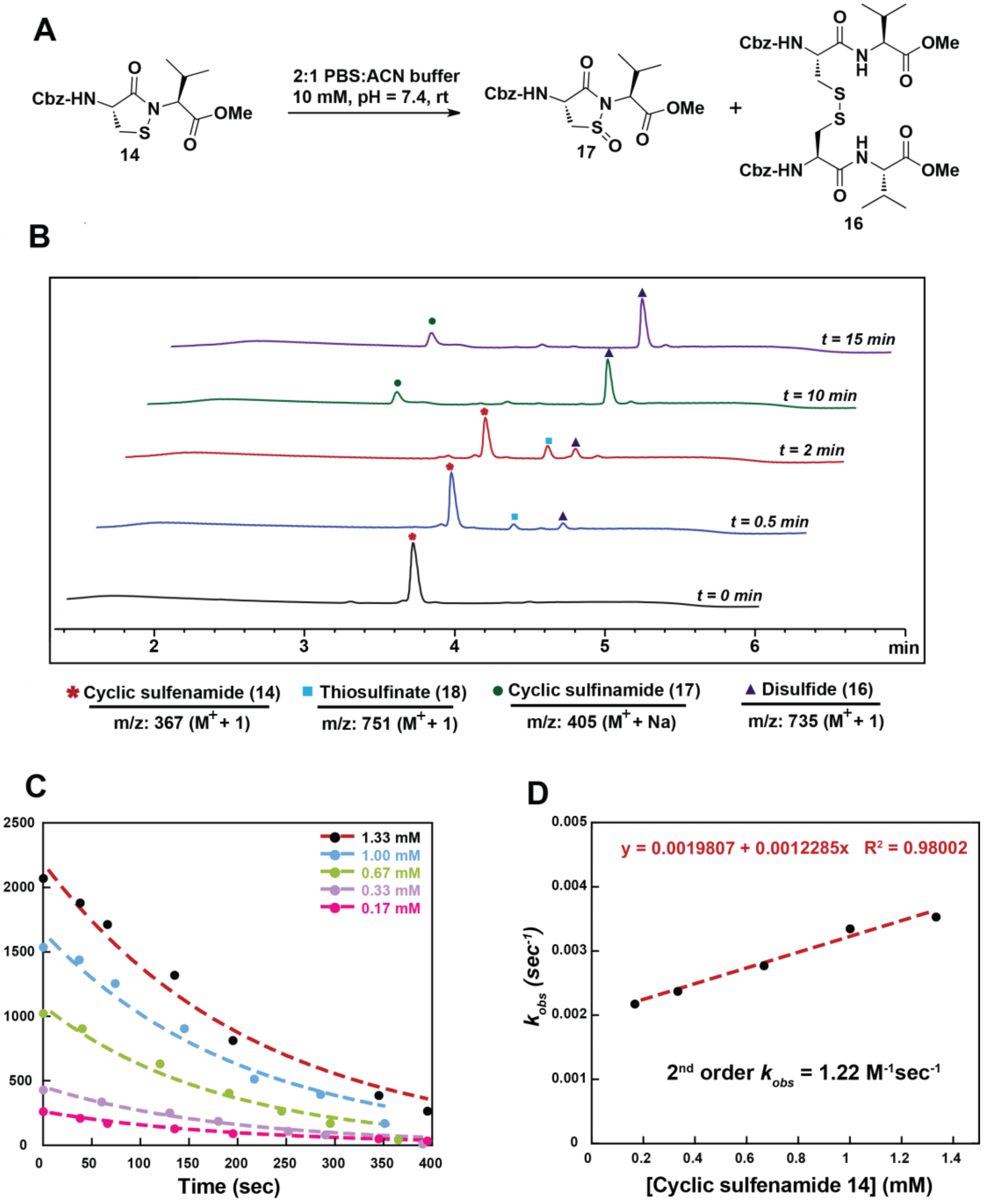

Scheme S2. Study of the kinetics of the self-reaction of dipeptide sulfenamide (14). (A) Reaction pathway showing the adducts formation as a result of the self-reaction of dipeptide cyclic sulfenamide. **(B)** Representative LC traces of self-reaction of dipeptide cyclic sulfenamide at different time points. **(C)** Pseudo 1st order rate constants for the self-reaction of **14** at varying concentrations (166.67 µM – 1.33 mM) were obtained. **(D)** k_{obs} at different concentrations of **14** were plotted to give the 2nd order rate constant value of 1.22 $M^{-1}s^{-1}$.

Scheme S3. Stability of cyclic sulfenamide (14) in acetonitrile. A 1 mM solution of **14** was prepared in acetonitrile and its stability over the time was evaluated by LC-MS. As seen from the data above, **14** is completely stable under anhydrous conditions.

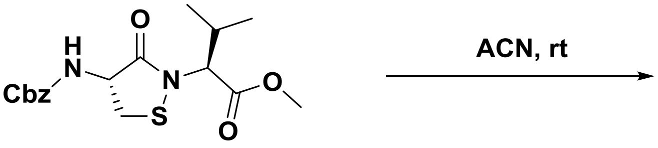

14 (1 mM in ACN)

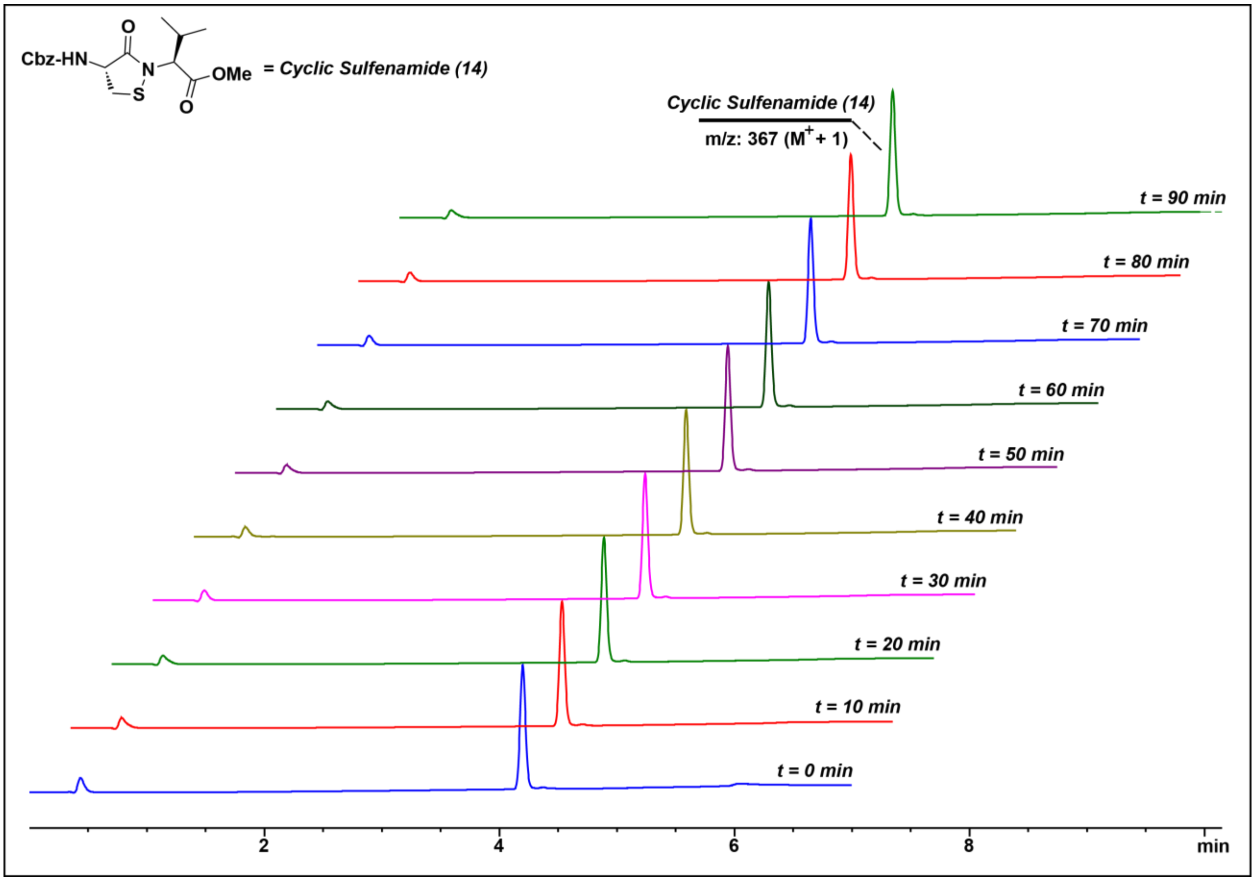

Scheme S4. Reaction of cyclic sulfenamide 14 (1 mM) with methyl iodide (1 M) under PBS:ACN (10 mM, 2:1, pH 7.4, rt) conditions.

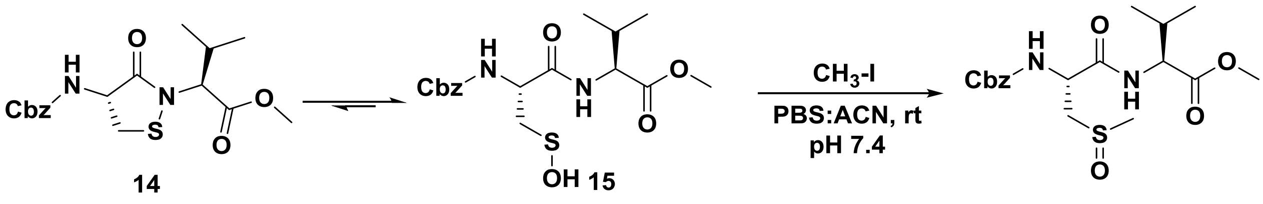

LC-MS trace

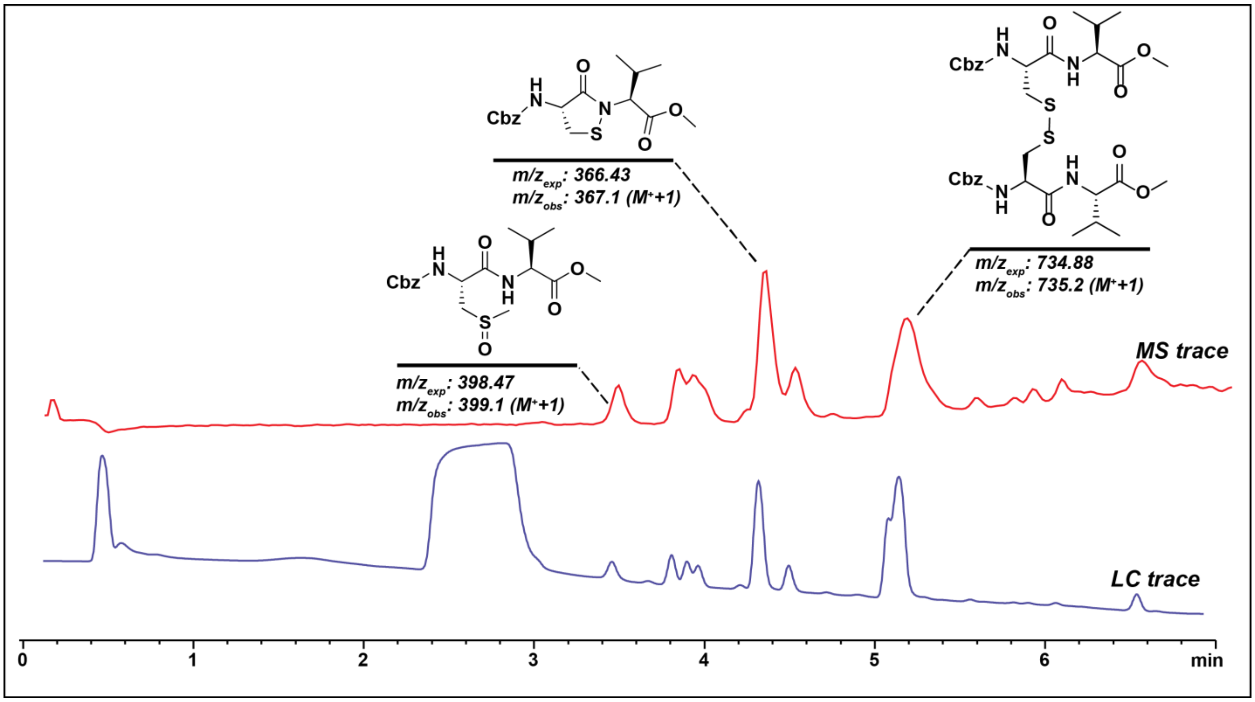

MS trace

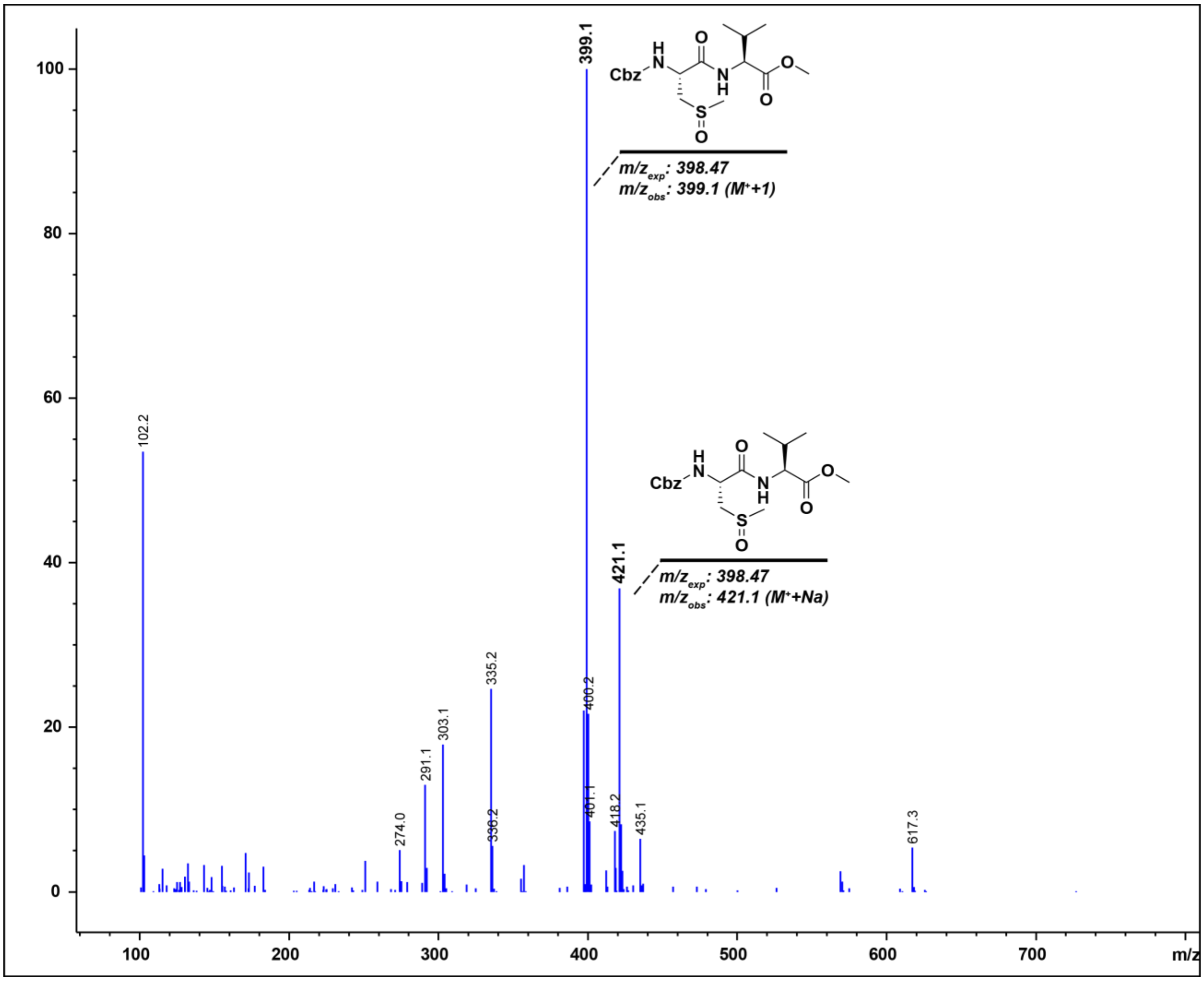

399.1
Cbz
H
N
O
N
H
O
O
S
O
m/z$_{exp}$: 398.47
m/z$_{obs}$: 399.1 (M$^+$+1)
421.1
m/z$_{exp}$: 398.47
m/z$_{obs}$: 421.1 (M$^+$+Na)
102.2
335.2
303.1
291.1
274.0
336.2
400.2
401.1
418.2
435.1
617.3
100
80
60
40
20
0
100
200
300
400
500
600
700
m/z

Scheme S5. Reaction of cyclic sulfenamide 14 (1 mM) with NBD-Cl (1 mM) under PBS:ACN (10 mM, 2:1, pH 7.4, rt) conditions.

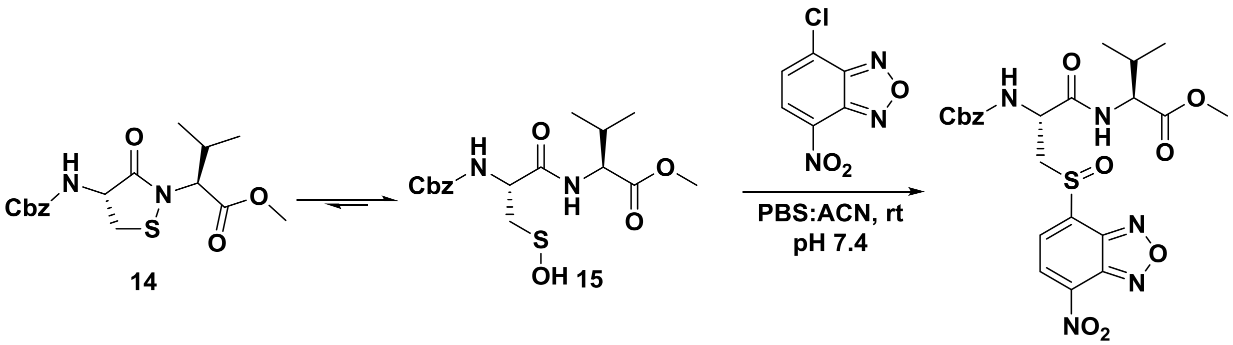

LC traces over 2 days

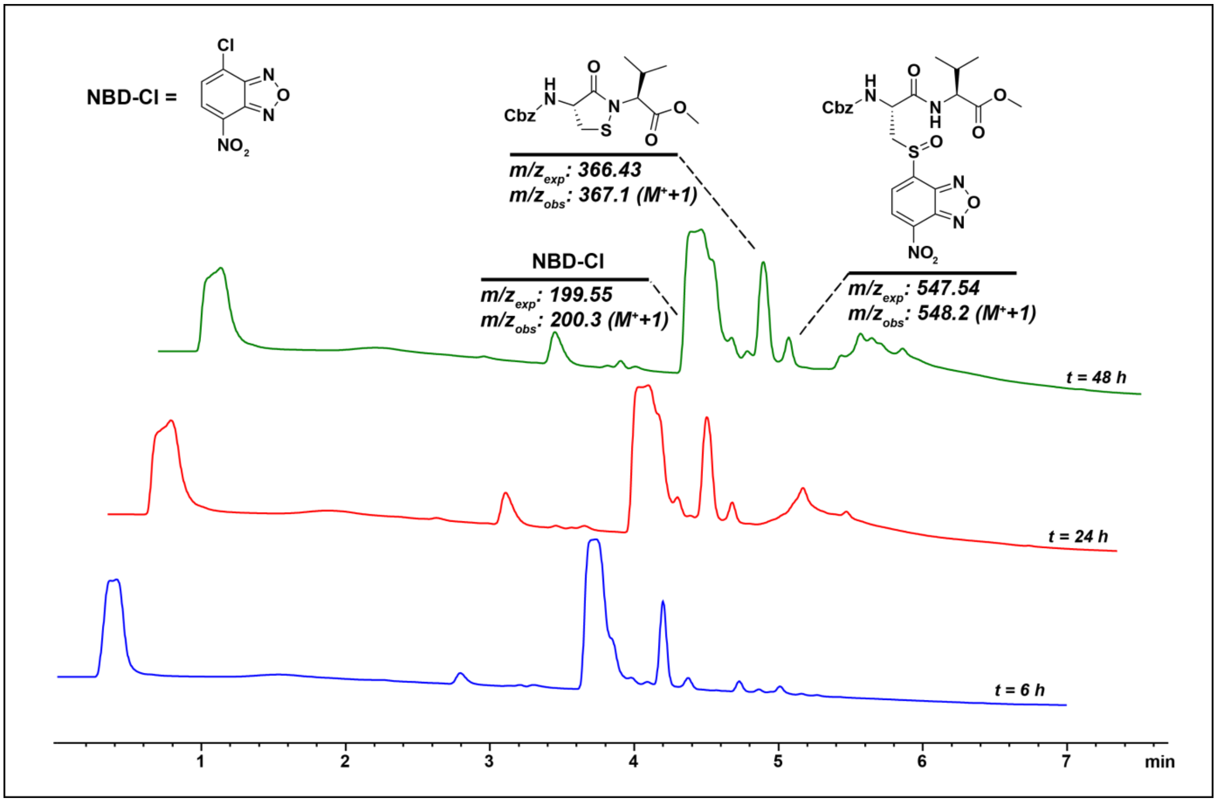

MS trace:

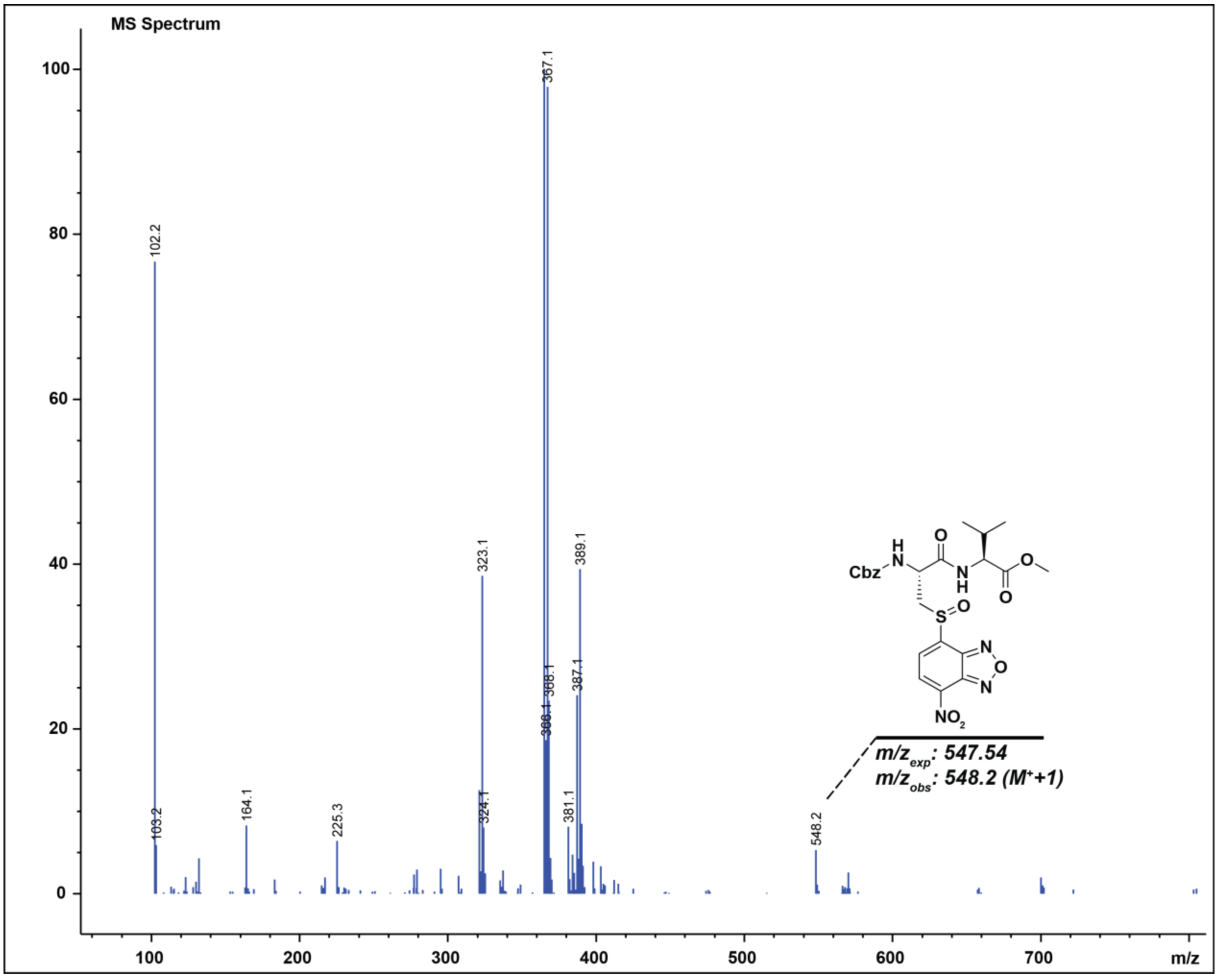

MS Spectrum
100
80
60
40
20
0
102.2
103.2
164.1
225.3
323.1
324.1
366.1
367.1
368.1
381.1
387.1
389.1
548.2
100
200
300
400
500
600
700
m/z
Cbz
H
N
O
O
S
N
O
N
NO2
m/zexp: 547.54
m/zobs: 548.2 (M++1)

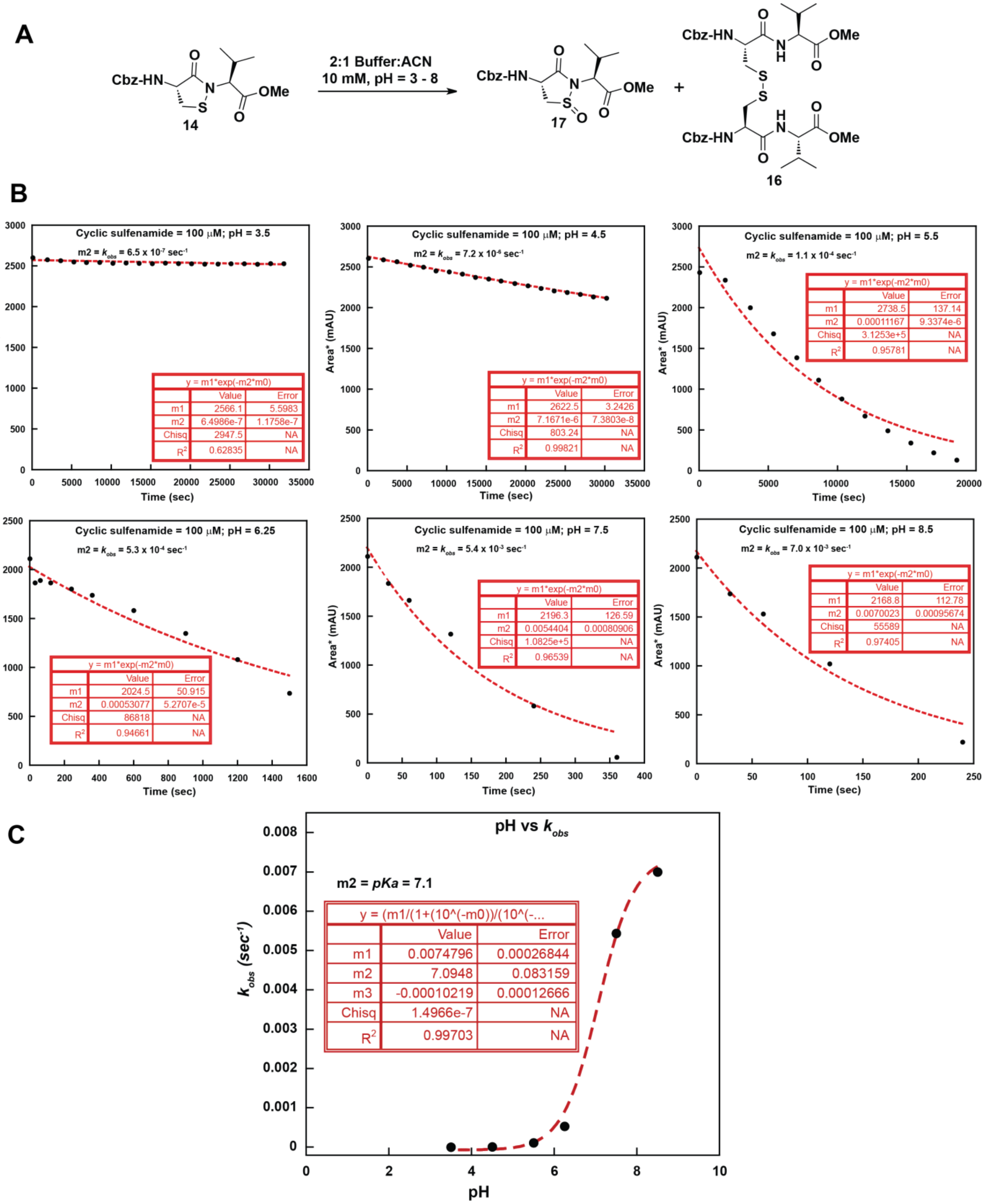

Scheme S6. (A) pH dependence of the self-reaction of dipeptide cyclic sulfenamide **14** was studied. **(B)** Pseudo 1st order k_{obs} were obtained at pH 3.5 – 8.5; **(C)** A plot of pH vs kobs provided a sigmoid curve. LogIC$_{50}$ value obtained was 7.1 which correspond to the pK$_a$ of sulfenic acid **15**.

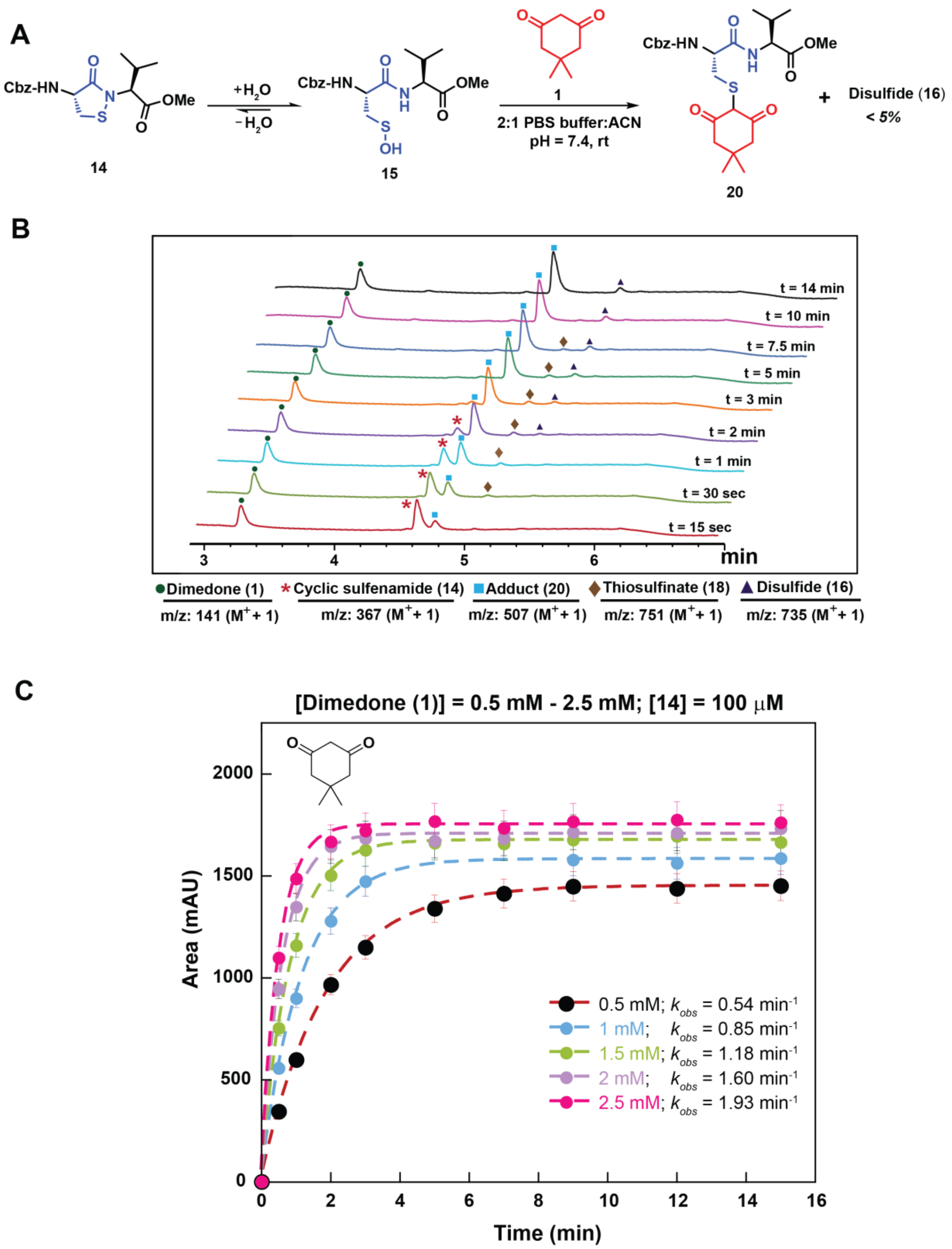

Scheme S7. Study of the kinetics of the reaction of dimedone **(1)** with dipeptide sulfenic acid **(15)**. **(A)** Reaction pathway showing thioether adduct **(20)** formation as a result of the reaction of **1** with **15**. **(B)** Representative LC traces of the reaction of **1** with **15** at different time points. **(C)** Pseudo 1st order k_{obs} for the reaction of **14** with varying concentrations (0.5 mM – 2.5 mM) of **1** were obtained.

Scheme S8. Reaction of cyclic phenyl sulfenamide **(41)** with dimedone **(1)** under aqueous conditions.

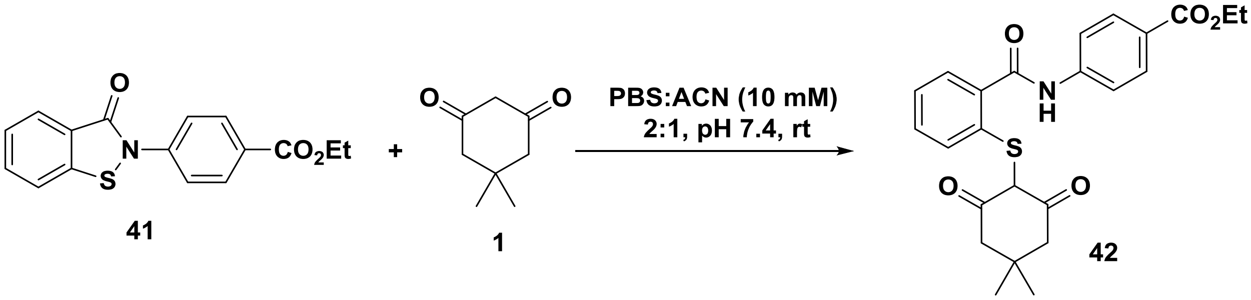

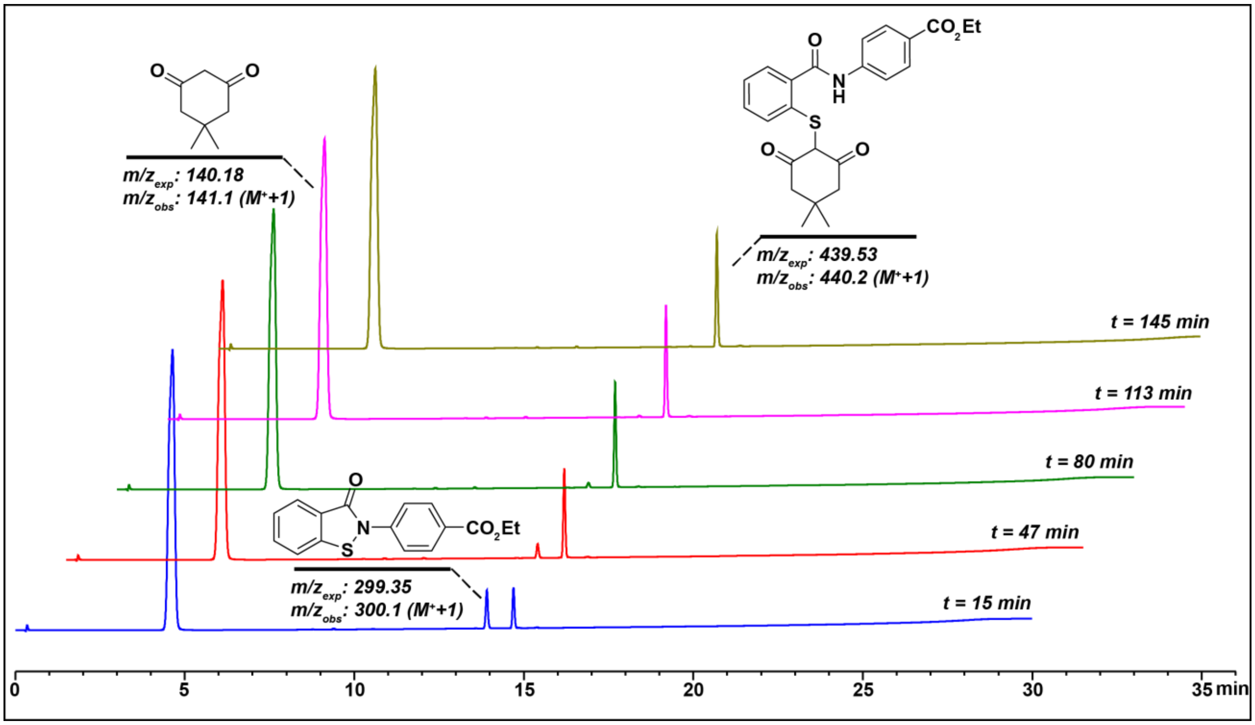

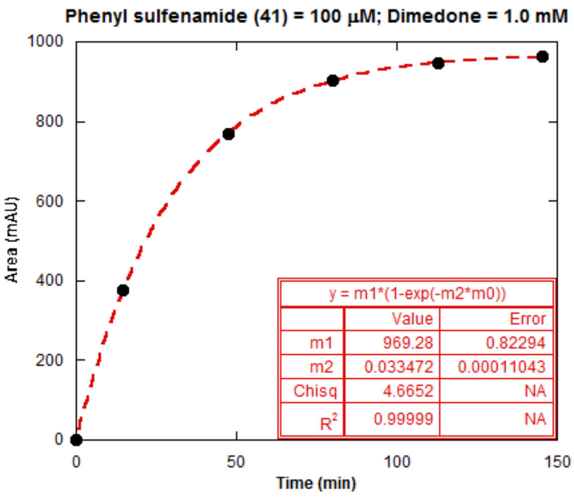

Scheme S9. Stability/decomposition profile of cyclic phenyl sulfenamide **(41)** under aqueous conditions.

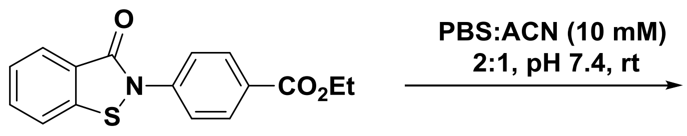

41

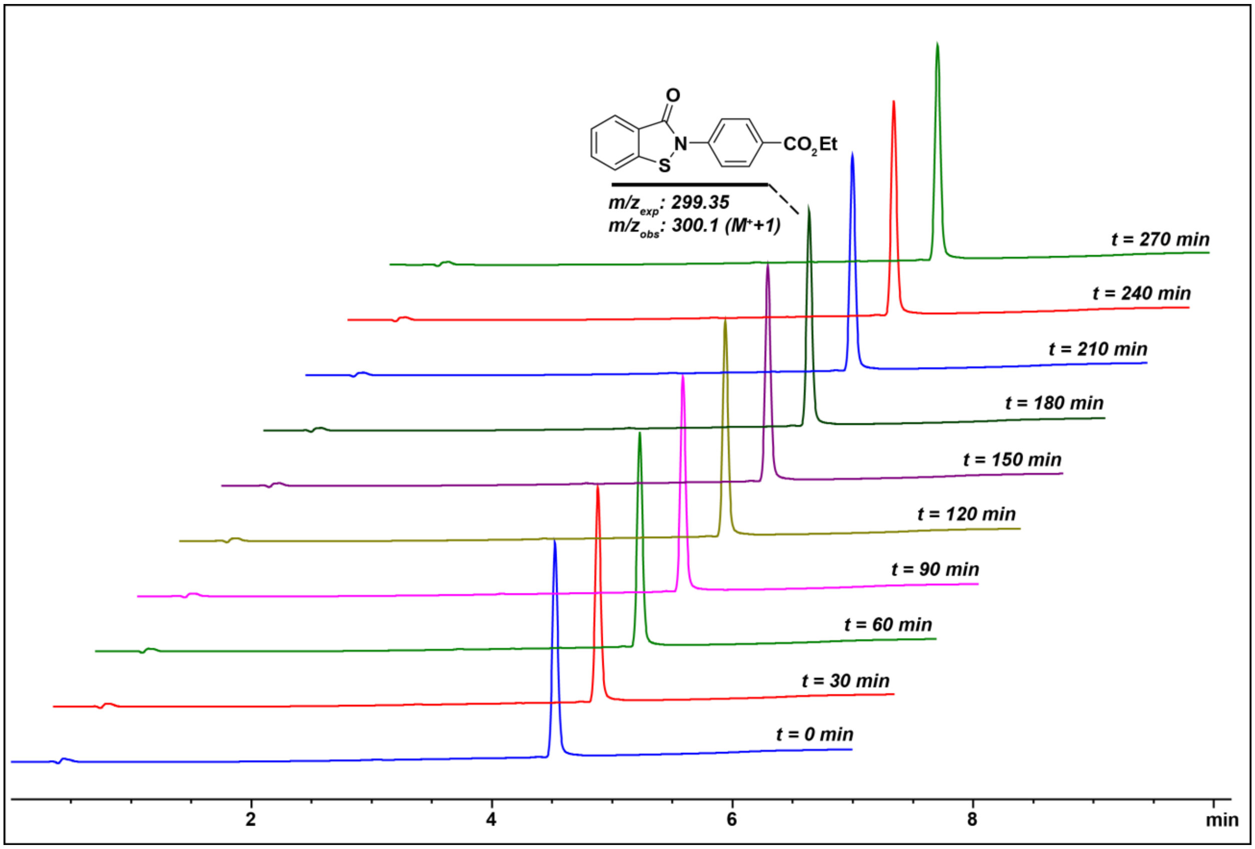

Scheme S10. Reaction of linear phenyl sulfenamide **(43)** with dimedone **(1)** under aqueous conditions.

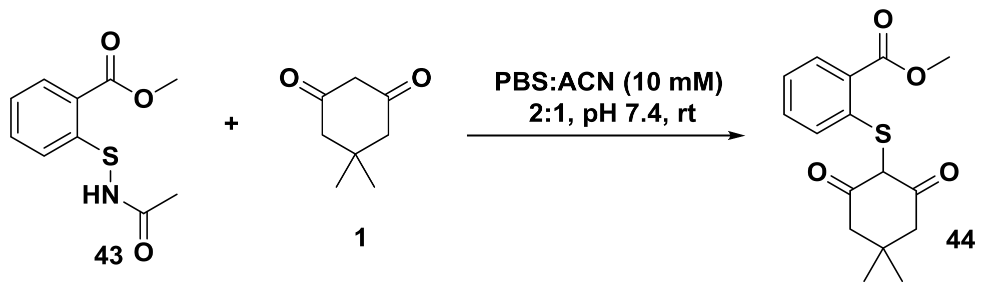

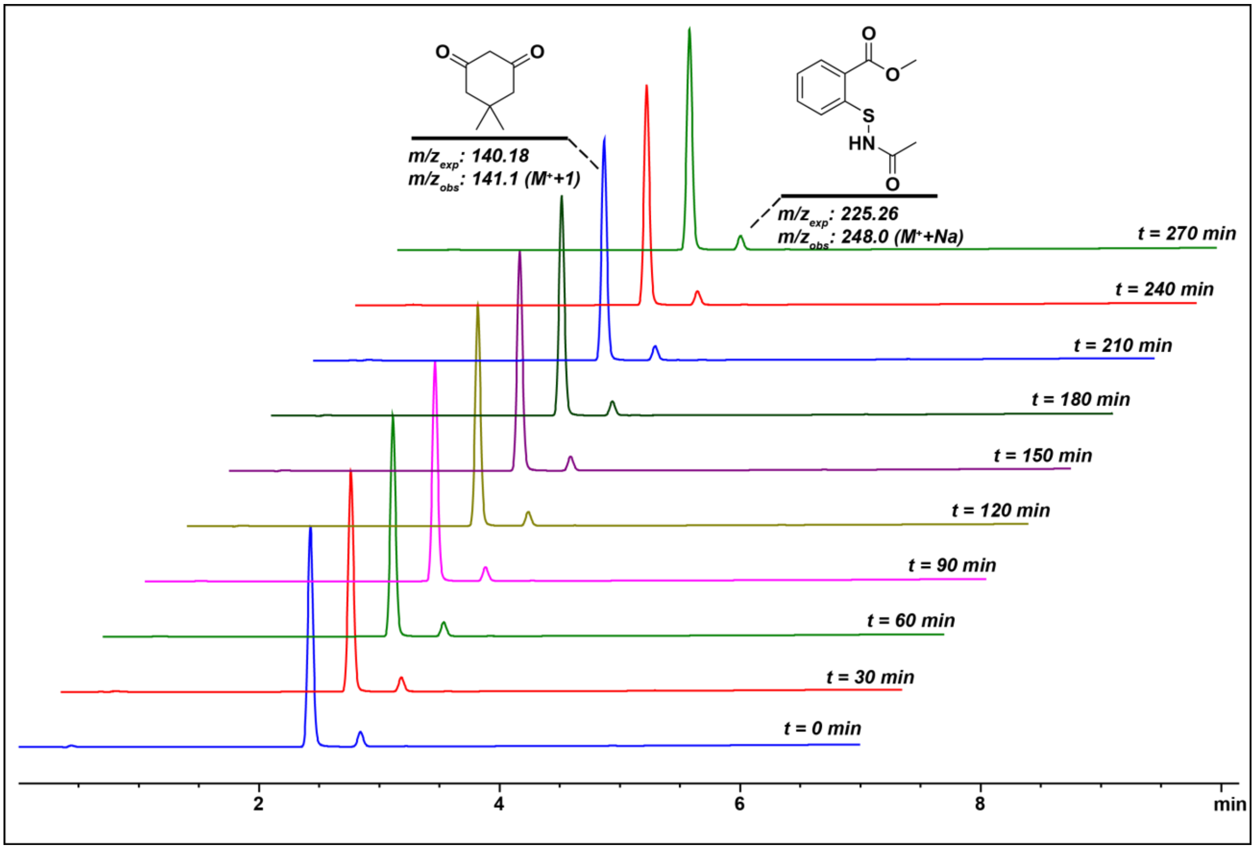

Scheme S11. Stability/decomposition profile of linear phenyl sulfenamide **(43)** under aqueous conditions.

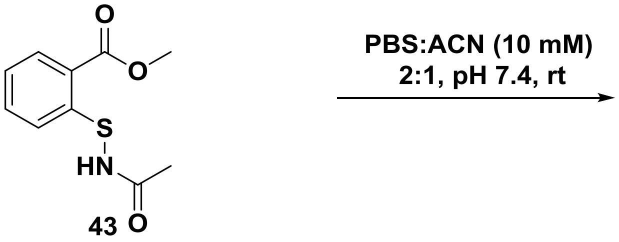

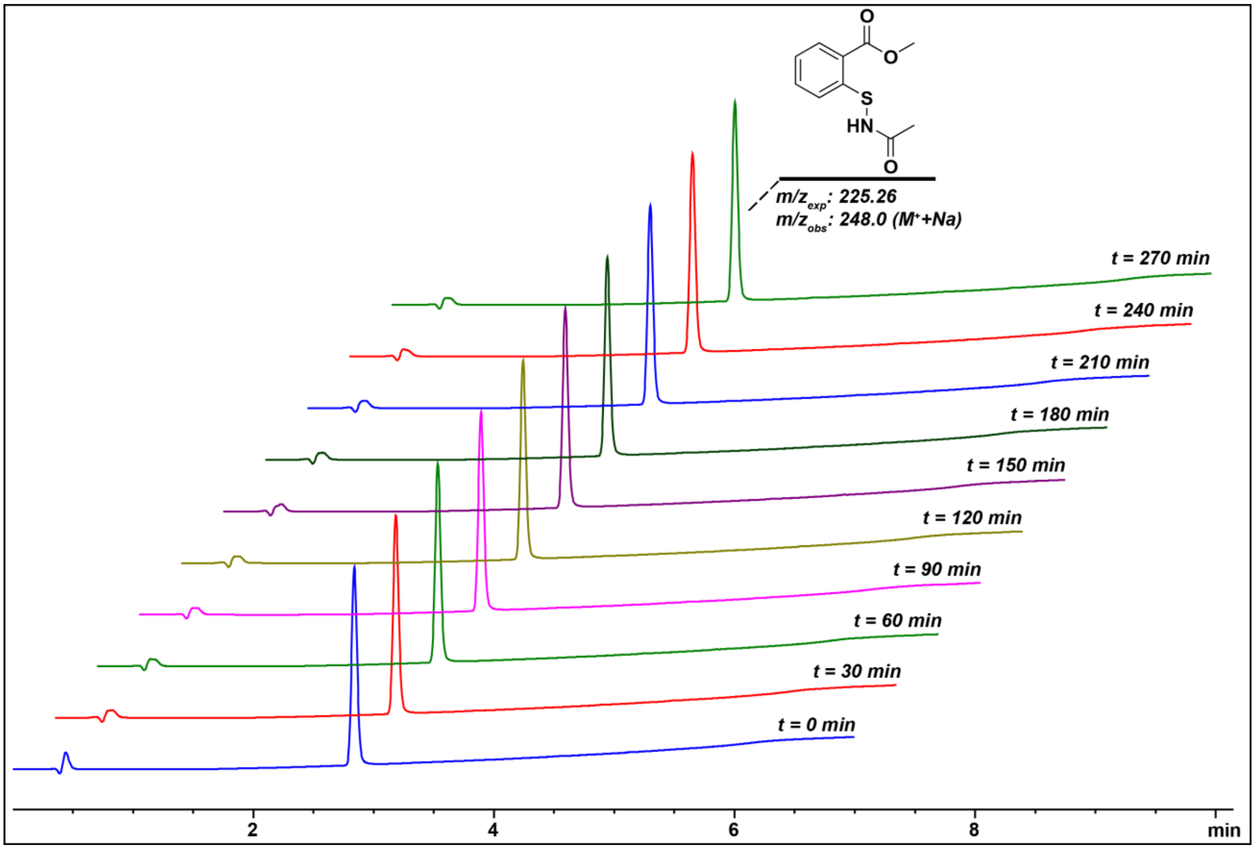

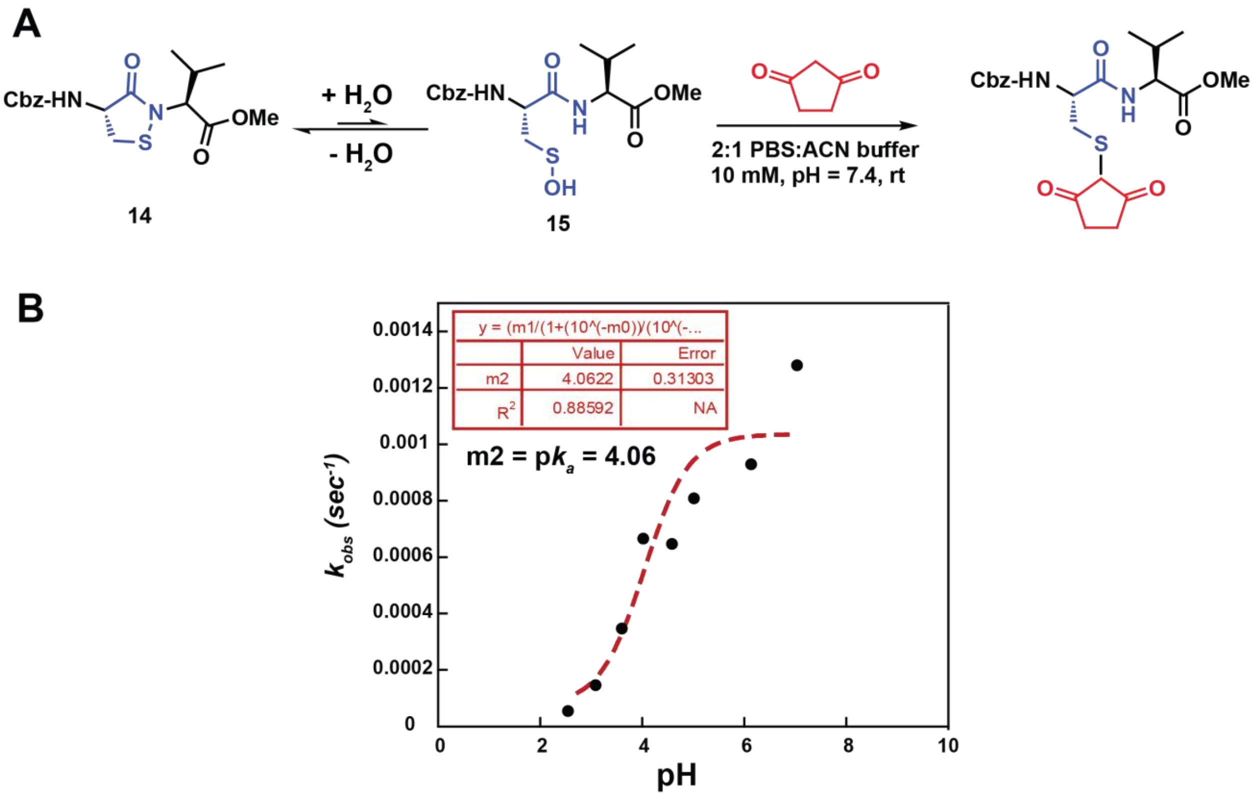

Scheme S12. pH dependence of the reaction of dipeptide cyclic sulfenamide **14** with 1,3-cyclopentanedione **(21a)** was studied. Pseudo 1st order k_{obs} obtained at different pHs were plotted against pH to obtain the sigmoid plots. $LogIC_{50}$ value obtained was 4.1 which correspond to the pK_a.

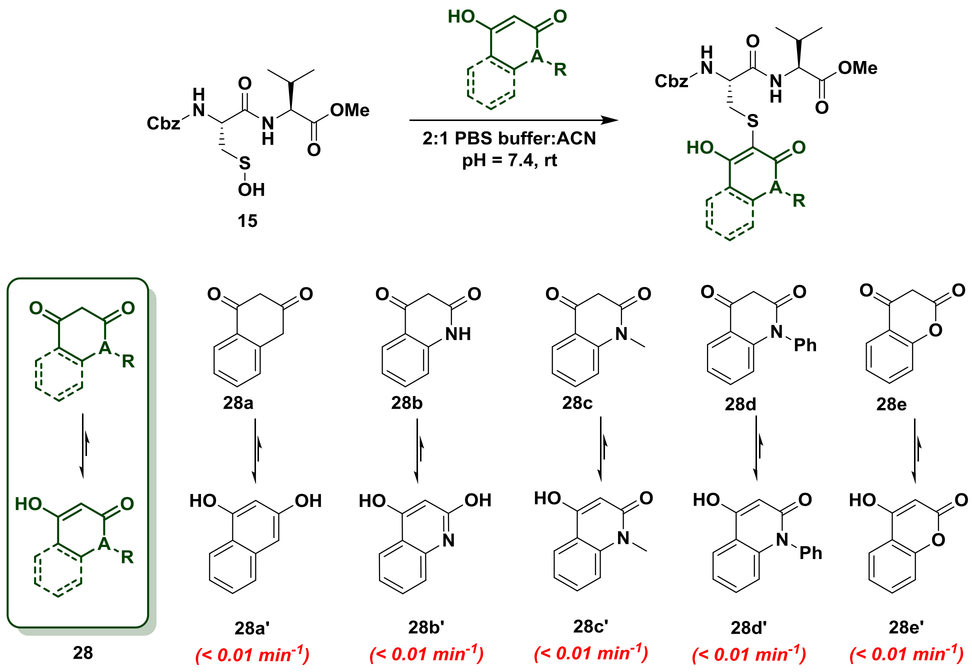

Chart S1. Reaction of dipeptide sulfenic acid **15** with nucleophiles - Effect of complete enolization.

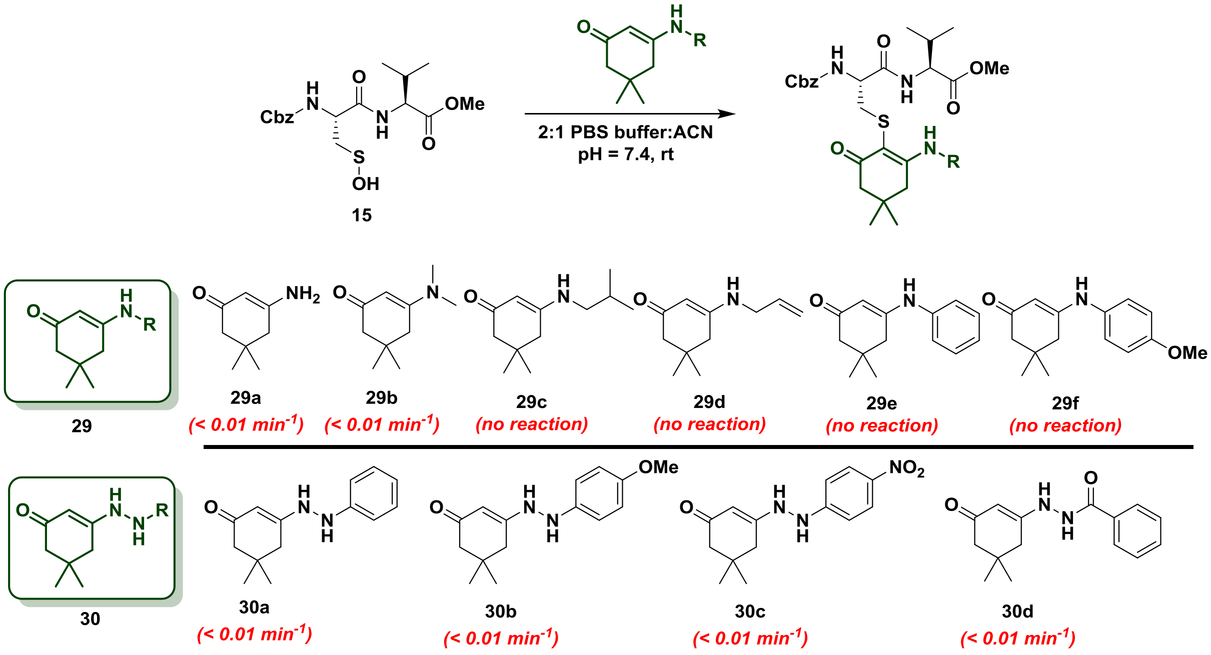

Chart S2. Reaction of dipeptide sulfenic acid **15** with enamines and hydrazides based nucleophiles.

Chart 1 – Rate Plots

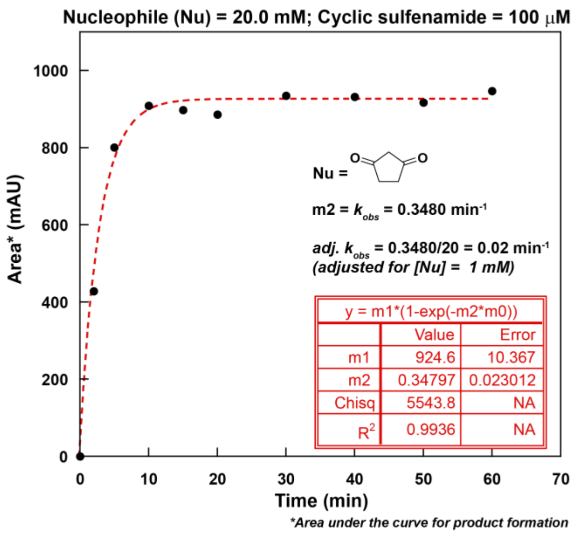

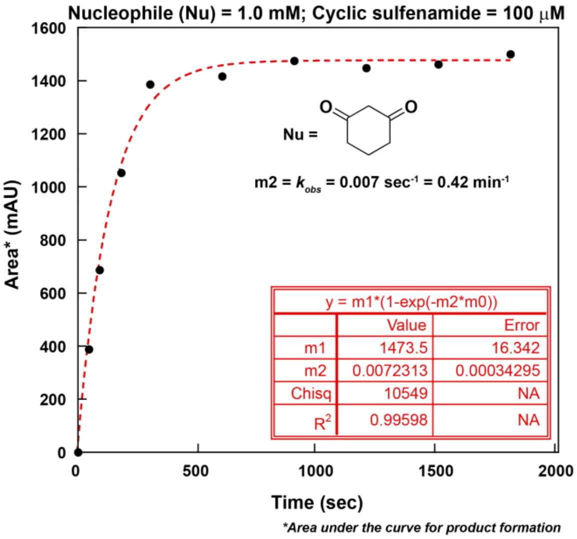

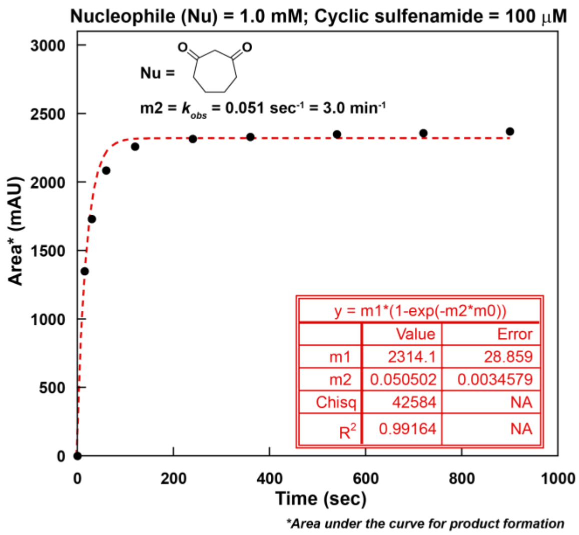

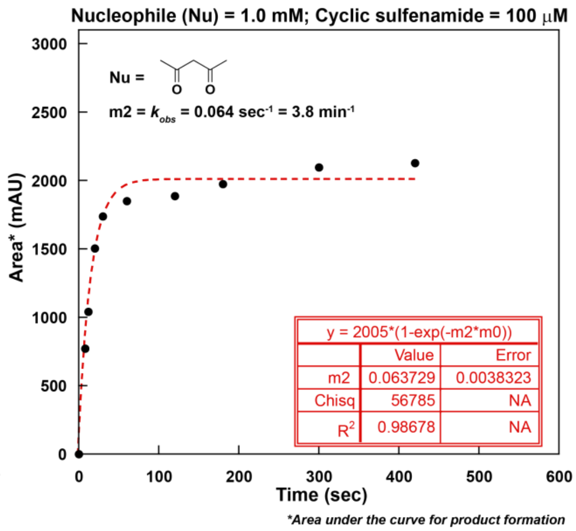

Chart 2 – Rate Plots

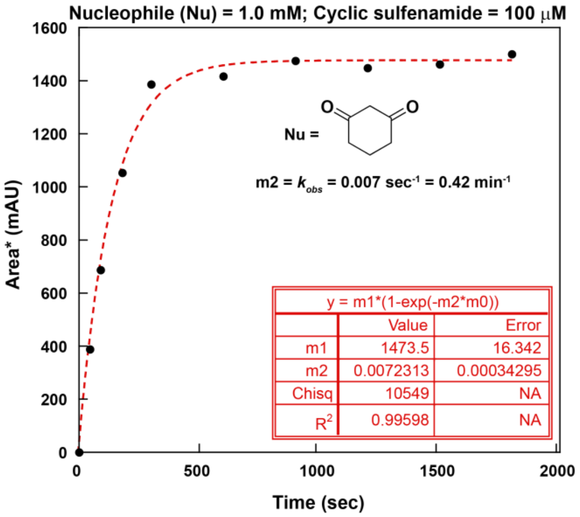

*Area under the curve for product formation

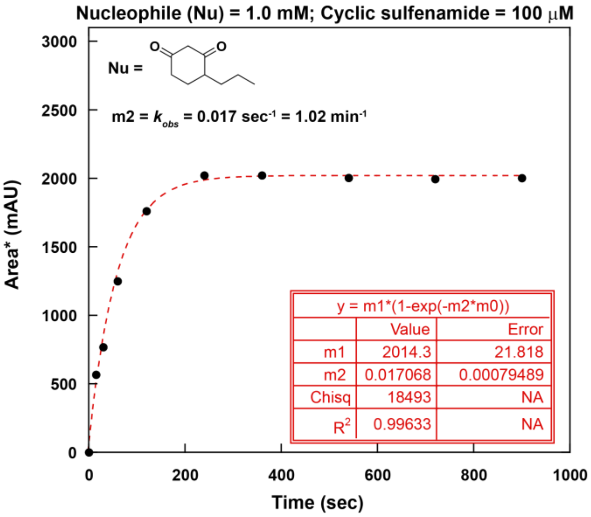

*Area under the curve for product formation

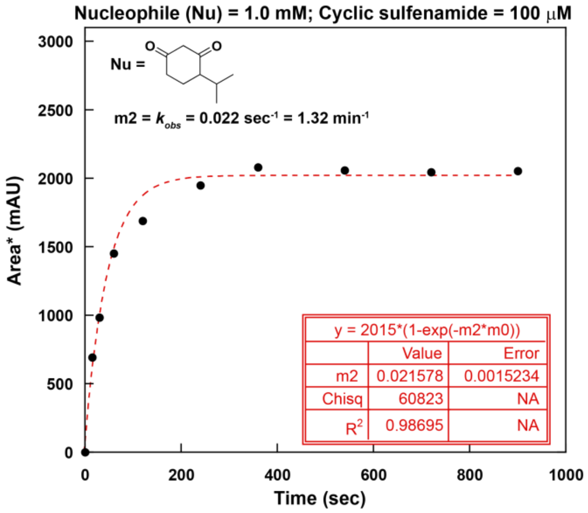

*Area under the curve for product formation

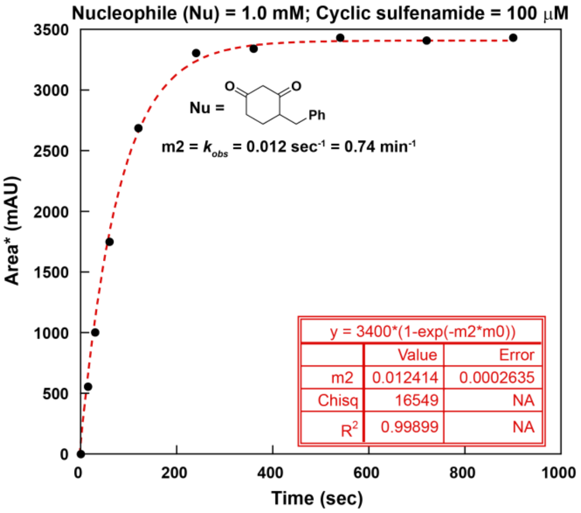

*Area under the curve for product formation

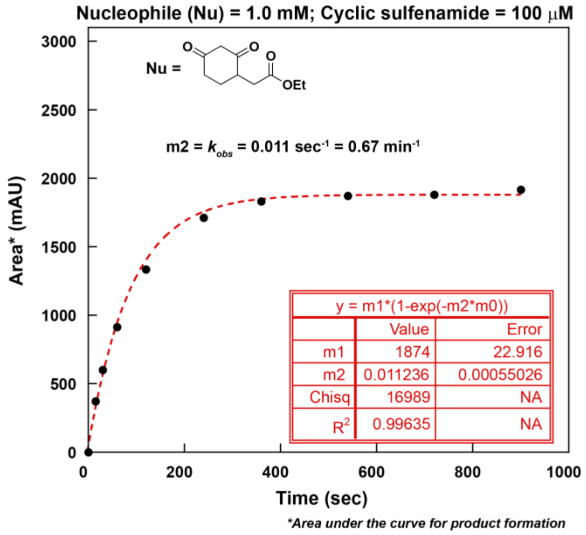
Nucleophile (Nu) = 1.0 mM; Cyclic sulfenamide = 100 μM
Nu =
O
O
O
OEt
m2 = kobs = 0.011 sec-1 = 0.67 min-1
y = m1*(1-exp(-m2*m0))
Value Error
m1 1874 22.916
m2 0.011236 0.00055026
Chisq 16989 NA
R2 0.99635 NA
Area* (mAU)
Time (sec)
*Area under the curve for product formation

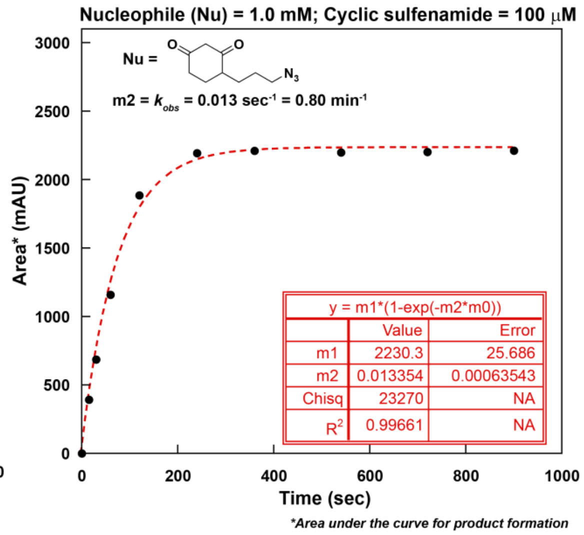
Nucleophile (Nu) = 1.0 mM; Cyclic sulfenamide = 100 μM
Nu =
O
O
N3
m2 = kobs = 0.013 sec-1 = 0.80 min-1
y = m1*(1-exp(-m2*m0))
Value Error
m1 2230.3 25.686
m2 0.013354 0.00063543
Chisq 23270 NA
R2 0.99661 NA
Area* (mAU)
Time (sec)
*Area under the curve for product formation

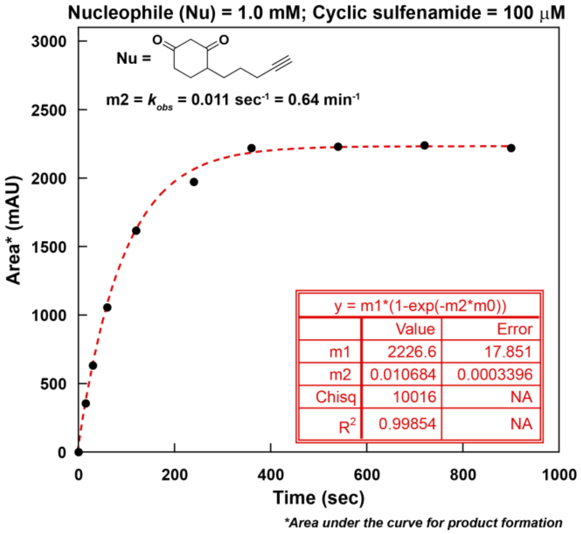
Nucleophile (Nu) = 1.0 mM; Cyclic sulfenamide = 100 μM
Nu =
O
O
m2 = kobs = 0.011 sec-1 = 0.64 min-1
y = m1*(1-exp(-m2*m0))
Value Error
m1 2226.6 17.851
m2 0.010684 0.0003396
Chisq 10016 NA
R2 0.99854 NA
Area* (mAU)
Time (sec)
*Area under the curve for product formation

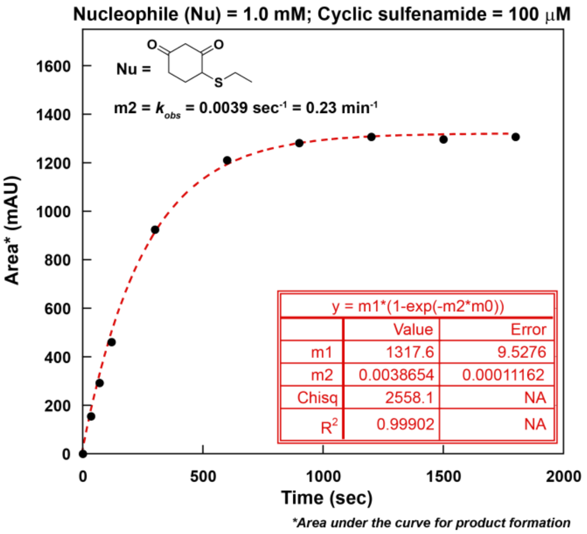
Nucleophile (Nu) = 1.0 mM; Cyclic sulfenamide = 100 μM
Nu =
O
O
S
m2 = kobs = 0.0039 sec-1 = 0.23 min-1
y = m1*(1-exp(-m2*m0))
Value Error
m1 1317.6 9.5276
m2 0.0038654 0.00011162
Chisq 2558.1 NA
R2 0.99902 NA
Area* (mAU)
Time (sec)
*Area under the curve for product formation

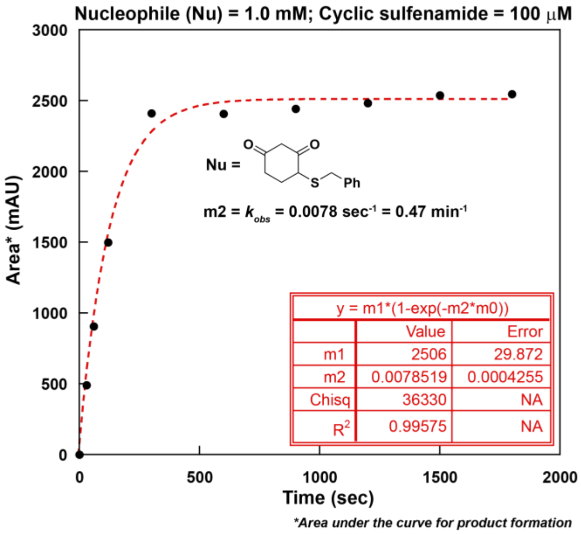
Nucleophile (Nu) = 1.0 mM; Cyclic sulfenamide = 100 μM
Nu =
O
O
S
Ph
m2 = kobs = 0.0078 sec-1 = 0.47 min-1
y = m1*(1-exp(-m2*m0))
Value Error
m1 2506 29.872
m2 0.0078519 0.0004255
Chisq 36330 NA
R2 0.99575 NA
Area* (mAU)
Time (sec)
*Area under the curve for product formation

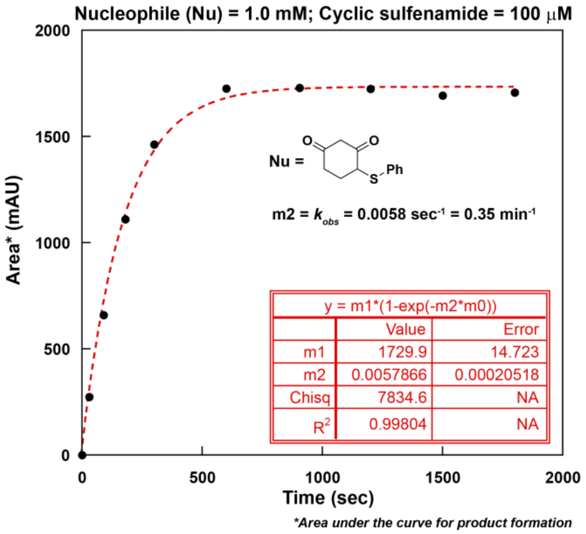
Nucleophile (Nu) = 1.0 mM; Cyclic sulfenamide = 100 μM
Nu =
O
O
S
Ph
m2 = kobs = 0.0058 sec-1 = 0.35 min-1
y = m1*(1-exp(-m2*m0))
Value Error
m1 1729.9 14.723
m2 0.0057866 0.00020518
Chisq 7834.6 NA
R2 0.99804 NA
Area* (mAU)
Time (sec)
*Area under the curve for product formation

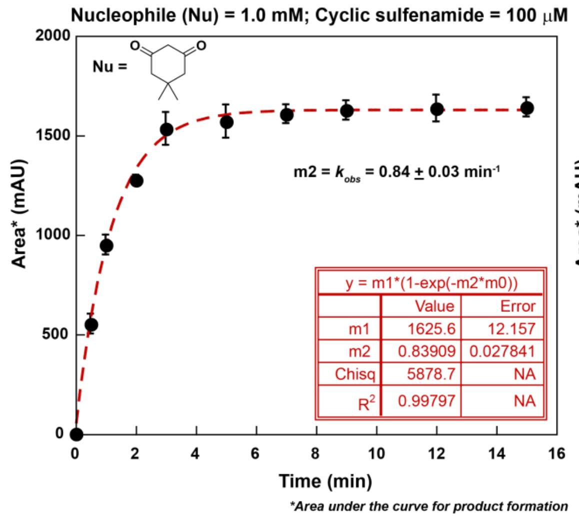
Nucleophile (Nu) = 1.0 mM; Cyclic sulfenamide = 100 µM
Nu =
O
O
m2 = k_obs = 0.84 ± 0.03 min⁻¹
Area* (mAU)
Time (min)
y = m1*(1-exp(-m2*m0))
Value Error
m1 1625.6 12.157
m2 0.83909 0.027841
Chisq 5878.7 NA
R² 0.99797 NA
*Area under the curve for product formation

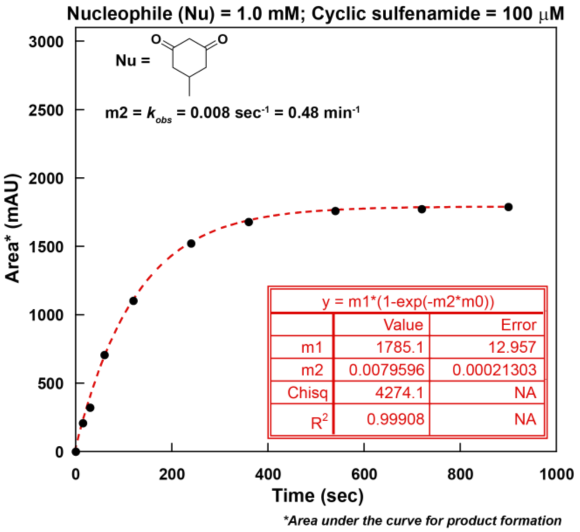
Nucleophile (Nu) = 1.0 mM; Cyclic sulfenamide = 100 µM
Nu =
O
O
m2 = k_obs = 0.008 sec⁻¹ = 0.48 min⁻¹
Area* (mAU)
Time (sec)
y = m1*(1-exp(-m2*m0))
Value Error
m1 1785.1 12.957
m2 0.0079596 0.00021303
Chisq 4274.1 NA
R² 0.99908 NA
*Area under the curve for product formation

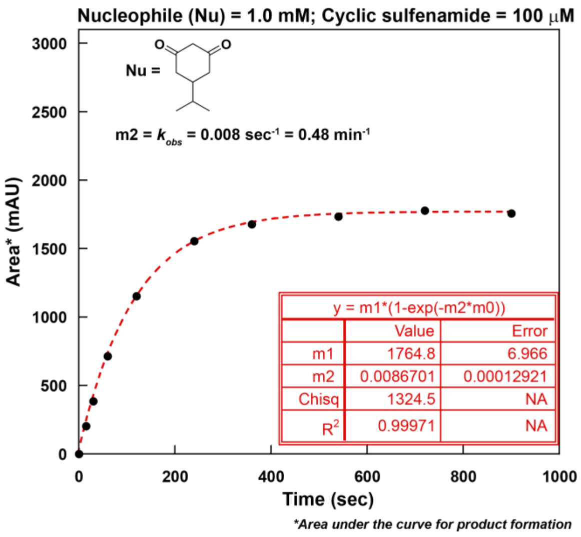
Nucleophile (Nu) = 1.0 mM; Cyclic sulfenamide = 100 µM
Nu =
O
O
m2 = k_obs = 0.008 sec⁻¹ = 0.48 min⁻¹
Area* (mAU)
Time (sec)
y = m1*(1-exp(-m2*m0))
Value Error
m1 1764.8 6.966
m2 0.0086701 0.00012921
Chisq 1324.5 NA
R² 0.99971 NA
*Area under the curve for product formation

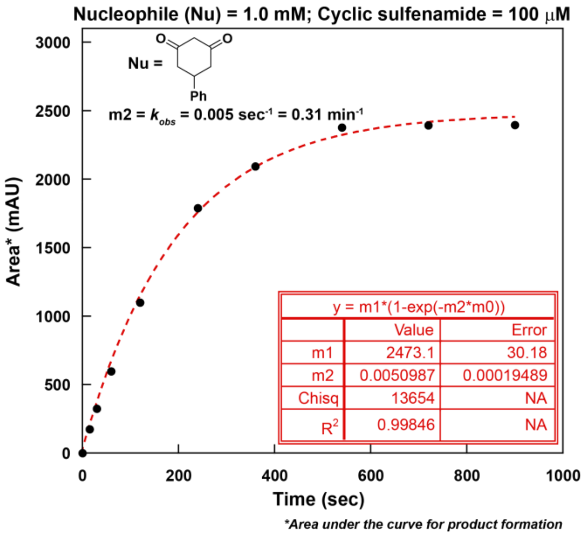
Nucleophile (Nu) = 1.0 mM; Cyclic sulfenamide = 100 µM
Nu =
O
O
Ph
m2 = k_obs = 0.005 sec⁻¹ = 0.31 min⁻¹
Area* (mAU)
Time (sec)
y = m1*(1-exp(-m2*m0))
Value Error
m1 2473.1 30.18
m2 0.0050987 0.00019489
Chisq 13654 NA
R² 0.99846 NA
*Area under the curve for product formation

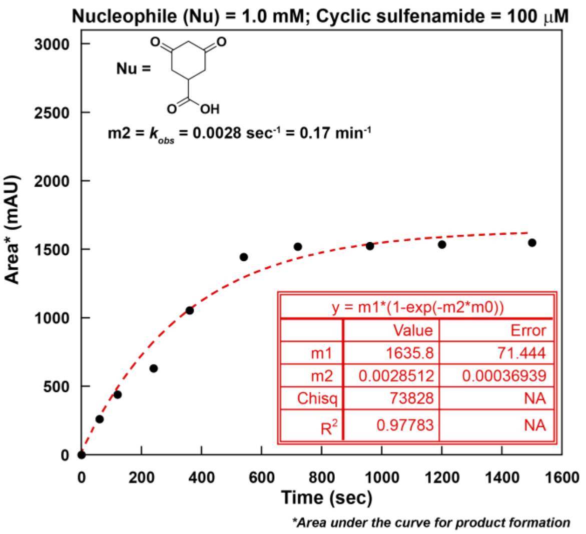
Nucleophile (Nu) = 1.0 mM; Cyclic sulfenamide = 100 µM
Nu =
O
O
O
OH
m2 = k_obs = 0.0028 sec⁻¹ = 0.17 min⁻¹
Area* (mAU)
Time (sec)
y = m1*(1-exp(-m2*m0))
Value Error
m1 1635.8 71.444
m2 0.0028512 0.00036939
Chisq 73828 NA
R² 0.97783 NA
*Area under the curve for product formation

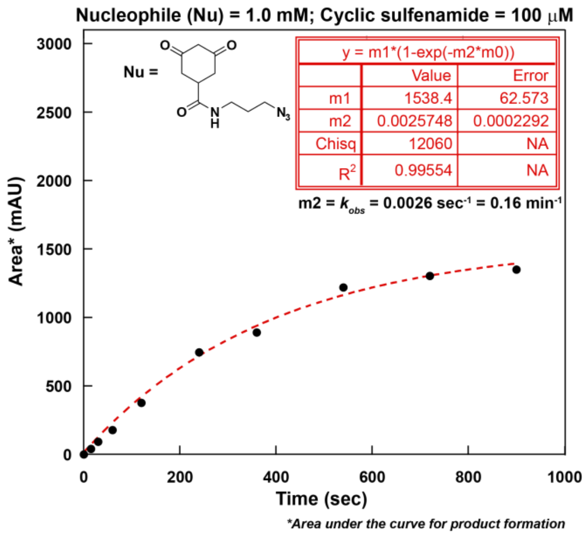
Nucleophile (Nu) = 1.0 mM; Cyclic sulfenamide = 100 µM
Nu =
O
O
O
N
H
N₃
y = m1*(1-exp(-m2*m0))
Value Error
m1 1538.4 62.573
m2 0.0025748 0.0002292
Chisq 12060 NA
R² 0.99554 NA
m2 = k_obs = 0.0026 sec⁻¹ = 0.16 min⁻¹
Area* (mAU)
Time (sec)
*Area under the curve for product formation

Chart 3 – Rate Plots

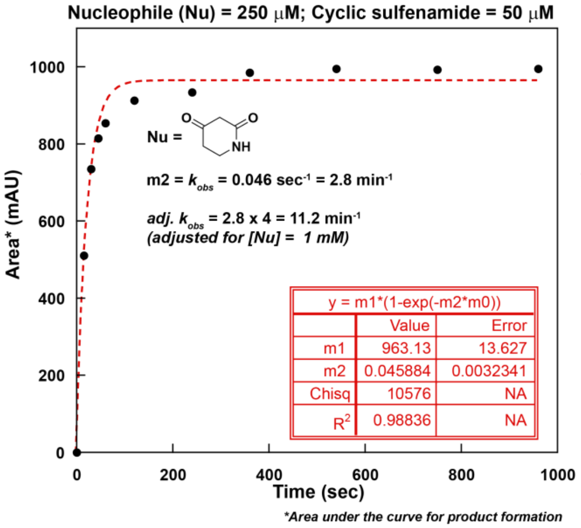

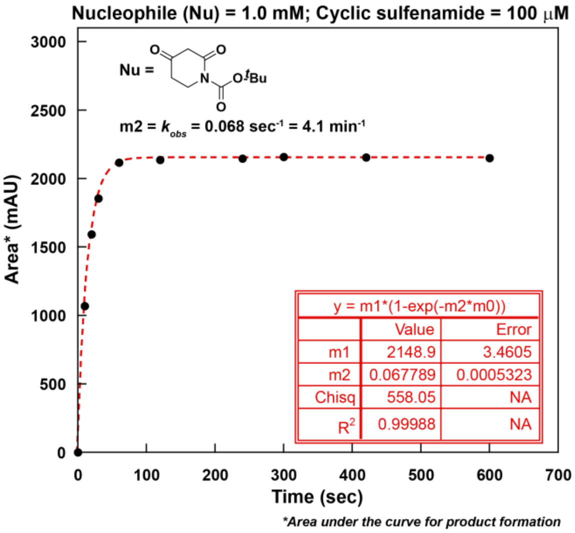

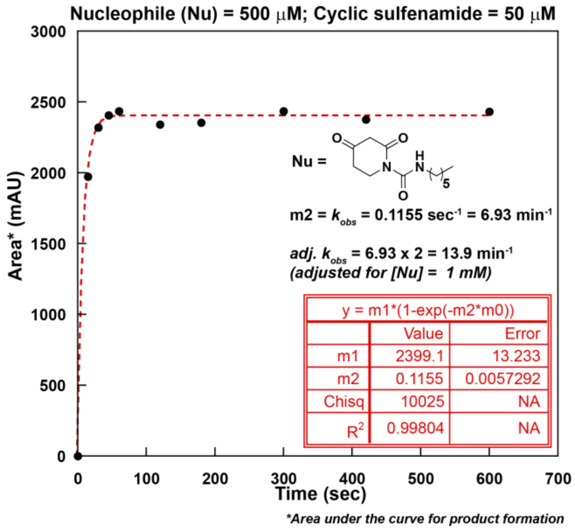

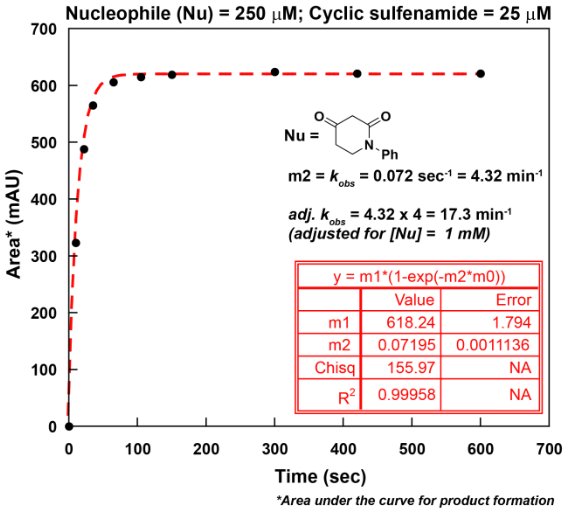

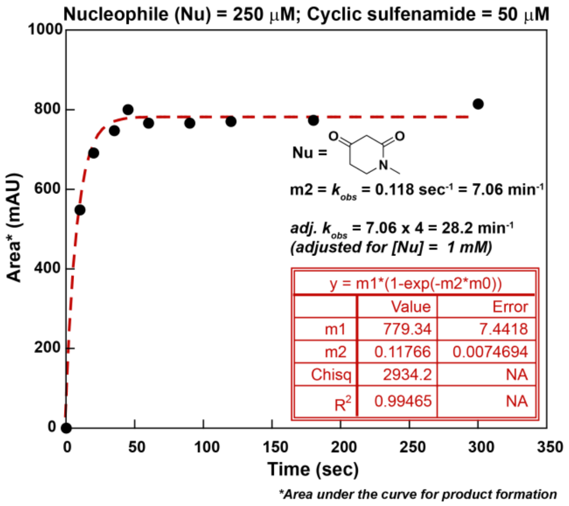
Nucleophile (Nu) = 250 µM; Cyclic sulfenamide = 50 µM
Nu =
m2 = k_obs = 0.118 sec⁻¹ = 7.06 min⁻¹
adj. k_obs = 7.06 x 4 = 28.2 min⁻¹
(adjusted for [Nu] = 1 mM)
y = m1*(1-exp(-m2*m0))
Value Error
m1 779.34 7.4418
m2 0.11766 0.0074694
Chisq 2934.2 NA
R2 0.99465 NA
Area* (mAU)
Time (sec)
*Area under the curve for product formation

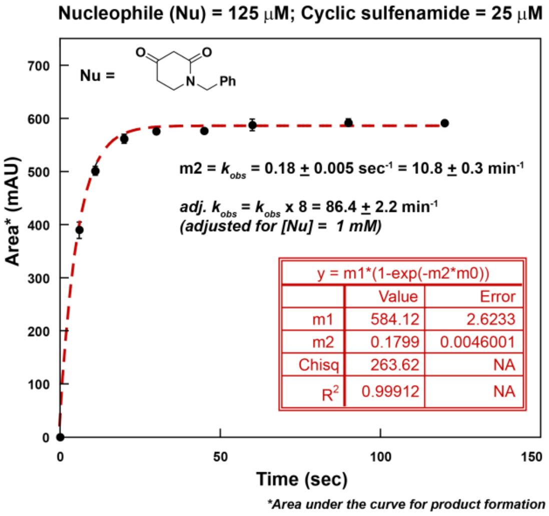
Nucleophile (Nu) = 125 µM; Cyclic sulfenamide = 25 µM
Nu =
Ph
m2 = k_obs = 0.18 ± 0.005 sec⁻¹ = 10.8 ± 0.3 min⁻¹
adj. k_obs = k_obs x 8 = 86.4 ± 2.2 min⁻¹
(adjusted for [Nu] = 1 mM)
y = m1*(1-exp(-m2*m0))
Value Error
m1 584.12 2.6233
m2 0.1799 0.0046001
Chisq 263.62 NA
R2 0.99912 NA
Area* (mAU)
Time (sec)
*Area under the curve for product formation

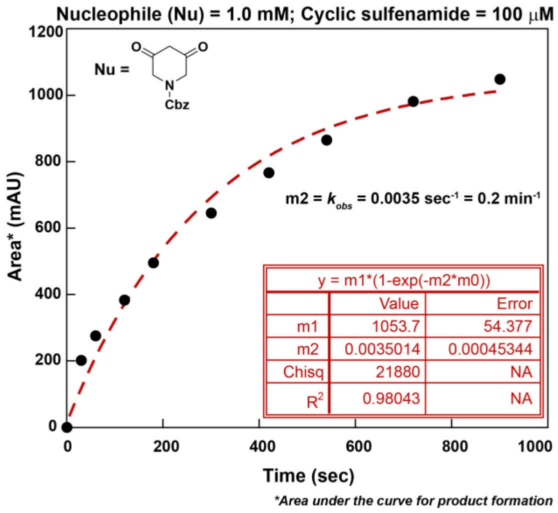
Nucleophile (Nu) = 1.0 mM; Cyclic sulfenamide = 100 µM
Nu =
Cbz
m2 = k_obs = 0.0035 sec⁻¹ = 0.2 min⁻¹
y = m1*(1-exp(-m2*m0))
Value Error
m1 1053.7 54.377
m2 0.0035014 0.00045344
Chisq 21880 NA
R2 0.98043 NA
Area* (mAU)
Time (sec)
*Area under the curve for product formation

Chart 4 – Rate Plots

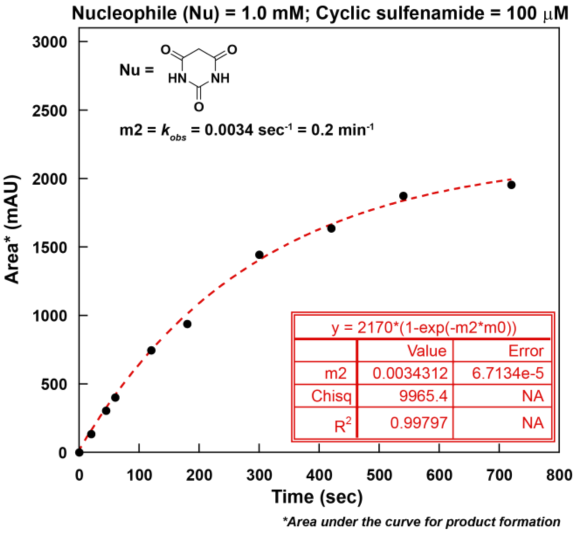

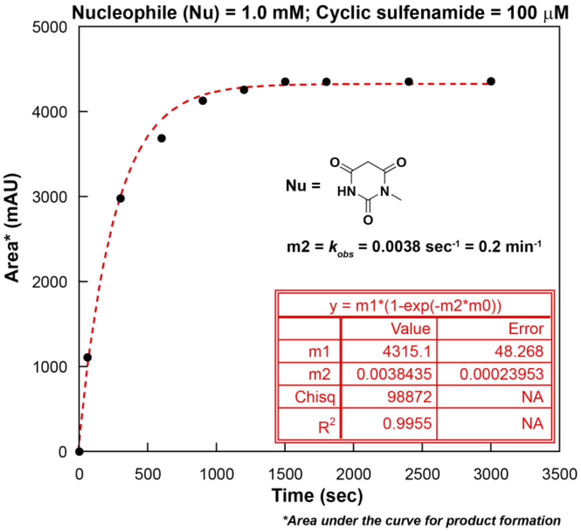

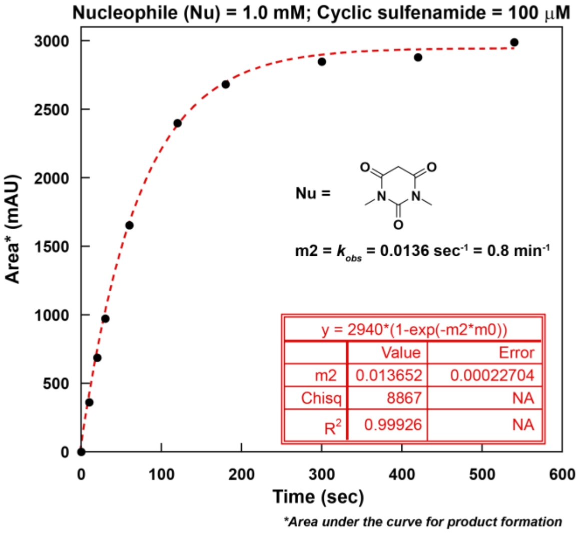

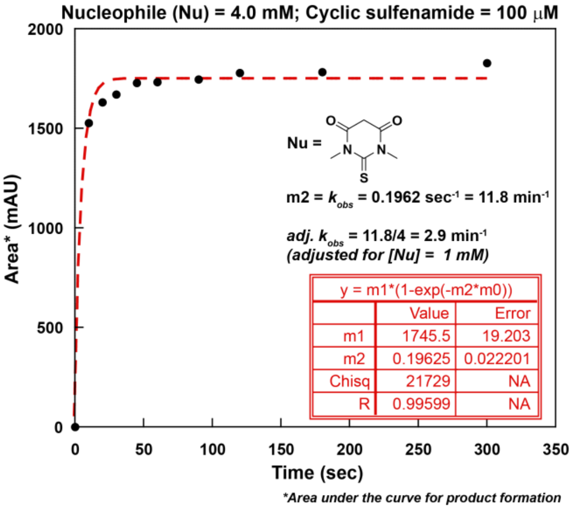

Chart 5 – Rate Plots

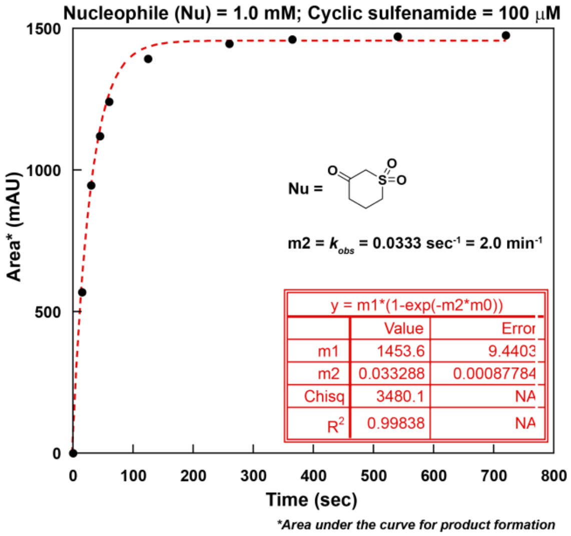

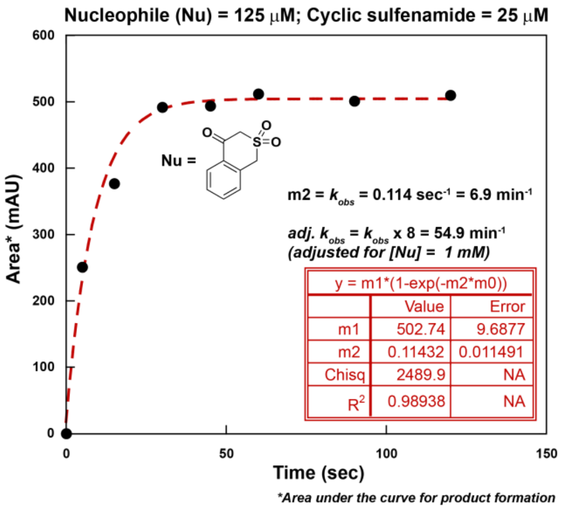

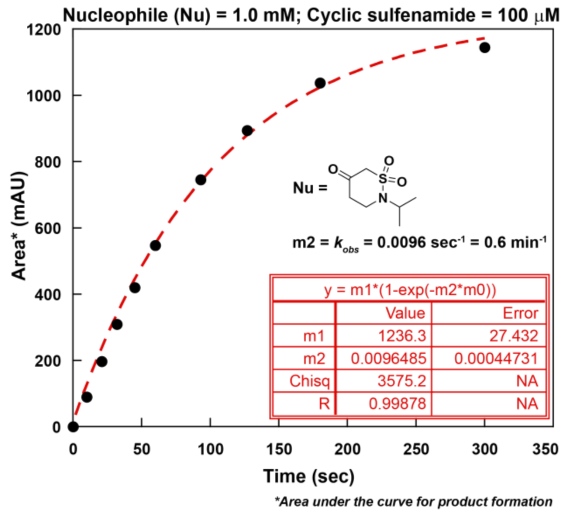

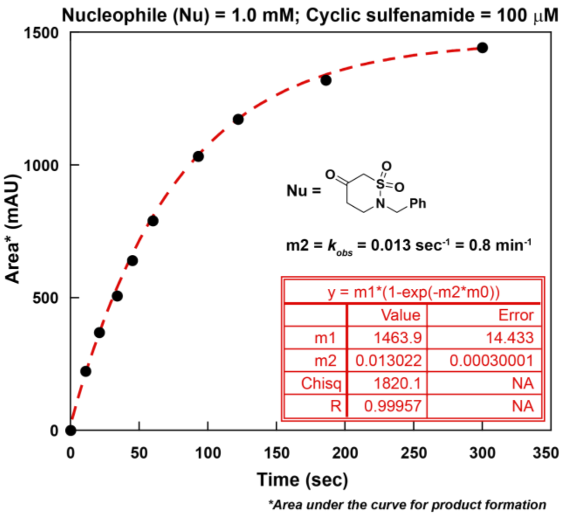

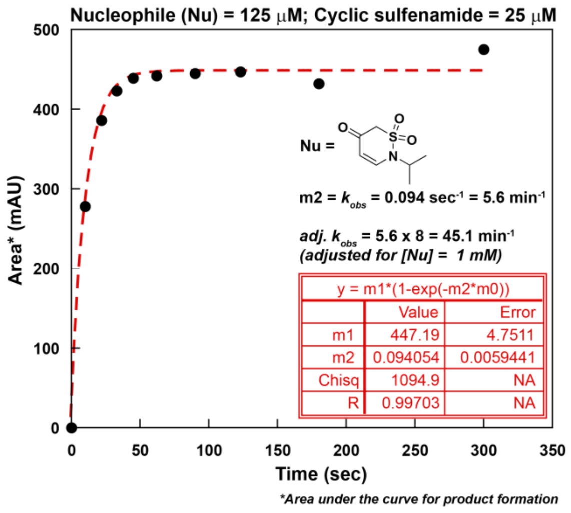

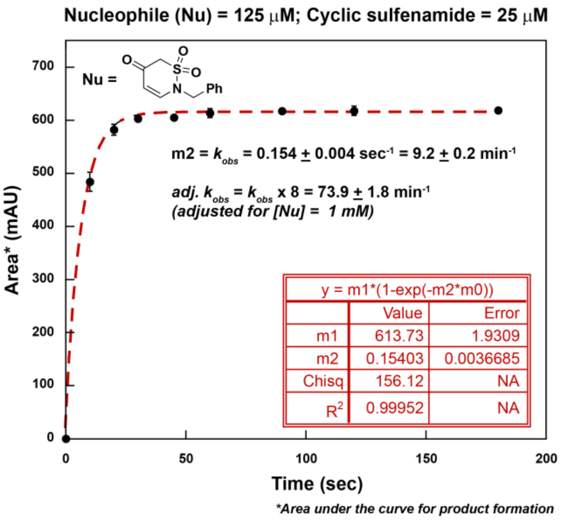

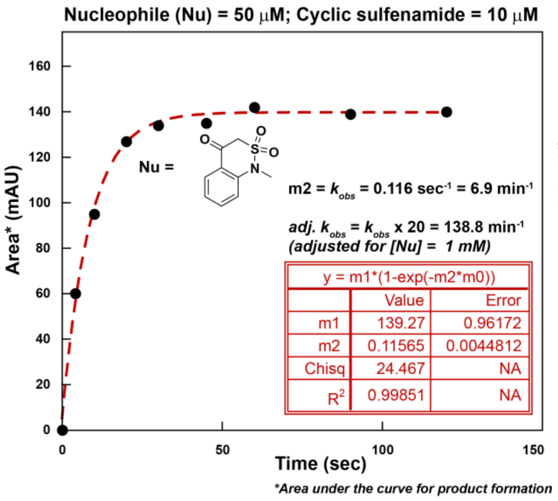
Nucleophile (Nu) = 50 μM; Cyclic sulfenamide = 10 μM
Nu =
m2 = k_obs = 0.116 sec-1 = 6.9 min-1
adj. k_obs = k_obs x 20 = 138.8 min-1
(adjusted for [Nu] = 1 mM)
y = m1*(1-exp(-m2*m0))
Value Error
m1 139.27 0.96172
m2 0.11565 0.0044812
Chisq 24.467 NA
R2 0.99851 NA
Area* (mAU)
Time (sec)
*Area under the curve for product formation

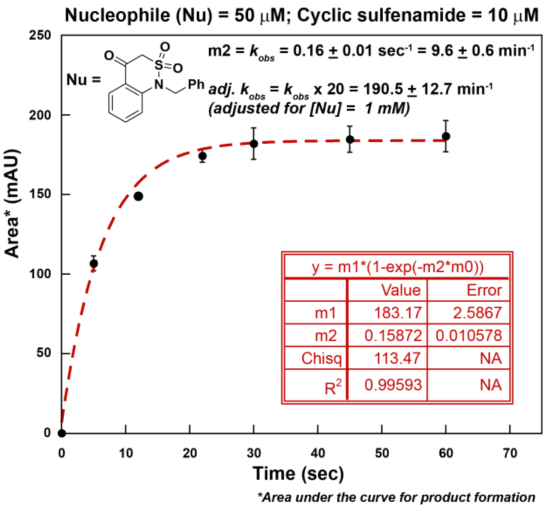
Nucleophile (Nu) = 50 μM; Cyclic sulfenamide = 10 μM
Nu =
Ph
m2 = k_obs = 0.16 ± 0.01 sec-1 = 9.6 ± 0.6 min-1
adj. k_obs = k_obs x 20 = 190.5 ± 12.7 min-1
(adjusted for [Nu] = 1 mM)
y = m1*(1-exp(-m2*m0))
Value Error
m1 183.17 2.5867
m2 0.15872 0.010578
Chisq 113.47 NA
R2 0.99593 NA
Area* (mAU)
Time (sec)
*Area under the curve for product formation

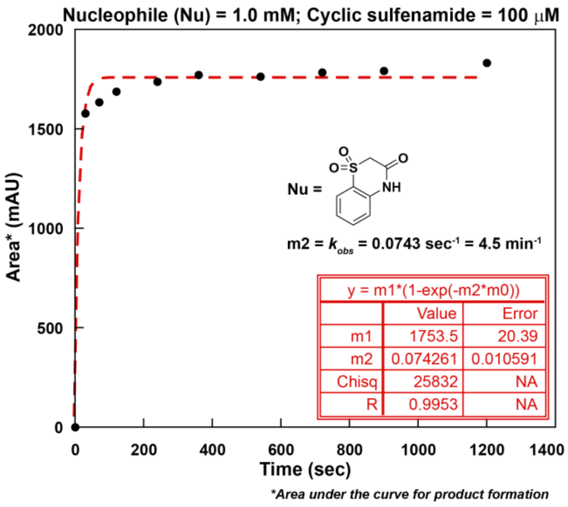
Nucleophile (Nu) = 1.0 mM; Cyclic sulfenamide = 100 μM
Nu =
NH
m2 = k_obs = 0.0743 sec-1 = 4.5 min-1
y = m1*(1-exp(-m2*m0))
Value Error
m1 1753.5 20.39
m2 0.074261 0.010591
Chisq 25832 NA
R 0.9953 NA
Area* (mAU)
Time (sec)
*Area under the curve for product formation

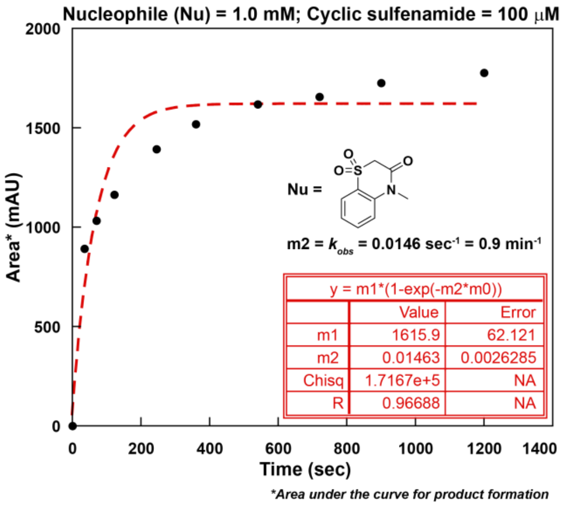
Nucleophile (Nu) = 1.0 mM; Cyclic sulfenamide = 100 μM
Nu =
m2 = k_obs = 0.0146 sec-1 = 0.9 min-1
y = m1*(1-exp(-m2*m0))
Value Error
m1 1615.9 62.121
m2 0.01463 0.0026285
Chisq 1.7167e+5 NA
R 0.96688 NA
Area* (mAU)
Time (sec)
*Area under the curve for product formation

Chart 6 – Rate Plots

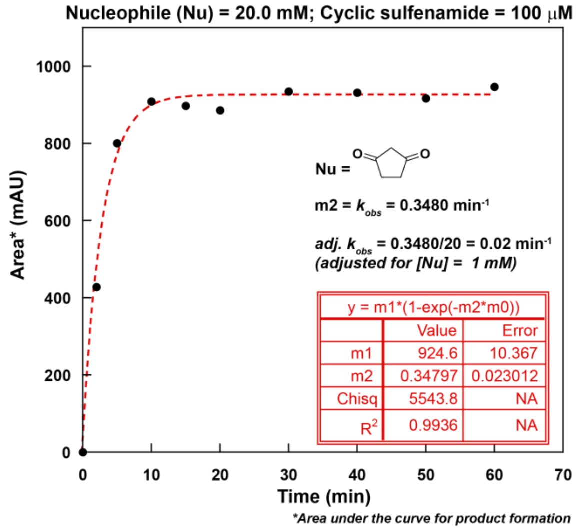

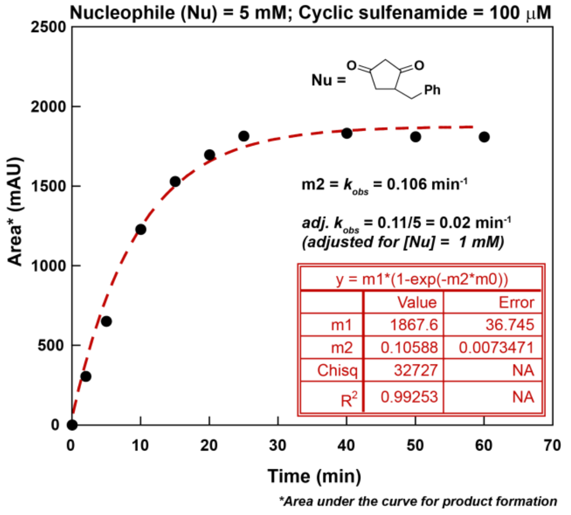

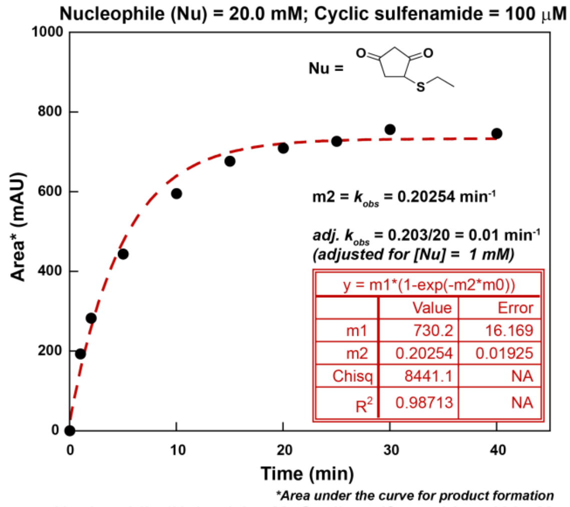

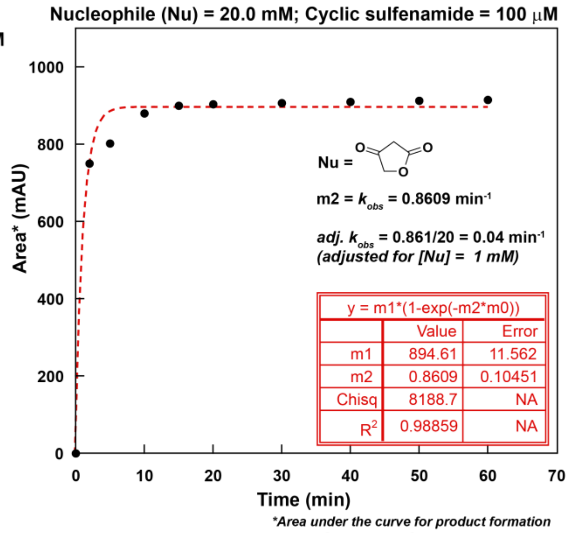

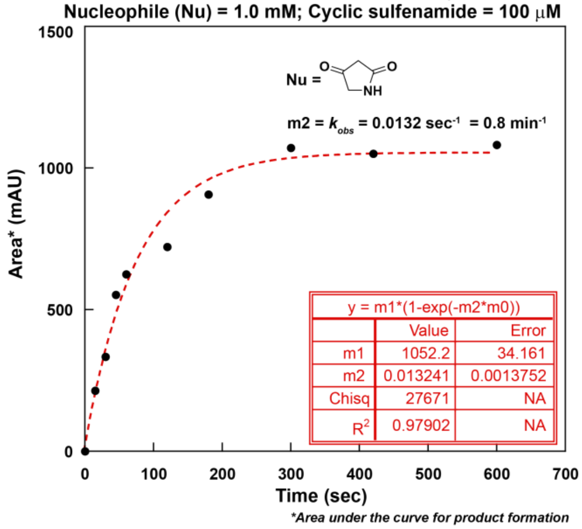

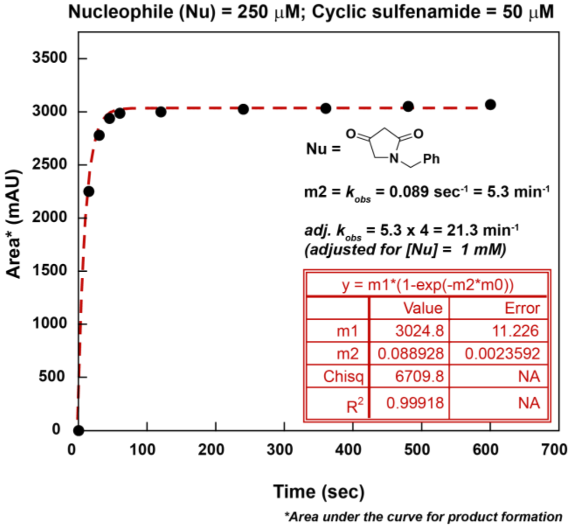

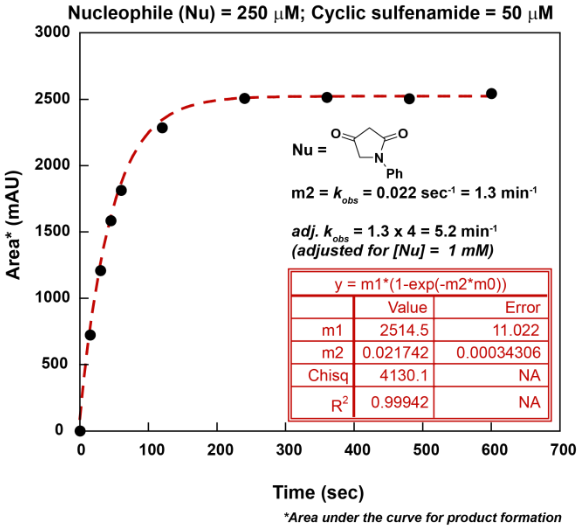
Nucleophile (Nu) = 250 µM; Cyclic sulfenamide = 50 µM
Nu =
Ph
m2 = k_obs = 0.022 sec^-1 = 1.3 min^-1
adj. k_obs = 1.3 x 4 = 5.2 min^-1
(adjusted for [Nu] = 1 mM)
y = m1*(1-exp(-m2*m0))
Value Error
m1 2514.5 11.022
m2 0.021742 0.00034306
Chisq 4130.1 NA
R^2 0.99942 NA
Area* (mAU)
Time (sec)
*Area under the curve for product formation

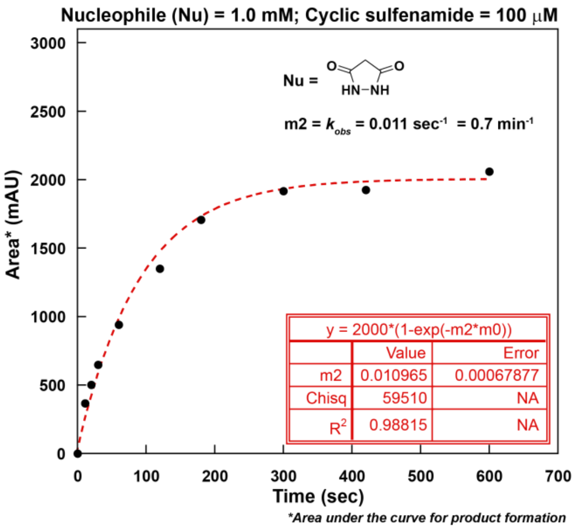
Nucleophile (Nu) = 1.0 mM; Cyclic sulfenamide = 100 µM
Nu =
HN–NH
m2 = k_obs = 0.011 sec^-1 = 0.7 min^-1
y = 2000*(1-exp(-m2*m0))
Value Error
m2 0.010965 0.00067877
Chisq 59510 NA
R^2 0.98815 NA
Area* (mAU)
Time (sec)
*Area under the curve for product formation

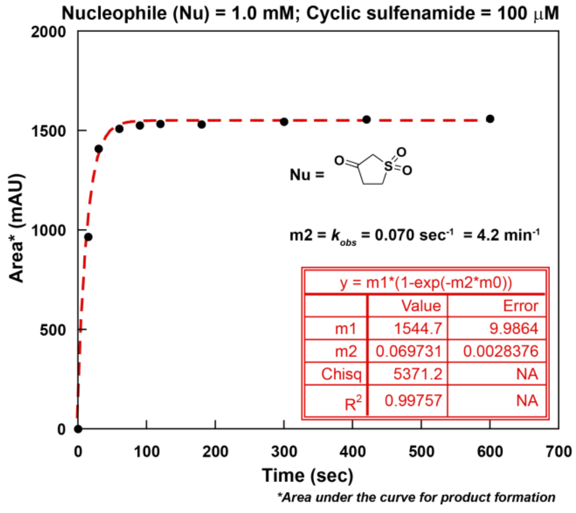
Nucleophile (Nu) = 1.0 mM; Cyclic sulfenamide = 100 µM
Nu =
m2 = k_obs = 0.070 sec^-1 = 4.2 min^-1
y = m1*(1-exp(-m2*m0))
Value Error
m1 1544.7 9.9864
m2 0.069731 0.0028376
Chisq 5371.2 NA
R^2 0.99757 NA
Area* (mAU)
Time (sec)
*Area under the curve for product formation

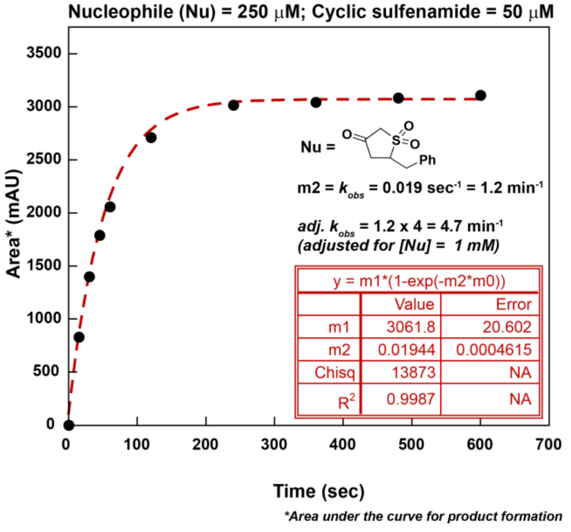
Nucleophile (Nu) = 250 µM; Cyclic sulfenamide = 50 µM
Nu =
Ph
m2 = k_obs = 0.019 sec^-1 = 1.2 min^-1
adj. k_obs = 1.2 x 4 = 4.7 min^-1
(adjusted for [Nu] = 1 mM)
y = m1*(1-exp(-m2*m0))
Value Error
m1 3061.8 20.602
m2 0.01944 0.0004615
Chisq 13873 NA
R^2 0.9987 NA
Area* (mAU)
Time (sec)
*Area under the curve for product formation

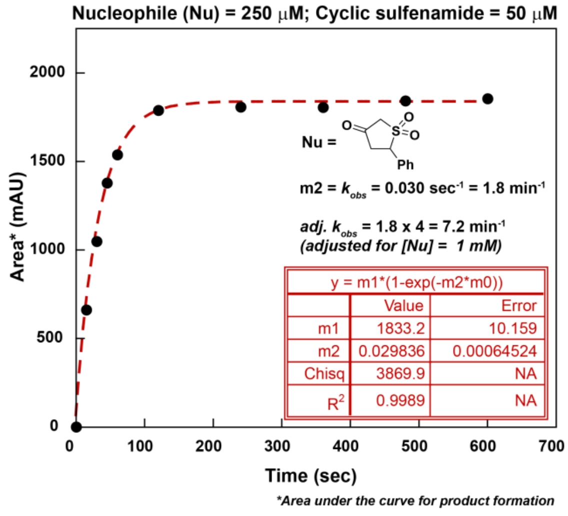
Nucleophile (Nu) = 250 µM; Cyclic sulfenamide = 50 µM
Nu =
Ph
m2 = k_obs = 0.030 sec^-1 = 1.8 min^-1
adj. k_obs = 1.8 x 4 = 7.2 min^-1
(adjusted for [Nu] = 1 mM)
y = m1*(1-exp(-m2*m0))
Value Error
m1 1833.2 10.159
m2 0.029836 0.00064524
Chisq 3869.9 NA
R^2 0.9989 NA
Area* (mAU)
Time (sec)
*Area under the curve for product formation

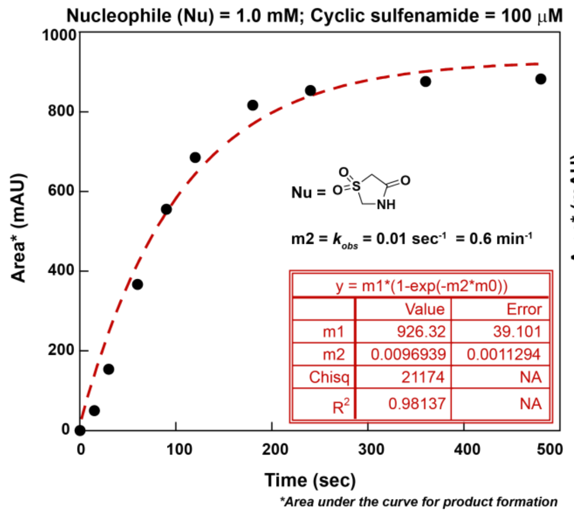
Nucleophile (Nu) = 1.0 mM; Cyclic sulfenamide = 100 μM
Area* (mAU)
Time (sec)
Nu =
m2 = k_obs = 0.01 sec-1 = 0.6 min-1
y = m1*(1-exp(-m2*m0))
Value Error
m1 926.32 39.101
m2 0.0096939 0.0011294
Chisq 21174 NA
R2 0.98137 NA
*Area under the curve for product formation

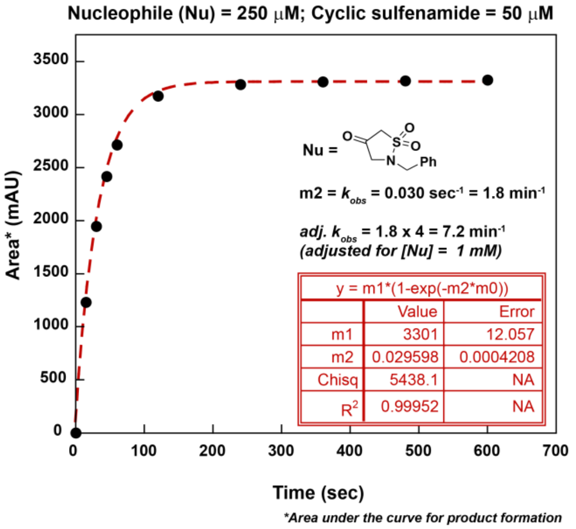
Nucleophile (Nu) = 250 μM; Cyclic sulfenamide = 50 μM
Area* (mAU)
Time (sec)
Nu =
m2 = k_obs = 0.030 sec-1 = 1.8 min-1
adj. k_obs = 1.8 x 4 = 7.2 min-1
(adjusted for [Nu] = 1 mM)
y = m1*(1-exp(-m2*m0))
Value Error
m1 3301 12.057
m2 0.029598 0.0004208
Chisq 5438.1 NA
R2 0.99952 NA
*Area under the curve for product formation

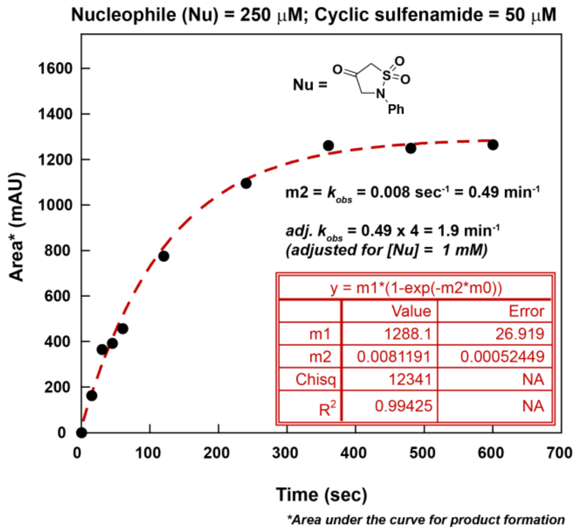
Nucleophile (Nu) = 250 μM; Cyclic sulfenamide = 50 μM
Area* (mAU)
Time (sec)
Nu =
m2 = k_obs = 0.008 sec-1 = 0.49 min-1
adj. k_obs = 0.49 x 4 = 1.9 min-1
(adjusted for [Nu] = 1 mM)
y = m1*(1-exp(-m2*m0))
Value Error
m1 1288.1 26.919
m2 0.0081191 0.00052449
Chisq 12341 NA
R2 0.99425 NA
*Area under the curve for product formation

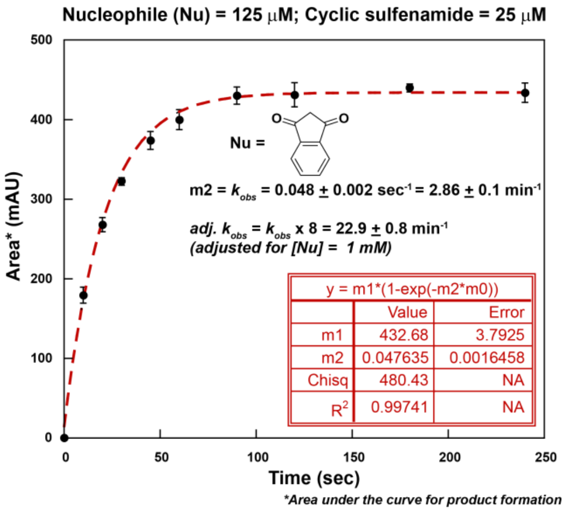
Nucleophile (Nu) = 125 μM; Cyclic sulfenamide = 25 μM
Area* (mAU)
Time (sec)
Nu =
m2 = k_obs = 0.048 ± 0.002 sec-1 = 2.86 ± 0.1 min-1
adj. k_obs = k_obs x 8 = 22.9 ± 0.8 min-1
(adjusted for [Nu] = 1 mM)
y = m1*(1-exp(-m2*m0))
Value Error
m1 432.68 3.7925
m2 0.047635 0.0016458
Chisq 480.43 NA
R2 0.99741 NA
*Area under the curve for product formation

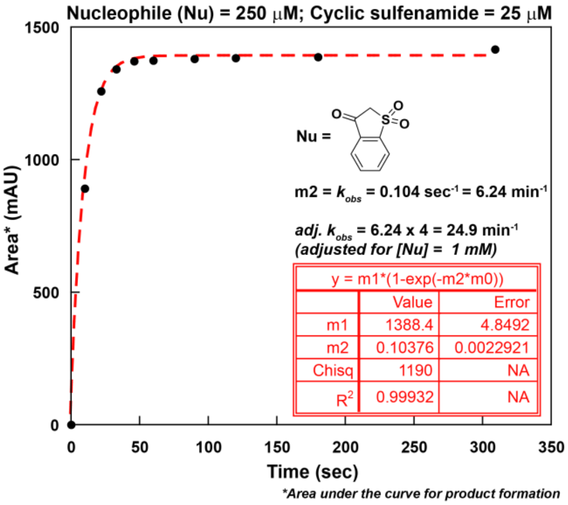
Nucleophile (Nu) = 250 μM; Cyclic sulfenamide = 25 μM
Area* (mAU)
Time (sec)
Nu =
m2 = k_obs = 0.104 sec-1 = 6.24 min-1
adj. k_obs = 6.24 x 4 = 24.9 min-1
(adjusted for [Nu] = 1 mM)
y = m1*(1-exp(-m2*m0))
Value Error
m1 1388.4 4.8492
m2 0.10376 0.0022921
Chisq 1190 NA
R2 0.99932 NA
*Area under the curve for product formation

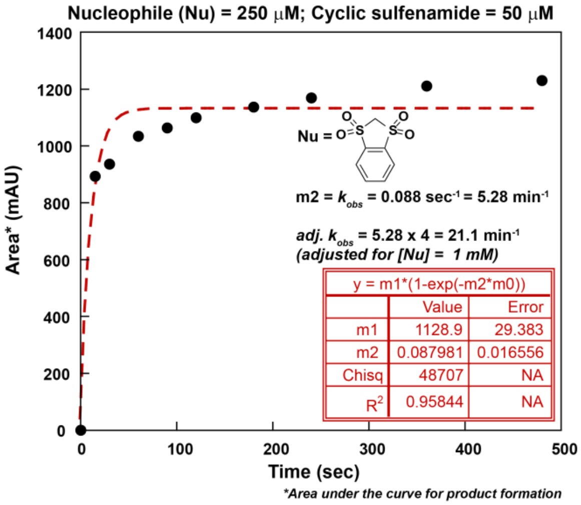
Nucleophile (Nu) = 250 μM; Cyclic sulfenamide = 50 μM
Area* (mAU)
Time (sec)
Nu =
m2 = k_obs = 0.088 sec-1 = 5.28 min-1
adj. k_obs = 5.28 x 4 = 21.1 min-1
(adjusted for [Nu] = 1 mM)
y = m1*(1-exp(-m2*m0))
Value Error
m1 1128.9 29.383
m2 0.087981 0.016556
Chisq 48707 NA
R2 0.95844 NA
*Area under the curve for product formation

Chart S3. Determination of 2nd order rate constants of selected nucleophiles

(A) 2nd order rate constant of dimedone – fixing the cyclic sulfenamide (100 μM) concentration and varying Dimedone concentration (0.5 mM – 2.5 mM).

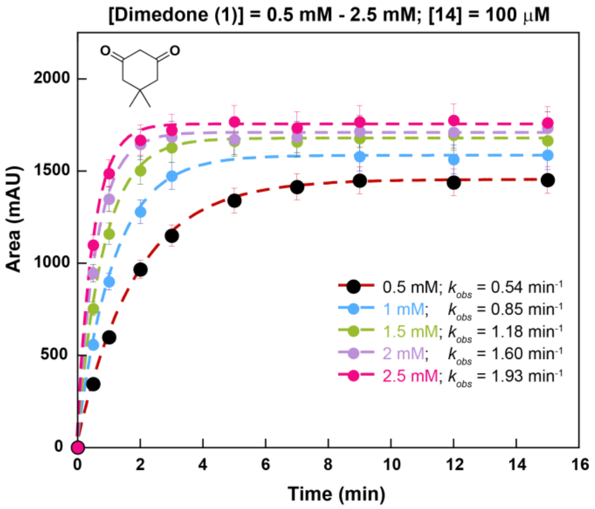

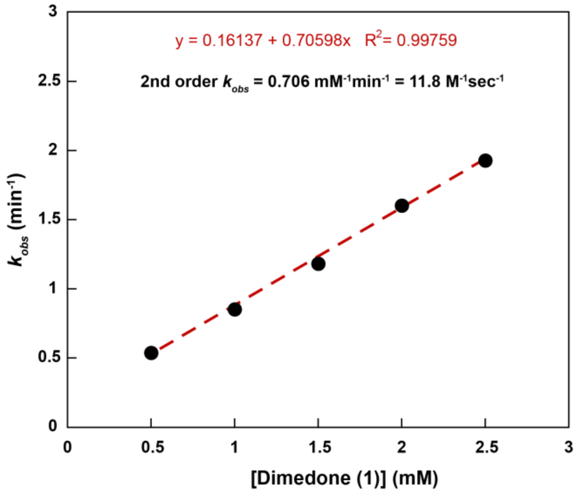

(B) 2nd order rate constant of benzyl-PRD (26f) – fixing the cyclic sulfenamide (14) concentration (20 μM) and varying 26f concentration (100 μM – 200 μM).

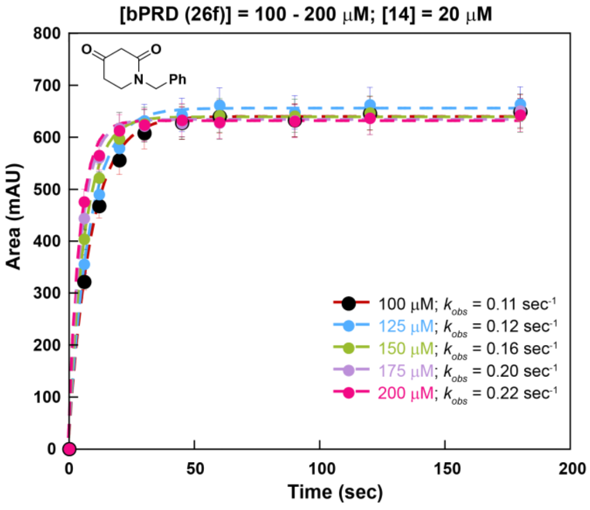

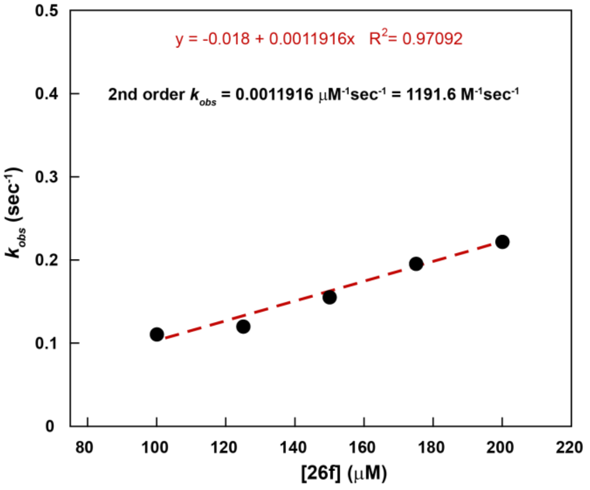

(C) 2nd order rate constant of Indandione (34b) – fixing the cyclic sulfenamide (14) concentration (10 μM) and varying 34b concentration (100 μM – 200 μM).

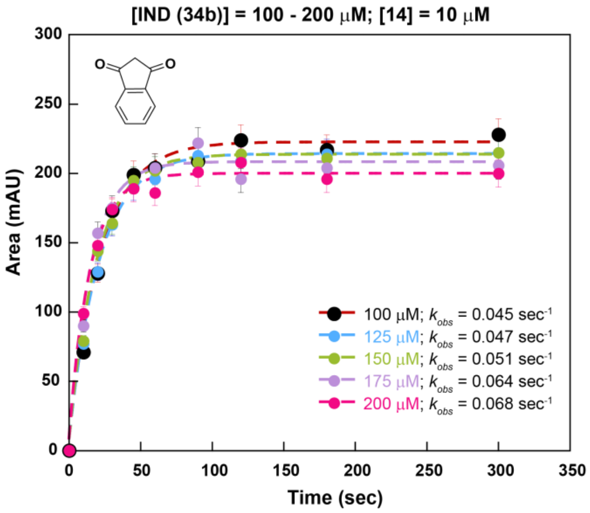

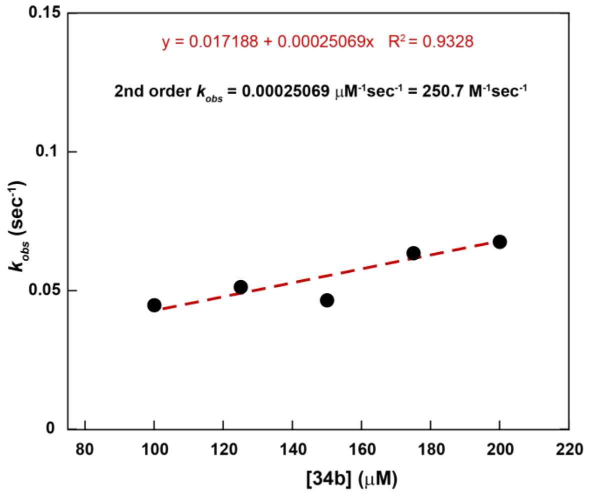

(D) 2nd order rate constant of benzyl-BTD (31h) – fixing the cyclic sulfenamide (14) concentration (10 μM) and 31h concentration (50 μM – 150 μM).

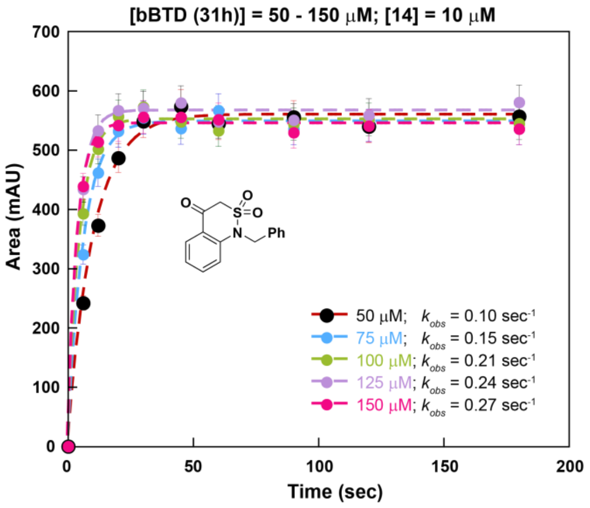

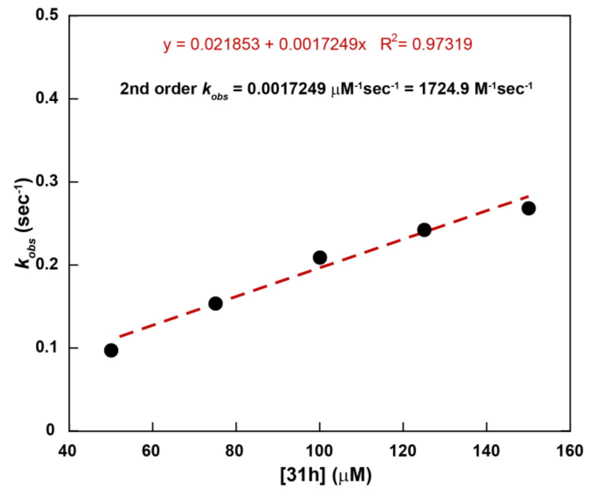

Summary Table:

Nucleophile	1st order k_{obs} (min^{-1})	Experimental 2nd order k_{obs} (M^{-1}sec^{-1})	Comparison of 1st order k_{obs} with Dimedone	Comparison of 2nd order k_{obs} with Dimdeone
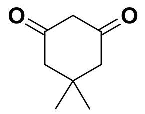	0.8	11.8	1	1
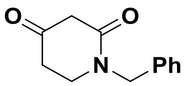	86.4	1191.6	108-fold	101-fold
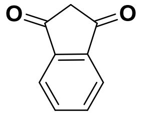	22.9	250.7	29-fold	21-fold
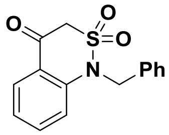	190.5	1724.9	238-fold	146-fold

Scheme S13. Improved synthesis of cyclic sulfenamide [a]

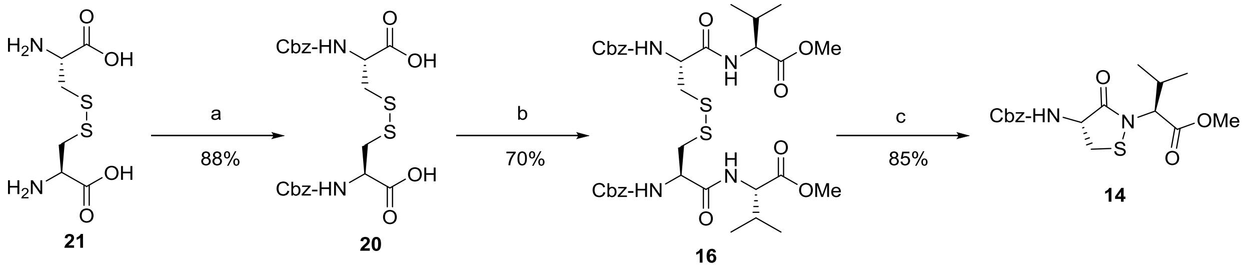

[a] Conditions: (a) Cbz-Cl, $NaHCO_3$(aq), 0 ^oC – rt; (b) H_2N-Val-OMe, EDCI.HCl, cat. DMAP, THF, rt, 15 h; (c) Br_2, pyridine, DCM, -78 ^oC – rt.

Synthesis of (*S*)-methyl 2-amino-3-methylbutanoate

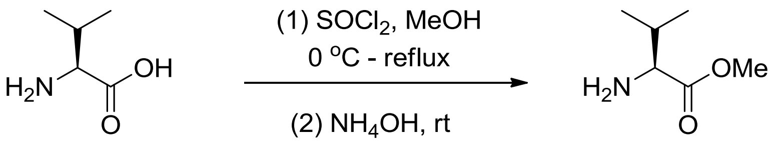

Anydrous methanol (35 ml) was taken in a 100 ml round bottom flask, equipped with a stir-bar and nitrogen balloon. The flask was cooled to 0 ^oC using an ice-bath and thionyl chloride (9.3 ml, 128 mmol) was added dropwise keeping the temperature under 5 ^oC. This solution was stirred at rt for 15 minutes followed by the addition of L-Valine (7.5 g, 64 mmol) in one batch. Resulting white slurry was refluxed till it turned into clear solution and another 2 h afterwards. After the completion of reaction, the reaction mixture was allowed to cool to room temperature and evaporated under reduced pressure. Residue was taken up in methanol and evaporated again. This was repeated twice. Cream residue thus obtained was taken-up in ammonium hydroxide and stirred for 30 minutes followed by extractions with diethyl ether (3 x 100 ml). Ether layers were combined, washed with water and brine, dried over anhydrous magnesium sulfate, filtered and evaporated to obtain light-yellow oil.

^{1}H-NMR: δ 3.62 (s, 3H), 3.19 (d, 1H, J = 8 Hz), 1.90 (h, 1H, J = 8.0 Hz), 1.31 (bs, 2H), 0.85 (d, 3H, J = 8.0 Hz), 0.80 (d, 3H, J = 8.0 Hz); ^{13}C-NMR: 176.2, 60.1, 51.8, 32.3, 19.4, 17.3.

Synthesis of (Cbz-Cys-Val-OMe)$_2$

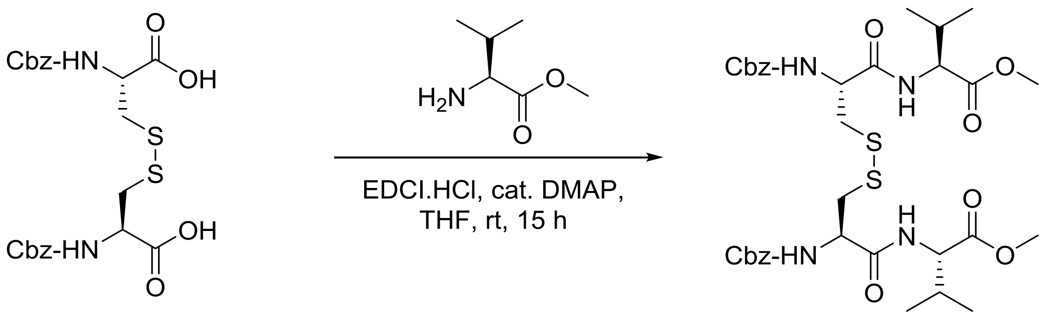

(Cbz-Cys)$_2$ (5.08 g, 10.0 mmol) and L-Val-OMe (3.29 g, 25.0 mmol) were taken together in anhydrous THF (100 ml) under nitrogen pressure. To this slurry was added EDCI.HCl (4.79 g, 25.0 mmol) and catalytic amount of 4-(dimethylamino)pyridine (DMAP, 0.3 g, 2.5 mmol) at 0 ^oC. The resulting reaction mixture was allowed to stir for 15 h at rt. After the completion (as indicated by TLC and LC-MS) the reaction was quenched by the addition of water and THF was removed *in vacuo*. The residue was taken in methylene chloride (200 ml) and washed twice with HCl (1 N, 100 ml), water (100 ml) and aqueous sodium bicarbonate solution (saturated, 100 ml). Organic layer was again washed with water (100 ml) and brine (100 ml) followed by drying over anhydrous magnesium sulfate, filtration and evaporation to give the crude product. Purification by silica gel column chromatography using a gradient of 100% DCM to 10% EtOAc:DCM resulted in the recovery of pure product as white powder in 77% isolated yield. Alternatively, the crude can also be purified by recrystallization from absolute ethanol. First crop gave a yield of 66% and second crop was recovered in 15% yield.

^{1}H-NMR (400 MHz, $CDCl_3$) δ 7.91 (d, 2H, J = 10.0 Hz), 7.30 – 7.38 (m, 5H), 5.91 (d, 2H, J = 8.0 Hz), 5.23 (d, 2H, J = 12.0 Hz), 5.13 (d, 2H, J = 12.0 Hz), 4.50 (dd, 2H, J_A = 4.0 Hz, J_B = 8.0 Hz), 3.70 (s, 6H), 3.11 (dd, 2H, J_A = 16.0 Hz, J_B = 4.0 Hz), 2.88 (dd, 2H, J_A = 12.0 Hz, J_B = 16.0 Hz), 2.16 (h, 2H, J = 4.0 Hz), 0.96 (t, 12H, J = 8.0 Hz); ^{13}C-NMR

(100 MHz, $CDCl_3$) δ 171.2, 169.9, 156.5, 136.3, 128.9, 128.6, 128.4, 67.5, 62.4, 54.6, 52.6, 37.8, 29.5, 19.7, 19.4.

Synthesis of (S)-methyl 2-((R)-4-(((benzyloxy)carbonyl)amino)-3-oxoisothiazolidin-2-yl)-3-methylbutanoate (Cyclic sulfenamide)

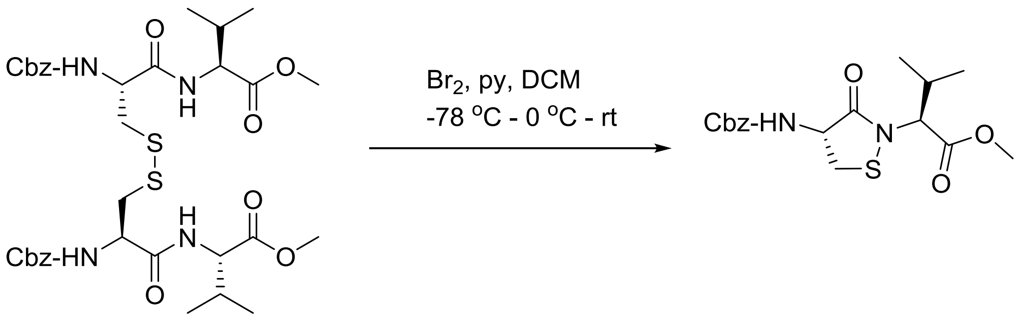

To a solution of $(Cbz\text{-}Cys\text{-}Val\text{-}OMe)_2$ (1.47 g, 2.0 mmol) in 100 mL anhydrous methylene chloride was added pyridine (1.3 ml, 16 mmol). The solution was cooled to -78 ^oC under N_2 and stirred for 15 min, and bromine (0.64 g, 4.0 mmol) in anhydrous methylene chloride (15.0 ml) was added dropwise over 10 minutes. The resulting pale yellow solution was stirred at -78 ^oC for 30 min and then at -10 ^oC (ice-acetone bath) for 3 h and finally allowed to warm to room temperature by removing the cooling bath. After the completion, reaction mixture was directly concentrated to give a yellow solid. The crude solid was then immediately purified by silica gel column chromatography using a gradient of 100% DCM to 5% EtOAc in DCM to obtain the pure product as white solid in 82% isolated yield.

^{1}H-NMR (400 MHz, $CDCl_3$) δ 7.31 – 7.39 (m, 5H), 5.63 (bs, 1H), 5.14 (s, 2H), 4.60 – 4.69 (m, 2H), 3.92 (t, 1H, J = 8.0 Hz), 3.73 (s, 3H), 3.41 (dd, 1H, J_A = 12.0 Hz, J_B = 12.0 Hz), 2.24 (h, 1H, J = 4.0 Hz), 0.98 (t, 6H, J = 8.0 Hz); ^{13}C-NMR (100 MHz, $CDCl_3$) δ 172.2, 170.6, 157.0, 136.4, 128.9, 128.5, 128.0, 67.6, 58.1, 55.0, 52.4, 47.2, 31.3, 19.4, 18.5.

Scheme S14. Synthesis of nucleophiles in Chart 2.

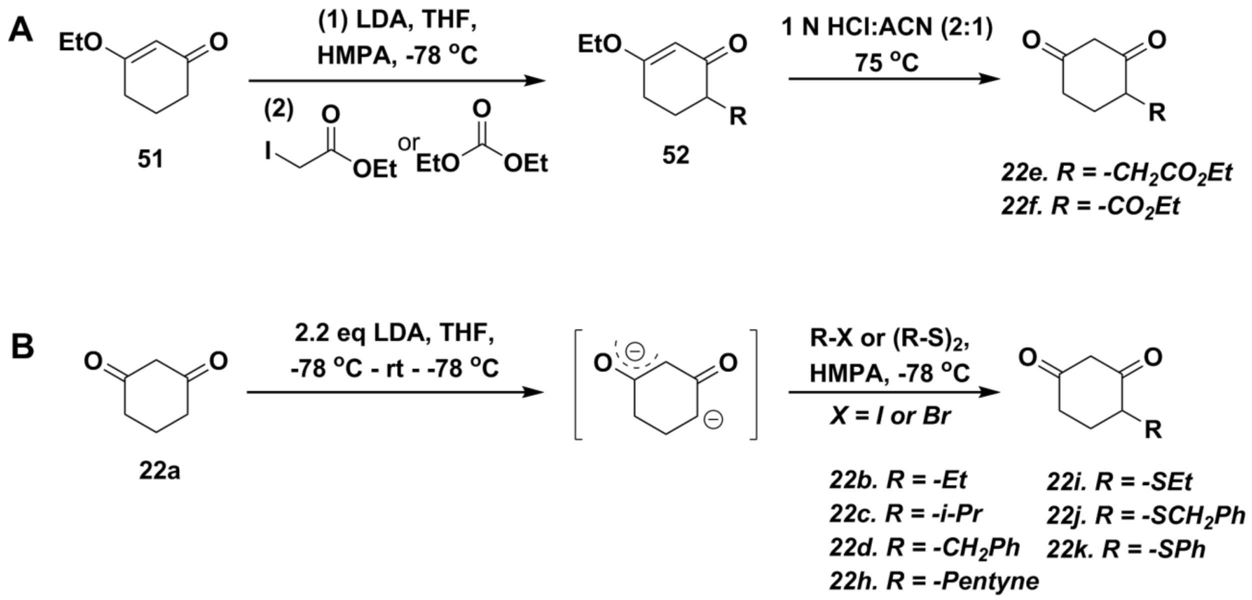

First strategy used to synthesize analogs **22e-f** was based on a 2-step strategy. First step was to alkylate 3-ethoxy-2-cyclohexen-1-one (**51**) with ethyl iodoacetate or diethyl carbonate under kinetically controlled conditions (LDA, HMPA, THF) followed by acid mediated deprotection conditions resulting in the recovery of targeted C-4 alkylated product **22e-f** in good yields. The second, more straight-forward strategy used the dianion chemistry where the dianion of 1,3-cyclohexanedione (**22a**) was smoothly generated with 2.2 equivalents of lithium diisopropylamide. Reaction of this with various alkyl iodides and bromides afforded the expected C-4 alkylated derivatives (**22b-d**, **h**). The reaction of dianion of **22a** with different disulfides resulted in clean formation of C-4 alkylthio derivatives **22i-k.**

Procedure for Scheme A: Step 1 – Base mediated C-4 alkylation. In a flame dried 100 ml flask equipped with stir bar and diisopropylamine (1.73 g, 17.1 mmol), 20 ml of freshly distilled dry THF was added followed by the addition of 2.5 M solution of *n*-BuLi (6.85 ml, 17.1 mmol) at -78 ^{o}C under N_2. This mixture was stirred at -78 ^{o}C for 45 minutes. A solution of 3-ethoxycyclohex-2-enone (2.0 g, 14.3 mmol) in freshly distilled dry THF (15 ml) was added drop wise to the LDA solution at -78 ^{o}C. Resulting dark orange colored reaction mixture was allowed to stir at -78 ^{o}C for another 60 minutes. A

solution of ethyl-2-iodoacetate (3.66 g, 17.1 mmol) and HMPA (3.1 g, 17.1 mmol) in freshly distilled dry THF (10 ml) was added directly at -78 ^{o}C to the enolate and the resulting orange solution was allowed to warm up to rt over 2 h with monitoring (TLC and LC-MS). The reaction was complete after 4 h of stirring and it was quenched with saturated ammonium chloride solution and THF was removed *in vacuo*. Residue was partitioned between ethyl acetate and water and organic layer was separated, washed with brine and dried over anhydrous magnesium sulfate. Solvent was evaporated to give crude product. The product was isolated by column chromatography using a gradient of Hexane:EtOAc (15% to 25%). The expected product was isolated in 72% yield, analyzed and confirmed by LC-MS and NMRs.

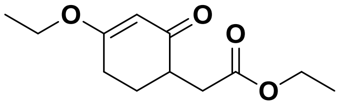

Ethyl 2-(4-ethoxy-2-oxocyclohex-3-en-1-yl)acetate (52a)

1H-NMR (in CDCl3, 400 MHz): δ 5.30 (s, 1H), 4.10 (q, 2H, J = 8.0 Hz), 3.84 (p, 2H, J = 8.0 Hz), 2.84 – 2.89 (m, 1H), 2.64 – 2.69 (m, 1H), 2.45 – 2.55 (m, 1H), 2.30 – 2.33 (m, 1H), 2.19 – 2.25 (m, 1H), 2.00 – 2.05 (m, 1H), 1.74 (dq, 1H, J1 = 12 Hz, J2 = 4 Hz), 1.31 (t, 3H, J = 8.0 Hz), 1.22 (t, 3H, J = 8.0 Hz); 13C-NMR (in CDCl3, 100 MHz): 199.1, 177.2, 172.9, 102.2, 64.5, 60.1, 42.5, 34.9, 29.2, 27.3, 14.4, 14.3.

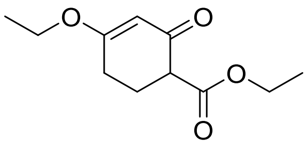

Ethyl 4-ethoxy-2-oxocyclohex-3-ene-1-carboxylate (52b)

1H-NMR (in CDCl3, 400 MHz): δ 5.37 (s, 1H), 4.20 (q, 2H, J = 8.0 Hz), 3.90 (dq, 2H, J1 = 8.0 Hz, J2 = 4.0 Hz), 3.30 (dd, 1H, J1 = 8.0 Hz, J2 = 4.0 Hz), 2.52 – 2.59 (m, 1H), 2.28 – 2.45 (m, 2H), 2.12 – 2.19 (m, 1H), 1.36 (t, 3H, J = 8.0 Hz), 1.27 (t, 3H, J = 8.0 Hz); 13C-NMR (in CDCl3, 100 MHz): 194.1, 177.9, 170.7, 102.4, 64.8, 61.5, 52.6, 27.6, 24.4, 14.5, 14.4.

Step 2 – Acid catalyzed deprotection. SM ester (0.5 g, 2.2 mmol) was taken in acetronitrile (5 ml). To this, 1.0 N HCl (10 ml) was added at rt. Resulting reaction mixture was stirred at 70 ^{o}C with monitoring. The reaction was completed after 1 h. Acetonitrile was removed *in vacuo* and residue was extracted with ethyl acetate (3x30 ml). Combined organic layers were washed with brine, dried over anhydrous magnesium sulfate, filtered and evaporated to give the crude product which was purified by column chromatography using 100% DCM – 5% MeOH:DCM gradient to obtain the pure product in 80% yield.

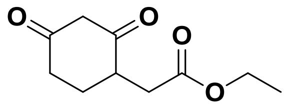

Ethyl 2-(2,4-dioxocyclohexyl)acetate (22e) (Mixture of keto and enol form)

^{1}H-NMR (in CDCl3, 400 MHz): δ 5.44 (s, 0.4H), 4.15 (q, 2H, J = 8.0 Hz), 3.46 (q, 1H, J = 16.0 Hz), 2.03 – 3.04 (m, 6H), 1.59 – 1.85 (m, 1H), 1.25 (dt, 3H, J1 = 8.0 Hz, J2 = 4.0 Hz); ^{13}C-NMR: 203.8, 203.3, 195.8, 185.9, 173.2, 172.2, 104.5, 61.2, 61.1, 58.4, 46.1, 40.3, 40.1, 35.5, 34.2, 30.4, 27.3, 24.7, 14.4.

Procedure for Scheme B: Lithium diisopropylaminde was prepared by the dropwise addition of 2.5 M solution of *n*-BuLi (15.7 ml, 39.25 mmol) to a solution of diisopropylamine (3.97 g, 39.25 mmol) in THF (40 ml) and stirring the resultant pale yellow mixture at -78 ^{o}C for 30 minutes in 250 ml flask equipped with stir bar under N_2 pressure. A solution of cyclohexane-1,3-dione (2.0 g, 17.84 mmol) in THF (20 ml) and HMPA (10 ml) was added dropwise to this lithium diisopropylamide solution at -78 ^{o}C. Resulting deep yellow colored reaction mixture was allowed to stir at -78 ^{o}C and the temperature was slowly increased to 0 ^{o}C. The reaction mixture was allowed to stir at 0 ^{o}C for 30 minutes after which it was again cooled to -78 ^{o}C. To this dianion slurry, a solution of 5-iodopent-1-yne (3.81 g, 19.62 mmol) in THF (20 ml) was added dropwise at -78 ^{o}C. Resulting orange slurry was stirred at -78 ^{o}C for 2 h with close monitoring. After the completion, the reaction mixture was acidified (to pH 4) with 1.0 M HCl. THF was removed under reduced pressure and the residue was partitioned between ethyl acetate and water. Organic layer was separated and aqueous layer was extracted with

ethyl acetate (3 x 75 ml). Organic layers were combined and washed with brine, dried over anhydrous magnesium sulfate, filtered and evaporated to give crude product. The crude was purified by column chromatography using a gradient of 100% DCM which was increased upto 2% MeOH in DCM to recover the product (3.0 g, 16.83 mmol) as a mixture of keto and enol form in 94% isolated yield. The product was further purified by reversed-phase preparative HPLC (Agilent 1260 Infinity instrument using Varian Pursuit XRs 5 C18-A 150 x 21.2 mm column) using a gradient of water/acetonitrile from 95:5 to 5:95 over 30 min.

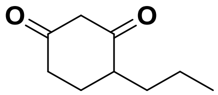

4-propylcyclohexane-1,3-dione (22b)

^{1}H-NMR (in DMSO-d6, 400 MHz): δ 10.89 (bs, 1H), 5.17 (s, 1H), 2.25 – 2.40 (m, 2H), 2.05 – 2.2 (m, 1H), 1.91 – 1.98 (m, 1H), 1.55 – 1.75 (m, 2H), 1.22 – 1.42 (m, 3H), 0.88 (t, 3H, J = 8.0 Hz); ^{13}C-NMR (in DMSO-d6, 100 MHz): 103.3, 31.6, 25.6, 19.7, 14.0.

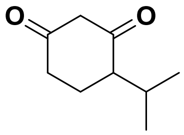

4-isopropylcyclohexane-1,3-dione (22c)

1H-NMR (in DMSO-d6, 400 MHz): δ 5.20 (s, 1H), 1.95 – 2.05 (m, 1H), 1.80 – 1.90 (m, 1H), 1.61 – 1.71 (m, 1H), 0.90 (d, 3H, J = 8.0 Hz), 0.77 (d, 3H, J = 8.0 Hz); 13C-NMR (in DMSO-d6, 100 MHz): 104.0, 26.0, 21.1, 20.4, 18.2.

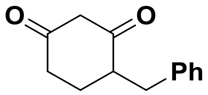

4-benzylcyclohexane-1,3-dione (22d)

^{1}H-NMR (in DMSO-d6): δ 11.08 (s, 1H), 7.25 – 7.32 (m, 2H), 7.15 – 7.23 (m, 3H), 5.26 (s, 1H), 3.14 (dd, 1H, J1 = 8.0 Hz, J2 = 4.0 Hz), 2.25 – 2.40 (m, 4H), 1.70 – 1.75 (m,

1H), 1.48 (sept, 1H, J = 8.0 Hz); ^{13}C-NMR (in DMSO-d6, 100 MHz): 140.1, 129.0, 128.2, 125.9, 103.4, 35.3, 24.9

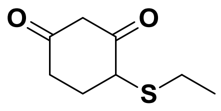

4-(ethylthio)cyclohexane-1,3-dione (22i)

^{1}H-NMR (in DMSO-d6, 400 MHz): δ 11.18 (s, 1H), 5.15 (s, 1H), 3.38 (bs, 1H), 2.40 – 2.65 (m, 3H), 2.15 - 2.30 (m, 2H), 1.87 – 1.97 (m, 1H), 1.18 (t, 3H, J = 8.0 Hz); ^{13}C-NMR (in DMSO-d6, 100 MHz): 102.2, 26.8, 24.5, 14.5

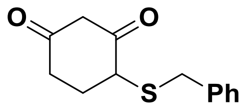

4-(benzylthio)cyclohexane-1,3-dione (22j)

^{1}H-NMR (in DMSO-d6, 400 MHz): δ 11.25 (s, 1H), 7.29 – 7.37 (m, 4H), 7.22 – 7.27 (m, 1H), 5.18 (s, 1H), 3.79 – 3.87 (m, 2H), 3.15 – 3.42 (m, 1H), 2.37 – 2.48 (m, 1H), 2.15 – 2.28 (m, 2H), 1.83 – 1.92 (m, 1H); ^{13}C-NMR (in DMSO-d6, 100 MHz): 138.2, 128.9, 128.4, 126.9, 26.5

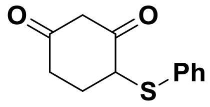

4-(phenylthio)cyclohexane-1,3-dione (22k)

^{1}H-NMR (in DMSO-d6, 400 MHz): δ 11.40 (s, 1H), 7.43 – 7.53 (m, 2H), 7.31 – 7.38 (m, 2H), 7.24 – 7.30 (m, 1H), 5.25 (s, 1H), 4.05 (dt, 1H, J1 = 6.0 Hz, J2 = 4.0 Hz), 2.43 – 2.50 (m, 1H), 2.27 – 2.42 (m, 1H), 2.13 – 2.25 (m, 1H), 1.85 – 1.96 (m, 1H); ^{13}C-NMR (in DMSO-d6, 100 MHz): 134.2, 131.0, 129.0, 126.9, 27.0.

Scheme S15. Synthesis of the nucleophiles in chart 3

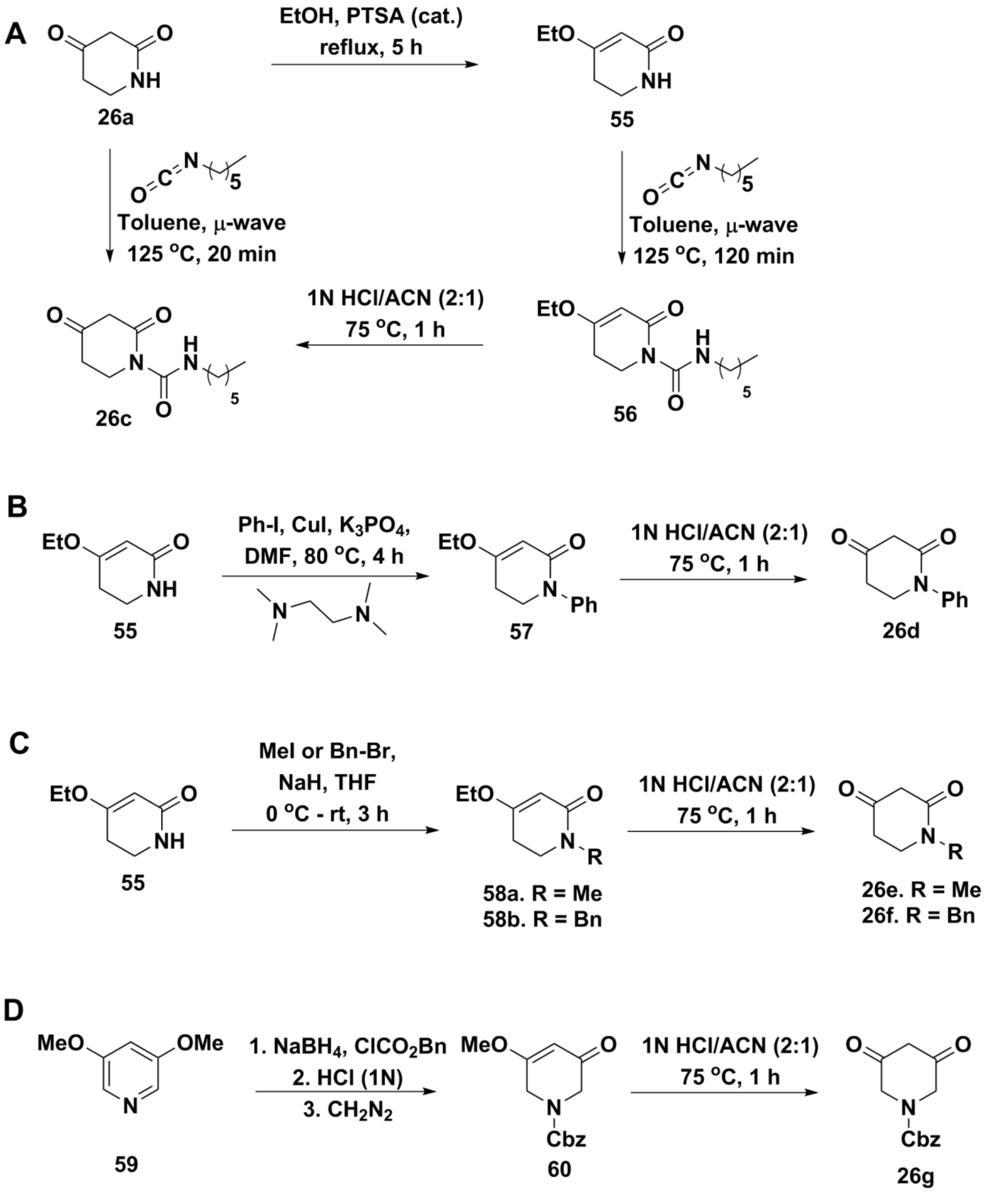

Both **26a** and its protected derivative 4-ethoxy-5-6-dihydropyridin-2(1*H*)-one **(55)** readily undergoes reaction with isocyanate (1-isocyanatopentane) to form corresponding alkylamide derivative **26c** and **56** respectively. Alkylamide derivative **56** was readily deprotected under acidic conditions to form **26c (Reaction A)**. We also developed new methods for the arylation and alkylation of **26a.** An Ullmann-type aromatic amination reaction between phenyl iodide and **55** using catalytic copper (I) iodide resulted in successful synthesis of *N*-arylated derivative **(57)**. Deprotection under acidic conditions gave *N*-arylated nucleophile **26d** in excellent yield **(Reaction B)**. Alkylation of **26a** was achieved by sodium hydride mediated deprotonation of protected lactam **55** and subsequent reaction with alkyl halide to form **58**. Deprotection using above described acidic conditions resulting in *N*-alkylated nucleophiles **(26e-f) (Reaction C)**. For the synthesis of 26g, 3,5-dimethoxypyridine **(59)** was reduced in the presence of benzyl chloroformate to afford an intermediate bis-enol ether, which was hydrolyzed with aqueous acid. The resulting vinylogous acid was etherified with diazomethane to yield the corresponding vinylogous ester **60**, which was then deprotected under acidic conditions to give **26g (Reaction D)**.

Synthesis of *tert*-butyl 2,4-dioxopiperidine-1-carboxylate (26b)

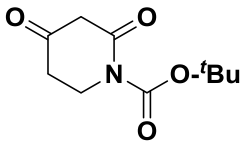

^{1}H-NMR (in CDCl3, 400 MHz): δ 4.09 (t, 2H, J = 8.0 Hz), 3.49 (s, 2H), 2.61 (t, 2H, J = 8.0 Hz), 1.53 (s, 9H); ^{13}C-NMR (in CDCl3, 100 MHz): 202.4, 165.6, 151.5, 84.5, 52.5, 41.0, 38.5, 28.3.

Synthesis of 4-ethoxy-5,6-dihydropyridin-2(1H)-one (55). To a slurry of 2,4-piperidinedione (1.1 g, 10 mmol) in absolute ethanol (75 ml) was added PTSA (0.15 g, 1 mmol) and resulting solution was refluxed for 18 h under nitrogen. After the completion of the reaction, ethanol was removed and residue was partitioned between ethyl acetate and water. Organic layer was separated and aqueous layer was subjected to ethyl acetate extractions (2 x 25 ml). Combined organic layers were washed with brine, dried

over anhydrous $MgSO_4$, filtered and evaporated. This crude was purified by column chromatography using a gradient of 2% - 5% MeOH/DCM to recover the title product in 75% yield.

Synthesis of Urea analog

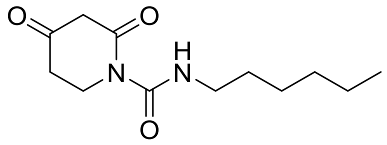

***N*-hexyl-2,4-dioxopiperidine-1-carboxamide (26c).**

^{1}H-NMR (in CDCl3, 400 MHz): δ 8.91 (s, 1H), 4.28 (t, 2H, J = 8.0 Hz), 3.53 (s, 2H), 3.31 (q, 2H, J = 8.0 Hz), 2.61 (t, 2H, J = 8.0 Hz), 1.57 (p, 2H, J = 8.0 Hz), 1.25 – 1.60 (m, 6H), 0.9 (t, 3H, J = 8.0 Hz); ^{13}C-NMR (in CDCl3, 100 MHz): 202.3, 169.4, 153.2, 51.6, 40.7, 38.4, 38.1, 31.4, 29.4, 26.6, 22.5, 14.0.

General procedure for the Cu(I) mediated *N*-arylation to prepare 57. A sealed tube was charged with copper (I) iodide (20 mg, 0.1 mmol), protected lactam **55** (212 mg, 1.5 mmol), K_3PO_4 (430 mg, 2.0 mmol), *N,N'*-dimethylethylenediamine (11 µL, 0.1 mmol), iodobenzene (204 mg, 1.0 mmol) and DMF (3 ml). The reaction mixture was degassed with nitrogen and allowed to heat at 175 ^{o}C for 24 h. After the completion, the reaction mixture was cooled to rt and filtered through a pad of silica gel (0.5 x 1.0 cm) using 10 ml of EtOAc and then 10 ml of 20% MeOH/EtOAc. Filtrate was purified by column chromatography using a gradient of 50% EtOAc/DCM to recover the pure product in quantitative yield.

^{1}H-NMR (400 MHz, CDCl3): δ 7.29 (t, 2H, J = 8.0 Hz), 7.21 (d, 2H, J = 8.0 Hz), 7.11 (t, 1H, J = 8.0 Hz), 5.14 (s, 1H), 3.87 (q, 2H, J = 8.0 Hz), 3.76 (t, 2H, J = 8.0 Hz), 2.52 (t, 2H, J = 8.0 Hz), 1.31 (t, 3H, J = 8.0 Hz). ^{13}C-NMR (in CDCl3, 100 MHz):168.6, 166.8, 143.0, 128.9, 125.7, 125.2, 95.3, 64.4, 47.6, 28.6, 14.3.

General procedure for base mediated *N*-alkylation to prepare 58. To a slurry of NaH (0.36 g, 60% w/w, 9.0 mmol) in anhydrous THF (5 ml), was added a solution of **55** (1.06 g, 7.5 mmol) in anhydrous THF (10 ml) at 0 ^oC under nitogen. The resulting reaction

mixture was stirred at rt for 15 minutes and then again cooled to 0 ^{o}C. To this anionic reaction mixture was added dropwise, a solution of benzyl bromide (1.41, 8.3 mmol) in THF (5 ml). Resulting RM was stirred at rt for 6 h. After completion, the reaction mixture was quenched with water. THF was removed under reduced pressure and residue was partitioned between ethyl acetate and water. Organic layer was separated and aqueous layer was extracted with ethyl acetate (2 x 25 ml). Combined organic layers were washed with brine, dried over anhydrous $MgSO_4$, filtered and evaporated. This crude product was purified by column chromatography using a gradient of 2% - 5% MeOH/DCM to recover the title product in 88 % yield as off-white solid.

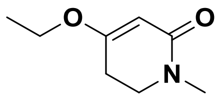

^{1}H-NMR (400 MHz, $CDCl_3$): δ 4.99 (s, 1H), 3.79 (q, 2H, J = 8.0 Hz), 3.30 (t, 2H, J = 8.0 Hz), 2.87 (s, 3H), 2.39 (t, 2H, J = 8.0 Hz), 1.27 (t, 3H, J = 8.0 Hz); ^{13}C-NMR (in CDCl3, 100 MHz):

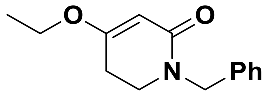

^{1}H-NMR (400 MHz, $CDCl_3$): δ 7.20 – 7.31 (m, 5H), 5.12 (s, 1H), 4.58 (s, 2H), 3.86 (q, 2H, J = 8.0 Hz), 3.26 (t, 2H, J = 8.0 Hz), 2.37 (t, 2H, J = 8.0 Hz), 1.32 (t, 3H, J = 8.0 Hz); ^{13}C-NMR (in CDCl3, 100 MHz): 167.7, 167.2, 137.8, 128.5, 127.9, 127.2, 94.5, 64.1, 49.2, 43.6, 28.0, 14.2.

Scheme S16. Synthesis of the nucleophiles in chart 4.

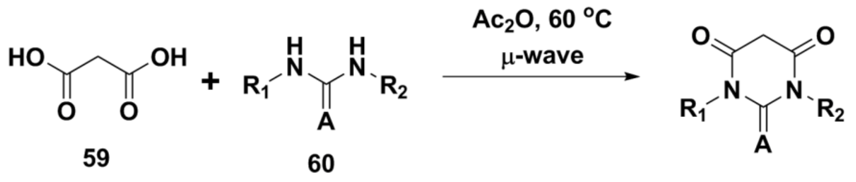

54. R_1 = $-CH_3$; R_2 = H; A = O
55. R_1 = R_2 = $-CH_3$; A = O
57. R_1 = R_2 = $-CH_3$; A = S

General procedure: Mixture of malonic acid (1.04 g, 10 mmol) **(59)** and appropriate urea (10 mmol, 1 eq) **(60)** were dissolved in acetic anhydride (2.04 g, 20 mmol, 2 eq) and the resulting solution was microwave heated at 60 ^{o}C for 30 minutes to form the corresponding barbituric acid derivatives **(Scheme 7)**. After completion, the pressure was released and reaction mixture was evaporated under reduced pressure to remove acetic acid. Residue was recrystallized from hot ethanol to obtain the product.

1-methylpyrimidine-2,4,6(1H,3H,5H)-trione (27b)

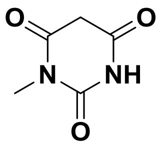

^{1}H-NMR (400 MHz, DMSO-d_6): δ 11.33 (s, 1H), 3.58 (s, 2H), 3.05 (s, 3H); ^{13}C-NMR (in DMSO-d_6, 100 MHz): δ 167.0, 166.5, 151.9, 26.8

1,3-dimethylpyrimidine-2,4,6(1H,3H,5H)-trione (27c)

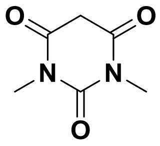

^{1}H-NMR (400 MHz, DMSO-d_6): δ 3.69 (s, 2H), 3.10 (s, 6H); ^{13}C-NMR (in DMSO-d_6, 100 MHz): δ 166.0, 152.4, 27.8

1,3-dimethyl-2-thioxodihydropyrimidine-4,6(1H,5H)-dione (27e)

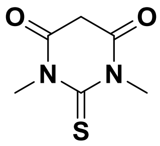

^{1}H-NMR (400 MHz, DMSO-d_6): δ 7.96 (bs, 1H), 3.55 (s, 6H); ^{13}C-NMR (in DMSO-d_6, 100 MHz): δ 35.0.

Scheme S17. Synthesis of the nucleophiles in chart 5

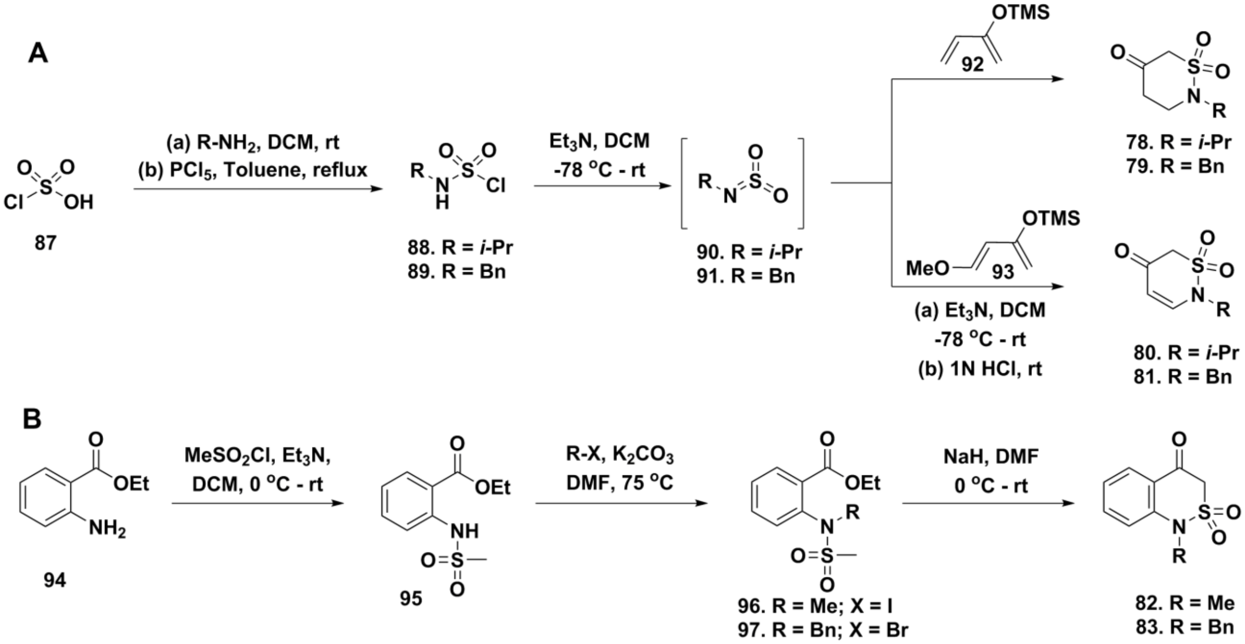

These targets were prepared by [2+4] cycloaddition of *N*-sulfonyl-alkylamines **(90-91)** with 3-trimethoxysiloxy-1,3-butadine **(92)** and 1-methoxy-3-trimethylsiloxy-1,3-butadiene **(93)**. Briefly, chlorosulfonic acid **(87)** and alkyl amine were reacted to get alkylsulfamic acid which was directly converted to alkylsulfamoyl chloride **(88-89)**. Alkylsulfamoyl chloride **(88-89)** then underwent dehydrohalogenation under mild basic conditions to give *N*-sulfonyl-alkylamines **(90-91)**. *N*-sulfonyl-alkylamines **(90-91)** were trapped with 3-trimethoxysiloxy-1,3-butadine **(92)** to give and 1-methoxy-3-trimethylsiloxy-1,3-butadiene respectively **(93)** to give 2-alkyl-1,2-thiazinan-5-one 1,1-dioxide **(78–79)**. Similarly, *N*-sulfonyl-alkylamines **(90-91)** were reacted with 1-methoxy-3-trimethylsiloxy-1,3-butadiene **(93)** followed by acid hydrolysis to give 2*H*-1,2-thiazin-5(6*H*)-one 1,1-dioxides **(80–81) (Scheme 8A)**.

Synthesis of these 1-alkyl-1*H*-benzo[*c*][1,2]thiazin-4(3H)-one 2,2-dioxides is well established with several reported analogs. The synthesis begin with *N*-mesylation of ethyl-2-aminobenzoate **(94)** resulting in ethyl 2-(methylsulfonamido)benzoate **(95)** which was then *N*-alkylated under basic conditions to give ethyl 2-(*N*-

alkylmethylsulfonamido)benzoate **(96-97)** which readily underwent base mediated intramolecular cyclization resulting in the formation of 1-alkyl-1*H*-benzo[*c*][1,2]thiazin-4(3*H*)-one 2,2-dioxides **(82-83)** over three steps.

Preparation of alkylsulfamic acid - A solution of isopropylamine (5.9 g, 100 mmol) in 40 ml chloroform was cooled in an ice-water bath. Chlorosulfonic acid (3.5 g, 30 mmol) was added dropwise over a period of 15 minutes. The mixture was stirred for another hour under nitrogen and then evaporated to yield pink amorphous solid which was taken to next step as is.

Synthesis of *N*-alkylsulfamoyl chloride – Slurry of isopropylsulfamic acid and phosphorus pentachloride (6.24 g, 30 mmol) in toluene (25 ml) was refluxed for 6 h under nitrogen. After cooling, the reaction mixture was filtered through celite and filtrate was evaporated to dryness. Residue was taken up in toluene again and evaporated *in vacuo* (x 2). Resulting yellow oil was dried under high vacuum to give the crude sulfamoyl chloride which was used in next step as is.

Synthesis of 2-alkyl-1,2-thiazinan-5-one 1,1-dioxide (31c-d). In a modified literature procedure, a solution of isopropyl sulfamoyl chloride (1.26 g, 8 mmol) in anhydrous dichloromethane (10 ml) was added dropwise to a solution of (buta-1,3-dien-2-yloxy)trimethylsilane (1.14 g, 8 mmol) and triethylamine (1.34 ml, 9.6 mmol) in anhydrous dichloromethane (10 ml) at -78 ^{o}C under nitrogen. Resulting reaction mixture was allowed to stir while steadily increasing the temperature to rt over 6 h under nitrogen. Then, the reaction mixture was stirred at rt for 2 h. After the completion, the solvents were removed under reduced pressure and the crude product was purified by column chromatography using a gradient of 40% EtOAc/hexanes and the product is isolated in 20% yield.

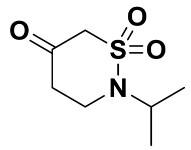

2-isopropyl-1,2-thiazinan-5-one 1,1-dioxide (31c)

^{1}H-NMR (400 MHz, $CDCl_3$): δ 4.44 (sep, 1H, J = 8.0 Hz), 3.93 (s, 2H), 3.40 (t, 2H, J = 8.0 Hz), 2.62 (t, 2H, J = 8.0 Hz), 1.26 (d, 6H, J = 8.0 Hz); ^{13}C-NMR (100 MHz, $CDCl_3$): δ 195.7, 65.6, 48.1, 41.1, 36.3, 21.1.

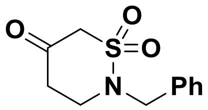

2-benzyl-1,2-thiazinan-5-one 1,1-dioxide (31d)

^{1}H-NMR (in $CDCl_3$, 400 MHz): δ 7.28 – 7.32 (m, 5H), 4.40 (s, 2H), 3.92 (s, 2H), 3.31 (t, 2H, J = 8.0 Hz), 2.42 (t, 2H, J = 8.0 Hz); ^{13}C-NMR (in $CDCl_3$, 100 MHz): δ 195.3, 134.9, 129.1, 128.6, 63.4, 51.1, 41.6, 37.5.

Synthesis of 2-isopropyl-2H-1,2-thiazin-5(6H)-one 1,1-dioxide (31e-f). In a modified literature procedure, a solution of isopropylsulfamoyl chloride (1.26 g, 8 mmol) in THF was added dropwise to a solution of (*E*)-((4-methoxybuta-1,3-dien-2-yl)oxy)trimethylsilane (1.38 g, 8 mmol) and triethylamine (1.34 ml, 9.6 mmol) in anhydrous THF at -78 ^{o}C under nitrogen. Resulting RM was allowed to stir while steadily increasing the temperature to rt over 6 h under nitrogen. Then, the reaction mixture was stirred at rt for 2 h afterwhich 2N HCl was added and reaction mixture was further stirred for 2 h. At this stage, THF was removed under reduced pressure and residue was extracted with ethyl acetate (3 x 50 ml). Combined organic layers were washed with brine (1 x 50 ml), dried over anhydrous magnesium sulfate, filtered and evaporated to give the crude product which was purified by column chromatography using a gradient of 40% EtOAc/hexanes and the pure product is isolated in 51% yield.

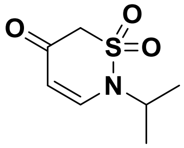

2-isopropyl-2H-1,2-thiazin-5(6H)-one 1,1-dioxide (31e)

^{1}H-NMR (in $CDCl_3$, 400 MHz): δ 7.25 (d, 1H, J = 8.0 Hz), 5.69 (d, 1H, J = 8.0 Hz), 4.62 (sep, 1H, J = 8.0 Hz), 4.14 (s, 2H), 1.41 (d, 6H, J = 8.0 Hz); ^{13}C-NMR (in $CDCl_3$, 100 MHz): δ 189.3, 143.0, 107.6, 61.9, 48.4, 22.6.

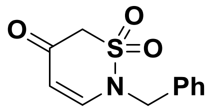

2-benzyl-2H-1,2-thiazin-5(6H)-one 1,1-dioxide (31f)

^{1}H-NMR (in $CDCl_3$, 400 MHz): δ 7.33 – 7.46 (m, 5H), 7.09 (d, 1H, J = 8.0 Hz), 5.60 (d, 1H, J = 8.0 Hz), 4.81 (s, 2H), 4.18 (s, 2H); ^{13}C-NMR (in $CDCl_3$, 100 MHz): δ 183.3, 146.4, 134.4, 129.4, 129.0, 128.4, 107.9, 61.7, 50.5.

Synthesis of 31g-h.

Preparation of ethyl 2-(methylsulfonamido)benzoate. In a modified literature procedure, ethyl 2-aminobenzoate (3.3 g, 20 mmol) was taken in anhydrous DCM (100 ml) and triethylamine (2.43 g , 24 mmol) was added to it. Resulting reaction mixture was cooled to 0 ^oC and methanesulfonyl chloride (2.75 g, 24 mmol) was added directly. The reaction mixture became cloudy after which it was allowed to stir at room temperature for 3 h under nitrogen. After the completion of reaction as indicated by TLC and LC-MS, reaction mixture was further diluted by dichloromethane to 150 ml and washed with water (3 x 50 ml) and brine. Organic layer was then dried over anhydrous magnesium sulfate, filtered and evaporated to dryness. Crude liquid was purified by column chromatography using 25% EtOAc/Hexane gradient to obtain pure product (pale yellow crystalline solid) in 91% yield.

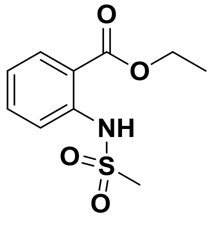

Ethyl 2-(methylsulfonamido)benzoate (95)

^{1}H-NMR (in $CDCl_3$, 400 MHz): δ 10.53 (s, 1H), 8.08 (dd, 1H, J1 = 8.0 Hz, J2 = 1.6 Hz), 7.76 (dd, 1H, J1 = 8.0 Hz, J2 = 0.9 Hz), 7.56 (dt, 1H, J1 = 8.0 Hz, J2 = 1.6 Hz), 7.13 (dt, 1H, J1 = 8.0 Hz, J2 = 1.1 Hz), 4.40 (q, 2H, J = 8.0 Hz), 3.06 (s, 3H), 1.42 (t, 3H, J = 8.0 Hz); ^{13}C-NMR (in $CDCl_3$, 100 MHz): δ 168.0, 140.9, 134.8, 131.5, 122.8, 118.1, 115.7, 61.8, 40.0, 14.2.

Preparation of ethyl 2-(*N*-alkylmethylsulfonamido)benzoate. Sulfonamide prepared above (2.43 g, 10 mmol) was taken in anhydrous DMF (10 ml) and anhydrous potassium carbonate (2.8 g, 20 mmol, 2 eq) was added to it. Resulting reaction mixture was stirred for 15 minutes followed by the addition of methyl iodide (2.13 g, 15 mmol). Resulting reaction mixture was heated and stirred at 75 ^{o}C for 5 h under nitrogen. After completion, the reaction mixture was neutralized by the addition of water and aqueous layer was extracted with ethyl acetate (5 x 25 ml). Combined organic layers were washed with water (2 x 50 ml) and brine (1 x 50 ml). Organic layer was then dried over anhydrous magnesium sulfate, filtered and evaporated to dryness. Crude liquid was purified by column chromatography using 25% - 40% EtOAc/Hexane gradient to obtain pure product in quantitative yield.

Preparation of 1-alkyl-1H-benzo[c][1,2]thiazin-4(3H)-one 2,2-dioxide (31g-h). To a stirred solution of NaH (60% in mineral oil, 0.6 g, 15 mmol, 2 eq.) in dry DMF (5 ml) was added dropwise a solution of ethyl 2-(*N*-methylmethylsulfonamido)benzoate (1.9 g, 7.5 mmol) in DMF (10 ml). Resulting reaction mixture was allowed to stir under nitrogen with monitoring. The reaction mixture color changed from white to red-brown. After the completion (7 h), the reaction mixture was acidified to pH 3 with 2N HCl. Aqueous layer was extracted with ethyl acetate (5 x 25 ml). Combined organic layers were washed with water (2 x 50 ml) and brine (1 x 50 ml). Organic layer was then dried over anhydrous magnesium sulfate, filtered and evaporated to dryness. Crude liquid was purified by column chromatography using 20% EtOAc/Hexane gradient followed by 100% DCM gradient to obtain pure product as a cream solid in 70% yield.

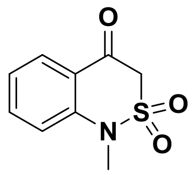

1-methyl-1H-benzo[c][1,2]thiazin-4(3H)-one 2,2-dioxide (31g)

^{1}H-NMR (in $CDCl_3$, 400 MHz): δ 8.13 (dd, 1H, J1 = 8.0 Hz, J2 = 1.3 Hz), 7.68 (dt, 1H, J1 = 8.0 Hz, J2 = 1.5 Hz), 7.26 (t, 1H, J = 8.0 Hz), 7.17 (d, 1H, J = 8.0 Hz), 4.33 (s, 2H), 3.44 (s, 3H); ^{13}C-NMR: δ 184.0, 144.3, 136.7, 129.5, 123.7, 123.0, 117.3, 61.6, 31.1.

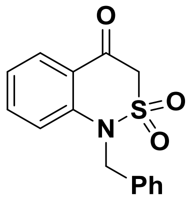

1-benzyl-1H-benzo[c][1,2]thiazin-4(3H)-one 2,2-dioxide (31h)

^{1}H-NMR (in $CDCl_3$, 400 MHz): δ 8.13 (dd, 1H, J1 = 8.0 Hz, J2 = 1.6 Hz), 7.58 (dt, 1H, J1 = 8.0 Hz, J2 = 1.7 Hz), 7.30 – 7.40 (m, 5H), 7.24 (dt, 1H, J1 = 8.0 Hz, J2 = 1.0 Hz), 7.16 (dd, 1H, J1 = 8.0 Hz, J2 = 0.6 Hz), 5.18 (s, 2H), 4.26 (s, 2H); ^{13}C-NMR: δ 184.2, 143.6, 136.5, 135.3, 129.4, 129.1, 128.2, 127.1, 124.4, 123.9, 119.8, 61.8, 51.3.

Scheme S18. Synthesis of the nucleophiles in chart 6

Synthesis of 4-benzylcyclopentane-1,3-dione (21b)

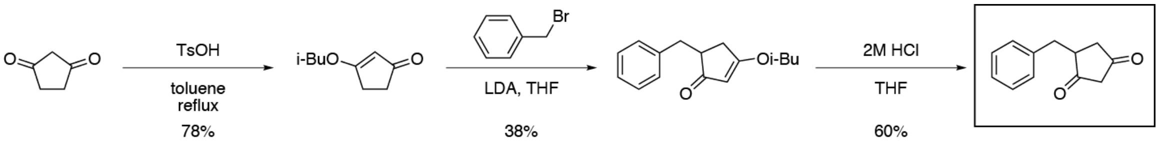

^{1}H-NMR (in $CDCl_3$, 400 MHz): δ 11.73 (s, 1H), 7.17 – 7.35 (m, 5H), 5.26 (s, 1H), 3.25 (dd, 1H, J1 = 16.0 Hz, J2 = 4.4 Hz); 2.95 – 3.05 (m, 1H), 2.52 – 2.65 (m, 2H), 2.28 – 2.35 (m, 1H). LRMS: MF - $C_{12}H_{12}O_2$, MW_{exp} = 188.23, MW_{obs} = 189.1 (M^++1).

Synthesis of 4-benzylidenecyclopentane-1,3-dione (21c)

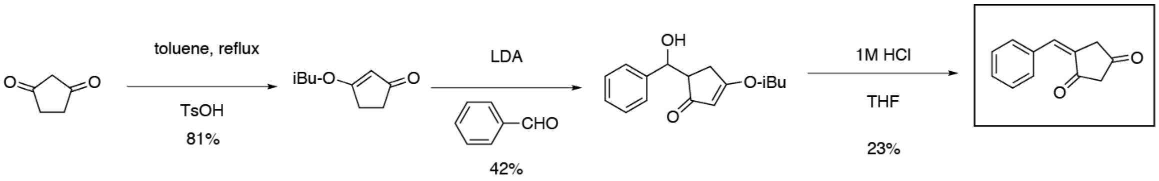

^{1}H-NMR (in MeOH-d4, 400 MHz): δ 7.23 – 7.45 (m, 5H), 5.28 (s, 1H), 3.00 (dd, 1H, J1 = 4.0 Hz, J2 = 2.4 Hz), 2.59 (dd, 1H, J1 = 18.0 Hz, J2 = 2.8 Hz), 2.21 (dd, 1H, J1 = 18.0 Hz, J2 = 7.2 Hz); LRMS: MF - $C_{12}H_{10}O_2$, MW_{exp} = 186.21, MW_{obs} = 187.2 (M^++1).

Synthesis of 4-phenylcyclopentane-1,3-dione (21d)

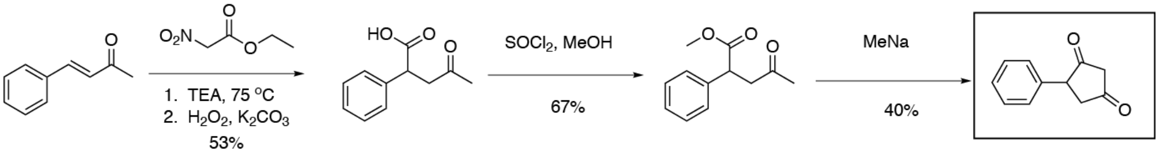

^{1}H-NMR (in MeOH-d4, 400 MHz): δ 7.23 – 7.35 (m, 5H), 4.01 (dd, 1H, J1 = 10.0 Hz), J2 = 4.0 Hz), 3.25 – 3.40 (m, 2H), 2.77 (dd, 1H, J1 = 18.0 Hz, J2 = 4.4 Hz); LRMS: MF - $C_{11}H_{10}O_2$, MW_{exp} = 174.20, MW_{obs} = 175.1 (M^++1).

Synthesis of 1-benzylpyrrolidine-2,4-dione (32c)

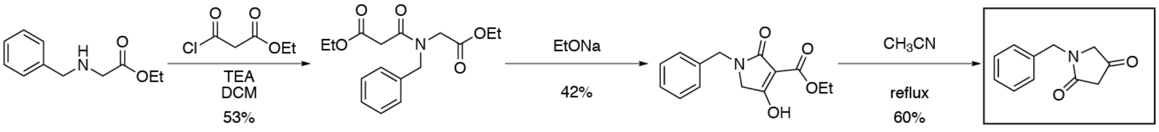

^{1}H-NMR (in $CDCl_3$, 400 MHz): δ 7.27 – 7.40 (m, 5H), 4.65 (s, 2H), 3.75 (s, 2H), 3.12 (s, 2H); LRMS: MF - $C_{11}H_{11}NO_2$, MW_{exp} = 189.21, MW_{obs} = 190.1 (M^++1).

Synthesis of 1-phenylpyrrolidine-2,4-dione (32d)

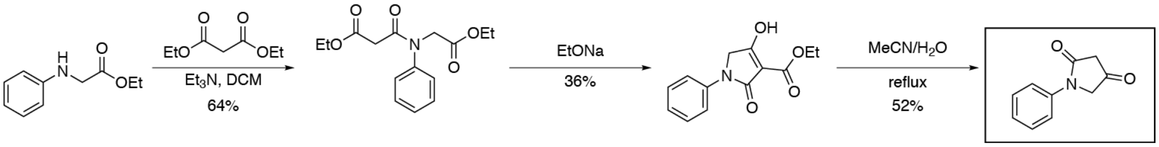

^{1}H-NMR (in DMSO-*d6*, 400 MHz): (Mixture of keto-enol tautomers) δ 11.90 (bs, 1H), 6.98 – 7.75 (m, 5H), 4.97 (s, 1H), 4.36 (s, 2H); LRMS: MF - $C_{10}H_9NO_2$, MW_{exp} = 175.19, MW_{obs} = 176.1 (M^++1).

Synthesis of 5-benzyldihydrothiophen-3(2H)-one 1,1-dioxide(33b)

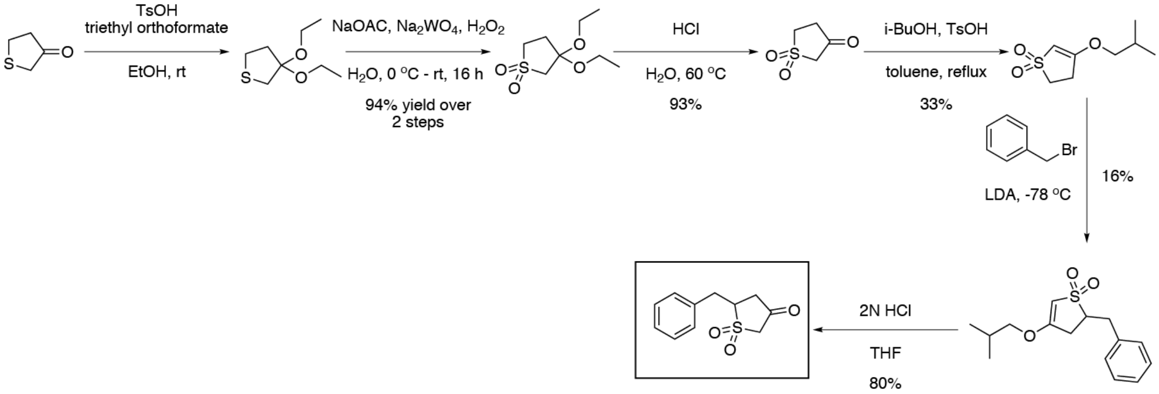

^{1}H-NMR (in $CDCl_3$, 400 MHz): δ 7.20 – 7.40 (m, 5H), 3.84 – 3.88 (m, 1H), 3.53 (s, 2H), 3.47 – 3.51 (m, 1H), 2.80 – 2.96 (m, 3H); LRMS: MF - $C_{11}H_{12}O_3S$, MW_{exp} = 224.27, MW_{obs} = 225.0 (M^++1).

Synthesis of 5-benzylidenedihydrothiophen-3(2H)-one 1,1-dioxide (33c)

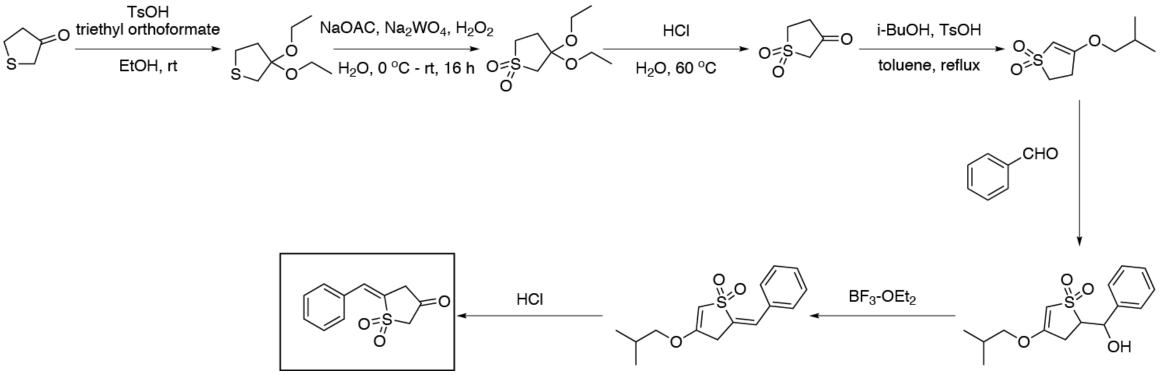

^{1}H-NMR (in $CDCl_3$, 400 MHz): δ 7.95 – 8.03 (m, 3H), 7.50 – 7.62 (m, 3H), 3.52 (t, 2H, J = 8.0 Hz), 3.15 (t, 2H, J = 8.0 Hz); LRMS: MF - $C_{11}H_{10}O_3S$, MW_{exp} = 222.26, MW_{obs} = 223.0 (M^++1).

Synthesis of 5-phenyldihydrothiophen-3(2H)-one 1,1-dioxide (33d)

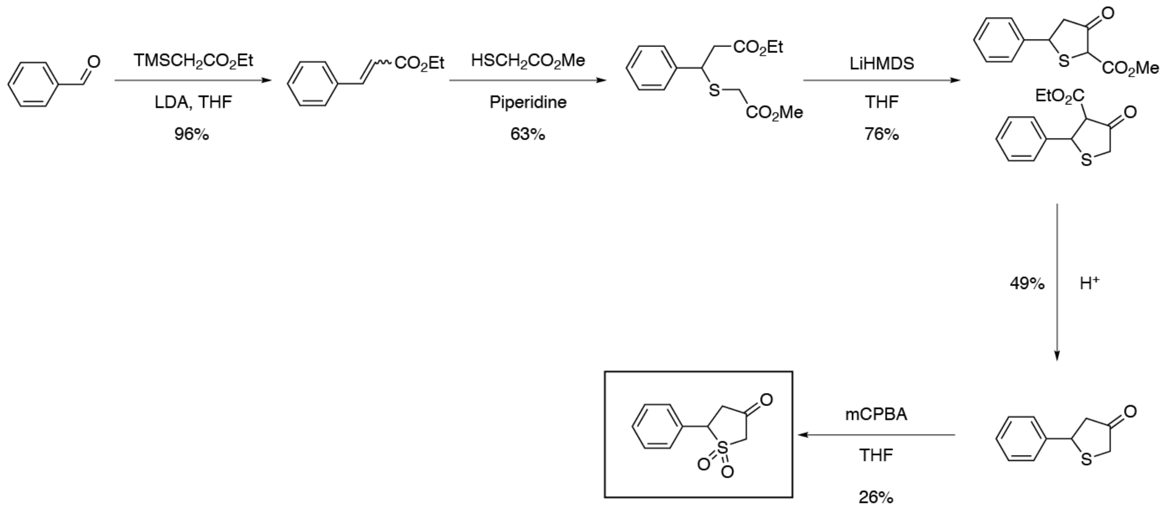

^{1}H-NMR (in $CDCl_3$, 400 MHz): δ 7.40 – 7.50 (m, 5H), 4.84 (t, 1H, J = 8.0 Hz), 3.85 (q, 2H, J = 18.0 Hz), 3.40 (t, 2H, J = 8.0 Hz); LRMS: MF - $C_{10}H_{10}O_3S$, MW_{exp} = 210.25, MW_{obs} = 211.0 (M^++1).

Synthesis of 2-benzylisothiazolidin-4-one 1,1-dioxide (33f)

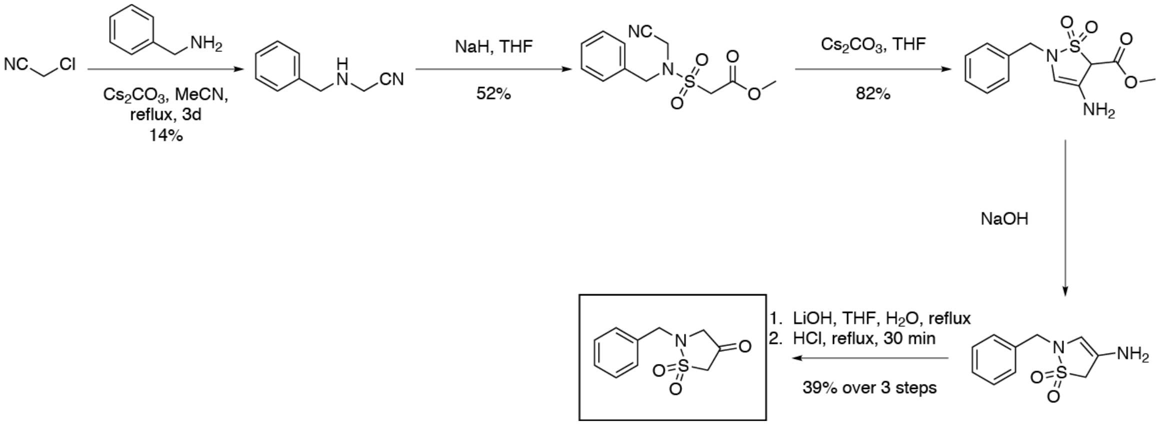

^{1}H-NMR (in $CDCl_3$, 400 MHz): δ 7.32 – 7.42 (m, 5H), 4.39 (s, 2H), 3.83 (s, 2H), 3.68 (s, 2H); LRMS: MF - $C_{10}H_{11}NO_3S$, MW_{exp} = 225.26, MW_{obs} = 226.0 (M^++1).

Synthesis of 2-phenylisothiazolidin-4-one 1,1-dioxide (33g)

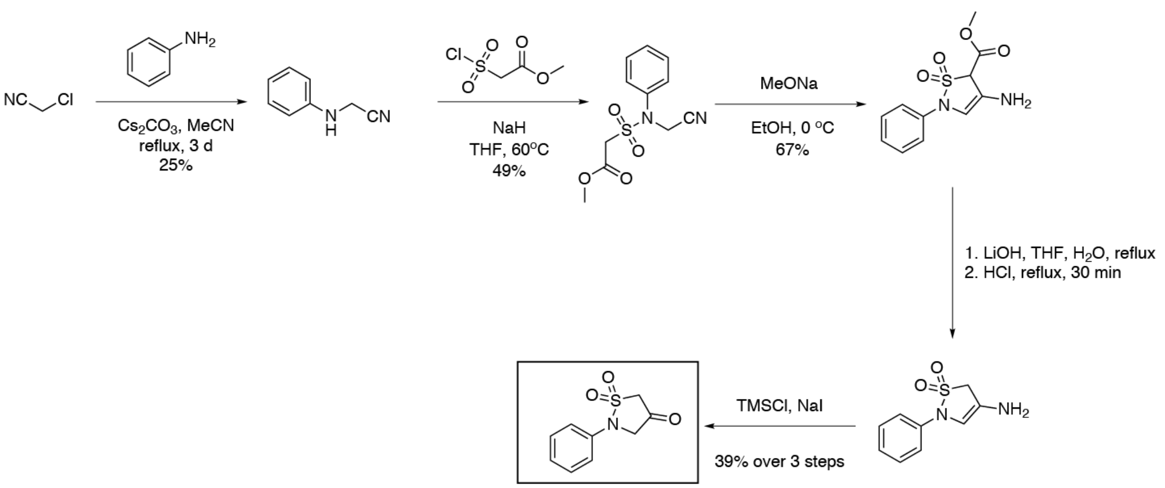

^{1}H-NMR (in $CDCl_3$, 400 MHz): δ 7.28 – 7.47 (m, 5H), 4.37 (s, 2H), 4.06 (s, 2H); LRMS: MF – $C_9H_9NO_3S$, MW_{exp} = 211.24, MW_{obs} = 212.0 (M^++1).

Scheme S19. Cross-reactivity profiles of various cyclic nucleophiles with biological electrophiles/nucleophiles.

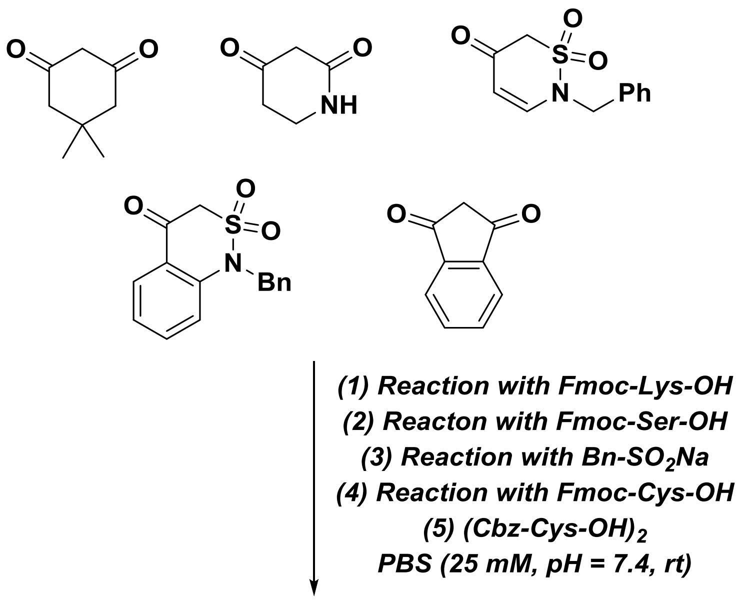

Procedure: A 100 mM solution of each nucleophile was prepared in DMSO. Solutions of Fmoc-Lys-OH (100 mM, in PBS buffer), Fmoc-Ser-OH (100 mM in DMSO), Bn-SO_2Na (100 mM in PBS buffer), Fmoc-Cys-OH (100 mM in DMSO) and $(Cbz\text{-}Cys\text{-}OH)_2$ (100 mM in DMSO) were prepared. 10 μL of nucleophile solution and 10 μL of each biological nucleophile/electrophile solution was added to 980 μL of PBS buffer (25 mM, pH = 7.4, rt). Effective concentrations were as follow:

(1) Cyclic Nucelophile = 1 mM

(2) Biological nucleophile/electrophile = 1mM

(3) Buffer = 25 mM

Each reaction mixture was analyzed by LC-MS at 2 h intervals.

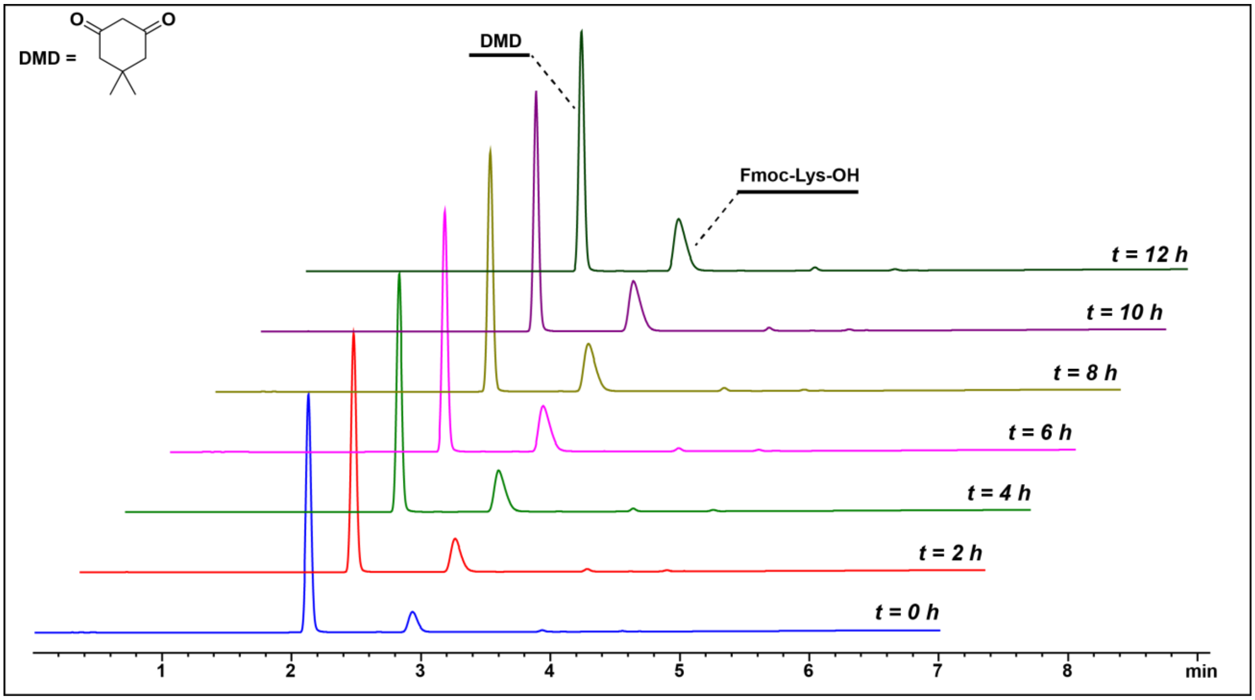

Figure S1. Reaction of dimedone (DMD, 1 mM) with Fmoc-Lys-OH (1 mM) in PBS (25 mM, pH = 7.4, rt) over 12 h.

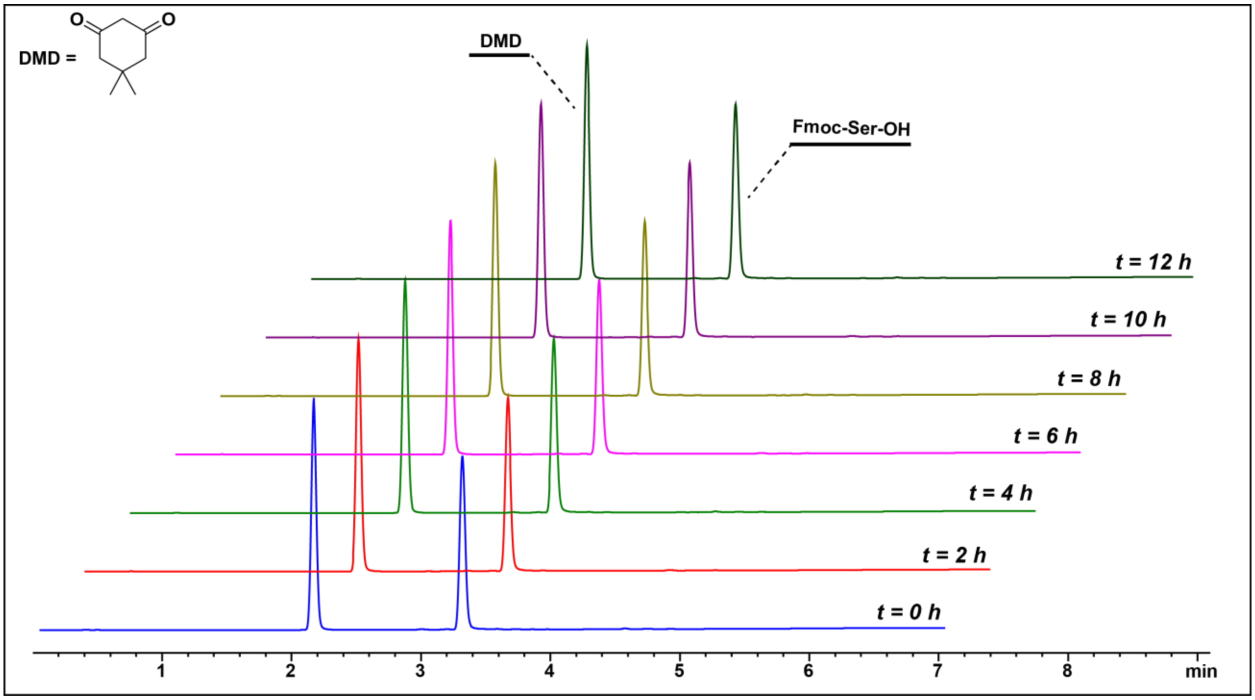

Figure S2. Reaction of dimedone (DMD, 1 mM) with Fmoc-Ser-OH (1 mM) in PBS (25 mM, pH = 7.4, rt) over 12 h.

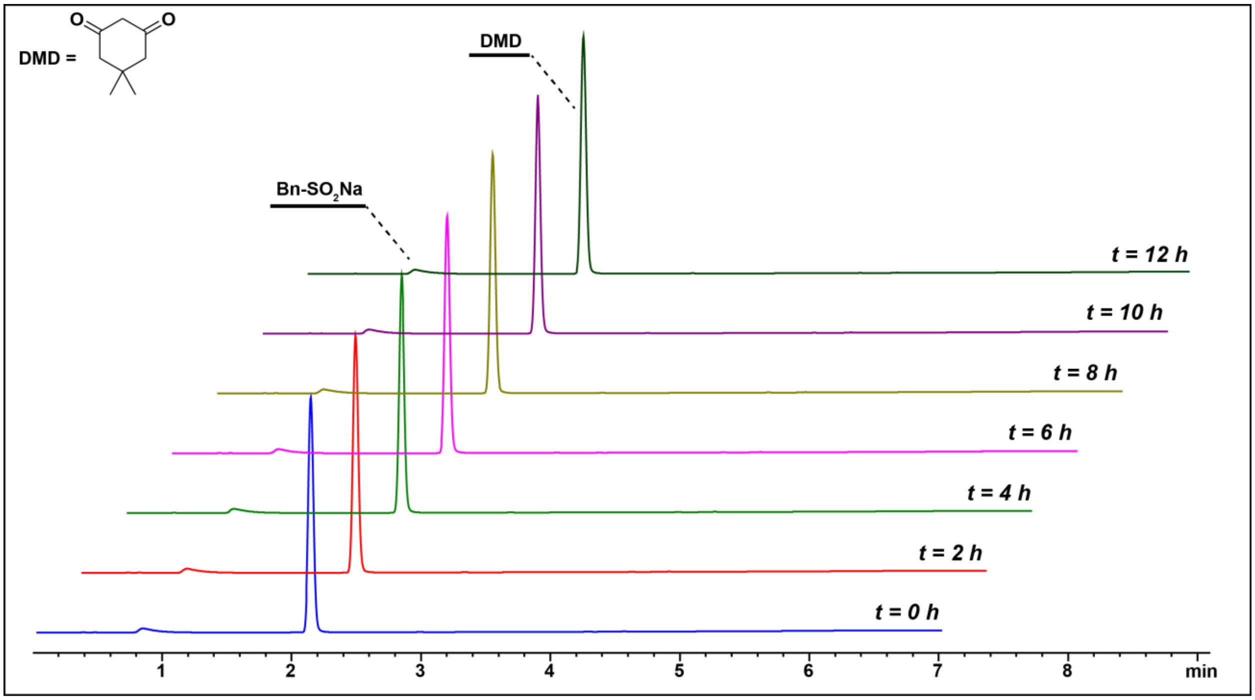

Figure S3. Reaction of dimedone (DMD, 1 mM) with benzyl sulfinate sodium salt (1 mM) in PBS (25 mM, pH = 7.4, rt) over 12 h.

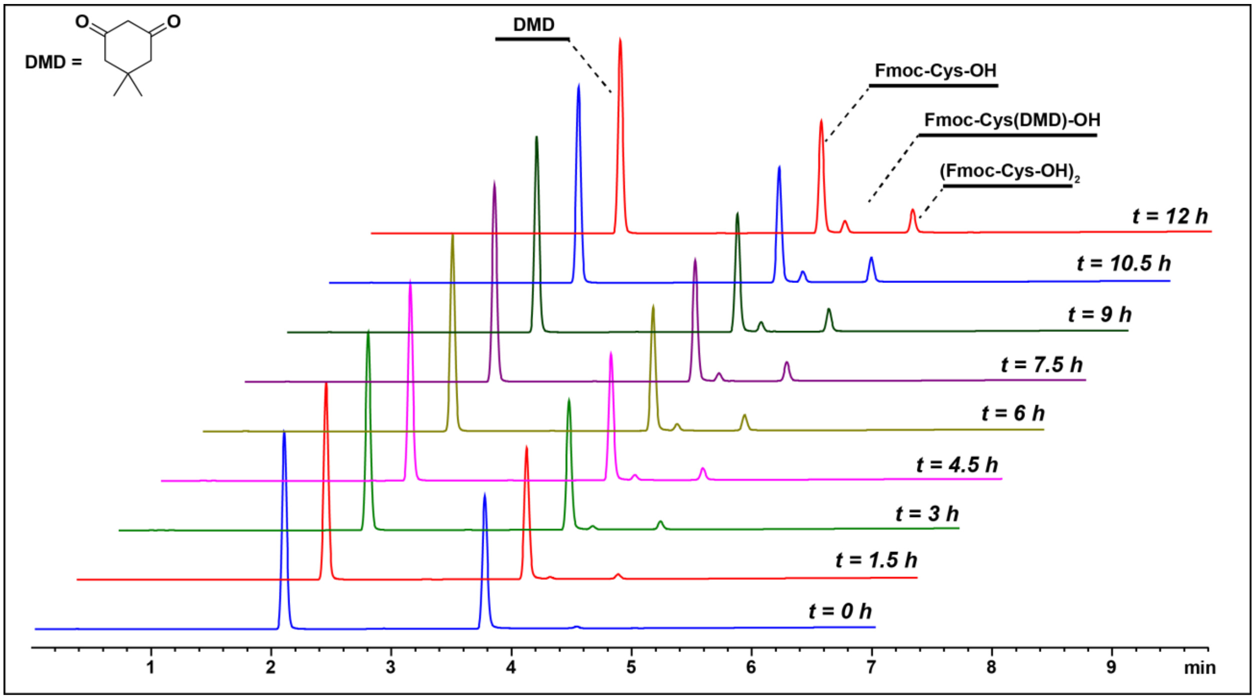

Figure S4. Reaction of dimedone (DMD, 1 mM) with Fmoc-Cys-OH (1 mM) in PBS (25 mM, pH = 7.4, rt) over a course of 12 h.

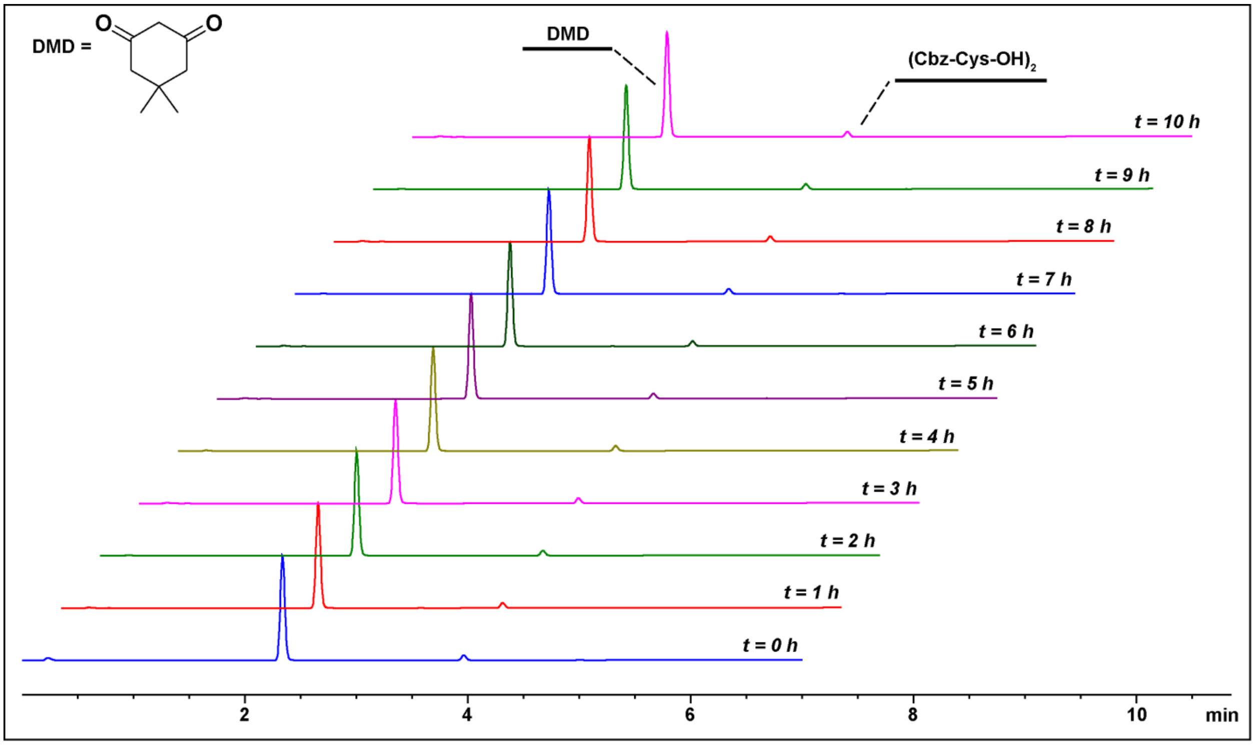

Figure S5. Reaction of dimedone (DMD, 1 mM) with (Cbz-Cys-OH) (1 mM) in PBS (25 mM, pH = 7.4, rt) over a course of 10 h.

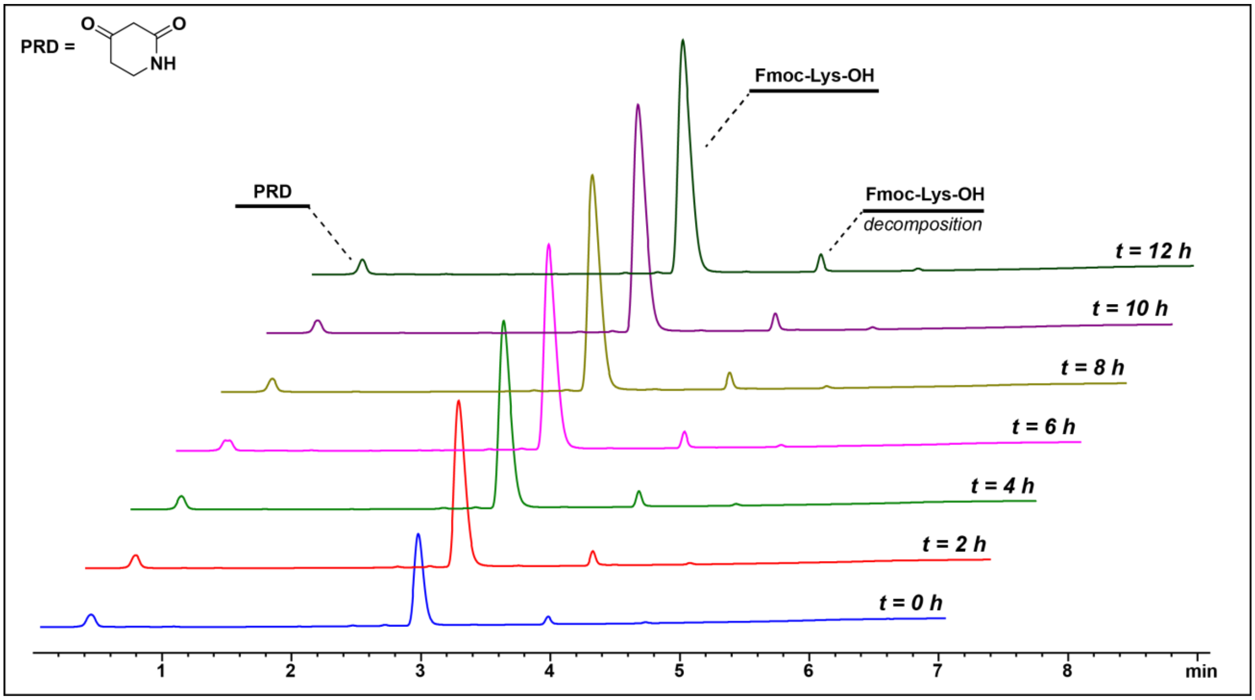

Figure S6. Reaction of 2,4-piperidinedione (PRD, 1 mM) with Fmoc-Lys-OH (1 mM) in PBS (25 mM, pH = 7.4, rt) over 12 h.

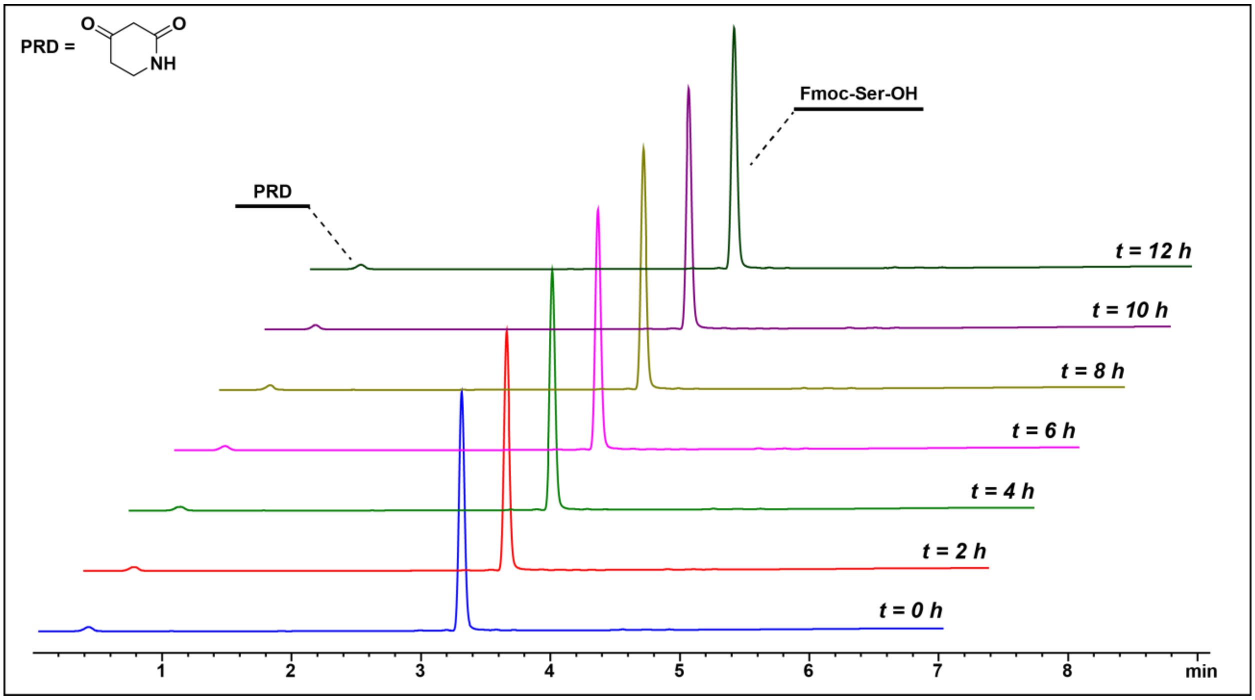

Figure S7. Reaction of 2,4-piperidinedione (PRD, 1 mM) with Fmoc-Ser-OH (1 mM) in PBS (25 mM, pH = 7.4, rt) over 12 h.

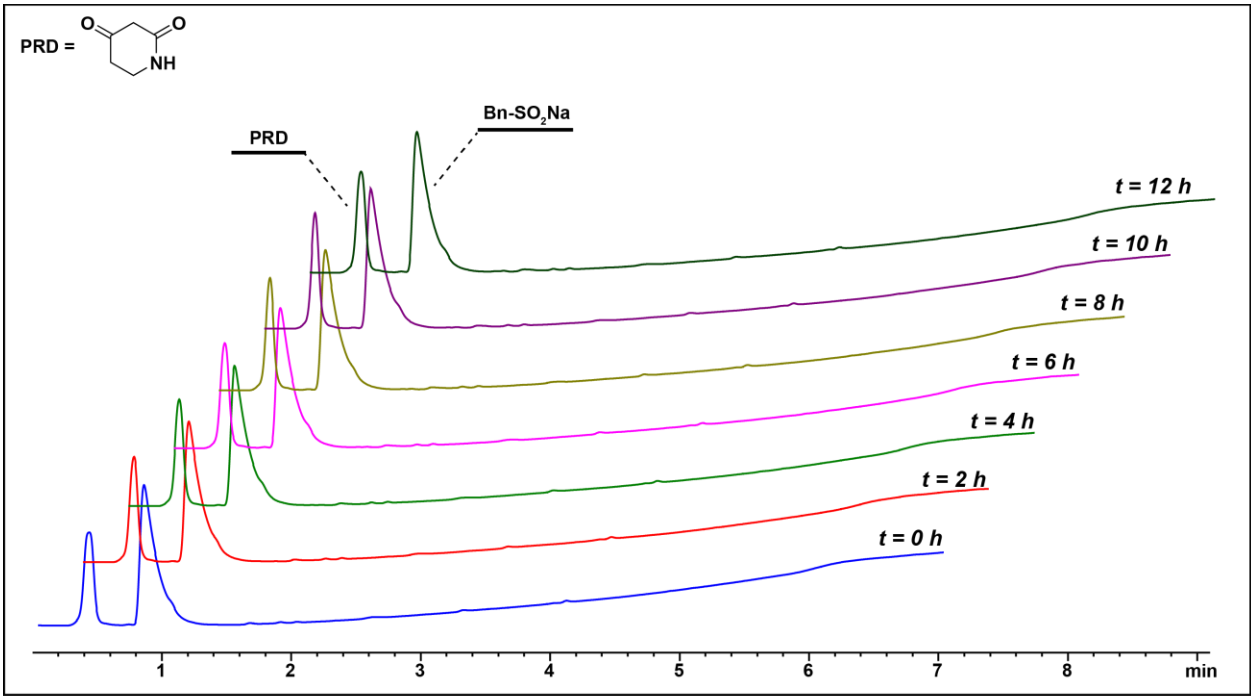

Figure S8. Reaction of 2,4-piperidinedione (PRD, 1 mM) with benzyl sulfinate sodium salt (1 mM) in PBS (25 mM, pH = 7.4, rt) over 12 h.

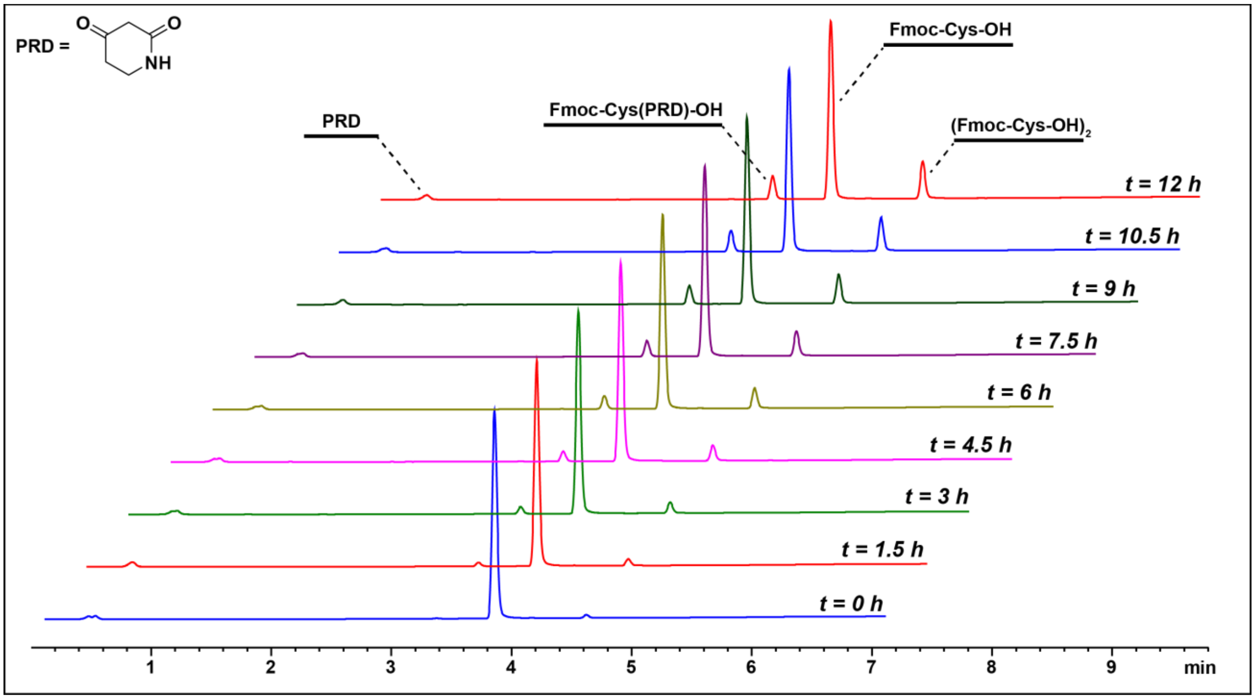

Figure S9. Reaction of 2,4-piperidinedione (PRD, 1 mM) with Fmoc-Cys-OH (1 mM) in PBS (25 mM, pH = 7.4, rt) over a course of 12 h.

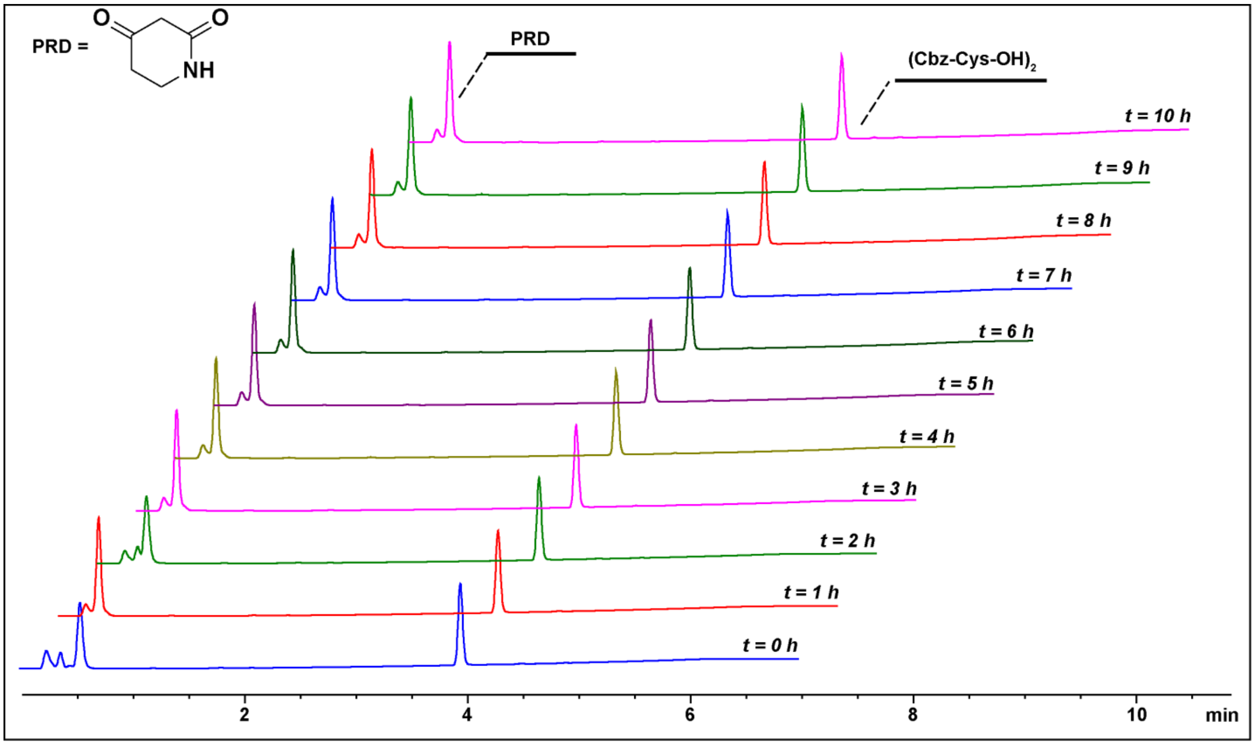

Figure S10. Reaction of 2,4-piperidinedione (PRD, 1 mM) with (Cbz-Cys-OH) (1 mM) in PBS (25 mM, pH = 7.4, rt) over a course of 10 h.

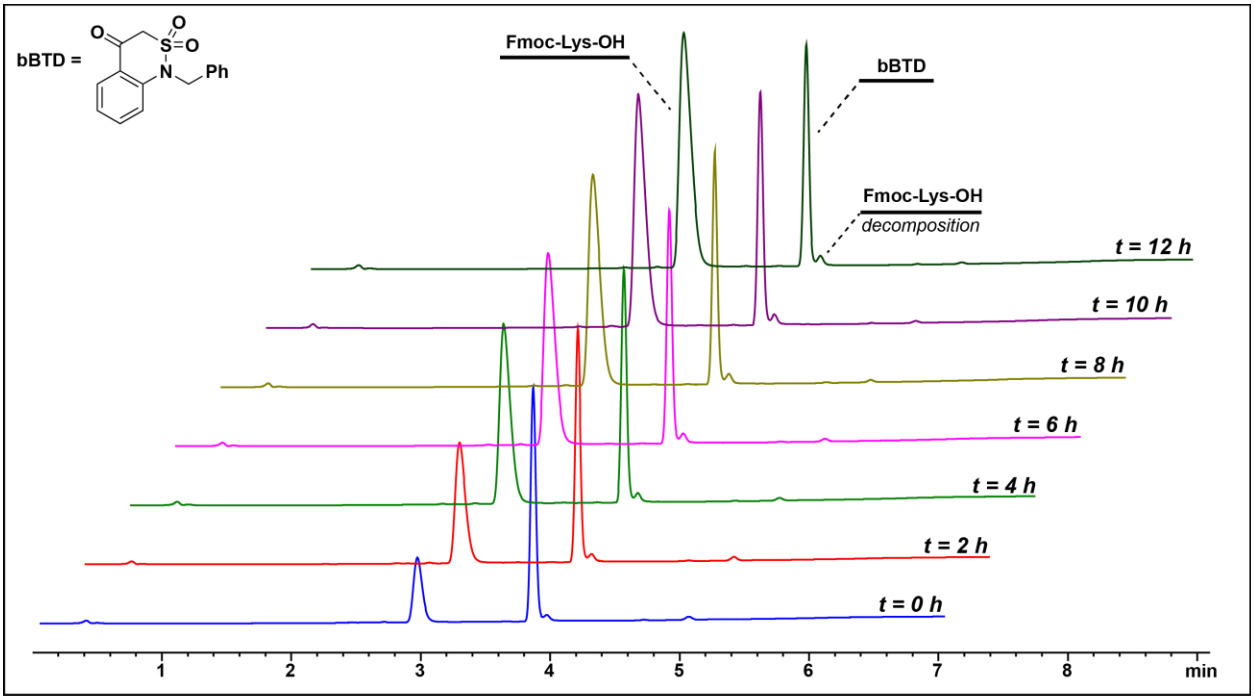

Figure S11. Reaction of bBTD (1 mM) with Fmoc-Lys-OH (1 mM) in PBS (25 mM, pH = 7.4, rt) over 12 h.

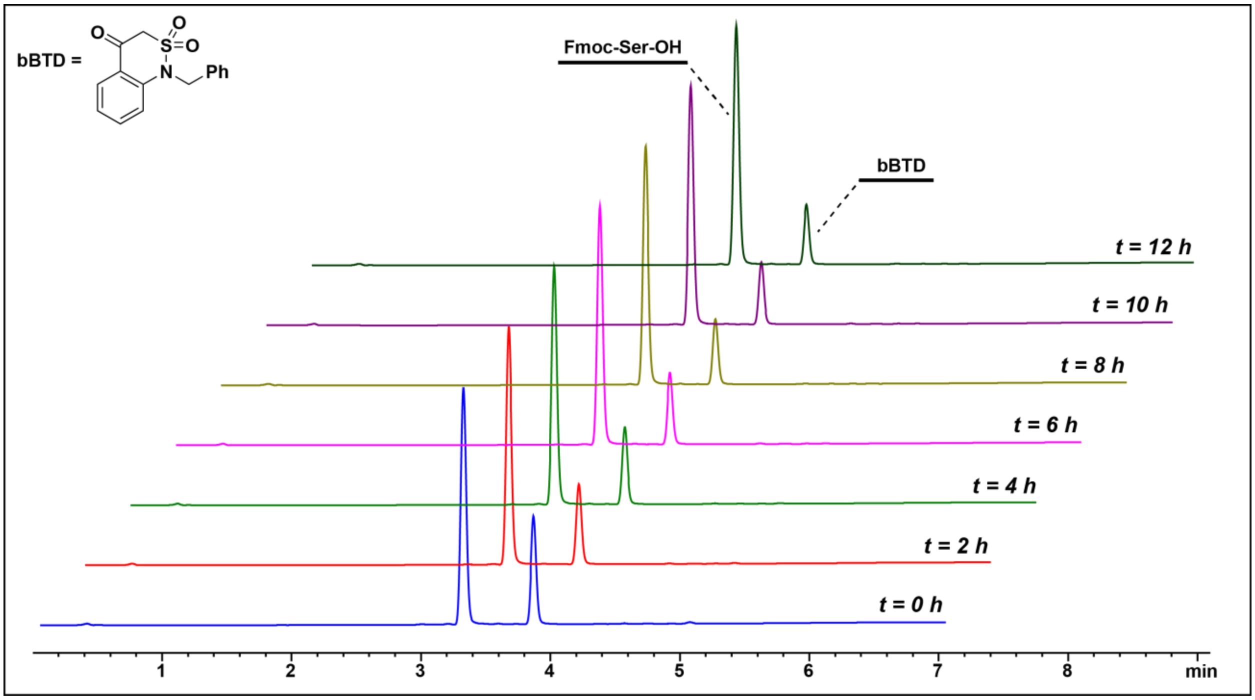

Figure S12. Reaction of bBTD (1 mM) with Fmoc-Ser-OH (1 mM) in PBS (25 mM, pH = 7.4, rt) over 12 h.

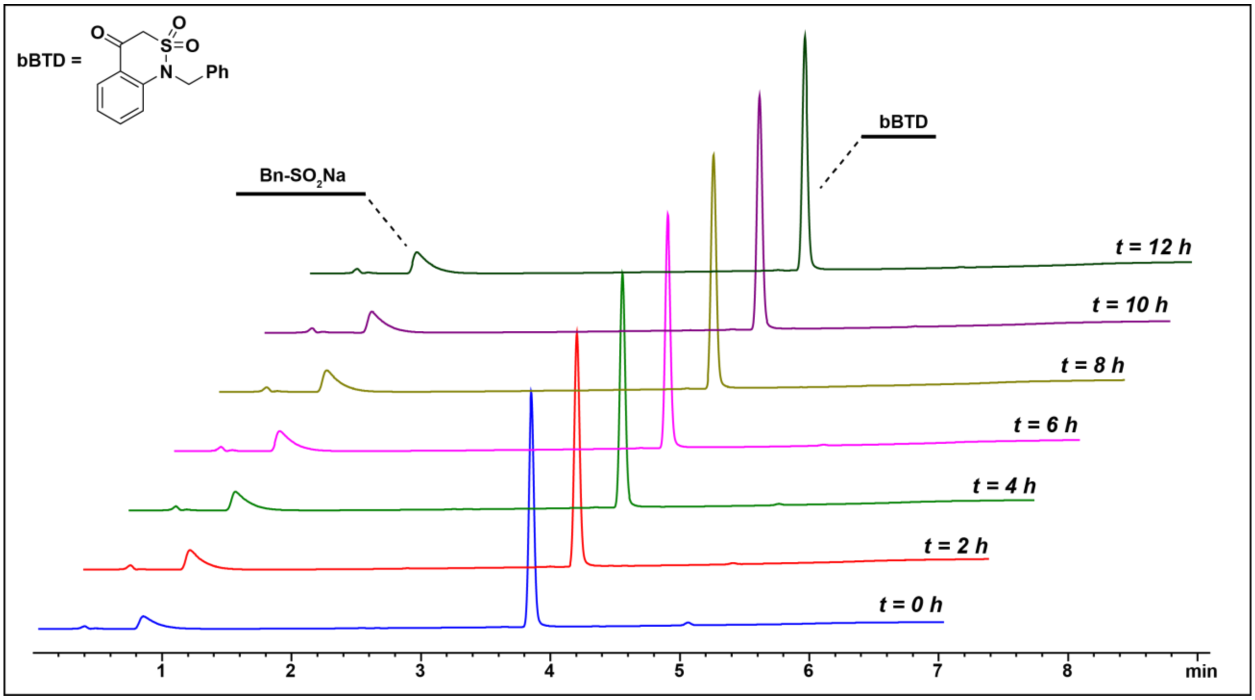

Figure S13. Reaction of bBTD (1 mM) with benzyl sulfinate sodium salt (1 mM) in PBS (25 mM, pH = 7.4, rt) over 12 h.

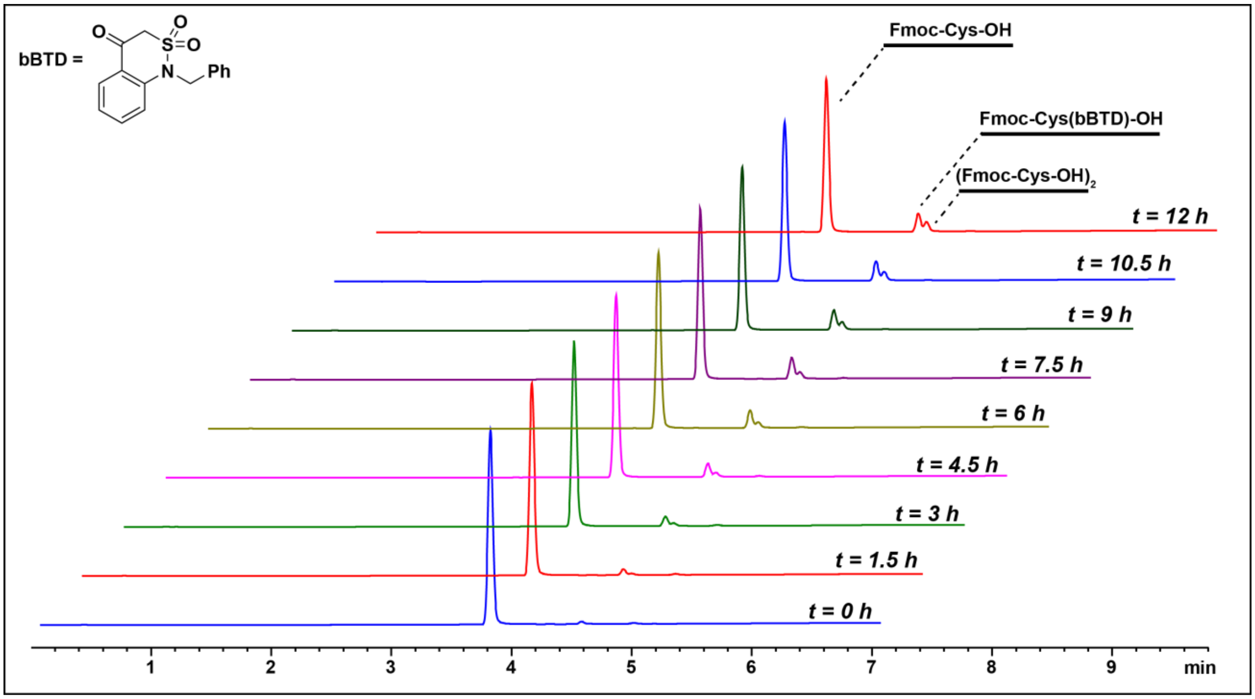

Figure S14. Reaction of bBTD (1 mM) with Fmoc-Cys-OH (1 mM) in PBS (25 mM, pH = 7.4, rt) over a course of 12 h.

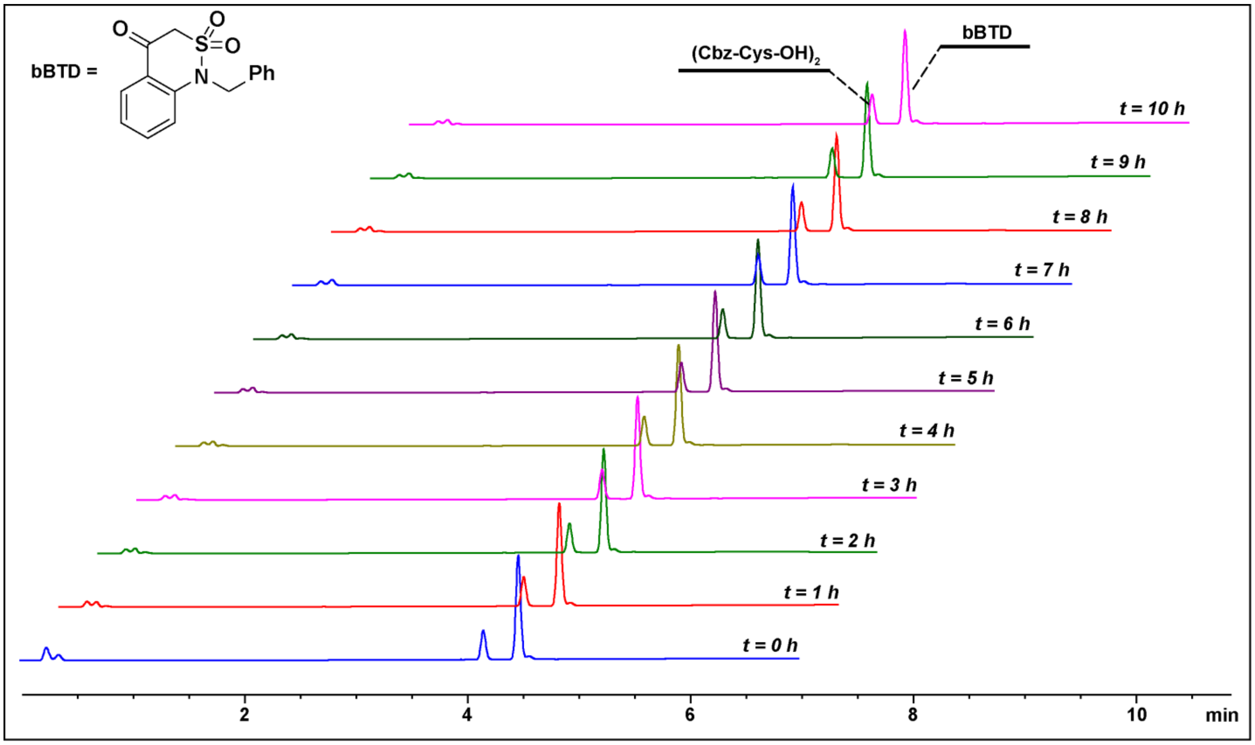

Figure S15. Reaction of bBTD (1 mM) with (Cbz-Cys-OH) (1 mM) in PBS (25 mM, pH = 7.4, rt) over a course of 10 h.

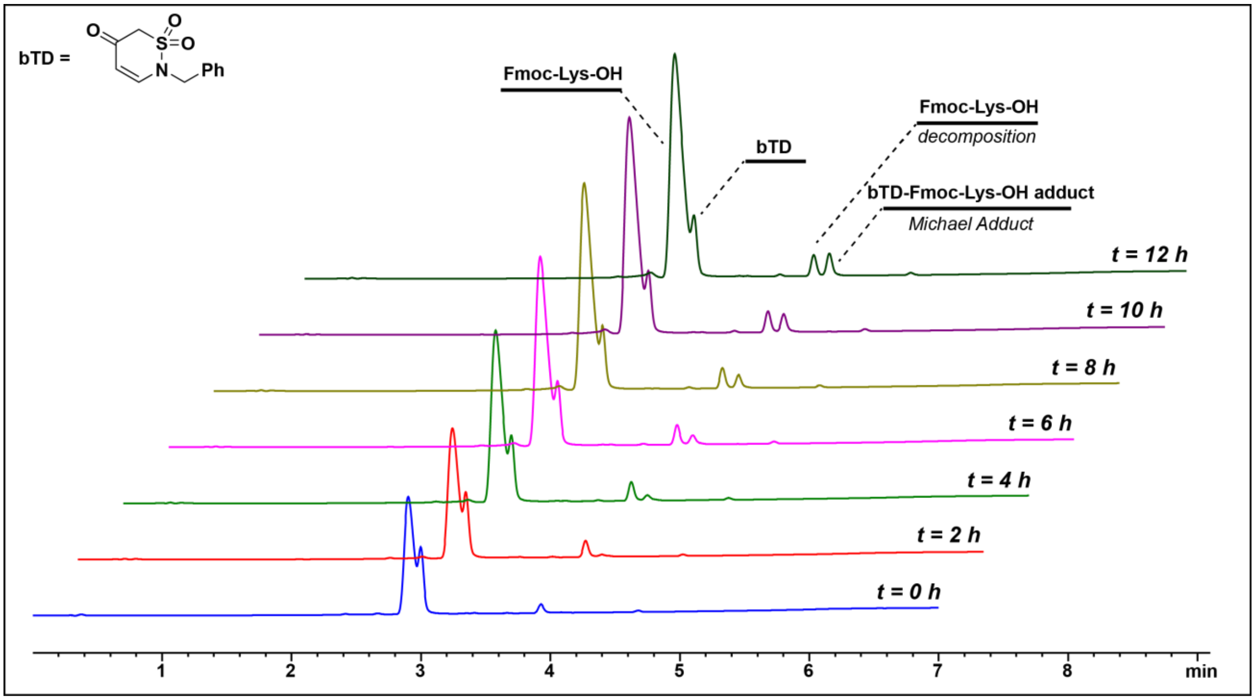

Figure S16. Reaction of bTD (1 mM) with Fmoc-Lys-OH (1 mM) in PBS (25 mM, pH = 7.4, rt) over 12 h.

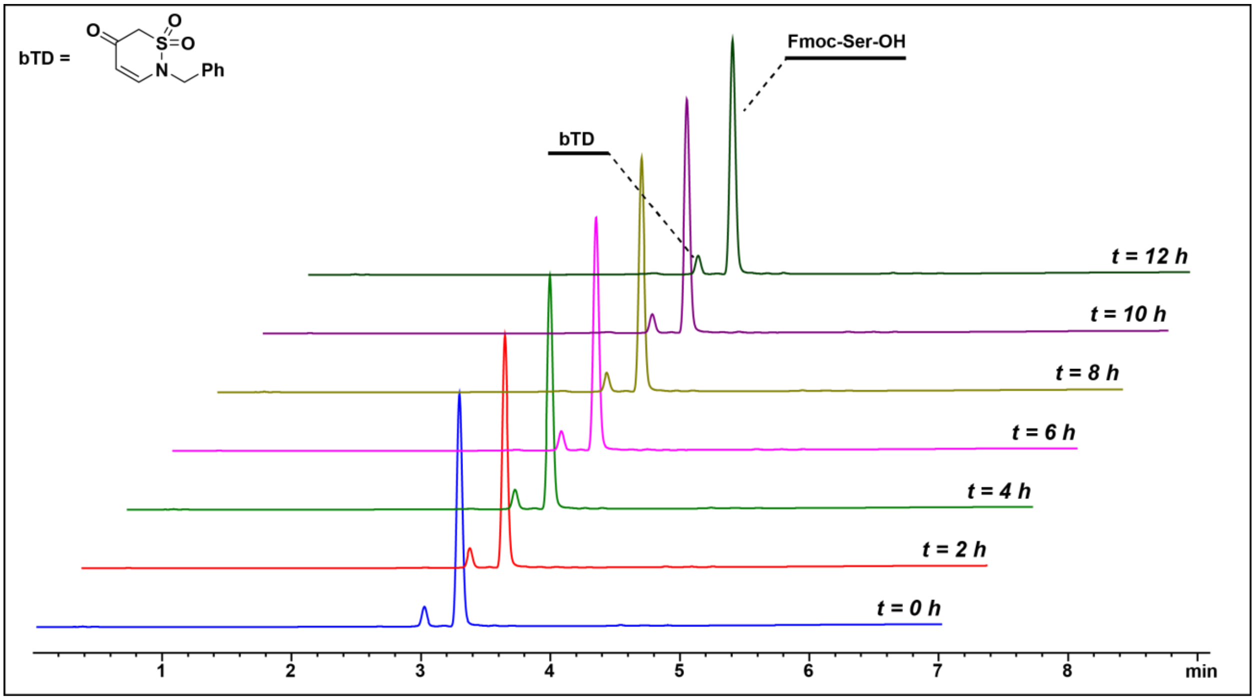

Figure S17. Reaction of bTD (1 mM) with Fmoc-Ser-OH (1 mM) in PBS (25 mM, pH = 7.4, rt) over 12 h.

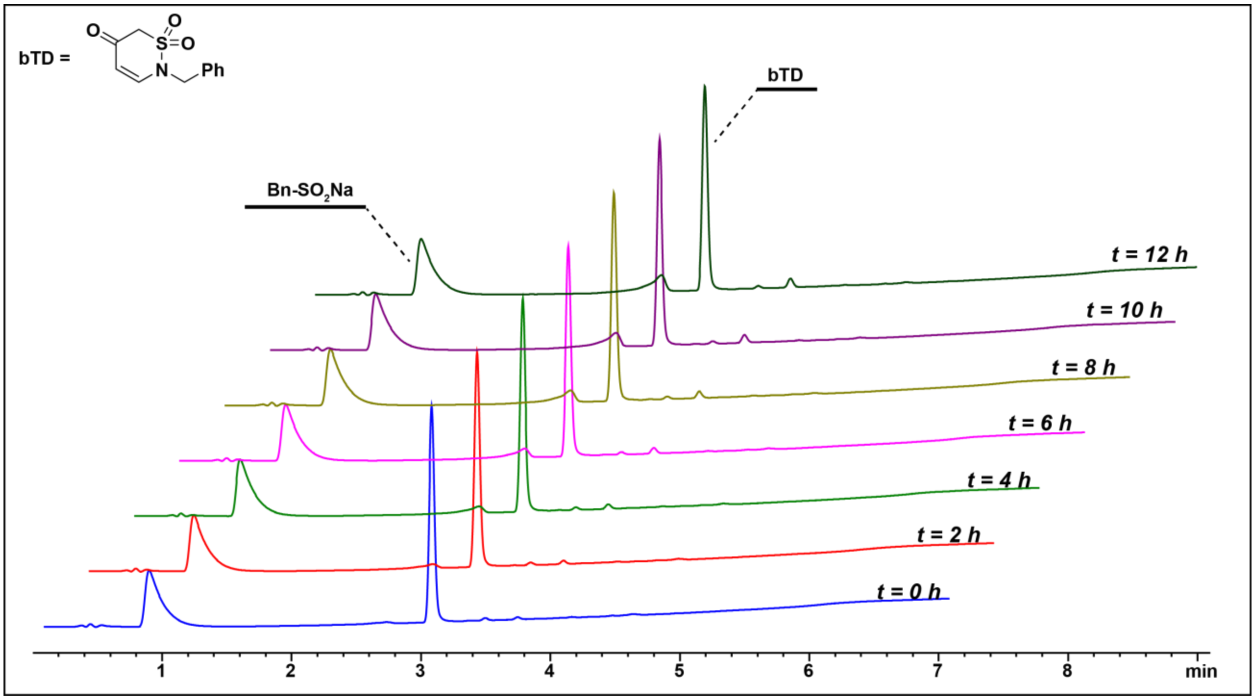

Figure S18. Reaction of bTD (1 mM) with benzyl sulfinate sodium salt (1 mM) in PBS (25 mM, pH = 7.4, rt) over 12 h.

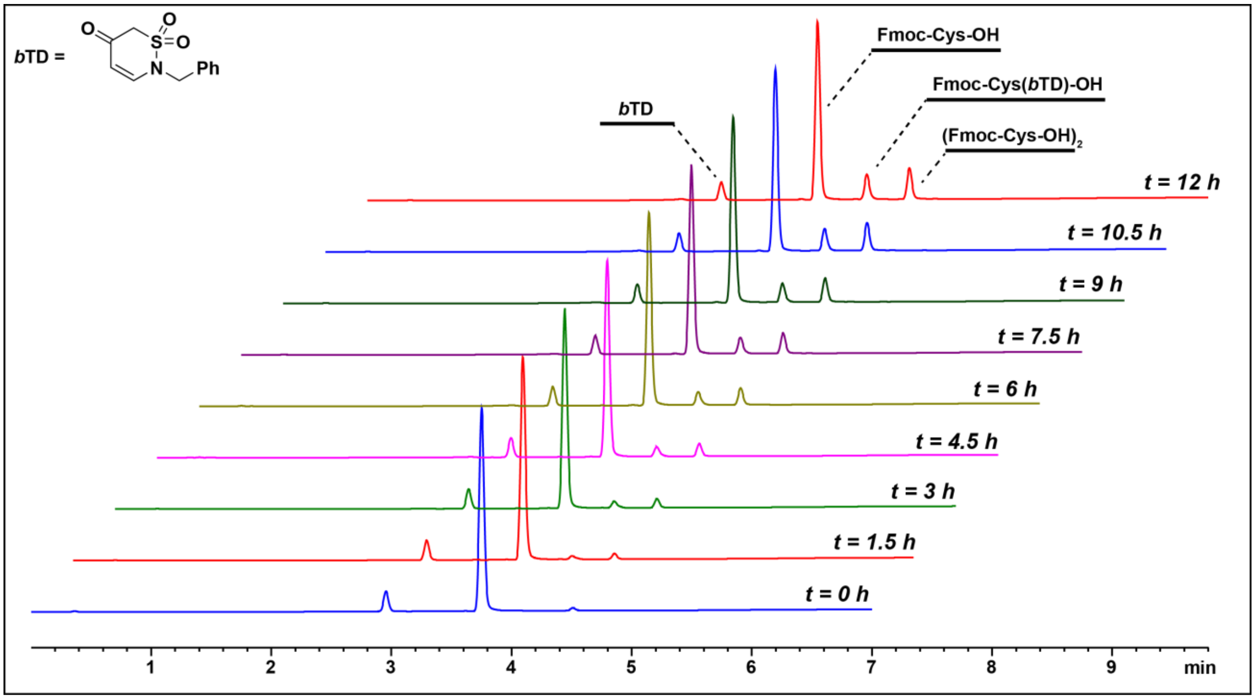

Figure S19. Reaction of bTD (1 mM) with Fmoc-Cys-OH (1 mM) in PBS (25 mM, pH = 7.4, rt) over a course of 12 h.

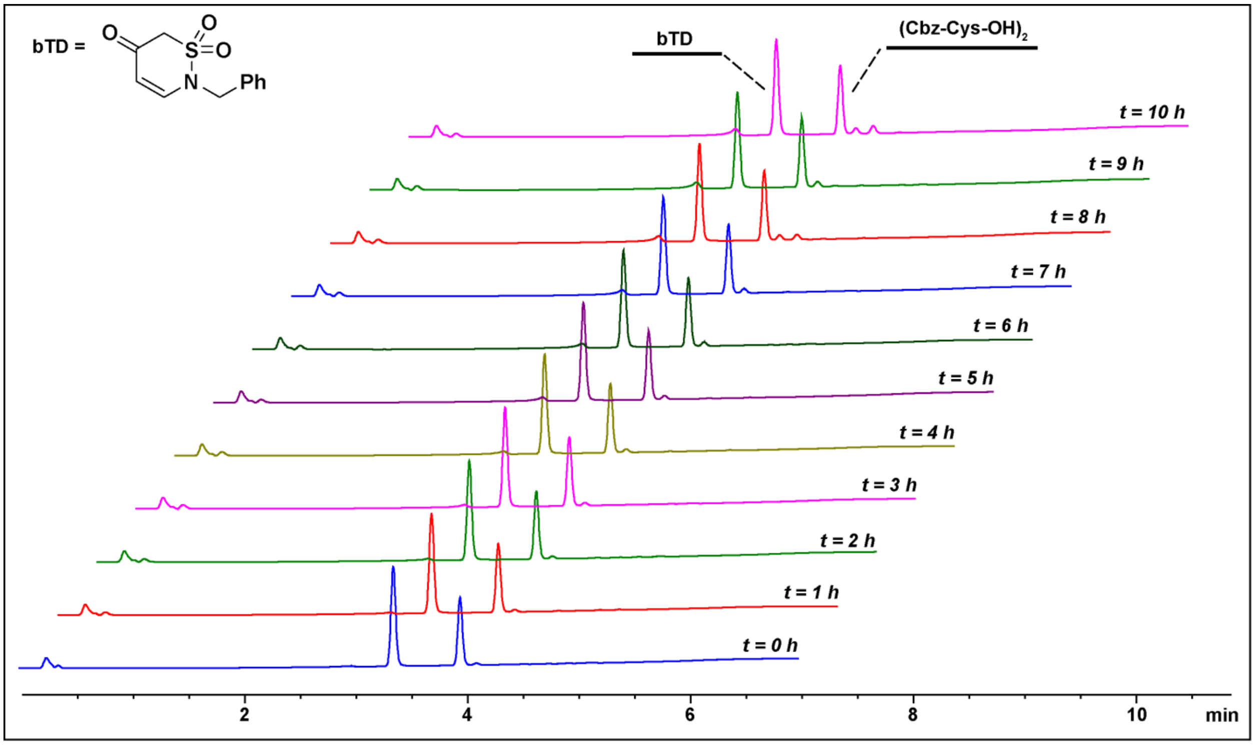

Figure S20. Reaction of bTD (1 mM) with (Cbz-Cys-OH) (1 mM) in PBS (25 mM, pH = 7.4, rt) over a course of 10 h.

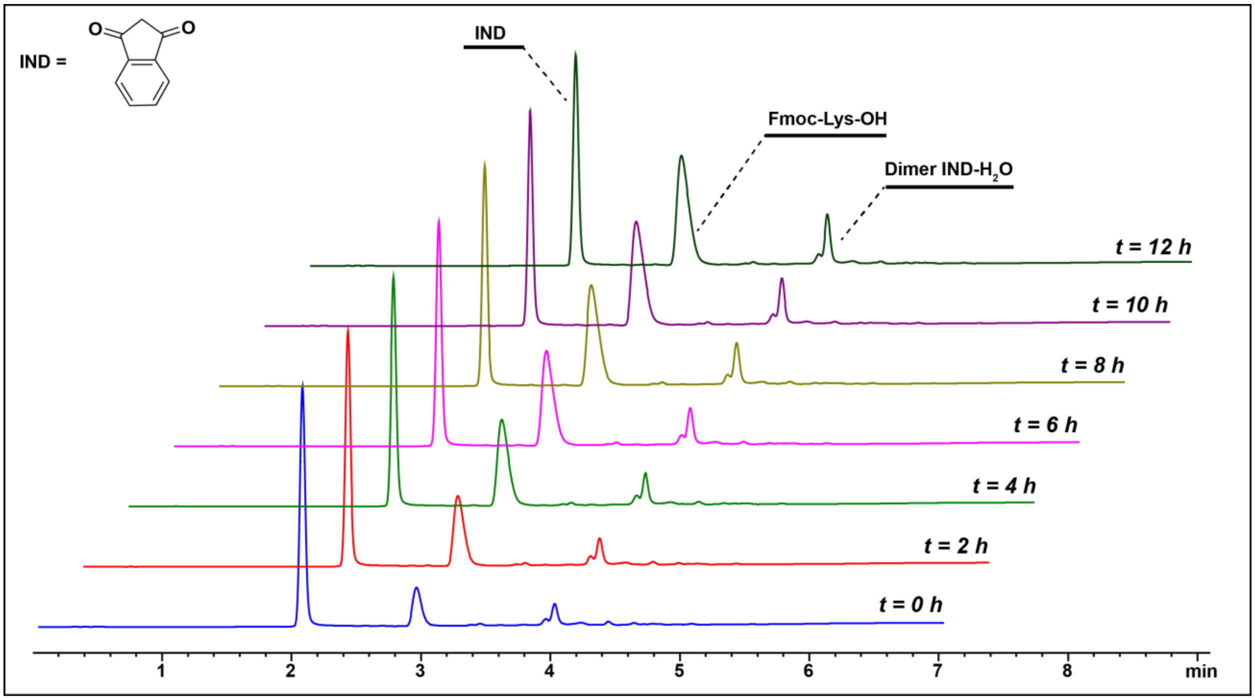

Figure S21. Reaction of Indanedione (IND, 1 mM) with Fmoc-Lys-OH (1 mM) in PBS (25 mM, pH = 7.4, rt) over 12 h.

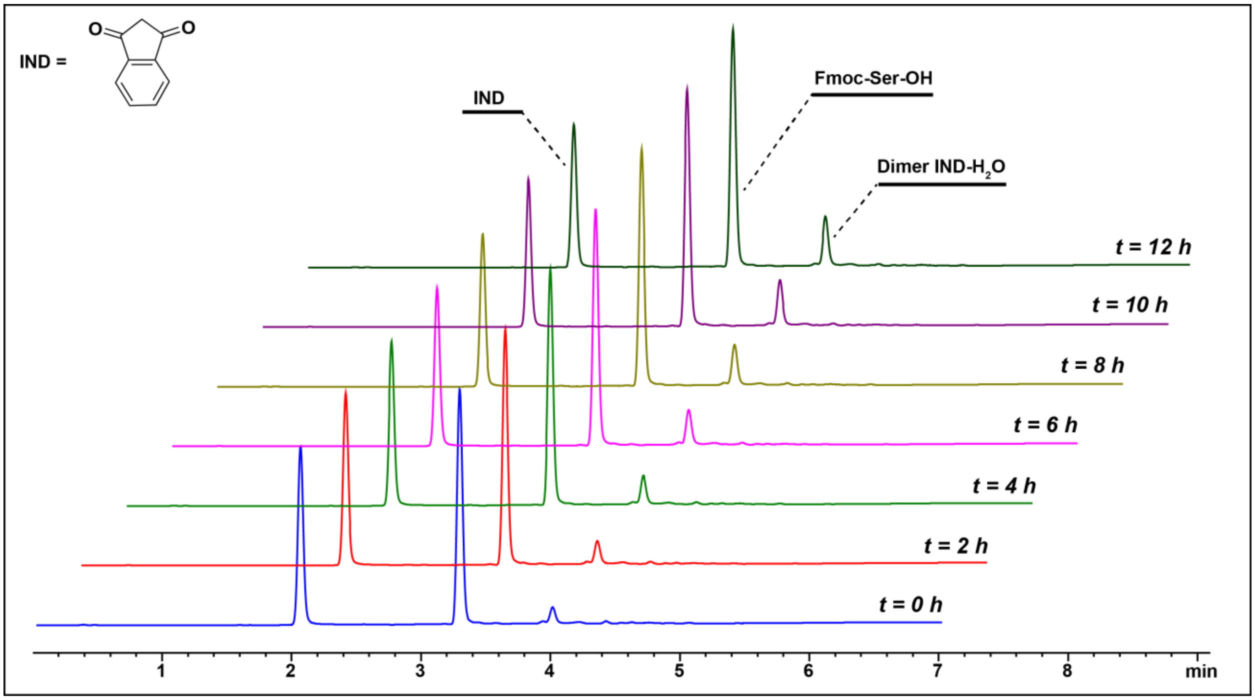

Figure S22. Reaction of Indanedione (IND, 1 mM) with Fmoc-Ser-OH (1 mM) in PBS (25 mM, pH = 7.4, rt) over 12 h.

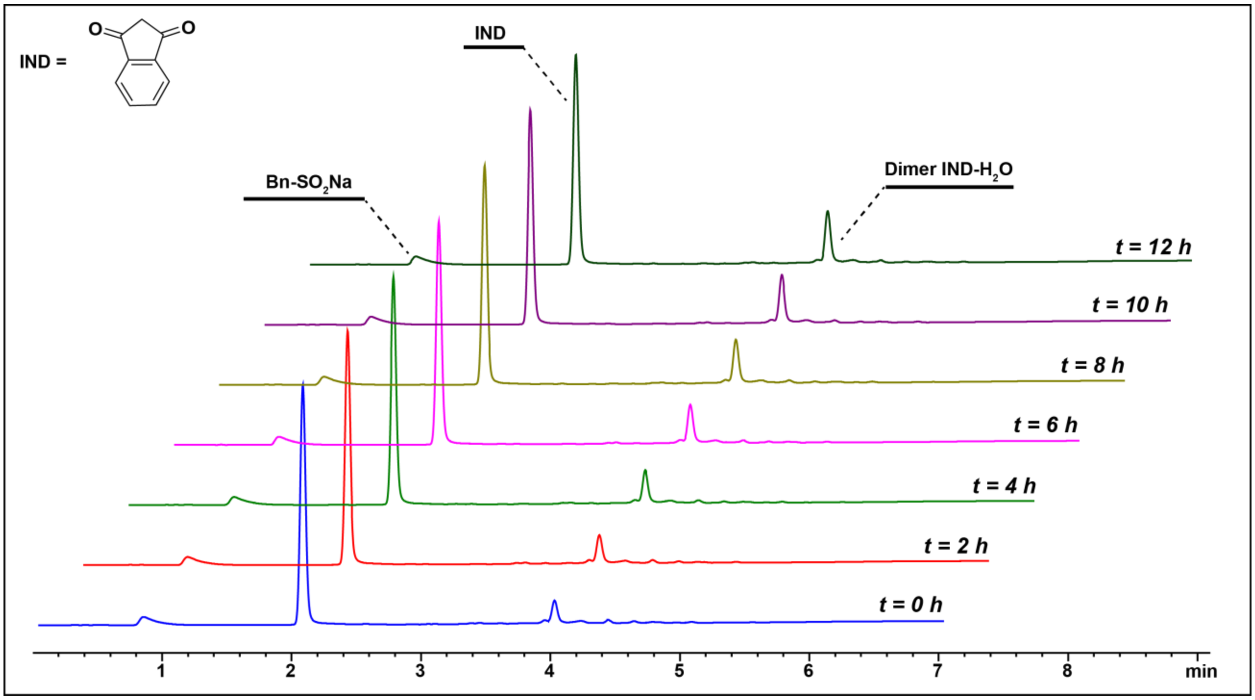

Figure S23. Reaction of Indanedione (IND, 1 mM) with benzyl sulfinate sodium salt (1 mM) in PBS (25 mM, pH = 7.4, rt) over 12 h.

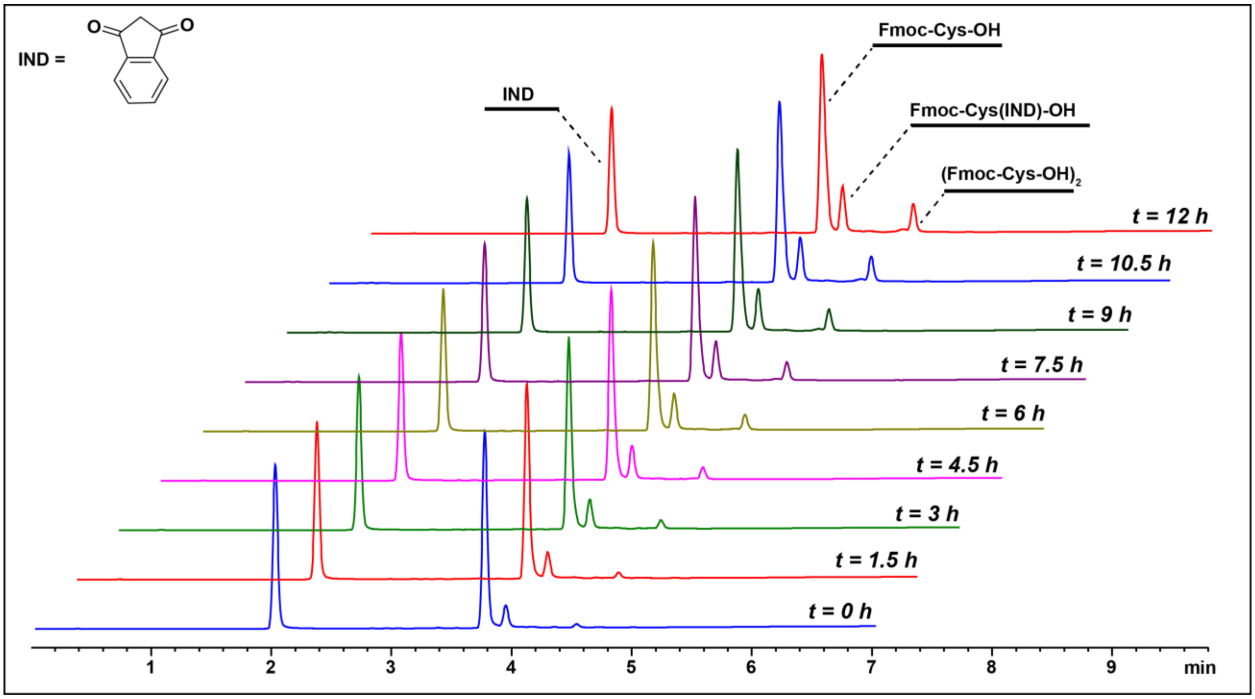

Figure S24. Reaction of IND (1 mM) with Fmoc-Cys-OH (1 mM) in PBS (25 mM, pH = 7.4, rt) over a course of 12 h.

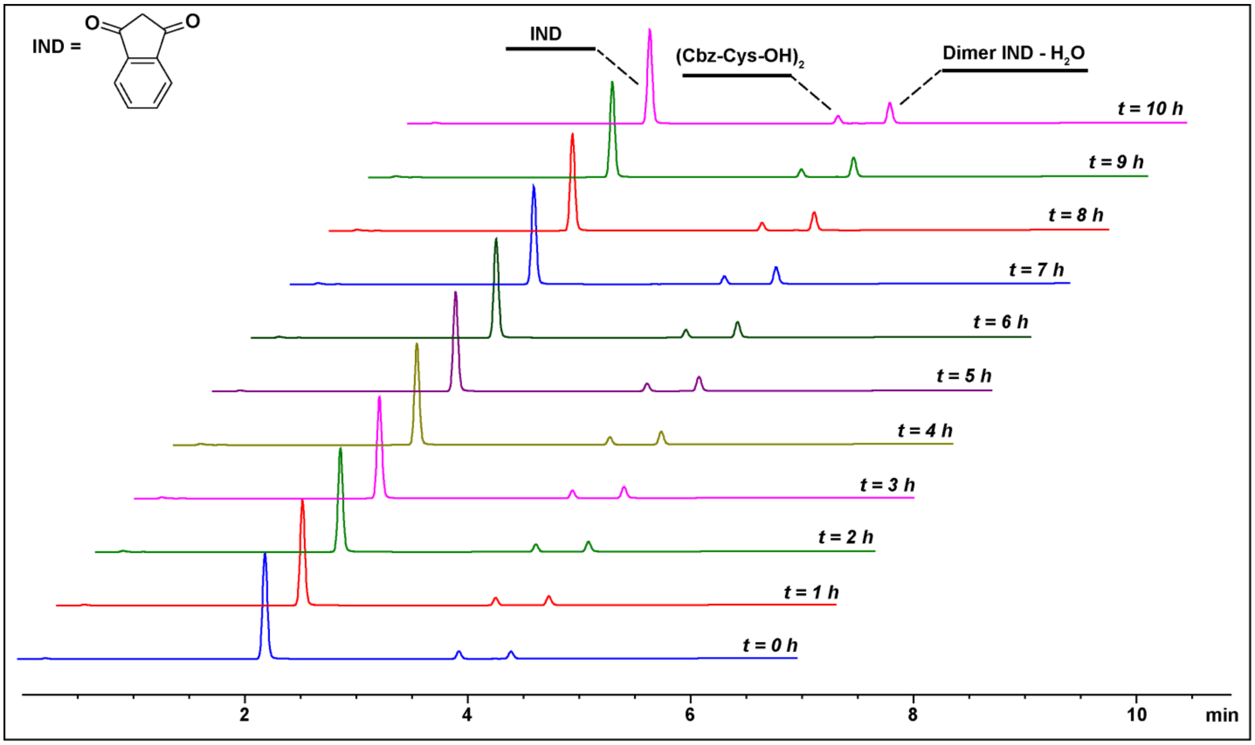

Figure S25. Reaction of IND (1 mM) with (Cbz-Cys-OH) (1 mM) in PBS (25 mM, pH = 7.4, rt) over a course of 10 h.

Scheme S20. Synthesis of dipeptide sulfenic acid adduct with various nucleophiles

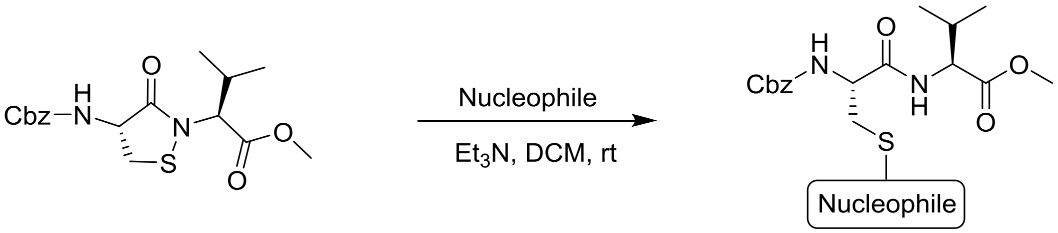

General Procedure: To a solution of dipeptide cyclic sulfenamide (0.11 g, 0.3 mmol) and nucleophile (0.33 mmol, 1.1 eq) in anhydrous DCM, was added Et_3N (0.5 ml). Resulting reaction mixture was stirred at room temperature with monitoring. After completion, the reaction mixture was evaporated under reduced pressure. Crude thus obtained was purified directly by prep-HPLC.

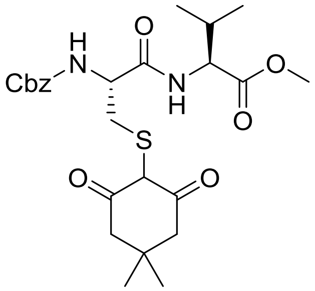

^{1}H-NMR (400 MHz, DMSO-*d6*) *δ* 8.22 (d, 1H, J = 8.0 Hz), 7.53 (d, 1H, J = 8.0 Hz), 7.25 – 7.40 (m, 5H), 5.02 (s, 2H), 4.17 (t, 1H, J = 8.0 Hz), 4.07 (q, 1H, J = 8.0 Hz), 3.62 (s, 3H), 2.65 – 2.75 (m, 2H), 2.34 (s, 4H), 2.05 (hep, 1H, J = 8.0 Hz), 0.97 (s, 6H), 0.88 (d, 3H, J = 6.0 Hz), 0.87 (d, 3H, J = 6.0 Hz); ^{13}C-NMR (100 MHz, DMSO-*d6*) δ 171.7, 170.8, 155.7, 137.0, 128.4, 127.9, 127.8, 104.8, 65.5, 57.6, 54.4, 51.8, 36.0, 31.2, 30.0, 27.8, 19.0, 18.1

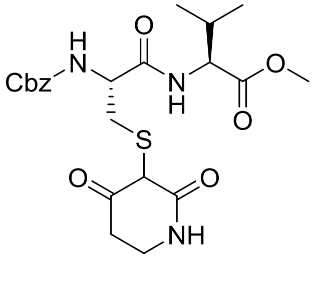

^{1}H-NMR (400 MHz, DMSO-*d6*) *δ* 8.52 (d, 1H, J = 8.0 Hz), 7.75 (d, 1H, J = 4.0 Hz), 7.25 – 7.45 (m, 5H), 5.02 (s, 2H), 4.05 – 4.25 (m, 2H), 3.62 (s, 3H), 3.10 – 3.25 (m, 2H), 2.60 – 2.80 (m, 2H), 2.45 – 2.60 (m, 2H), 2.06 (hep, 1H, J = 8.0 Hz), 0.97 (s, 6H), 0.89 (d, 3H, J = 6.0 Hz), 0.87 (d, 3H, J = 6.0 Hz); ^{13}C-NMR (100 MHz, DMSO-*d6*) δ 171.7, 170.8, 168.0, 155.7, 137.0, 128.4, 127.9, 127.8, 65.5, 57.7, 54.1, 51.7, 36.7, 30.0, 19.0, 18.1

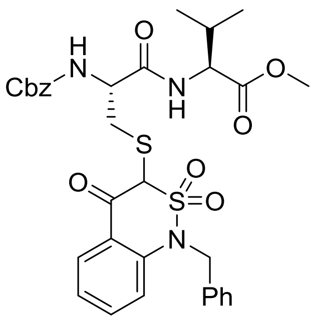

^{1}H-NMR (400 MHz, DMSO-*d6*) *δ* 8.32 (d, 1H, J = 8.0 Hz), 7.92 (d, 1H, J = 8.0 Hz), 7.77 (d, 1H, J = 8.0 Hz), 7.51 (t, 1H, J = 8.0 Hz), 7.10 – 7.45 (m, 12H), 5.23 (s, 2H), 5.07 (s, 2H), 4.39 (q, 1H, J = 8.0 Hz), 4.23 (t, 1H, J = 8.0 Hz), 3.62 (s, 3H), 3.12 – 3.25 (m, 1H), 2.97 – 3.12 (m, 1H), 2.07 (hep, 1H, J = 8.0 Hz), 0.89 (d, 3H, J = 6.0 Hz), 0.88 (d, 3H, J = 6.0 Hz); ^{13}C-NMR (100 MHz, DMSO-*d6*) δ 171.7, 170.6, 156.0, 138.5, 136.8, 136.3, 132.4, 128.6, 128.4, 127.9, 127.8, 127.5, 127.1, 126.3, 123.1, 118.4, 65.8, 57.6, 54.0, 51.8, 48.8, 38.4, 30.0, 18.9, 18.1

Scheme S21. Stability of Dipeptide sulfenic acid – Nucleophile adducts under reductive conditions.

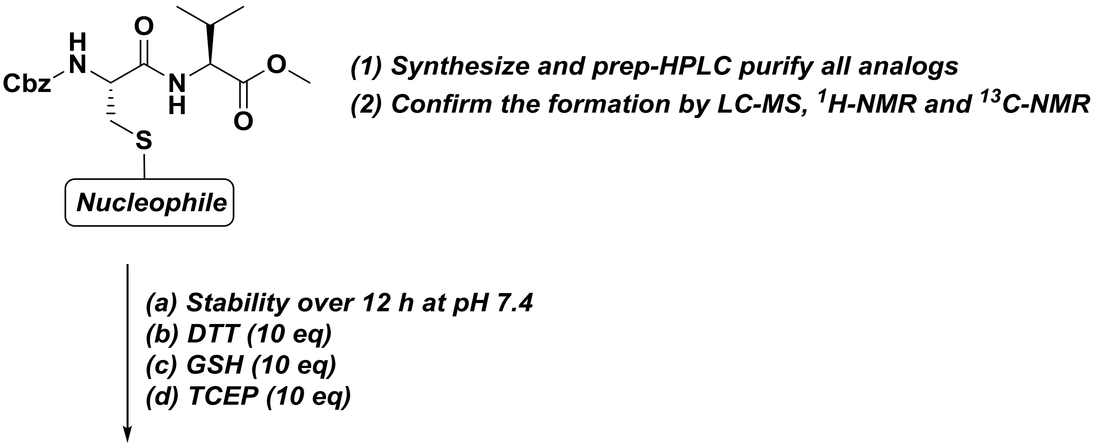

Procedure: A 25 mM solution of dipeptide-S-Nu adduct was prepared in DMSO. Solutions of DTT (250 mM), GSH (250 mM) and TCEP (250 mM) were prepared in PBS buffer. 10 μL of dipepride-S-Nu solution and 10 μL of each reducing agent solution was added to 980 μL of PBS buffer (25 mM, pH = 7.4, rt). Effective concentrations were as follow:

(1) Dipeptide-S-Nu = 250 μM

(2) Reducting agent = 2.5 mM

(3) Buffer = 25 mM

Each reaction mixture was analyzed by LC-MS at 3 h intervals.

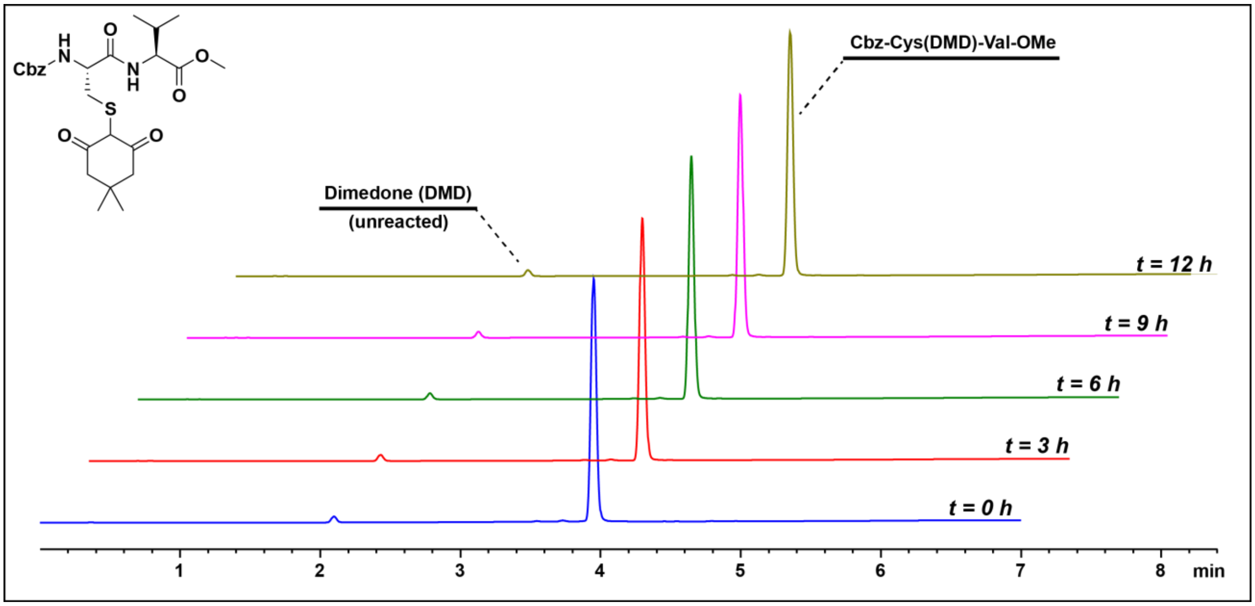

Figure S21. Stability of Cbz-Cys(DMD)-Val-OMe (250 μM) in PBS buffer (25 mM, pH = 7.4, rt) over a course of 12 h.

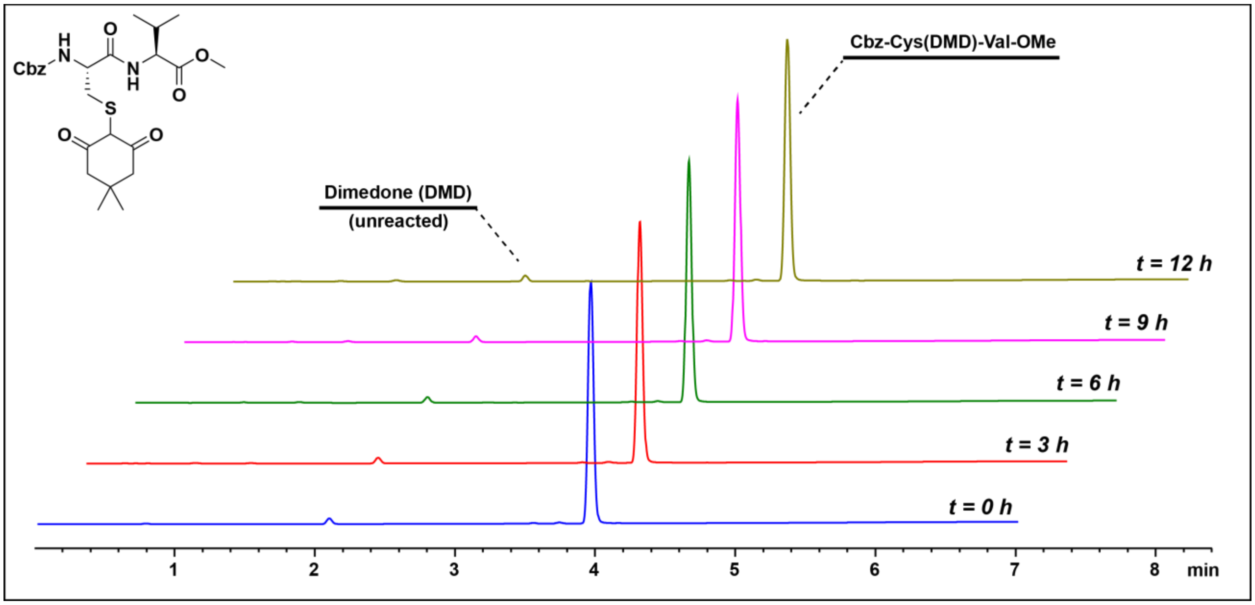

Figure S22. Stability of Cbz-Cys(DMD)-Val-OMe (250 μM) in presence of DTT (2.5 mM) in PBS buffer (25 mM, pH = 7.4, rt) over a course of 12 h.

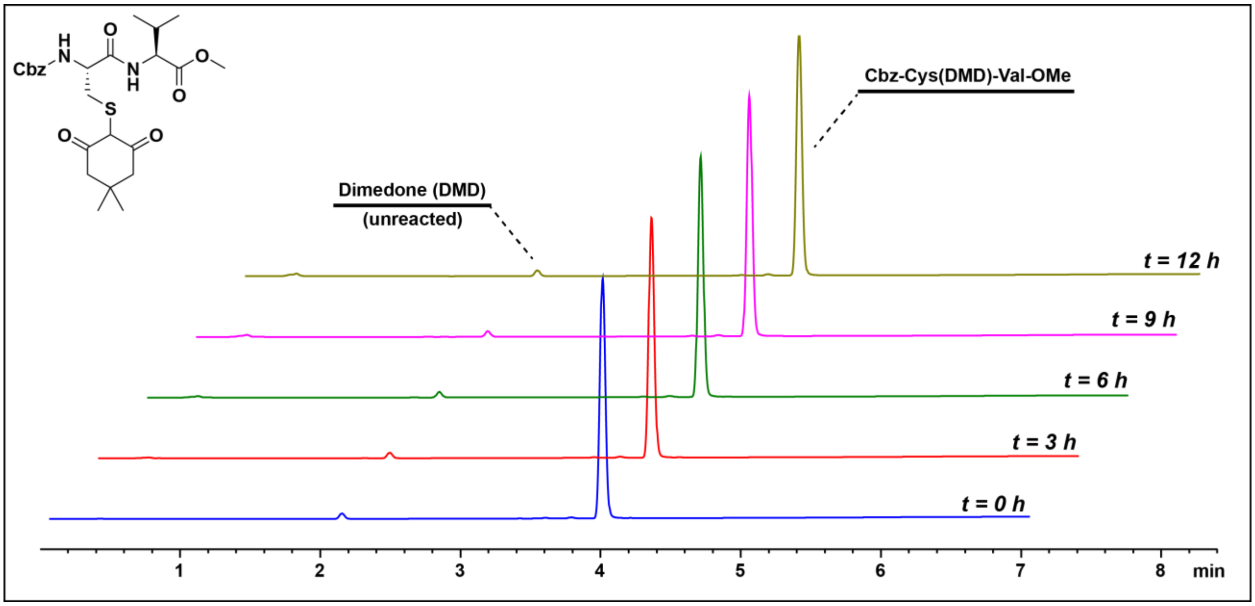

Figure S23. Stability of Cbz-Cys(DMD)-Val-OMe (250 μM) in presence of GSH (2.5 mM) in PBS buffer (25 mM, pH = 7.4, rt) over a course of 12 h.

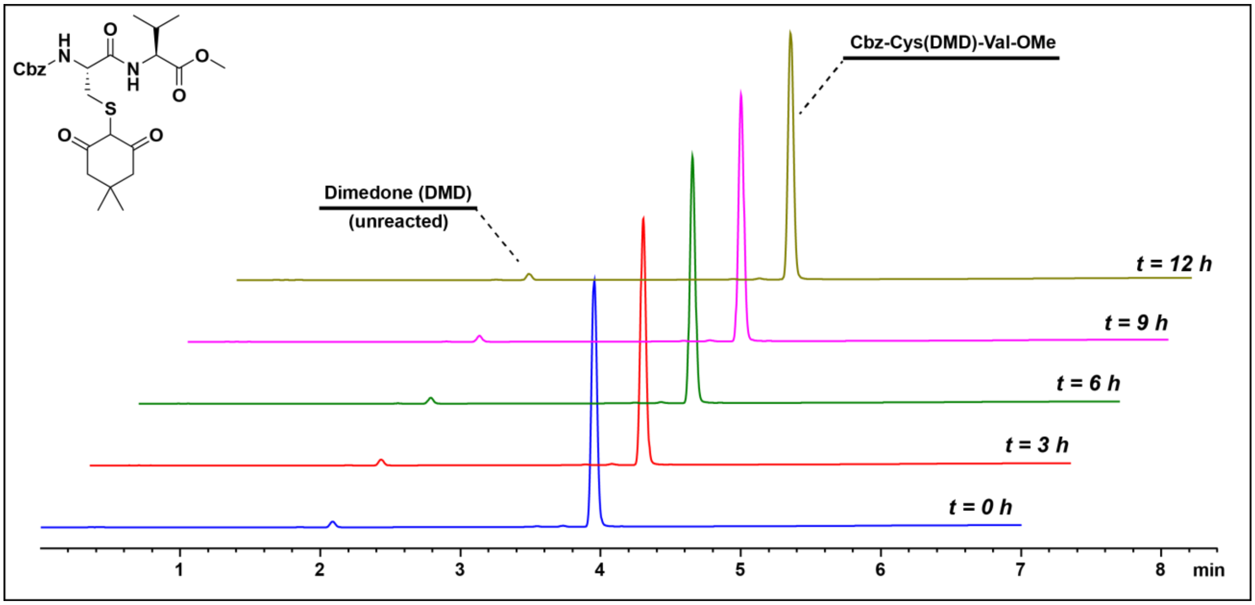

Figure S24. Stability of Cbz-Cys(DMD)-Val-OMe (250 μM) in presence of TCEP (2.5 mM) in PBS buffer (25 mM, pH = 7.4, rt) over a course of 12 h.

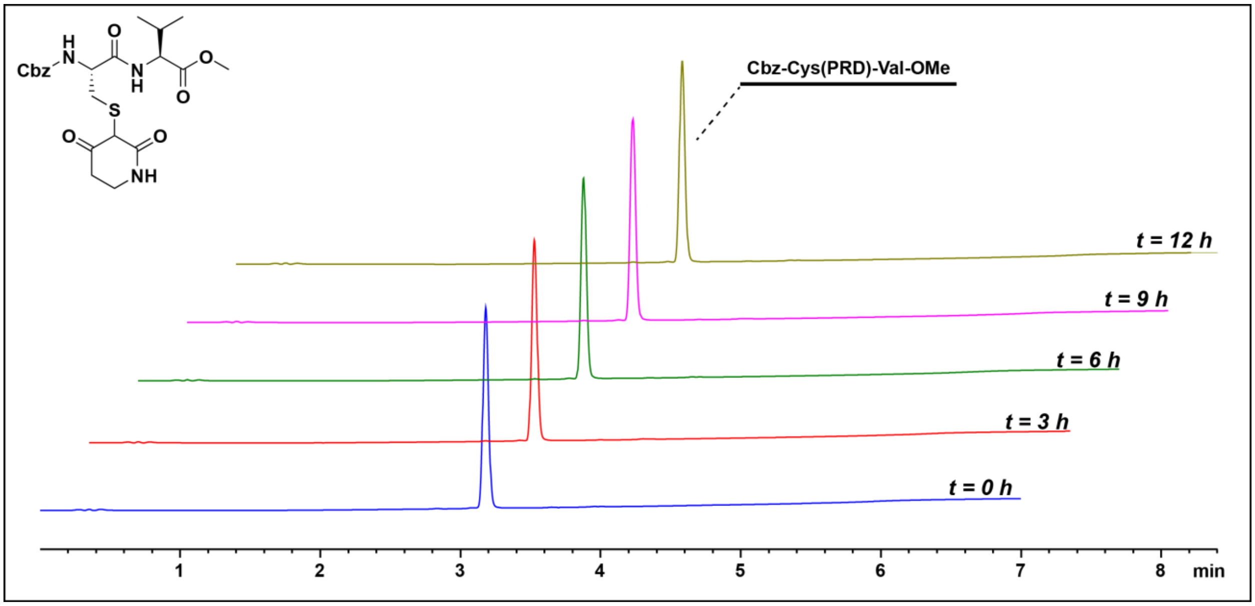

Figure S25. Stability of Cbz-Cys(PRD)-Val-OMe (250 μM) in PBS buffer (25 mM, pH = 7.4, rt) over a course of 12 h.

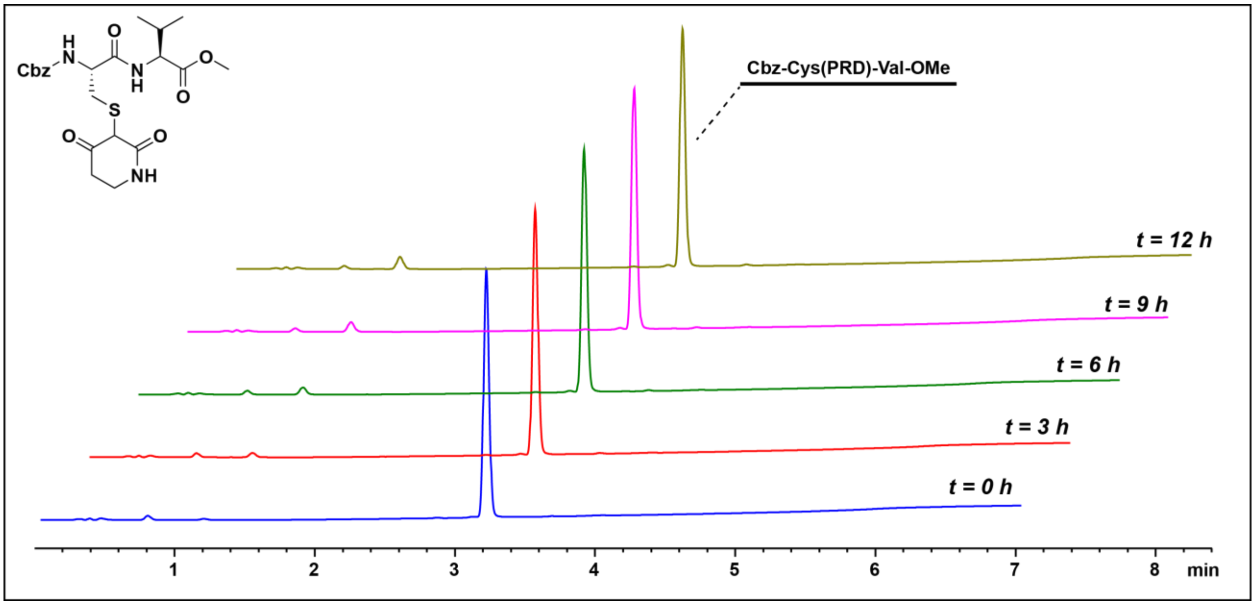

Figure S26. Stability of Cbz-Cys(PRD)-Val-OMe (250 μM) in presence of DTT (2.5 mM) in PBS buffer (25 mM, pH = 7.4, rt) over a course of 12 h.

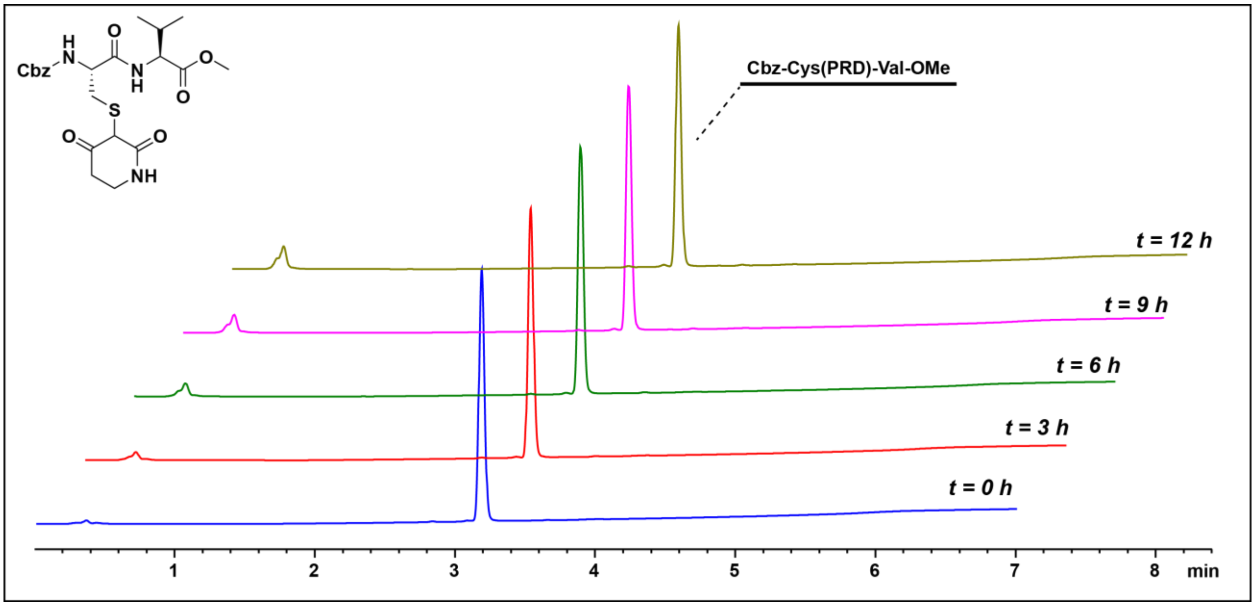

Figure S27. Stability of Cbz-Cys(PRD)-Val-OMe (250 μM) in presence of GSH (2.5 mM) in PBS buffer (25 mM, pH = 7.4, rt) over a course of 12 h.

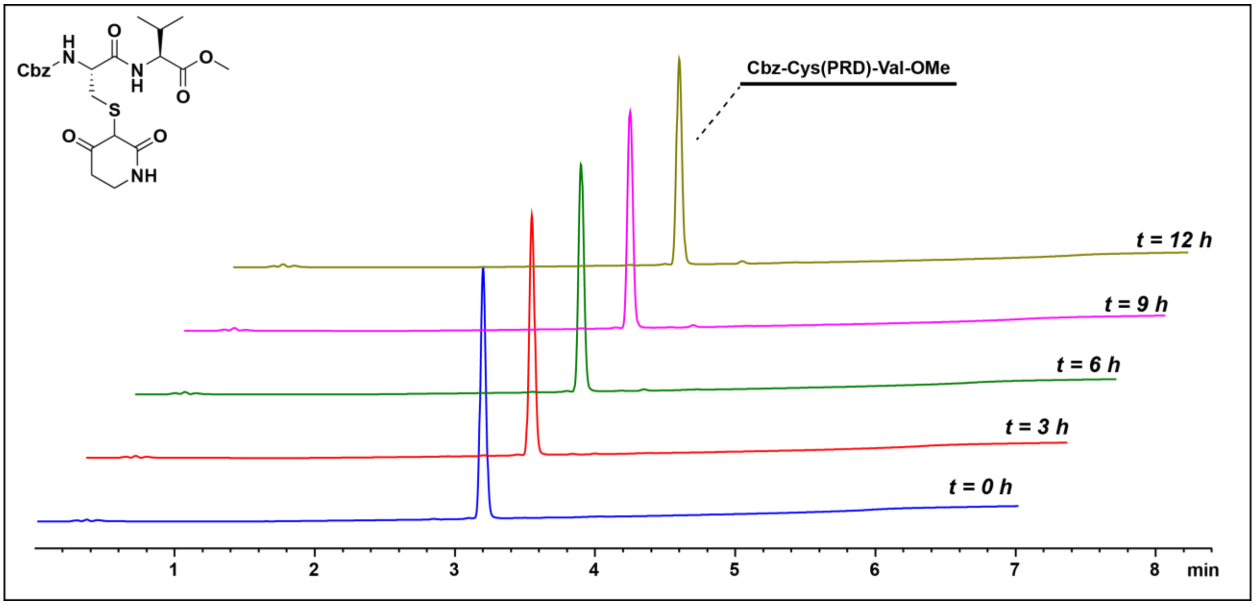

Figure S28. Stability of Cbz-Cys(PRD)-Val-OMe (250 μM) in presence of TCEP (2.5 mM) in PBS buffer (25 mM, pH = 7.4, rt) over a course of 12 h.

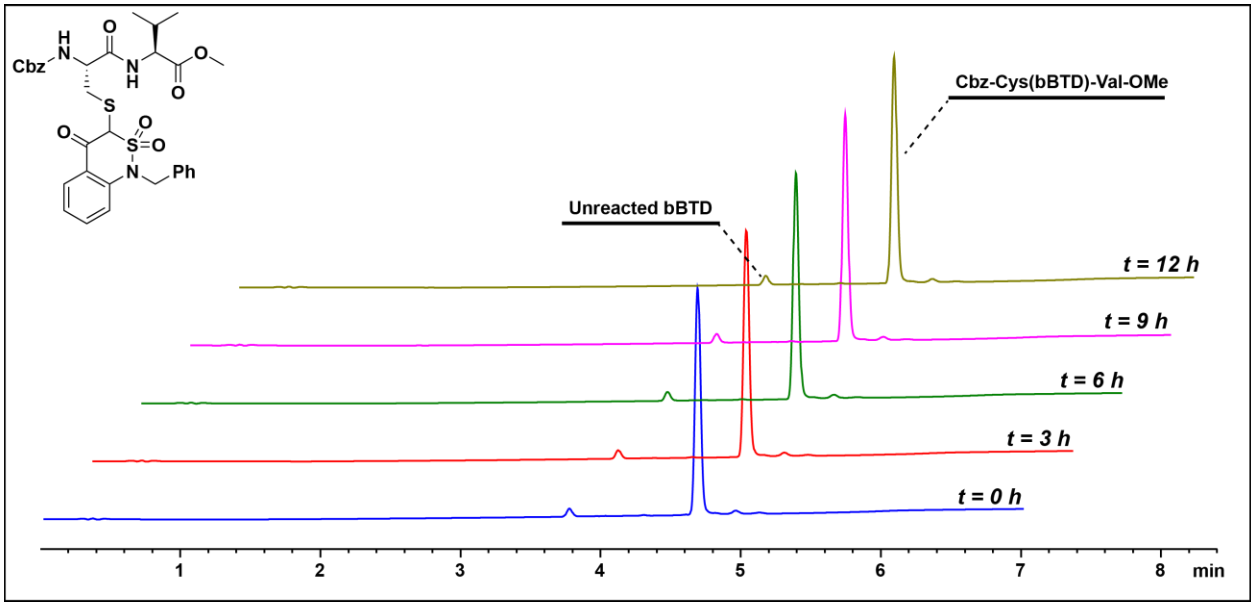

Figure S29. Stability of Cbz-Cys(bBTD)-Val-OMe (250 μM) in PBS buffer (25 mM, pH = 7.4, rt) over a course of 12 h.

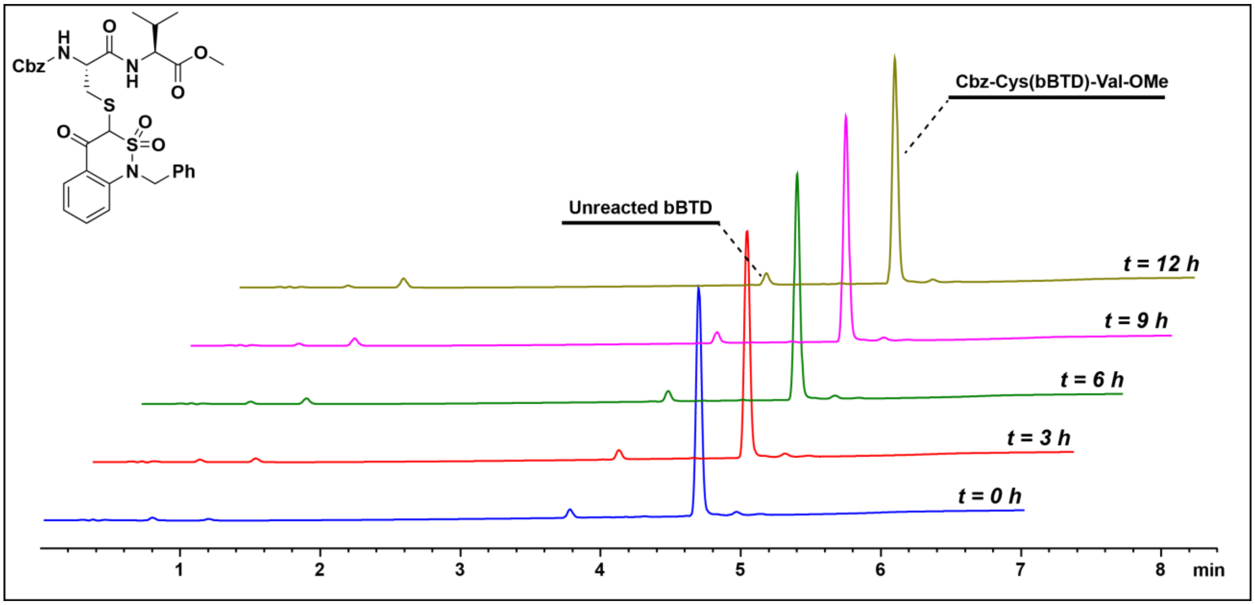

Figure S30. Stability of Cbz-Cys(bBTD)-Val-OMe (250 μM) in presence of DTT (2.5 mM) in PBS buffer (25 mM, pH = 7.4, rt) over a course of 12 h.

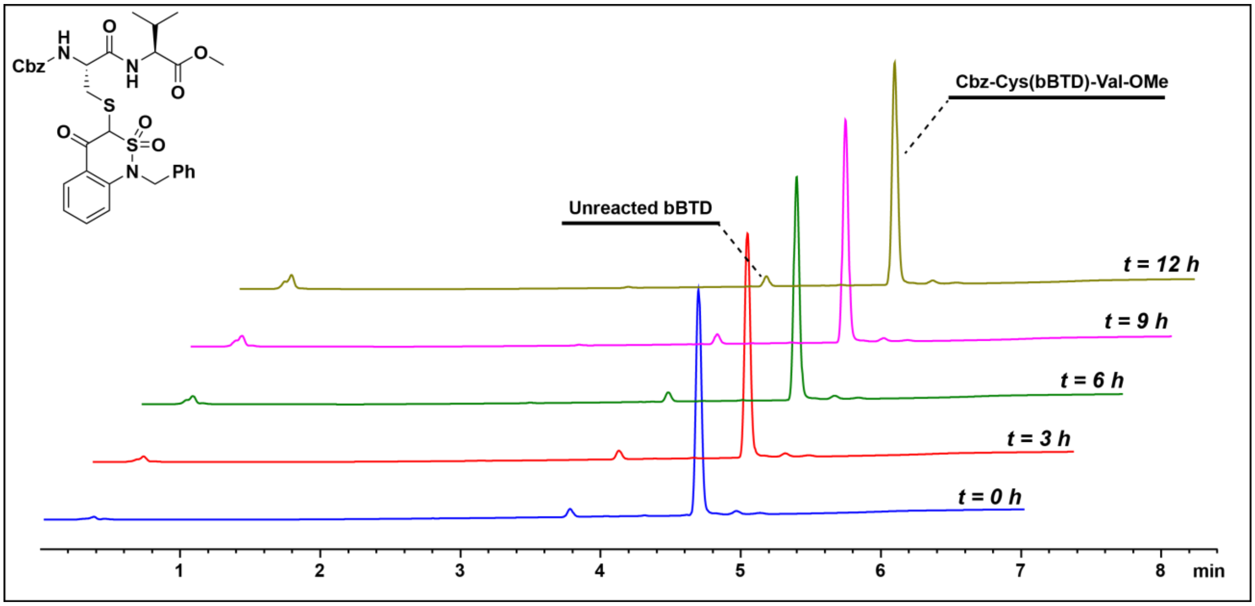

Figure S31. Stability of Cbz-Cys(bBTD)-Val-OMe (250 μM) in presence of GSH (2.5 mM) in PBS buffer (25 mM, pH = 7.4, rt) over a course of 12 h.

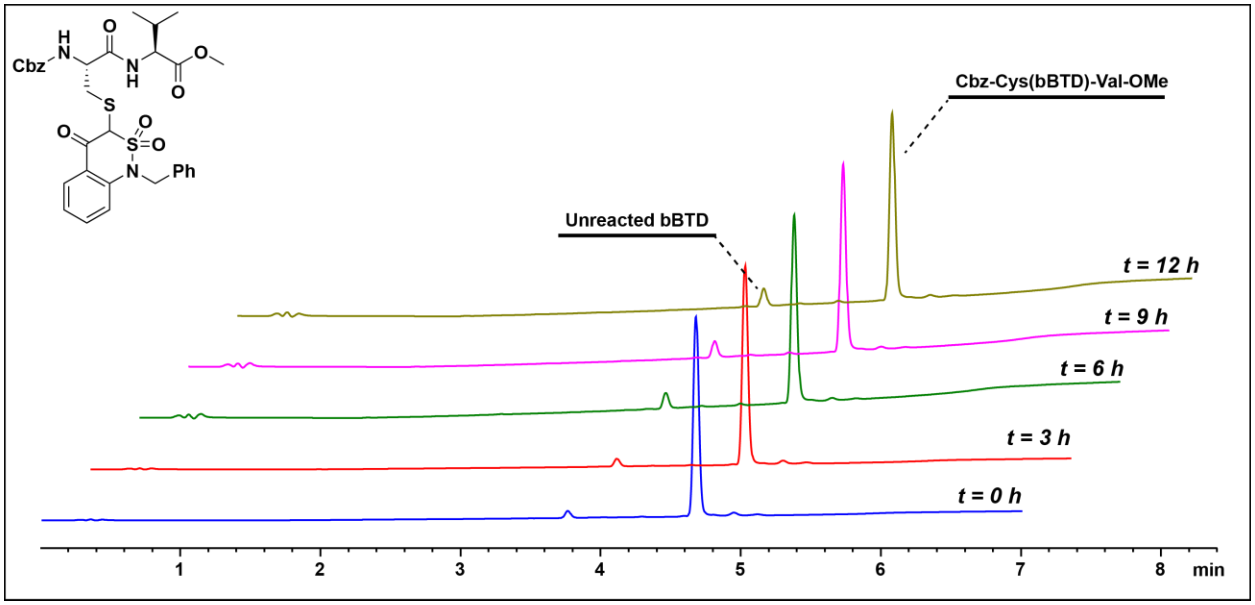

Figure S32. Stability of Cbz-Cys(bBTD)-Val-OMe (250 μM) in presence of TCEP (2.5 mM) in PBS buffer (25 mM, pH = 7.4, rt) over a course of 12 h.

Scheme S22. Labeling of recombinant C64,82S Gpx3-SOH with various nucleophiles

Reaction:

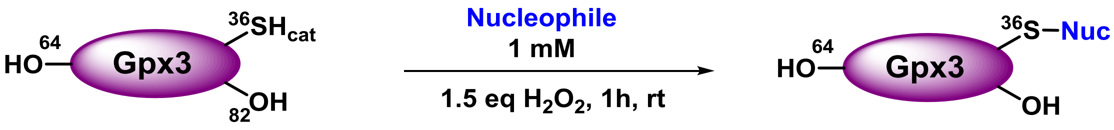

***C64,82S* Gpx3 (10 μM)**

Nucleophiles/Electrophiles tested:

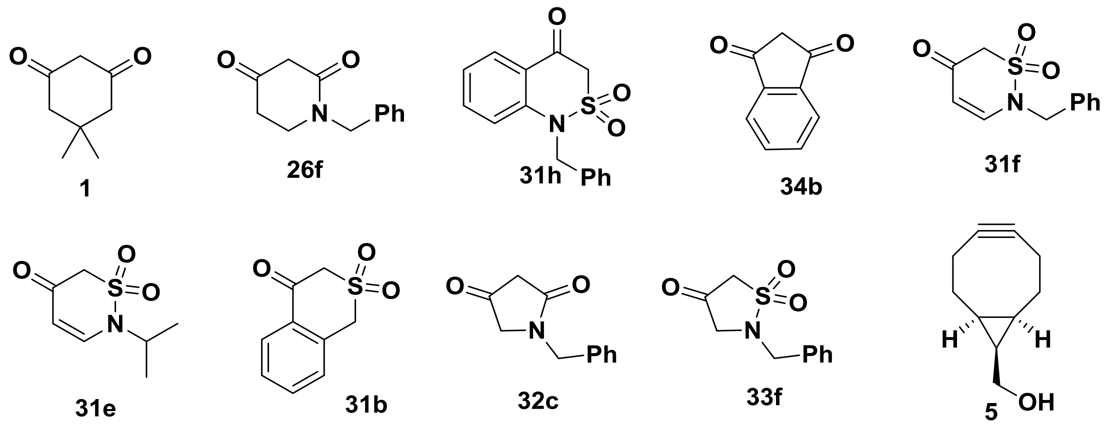

(1) 100 mM stock solutions of nucleophiles were prepared in DMSO – If not soluble at room temperature, the eppendorf tubes were heated in 100 ^{o}C water bath for 2 minutes. 100 mM stock solutions were then diluted to 20 mM and 2 mM solution in 70% Gpx3 labeling buffer (50 mM HEPES, 100 mM NaCl, pH = 7.4), 30% DMSO.

(2) C64, 82S Gpx3 was thawed at 0 ^{o}C and reduced with 50 mM (2M, 5 μL) DTT for 20 minutes on ice.

A 2 M solution of DTT in water was prepared (31 mg of DTT in 100 μl of water). 5 μl of this DTT solution was added to the protein solution (200 μl) giving an effective DTT concentration of 50 mM.

(3) C64, 82S Gpx3-SH was buffer exchanged to labeling buffer (50 mM HEPES, 100 mM NaCl, pH = 7.4) using pre-equilibrated Nap-5 column. Nap-5 column was equilibrated by passing 10 ml of Gpx3 labeling buffer.

(4) Determine the concentration of C64, 82S Gpx3 by using A_{280} (ε = 24410 $M^{-1}cm^{-1}$).

(5) Label 10 μM of C64, 82S Gpx3 with 1.5 eq of H_2O_2 and 1 mM or 100 μM probe keeping DMSO $\leq$ 5%. Incubate for 1 h [REACTION VOLUME = 100 μl].

For each probe, three reactions were performed:

(i) Protein incubated with TCEP (10 mM) + Nucleophile/electrophile (1 mM)

(ii) Protein + Nucleophile/electrophile (100 μM) + H_2O_2 (1.5 eq)

(iii) Protein + Nucleophile/electrophile (1 mM) + H_2O_2 (1.5 eq)

Order of addition was as follow:

(a) Appropriate amounts of Gpx3 labeling buffer were added to the eppy tubes.

(b) C64, 82S Gpx3 was added to the tube and the contents were mixed gently.

(c) TCEP (10 mM) was added to the appropriate tubes.

(d) Subsequently, probe solution was added to each of the tubes. For the probe, 5 μL of a 2 mM or 20 mM solution was used. This gave 100 μM or 1 mM effective probe concentration for each reaction and kept the DMSO concentration at < 5%.

(e) Hydrogen peroxide solution was added last. A 1 mM solution of hydrogen peroxide was prepared in water by serial dilution starting with commercially available 8.8 M solution. 1.5 μl of this 1 mM peroxide solution was used in each case to give an effective concentration of 15 μM.

(f) Reaction mixtures were incubated for 1 h at rt.

(6) The reaction mixtures were quenched by the filtration through pre-equilibrated P30 columns.

(7) During the filtration through P30 columns, each sample was buffer exchanged to 25 mM ammonium bicarbonate (pH 8.0).

(8) Each sample was then analyzed by LTQ-MS by injecting 3 μM concentration in 0.1% formic acid in water.

Controls

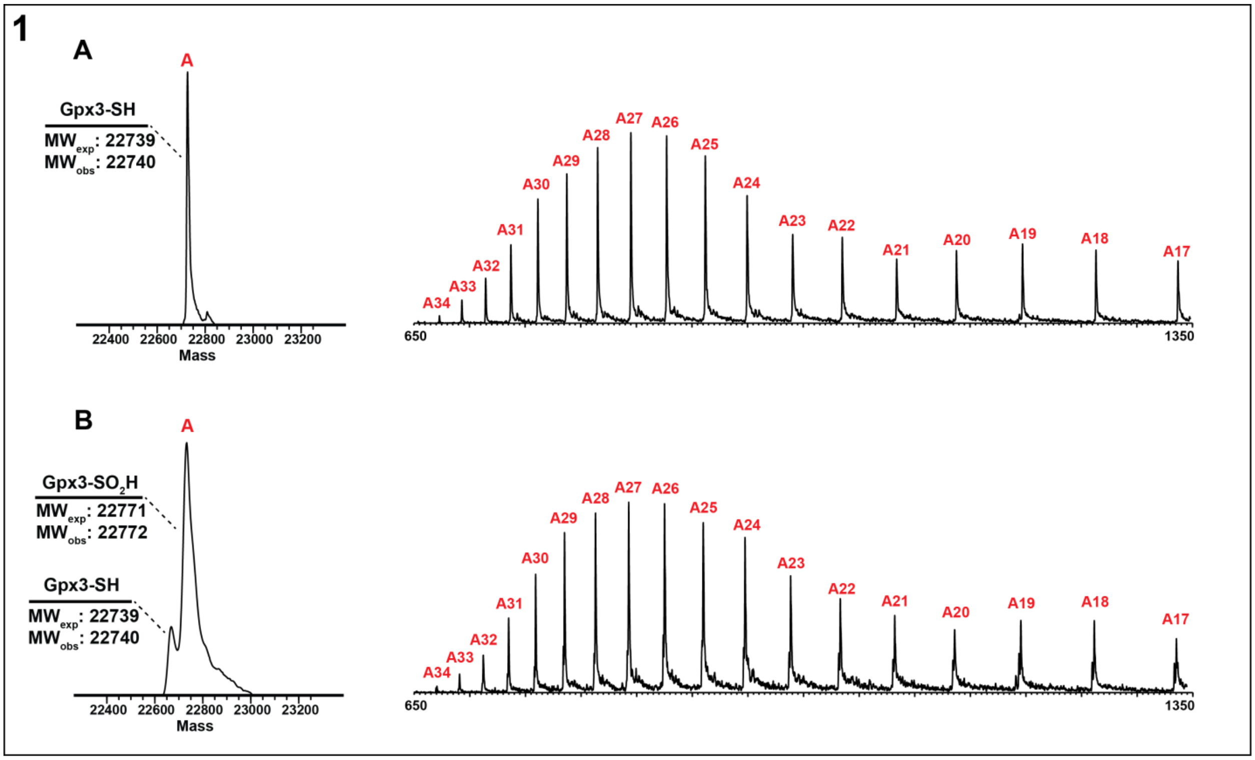

Fig. S33 Controls – (A) C64,82S Gpx3 (reduced form); **(B)** C64,82S Gpx3 (oxidized form)

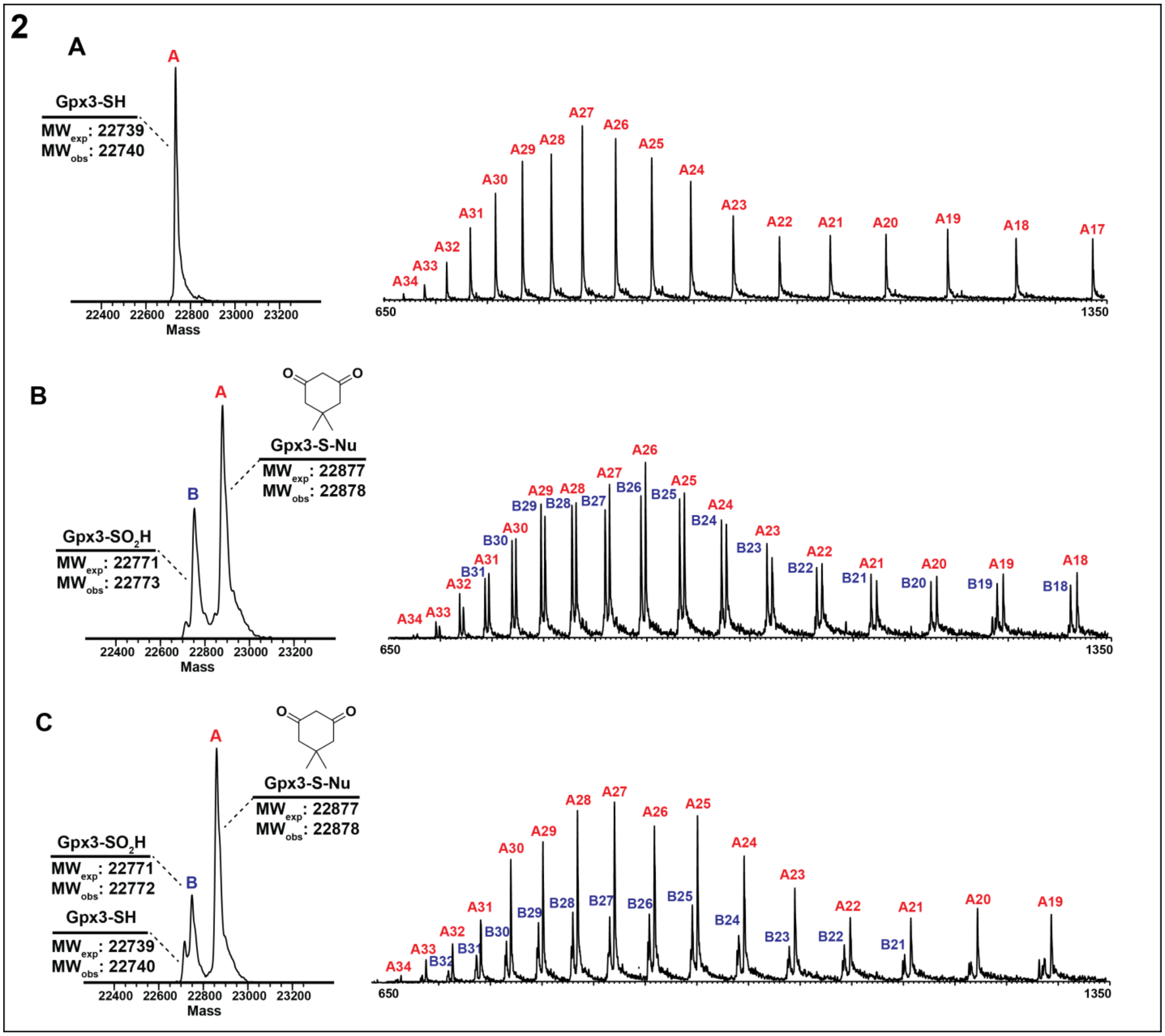

Fig. S34 Reaction of C64, 82S Gpx3 with dimedone (1) – (A) Gpx3 (10 μM) + TCEP (10 mM) + **1** (1 mM); **(B)** Gpx3 (10 μM) + **1** (100 μM) + H_2O_2 (1.5 eq); **(C)** Gpx3 (10 μM) + **1** (1 mM) + H_2O_2 (1.5 eq)

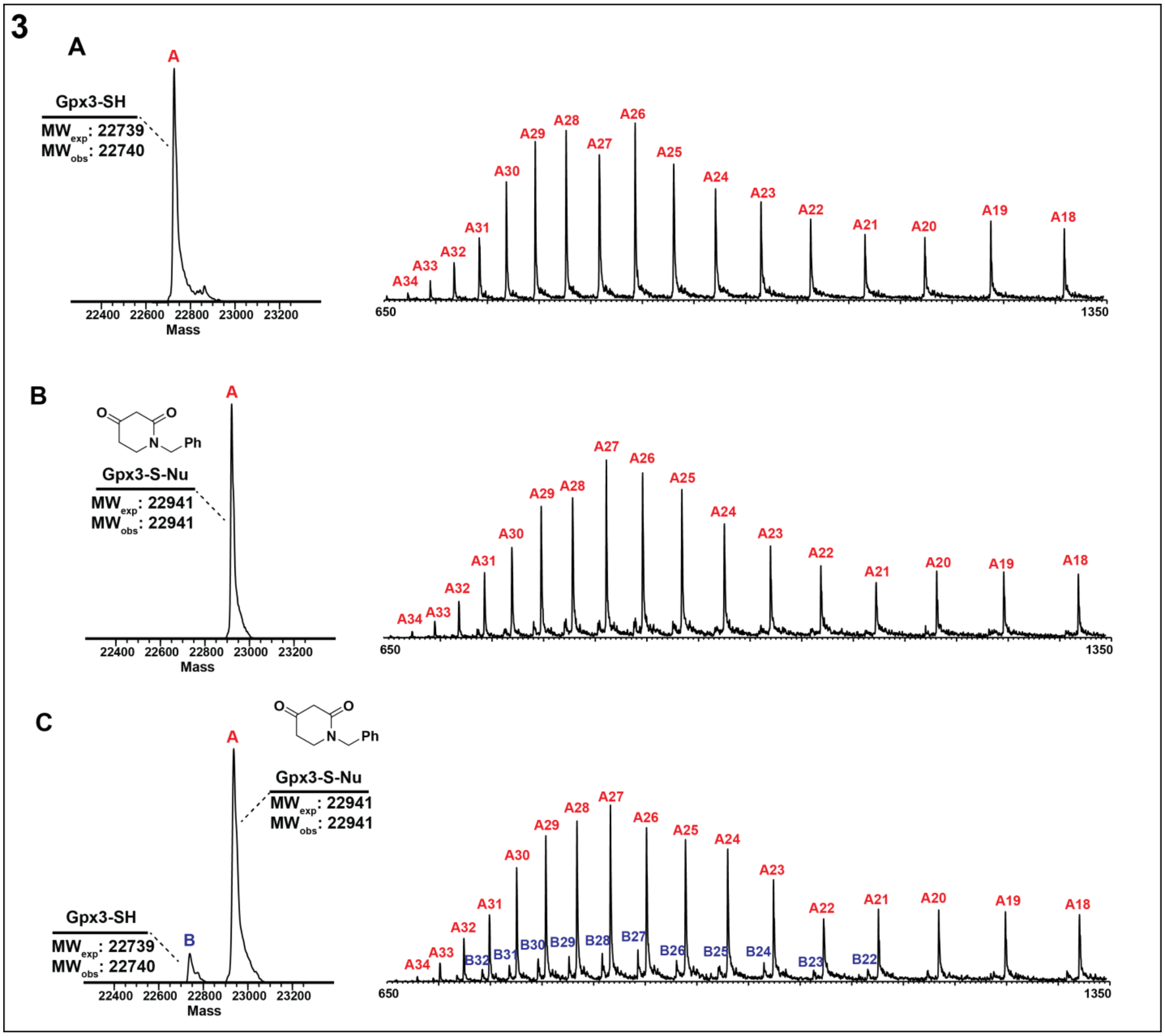

Fig. S35 Reaction of C64, 82S Gpx3 with 1-benzylpiperidine-2,4-dione (26f) – (A) Gpx3 (10 μM) + TCEP (10 mM) + **26f** (1 mM); **(B)** Gpx3 (10 μM) + **26f** (100 μM) + H_2O_2 (1.5 eq); **(C)** Gpx3 (10 μM) + **26f** (1 mM) + H_2O_2 (1.5 eq)

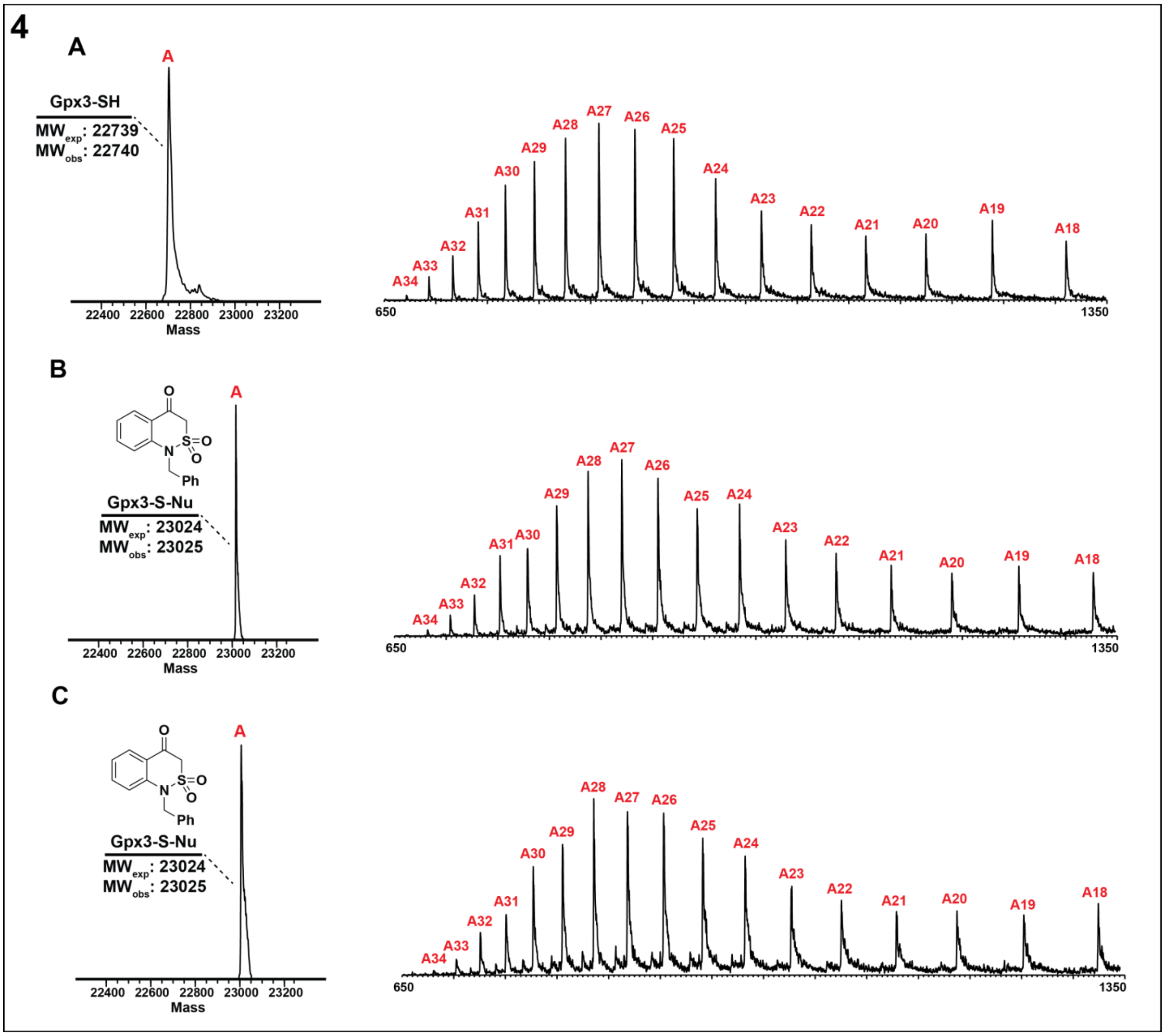

Fig. S36 Reaction of C64, 82S Gpx3 with 1-benzyl-1*H*-benzo[*c*][1,2]thiazin-4(3*H*)-one 2,2-dioxide (31h) – (A) Gpx3 (10 μM) + TCEP (10 mM) + **31h** (1 mM); **(B)** Gpx3 (10 μM) + **31h** (100 μM) + H_2O_2 (1.5 eq); **(C)** Gpx3 (10 μM) + **31h** (1 mM) + H_2O_2 (1.5 eq)

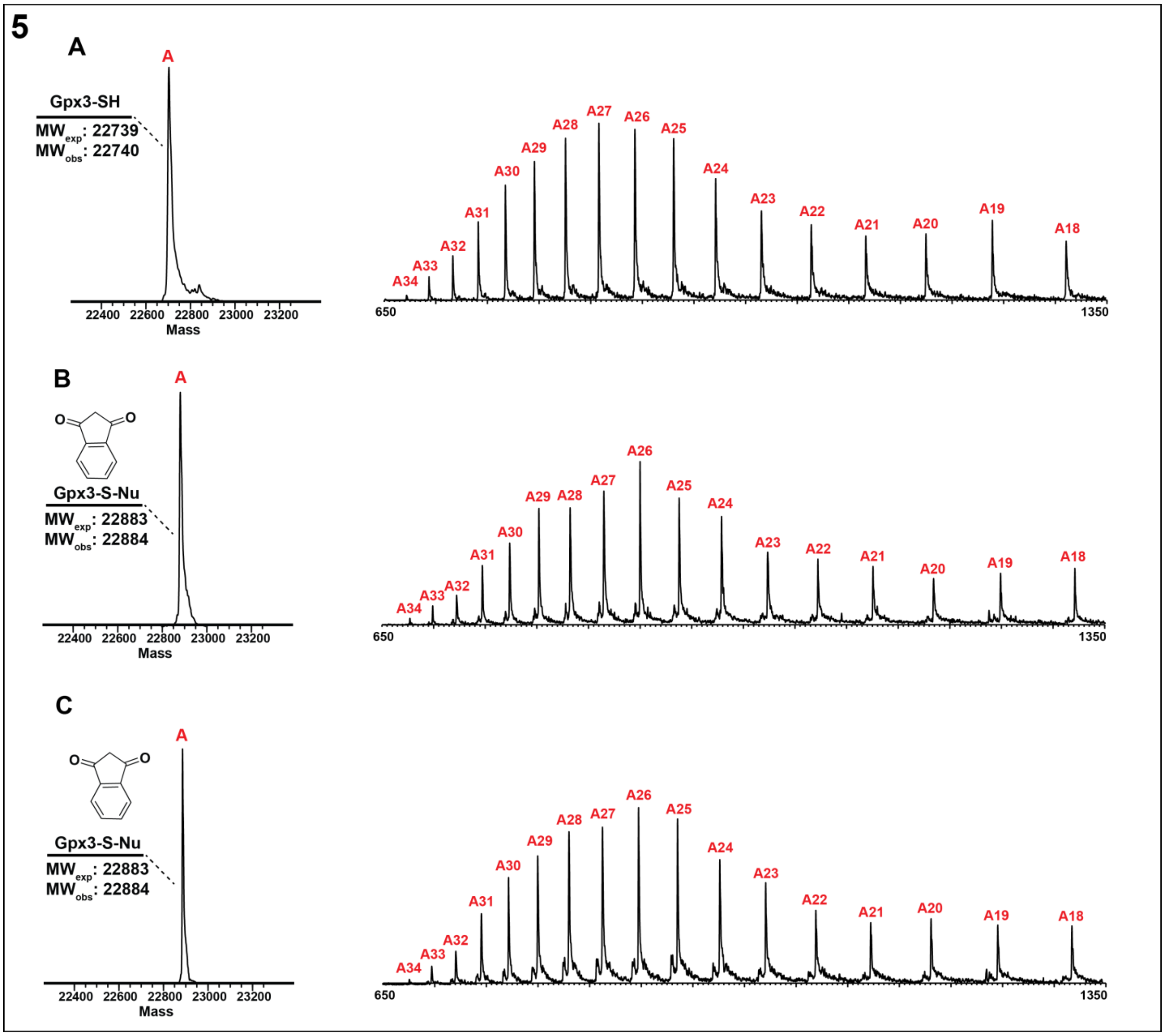

Fig. S37 Reaction of C64, 82S Gpx3 with 1,3-indandione (34b) – (A) Gpx3 (10 μM) + TCEP (10 mM) + **34b** (1 mM); **(B)** Gpx3 (10 μM) + **34b** (100 μM) + H_2O_2 (1.5 eq); **(C)** Gpx3 (10 μM) + **34b** (1 mM) + H_2O_2 (1.5 eq)

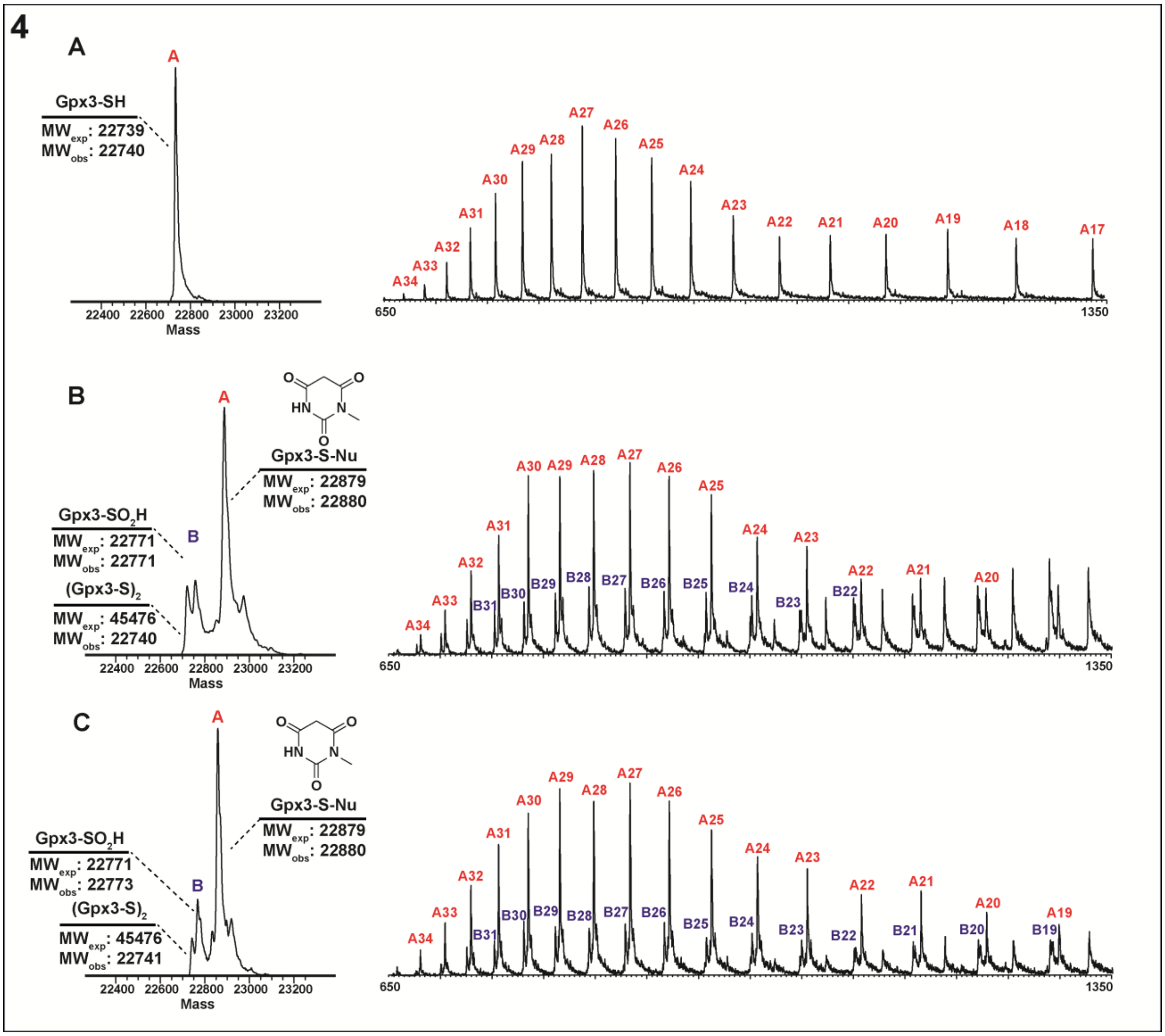

Fig. S38 Reaction of C64, 82S Gpx3 with N-methylbarbituric acid (27b) – (A) Gpx3 (10 μM) + TCEP (10 mM) + **27b** (1 mM); **(B)** Gpx3 (10 μM) + **27b** (1 mM) + H_2O_2 (1.5 eq); **(C)** Gpx3 (10 μM) + **27b** (5 mM) + H_2O_2 (1.5 eq)

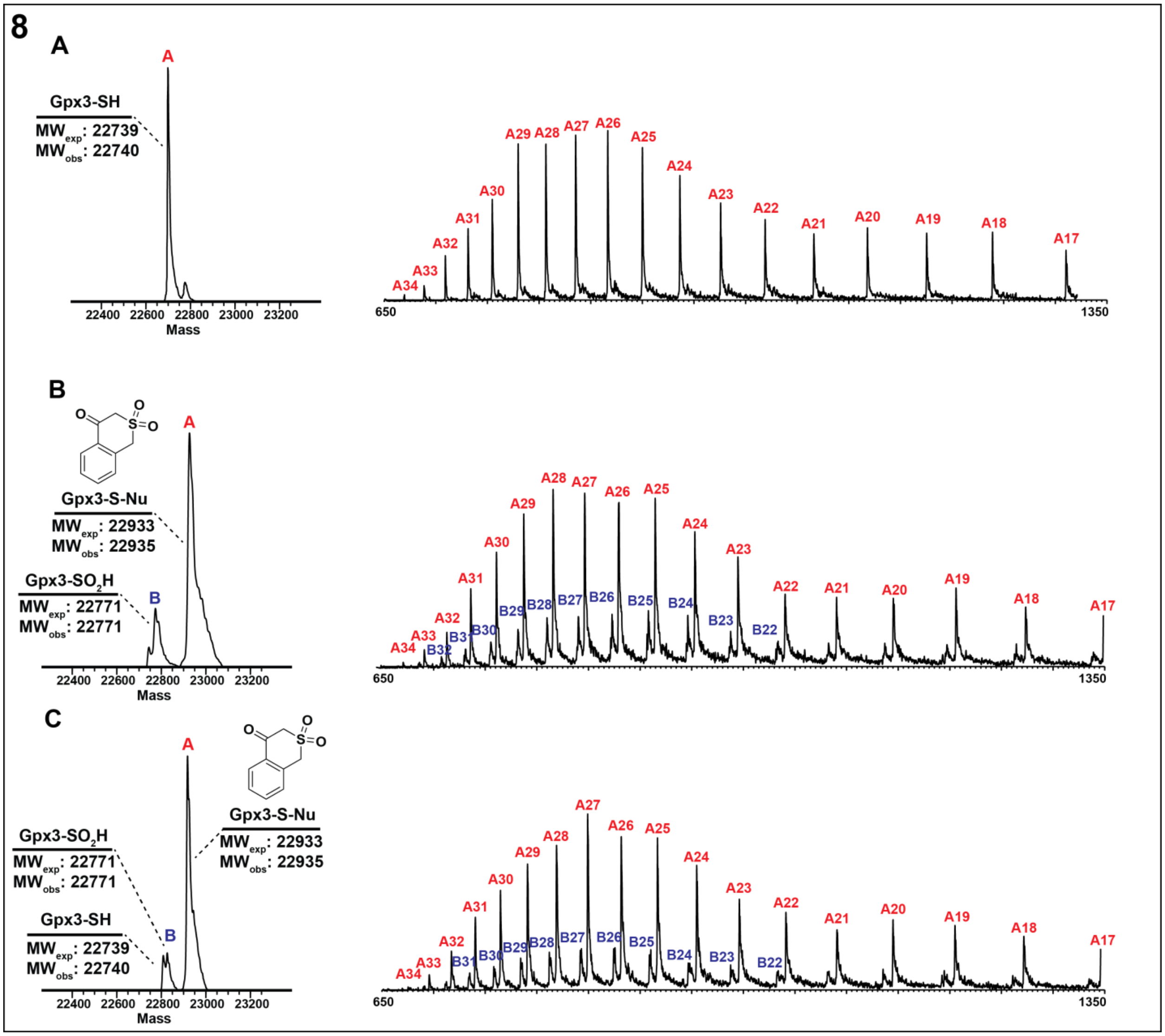

Fig. S39 Reaction of C64, 82S Gpx3 with isothiochroman-4-one 2,2-dioxide (31b) – (A) Gpx3 (10 μM) + TCEP (10 mM) + **34b** (1 mM); **(B)** Gpx3 (10 μM) + **34b** (100 μM) + H_2O_2 (1.5 eq); **(C)** Gpx3 (10 μM) + **34b** (1 mM) + H_2O_2 (1.5 eq)

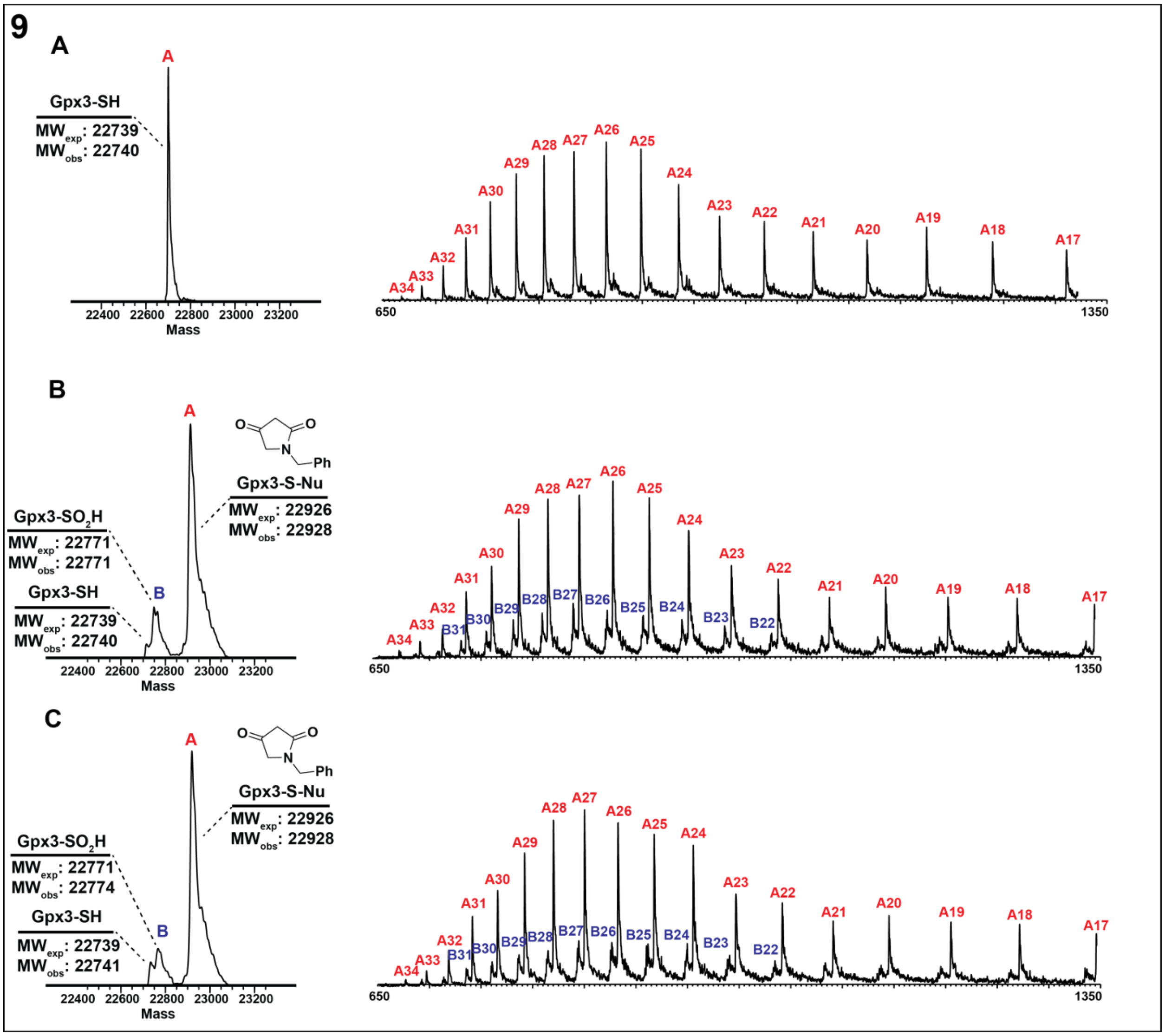

Fig. S40 Reaction of C64, 82S Gpx3 with 1-benzylpyrrolidine-2,4-dione (32c) – (A) Gpx3 (10 μM) + TCEP (10 mM) + **34b** (1 mM); **(B)** Gpx3 (10 μM) + **34b** (100 μM) + H_2O_2 (1.5 eq); **(C)** Gpx3 (10 μM) + **34b** (1 mM) + H_2O_2 (1.5 eq)

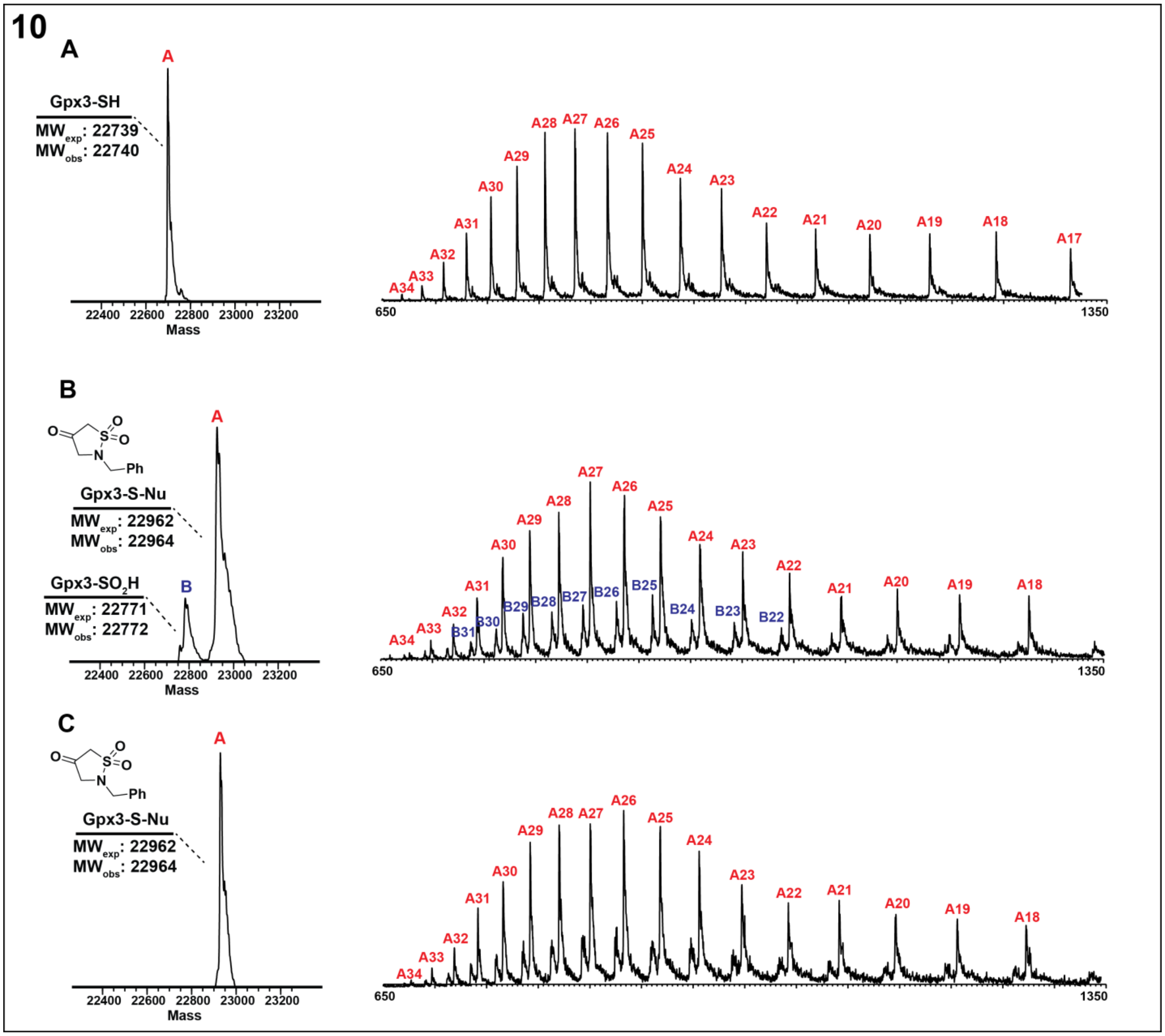

Fig. S41 Reaction of C64, 82S Gpx3 with 2-benzylisothiazolidin-4-one 1,1-dioxide (33f) – (A) Gpx3 (10 μM) + TCEP (10 mM) + **34b** (1 mM); **(B)** Gpx3 (10 μM) + **34b** (100 μM) + H_2O_2 (1.5 eq); **(C)** Gpx3 (10 μM) + **34b** (1 mM) + H_2O_2 (1.5 eq)

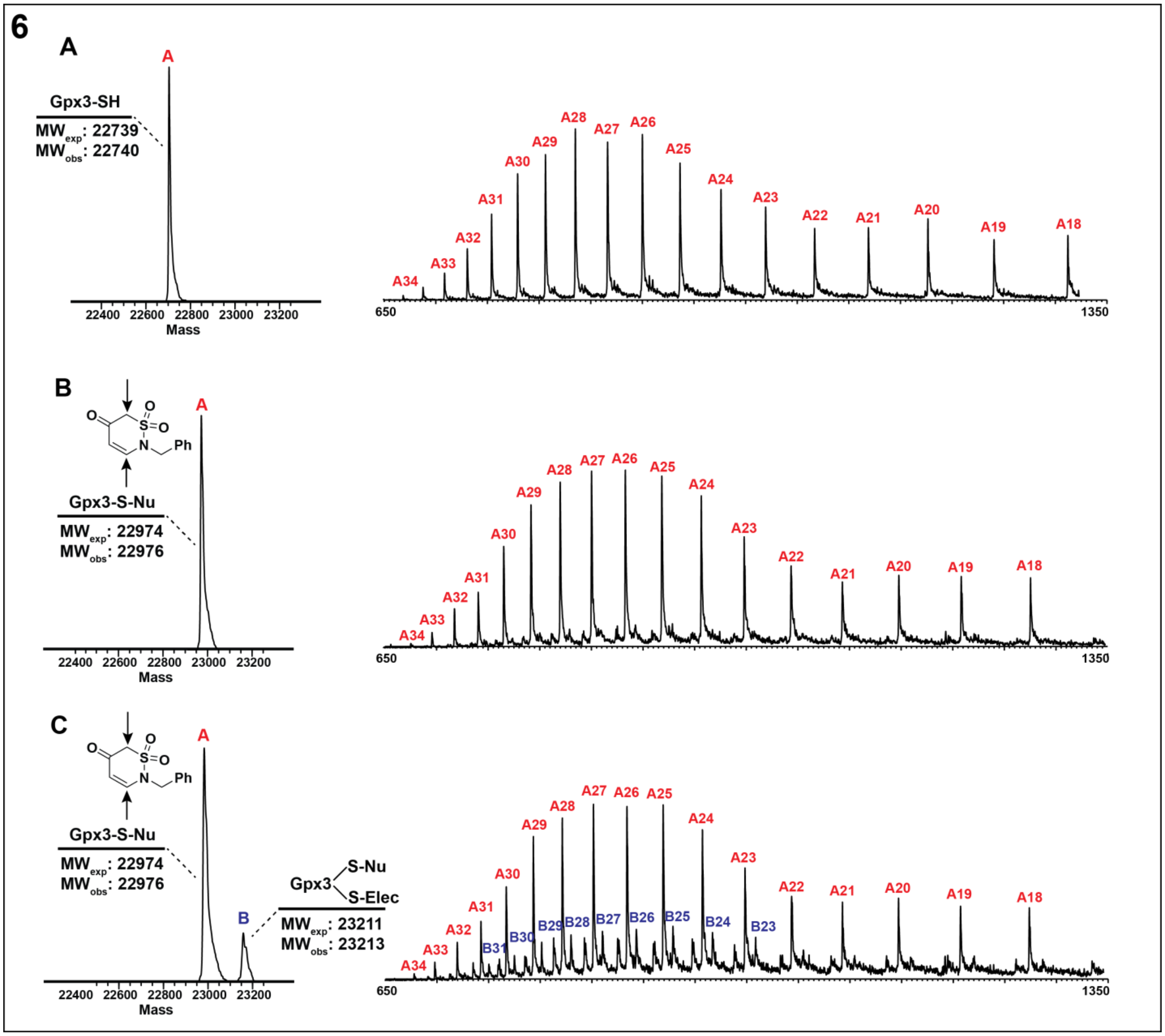

Fig. S42 Reaction of C64, 82S Gpx3 with 2-benzyl-2*H*-1,2-thiazin-5(6*H*)-one 1,1-dioxide (31f) – (A) Gpx3 (10 μM) + TCEP (10 mM) + **34b** (1 mM); **(B)** Gpx3 (10 μM) + **34b** (100 μM) + H_2O_2 (1.5 eq); **(C)** Gpx3 (10 μM) + **34b** (1 mM) + H_2O_2 (1.5 eq)

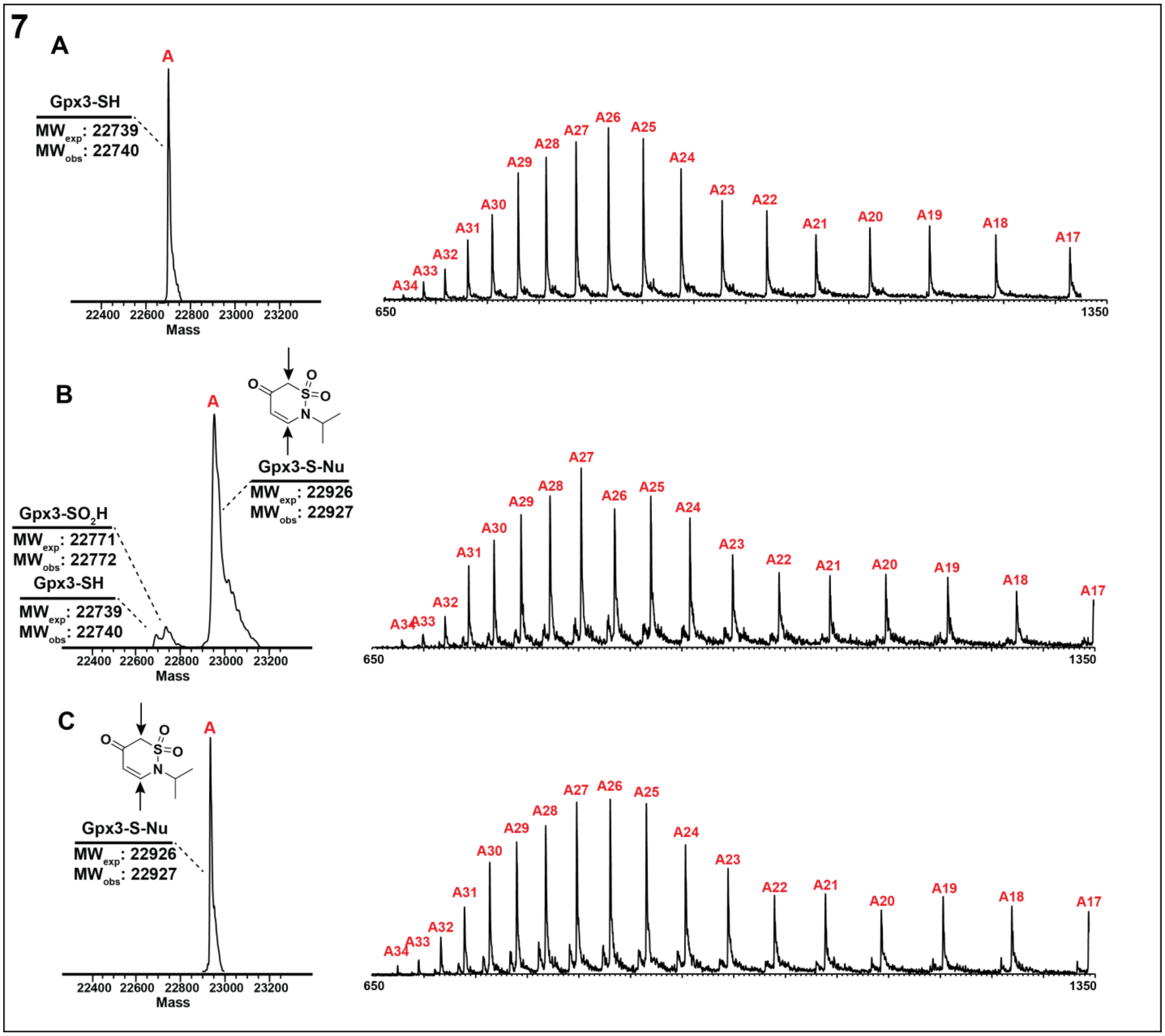

Fig. S43 Reaction of C64, 82S Gpx3 with 2-isopropyl-2*H*-1,2-thiazin-5(6*H*)-one 1,1-dioxide (31e) – (A) Gpx3 (10 μM) + TCEP (10 mM) + **34b** (1 mM); **(B)** Gpx3 (10 μM) + **34b** (100 μM) + H_2O_2 (1.5 eq); **(C)** Gpx3 (10 μM) + **34b** (1 mM) + H_2O_2 (1.5 eq)

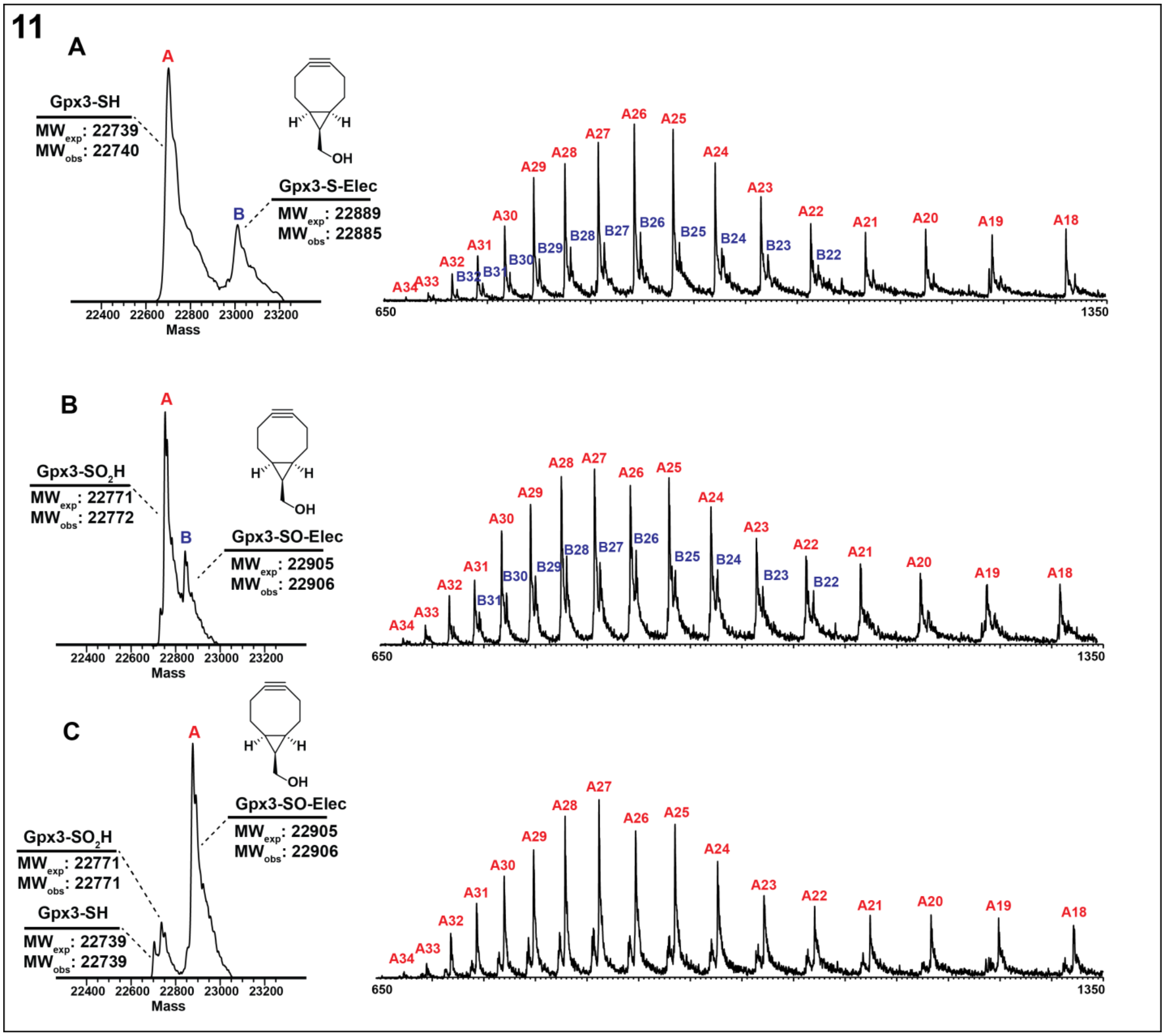

Fig. S44 Reaction of C64, 82S Gpx3 with ((1*R*,8*S*,9*s*)-bicyclo[6.1.0]non-4-yn-9-yl)methanol (5) – (A) Gpx3 (10 μM) + TCEP (10 mM) + **1** (1 mM); **(B)** Gpx3 (10 μM) + **1** (100 μM) + H_2O_2 (1.5 eq); **(C)** Gpx3 (10 μM) + **1** (1 mM) + H_2O_2 (1.5 eq)

Scheme S23. Competitive reaction of various nucleophiles and dimedone towards C64,82S Gpx3-SOH

Reaction:

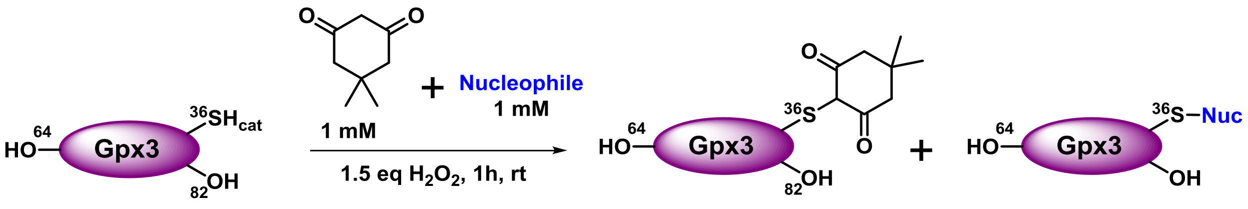

C64,82S Gpx3 (10 μM)

Purpose: To label Gpx3 with various nucleophiles in the presence of dimedone

Nucleophiles/Electrophiles tested:

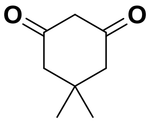

1

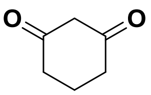

22a

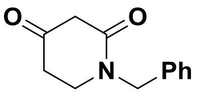

26f

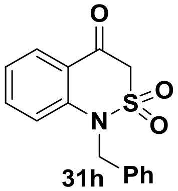

31h

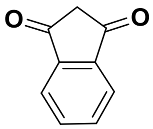

34b

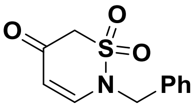

31f

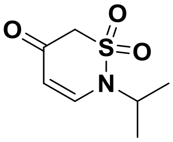

31e

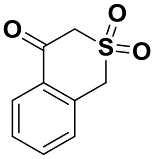

31b

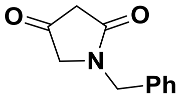

32c

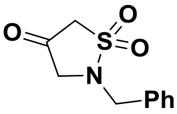

33f

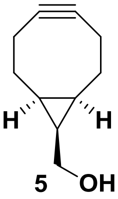

5

Procedure:

(1) 100 mM stock solutions of nucleophiles and the electrophile were prepared in DMSO – If not soluble at room temperature, the eppendorf tubes were heated in 100 ^{o}C water bath for 2 minutes. 100 mM stock solutions were then diluted to 20 mM solution in Gpx3 labeling buffer (50 mM HEPES, 100 mM NaCl, pH = 7.4).

(2) C64, 82S Gpx3 was thawed at 0 ^{o}C and reduced with 50 mM (2M, 5 µL) DTT for 20 minutes on ice - A 2 M solution of DTT in water was prepared (31 mg of DTT in 100 µl of water). 5 µl of this DTT solution was added to the protein solution (200 µl) giving an effective DTT concentration of 50 mM.

(3) Buffer exchange C64, 82S Gpx3 to labeling buffer (50 mM HEPES, 100 mM NaCl, pH = 7.4) using pre-equilibrated Nap-5 column. Nap-5 column was equilibrated by passing 10 ml of Gpx3 labeling buffer.

(4) Determine the conc. of C64, 82S Gpx3 by using A_{280} (ε = 24410 $M^{-1}cm^{-1}$).

(5) Label 10 µM of C64, 82S Gpx3 with 1.5 eq of H_2O_2 and a mixture of 1 mM nucleophile & 1 mM dimedone, keeping DMSO $\leq$ 5%. Incubate for 1 h [REACTION VOLUME = 100 µl].

Order of addition was as follow:

(a) Appropriate amounts of Gpx3 labeling buffer were added to the eppy tubes.

(b) C64, 82S Gpx3 was added to the tube and the contents were mixed gently.

(c) Subsequently, probe solution (mixture of 20 mM probe and 20 mM dimedone) was added to each of the tubes. For this 5 µL of a 20 mM solution of each probe and dimedone was used. This gave 1 mM effective probe concentration for each reaction and kept the DMSO concentration at < 5%.

(d) Hydrogen peroxide solution was added last. A 1 mM solution of hydrogen peroxide was prepared in water by serial dilution starting with commercially available 8.8 M solution. 1.5 µl of this 1 mM peroxide solution was used in each case to give an effective concentration of 15 µM.

(e) Reaction mixtures were incubated for 1 h at rt.

(6) The reaction mixtures were quenched by the filtration through pre-equilibrated P30 columns.

(7) During the filtration through P30 columns, each sample was buffer exchanged to 25 mM ammonium bicarbonate (pH 8.0).

(8) Each sample was then analyzed by LTQ-MS by injecting 3 µM concentration.

Controls

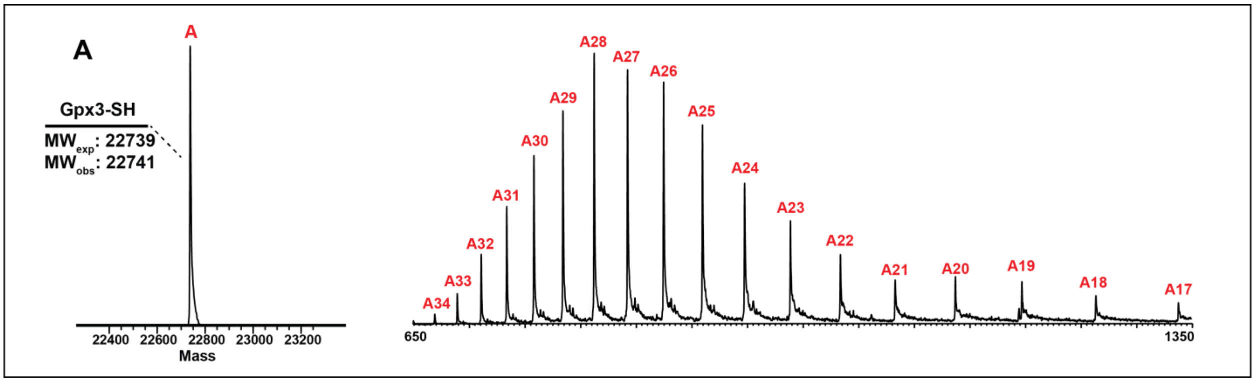

Fig. S45 C64,82S Gpx3-SH (reduced form) - control

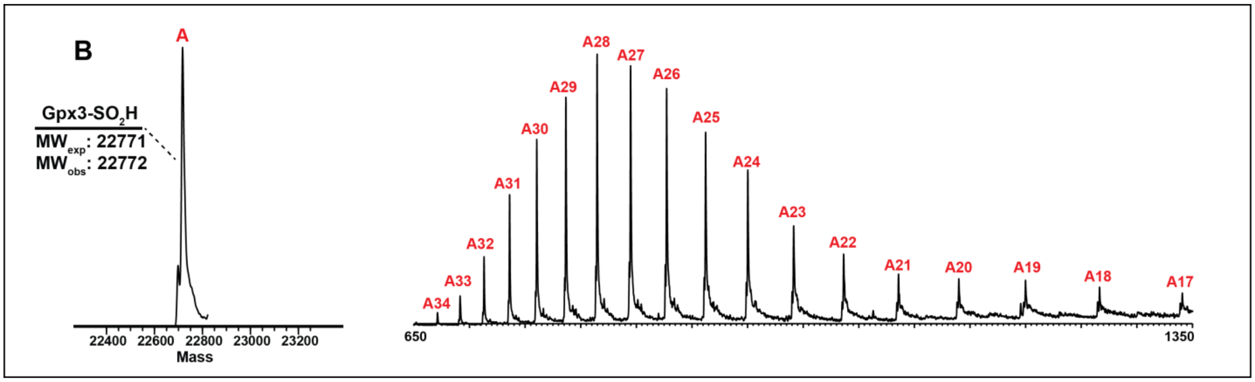

Fig. S46 C64,82S Gpx3-SOH (oxidized form) - control

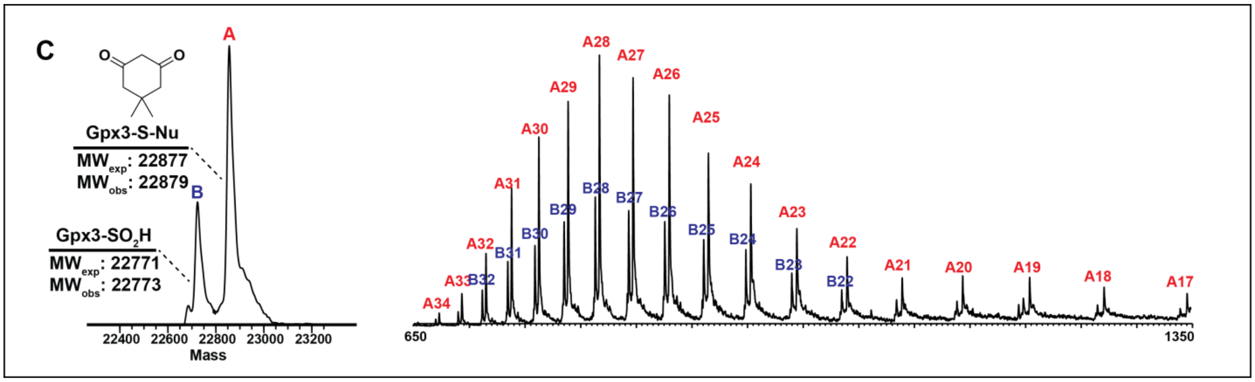

Fig. S47 C64,82S Gpx3-SH + Dimedone (1, 1 mM) + H_2O_2 (1.5 eq) – control

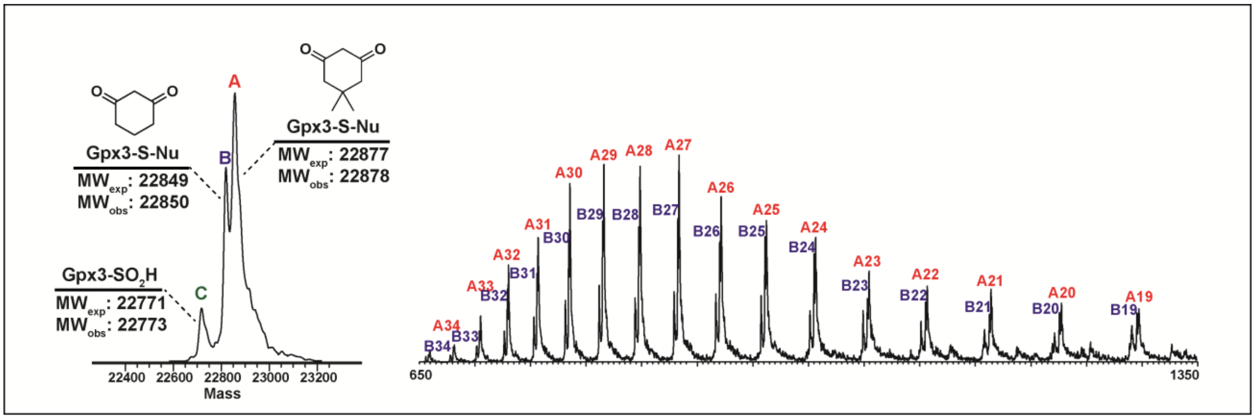

Fig. S48 C64,82S Gpx3-SH + 22a (1 mM) + Dimedone (1 mM) + H_2O_2 (1.5 eq)

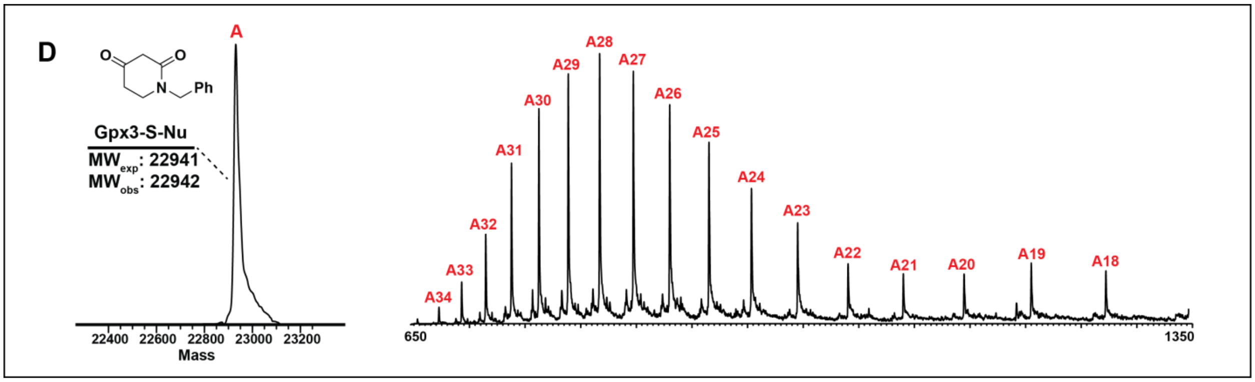

Fig. S49 C64,82S Gpx3-SH + 26f (1 mM) + Dimedone (1 mM) + H_2O_2 (1.5 eq)

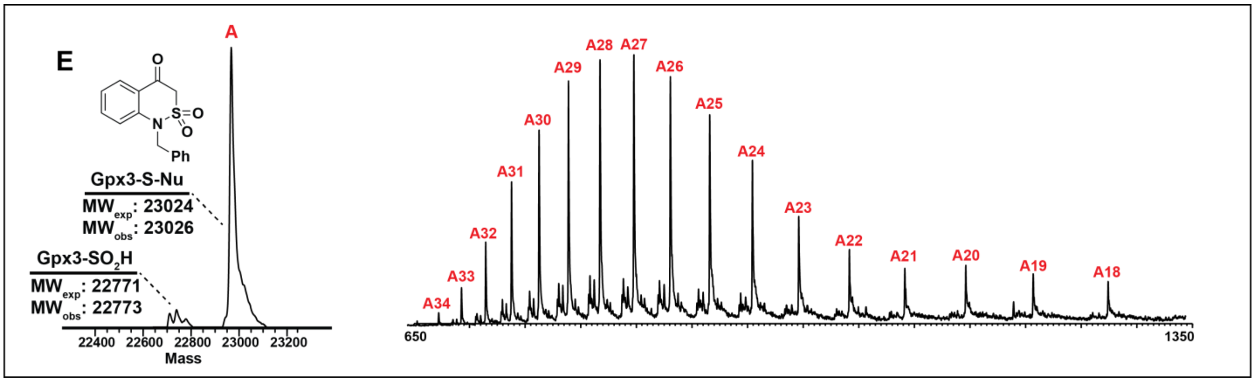

Fig. S50 C64,82S Gpx3-SH + 31h (1 mM) + Dimedone (1 mM) + H_2O_2 (1.5 eq)

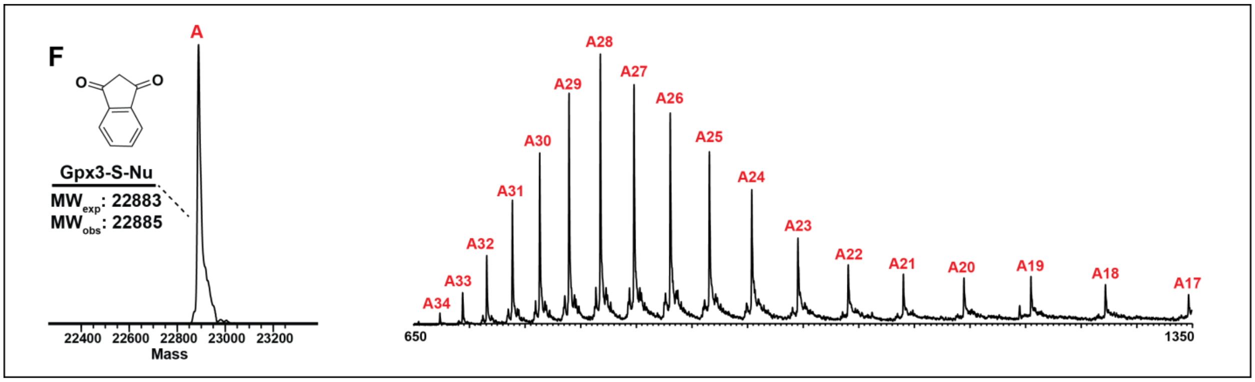

Fig. S51 C64,82S Gpx3-SH + 34b (1 mM) + Dimedone (1 mM) + H_2O_2 (1.5 eq)

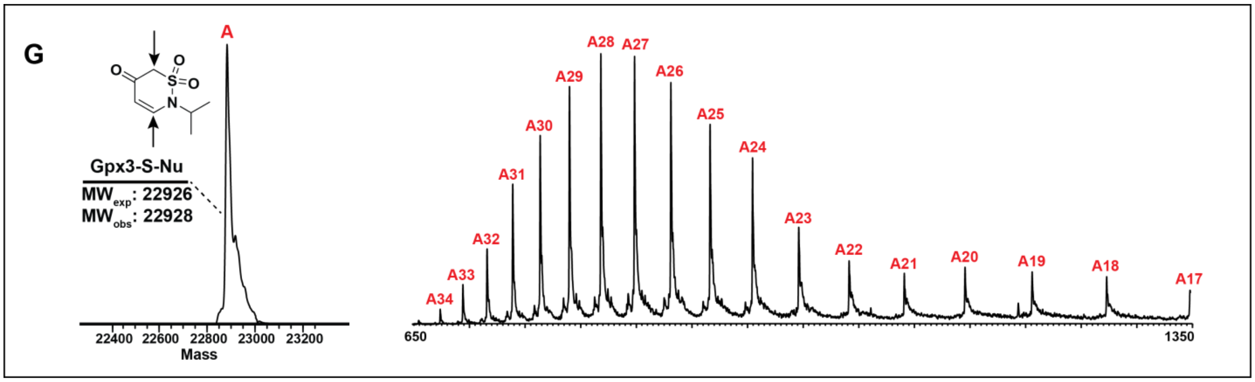

Fig. S52 C64,82S Gpx3-SH + 31e (1 mM) + Dimedone (1 mM) + H_2O_2 (1.5 eq)

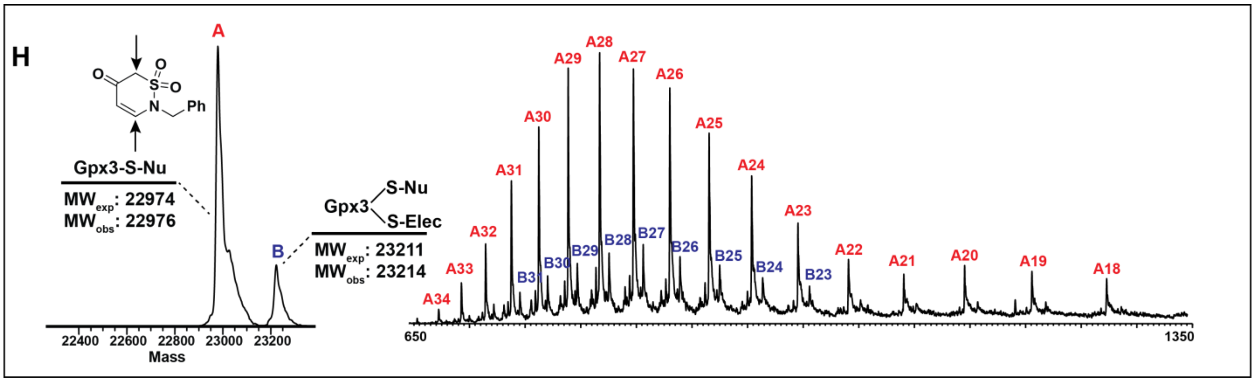

Fig. S53 C64,82S Gpx3-SH + 31f (1 mM) + Dimedone (1 mM) + H_2O_2 (1.5 eq)

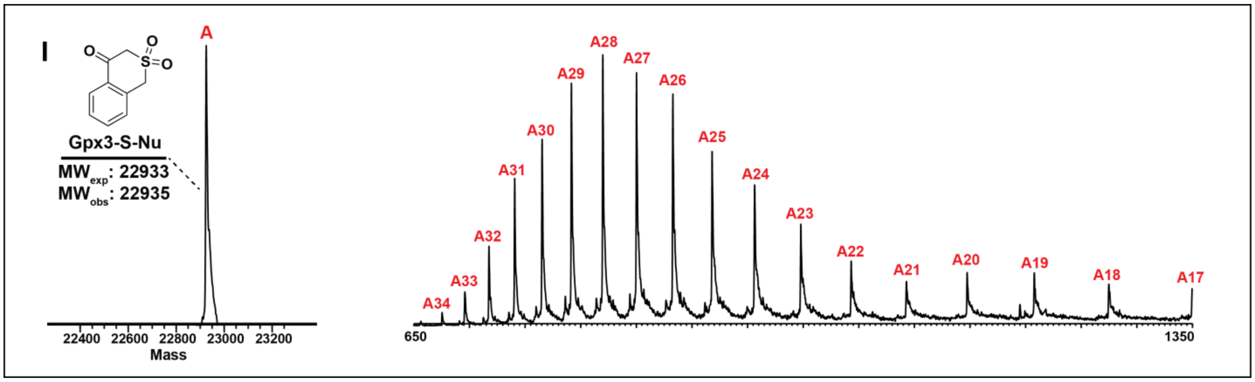

Fig. S54 C64,82S Gpx3-SH + 31b (1 mM) + Dimedone (1 mM) + H_2O_2 (1.5 eq)

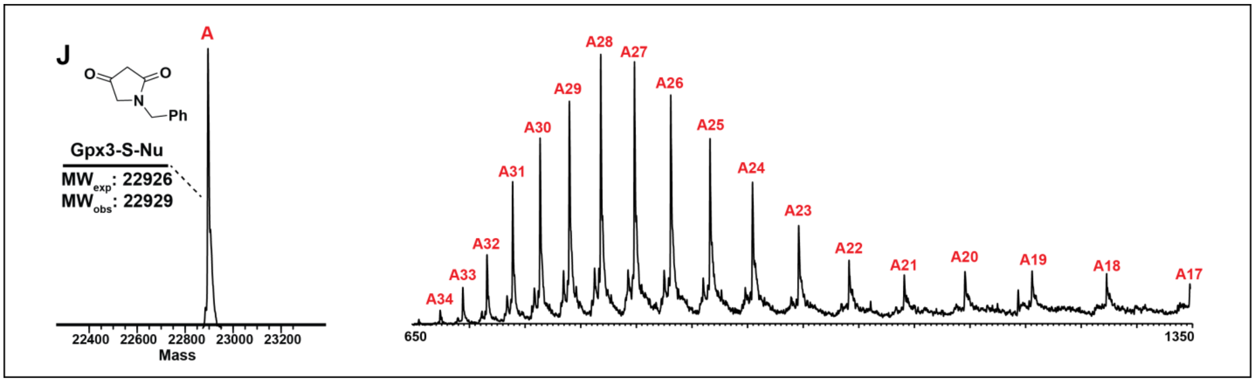

Fig. S55 C64,82S Gpx3-SH + 32c (1 mM) + Dimedone (1 mM) + H_2O_2 (1.5 eq)

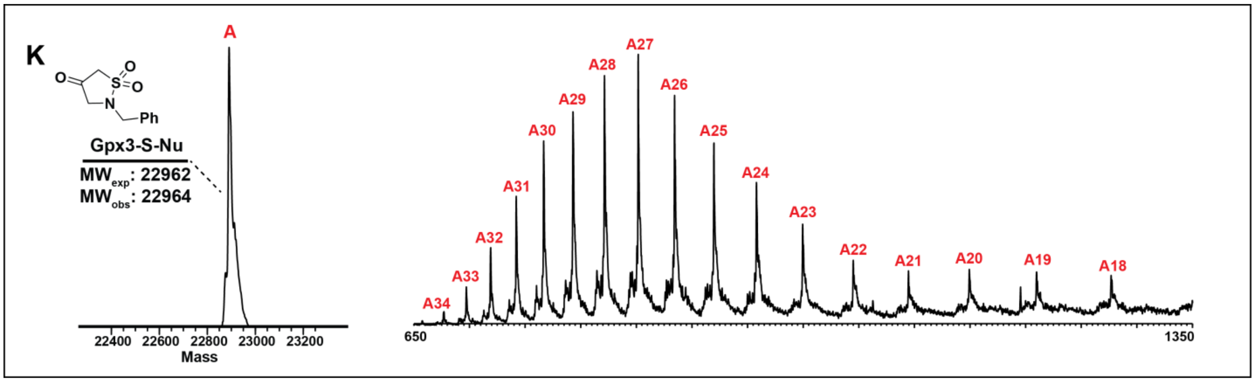

Fig. S56 C64,82S Gpx3-SH + 33f (1 mM) + Dimedone (1 mM) + H_2O_2 (1.5 eq)

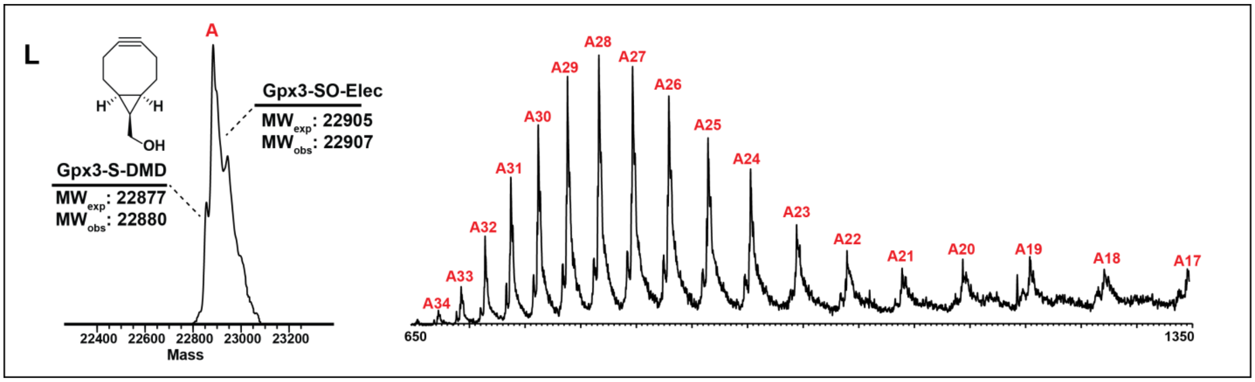

Fig. S57 C64,82S Gpx3-SH + BCN (1 mM) + Dimedone (1 mM) + H_2O_2 (1.5 eq)

Table S1 – Effect of keto-enol tautomerism on the reactivity of C-nucleophiles towards sulfenic acid

#	Structure	k_{obs} (min^{-1})	Keto:Enol
A	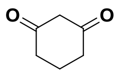	0.4	100% enol
B	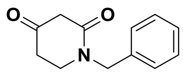	63.5	10:3
C	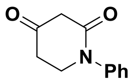	17.3	5:4
D	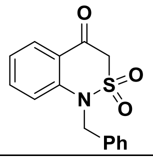	161.5	10:1
E	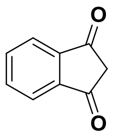	21.6	Keto only
F	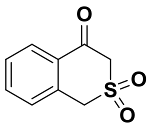	48.0	Keto only
G	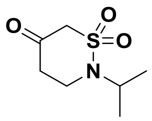	0.6	Keto only
H	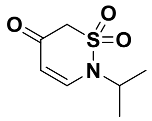	45.1	10:1
I	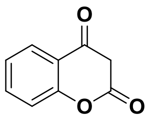	Very slow	Enol only

**J**	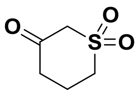	**2.0**	**Keto only**
**K**	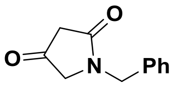	**21.3**	**2:1**
**L**	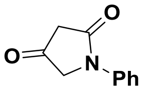	**5.2**	**1:1**

(A)

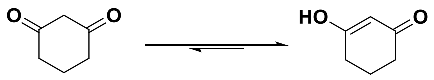

~ 100% enol

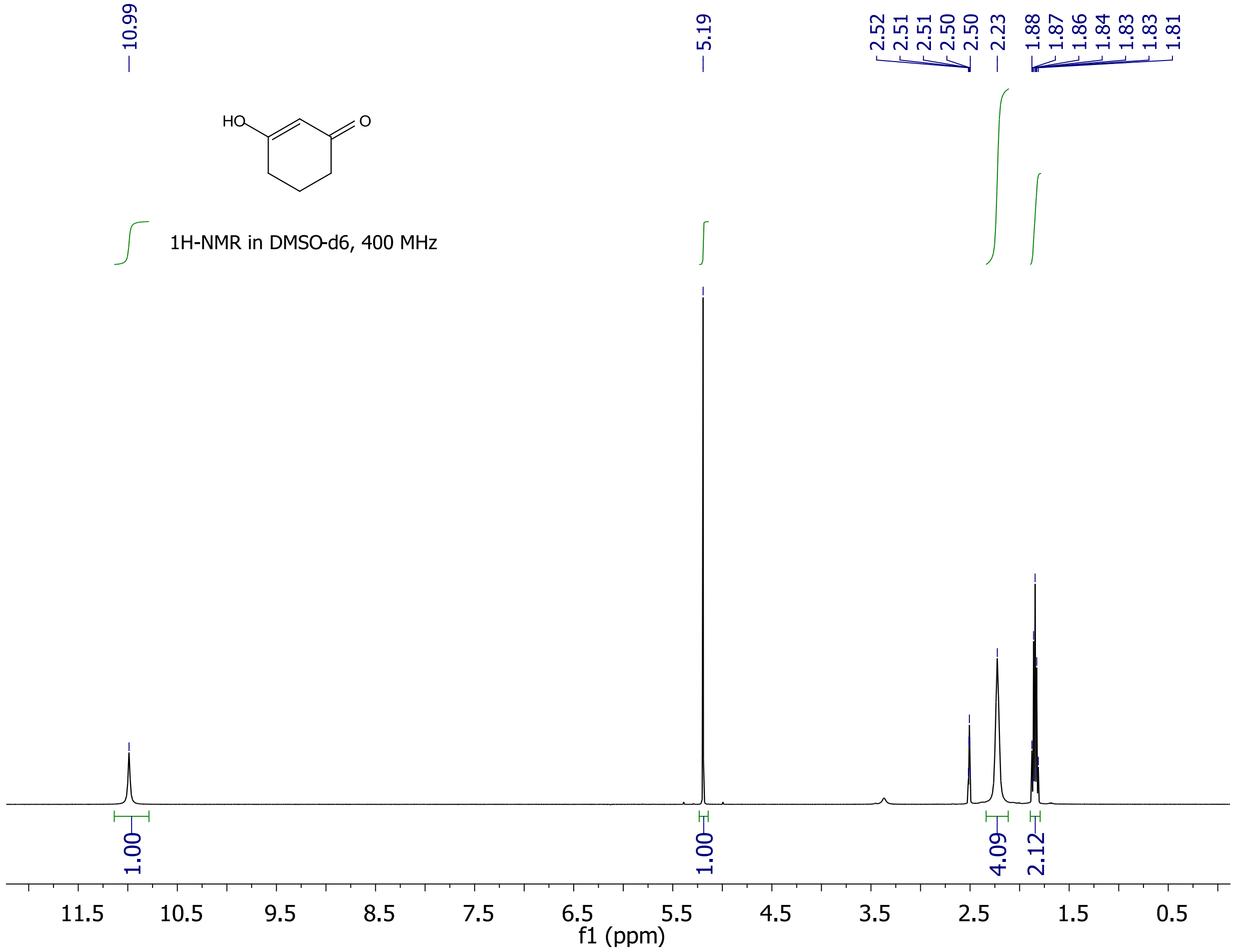
HO
O
1H-NMR in DMSO-d6, 400 MHz
10.99
5.19
2.52
2.51
2.51
2.50
2.50
2.23
1.88
1.87
1.86
1.84
1.83
1.83
1.81
1.00
1.00
4.09
2.12
11.5
10.5
9.5
8.5
7.5
6.5
5.5
4.5
3.5
2.5
1.5
0.5
f1 (ppm)

(B)

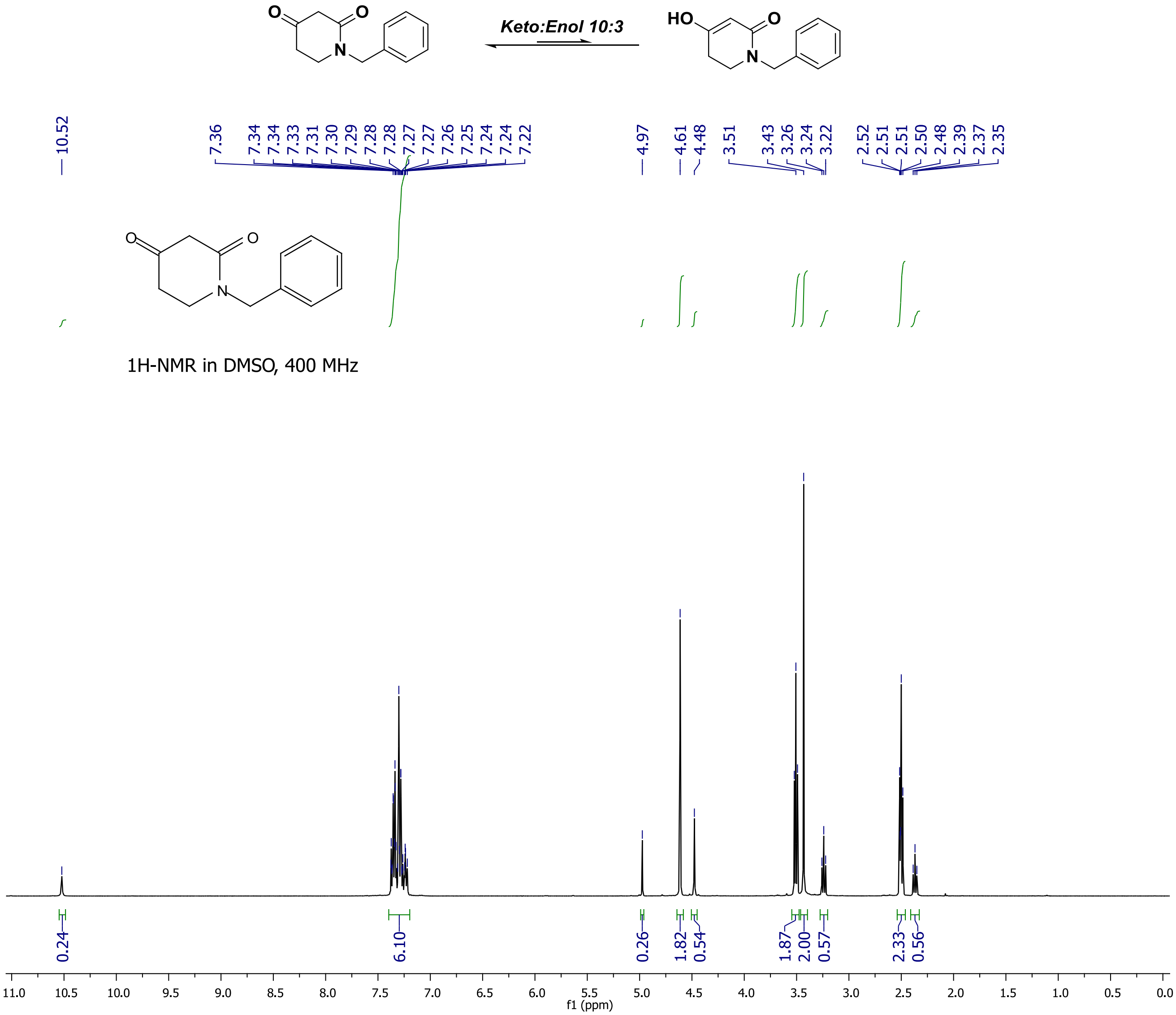

(C)

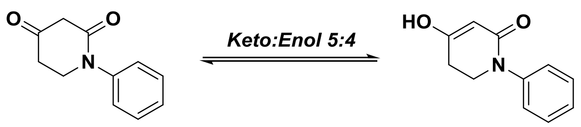
Keto:Enol 5:4
HO
N
O

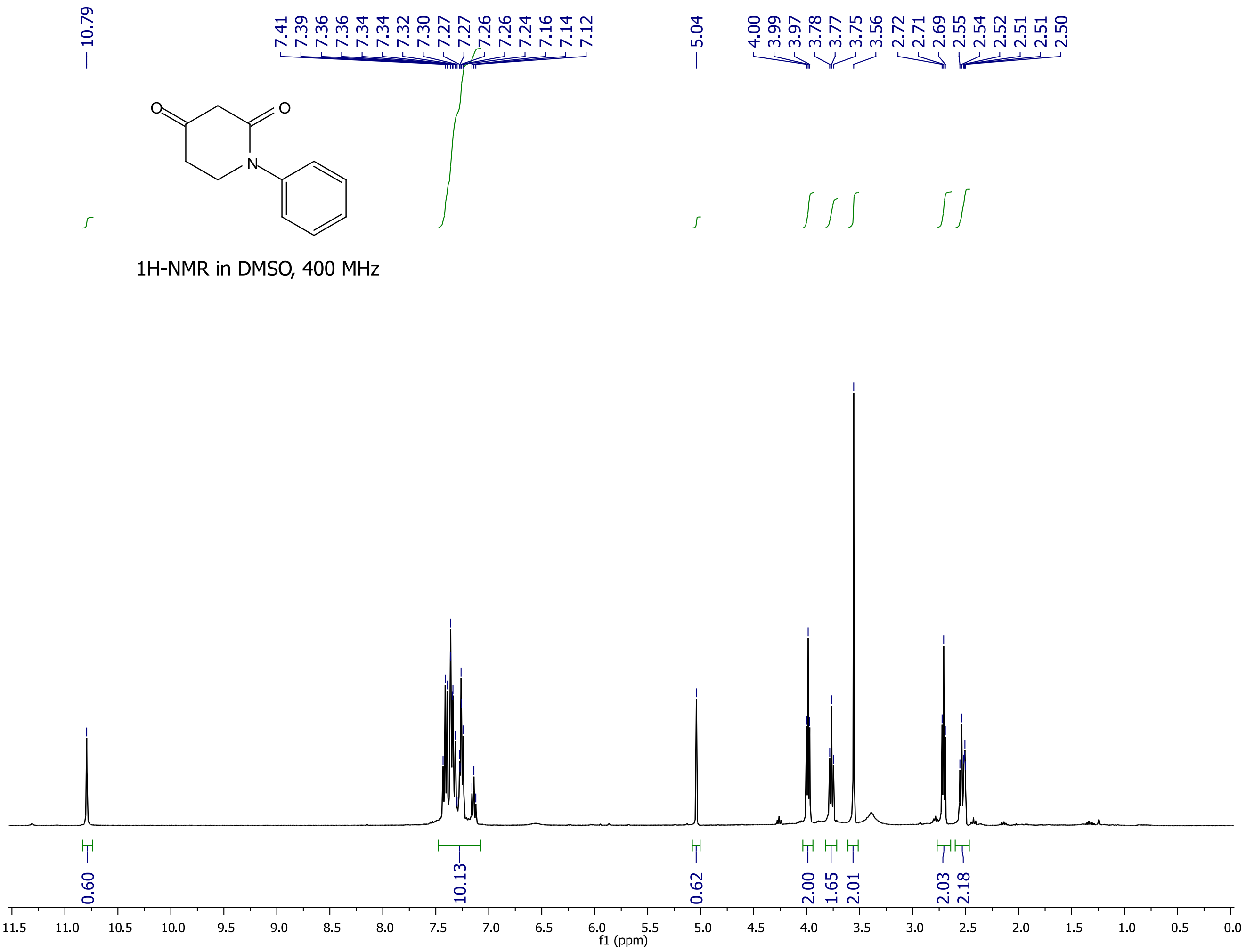
1H-NMR in DMSO, 400 MHz
10.79
7.41
7.39
7.36
7.36
7.34
7.34
7.32
7.30
7.27
7.27
7.26
7.26
7.24
7.16
7.14
7.12
5.04
4.00
3.99
3.97
3.78
3.77
3.75
3.56
2.72
2.71
2.69
2.55
2.54
2.52
2.51
2.51
2.50
0.60
10.13
0.62
2.00
1.65
2.01
2.03
2.18
f1 (ppm)

(D)

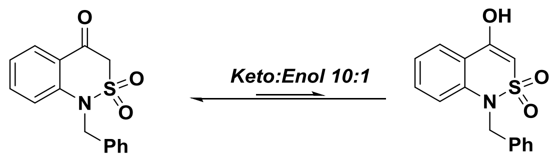
Keto:Enol 10:1
O
OH
N
S
O
Ph

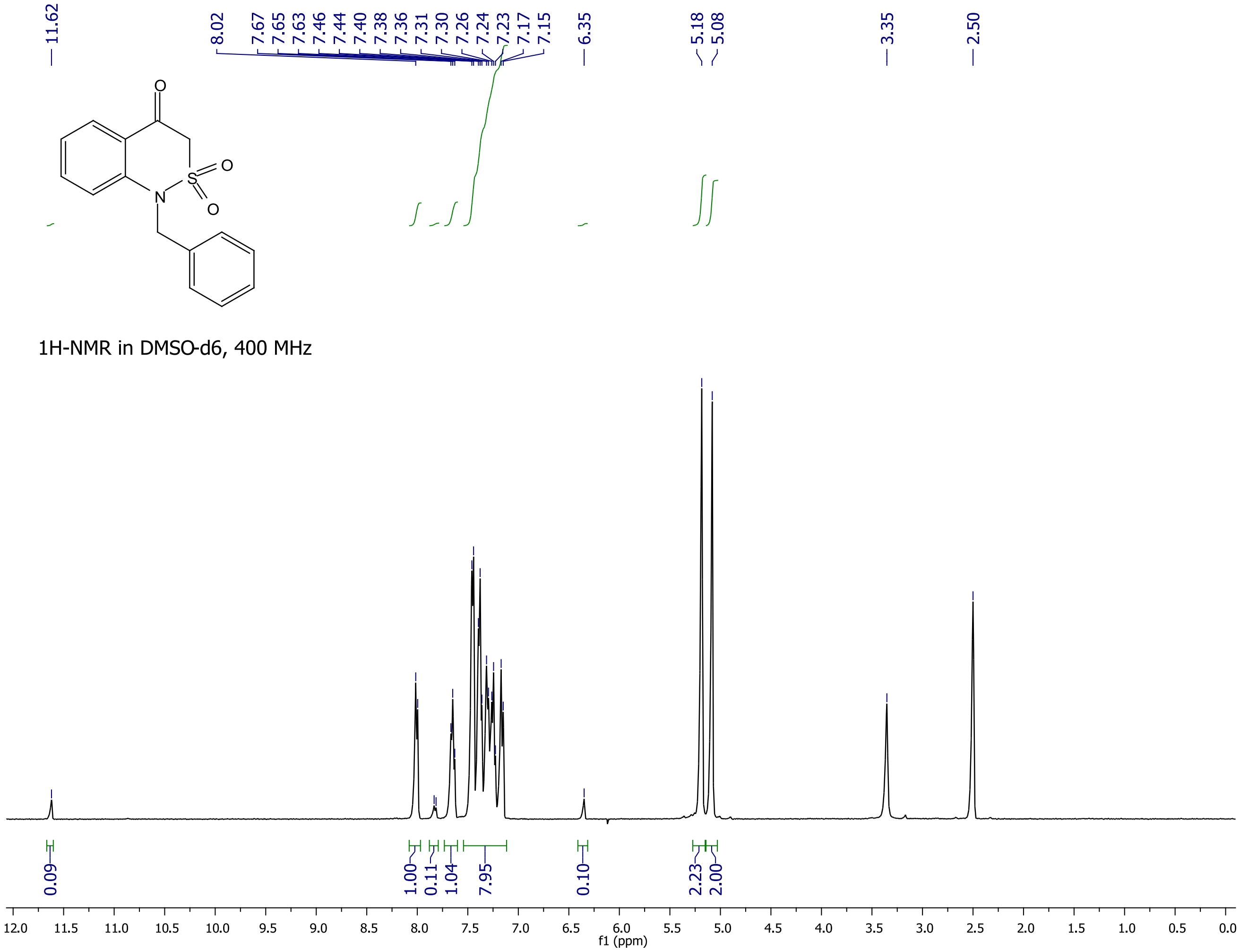
11.62
8.02
7.67
7.65
7.63
7.46
7.44
7.40
7.38
7.36
7.31
7.30
7.26
7.24
7.23
7.17
7.15
6.35
5.18
5.08
3.35
2.50
1H-NMR in DMSO-d6, 400 MHz
0.09
1.00
0.11
1.04
7.95
0.10
2.23
2.00
12.0 11.5 11.0 10.5 10.0 9.5 9.0 8.5 8.0 7.5 7.0 6.5 6.0 5.5 5.0 4.5 4.0 3.5 3.0 2.5 2.0 1.5 1.0 0.5 0.0
f1 (ppm)

(E)

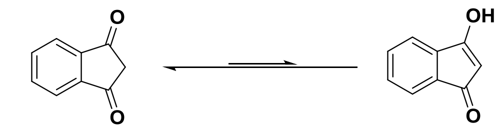

~ 100% Keto

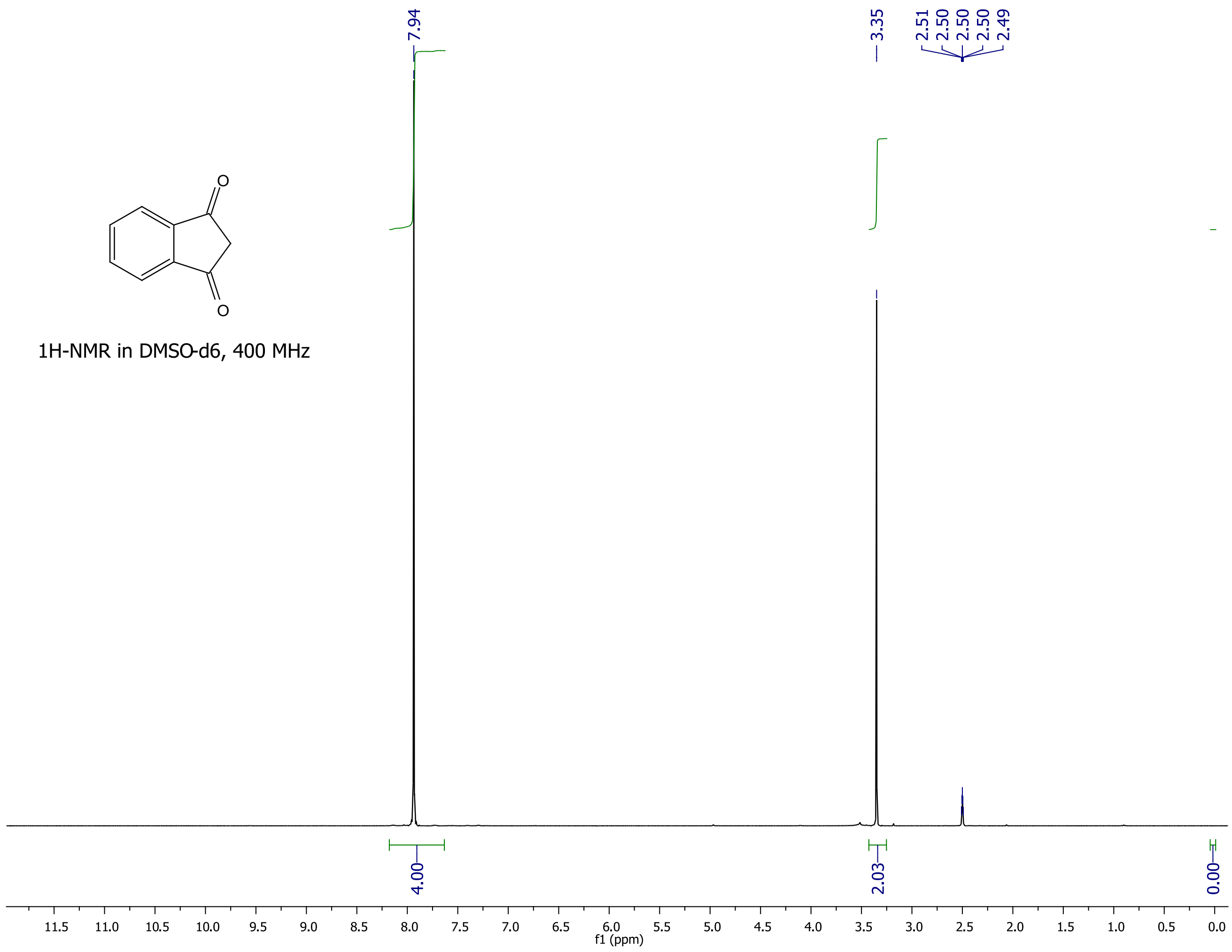
1H-NMR in DMSO-d6, 400 MHz
7.94
3.35
2.51
2.50
2.50
2.50
2.49
4.00
2.03
0.00
11.5 11.0 10.5 10.0 9.5 9.0 8.5 8.0 7.5 7.0 6.5 6.0 5.5 5.0 4.5 4.0 3.5 3.0 2.5 2.0 1.5 1.0 0.5 0.0
f1 (ppm)

(F)

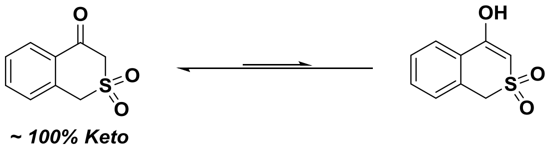
O
S
O
O
OH
S
O
O
~ 100% Keto

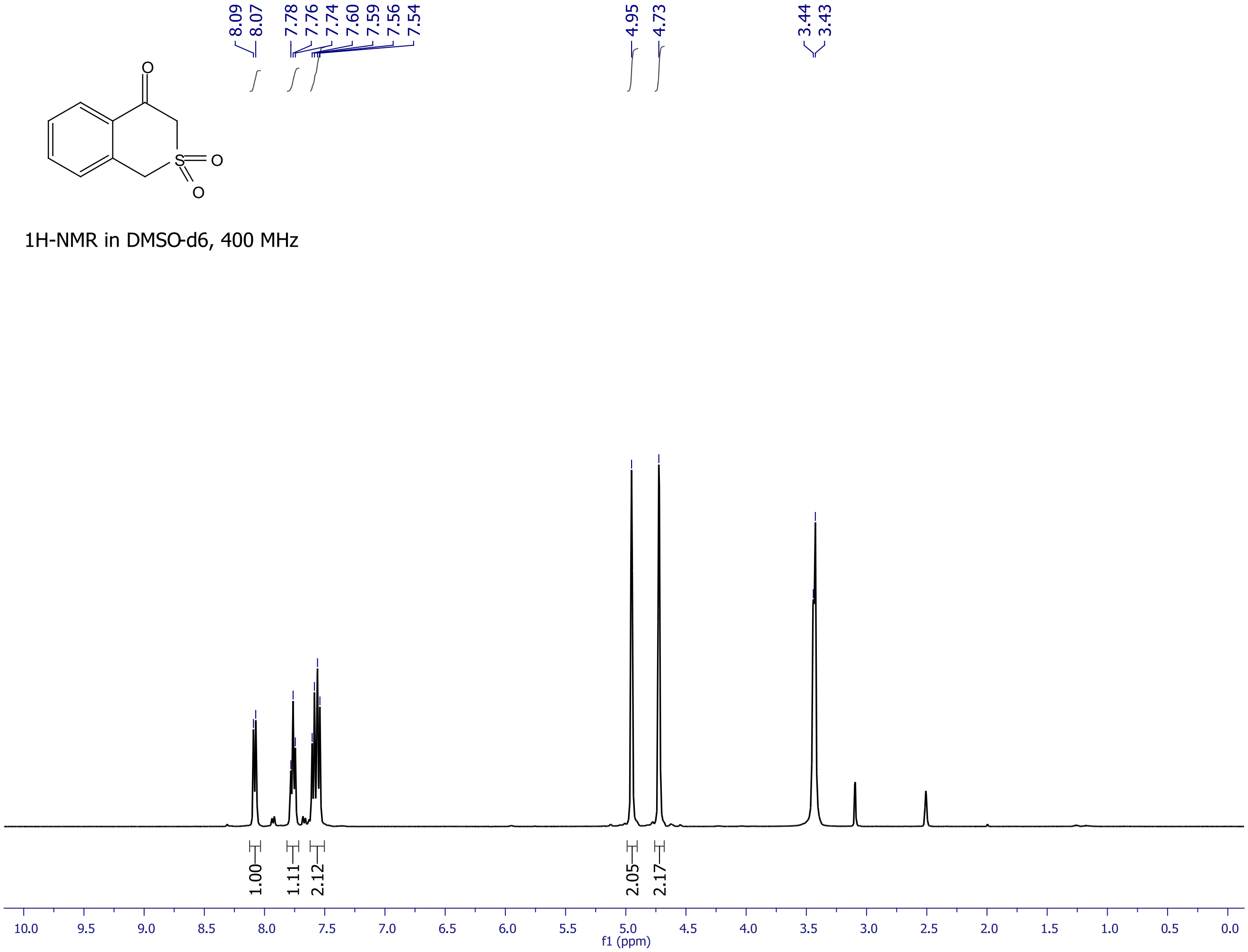
8.09
8.07
7.78
7.76
7.74
7.60
7.59
7.56
7.54
4.95
4.73
3.44
3.43
O
S
O
O
1H-NMR in DMSO-d6, 400 MHz
1.00
1.11
2.12
2.05
2.17
10.0
9.5
9.0
8.5
8.0
7.5
7.0
6.5
6.0
5.5
5.0
4.5
4.0
3.5
3.0
2.5
2.0
1.5
1.0
0.5
0.0
f1 (ppm)

(G)

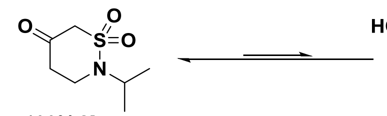

O
S=O
N
HO

~ 100% Keto

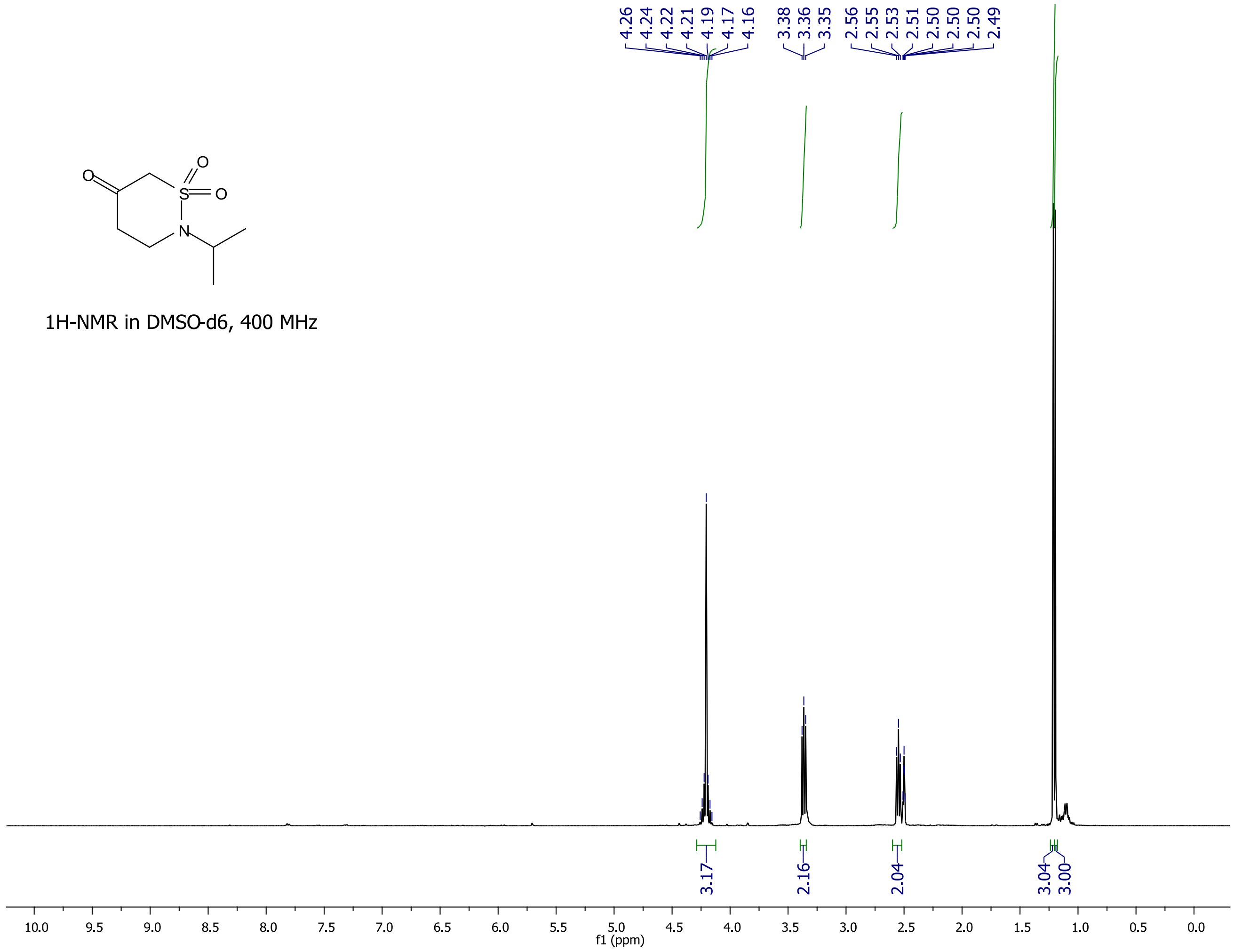

1H-NMR in DMSO-d6, 400 MHz
4.26
4.24
4.22
4.21
4.19
4.17
4.16
3.38
3.36
3.35
2.56
2.55
2.53
2.51
2.50
2.50
2.50
2.49
3.17
2.16
2.04
3.04
3.00
10.0 9.5 9.0 8.5 8.0 7.5 7.0 6.5 6.0 5.5 5.0 4.5 4.0 3.5 3.0 2.5 2.0 1.5 1.0 0.5 0.0
f1 (ppm)

(H)

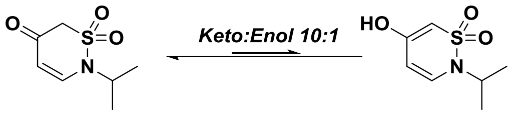
Keto:Enol 10:1
HO

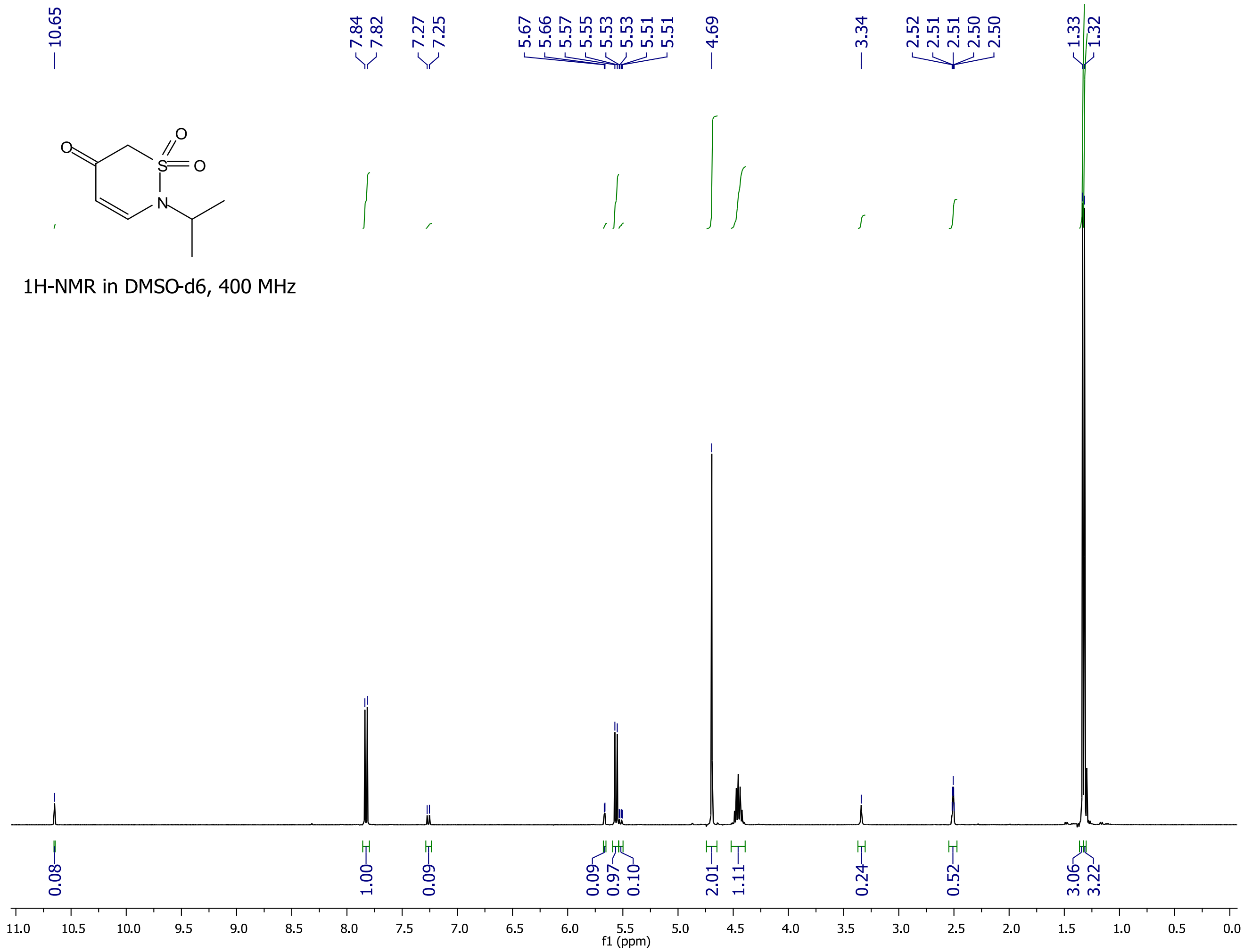
10.65
7.84
7.82
7.27
7.25
5.67
5.66
5.57
5.55
5.53
5.53
5.51
5.51
4.69
3.34
2.52
2.51
2.51
2.50
2.50
1.33
1.32
1H-NMR in DMSO-d6, 400 MHz
0.08
1.00
0.09
0.09
0.97
0.10
2.01
1.11
0.24
0.52
3.06
3.22
11.0
10.5
10.0
9.5
9.0
8.5
8.0
7.5
7.0
6.5
6.0
5.5
5.0
4.5
4.0
3.5
3.0
2.5
2.0
1.5
1.0
0.5
0.0
f1 (ppm)

(I)

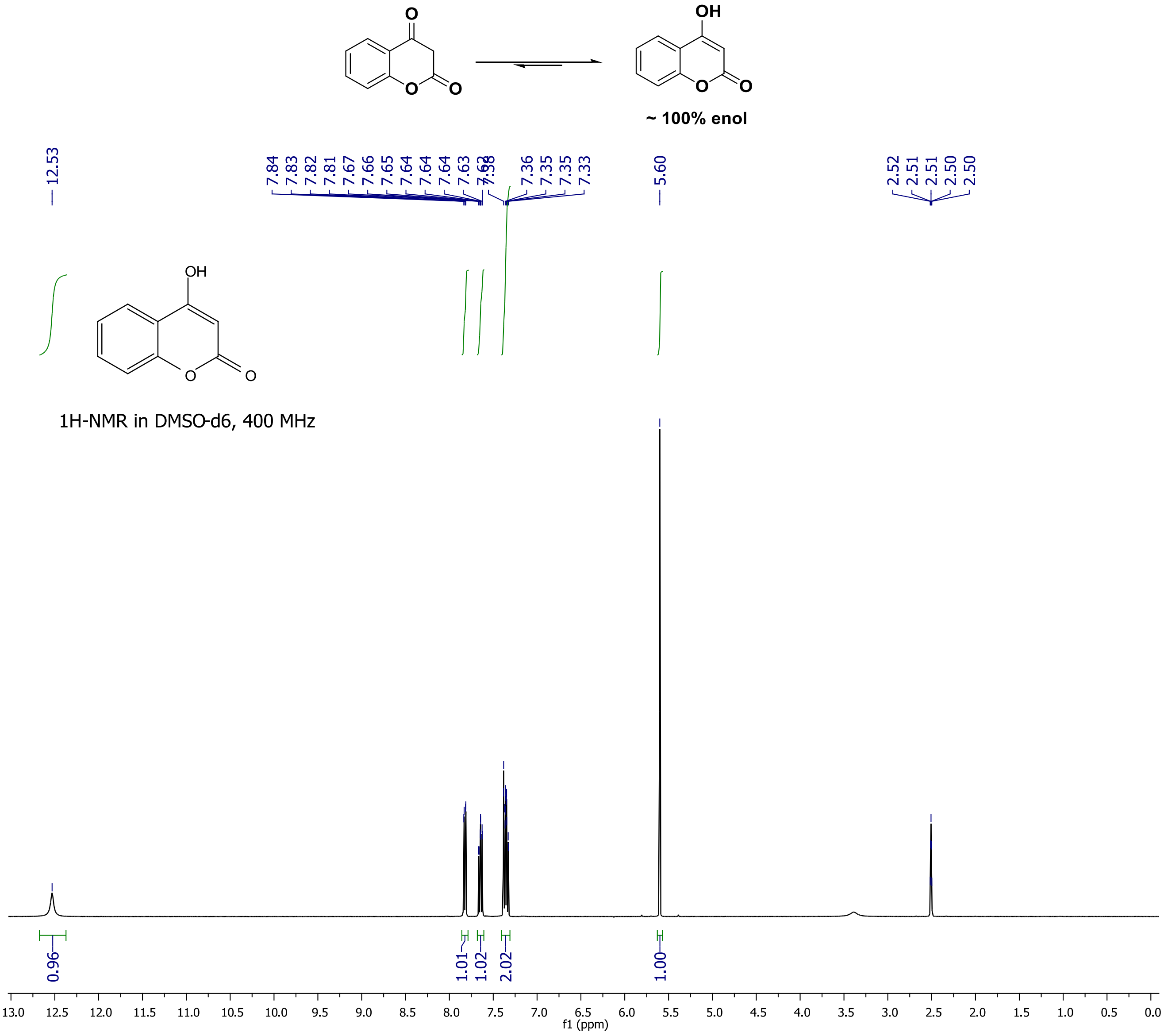

OH
~ 100% enol
12.53
7.84
7.83
7.82
7.81
7.67
7.66
7.65
7.64
7.64
7.63
7.62
7.38
7.36
7.35
7.35
7.33
5.60
2.52
2.51
2.51
2.50
2.50
1H-NMR in DMSO-d6, 400 MHz
0.96
1.01
1.02
2.02
1.00
f1 (ppm)

(J)

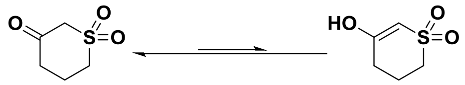

~ 100% Keto

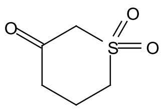

1H-NMR in DMSO-d6, 400 MHz

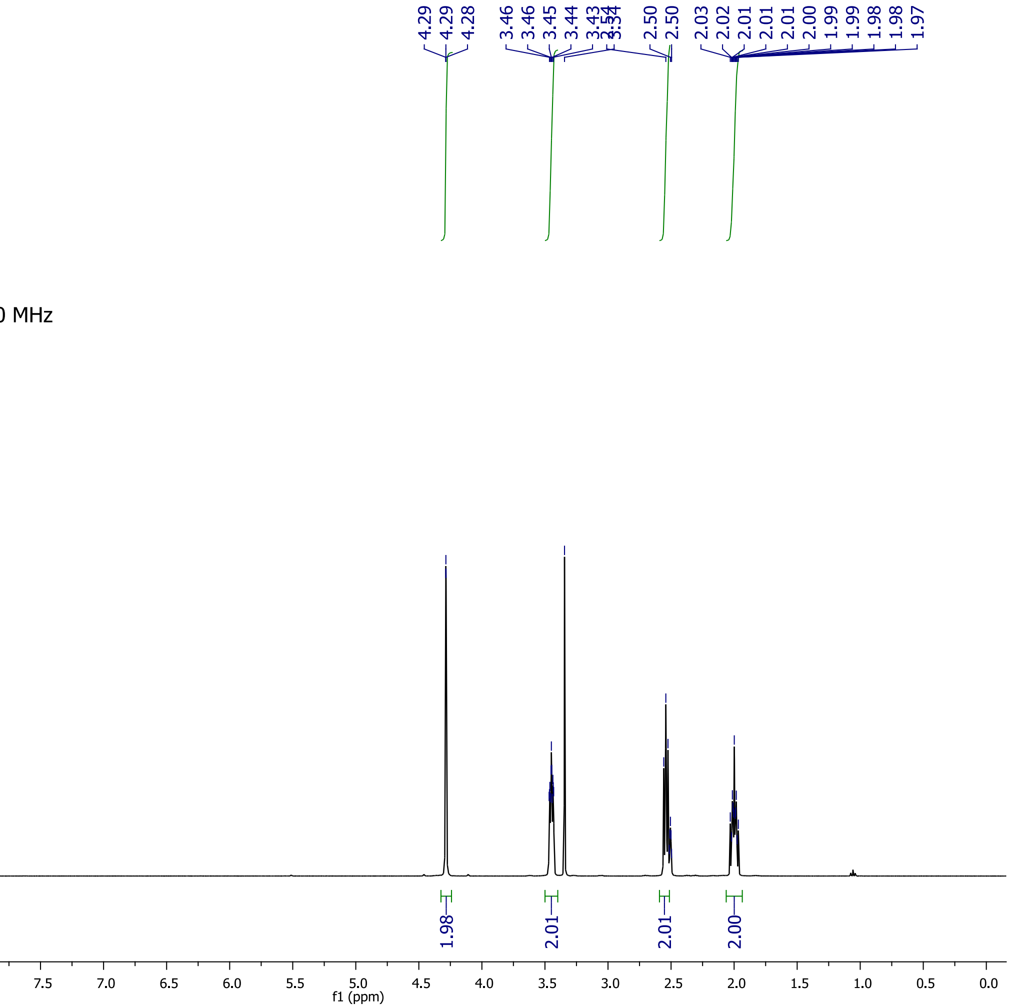

(K)

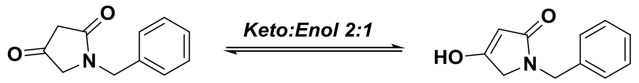
Keto:Enol 2:1
O
O
N
HO
O
N

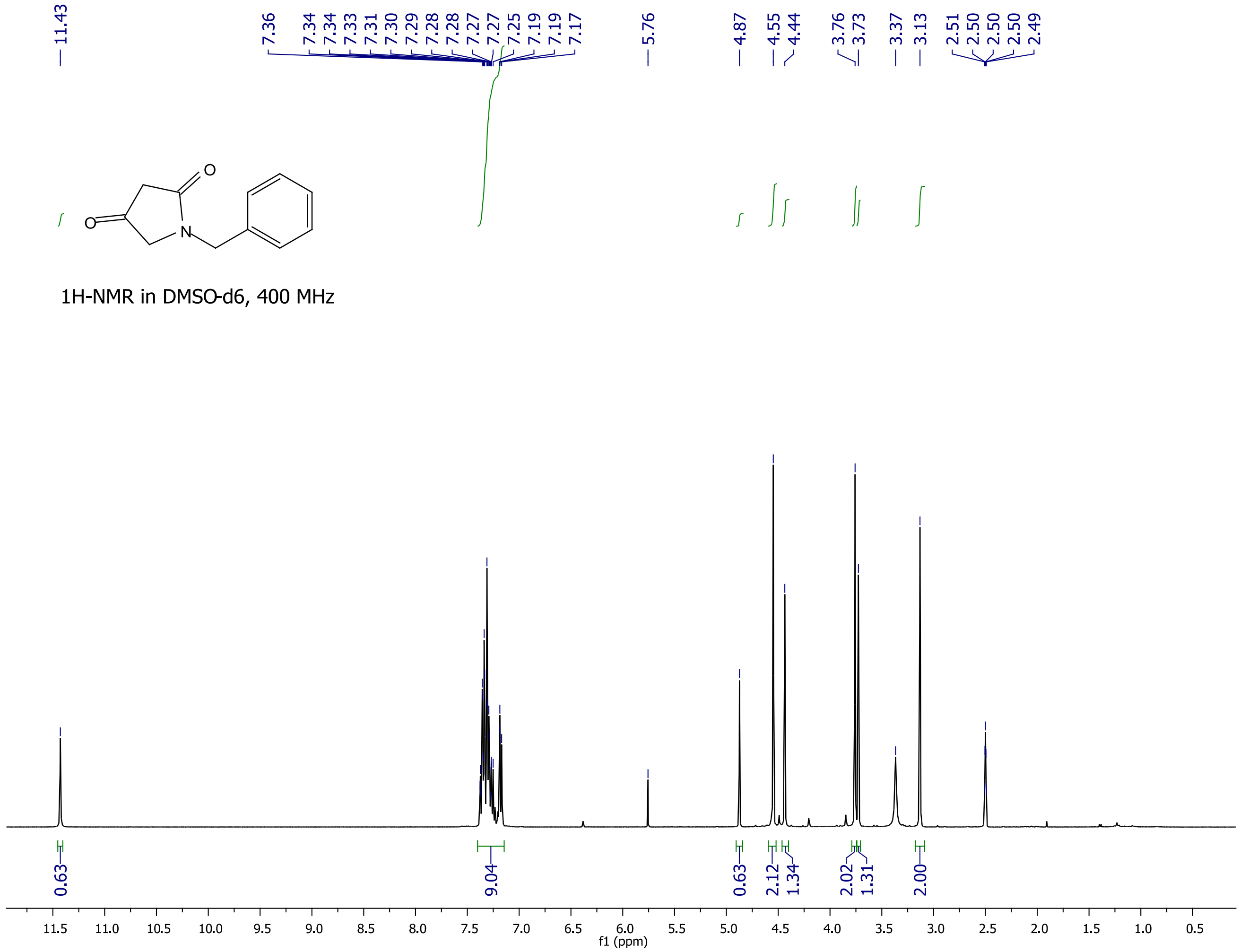
11.43
7.36 7.34 7.34 7.33 7.31 7.30 7.29 7.28 7.28 7.27 7.27 7.25 7.19 7.19 7.17
5.76
4.87 4.55 4.44
3.76 3.73
3.37 3.13
2.51 2.50 2.50 2.50 2.49
1H-NMR in DMSO-d6, 400 MHz
0.63
9.04
0.63 2.12 1.34
2.02 1.31
2.00
11.5 11.0 10.5 10.0 9.5 9.0 8.5 8.0 7.5 7.0 6.5 6.0 5.5 5.0 4.5 4.0 3.5 3.0 2.5 2.0 1.5 1.0 0.5
f1 (ppm)

(L)

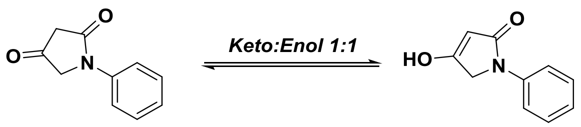

Keto:Enol 1:1
HO

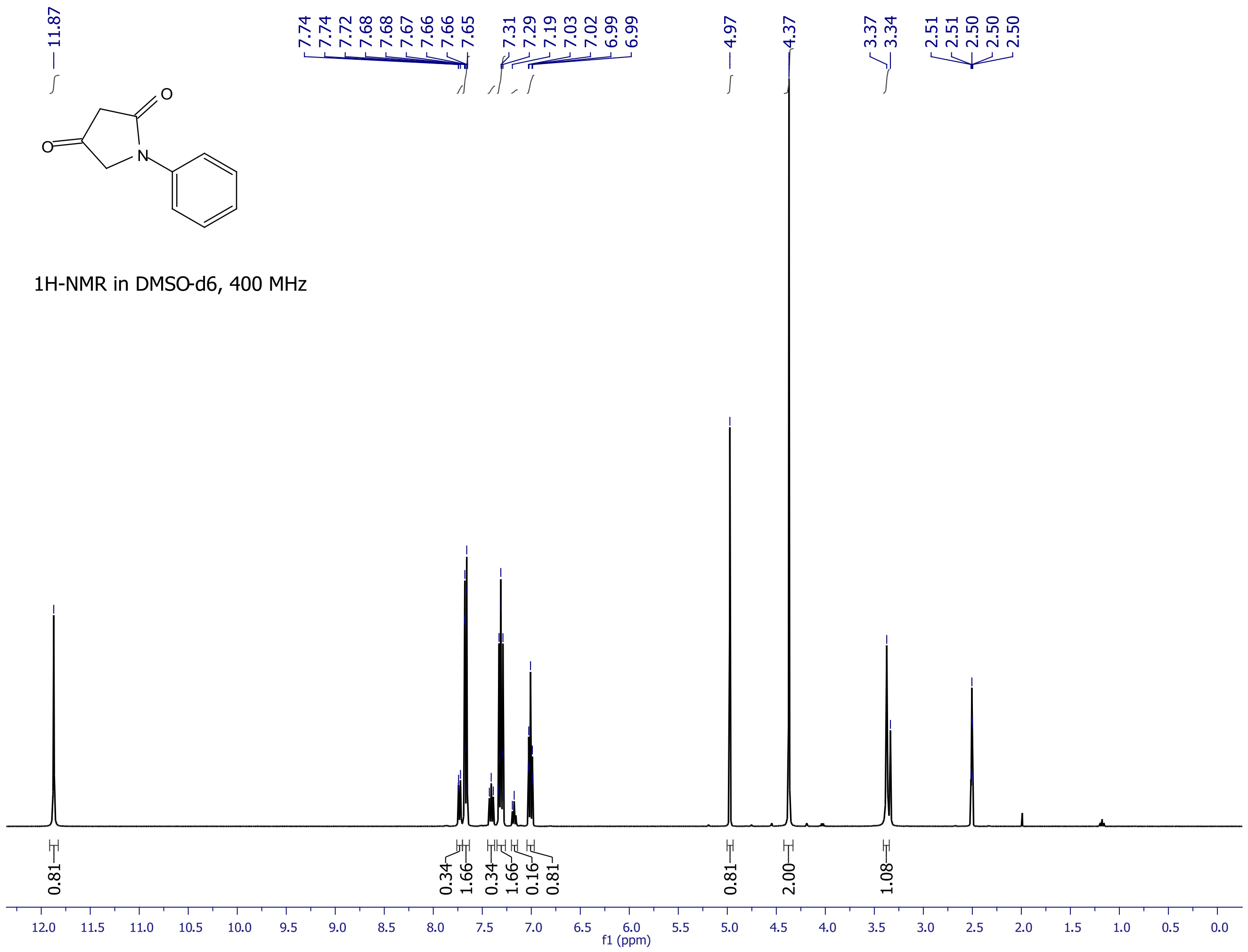

1H-NMR in DMSO-d6, 400 MHz
11.87
7.74
7.74
7.72
7.68
7.68
7.67
7.66
7.66
7.65
7.31
7.29
7.19
7.03
7.02
6.99
6.99
4.97
4.37
3.37
3.34
2.51
2.51
2.50
2.50
2.50
0.81
0.34
1.66
0.34
1.66
0.16
0.81
0.81
2.00
1.08
f1 (ppm)
